# Integrative Mendelian randomization and experimental validation prioritize KLF4 in the gut microbiota–pyroptosis–barrier axis of ulcerative colitis

**DOI:** 10.3389/fimmu.2026.1773990

**Published:** 2026-03-16

**Authors:** Wenya Zhu, Xiaoxu Jin, Xiaofeng Guo, Xin Gao

**Affiliations:** 1 Department of Geriatric, Chinese People's Liberation Army (PLA) General Hospital, The Sixth Medical Center, Beijing, China; 2 Postdoctoral Research Mobile Station of Clinical Medicine, The Second Hospital of Hebei Medical University, Shijiazhuang, China; 3 Department of Gastroenterology, The Second Hospital of Hebei Medical University, Hebei Key Laboratory of Gastroenterology, Hebei Institute of Gastroenterology, Hebei Clinical Research Center for Digestive Diseases, Shijiazhuang, Hebei, China; 4 Department of Gastroenterology, Shanxi Provincial People’s Hospital, Taiyuan, Shanxi, China

**Keywords:** bioinformatics, GSDMD, inflammatory bowel disease, intestinal inflammation, KLF4

## Abstract

**Background:**

Ulcerative colitis (UC) arises from complex crosstalk between gut microbiota, epithelial barrier integrity, and inflammatory cell death, yet causal mediators along this axis remain poorly defined. We aimed to delineate microbiota–pyroptosis–UC pathways and functionally validate key effectors, with a focus on KLF4.

**Methods:**

A multistage framework integrating genome-wide association studies of gut microbiota (MiBioGen), plasma proteomics (deCODE), and UC (UK Biobank) was constructed to perform two-sample Mendelian randomization (MR). Pyroptosis-related proteins were screened for causal associations with UC, followed by MR of UC-associated microbial taxa on these proteins and two-step mediation analysis. KLF4 was further evaluated using bulk and single-cell transcriptomic datasets, including virtual knockout network perturbation. Its clinical relevance was tested in two cohorts of UC patients receiving anti-TNF-α therapy. Finally, the KLF4 function was validated in a dextran sulfate sodium (DSS)-induced colitis model with systemic AAV9-mediated KLF4 overexpression.

**Results:**

MR identified 35 pyroptosis-related plasma proteins and 23 microbial taxa with putative causal effects on UC. Mediation analysis highlighted MAPK11, PTEN, and KLF4 as dominant intermediates linking specific taxa to UC risk. KLF4 was consistently downregulated in UC, and low KLF4 expression was associated with enrichment of pro-inflammatory and immune-activation signatures. Higher mucosal KLF4 levels predicted response to anti-TNF-α therapy with moderate discriminatory performance. In DSS-induced colitis, KLF4 overexpression mitigated weight loss and disease activity, preserved colon length, improved histology, and reduced myeloperoxidase activity. KLF4 restored Claudin-1, Occludin, and Zo-1; suppressed Claudin-2; decreased intestinal permeability; limited gasdermin-D (GSDMD) cleavage; lowered IL-1β/IL-18 levels; and reshaped splenic leukocyte composition.

**Conclusions:**

Our integrative genetic and experimental data position KLF4 as a central node in a gut microbiota–pyroptosis–barrier axis in UC, supporting KLF4 as a promising biomarker and therapeutic target for the precision management of UC.

## Introduction

Ulcerative colitis (UC) is a chronic inflammatory disorder of the colon, where genetic susceptibility, environmental exposures, dysregulated mucosal immunity, and gut microbiota alterations converge to drive persistent mucosal injury and a relapsing–remitting disease course (1, 2). Despite therapeutic advances, a substantial proportion of patients prove refractory to treatment or experience disease complications, underscoring a critical need to decipher the fundamental causal pathways that could be targeted for intervention (1).

Mounting evidence implicates gut microbial dysbiosis in UC pathogenesis, yet distinguishing causal drivers from correlative bystanders has remained a central challenge. The emergence of large-scale genome-wide association studies (GWASs) for gut microbiome composition, such as the MiBioGen consortium’s effort in 18,340 individuals, now provides a powerful framework to interrogate microbe–host interactions using genetic proxies (3). Concurrently, large-scale plasma proteomic GWASs have begun mapping the genetic architecture of thousands of circulating proteins, offering a window into potential mechanistic intermediates between genetic variation and disease (4). Together, these resources enable Mendelian randomization (MR) analyses to assess whether genetically predicted variation in microbial abundance or plasma protein levels causally influences UC risk (5, 6).

The central question remains: by what specific mechanisms do gut microbes exert their effect? Strong evidence shows that microbial signals directly regulate the execution of intestinal pyroptosis. Canonically, dysbiosis heightens NLRP3–caspase-1 signaling, driving gasdermin-D (GSDMD) pore formation and release of IL-1β/IL-18, thereby amplifying mucosal injury. In mouse colitis, NLRP3 activation aggravates epithelial damage and inflammatory readouts, whereas restraining this pathway mitigates disease severity (7, 8). Consistently, epithelial GSDMD-dependent pyroptosis has been shown to worsen chronic colitis *in vivo*, underscoring pyroptosis as an effector arm linking innate sensing to barrier breakdown (9). This underscores pyroptosis as a critical effector mechanism linking host–environment interactions to barrier breakdown.

Guided by this mechanistic backdrop, we postulated that specific gut taxa impact UC risk by modulating pyroptosis-related circulating proteins. To test this, we applied an integrated MR framework combining microbiome GWASs, plasma proteomic protein quantitative trait loci (pQTLs), and UC GWASs. This approach allowed us to first infer putative microbe–protein–UC pathways and then to biologically ground these findings by prioritizing KLF4—an epithelial transcription factor tightly linked to barrier programs—for direct validation in a murine colitis model.

## Methods

### Study design and overview

This study aimed to assess whether gut microbiota influence UC risk through the regulation of pyroptosis-related circulating proteins, using a multistage MR framework. Summary-level data were integrated from GWASs of microbial taxa, plasma proteomics, and UC diagnosis. A four-step MR pipeline was applied: 1) identification of pyroptosis-related plasma proteins and their pQTLs, 2) MR screening of microbial taxa associated with UC, 3) microbe-to-protein MR analysis, and 4) mediation analysis to quantify indirect effects via pyroptosis proteins.

### Pyroptosis-related plasma protein instrument selection

Pyroptosis-associated genes (n = 874) were extracted from GeneCards (https://www.genecards.org/) using “pyroptosis” as the search term. This broad, inclusive criterion was intentionally selected during the initial screening phase to capture a comprehensive functional landscape, encompassing both canonical inflammasome components and peripheral regulatory effectors that may be omitted by strict pathway-based curations. A total of 4,907 protein-level pQTL summary statistics were downloaded from the deCODE Icelandic population cohort. Genetic instruments were defined using the following thresholds: p < 5 × 10^−8^, clumping window of 10 Mb, and linkage disequilibrium (LD) threshold r^2^ < 0.1. After deduplication, 4,759 pQTL entries were retained, corresponding to 4,536 unique protein-coding genes. Then, these gene symbols were intersected with plasma proteins measured using the deCODE genetics proteomics panel (10). Using gene name matching, 237 proteins were identified as both pyroptosis-related and pQTL-available. These were retained for downstream MR analyses.

### Gut microbiota GWAS and exposure instrument selection

Microbial GWAS summary statistics were obtained from the MiBioGen consortium (11), comprising 18,340 individuals of European descent. A total of 473 taxa from the phylum to genus level were included. Genetic instruments for each taxon were filtered at a suggestive significance level (p < 5 × 10^−6^) and LD-clumped (r^2^ < 0.1, 10-Mb window).

### Ulcerative colitis GWAS summary statistics

GWAS summary statistics for UC were obtained from UK Biobank-based fastGWA analysis (PheCode 555.2), as reported in Yang et al. (12). The dataset included 456,348 individuals of European ancestry and was processed using linear mixed models. Single-nucleotide polymorphism (SNP) positions were aligned to GRCh37 (hg19).

### MR of pyroptosis proteins and microbial taxa on UC

For each of the 237 pyroptosis-related proteins, two-sample MR was performed using their pQTLs as instruments and UC GWAS summary statistics as outcomes. Instruments were harmonized and filtered using harmonise_data in the TwoSampleMR R package (13). Steiger filtering was applied to retain variants with correct causal direction. Inverse-variance weighted (IVW) was used as the primary MR method, with weighted median and MR-Egger as sensitivity checks. Proteins with IVW p < 0.05 were considered UC-related. A total of 35 proteins met this criterion and were advanced to mediation modeling. Each of the 473 microbial traits was tested against UC using the same MR procedure. Given the large-scale screening nature of this analysis, these nominal associations were explicitly utilized as an exploratory discovery framework to prioritize a restricted set of robust candidates for subsequent *in vivo* validation. After filtering, 23 microbial taxa demonstrated significant associations with UC (IVW p < 0.05). These were selected as candidate upstream exposures in subsequent analyses.

### MR of microbial taxa on pyroptosis proteins

To assess whether UC-associated microbes influence pyroptosis biology, pairwise MR was conducted between the 23 UC-related microbial taxa and the 35 UC-associated pyroptosis proteins (805 tests in total). Exposure and outcome data were harmonized and filtered as above. IVW, pleiotropy (MR-Egger intercept), heterogeneity (Cochran’s Q), and sensitivity analyses (leave-one-out, funnel, and forest plots) were conducted. All result sets were retained regardless of significance for comprehensive mediation modeling.

### Two-step MR mediation analysis

Two-step MR was performed to assess whether pyroptosis-related proteins mediate the causal relationship between gut microbiota and UC. For each microbe–protein–UC triplet, three estimates were calculated: the total effect of the gut microbe on UC (beta_total), the effect of the microbe on the protein (beta1), and the effect of the protein on UC (beta2). The mediated effect (beta12) was calculated as beta1 × beta2, with its standard error estimated using the delta method. The statistical significance of the mediation was assessed using a Z-test (Z = beta12/SE), and the corresponding p-value (p_beta12) was computed. The mediation proportion was estimated as beta12/beta_total.

Given the binary nature of the UC outcome (assessed on a log odds scale), the calculated mediation proportion was interpreted conceptually as a relative ranking metric to prioritize top biological intermediates for downstream experimental validation, rather than as an absolute biological quantification of the indirect effect.

### KLF4 expression and functional validation analyses

The relevance of KLF4, one of the top microbial-mediated pyroptosis genes, was further validated through a series of transcriptomic and computational functional analyses. Differential expression of KLF4 was assessed in UC patients from two microarray datasets, GSE107499 and GSE47908 (14), using the limma R package (15) to compare expression between UC and healthy controls.

To explore downstream biological effects of KLF4, UC patients were stratified into the high and low KLF4 expression subgroups based on median expression. Differential gene expression analysis between these subgroups was performed using limma, followed by Gene Set Enrichment Analysis (GSEA) using the Hallmark gene sets from the MSigDB database (16). Normalized enrichment scores (NESs) and adjusted p-values were used to assess significance.

To predict KLF4-driven network alterations at single-cell resolution, the scTenifoldKnk algorithm (17) on GSE125527, a single-cell RNA-seq dataset from UC patients, was utilized (18). Genes significantly perturbed after virtual knockout of KLF4 were then subjected to Gene Ontology (GO) enrichment analysis.

### Assessment of KLF4 expression in anti-TNF-α treatment response

To evaluate the clinical relevance of KLF4 expression in UC patients receiving anti-TNF-α therapy, two publicly available transcriptomic datasets, GSE16879 (19) and GSE12251 (20), were analyzed. In each cohort, samples were stratified into responders and non-responders based on clinical annotation. KLF4 expression levels were compared between groups. Additionally, receiver operating characteristic (ROC) curve analysis was performed using the pROC package to assess the discriminatory capacity of KLF4 expression for treatment response prediction. The area under the curve (AUC) was used as a summary measure of predictive performance.

### Systemic KLF4 overexpression and colitis model

Specific pathogen-free C57BL/6J mice (6–8 weeks, sex-matched) were housed under a 12-h light/dark cycle with food and water *ad libitum*. To achieve body-wide overexpression of KLF4, mice received an intravenous injection of AAV9-KLF4 (control: AAV9-Empty; GENCEFE Biotech, Wuxi, China) via the tail vein (~1 × 10^11^ vg per 20–25 g mouse in 150–200 µL sterile phosphate-buffered saline (PBS)) 7 days before dextran sulfate sodium (DSS; MP Biomedicals, Solon, OH, USA) administration. Experimental colitis was induced with 4% (w/v) DSS in drinking water for 7 days (day 0–day 7), and mice were euthanized on day 7 by CO_2_ inhalation (30% chamber volume/min) followed by cervical dislocation to ensure death. Mice were randomly assigned to groups, and clinical scoring and histopathology were performed under blinding.

### Colitis disease activity index and body weight

From day 0 to day 7, mice were monitored daily for body weight, stool consistency, and fecal blood. Each component was scored on a 0–4 scale (weight loss: 0, ≤1%; 1, 1%–5%; 2, 5%–10%; 3, 10%–15%; 4, >15%; stool: 0, normal; 2, loose; 4, diarrhea; blood: 0, negative; 2, Hemoccult-positive; 4, gross bleeding). The Disease Activity Index (DAI) was calculated as the sum of subscores (0–12) (21). Scoring was performed under blinding.

### Assessment of colitis

At day 7, colons were excised from the ileocecal junction to the rectum, gently rinsed, laid flat without stretch, and measured on a calibrated ruler. Distal colon segments were fixed in 4% paraformaldehyde, paraffin-embedded, sectioned at 4 µm, and stained with H&E. Inflammation and tissue injury were graded using the Cooper histological score, averaging ≥5 non-overlapping fields per mouse. Representative whole-section images were acquired at ×40 (scale bar, 100 µm).

### Myeloperoxidase activity

Colon tissues were homogenized on ice and cleared by centrifugation. Myeloperoxidase (MPO) activity was quantified using a commercial MPO assay kit (Nanjing Jiancheng Bioengineering Institute, Nanjing, China) according to the manufacturer’s instructions and reported as U per g tissue.

### Immunohistochemistry of tight junction proteins and H-score

Paraffin sections underwent deparaffinization, citrate-buffer antigen retrieval (pH 6.0, ~95 °C–98°C, 15 min), peroxidase quenching, and 5% bovine serum albumin (BSA) blocking. Sections were incubated overnight (4°C) with primary antibodies against Claudin-1, Claudin-2, Occludin, and Zo-1 (rabbit mAbs; Affinity Biosciences, Cincinnati, OH, USA; working 1:200–1:400), followed by horseradish peroxidase (HRP)-conjugated secondary antibodies, 3,3′-diaminobenzidine (DAB) development, and hematoxylin counterstaining. Images were captured at ×400 under identical exposure. Staining was quantified using the H-score (22).

### Cytokine ELISA

Equal-length distal colon segments were homogenized in ice-cold PBS (1:9, w:v), clarified (12,000 *g*, 10 min, 4°C), and assayed for IL-1β, IL-18, TNF-α, and IL-6 using commercial ELISA kits according to the manufacturer’s instructions (Enova, Wuhan, China). Standard curves were used for quantification; concentrations were reported as pg/mL.

### Western blotting

The distal colon was lysed in radioimmunoprecipitation assay (RIPA) buffer containing protease/phosphatase inhibitors and quantified using bicinchoninic acid (BCA), and equal protein (20–40 μg) was resolved by sodium dodecyl sulfate–polyacrylamide gel electrophoresis (SDS–PAGE) and transferred to polyvinylidene fluoride (PVDF) membranes. After blocking, membranes were probed with antibodies to Claudin-1, Claudin-2, Occludin, Zo-1 (Affinity), total GSDMD, and GSDMD-N (AbioWell, Wuhan, China), and GAPDH as a loading control, followed by HRP-conjugated secondary antibodies and enhanced chemiluminescence (ECL) detection. Band intensities were quantified using ImageJ.

### Intestinal permeability assay

At the study endpoint, intestinal barrier function was assessed using fluorescein isothiocyanate–dextran (FITC–dextran; 4 kDa; Sigma-Aldrich, St. Louis, MO, USA). Mice were fasted for 4 h with water *ad libitum* and then gavaged with FITC–dextran (60 mg per 100 g body weight) under light-protected conditions. Four hours later, blood was collected and allowed to clot, and serum was obtained by centrifugation (12,000 *g*, 5 min, 4°C). Serum FITC fluorescence was measured on a spectrofluorometer (Ex = 490 nm, Em = 520 nm) against a standard curve prepared in blank mouse serum.

### Flow cytometry

Single‐cell suspensions were prepared from the spleen, red cells were lysed, and leukocytes were counted in staining buffer. After Fc block (anti-CD16/32), cells were stained for 20–30 min at 4°C with a surface panel including CD45, CD4, CD8a, CD11b, F4/80, Ly6C, and Ly6G (Elabscience, Houston, TX, USA), washed, and acquired on an Attune NxT flow cytometer (Thermo Fisher, Waltham, MA, USA). Compensation used single-stained controls; fluorescence minus one (FMO) controls were used where indicated. Gating proceeded as singlets → CD45^+^ leukocytes → i) T cells (CD4^+^ and CD8^+^) and ii) myeloid subsets (CD11b^+^F4/80^+^ macrophages, CD11b^+^Ly6C^hi^ monocytes, and CD11b^+^Ly6G^+^ neutrophils). Data were analyzed in FlowJo v10 under blinding, with ≥50,000 events collected per sample.

### Statistical analysis

To rule out weak instrument bias, the F-statistic for each SNP was calculated, with an F-statistic > 10 indicating sufficient instrument strength. Furthermore, Cochran’s Q test was utilized to assess heterogeneity among SNPs, and the MR-Egger intercept was evaluated to detect potential directional pleiotropy. Data are presented as mean ± SD. Two-group comparisons used two-sided unpaired Student’s t-test (Shapiro–Wilk non-normality → Mann–Whitney U). Time-course data (body weight and DAI) were analyzed using two-way repeated-measures ANOVA with the Geisser–Greenhouse correction. p < 0.05 was considered significant. Analyses were performed in R (v4.4.0).

## Results

### Mendelian randomization identifies 35 pyroptosis-related plasma proteins with causal associations with UC

**Figure 1:**
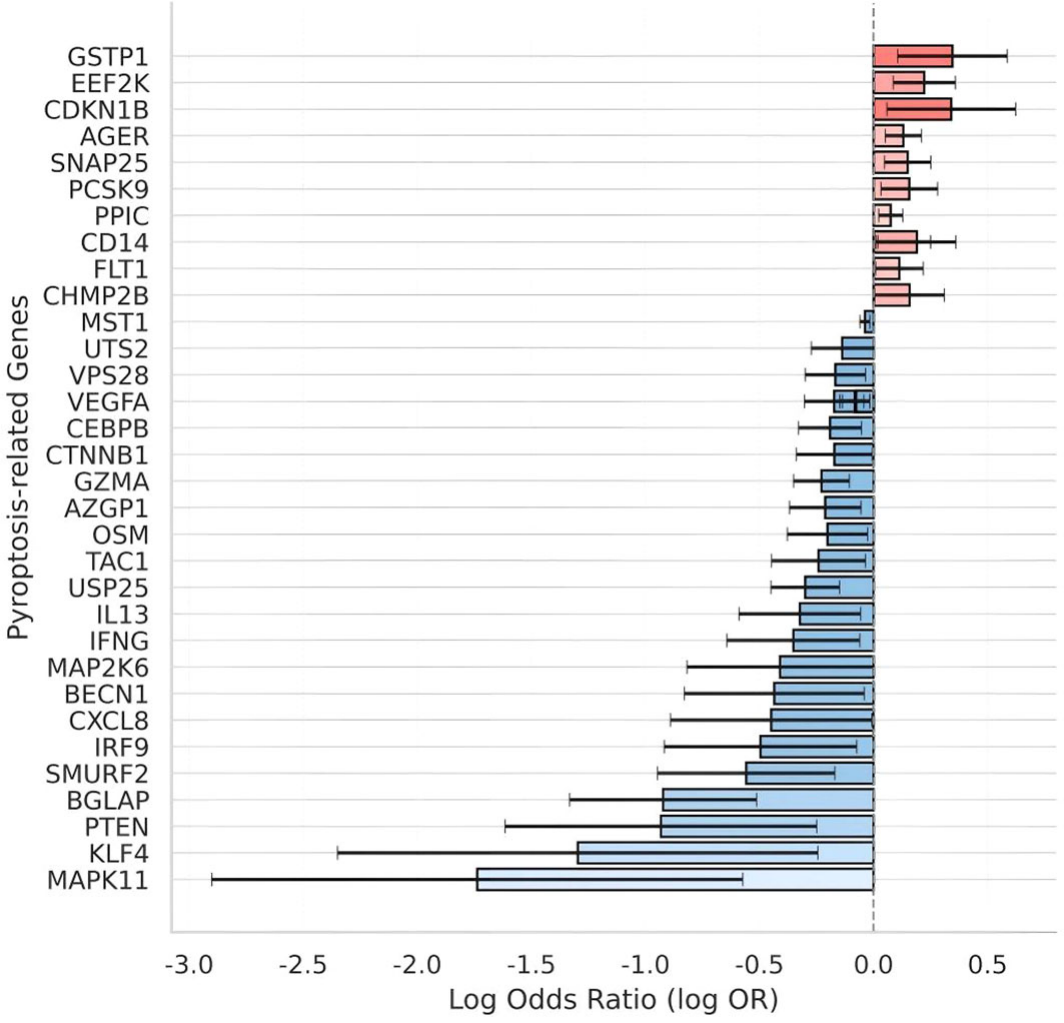
Genetically predicted circulating levels of 35 pyroptosis-related proteins and their associations with UC risk. Shown are the inverse-variance weighted (IVW) Mendelian randomization (MR) estimates for 35 plasma proteins with exploratory nominally significant associations (p < 0.05) with ulcerative colitis (UC). Log odds ratios (logOR) are plotted for each protein, ranked by effect size. Positive logOR indicates increased UC risk, while negative logOR suggests a protective association. Error bars represent standard errors. All proteins were selected from a set of 237 genetically instrumented pyroptosis-related candidates. Data sources, harmonization details, and sensitivity analyses are provided in **Supplementary Data Sheet 1**.

To evaluate whether genetically predicted levels of pyroptosis-related circulating proteins are associated with UC, we performed two-sample MR analysis using 237 plasma proteins previously identified as both pyroptosis-related and genetically instrumented. A total of 35 proteins exhibited exploratory nominally significant causal associations with UC (IVW p < 0.05). As shown in **Figure 1**, we visualized the log odds ratios (logOR) for these 35 proteins, ordered by effect size. Notably, a majority (27/35) showed protective directions. Among the strongest protective associations were MAPK11, KLF4, and PTEN, all with logOR < −1.0. Full harmonized summary data and forest/funnel plots are provided in **Supplementary Data Sheet 1**.

### Mendelian randomization identifies 23 gut microbial taxa with causal associations with UC

**Figure 2:**
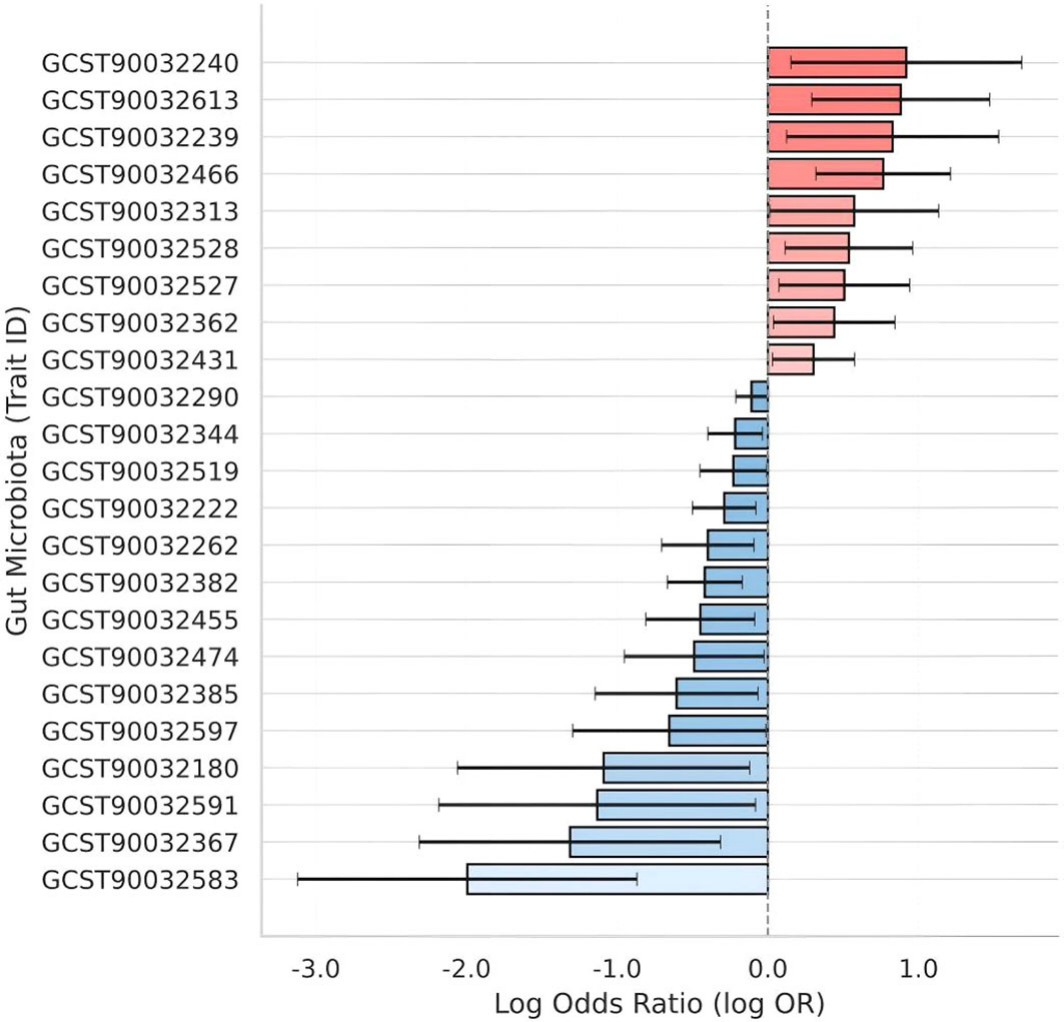
Genetically predicted gut microbial traits associated with ulcerative colitis (UC) risk. Inverse-variance weighted Mendelian randomization (MR) estimates (logOR ± s.e.) for 23 microbial traits that met a nominal significance threshold (p < 0.05). Bars are colored by effect direction: blue, protective (logOR < 0; n = 14); red, risk-enhancing (logOR > 0; n = 9). Trait labels are shown as MiBioGen GCST identifiers; taxonomic names are listed in **Supplementary Data Sheet 2**.

A two-sample Mendelian randomization scan across 473 MiBioGen taxa identified 23 microbial traits whose genetically predicted abundances were nominally associated with UC (IVW p < 0.05). As illustrated in **Figure 2**, nine taxa displayed positive log odds ratios (risk-enhancing), whereas 14 taxa showed negative log odds ratios (protective). Full GWAS Catalog study accession (GCST) identifiers, taxonomic labels, and MR statistics are provided in **Supplementary Data Sheet 2**.

### Gut microbiota–pyroptosis–UC axis: MAPK11, KLF4, and PTEN as dominant mediators

To determine whether gut microbiota modulate UC via pyroptosis-related proteins, we performed 805 pairwise MR tests between 23 UC-associated microbial taxa and 35 UC-associated pyroptosis proteins (**Supplementary Data Sheet 3**). We retained all results for downstream mediation analysis.

Using a two-step MR framework, we identified 31 microbe–protein–UC triplets with mediation proportions exceeding 10% (**Table 1**), suggesting biologically meaningful indirect pathways. As summarized in **Table 1**, these events were predominantly mediated by three proteins—MAPK11, KLF4, and PTEN. We observed the strongest mediation for the CAG-495 → MAPK11 → UC pathway (51.7%). Additional microbes, including UBA1066, *Prevotella*, and *Leuconostoc*, also exhibited indirect associations with UC via these proteins. These findings highlight MAPK11, KLF4, and PTEN as key intermediaries linking microbial signals to pyroptosis in the context of UC. Further, we *a priori* prioritized KLF4 given its established role in epithelial barrier biology and an emerging link to inflammasome-driven pyroptosis.

**Table 1:** Mediation analysis of gut microbiota–pyroptosis–UC axis.

Microbial taxa	Protein	Path a (β_1_)	Path b (β_2_)	β_12_ (indirect effect)	β_12__p (proportion mediated)
CAG-495	MAPK11	0.032	−1.737	−0.056	51.7%
Eremiobacterota	MAPK11	0.357	−1.737	−0.621	47.3%
UBA1066 sp900317515	MAPK11	0.271	−1.737	−0.471	41.6%
CAG-495	KLF4	0.024	−1.297	−0.032	29.2%
UBA11963	KLF4	0.131	−1.297	−0.17	26.0%
UBA1066 sp900317515	PTEN	0.273	−0.932	−0.255	22.5%
Borreliaceae	MAPK11	−0.099	−1.737	0.173	20.9%
*Prevotella* sp000436915	PTEN	0.048	−0.932	−0.044	19.4%
*Prevotella* sp000436915	KLF4	0.033	−1.297	−0.043	19.0%
UBA1066 sp900317515	KLF4	0.144	−1.297	−0.186	16.5%
Faecalicatena sp002161355	PTEN	0.107	−0.932	−0.099	16.4%
*Leuconostoc*	MAPK11	−0.068	−1.737	0.118	15.4%
*Desulfovibrio piger*	PTEN	0.035	−0.932	−0.033	15.1%
*D. piger*	MAPK11	0.018	−1.737	−0.032	14.7%
Megasphaera sp900066485	PTEN	0.076	−0.932	−0.071	14.5%
*Provencibacterium*	KLF4	−0.059	−1.297	0.077	14.2%
*Provencibacterium massiliense*	KLF4	−0.049	−1.297	0.063	12.4%
UBA11963	PTEN	0.082	−0.932	−0.076	11.7%

UC, ulcerative colitis.

### KLF4 is downregulated in UC and associated with immune-related signaling

**Figure 3:**
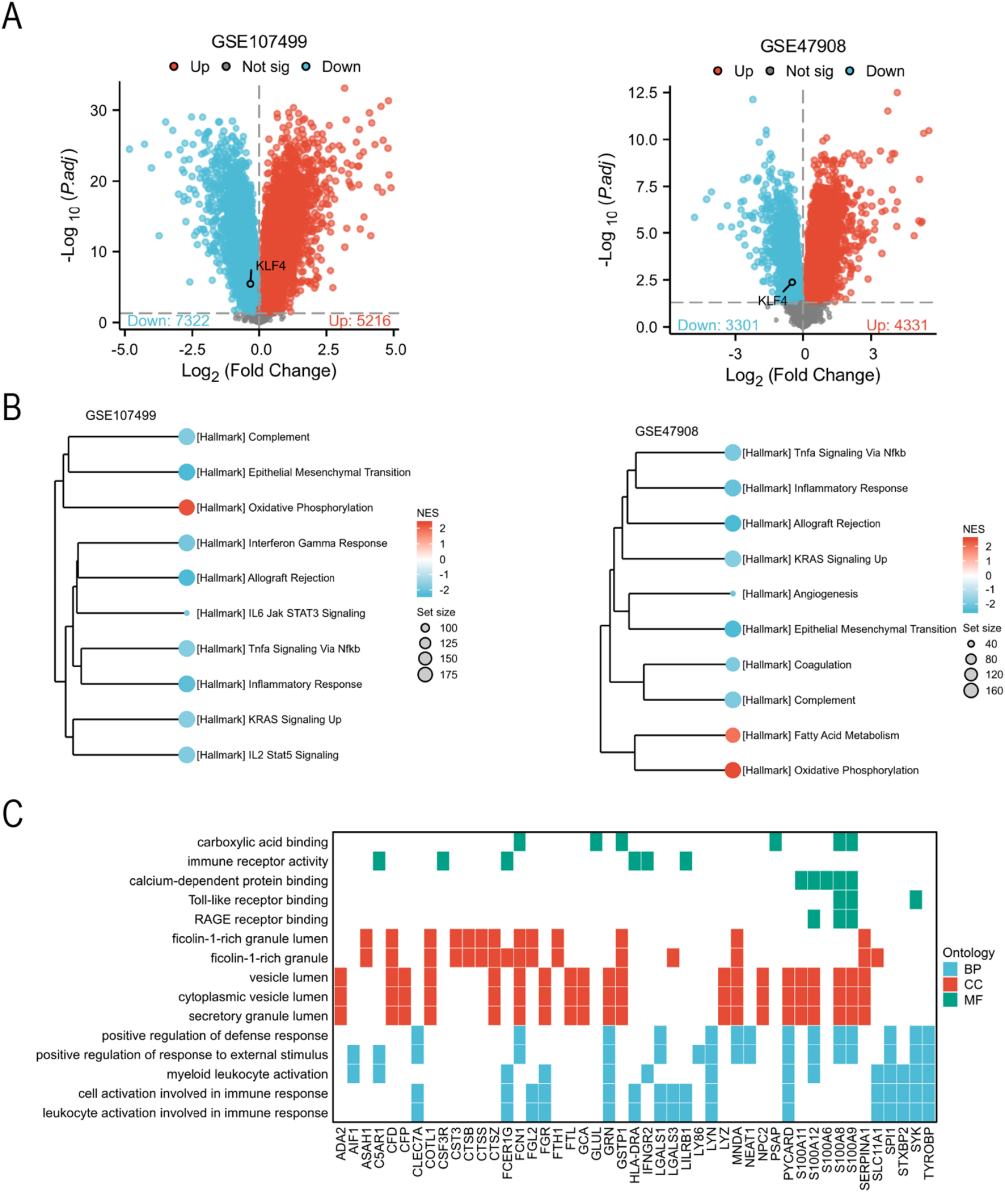
KLF4 is downregulated in ulcerative colitis and regulates immune-related pathways. **(A)** Volcano plots showing differential gene expression between ulcerative colitis (UC) patients and healthy controls in two transcriptomic datasets (GSE107499 and GSE47908). KLF4 is significantly downregulated in both datasets. **(B)** Gene Set Enrichment Analysis (GSEA) of Hallmark pathways comparing UC patients with high *vs*. low KLF4 expression. Enrichment scores (NESs) are visualized, with immune-related pathways prominently enriched in the low KLF4 group. **(C)** Gene Ontology (GO) enrichment analysis of differentially expressed genes following *in silico* KLF4 knockout simulation using scTenifoldKnk in GSE125527.

To explore the functional implications of KLF4 in UC, we examined its expression in two independent bulk transcriptomic datasets. KLF4 was significantly downregulated in UC patients compared to healthy controls in both the GSE107499 and GSE47908 cohorts (**Figure 3A**). We next stratified UC samples into the high and low KLF4 expression groups and conducted GSEA using the Hallmark gene sets. Low KLF4 expression was associated with enrichment of pro-inflammatory and immune-related pathways, including interferon gamma response, TNFα signaling via NF-κB, and IL6–JAK–STAT3 signaling (**Figure 3B**). To further assess functional consequences of KLF4 loss, we performed *in silico* knockout simulation using scTenifoldKnk on single-cell transcriptomes of UC (GSE125527). GO enrichment of the perturbed transcriptome revealed prominent activation of neutrophil degranulation, leukocyte activation, and secretory granule function (**Figure 3C**), suggesting that KLF4 may regulate inflammatory immune responses in UC.

### KLF4 expression predicts anti-TNF-α treatment response in UC patients

**Figure 4:**
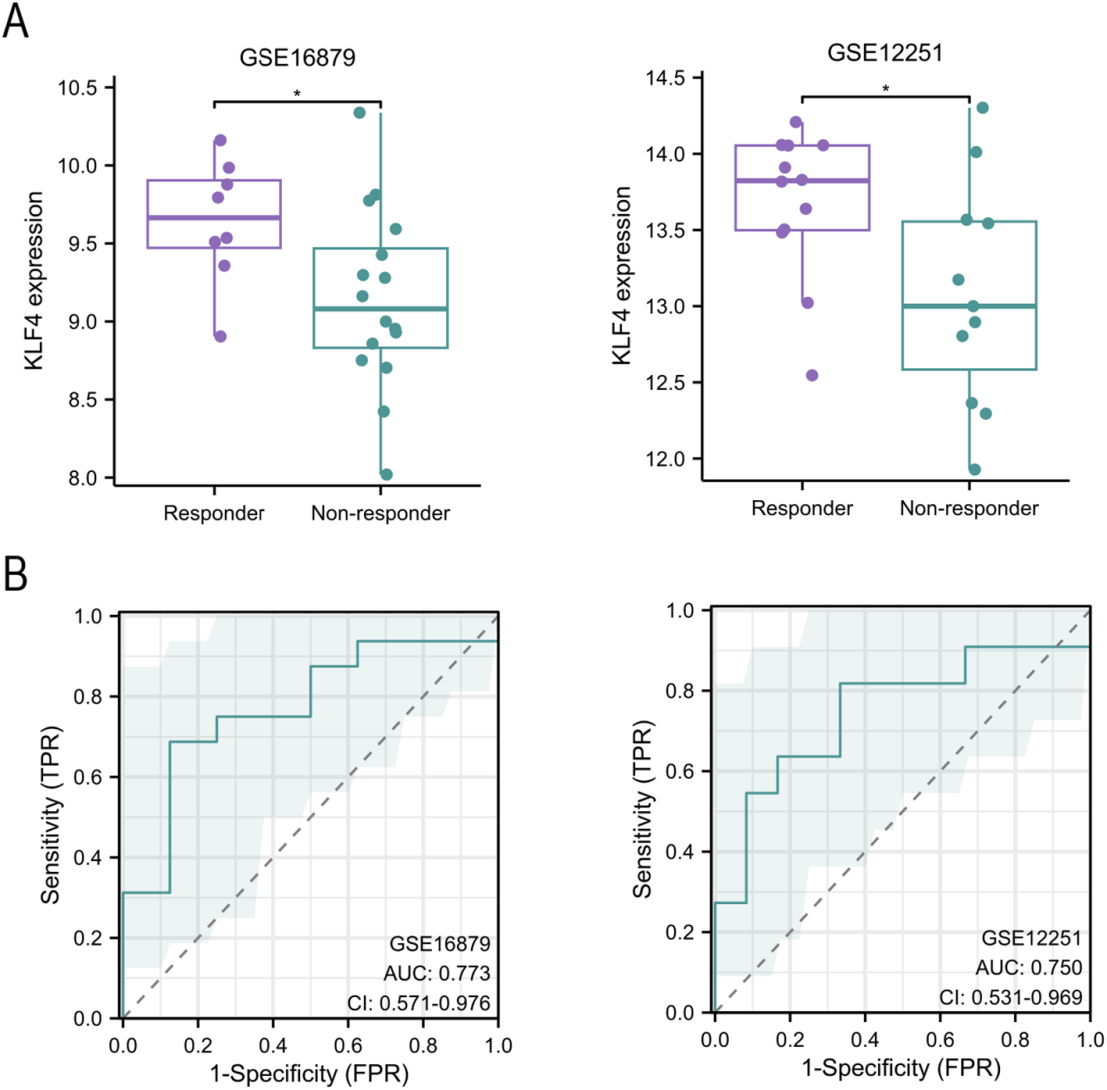
KLF4 expression is associated with anti-TNF-α treatment response in ulcerative colitis (UC). **(A)** Box plots comparing KLF4 expression between responders and non-responders to anti-TNF-α therapy in GSE16879 and GSE12251 datasets. **(B)** Receiver operating characteristic (ROC) curves evaluating the predictive performance of KLF4 for treatment response.

To determine whether KLF4 expression is associated with therapeutic outcomes, we analyzed its levels in patients stratified by response to anti-TNF-α therapy in two independent datasets. In both GSE16879 and GSE12251, KLF4 expression was significantly higher in responders than in non-responders (**Figure 4A**). ROC analysis further demonstrated that KLF4 has moderate predictive performance for treatment response, with AUC values of 0.773 (95% CI: 0.571–0.976) and 0.750 (95% CI: 0.531–0.969) in GSE16879 and GSE12251, respectively (**Figure 4B**). These results support the potential utility of KLF4 as a biomarker for anti-TNF-α therapy responsiveness in UC.

### KLF4 overexpression ameliorates DSS-induced colitis severity

**Figure 5:**
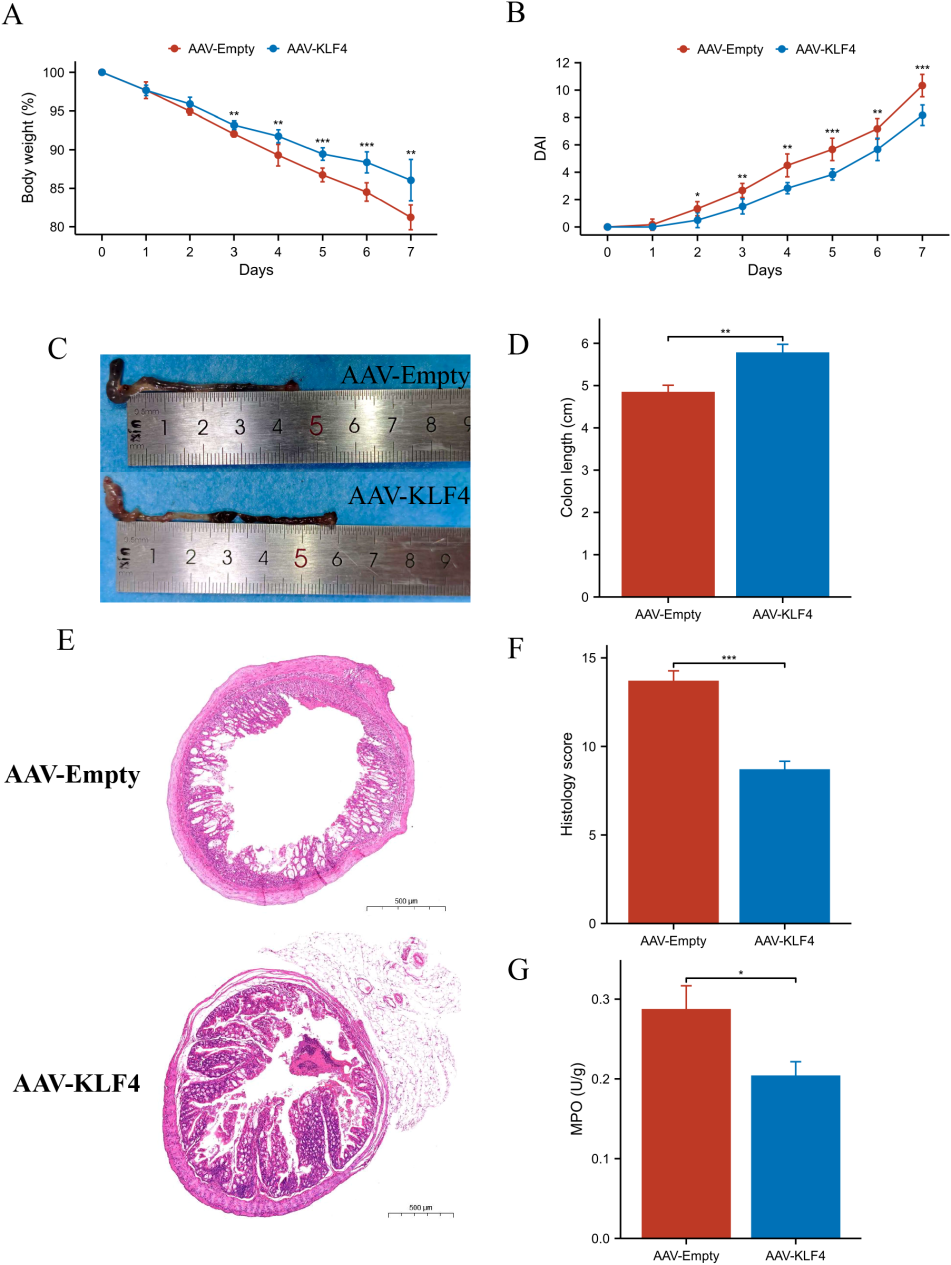
Systemic KLF4 overexpression attenuates dextran sulfate sodium (DSS)-induced colitis. **(A)** Body-weight trajectories during DSS (percent of day 0). **(B)** Disease Activity Index (DAI) over time. **(C)** Representative gross colons at necropsy. **(D)** Quantified colon length. **(E)** Representative H&E images of distal colon. **(F)** Histology scores. **(G)** Myeloperoxidase (MPO) activity (U/g). Symbols/asterisks indicate between-group significance as shown in the plots. N = 6 mice per group. Data are representative of two independent experiments.

Mice receiving AAV-KLF4 lost less body weight than AAV-Empty controls during DSS exposure, with significant differences on multiple days (**Figure 5A**). DAI rose in both groups but was consistently lower in AAV-KLF4 from ~day 3 onward, with several time points reaching significance (**Figure 5B**). Grossly, colons were longer in AAV-KLF4 mice (**Figures 5C, D**). Representative H&E sections illustrated milder epithelial injury and inflammatory infiltration in AAV-KLF4 compared with AAV-Empty (**Figure 5E**), and histology scores were significantly reduced (**Figure 5F**). MPO activity was also lower in AAV-KLF4 (**Figure 5G**).

### KLF4 preserves epithelial barrier markers and reduces GSDMD cleavage

**Figure 6:**
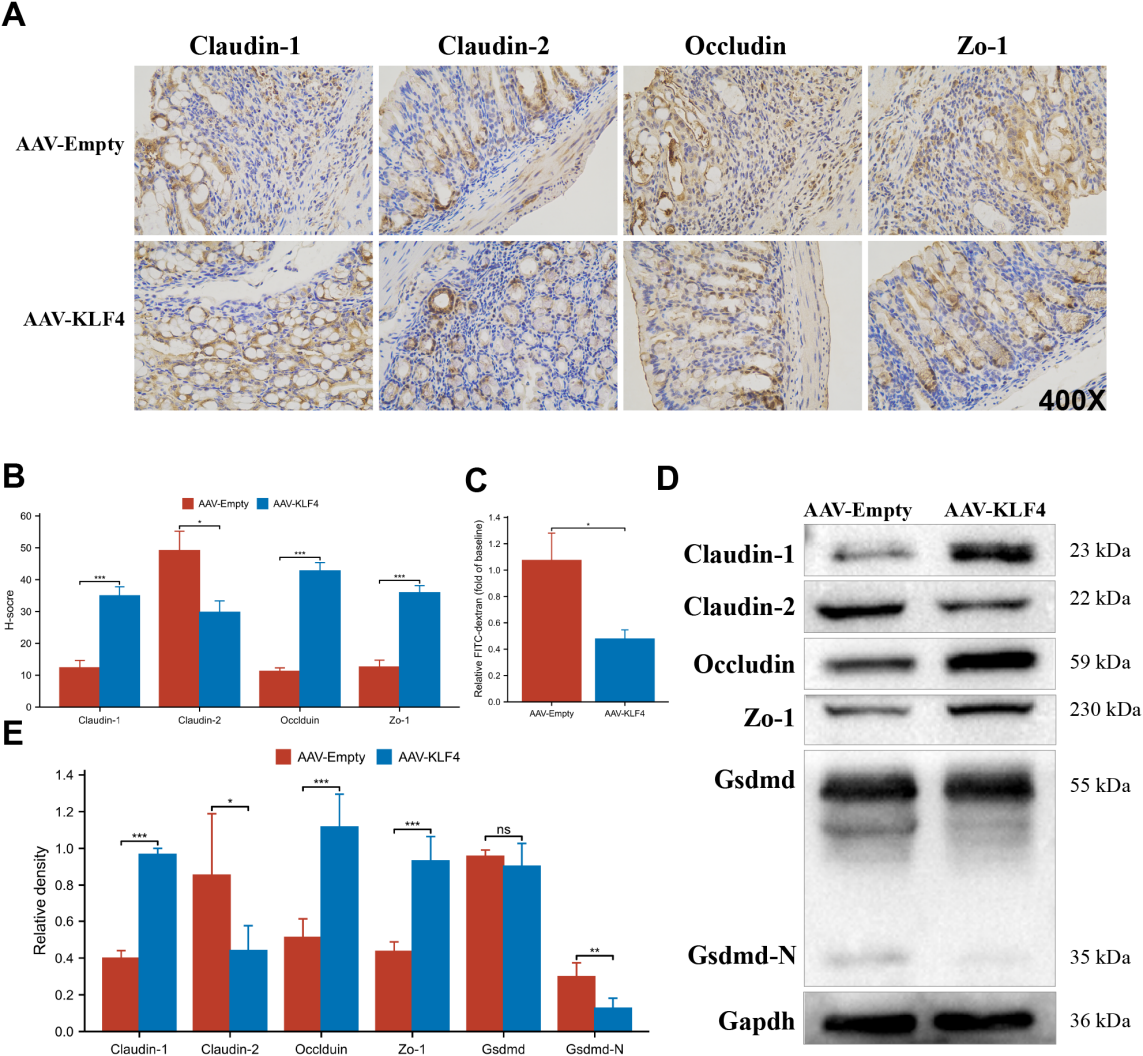
KLF4 strengthens epithelial barrier markers, lowers permeability, and reduces gasdermin-D (GSDMD) cleavage. **(A)** Representative immunohistochemistry (IHC) for Claudin-1, Claudin-2, Occludin, and Zo-1 (×400); top, AAV-Empty; bottom, AAV-KLF4. **(B)** H-score quantification of IHC. **(C)** Serum fluorescein isothiocyanate (FITC)–dextran after gavage (relative fold to baseline). **(D)** Immunoblots for tight junction proteins, total GSDMD, and GSDMD-N; GAPDH, loading control; expected molecular masses indicated. **(E)** Densitometric quantification (normalized to GAPDH). Asterisks denote significant differences; “ns”, not significant. N = 6 mice per group. Data are representative of two independent experiments.

Immunohistochemistry (IHC) showed stronger membranous staining of Claudin-1, Occludin, and Zo-1 in AAV-KLF4, whereas Claudin-2 staining was weaker compared to AAV-Empty (**Figure 6A**). Quantification using the H-score confirmed higher Claudin-1, Occludin, and Zo-1 and lower Claudin-2 with KLF4 (**Figure 6B**). FITC–dextran permeability was reduced in AAV-KLF4 (**Figure 6C**). By immunoblot, Claudin-1, Occludin, and Zo-1 bands were increased and Claudin-2 decreased in AAV-KLF4 (**Figure 6D**), with densitometry mirroring the IHC (**Figure 6E**). Total GSDMD did not differ significantly, whereas the cleaved GSDMD-N fragment was reduced in AAV-KLF4 (**Figures 6D, E**).

### KLF4 alters splenic immune profile and reduces colonic cytokines

**Figure 7:**
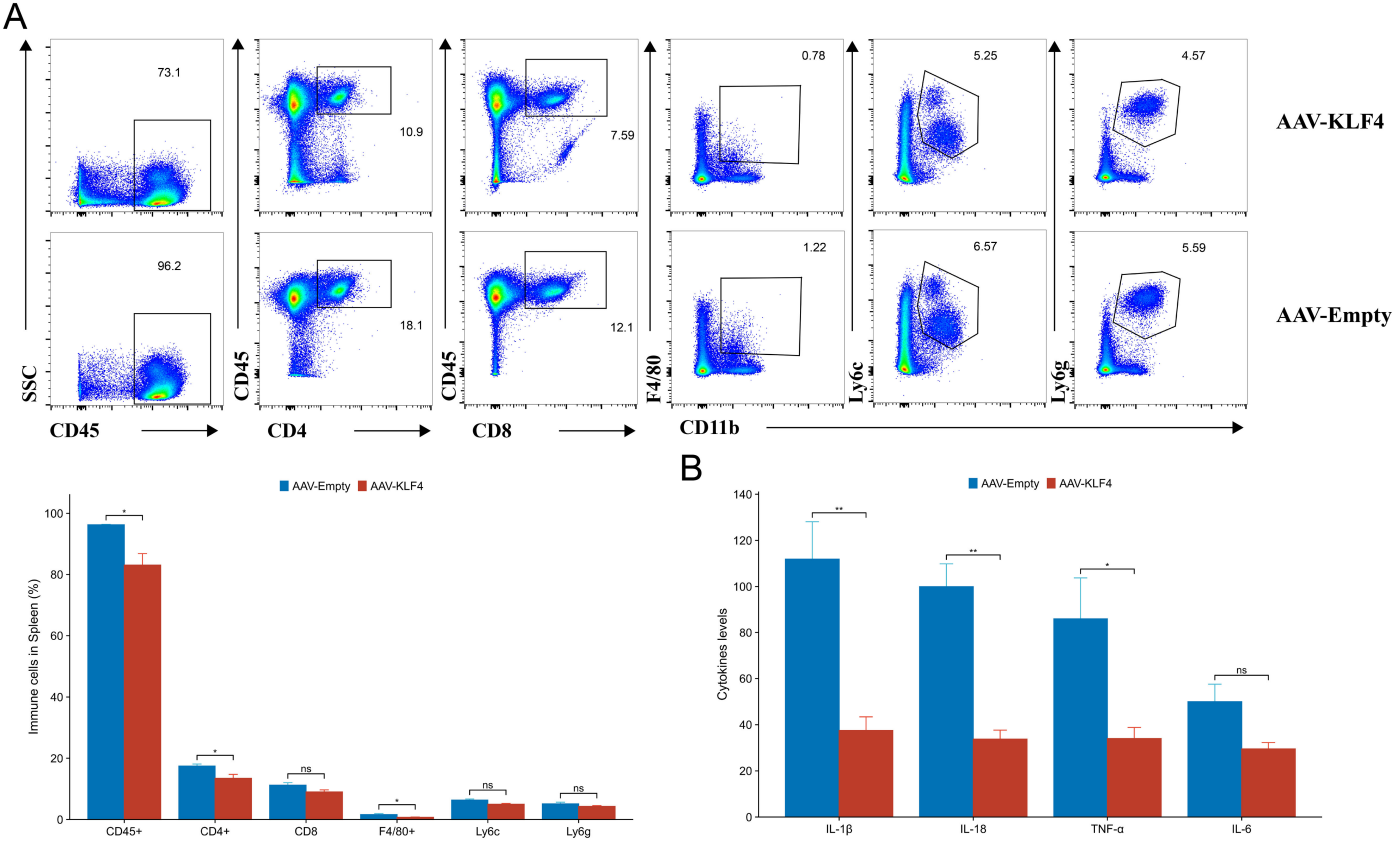
KLF4 reshapes splenic leukocyte composition and reduces inflammatory cytokines. **(A)** Flow cytometry gating (top row, AAV-KLF4; bottom row, AAV-Empty) and summary bar graph of splenic CD45^+^, CD4^+^, CD8^+^, F4/80^+^, Ly6C^+^, and Ly6G^+^ populations (percent of live singlets). **(B)** Cytokine concentrations (pg/mL) in colon tissue homogenates (IL-1β, IL-18, TNF-α, and IL-6). Statistical annotations as displayed on the plots: “ns”, not significant. N = 6 mice per group. Data are representative of two independent experiments.

Flow cytometry of the spleen showed lower CD45^+^ leukocyte frequency in AAV-KLF4, with a reduction in CD4^+^ T cells and F4/80^+^ myeloid cells; CD8^+^ T cells, Ly6C^+^, and Ly6G^+^ subsets did not differ significantly (**Figure 7A**). Cytokines measured from colon homogenates by ELISA were lower for IL-1β and IL-18 in AAV-KLF4 (both significant), with a modest reduction in TNF-α and a non-significant trend for IL-6 (**Figure 7B**). Collectively, these data suggest that systemic KLF4 overexpression may ameliorate DSS-induced colitis, potentially by limiting GSDMD cleavage and pyroptosis, thereby lowering IL-1β/IL-18 levels and reducing splenic leukocyte proportions.

## Discussion

In this study, we aimed to assess the causal role of gut microbiota in ulcerative colitis through the regulation of pyroptosis-related proteins, specifically KLF4, which is crucial for epithelial barrier integrity. Our findings underscore the significant interplay between the gut microbiota and inflammatory cell death pathways in UC, with implications for therapeutic strategies targeting microbial communities and epithelial barrier function.

First, MR analysis was conducted using large-scale GWAS data on microbial taxa, plasma proteomics, and UC diagnosis. Our results identified 35 pyroptosis-related proteins with causal associations with UC, among which KLF4, MAPK11, and PTEN were highlighted for their protective roles in UC. KLF4 was selected for subsequent experimental validation due to its well-established role in regulating epithelial barrier integrity and its involvement in tight junction protein regulation—critical factors in UC pathogenesis (23). While MAPK11 and PTEN have also been implicated in inflammatory processes and cell survival in UC, KLF4 stands out due to its direct influence on the gut epithelial barrier, which is essential for maintaining intestinal homeostasis. Although the role of KLF4 in UC remains debated, with some studies suggesting context-dependent effects (24), its strong link to immune regulation and its potential to modulate intestinal permeability make it the most compelling candidate among the three. This makes KLF4 a logical target to explore the microbiota-mediated effects on UC, particularly its role in regulating epithelial barrier function and pyroptosis-related signaling (25).

A conceptual challenge arises from the fact that KLF4 is canonically recognized as an intracellular transcription factor, whereas our Mendelian randomization utilized genetically predicted plasma pQTLs. However, during active intestinal inflammation and epithelial turnover—processes central to UC pathogenesis—intracellular proteins are frequently released into the extracellular space and systemic circulation following cell death events, such as necrosis and pyroptosis. Consequently, circulating KLF4 levels can serve as a measurable proxy reflecting the extent of mucosal expression, barrier disruption, and intracellular signaling dynamics.

While KLF4 has long been implicated in the regulation of epithelial barrier integrity, our study reveals a complex and yet underexplored relationship between KLF4 and pyroptosis in UC. Despite its critical role in maintaining intestinal barrier function, the exact mechanism by which KLF4 influences UC pathogenesis remains controversial. Some studies have suggested that KLF4’s effects may be context-dependent, with evidence showing both protective and detrimental roles depending on the tissue and inflammatory microenvironment (26). This complexity mirrors the ongoing debate in the literature surrounding other key inflammatory mediators, such as NLRP3 and GSDMD, whose roles in UC have also been marked by contradictions (27–30).

Interestingly, our study’s finding that KLF4 regulates intestinal permeability and modulates GSDMD cleavage provides a potential explanation for its seemingly dual role. GSDMD, a key mediator of pyroptosis, has been widely associated with intestinal inflammation and epithelial cell death in UC (28, 31). By inhibiting GSDMD cleavage, KLF4 may dampen inflammatory cell death, leading to a more controlled inflammatory response. This observation aligns with recent findings suggesting that targeting pyroptosis could offer a promising therapeutic strategy for managing UC (31). Additionally, KLF4’s capacity to modulate immune cell function further underscores its potential as a multifaceted regulator in UC. As shown in our study, KLF4 overexpression in a mouse model significantly altered the splenic immune profile, reducing the infiltration of pro-inflammatory immune cells, which is consistent with its proposed role in immune tolerance and mucosal immunity (32).

The role of KLF4 in modulating intestinal permeability and regulating tight junction proteins like Claudin-1 and Occludin adds another layer of complexity to its functional repertoire. Previous studies have shown that intestinal tight junctions play a central role in maintaining the integrity of the intestinal barrier and regulating inflammatory responses (33, 34). Our results, demonstrating that KLF4 overexpression restores the expression of these tight junction proteins, further support the hypothesis that KLF4 acts as a critical gatekeeper of intestinal permeability in UC. These findings also offer potential insights into the therapeutic targeting of KLF4 to restore epithelial integrity and prevent inflammation-associated barrier dysfunction.

Given KLF4’s central role in UC pathogenesis, our findings have significant clinical implications. Not only does KLF4 serve as a promising biomarker for disease severity and treatment response, but it also emerges as a novel therapeutic target. This could open avenues for developing KLF4-based therapies or biomarker-guided personalized treatments for UC, especially for patients who are refractory to conventional therapies. KLF4 expression was notably higher in UC patients responding to anti-TNF-α therapy, which suggests that KLF4 could help stratify patients for more effective treatment regimens. Future studies should focus on clinical trials to validate KLF4-based interventions, especially those aimed at modulating intestinal permeability and pyroptosis.

Several limitations warrant consideration. First, while MR minimizes confounding, horizontal pleiotropy remains a potential caveat, despite our use of robust sensitivity analyses. Furthermore, due to the lack of complete full-summary statistics across all utilized datasets, we were unable to perform formal colocalization analyses. Consequently, we cannot definitively differentiate true causality from the confounding effects of linkage disequilibrium for our pQTL-based findings. Second, the microbial and proteomic GWAS data are primarily from European ancestry, limiting the generalizability of our findings. Third, the AAV-KLF4 model induces systemic overexpression; thus, the cell type-specific contributions of KLF4 in intestinal epithelial versus immune cells remain to be dissected using conditional knockout models. Finally, the predictive biomarker analysis for anti-TNF-α response utilizing microarray cohorts involved relatively small sample sizes, which inherently carries a risk of overfitting. These specific predictive findings should be considered exploratory and require validation in larger, prospectively collected, and independent clinical cohorts.

Importantly, with respect to the upstream axis, the proposed “microbiota-to-KLF4” link was inferred computationally from human genetic data, whereas our experimental work was designed to validate the downstream host-effector mechanisms (KLF4-mediated barrier protection and attenuation of pyroptosis). We did not directly interrogate microbiome composition or causality *in vivo* (e.g., 16S rRNA profiling, metagenomics, or fecal microbiota transplantation). Therefore, the specific microbial drivers that modulate KLF4 activity remain to be experimentally established. Future studies integrating microbiome profiling and functional perturbation approaches will be required to validate and refine the upstream microbial component of this axis.

## Conclusions

This study highlights the critical role of KLF4 in gut barrier integrity, immune regulation, and pyroptosis in UC. By uncovering microbial-mediated effects on KLF4, we provide new insights into the complex interplay between host genetics, immune signaling, and gut microbiota in UC pathogenesis. As a potential biomarker and therapeutic target, KLF4 offers exciting prospects for improving UC management and personalized treatment strategies.

## Data Availability

The original contributions presented in the study are included in the article/**Supplementary Material**. Further inquiries can be directed to the corresponding author.

## Supplementary material

Supplementary Data Sheet 1 Harmonized summary data, forest plots, funnel plots, data sources, harmonization details, and sensitivity analyses for the Mendelian randomization analysis of pyroptosis-related proteins and ulcerative colitis.

Supplementary Data Sheet 2 Full GCST identifiers, taxonomic labels, and Mendelian randomization statistics for the gut microbial traits associated with ulcerative colitis.

Supplementary Data Sheet 3 Full results of the pairwise Mendelian randomization analyses between ulcerative colitis-associated microbial taxa and ulcerative colitis-associated pyroptosis proteins, used for the downstream mediation analysis.
