# Supplementary figures and images for: Integrative Mendelian randomization and experimental validation prioritize KLF4 in the gut microbiota–pyroptosis–barrier axis of ulcerative colitis (part 1 of 3)
Source: Front Immunol. 2026 Mar 16;17:1773990. doi: 10.3389/fimmu.2026.1773990 (PMC13033518; doi:10.3389/fimmu.2026.1773990)

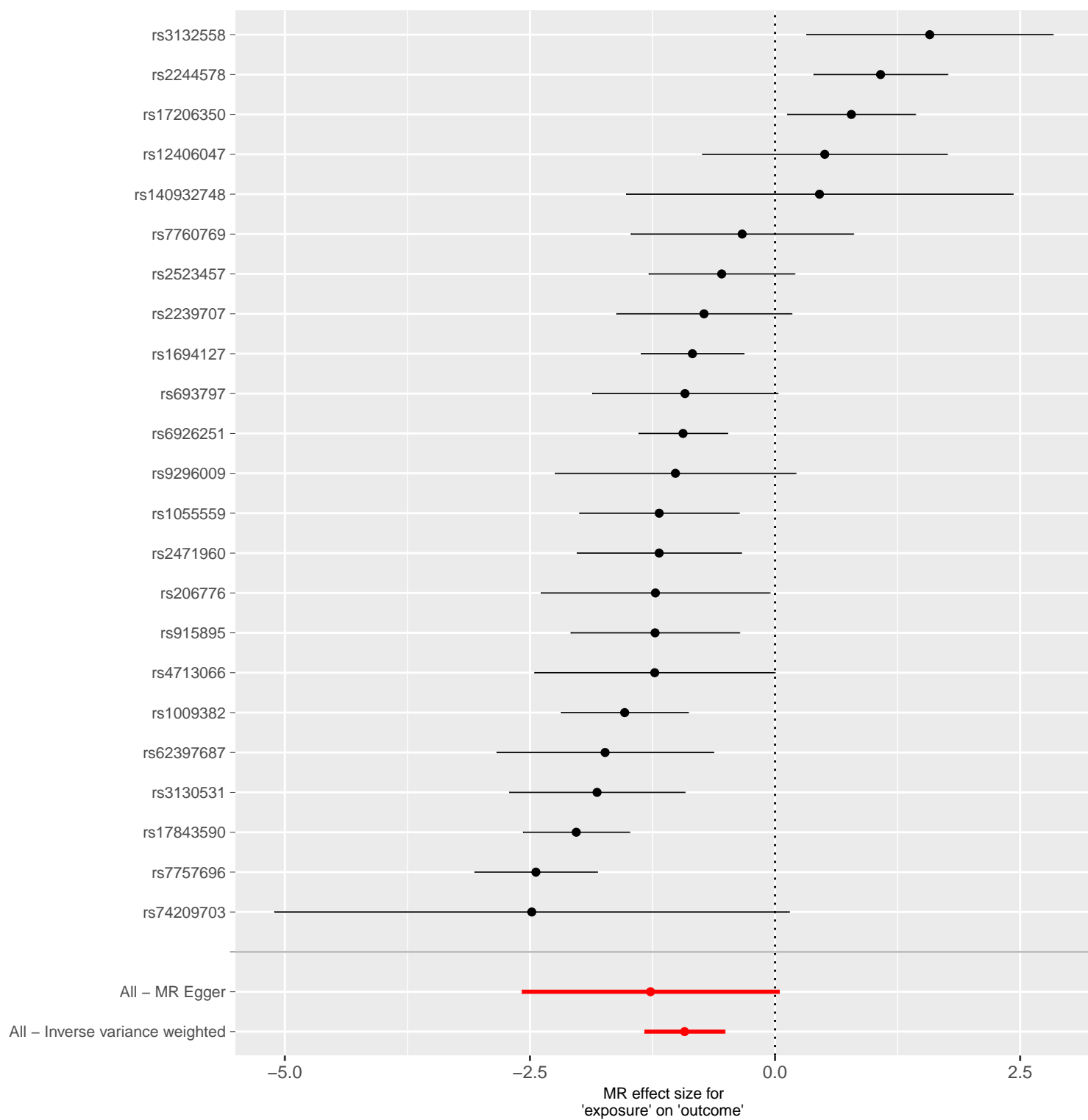

Supplement: Supplementary Data Sheet 1 — Harmonized summary data, forest plots, funnel plots, data sources, harmonization details, and sensitivity analyses for the Mendelian randomization analysis of pyroptosis-related proteins and ulcerative colitis. [file DataSheet1.zip › bdpqtlresult/11067_13_BGLAP_Osteocalcin/forest.pdf]

# MR Method

- Inverse variance weighted
- MR Egger

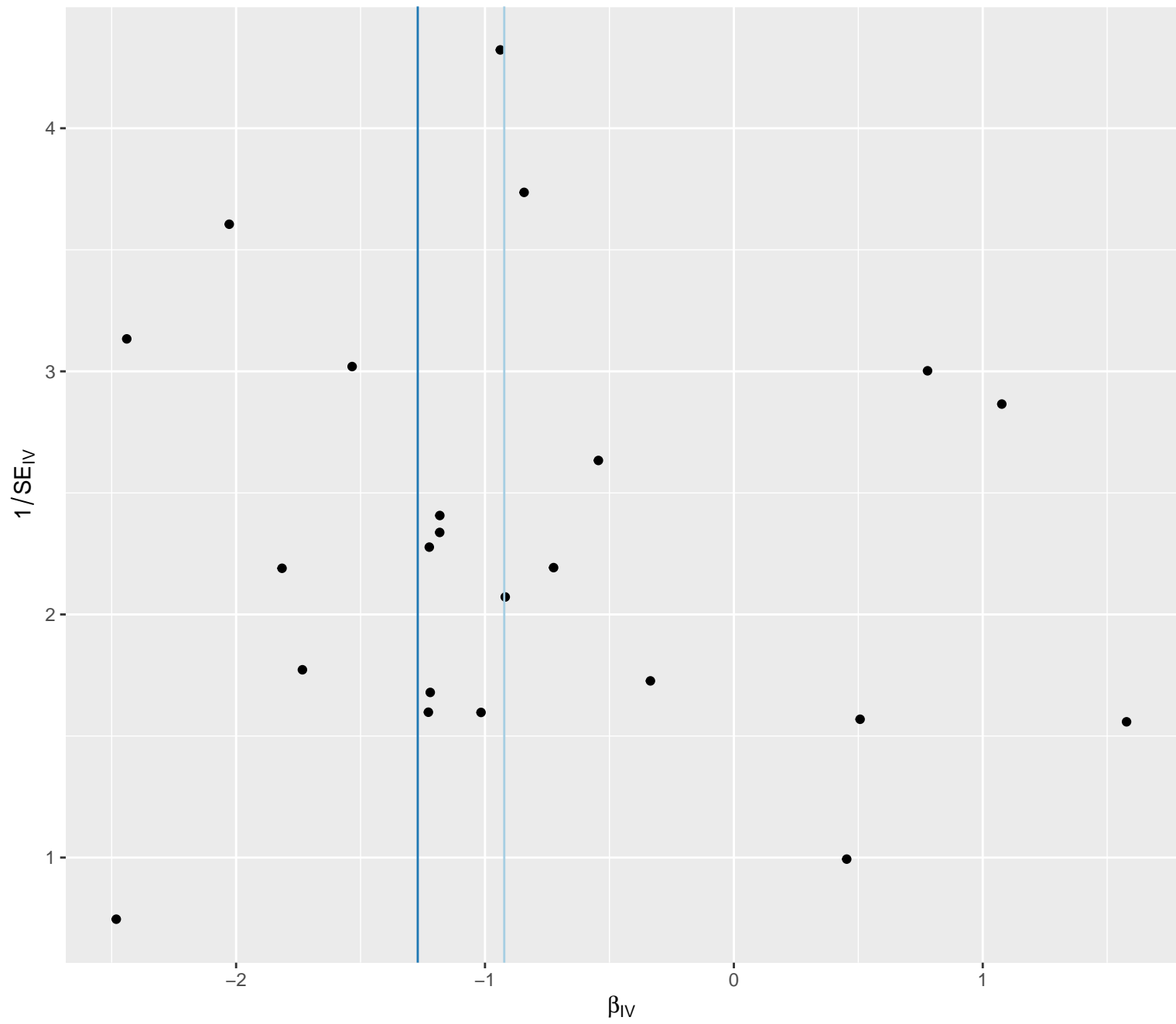

Supplement: Supplementary Data Sheet 1 — Harmonized summary data, forest plots, funnel plots, data sources, harmonization details, and sensitivity analyses for the Mendelian randomization analysis of pyroptosis-related proteins and ulcerative colitis. [file DataSheet1.zip › bdpqtlresult/11067_13_BGLAP_Osteocalcin/funnelplot.pdf]

# MR Test

- Inverse variance weighted
- MR Egger
- Simple mode
- Weighted median
- Weighted mode

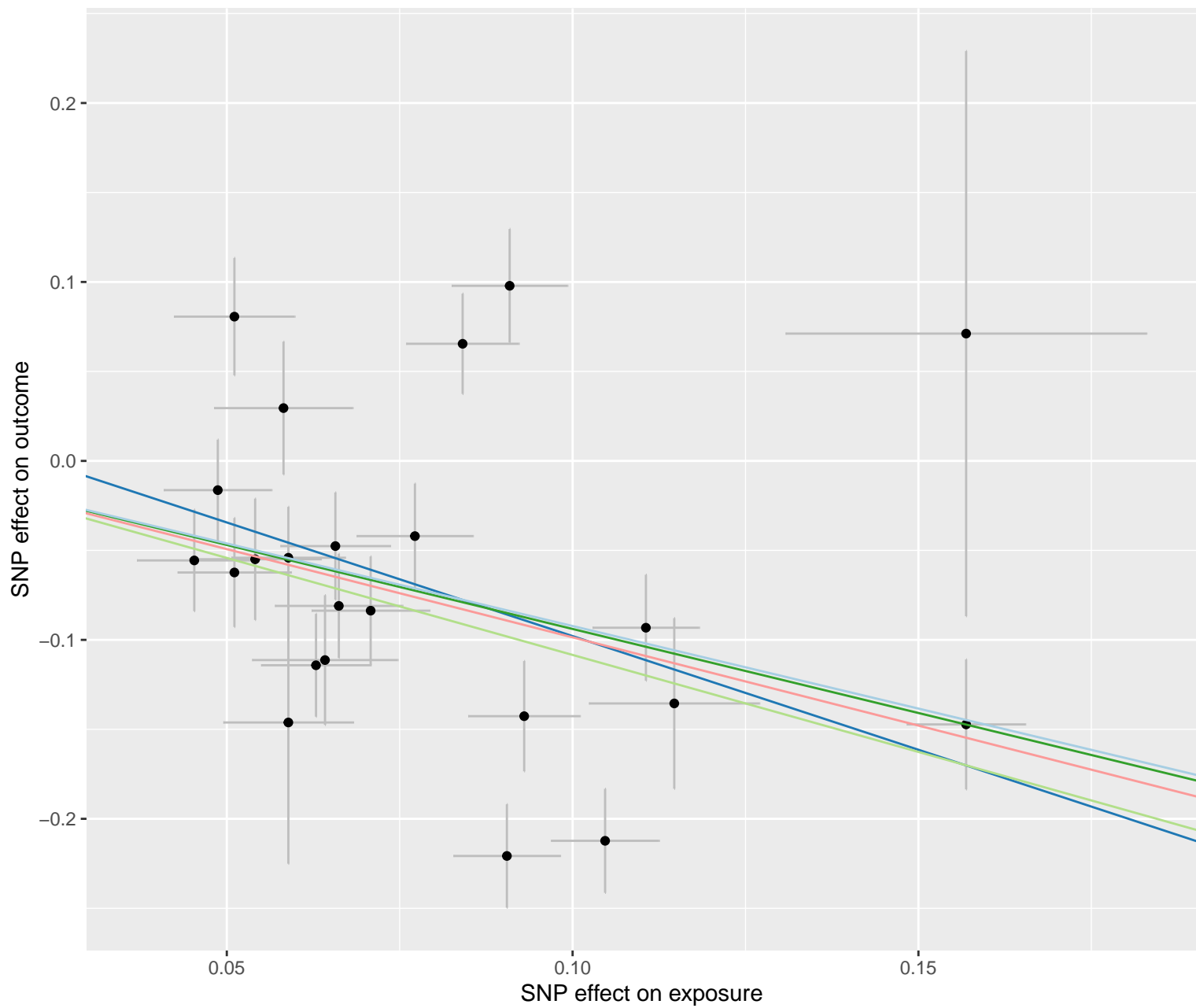

Supplement: Supplementary Data Sheet 1 — Harmonized summary data, forest plots, funnel plots, data sources, harmonization details, and sensitivity analyses for the Mendelian randomization analysis of pyroptosis-related proteins and ulcerative colitis. [file DataSheet1.zip › bdpqtlresult/11067_13_BGLAP_Osteocalcin/scatter.pdf]

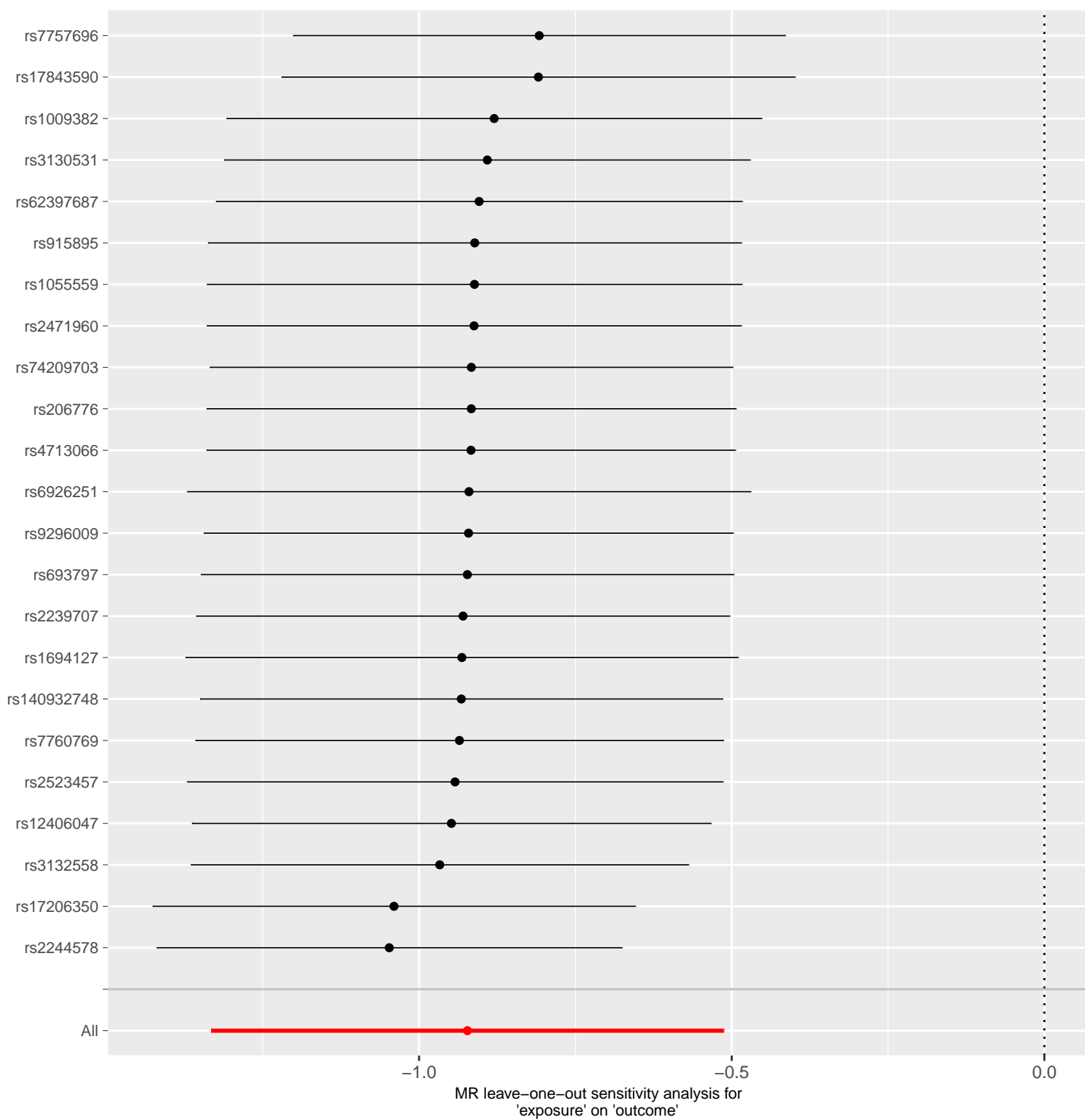

Supplement: Supplementary Data Sheet 1 — Harmonized summary data, forest plots, funnel plots, data sources, harmonization details, and sensitivity analyses for the Mendelian randomization analysis of pyroptosis-related proteins and ulcerative colitis. [file DataSheet1.zip › bdpqtlresult/11067_13_BGLAP_Osteocalcin/sensitivity-analysis.pdf]

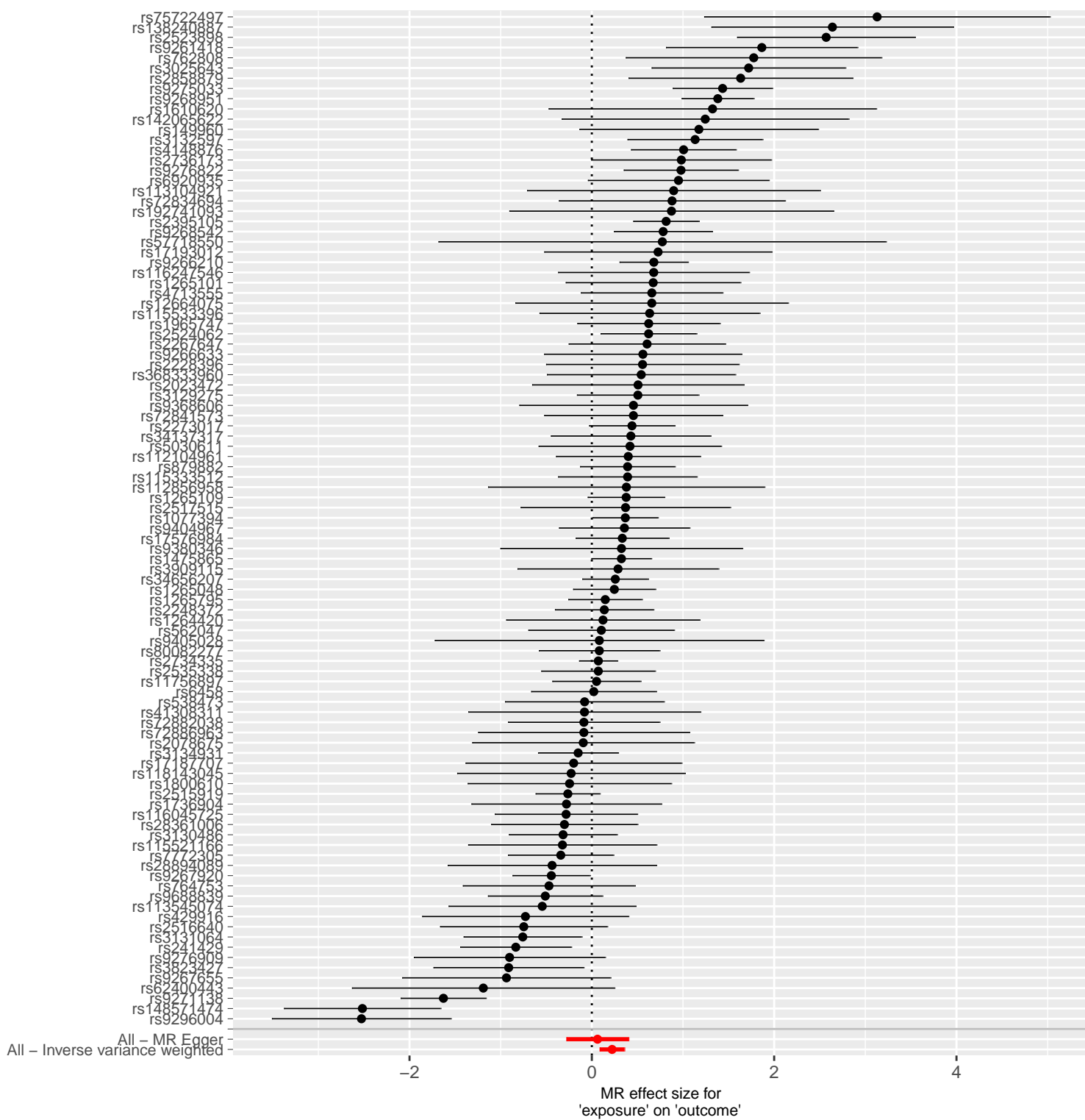

Supplement: Supplementary Data Sheet 1 — Harmonized summary data, forest plots, funnel plots, data sources, harmonization details, and sensitivity analyses for the Mendelian randomization analysis of pyroptosis-related proteins and ulcerative colitis. [file DataSheet1.zip › bdpqtlresult/12332_7_EEF2K_EF2K/forest.pdf]

# MR Method

- Inverse variance weighted
- MR Egger

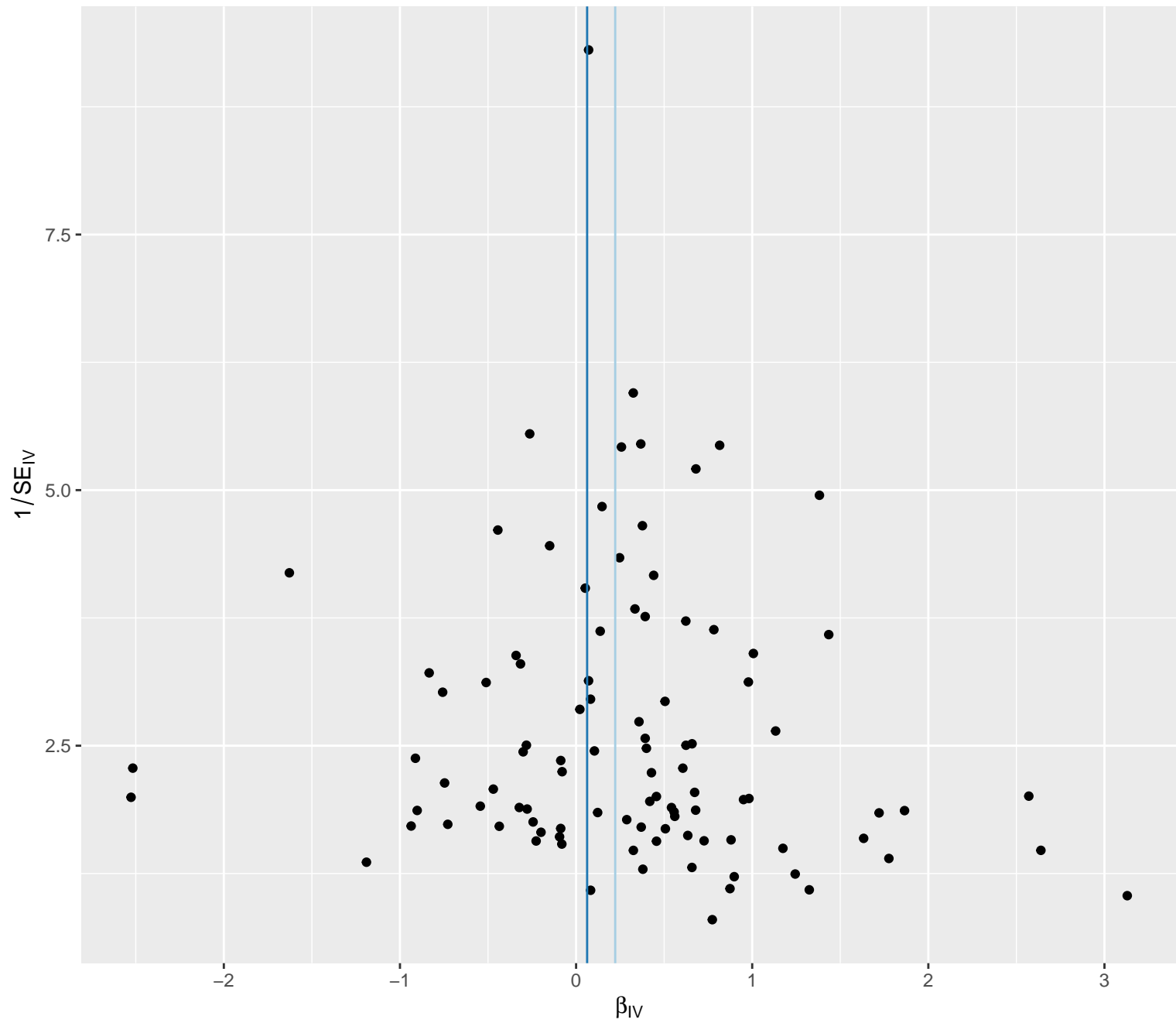

Supplement: Supplementary Data Sheet 1 — Harmonized summary data, forest plots, funnel plots, data sources, harmonization details, and sensitivity analyses for the Mendelian randomization analysis of pyroptosis-related proteins and ulcerative colitis. [file DataSheet1.zip › bdpqtlresult/12332_7_EEF2K_EF2K/funnelplot.pdf]

# MR Test

- Inverse variance weighted
- MR Egger
- Simple mode
- Weighted median
- Weighted mode

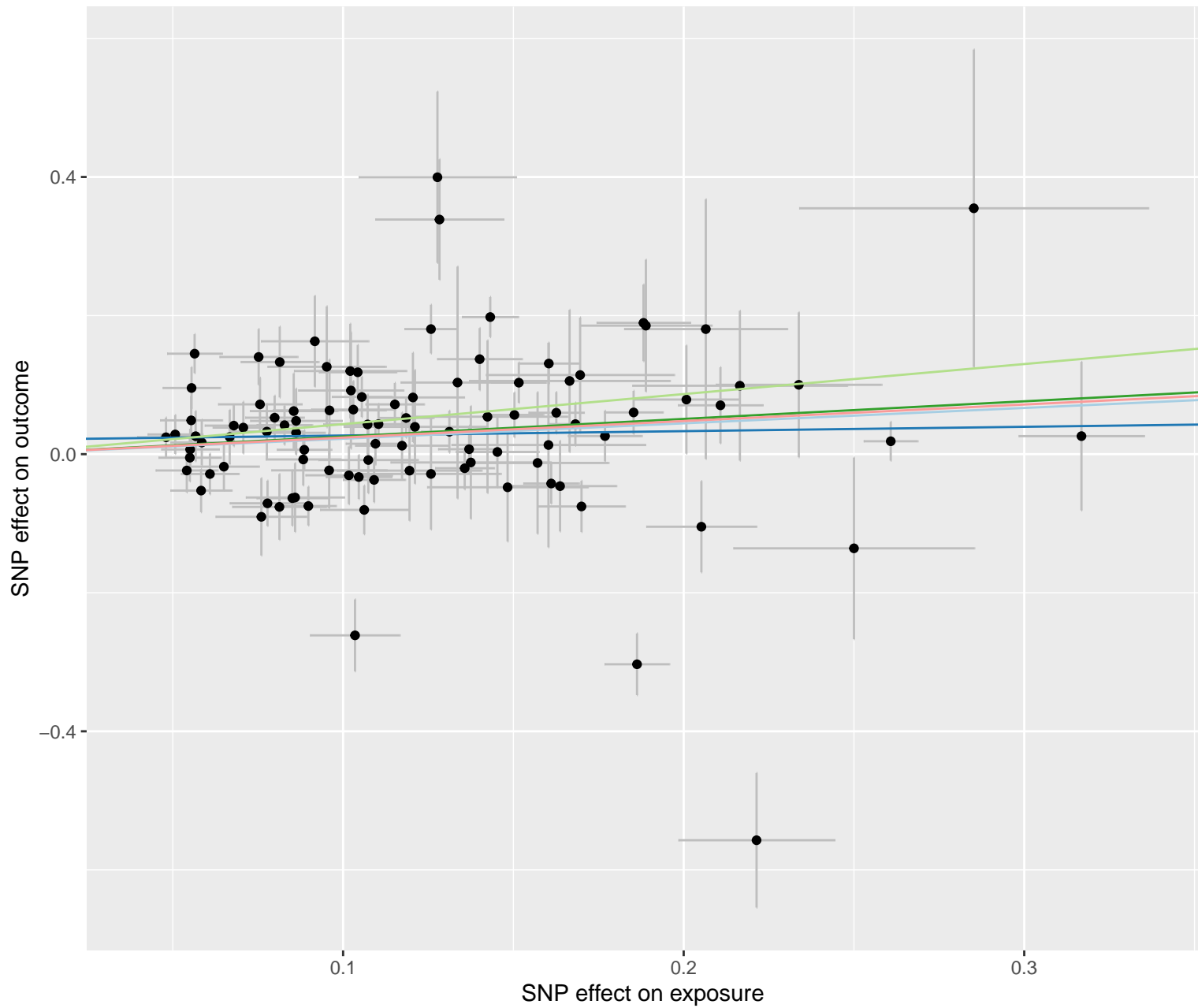

Supplement: Supplementary Data Sheet 1 — Harmonized summary data, forest plots, funnel plots, data sources, harmonization details, and sensitivity analyses for the Mendelian randomization analysis of pyroptosis-related proteins and ulcerative colitis. [file DataSheet1.zip › bdpqtlresult/12332_7_EEF2K_EF2K/scatter.pdf]

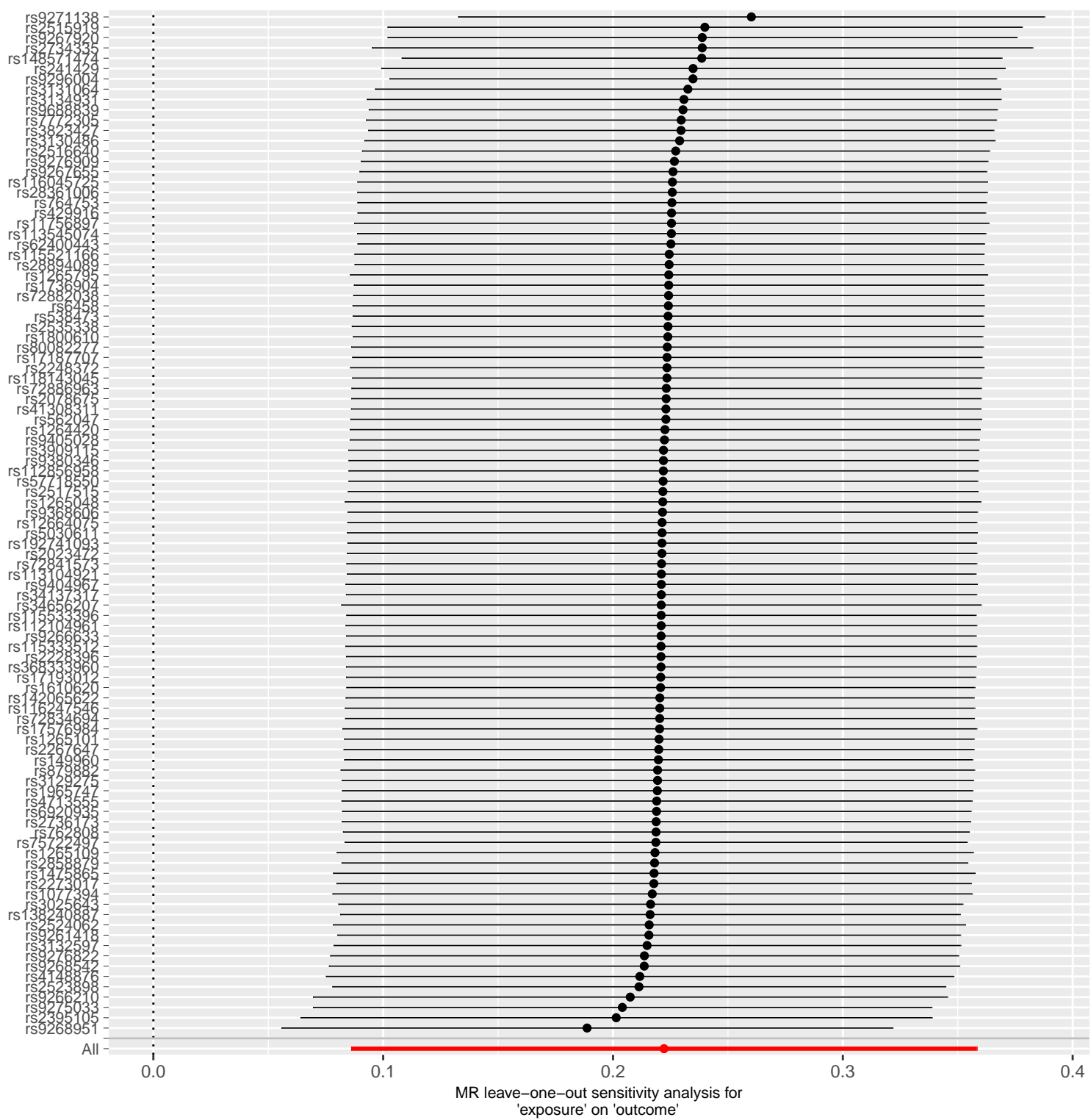

Supplement: Supplementary Data Sheet 1 — Harmonized summary data, forest plots, funnel plots, data sources, harmonization details, and sensitivity analyses for the Mendelian randomization analysis of pyroptosis-related proteins and ulcerative colitis. [file DataSheet1.zip › bdpqtlresult/12332_7_EEF2K_EF2K/sensitivity-analysis.pdf]

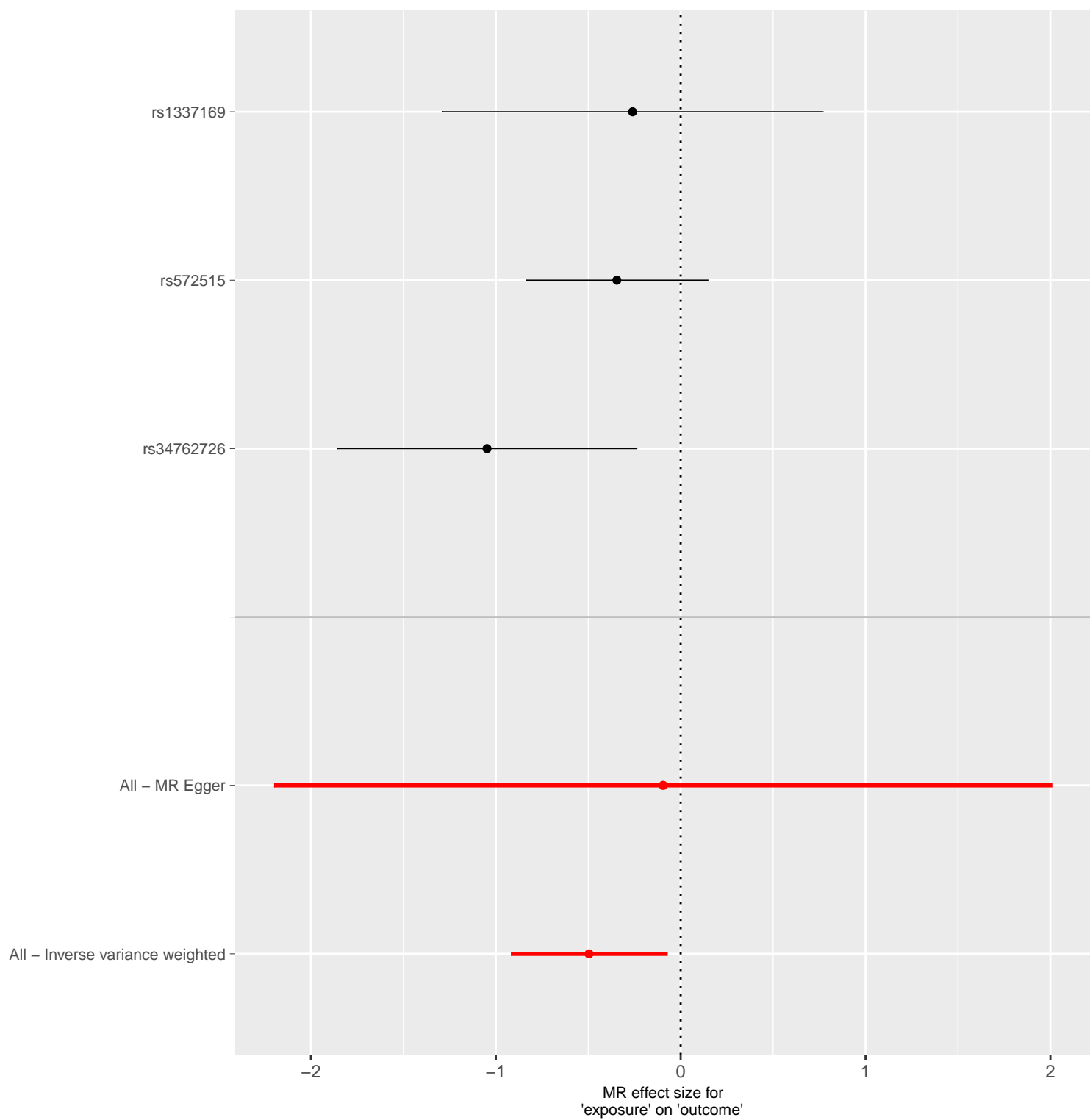

Supplement: Supplementary Data Sheet 1 — Harmonized summary data, forest plots, funnel plots, data sources, harmonization details, and sensitivity analyses for the Mendelian randomization analysis of pyroptosis-related proteins and ulcerative colitis. [file DataSheet1.zip › bdpqtlresult/12439_67_IRF9_ISGF3/forest.pdf]

# MR Method

- Inverse variance weighted
- MR Egger

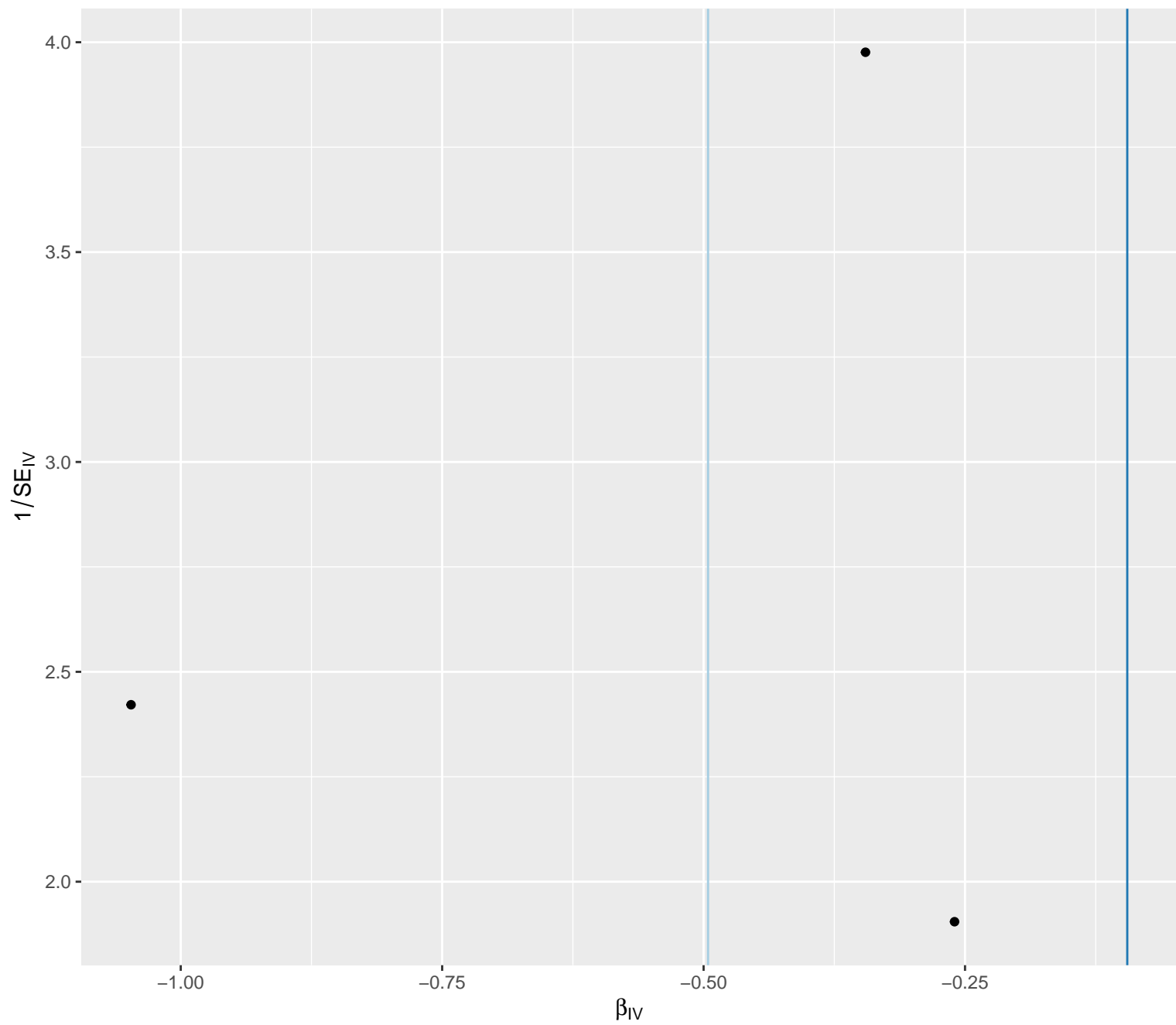

Supplement: Supplementary Data Sheet 1 — Harmonized summary data, forest plots, funnel plots, data sources, harmonization details, and sensitivity analyses for the Mendelian randomization analysis of pyroptosis-related proteins and ulcerative colitis. [file DataSheet1.zip › bdpqtlresult/12439_67_IRF9_ISGF3/funnelplot.pdf]

# MR Test

- Inverse variance weighted
- MR Egger
- Simple mode
- Weighted median
- Weighted mode

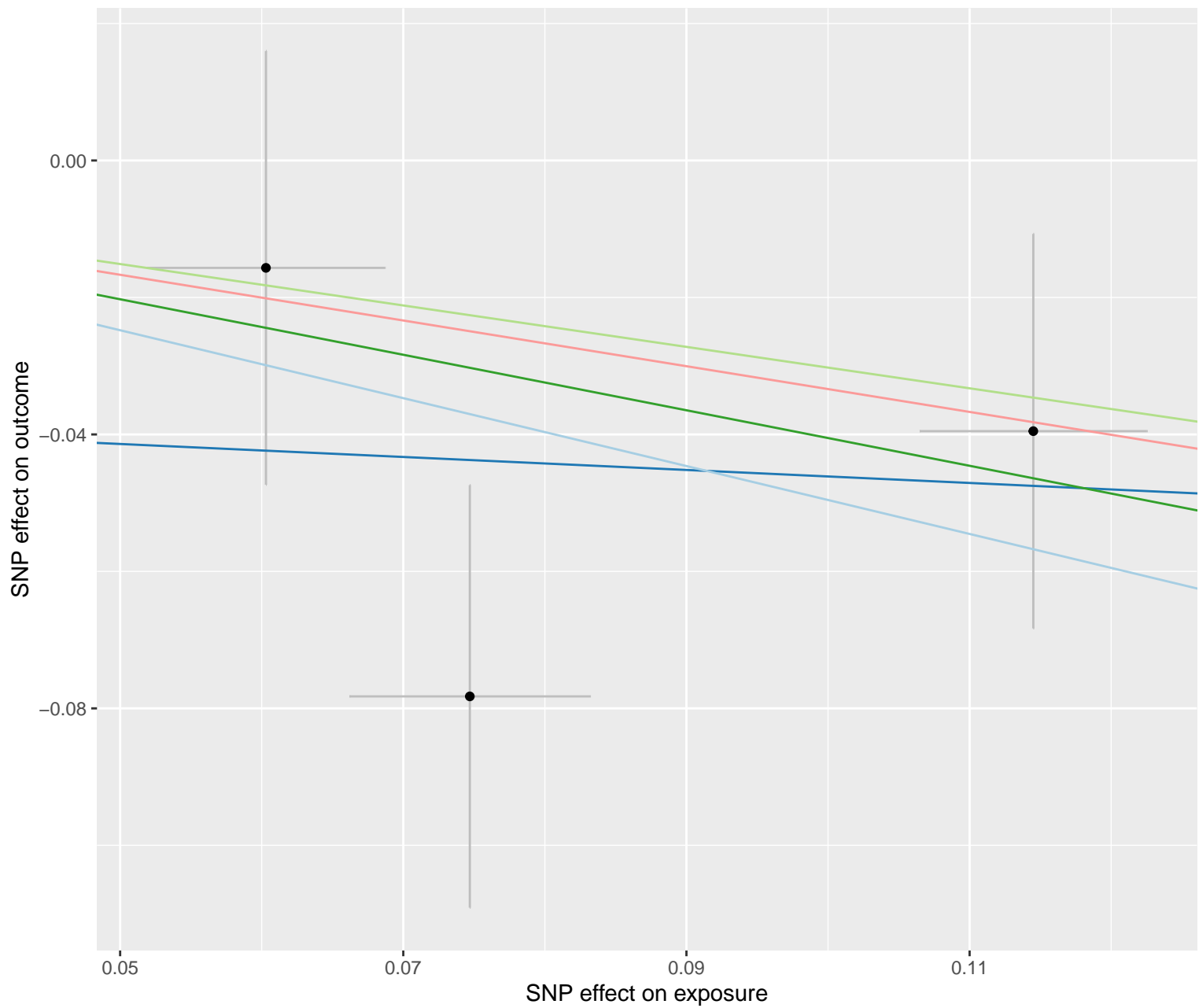

Supplement: Supplementary Data Sheet 1 — Harmonized summary data, forest plots, funnel plots, data sources, harmonization details, and sensitivity analyses for the Mendelian randomization analysis of pyroptosis-related proteins and ulcerative colitis. [file DataSheet1.zip › bdpqtlresult/12439_67_IRF9_ISGF3/scatter.pdf]

rs34762726

rs1337169

rs572515

All

-1.5

-1.0

-0.5

0.0

MR leave-one-out sensitivity analysis for  
'exposure' on 'outcome'

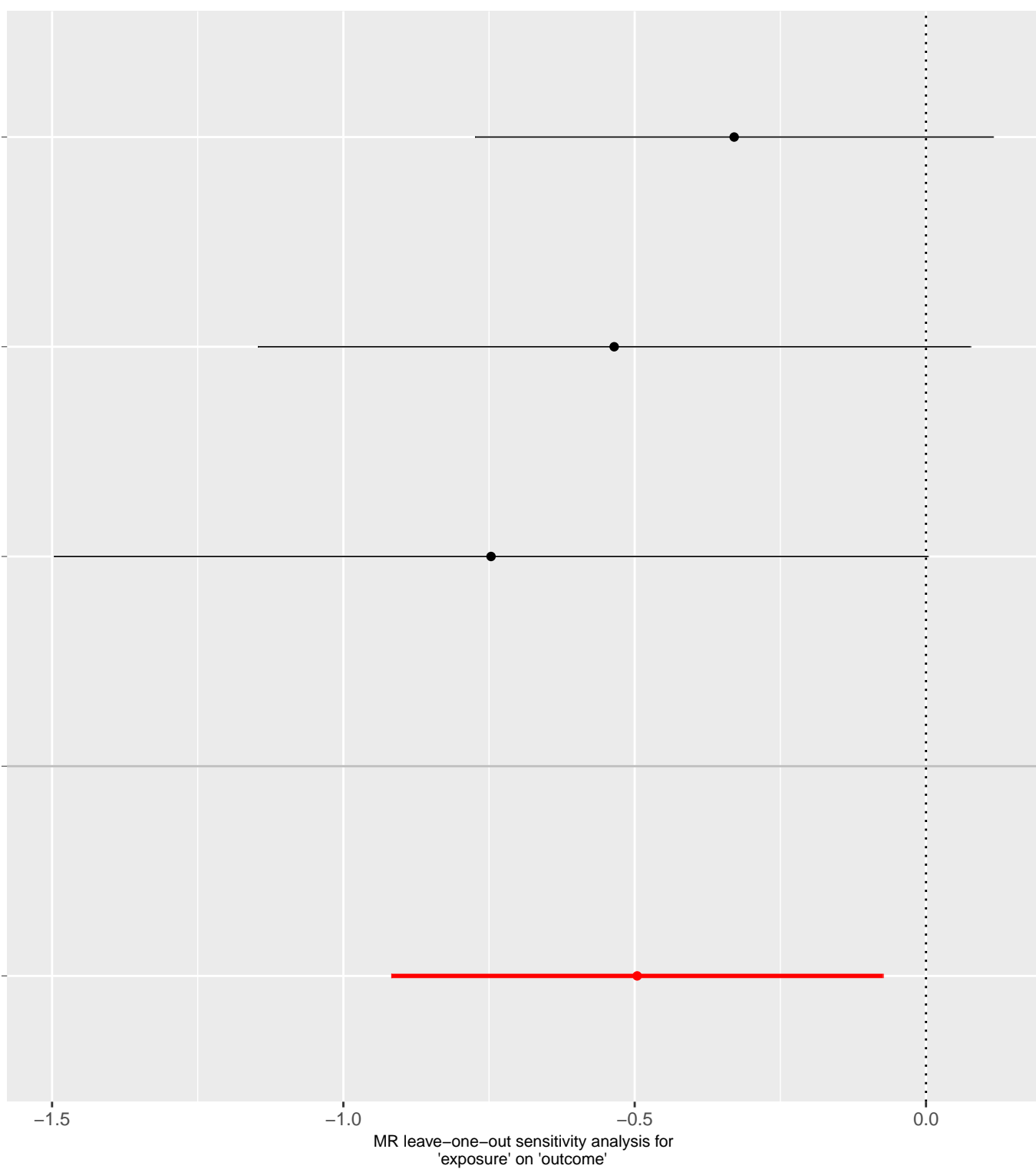

Supplement: Supplementary Data Sheet 1 — Harmonized summary data, forest plots, funnel plots, data sources, harmonization details, and sensitivity analyses for the Mendelian randomization analysis of pyroptosis-related proteins and ulcerative colitis. [file DataSheet1.zip › bdpqtlresult/12439_67_IRF9_ISGF3/sensitivity-analysis.pdf]

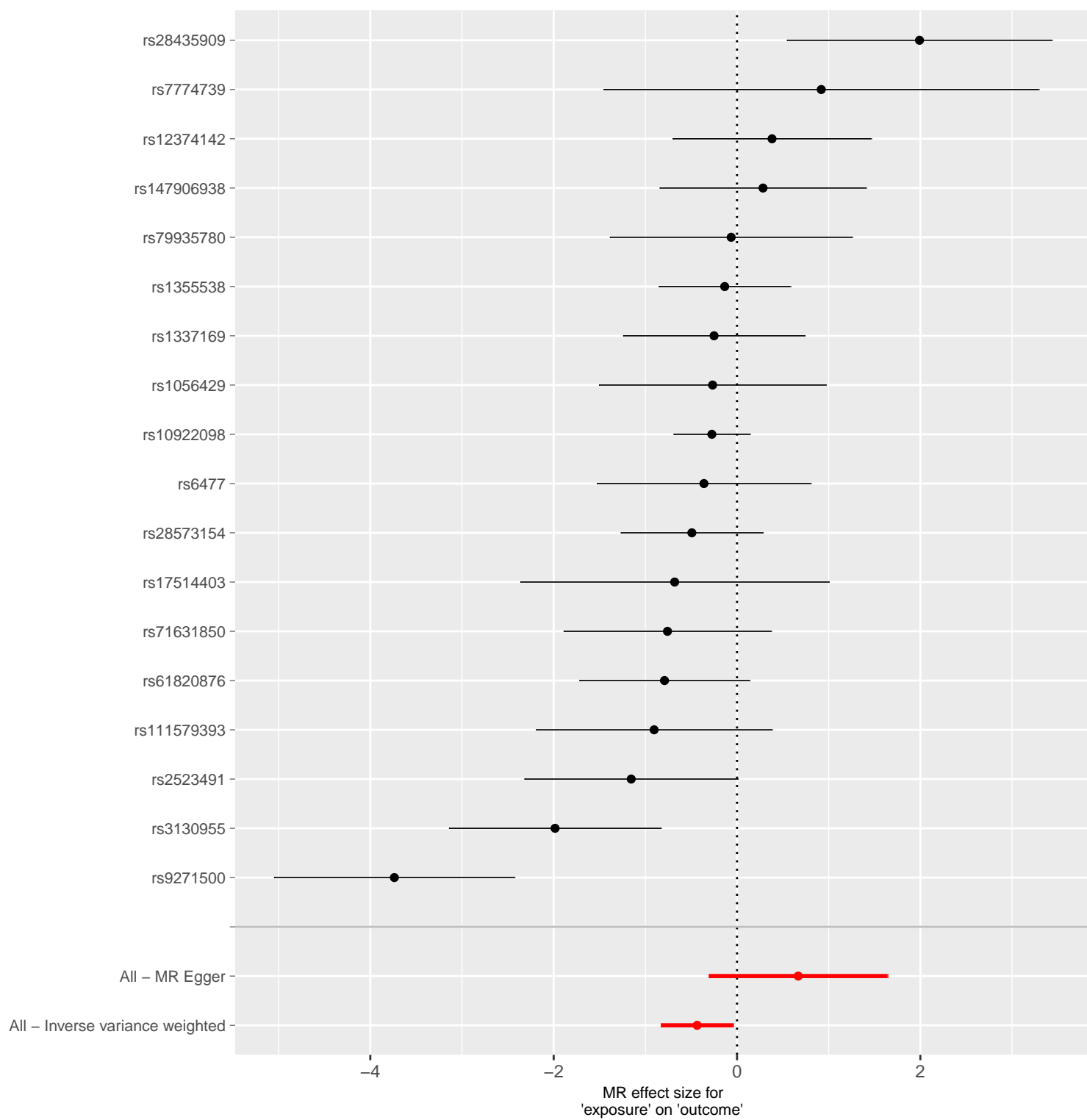

Supplement: Supplementary Data Sheet 1 — Harmonized summary data, forest plots, funnel plots, data sources, harmonization details, and sensitivity analyses for the Mendelian randomization analysis of pyroptosis-related proteins and ulcerative colitis. [file DataSheet1.zip › bdpqtlresult/13032_1_BECN1_BECN1/forest.pdf]

# MR Method

- Inverse variance weighted
- MR Egger

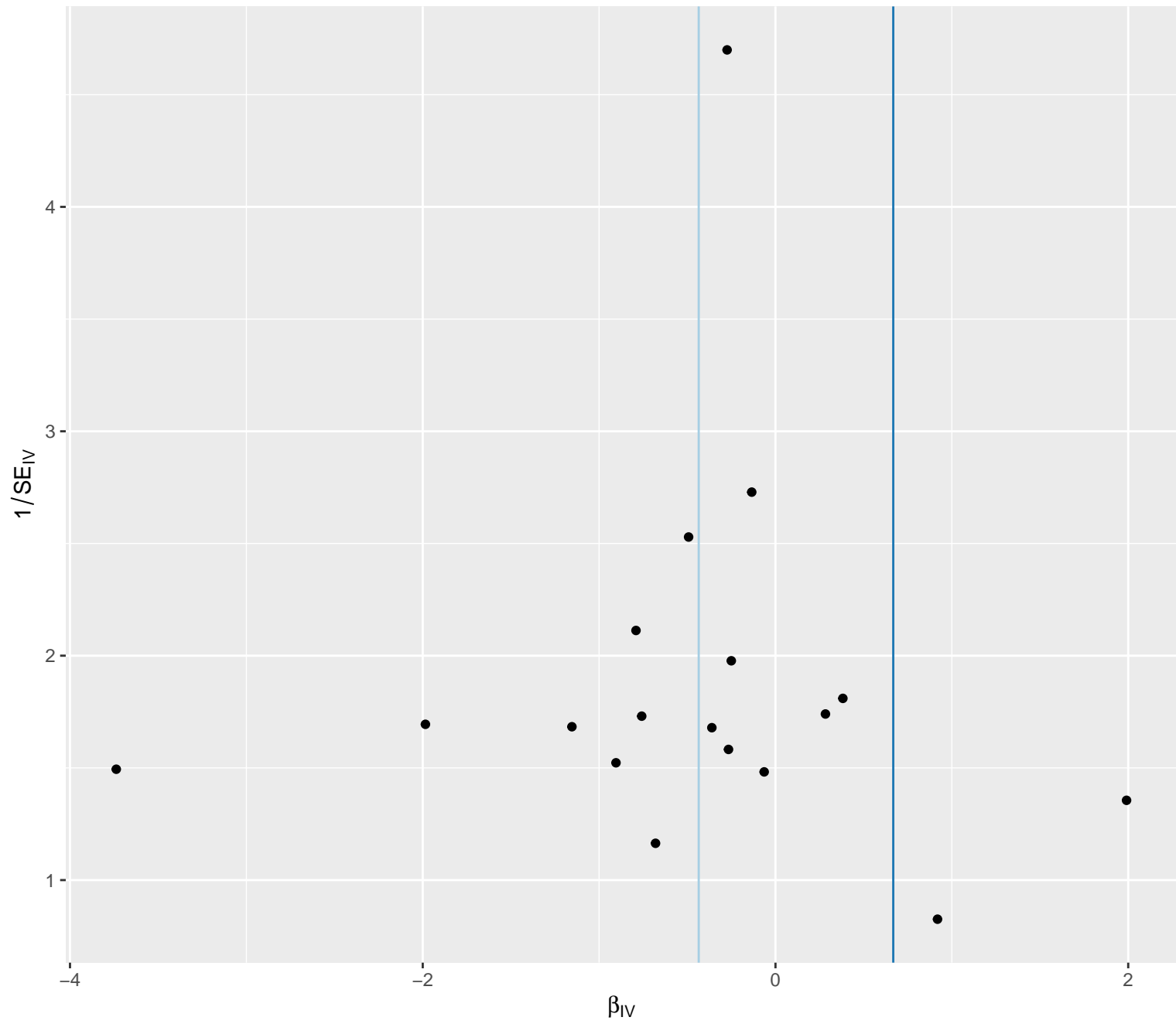

Supplement: Supplementary Data Sheet 1 — Harmonized summary data, forest plots, funnel plots, data sources, harmonization details, and sensitivity analyses for the Mendelian randomization analysis of pyroptosis-related proteins and ulcerative colitis. [file DataSheet1.zip › bdpqtlresult/13032_1_BECN1_BECN1/funnelplot.pdf]

# MR Test

- Inverse variance weighted
- MR Egger
- Simple mode
- Weighted median
- Weighted mode

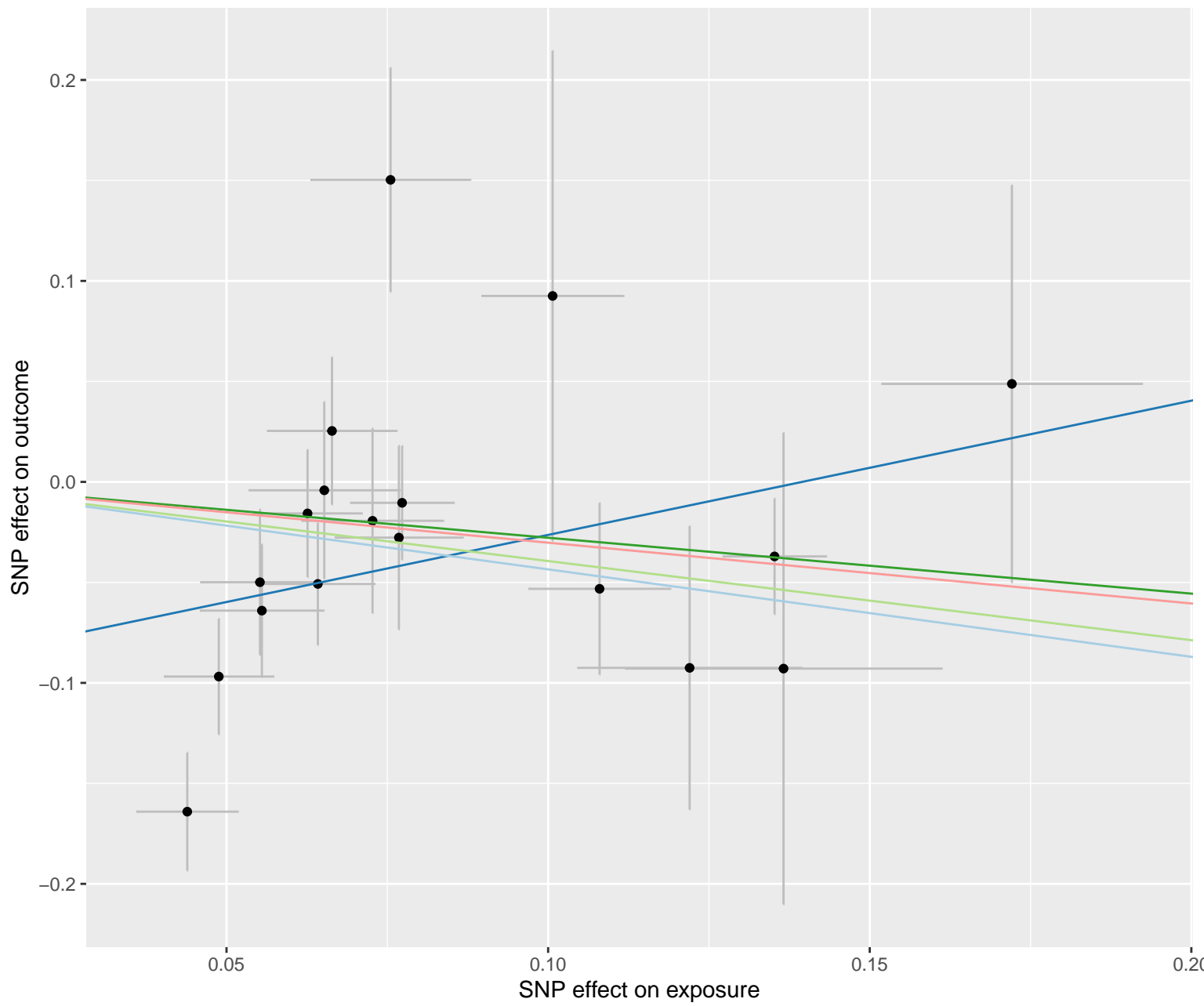

Supplement: Supplementary Data Sheet 1 — Harmonized summary data, forest plots, funnel plots, data sources, harmonization details, and sensitivity analyses for the Mendelian randomization analysis of pyroptosis-related proteins and ulcerative colitis. [file DataSheet1.zip › bdpqtlresult/13032_1_BECN1_BECN1/scatter.pdf]

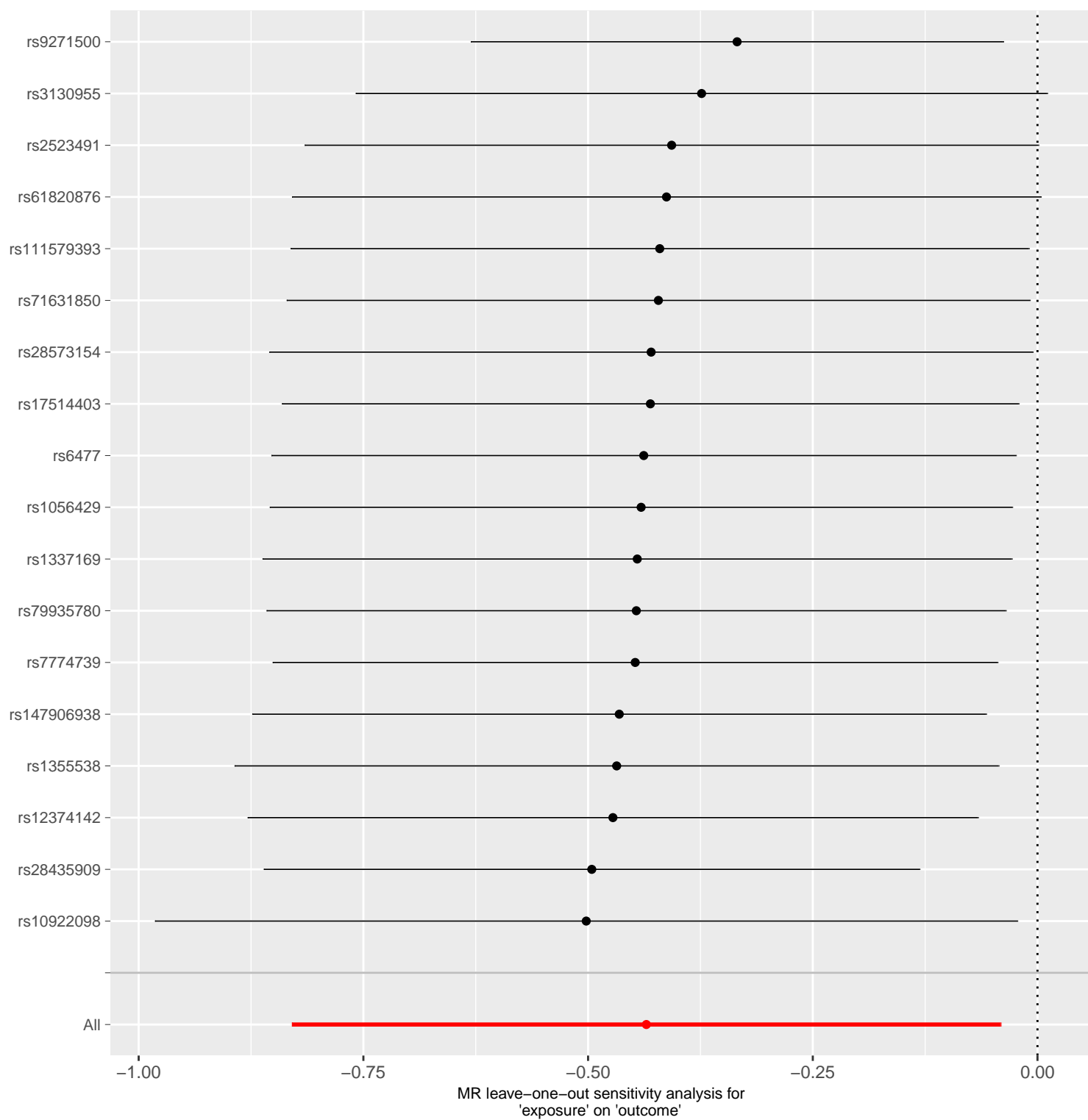

Supplement: Supplementary Data Sheet 1 — Harmonized summary data, forest plots, funnel plots, data sources, harmonization details, and sensitivity analyses for the Mendelian randomization analysis of pyroptosis-related proteins and ulcerative colitis. [file DataSheet1.zip › bdpqtlresult/13032_1_BECN1_BECN1/sensitivity-analysis.pdf]

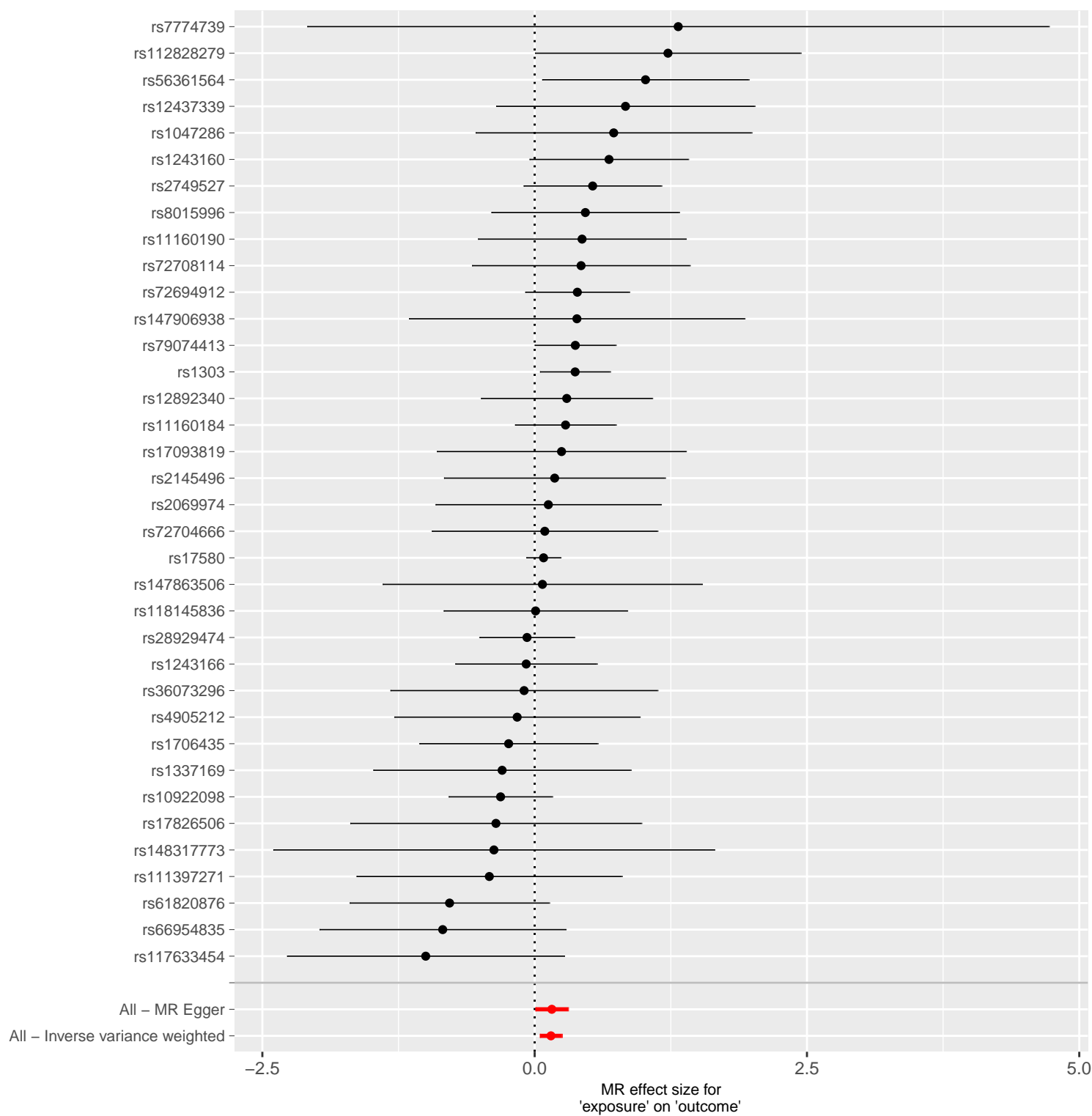

Supplement: Supplementary Data Sheet 1 — Harmonized summary data, forest plots, funnel plots, data sources, harmonization details, and sensitivity analyses for the Mendelian randomization analysis of pyroptosis-related proteins and ulcerative colitis. [file DataSheet1.zip › bdpqtlresult/13105_7_SNAP25_SNP25/forest.pdf]

# MR Method

- Inverse variance weighted
- MR Egger

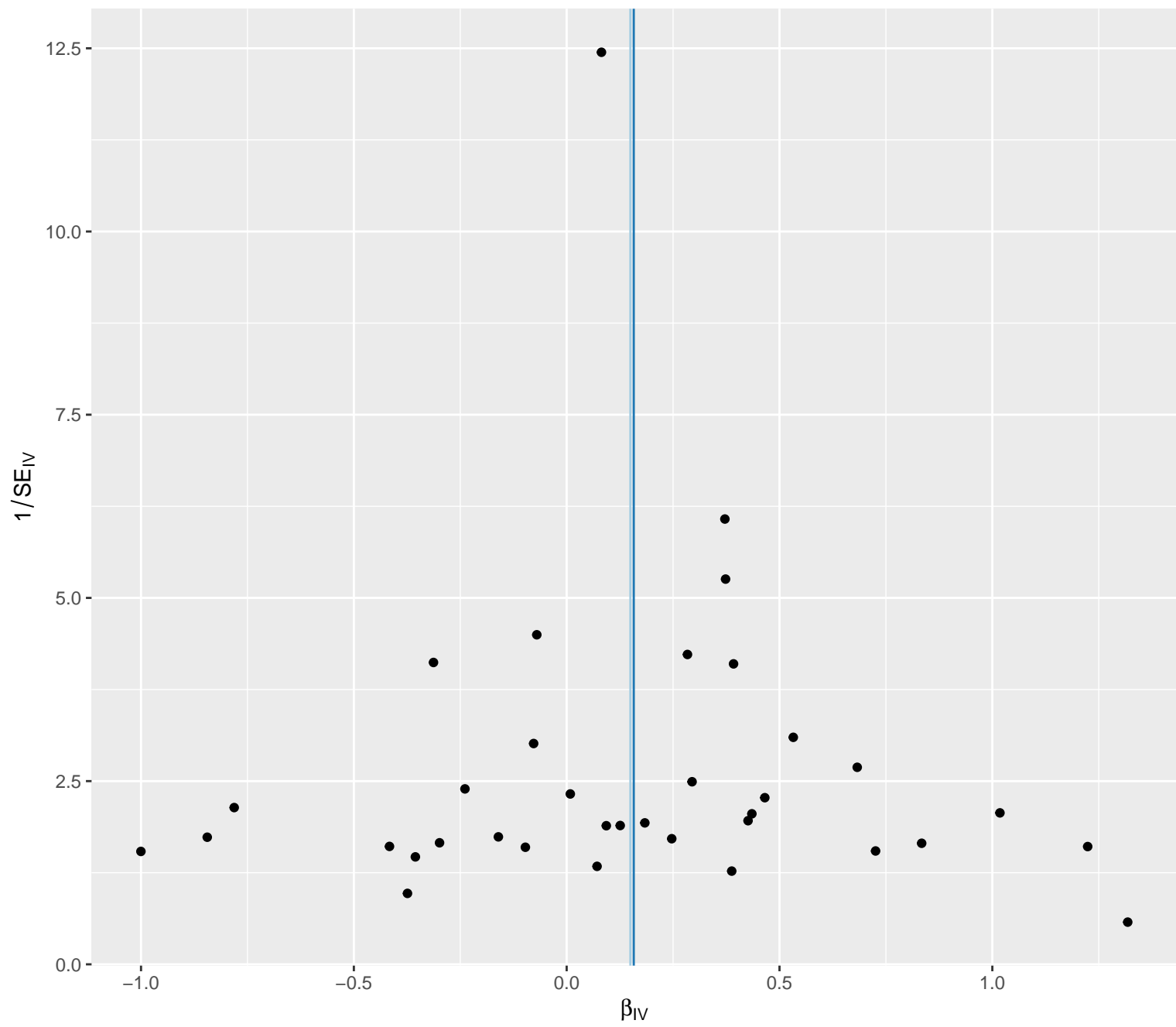

Supplement: Supplementary Data Sheet 1 — Harmonized summary data, forest plots, funnel plots, data sources, harmonization details, and sensitivity analyses for the Mendelian randomization analysis of pyroptosis-related proteins and ulcerative colitis. [file DataSheet1.zip › bdpqtlresult/13105_7_SNAP25_SNP25/funnelplot.pdf]

# MR Test

- Inverse variance weighted
- MR Egger
- Simple mode
- Weighted median
- Weighted mode

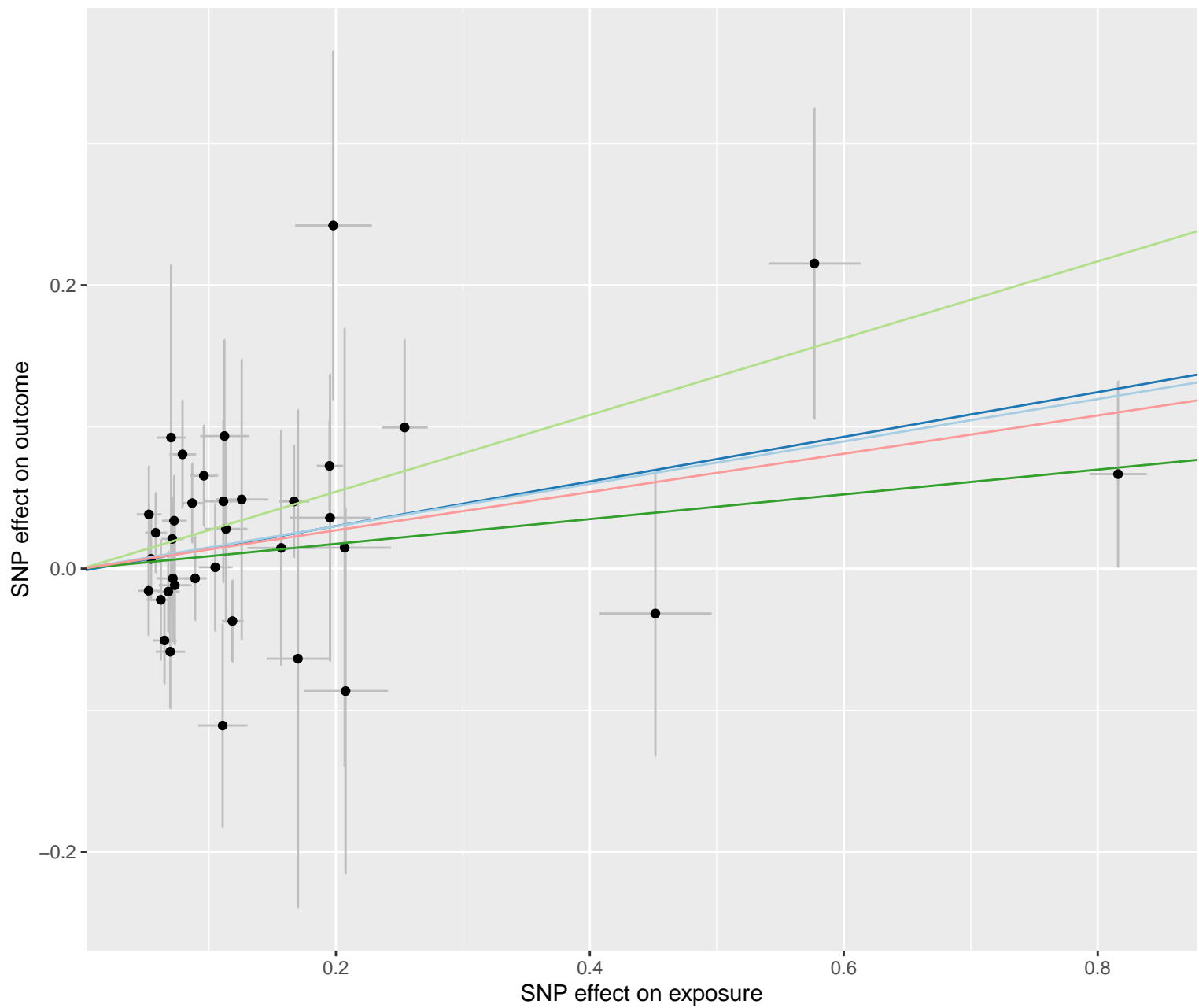

Supplement: Supplementary Data Sheet 1 — Harmonized summary data, forest plots, funnel plots, data sources, harmonization details, and sensitivity analyses for the Mendelian randomization analysis of pyroptosis-related proteins and ulcerative colitis. [file DataSheet1.zip › bdpqtlresult/13105_7_SNAP25_SNP25/scatter.pdf]

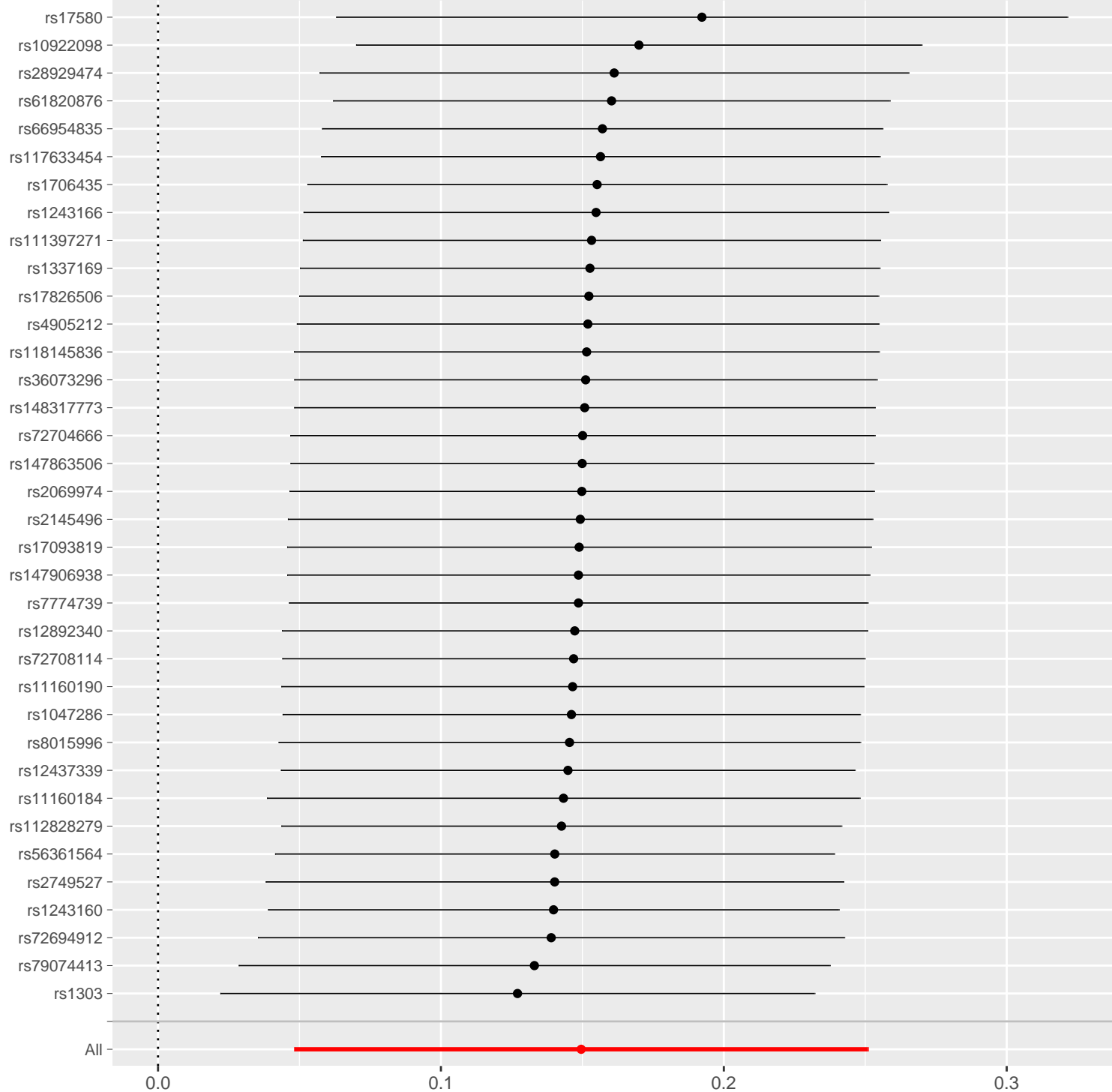

Supplement: Supplementary Data Sheet 1 — Harmonized summary data, forest plots, funnel plots, data sources, harmonization details, and sensitivity analyses for the Mendelian randomization analysis of pyroptosis-related proteins and ulcerative colitis. [file DataSheet1.zip › bdpqtlresult/13105_7_SNAP25_SNP25/sensitivity-analysis.pdf]

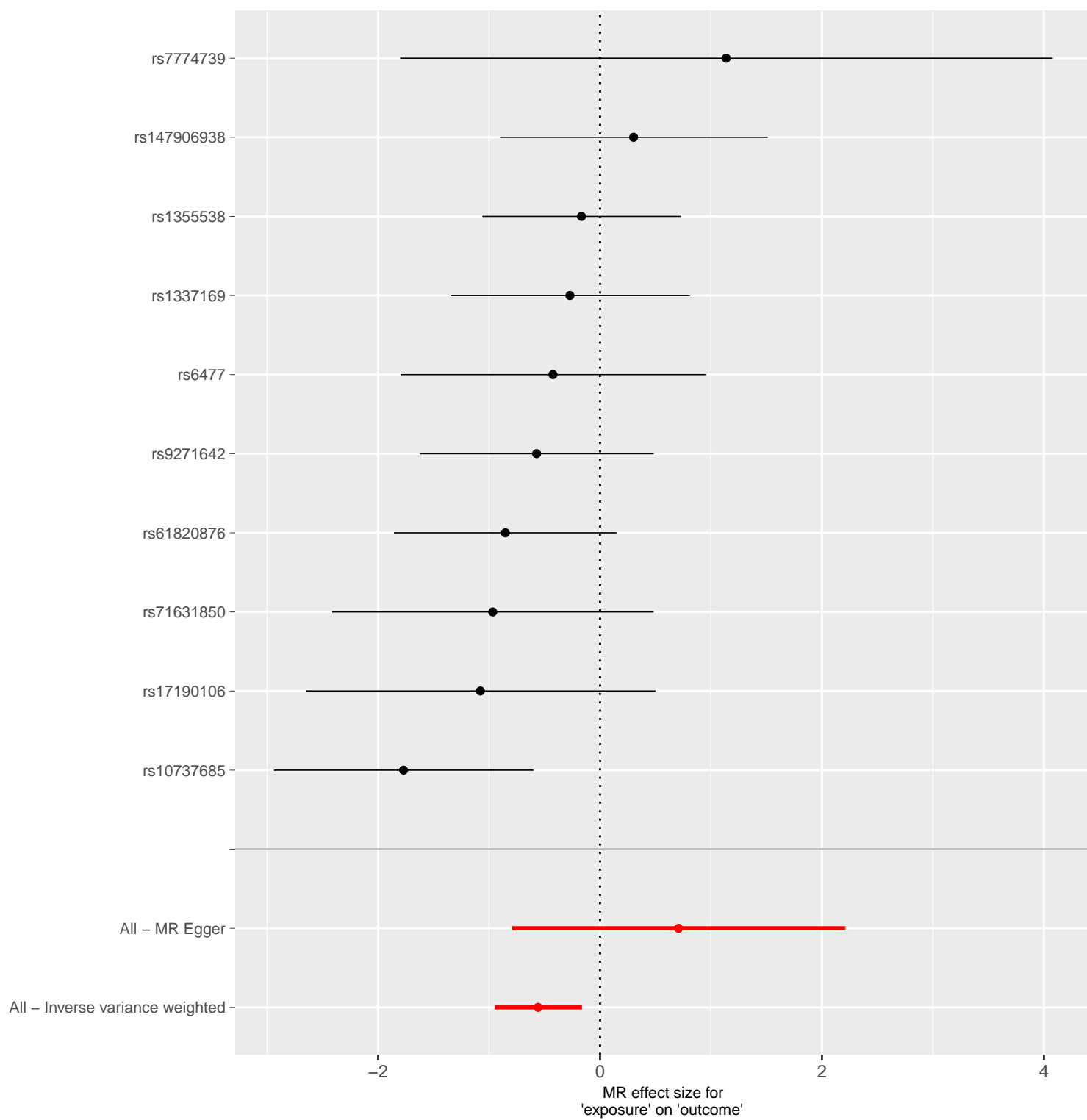

Supplement: Supplementary Data Sheet 1 — Harmonized summary data, forest plots, funnel plots, data sources, harmonization details, and sensitivity analyses for the Mendelian randomization analysis of pyroptosis-related proteins and ulcerative colitis. [file DataSheet1.zip › bdpqtlresult/13985_12_SMURF2_SMUF2/forest.pdf]

# MR Method

- Inverse variance weighted
- MR Egger

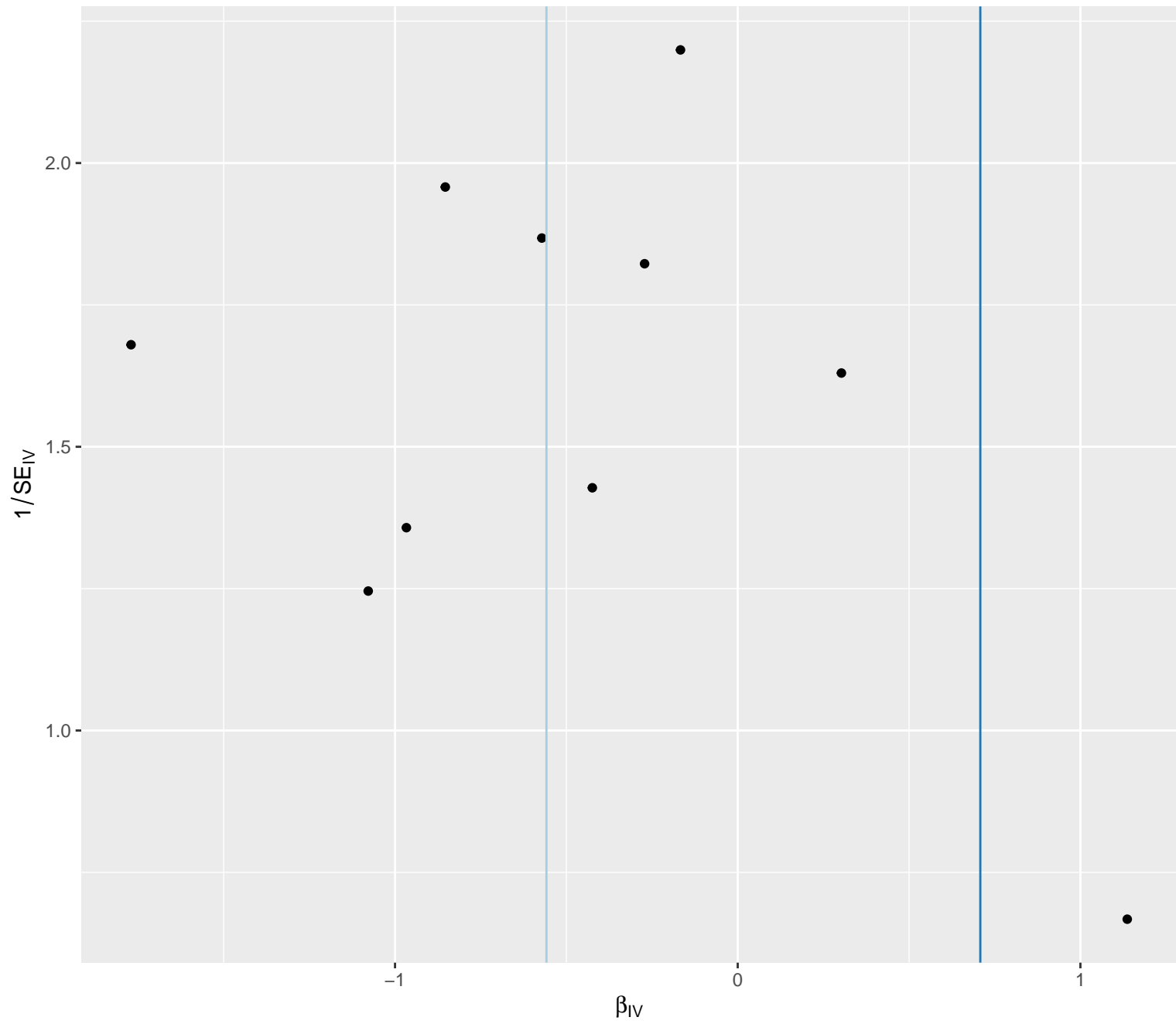

Supplement: Supplementary Data Sheet 1 — Harmonized summary data, forest plots, funnel plots, data sources, harmonization details, and sensitivity analyses for the Mendelian randomization analysis of pyroptosis-related proteins and ulcerative colitis. [file DataSheet1.zip › bdpqtlresult/13985_12_SMURF2_SMUF2/funnelplot.pdf]

# MR Test

- Inverse variance weighted
- MR Egger
- Simple mode
- Weighted median
- Weighted mode

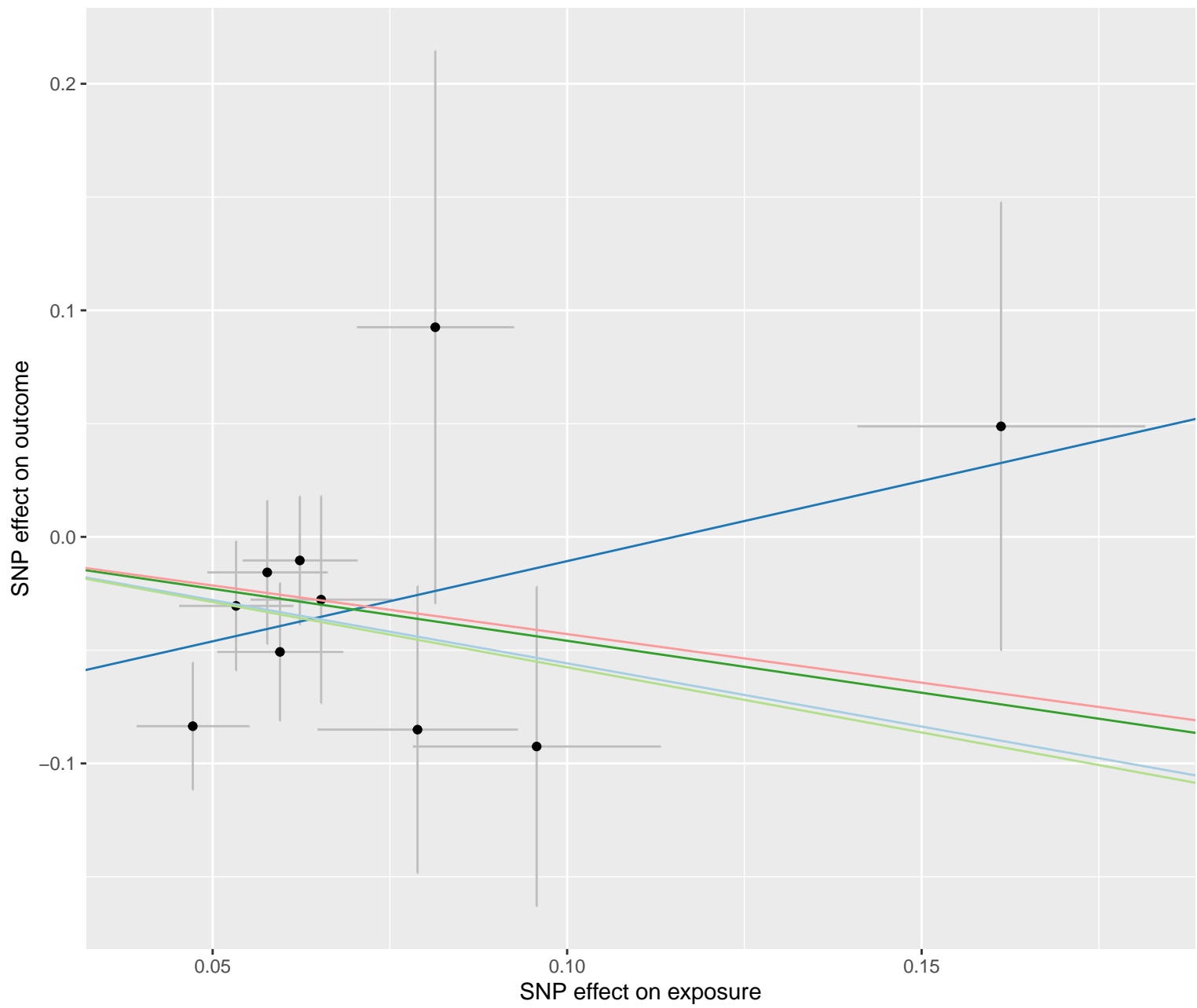

Supplement: Supplementary Data Sheet 1 — Harmonized summary data, forest plots, funnel plots, data sources, harmonization details, and sensitivity analyses for the Mendelian randomization analysis of pyroptosis-related proteins and ulcerative colitis. [file DataSheet1.zip › bdpqtlresult/13985_12_SMURF2_SMUF2/scatter.pdf]

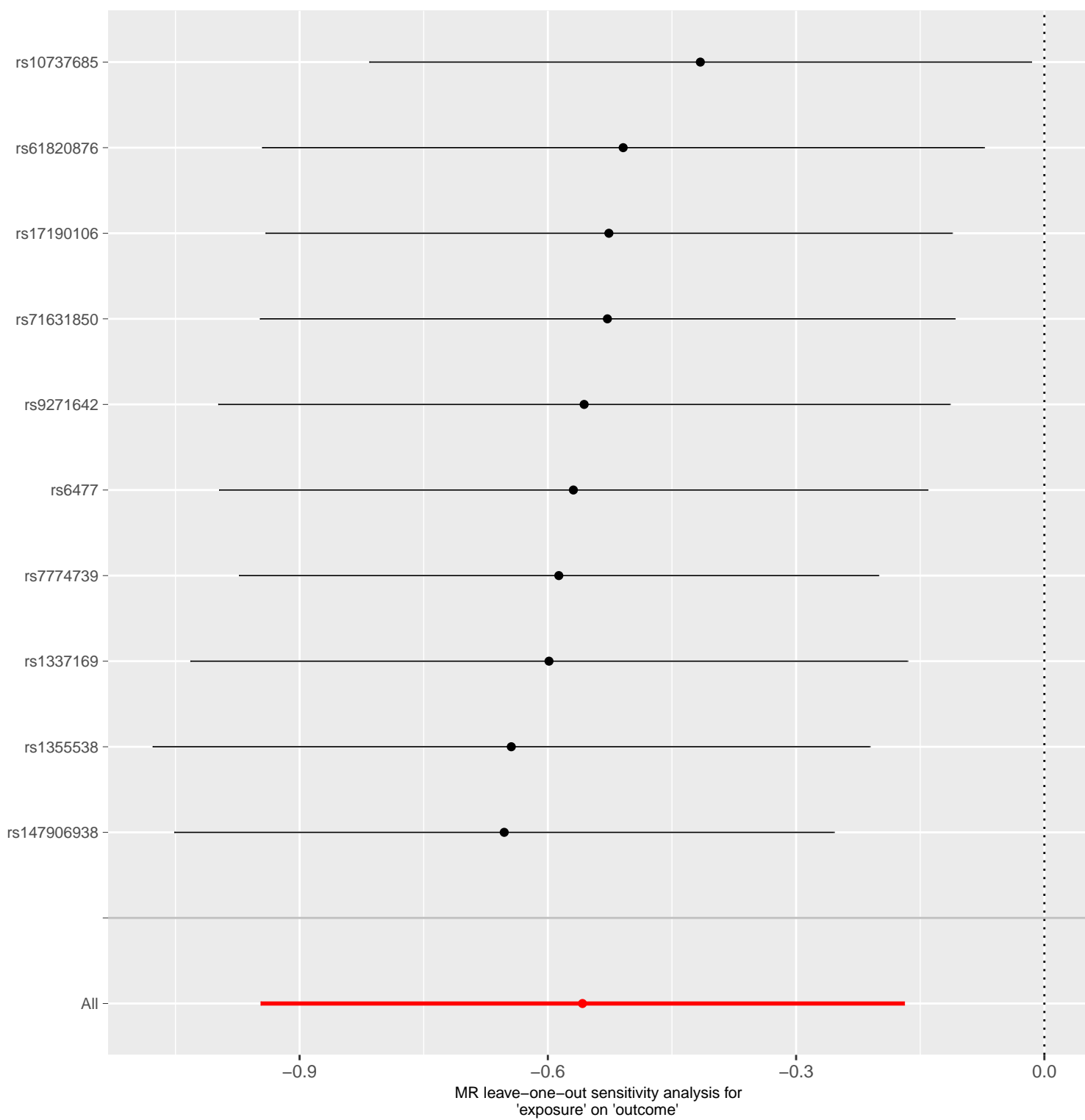

Supplement: Supplementary Data Sheet 1 — Harmonized summary data, forest plots, funnel plots, data sources, harmonization details, and sensitivity analyses for the Mendelian randomization analysis of pyroptosis-related proteins and ulcerative colitis. [file DataSheet1.zip › bdpqtlresult/13985_12_SMURF2_SMUF2/sensitivity-analysis.pdf]

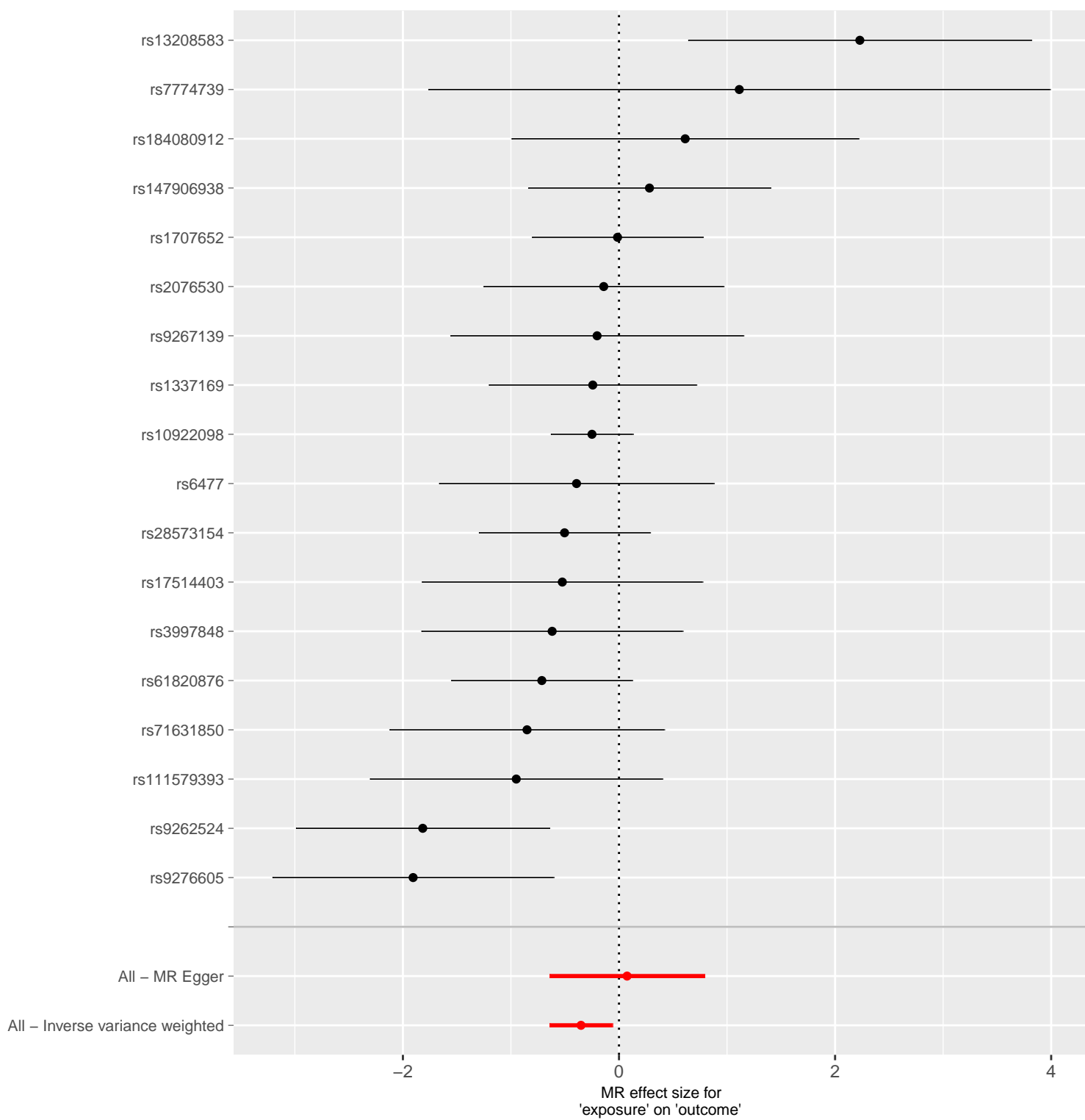

Supplement: Supplementary Data Sheet 1 — Harmonized summary data, forest plots, funnel plots, data sources, harmonization details, and sensitivity analyses for the Mendelian randomization analysis of pyroptosis-related proteins and ulcerative colitis. [file DataSheet1.zip › bdpqtlresult/15346_31_IFNG_IFN_g/forest.pdf]

# MR Method

- Inverse variance weighted
- MR Egger

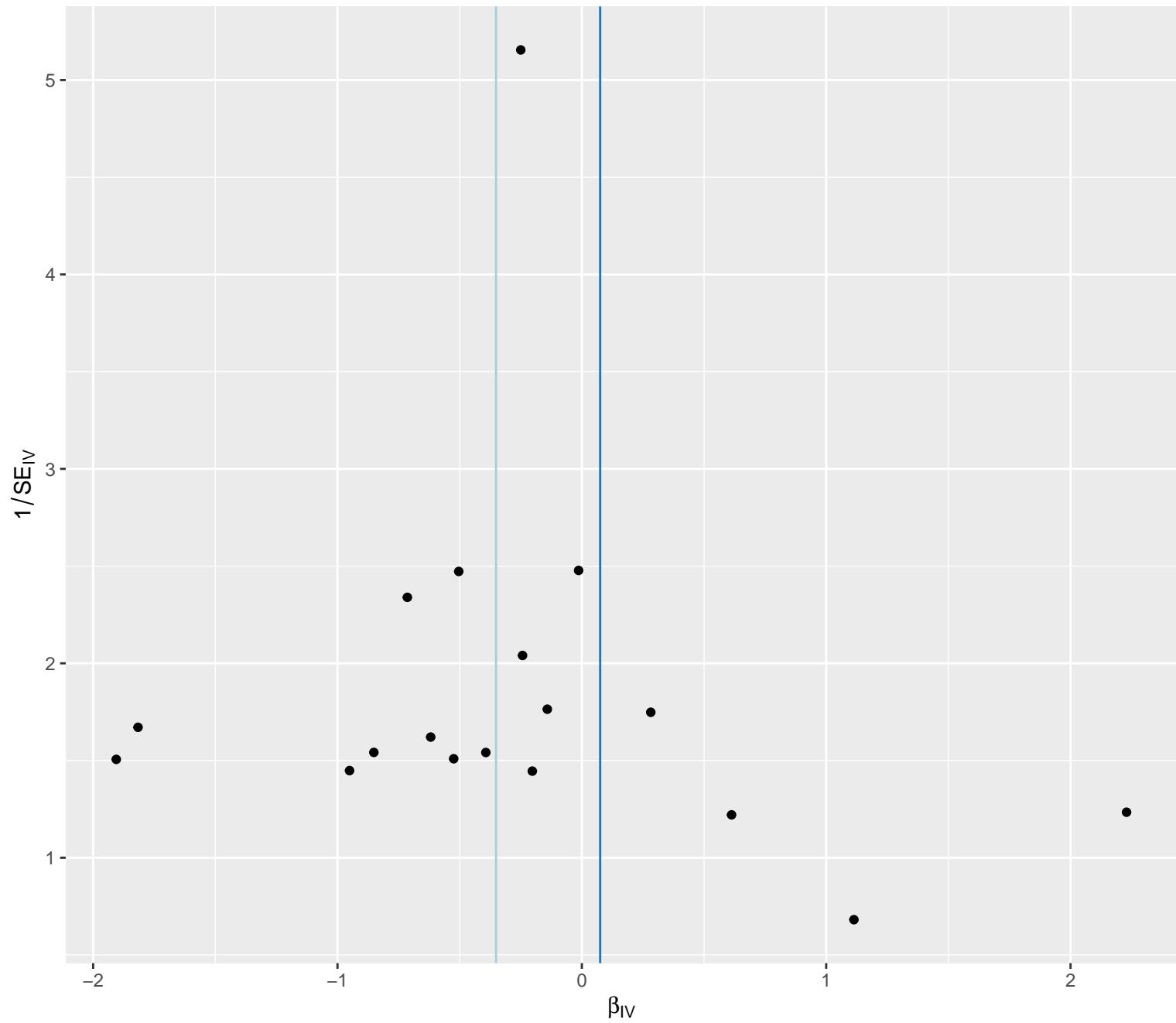

Supplement: Supplementary Data Sheet 1 — Harmonized summary data, forest plots, funnel plots, data sources, harmonization details, and sensitivity analyses for the Mendelian randomization analysis of pyroptosis-related proteins and ulcerative colitis. [file DataSheet1.zip › bdpqtlresult/15346_31_IFNG_IFN_g/funnelplot.pdf]

# MR Test

- Inverse variance weighted
- MR Egger
- Simple mode
- Weighted median
- Weighted mode

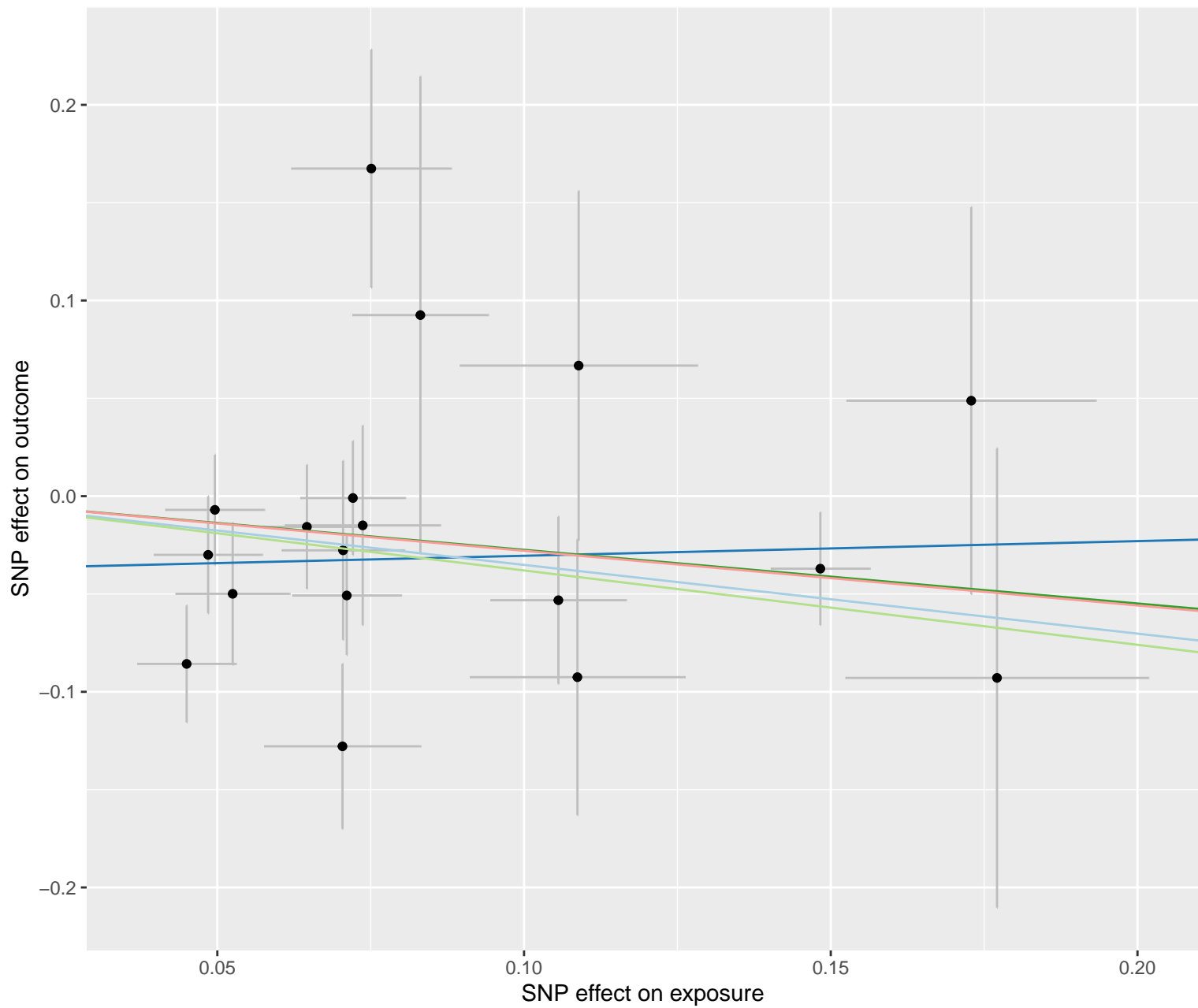

Supplement: Supplementary Data Sheet 1 — Harmonized summary data, forest plots, funnel plots, data sources, harmonization details, and sensitivity analyses for the Mendelian randomization analysis of pyroptosis-related proteins and ulcerative colitis. [file DataSheet1.zip › bdpqtlresult/15346_31_IFNG_IFN_g/scatter.pdf]

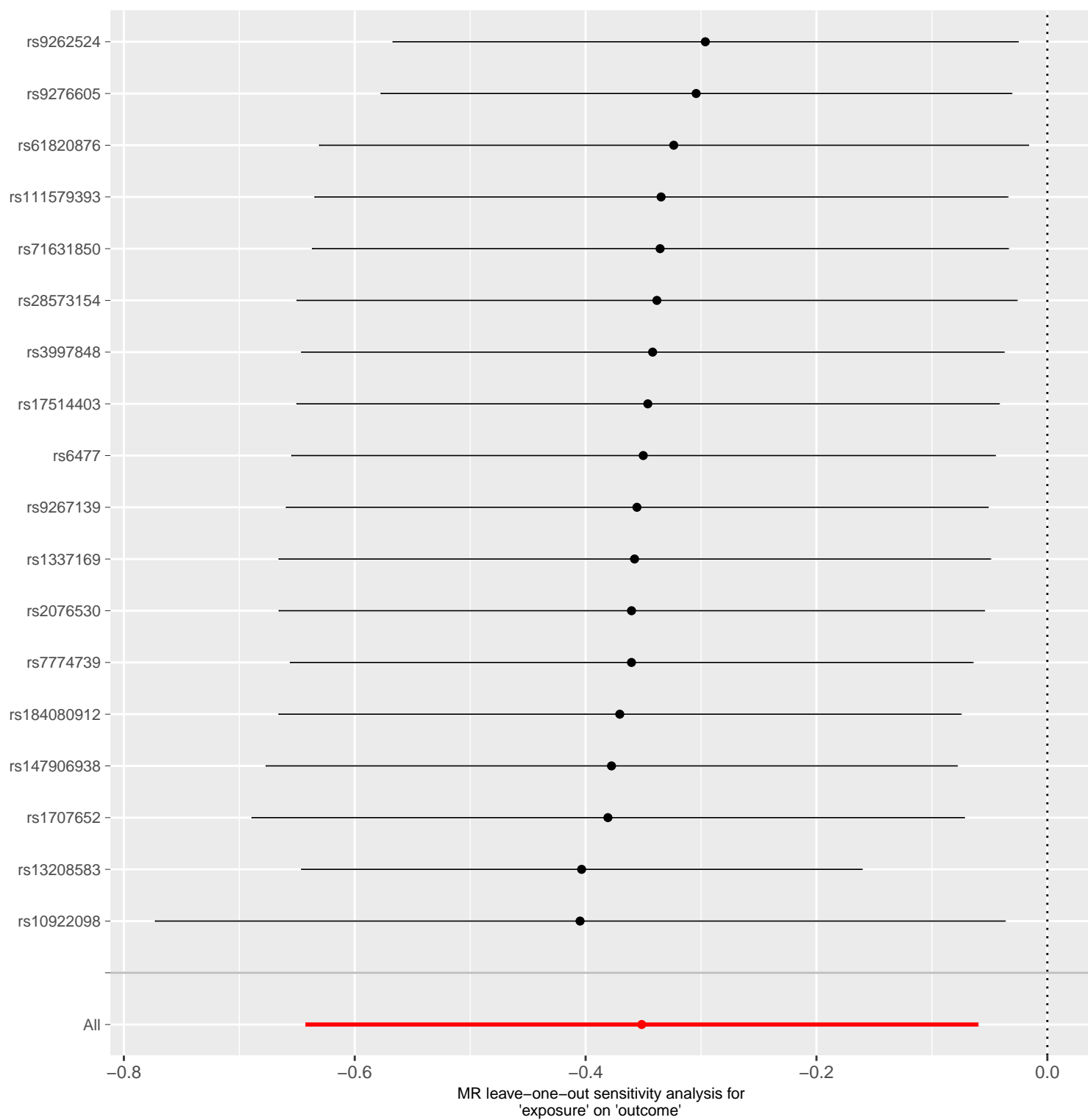

Supplement: Supplementary Data Sheet 1 — Harmonized summary data, forest plots, funnel plots, data sources, harmonization details, and sensitivity analyses for the Mendelian randomization analysis of pyroptosis-related proteins and ulcerative colitis. [file DataSheet1.zip › bdpqtlresult/15346_31_IFNG_IFN_g/sensitivity-analysis.pdf]

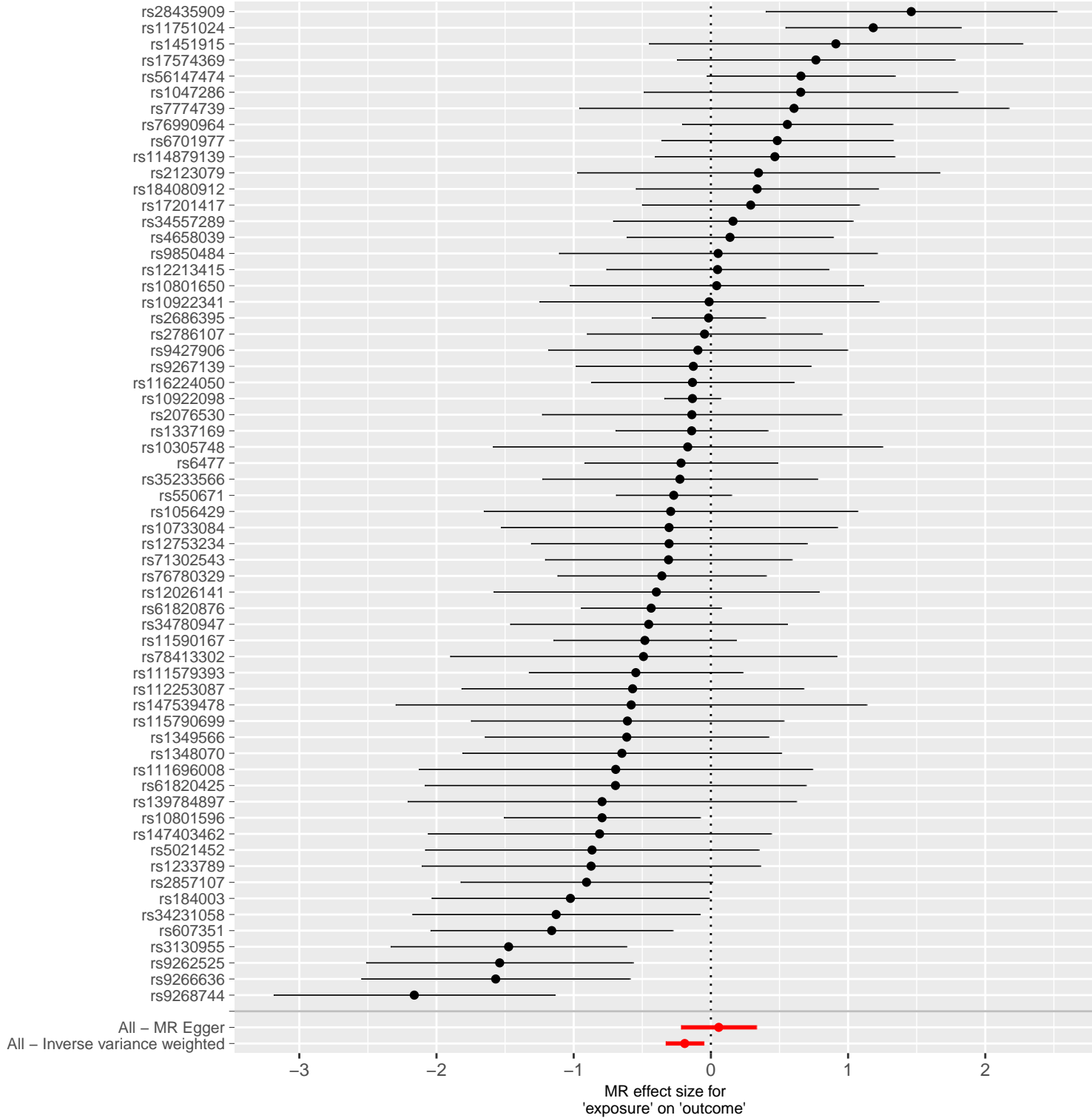

Supplement: Supplementary Data Sheet 1 — Harmonized summary data, forest plots, funnel plots, data sources, harmonization details, and sensitivity analyses for the Mendelian randomization analysis of pyroptosis-related proteins and ulcerative colitis. [file DataSheet1.zip › bdpqtlresult/15675_3_CEBPB_CEBPB/forest.pdf]

# MR Method

- Inverse variance weighted
- MR Egger

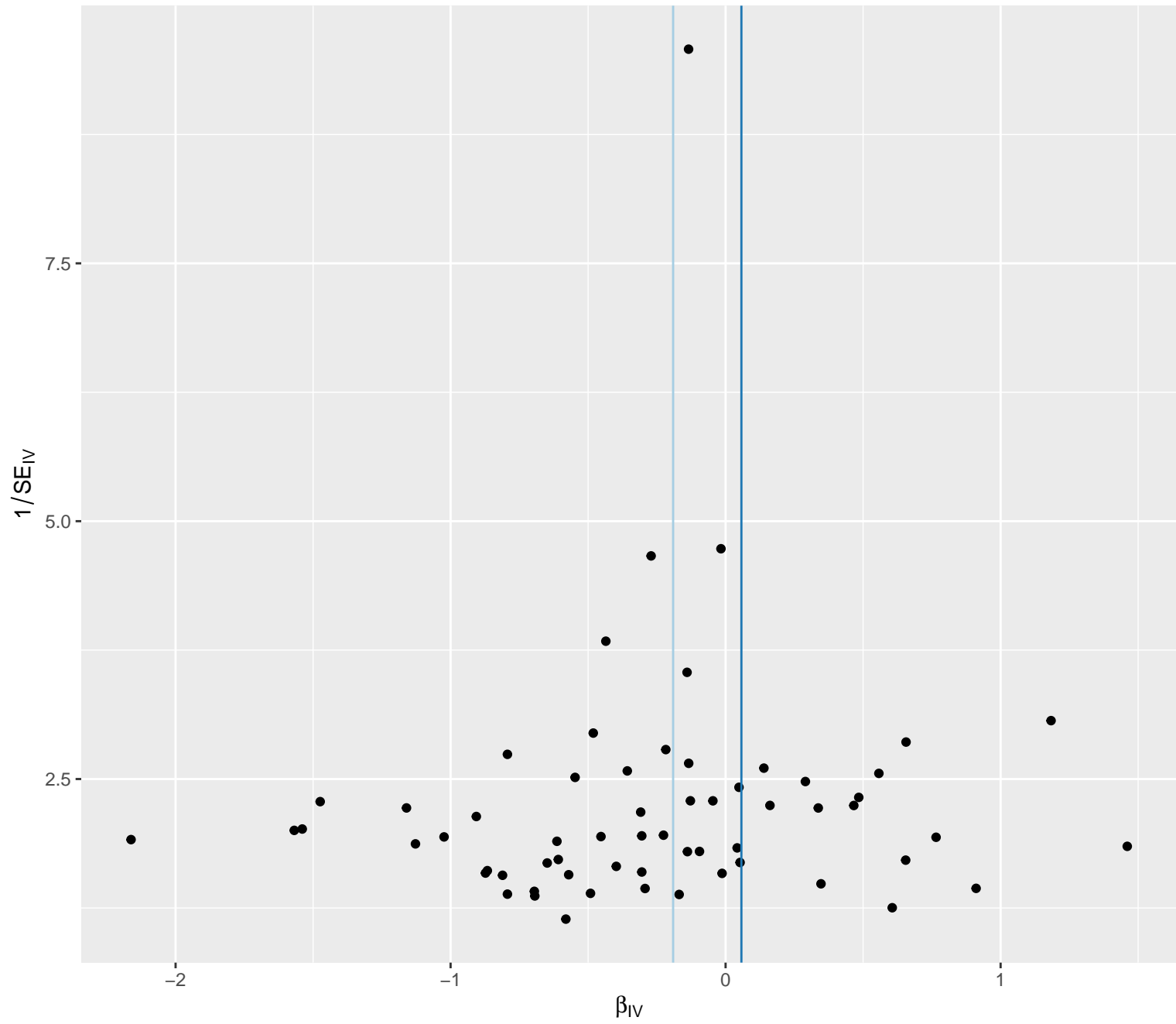

Supplement: Supplementary Data Sheet 1 — Harmonized summary data, forest plots, funnel plots, data sources, harmonization details, and sensitivity analyses for the Mendelian randomization analysis of pyroptosis-related proteins and ulcerative colitis. [file DataSheet1.zip › bdpqtlresult/15675_3_CEBPB_CEBPB/funnelplot.pdf]

# MR Test

- Inverse variance weighted
- MR Egger
- Simple mode
- Weighted median
- Weighted mode

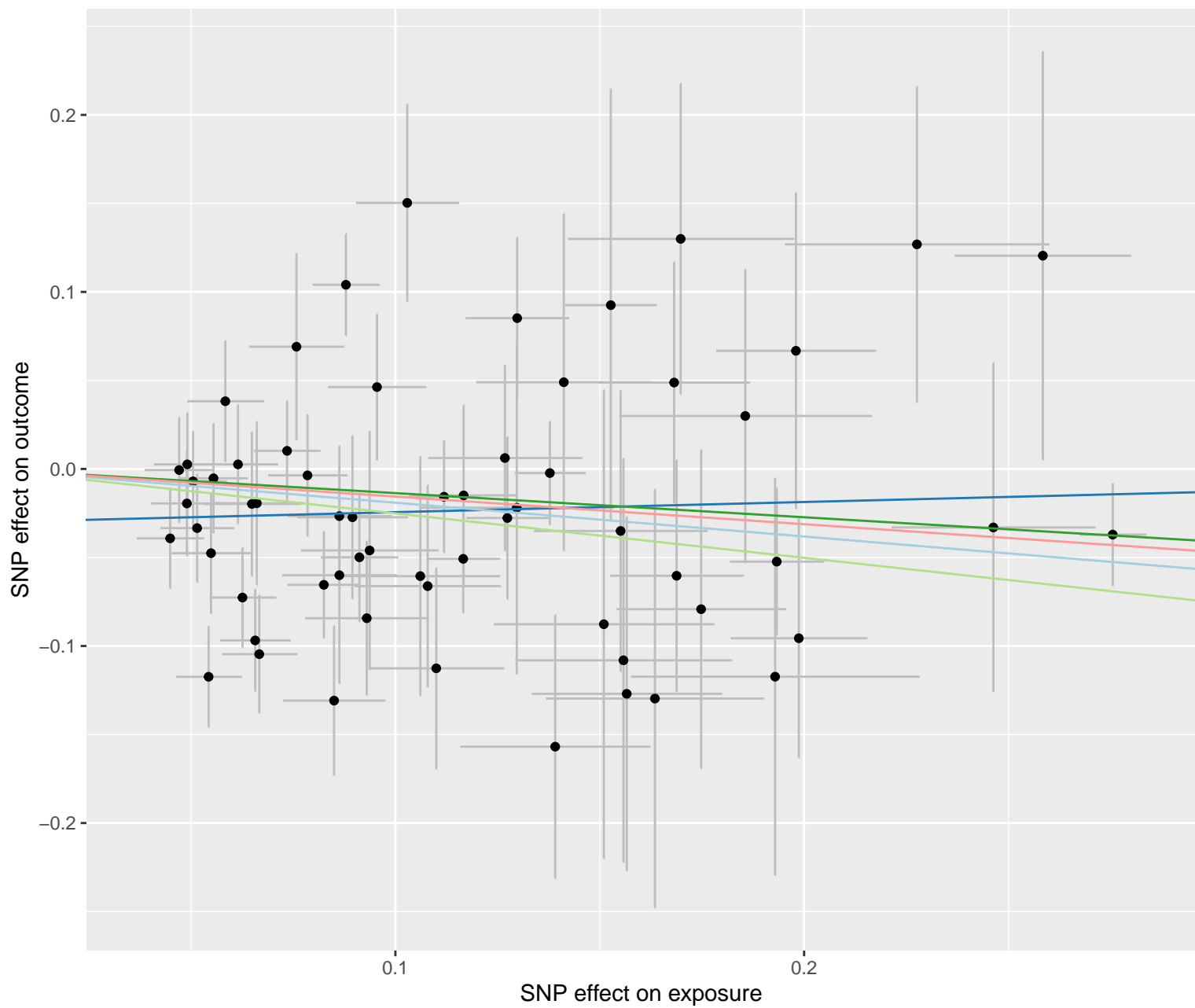

Supplement: Supplementary Data Sheet 1 — Harmonized summary data, forest plots, funnel plots, data sources, harmonization details, and sensitivity analyses for the Mendelian randomization analysis of pyroptosis-related proteins and ulcerative colitis. [file DataSheet1.zip › bdpqtlresult/15675_3_CEBPB_CEBPB/scatter.pdf]

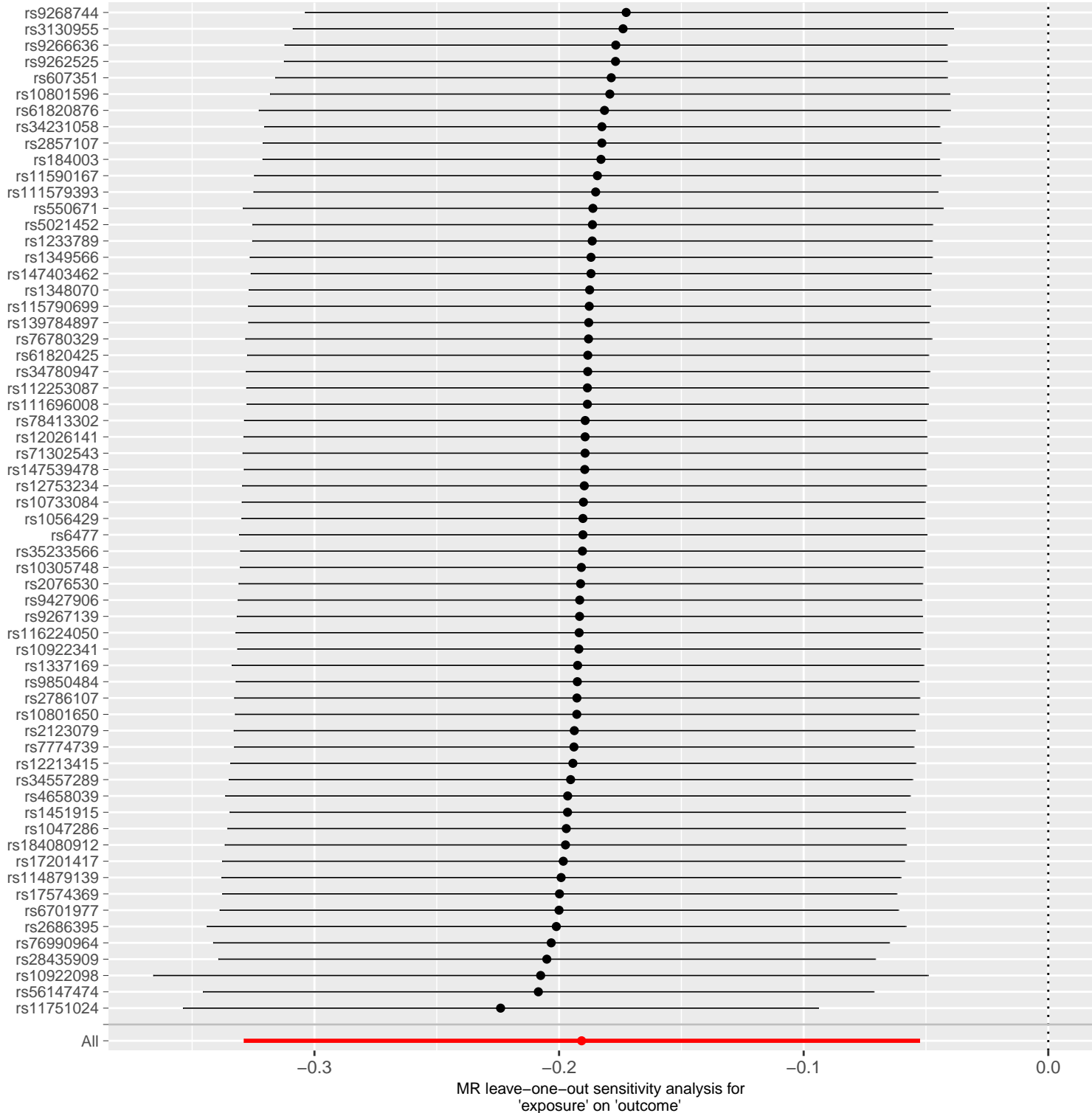

Supplement: Supplementary Data Sheet 1 — Harmonized summary data, forest plots, funnel plots, data sources, harmonization details, and sensitivity analyses for the Mendelian randomization analysis of pyroptosis-related proteins and ulcerative colitis. [file DataSheet1.zip › bdpqtlresult/15675_3_CEBPB_CEBPB/sensitivity-analysis.pdf]

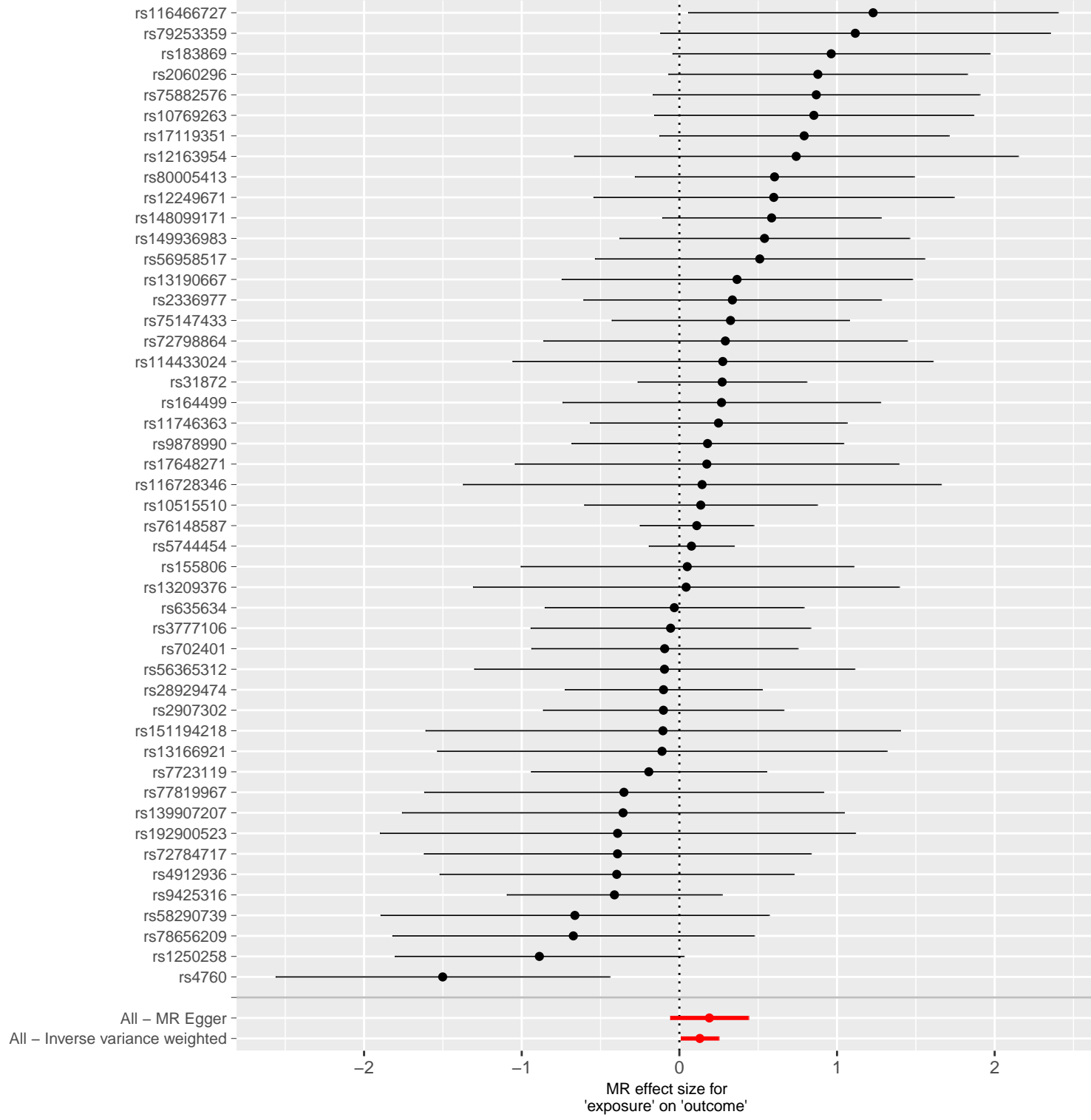

Supplement: Supplementary Data Sheet 1 — Harmonized summary data, forest plots, funnel plots, data sources, harmonization details, and sensitivity analyses for the Mendelian randomization analysis of pyroptosis-related proteins and ulcerative colitis. [file DataSheet1.zip › bdpqtlresult/16914_104_CD14_sCD14/forest.pdf]

# MR Method

- Inverse variance weighted
- MR Egger

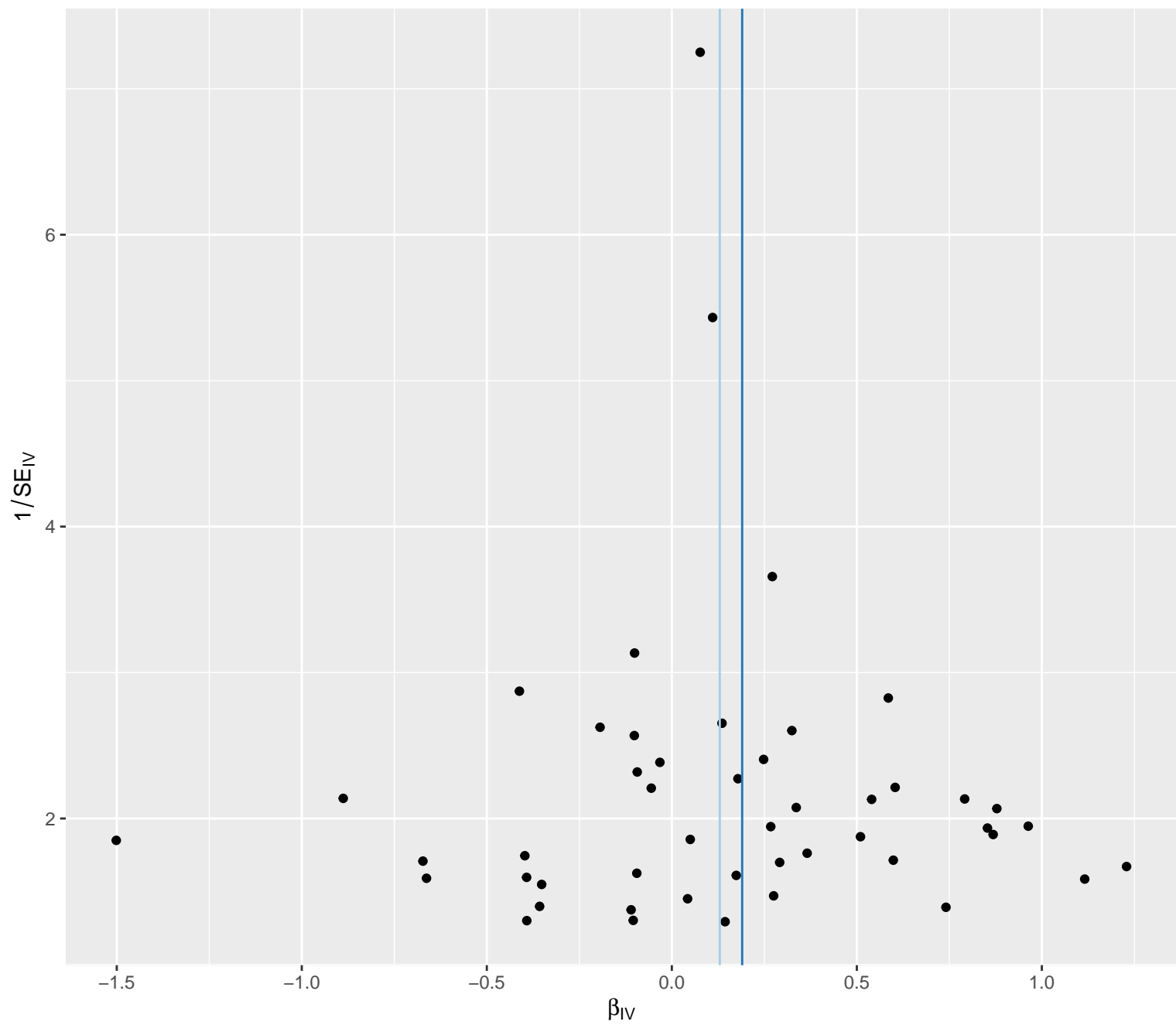

Supplement: Supplementary Data Sheet 1 — Harmonized summary data, forest plots, funnel plots, data sources, harmonization details, and sensitivity analyses for the Mendelian randomization analysis of pyroptosis-related proteins and ulcerative colitis. [file DataSheet1.zip › bdpqtlresult/16914_104_CD14_sCD14/funnelplot.pdf]

# MR Test

- Inverse variance weighted
- MR Egger
- Simple mode
- Weighted median
- Weighted mode

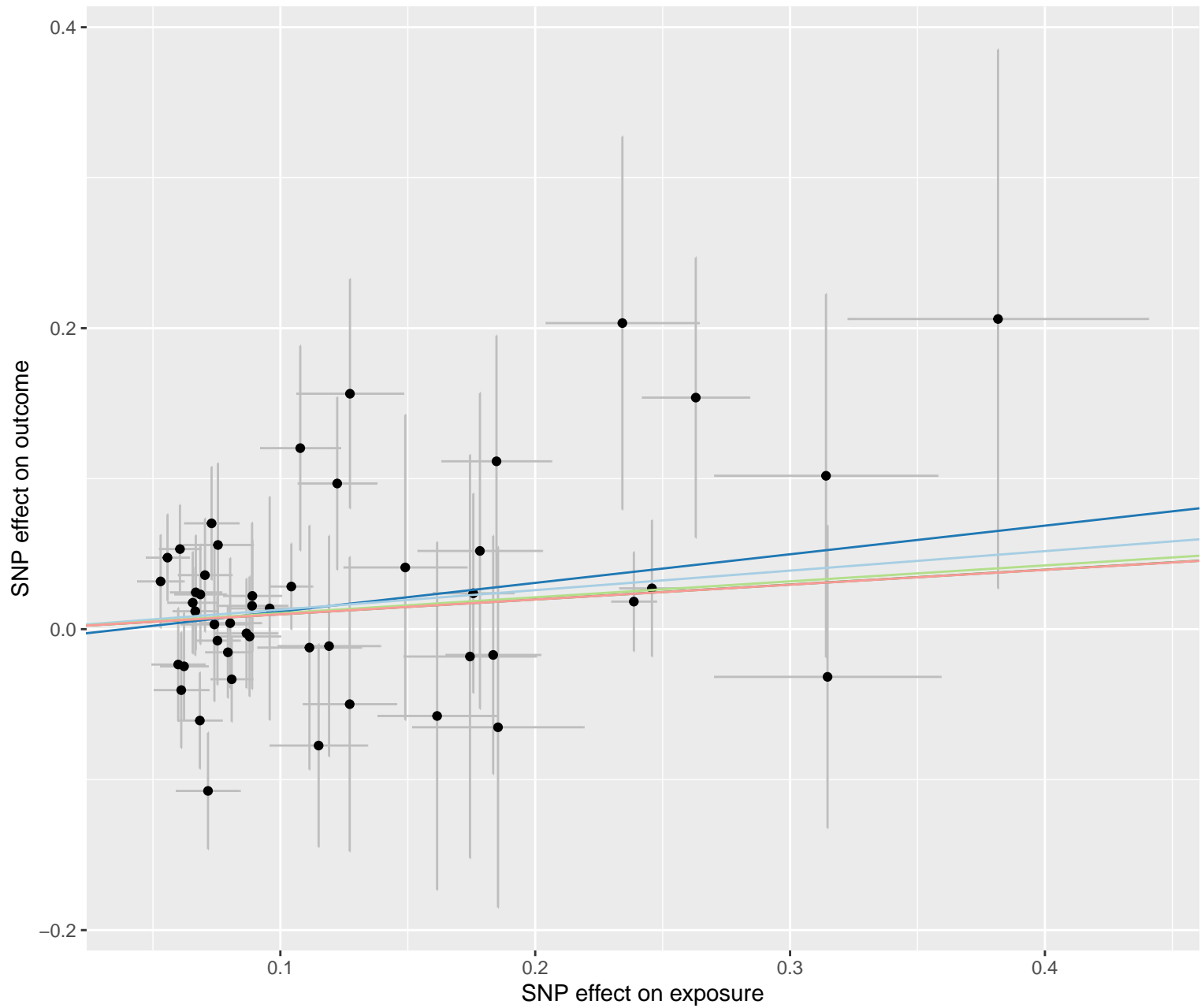

Supplement: Supplementary Data Sheet 1 — Harmonized summary data, forest plots, funnel plots, data sources, harmonization details, and sensitivity analyses for the Mendelian randomization analysis of pyroptosis-related proteins and ulcerative colitis. [file DataSheet1.zip › bdpqtlresult/16914_104_CD14_sCD14/scatter.pdf]

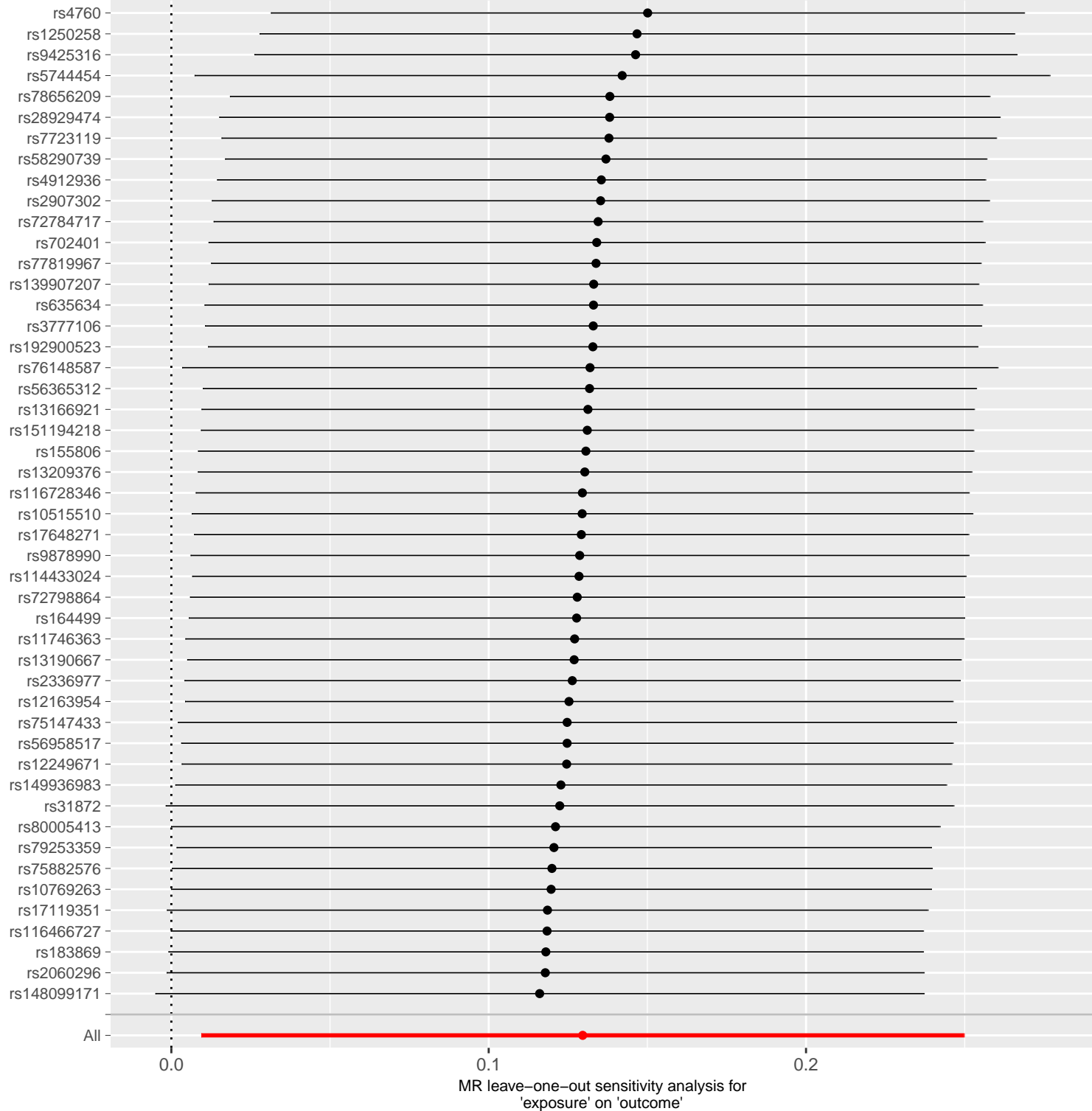

Supplement: Supplementary Data Sheet 1 — Harmonized summary data, forest plots, funnel plots, data sources, harmonization details, and sensitivity analyses for the Mendelian randomization analysis of pyroptosis-related proteins and ulcerative colitis. [file DataSheet1.zip › bdpqtlresult/16914_104_CD14_sCD14/sensitivity-analysis.pdf]

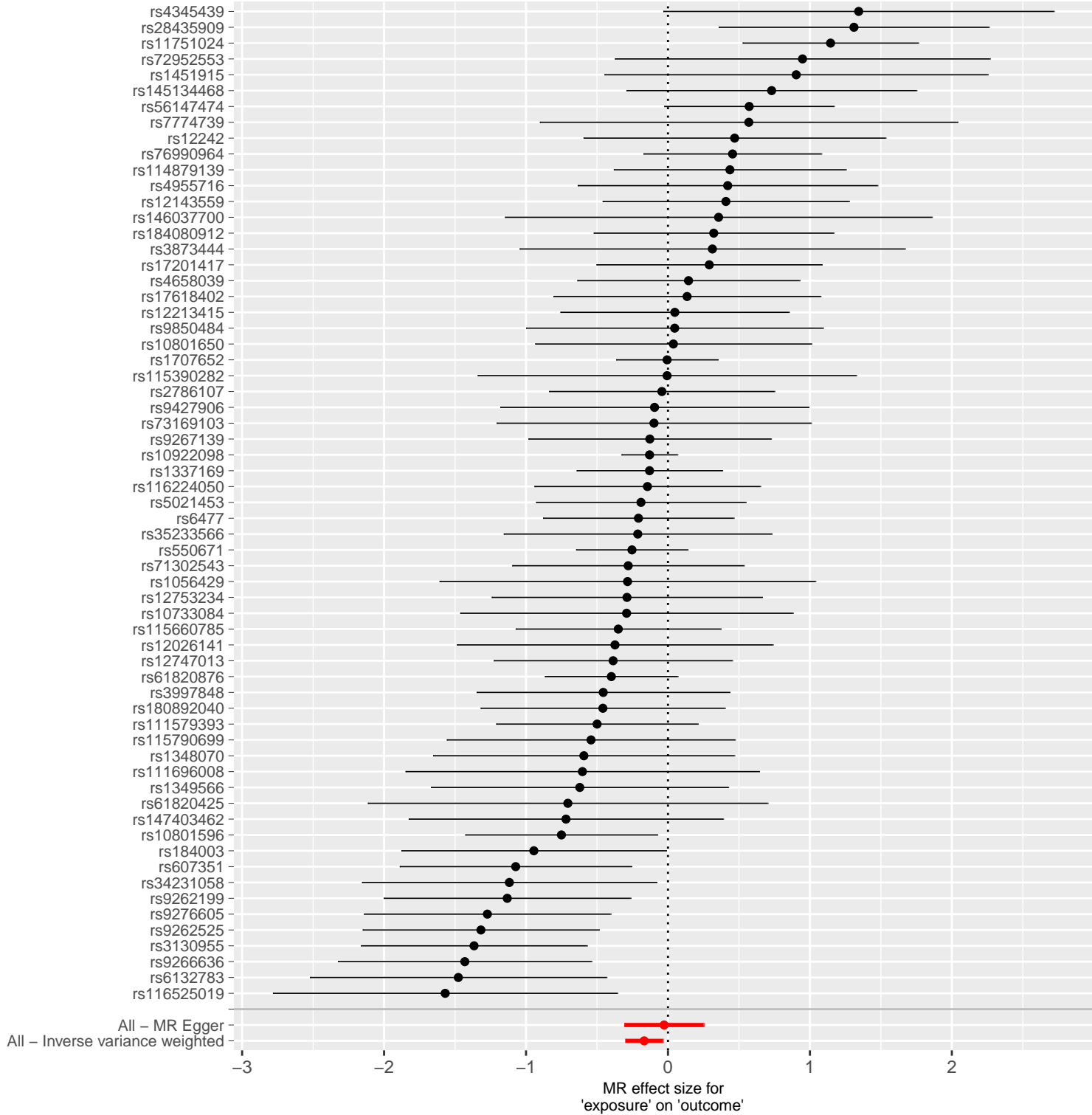

Supplement: Supplementary Data Sheet 1 — Harmonized summary data, forest plots, funnel plots, data sources, harmonization details, and sensitivity analyses for the Mendelian randomization analysis of pyroptosis-related proteins and ulcerative colitis. [file DataSheet1.zip › bdpqtlresult/17155_1_VPS28_VPS28_protein_homolog/forest.pdf]

# MR Method

- Inverse variance weighted
- MR Egger

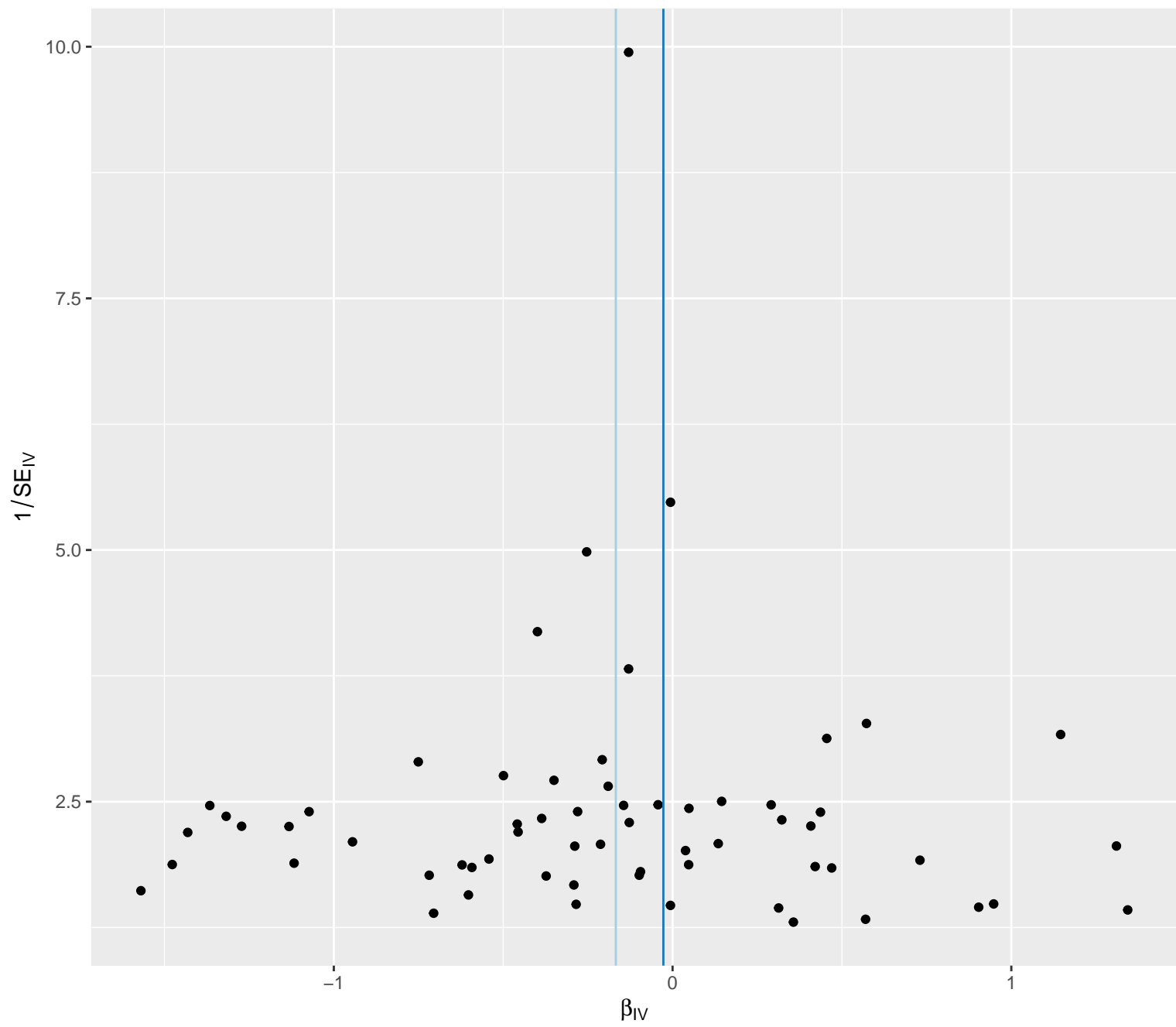

Supplement: Supplementary Data Sheet 1 — Harmonized summary data, forest plots, funnel plots, data sources, harmonization details, and sensitivity analyses for the Mendelian randomization analysis of pyroptosis-related proteins and ulcerative colitis. [file DataSheet1.zip › bdpqtlresult/17155_1_VPS28_VPS28_protein_homolog/funnelplot.pdf]

# MR Test

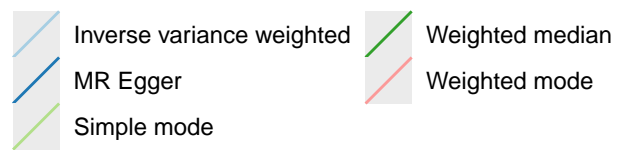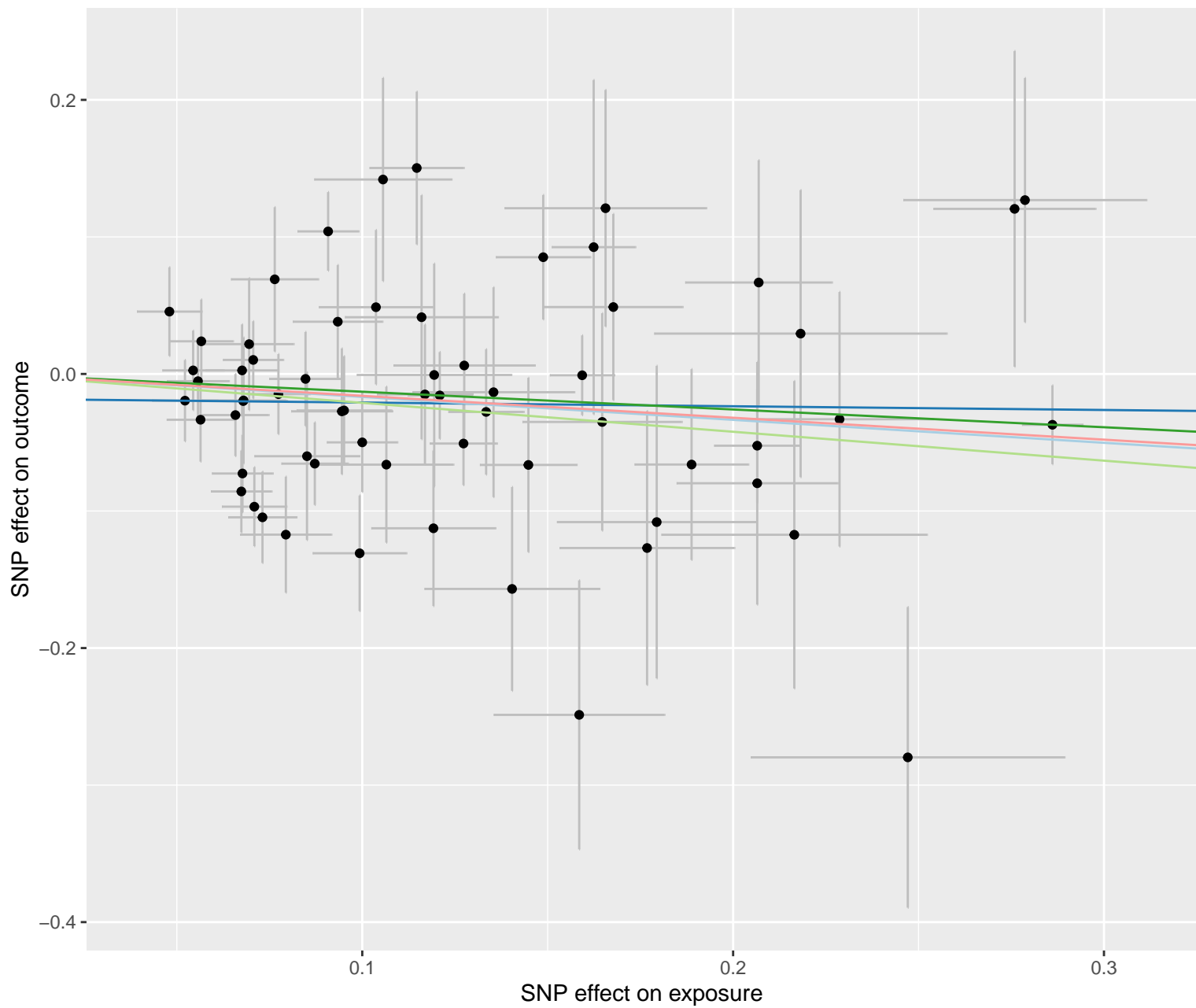

Supplement: Supplementary Data Sheet 1 — Harmonized summary data, forest plots, funnel plots, data sources, harmonization details, and sensitivity analyses for the Mendelian randomization analysis of pyroptosis-related proteins and ulcerative colitis. [file DataSheet1.zip › bdpqtlresult/17155_1_VPS28_VPS28_protein_homolog/scatter.pdf]

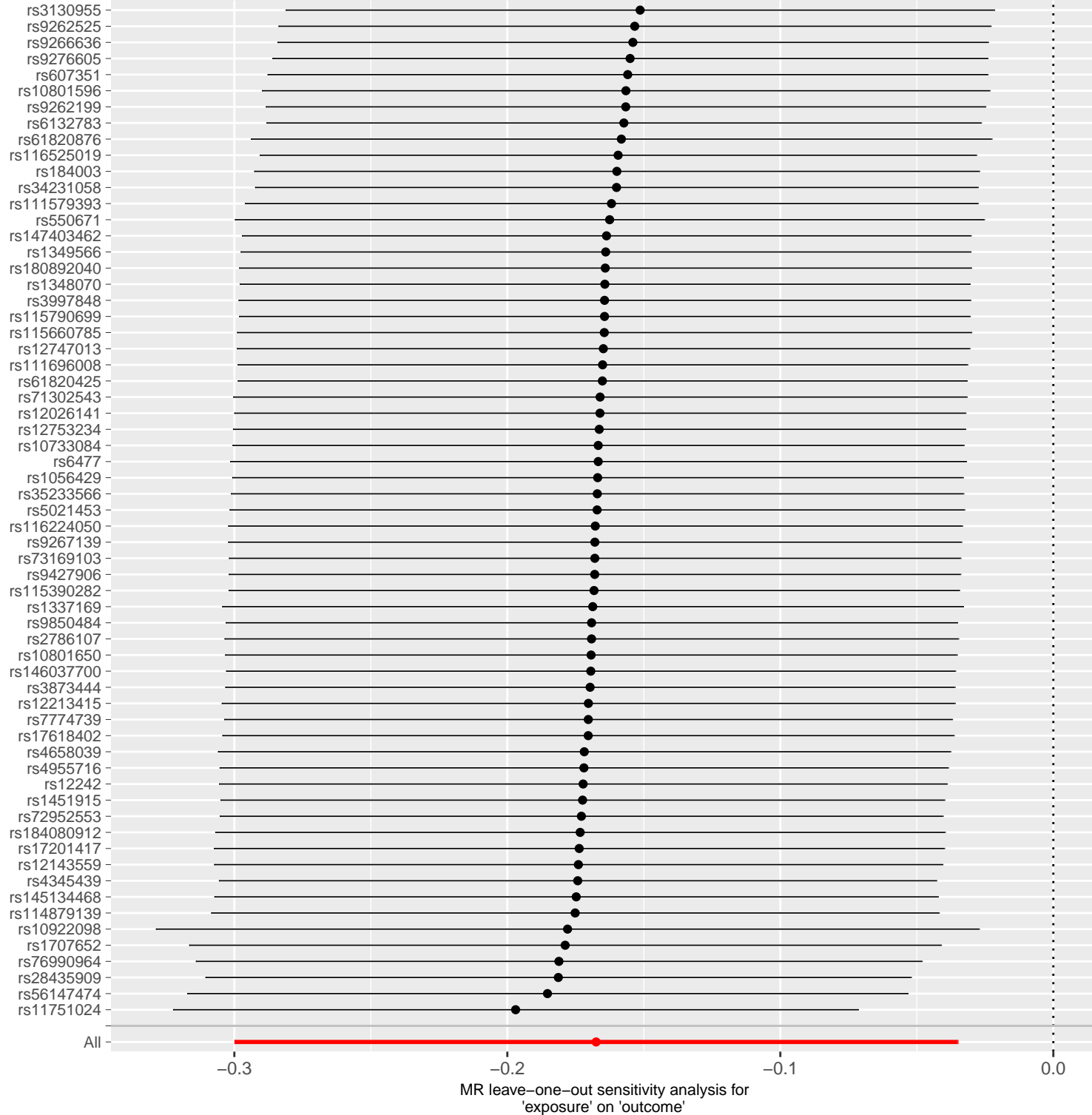

Supplement: Supplementary Data Sheet 1 — Harmonized summary data, forest plots, funnel plots, data sources, harmonization details, and sensitivity analyses for the Mendelian randomization analysis of pyroptosis-related proteins and ulcerative colitis. [file DataSheet1.zip › bdpqtlresult/17155_1_VPS28_VPS28_protein_homolog/sensitivity-analysis.pdf]

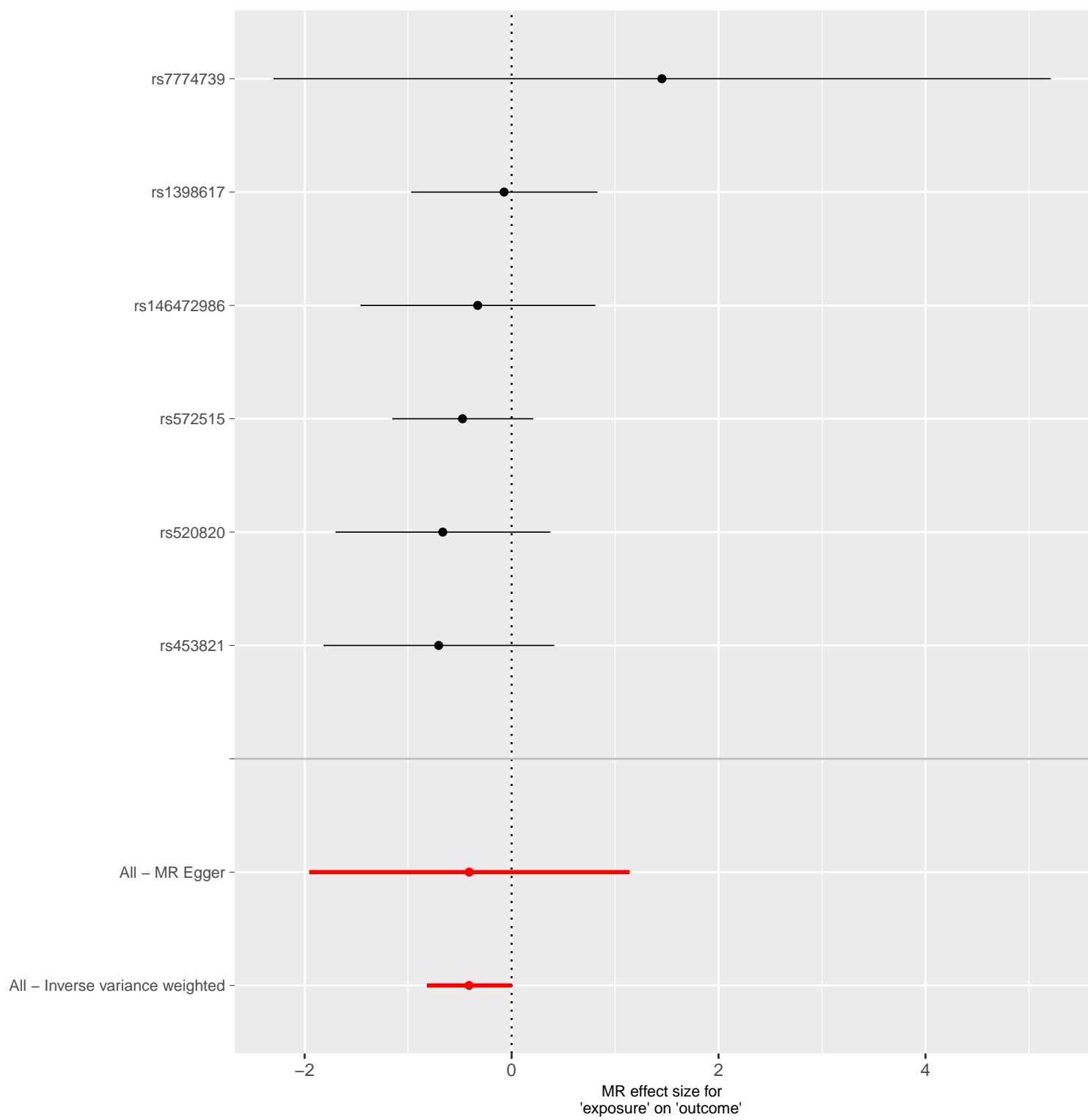

Supplement: Supplementary Data Sheet 1 — Harmonized summary data, forest plots, funnel plots, data sources, harmonization details, and sensitivity analyses for the Mendelian randomization analysis of pyroptosis-related proteins and ulcerative colitis. [file DataSheet1.zip › bdpqtlresult/17175_5_MAP2K6_MP2K6/forest.pdf]

# MR Method

- Inverse variance weighted
- MR Egger

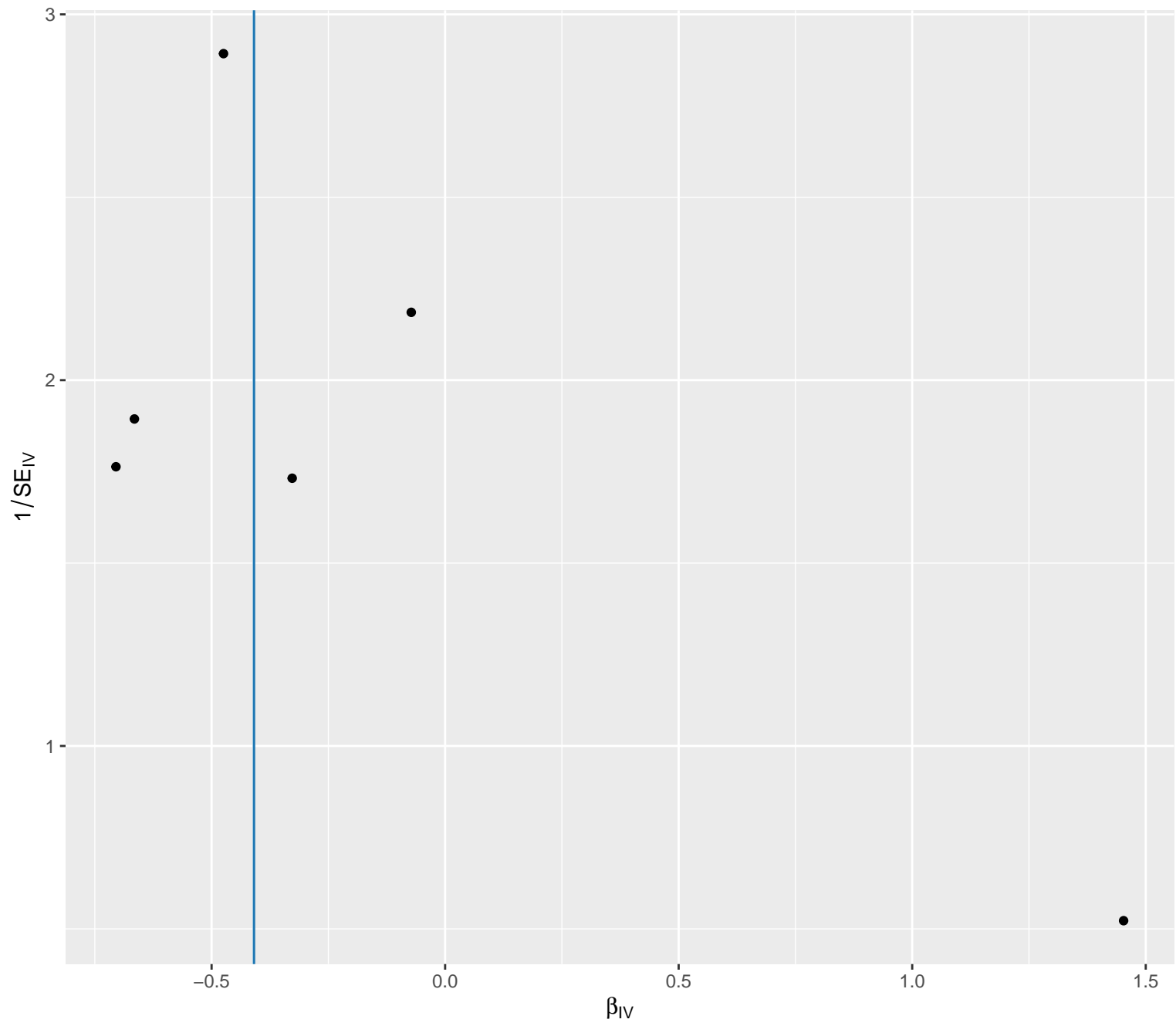

Supplement: Supplementary Data Sheet 1 — Harmonized summary data, forest plots, funnel plots, data sources, harmonization details, and sensitivity analyses for the Mendelian randomization analysis of pyroptosis-related proteins and ulcerative colitis. [file DataSheet1.zip › bdpqtlresult/17175_5_MAP2K6_MP2K6/funnelplot.pdf]

# MR Test

- Inverse variance weighted
- MR Egger
- Simple mode
- Weighted median
- Weighted mode

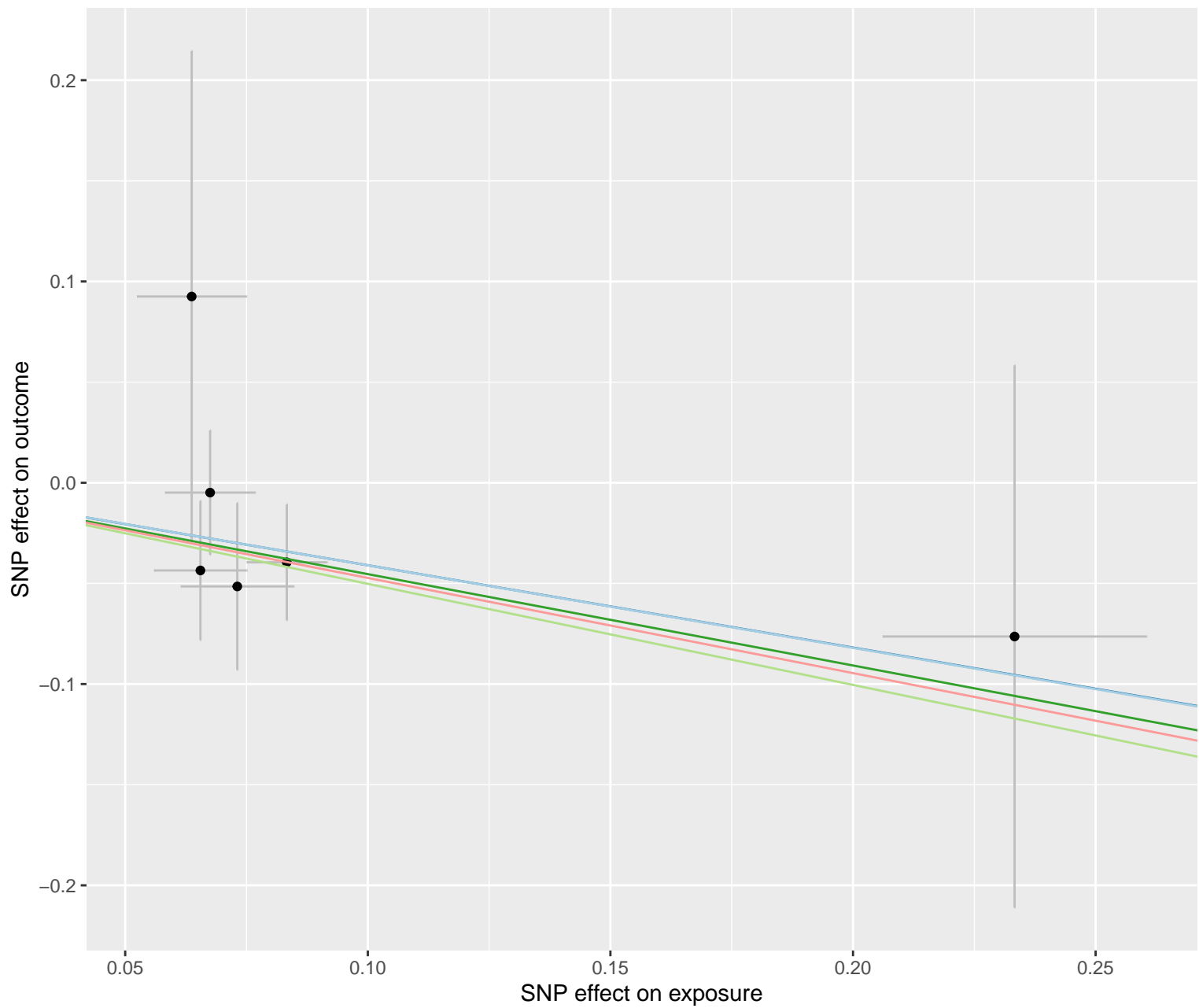

Supplement: Supplementary Data Sheet 1 — Harmonized summary data, forest plots, funnel plots, data sources, harmonization details, and sensitivity analyses for the Mendelian randomization analysis of pyroptosis-related proteins and ulcerative colitis. [file DataSheet1.zip › bdpqtlresult/17175_5_MAP2K6_MP2K6/scatter.pdf]

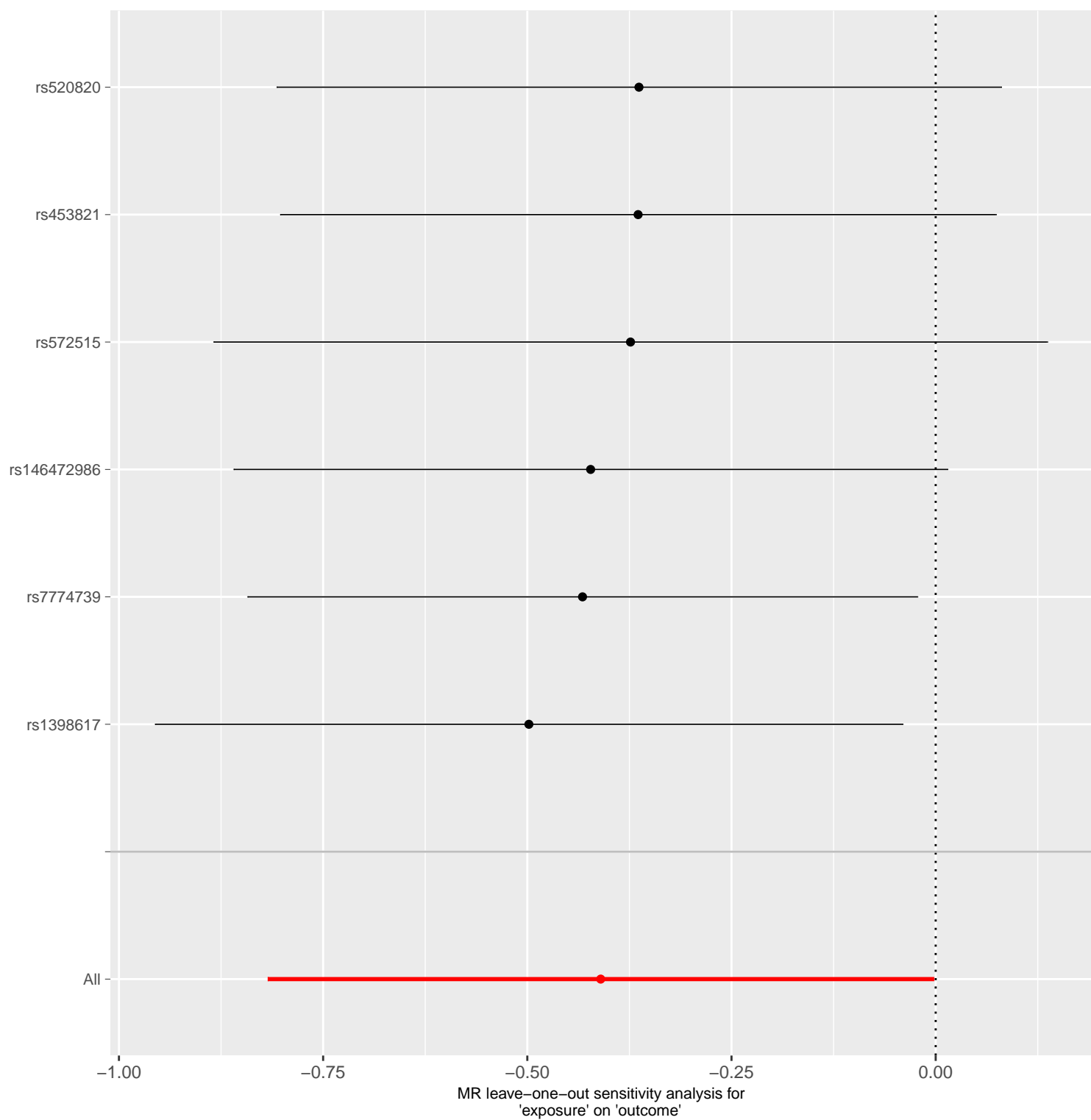

Supplement: Supplementary Data Sheet 1 — Harmonized summary data, forest plots, funnel plots, data sources, harmonization details, and sensitivity analyses for the Mendelian randomization analysis of pyroptosis-related proteins and ulcerative colitis. [file DataSheet1.zip › bdpqtlresult/17175_5_MAP2K6_MP2K6/sensitivity-analysis.pdf]

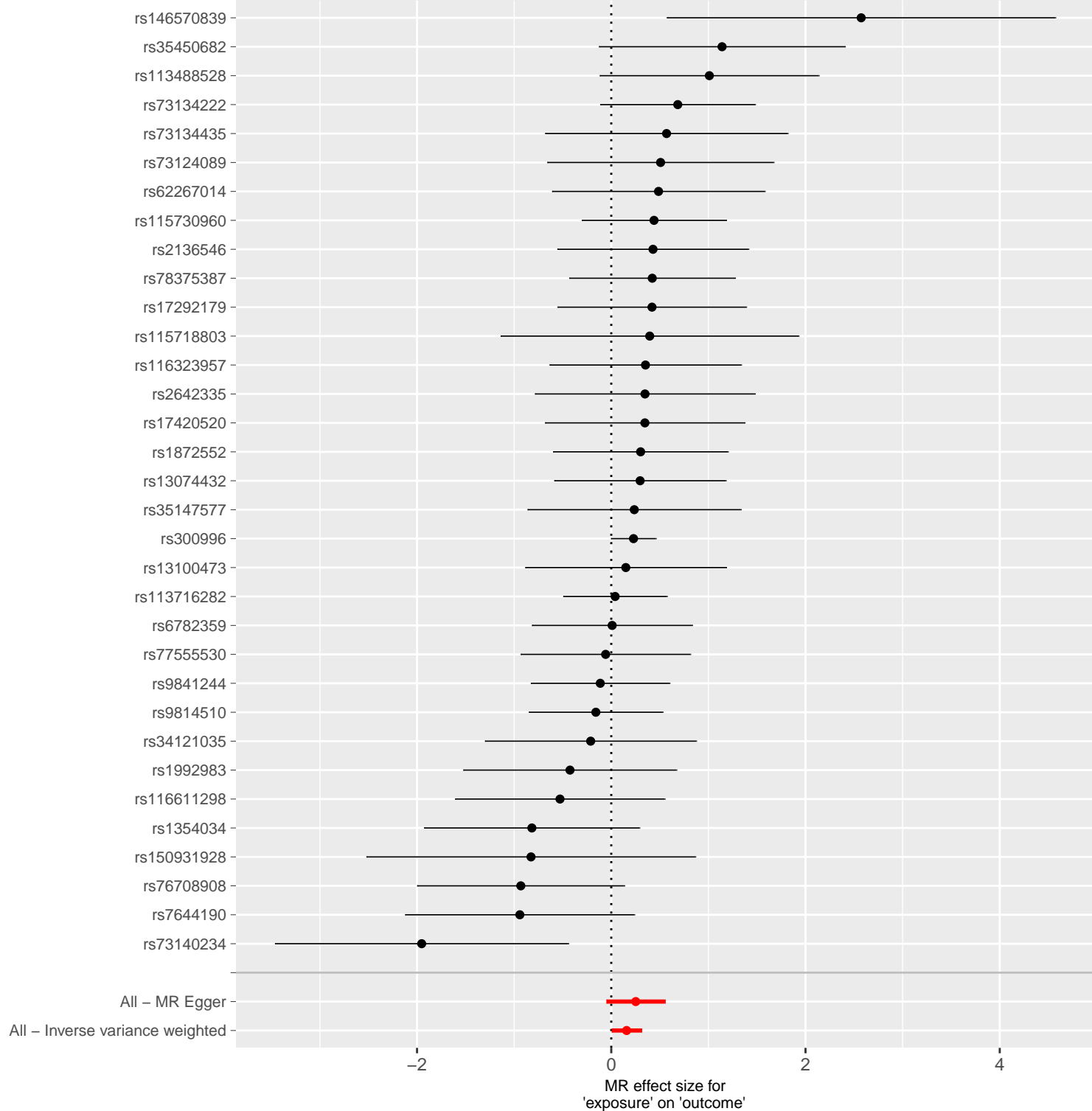

Supplement: Supplementary Data Sheet 1 — Harmonized summary data, forest plots, funnel plots, data sources, harmonization details, and sensitivity analyses for the Mendelian randomization analysis of pyroptosis-related proteins and ulcerative colitis. [file DataSheet1.zip › bdpqtlresult/17350_13_CHMP2B_CHM2B/forest.pdf]

# MR Method

- Inverse variance weighted
- MR Egger

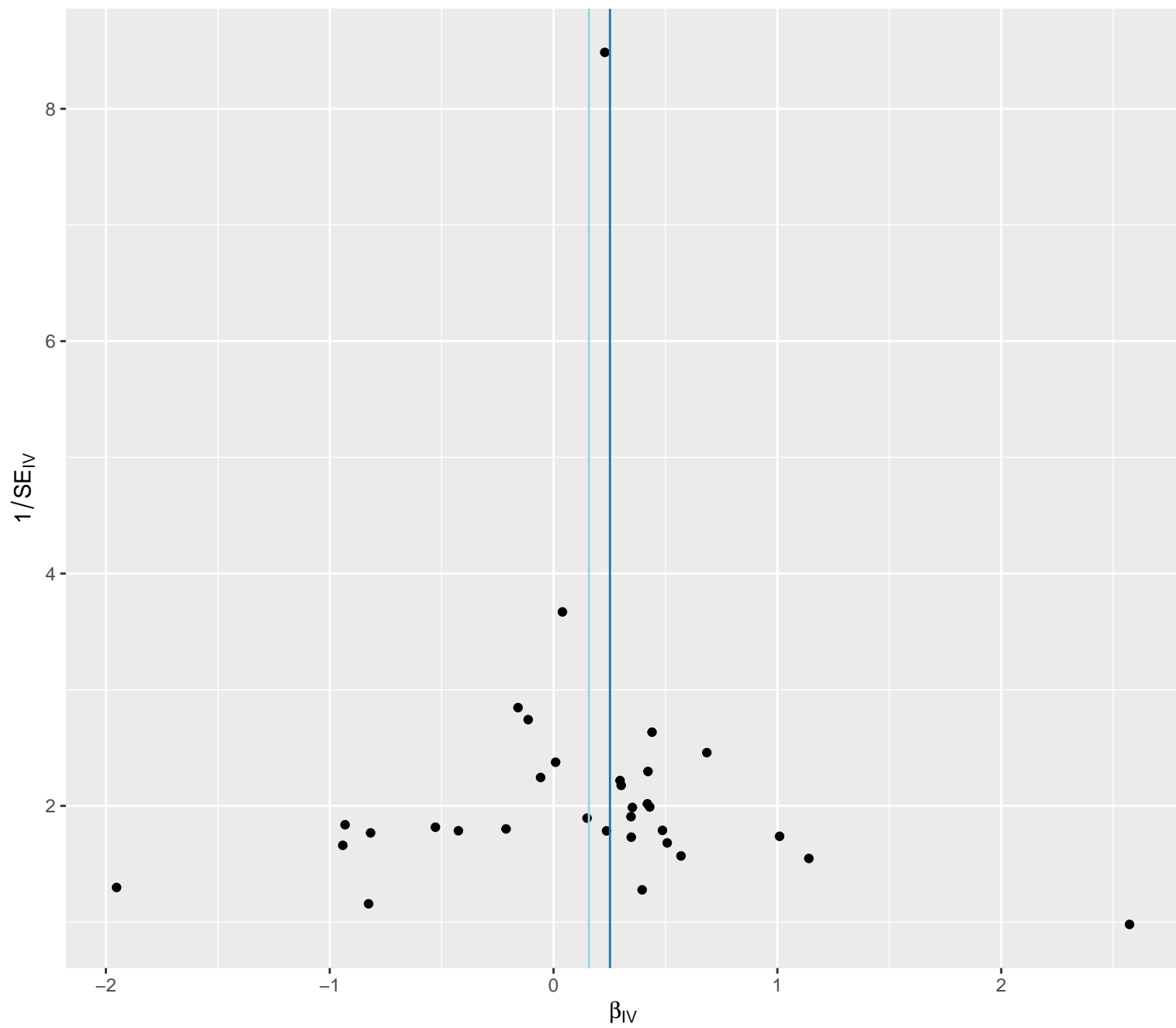

Supplement: Supplementary Data Sheet 1 — Harmonized summary data, forest plots, funnel plots, data sources, harmonization details, and sensitivity analyses for the Mendelian randomization analysis of pyroptosis-related proteins and ulcerative colitis. [file DataSheet1.zip › bdpqtlresult/17350_13_CHMP2B_CHM2B/funnelplot.pdf]

# MR Test

- Inverse variance weighted
- MR Egger
- Simple mode
- Weighted median
- Weighted mode

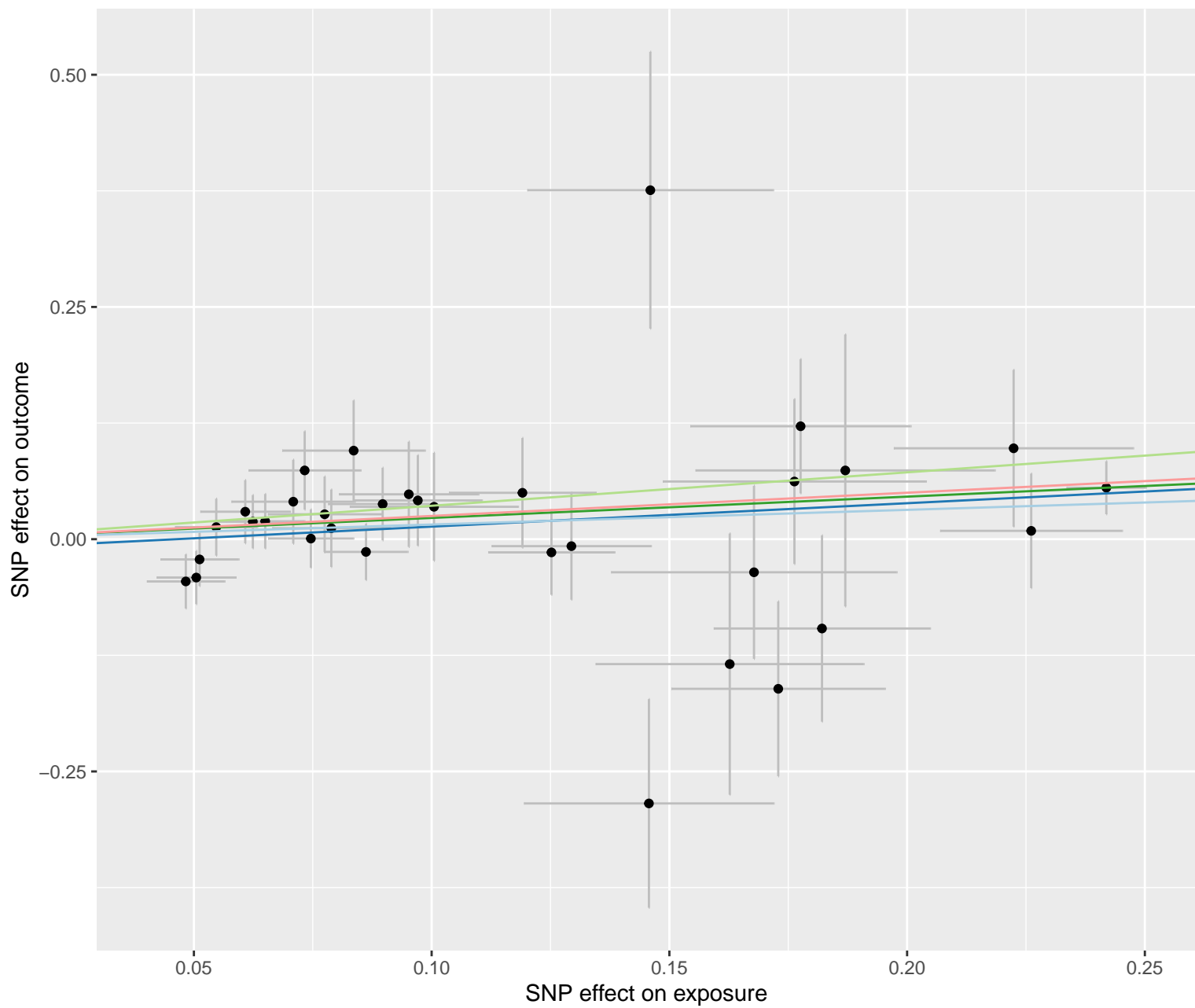

Supplement: Supplementary Data Sheet 1 — Harmonized summary data, forest plots, funnel plots, data sources, harmonization details, and sensitivity analyses for the Mendelian randomization analysis of pyroptosis-related proteins and ulcerative colitis. [file DataSheet1.zip › bdpqtlresult/17350_13_CHMP2B_CHM2B/scatter.pdf]

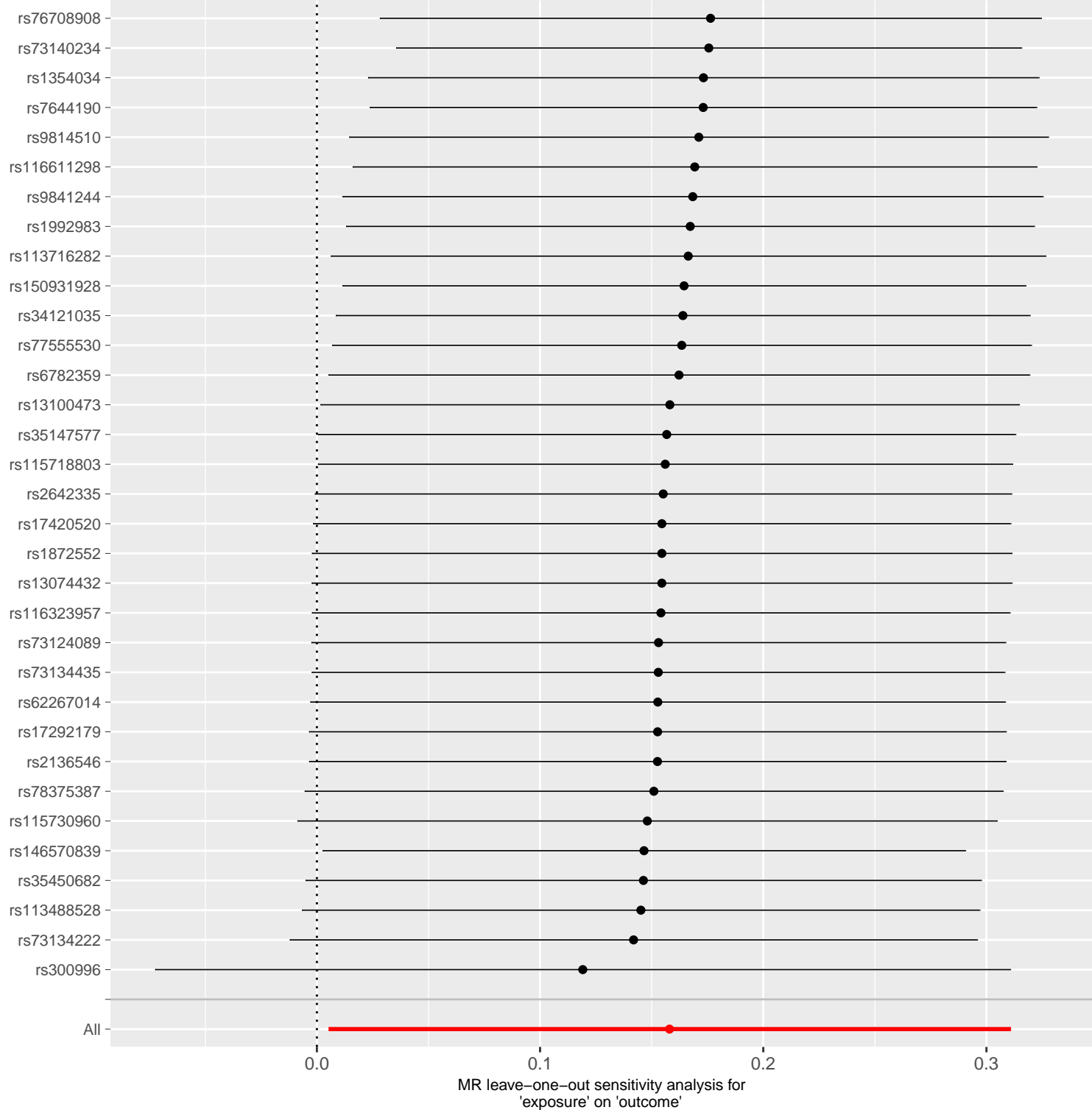

Supplement: Supplementary Data Sheet 1 — Harmonized summary data, forest plots, funnel plots, data sources, harmonization details, and sensitivity analyses for the Mendelian randomization analysis of pyroptosis-related proteins and ulcerative colitis. [file DataSheet1.zip › bdpqtlresult/17350_13_CHMP2B_CHM2B/sensitivity-analysis.pdf]

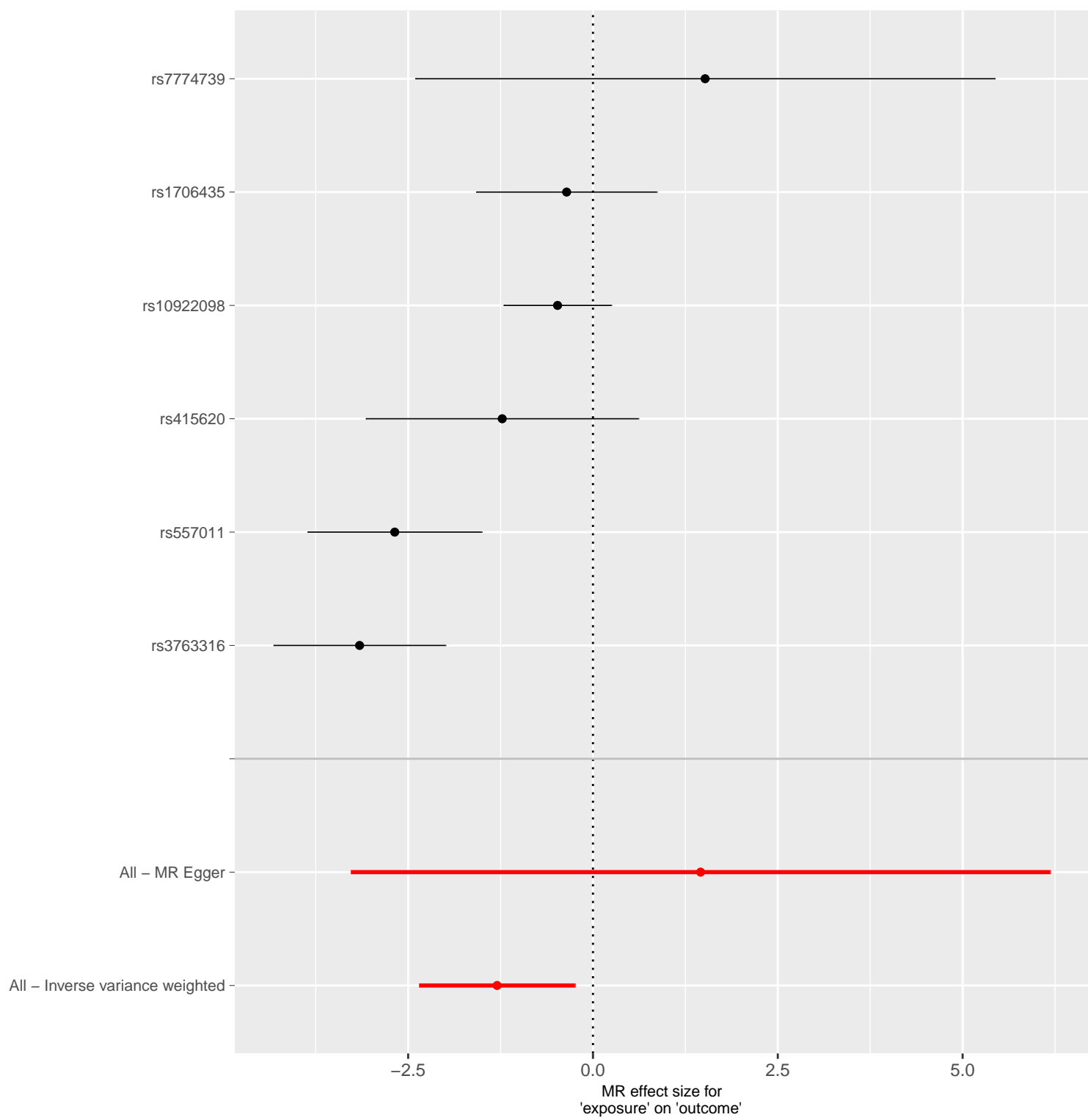

Supplement: Supplementary Data Sheet 1 — Harmonized summary data, forest plots, funnel plots, data sources, harmonization details, and sensitivity analyses for the Mendelian randomization analysis of pyroptosis-related proteins and ulcerative colitis. [file DataSheet1.zip › bdpqtlresult/17850_42_KLF4_KLF4/forest.pdf]

# MR Method

- Inverse variance weighted
- MR Egger

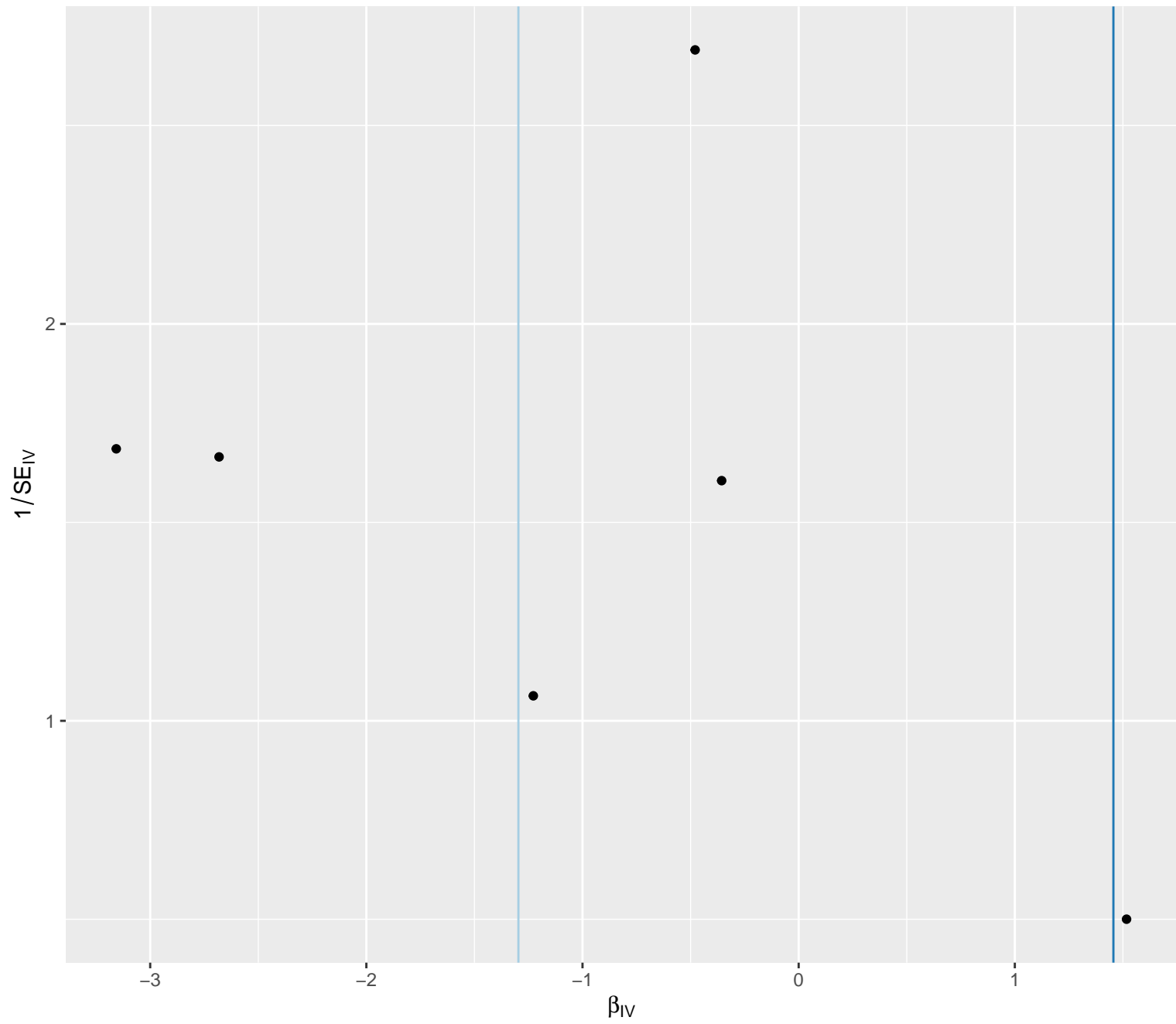

Supplement: Supplementary Data Sheet 1 — Harmonized summary data, forest plots, funnel plots, data sources, harmonization details, and sensitivity analyses for the Mendelian randomization analysis of pyroptosis-related proteins and ulcerative colitis. [file DataSheet1.zip › bdpqtlresult/17850_42_KLF4_KLF4/funnelplot.pdf]

# MR Test

- Inverse variance weighted
- MR Egger
- Simple mode
- Weighted median
- Weighted mode

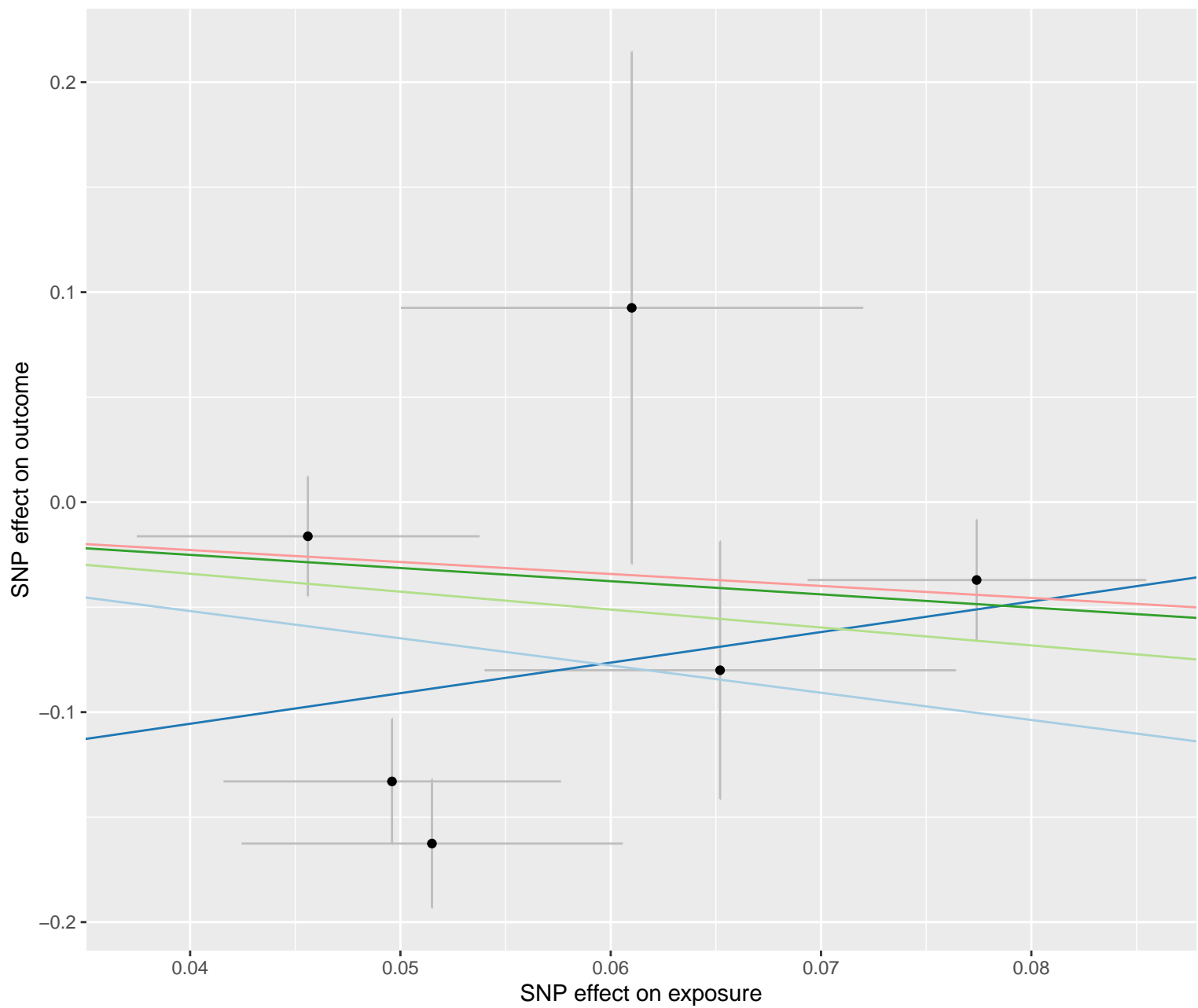

Supplement: Supplementary Data Sheet 1 — Harmonized summary data, forest plots, funnel plots, data sources, harmonization details, and sensitivity analyses for the Mendelian randomization analysis of pyroptosis-related proteins and ulcerative colitis. [file DataSheet1.zip › bdpqtlresult/17850_42_KLF4_KLF4/scatter.pdf]

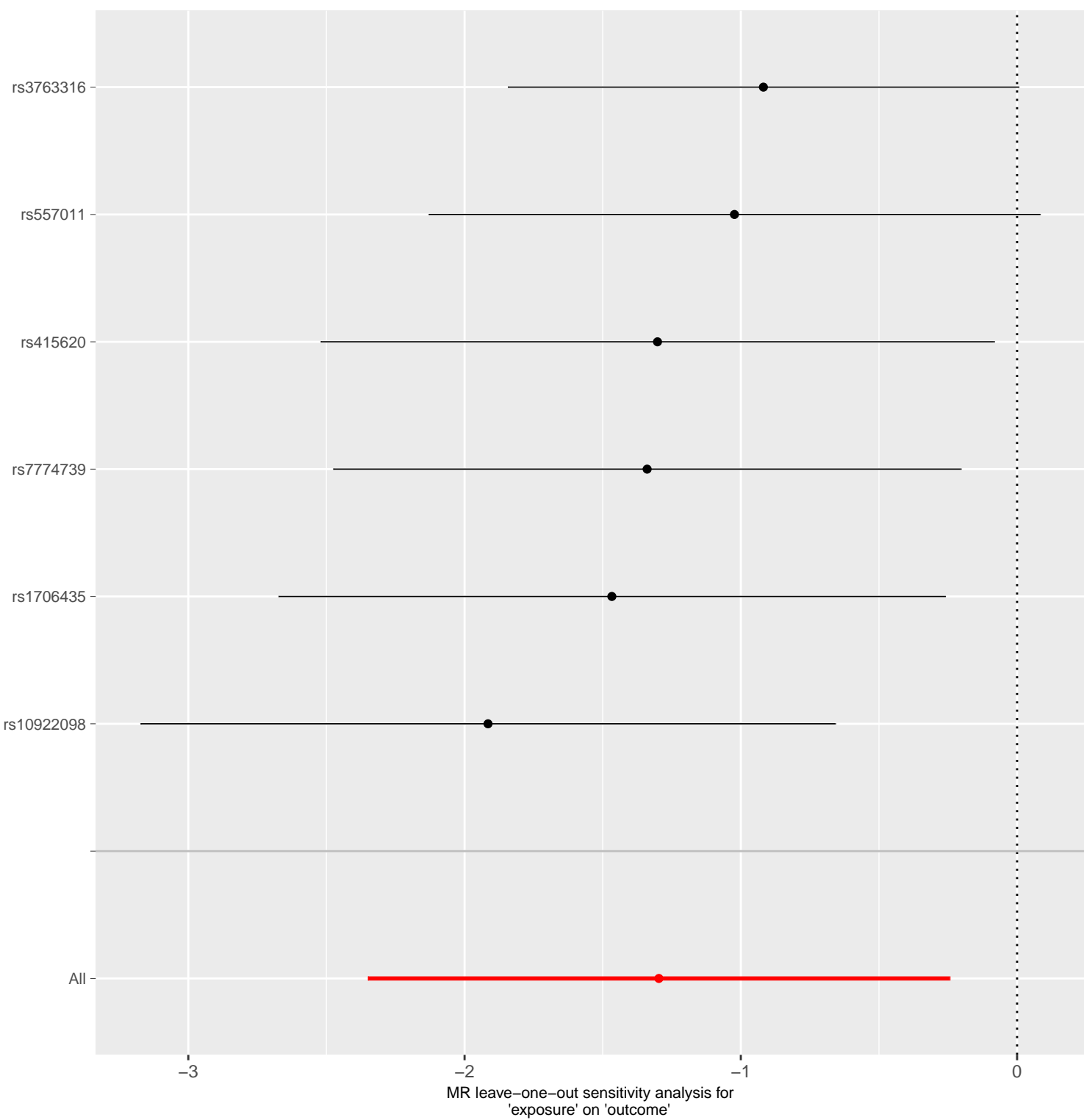

Supplement: Supplementary Data Sheet 1 — Harmonized summary data, forest plots, funnel plots, data sources, harmonization details, and sensitivity analyses for the Mendelian randomization analysis of pyroptosis-related proteins and ulcerative colitis. [file DataSheet1.zip › bdpqtlresult/17850_42_KLF4_KLF4/sensitivity-analysis.pdf]

All – Inverse variance weighted

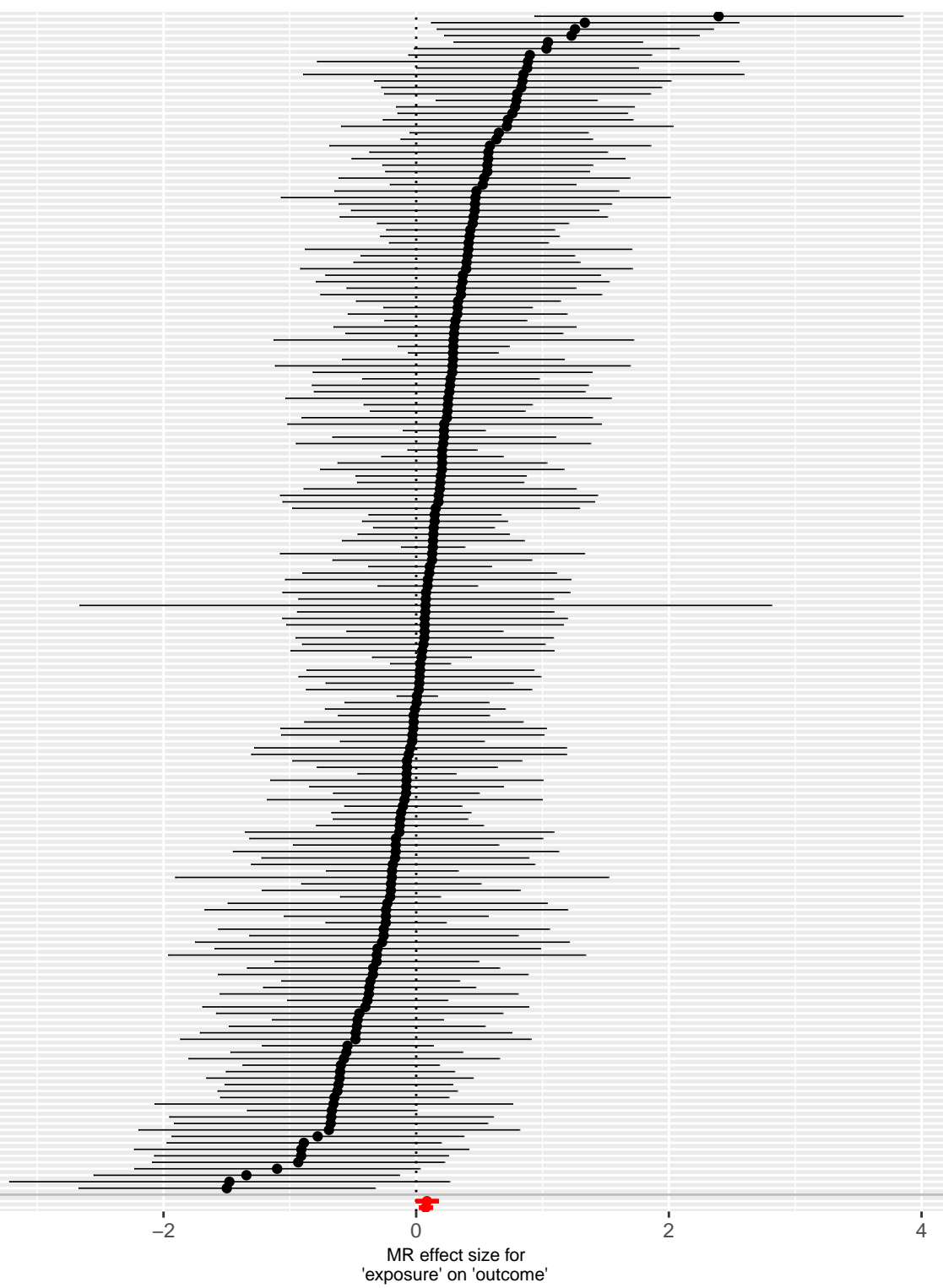

Supplement: Supplementary Data Sheet 1 — Harmonized summary data, forest plots, funnel plots, data sources, harmonization details, and sensitivity analyses for the Mendelian randomization analysis of pyroptosis-related proteins and ulcerative colitis. [file DataSheet1.zip › bdpqtlresult/18819_21_PPIC_PPIC/forest.pdf]

# MR Method

Inverse variance weighted

MR Egger

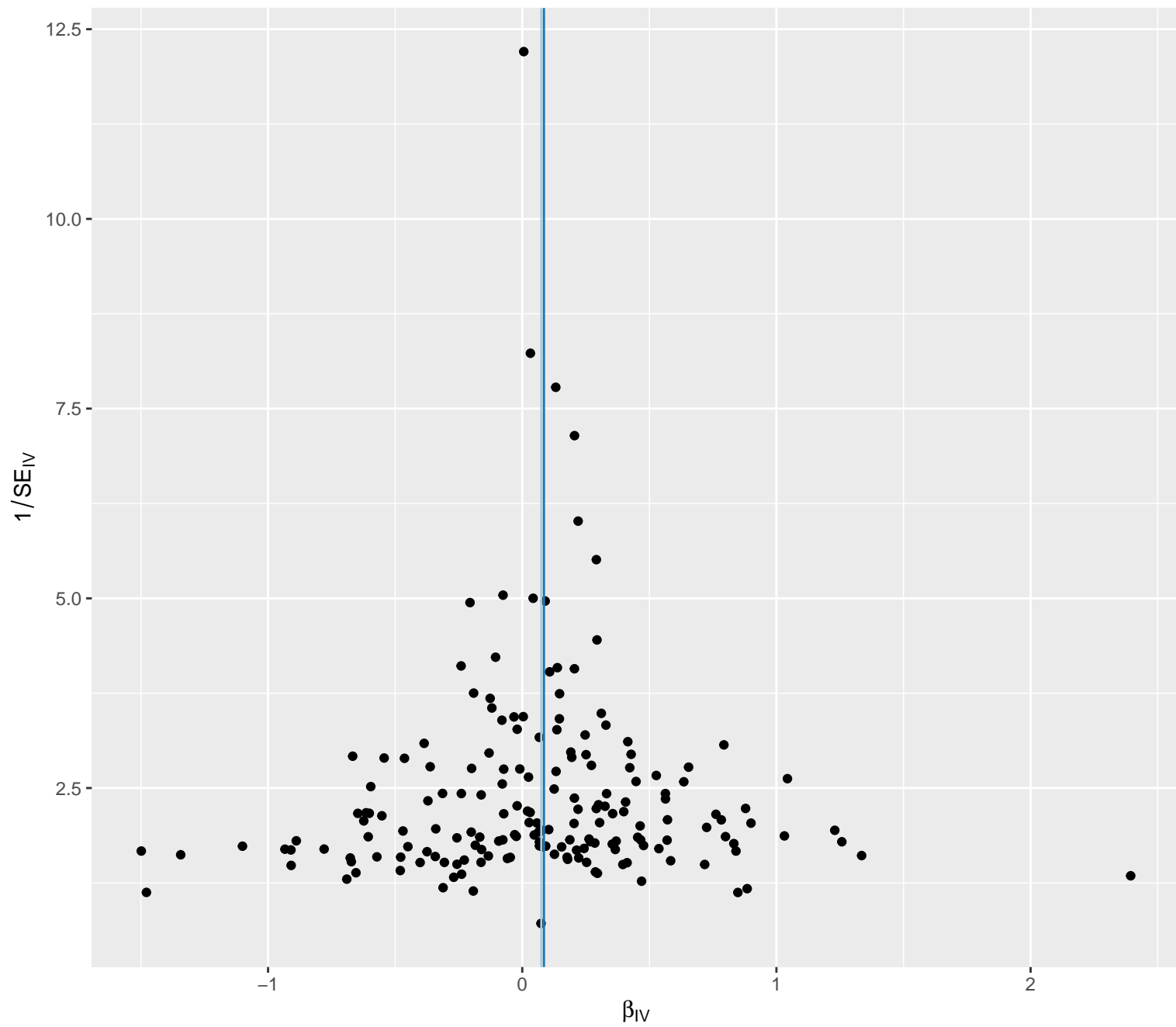

Supplement: Supplementary Data Sheet 1 — Harmonized summary data, forest plots, funnel plots, data sources, harmonization details, and sensitivity analyses for the Mendelian randomization analysis of pyroptosis-related proteins and ulcerative colitis. [file DataSheet1.zip › bdpqtlresult/18819_21_PPIC_PPIC/funnelplot.pdf]

# MR Test

- Inverse variance weighted
- MR Egger
- Simple mode
- Weighted median
- Weighted mode

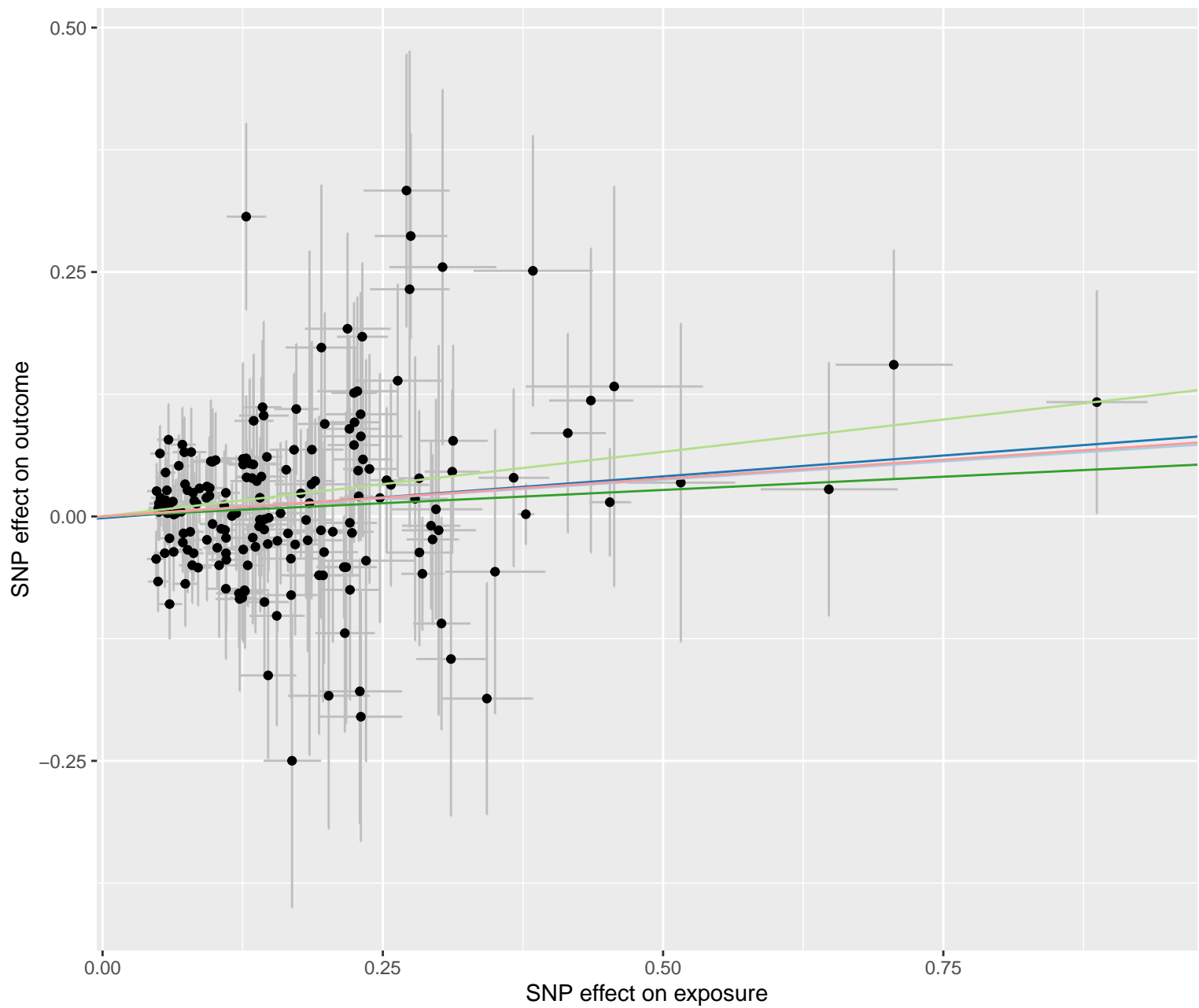

Supplement: Supplementary Data Sheet 1 — Harmonized summary data, forest plots, funnel plots, data sources, harmonization details, and sensitivity analyses for the Mendelian randomization analysis of pyroptosis-related proteins and ulcerative colitis. [file DataSheet1.zip › bdpqtlresult/18819_21_PPIC_PPIC/scatter.pdf]

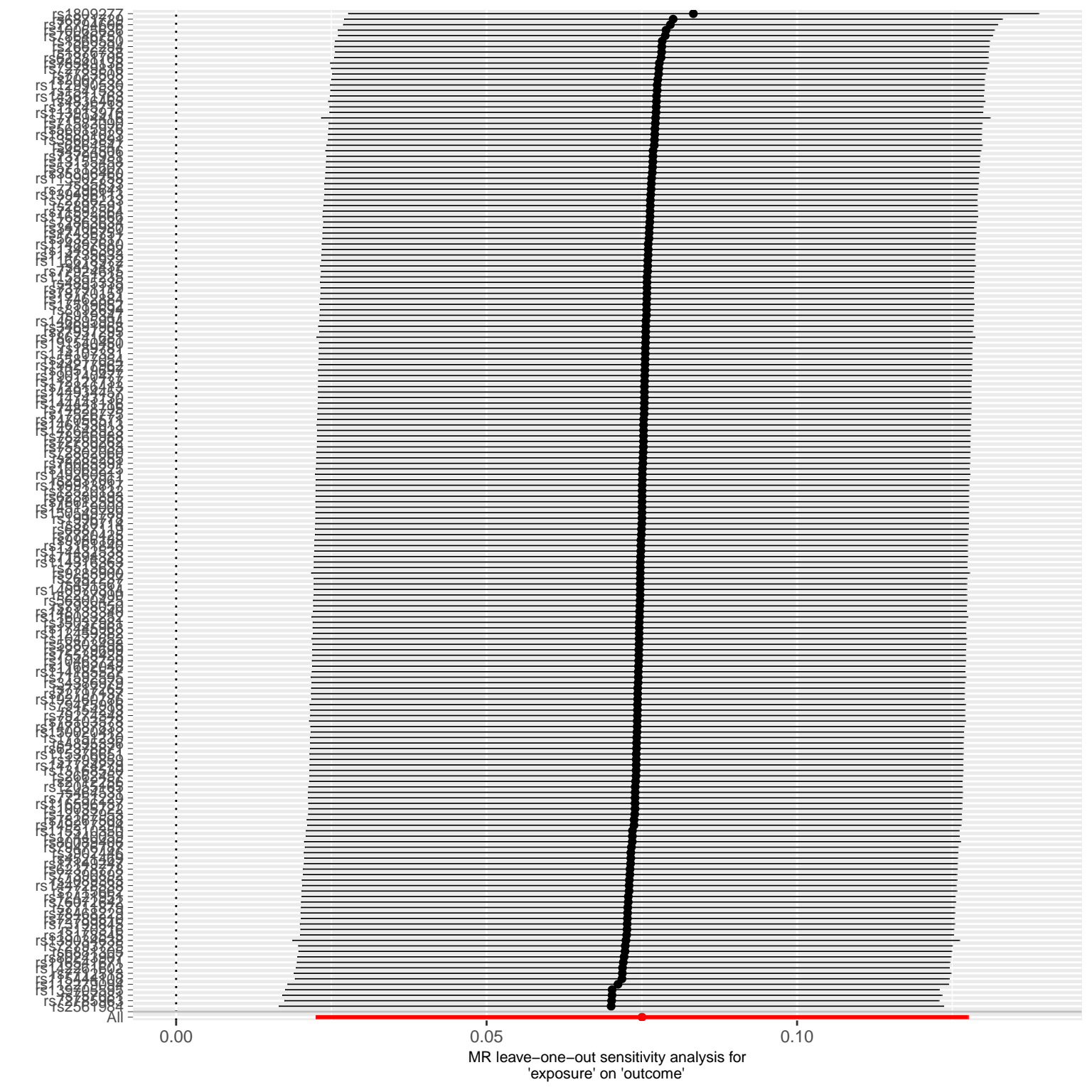

Supplement: Supplementary Data Sheet 1 — Harmonized summary data, forest plots, funnel plots, data sources, harmonization details, and sensitivity analyses for the Mendelian randomization analysis of pyroptosis-related proteins and ulcerative colitis. [file DataSheet1.zip › bdpqtlresult/18819_21_PPIC_PPIC/sensitivity-analysis.pdf]

# MR Method

- Inverse variance weighted
- MR Egger

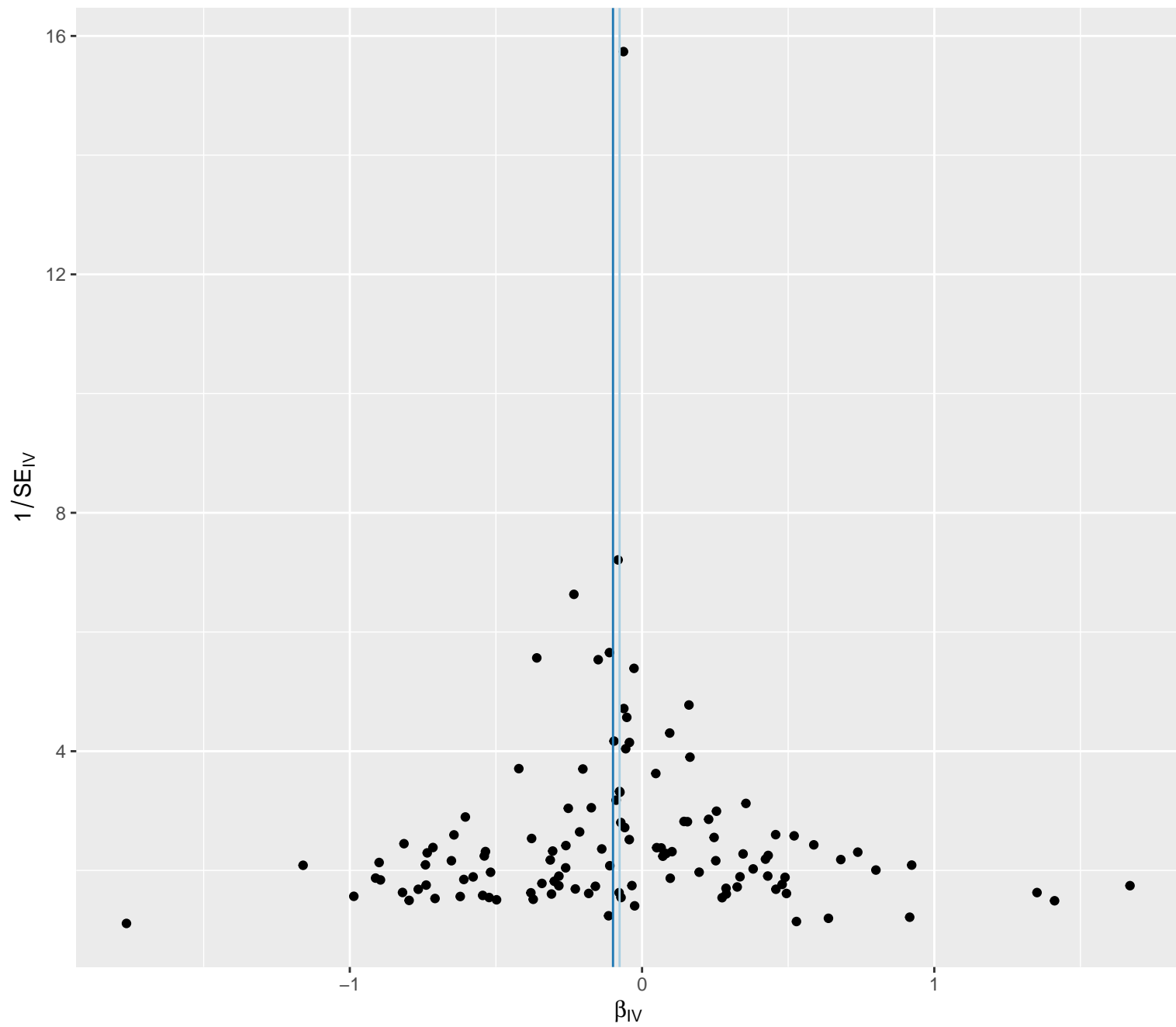

Supplement: Supplementary Data Sheet 1 — Harmonized summary data, forest plots, funnel plots, data sources, harmonization details, and sensitivity analyses for the Mendelian randomization analysis of pyroptosis-related proteins and ulcerative colitis. [file DataSheet1.zip › bdpqtlresult/19437_61_VEGFA_L_VEGF165/funnelplot.pdf]

# MR Test

- Inverse variance weighted
- MR Egger
- Simple mode
- Weighted median
- Weighted mode

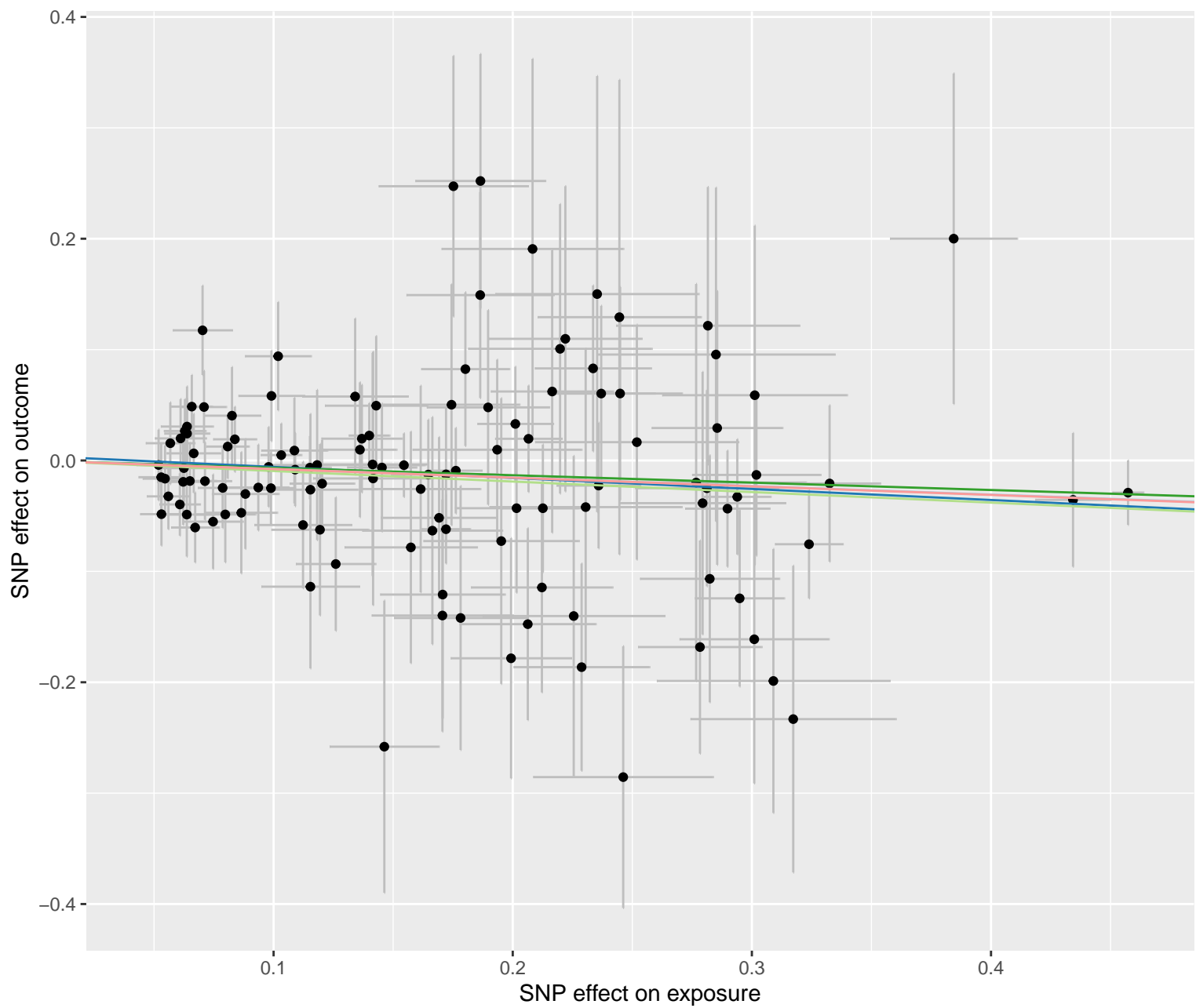

Supplement: Supplementary Data Sheet 1 — Harmonized summary data, forest plots, funnel plots, data sources, harmonization details, and sensitivity analyses for the Mendelian randomization analysis of pyroptosis-related proteins and ulcerative colitis. [file DataSheet1.zip › bdpqtlresult/19437_61_VEGFA_L_VEGF165/scatter.pdf]

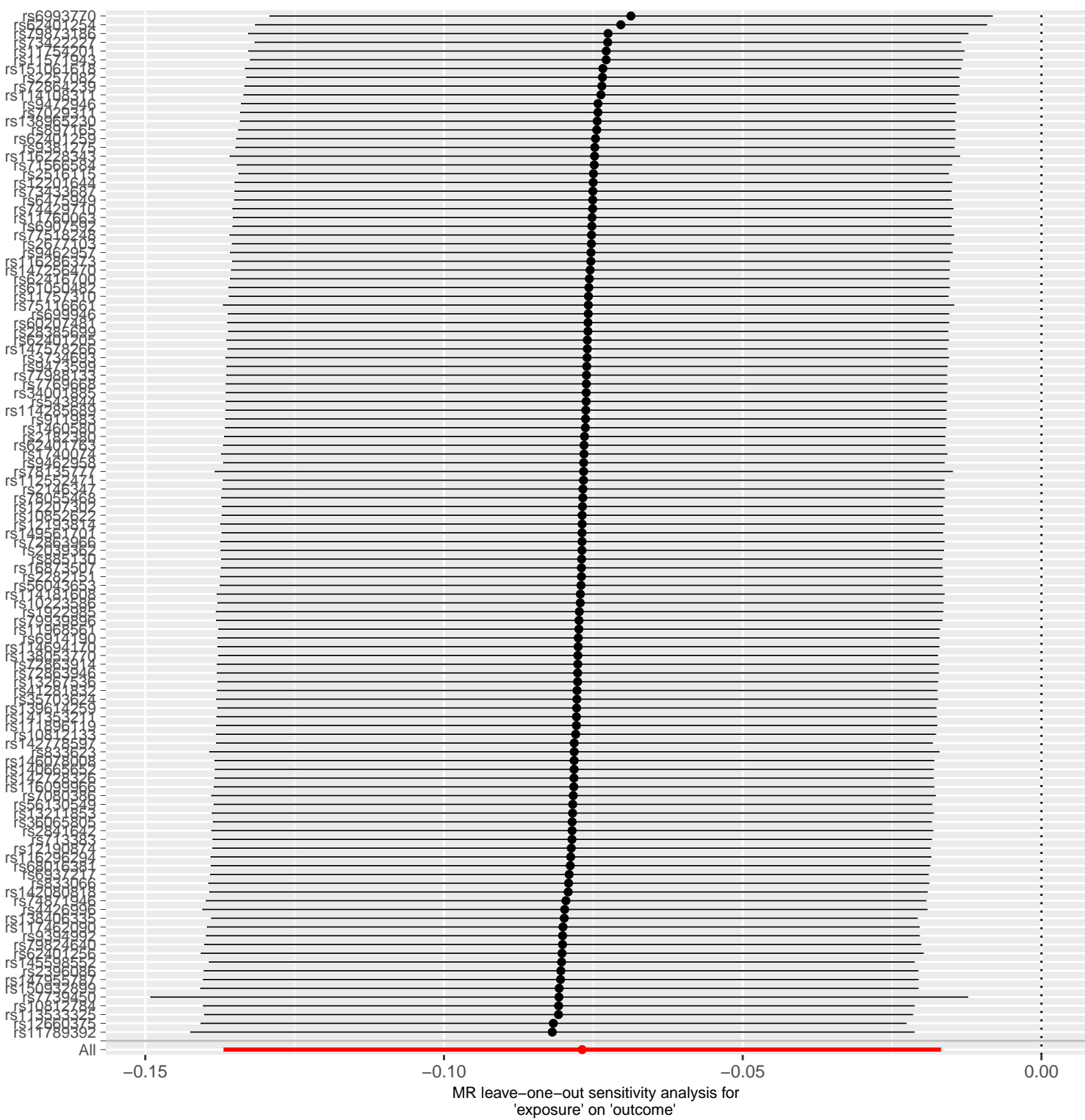

Supplement: Supplementary Data Sheet 1 — Harmonized summary data, forest plots, funnel plots, data sources, harmonization details, and sensitivity analyses for the Mendelian randomization analysis of pyroptosis-related proteins and ulcerative colitis. [file DataSheet1.zip › bdpqtlresult/19437_61_VEGFA_L_VEGF165/sensitivity-analysis.pdf]

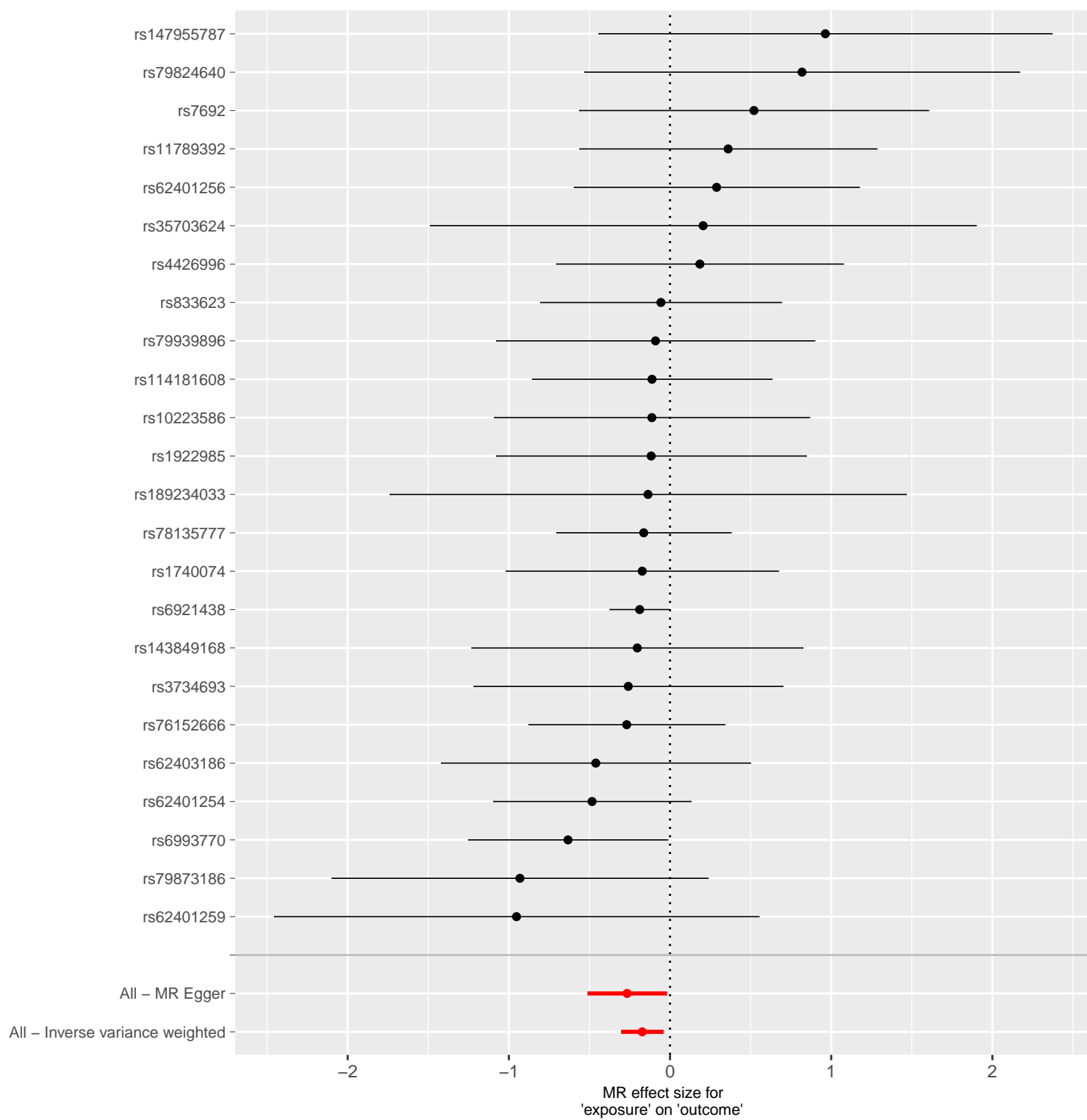

Supplement: Supplementary Data Sheet 1 — Harmonized summary data, forest plots, funnel plots, data sources, harmonization details, and sensitivity analyses for the Mendelian randomization analysis of pyroptosis-related proteins and ulcerative colitis. [file DataSheet1.zip › bdpqtlresult/2597_8_VEGFA_VEGF/forest.pdf]

# MR Method

- Inverse variance weighted
- MR Egger

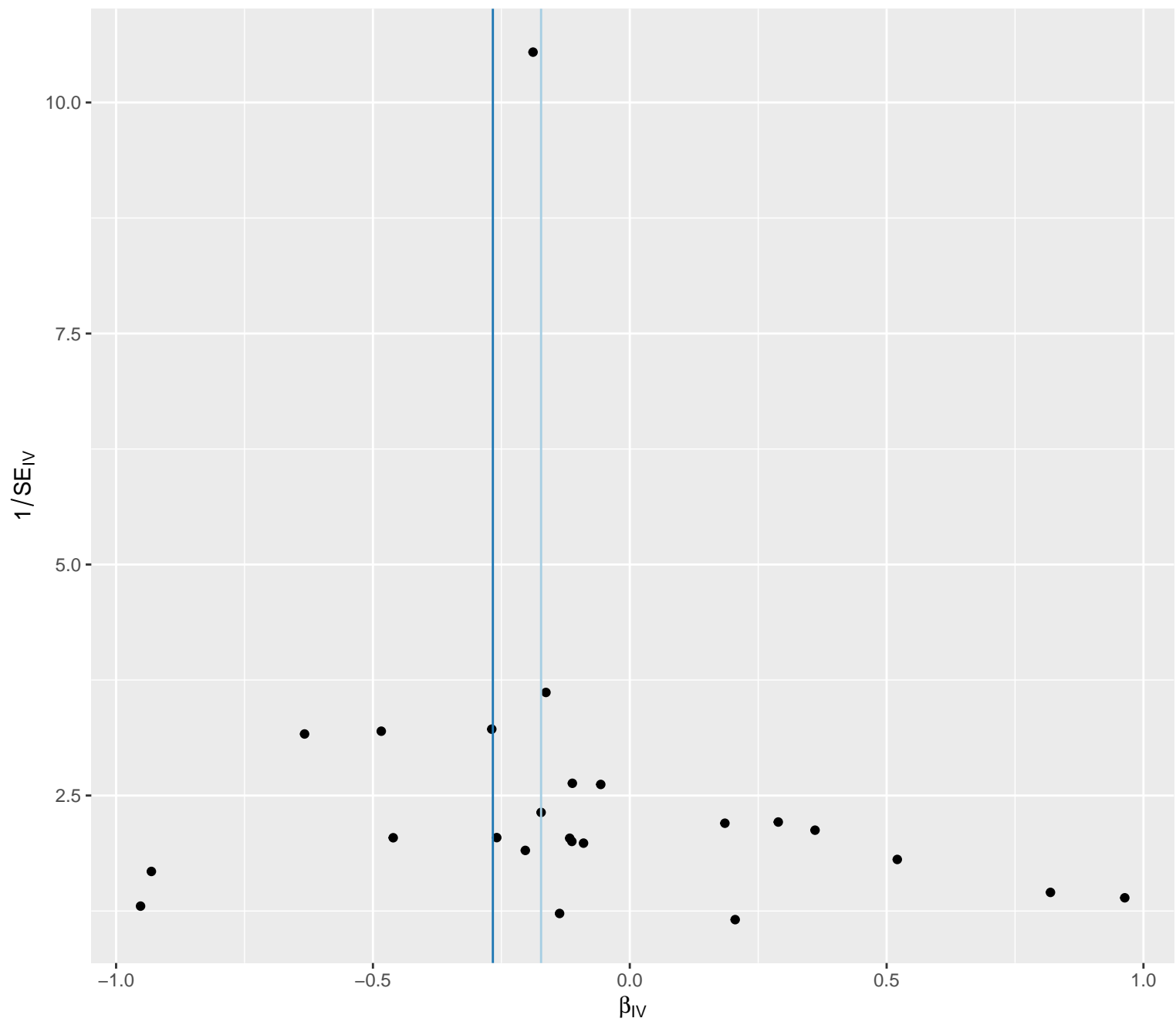

Supplement: Supplementary Data Sheet 1 — Harmonized summary data, forest plots, funnel plots, data sources, harmonization details, and sensitivity analyses for the Mendelian randomization analysis of pyroptosis-related proteins and ulcerative colitis. [file DataSheet1.zip › bdpqtlresult/2597_8_VEGFA_VEGF/funnelplot.pdf]

# MR Test

- Inverse variance weighted
- MR Egger
- Simple mode
- Weighted median
- Weighted mode

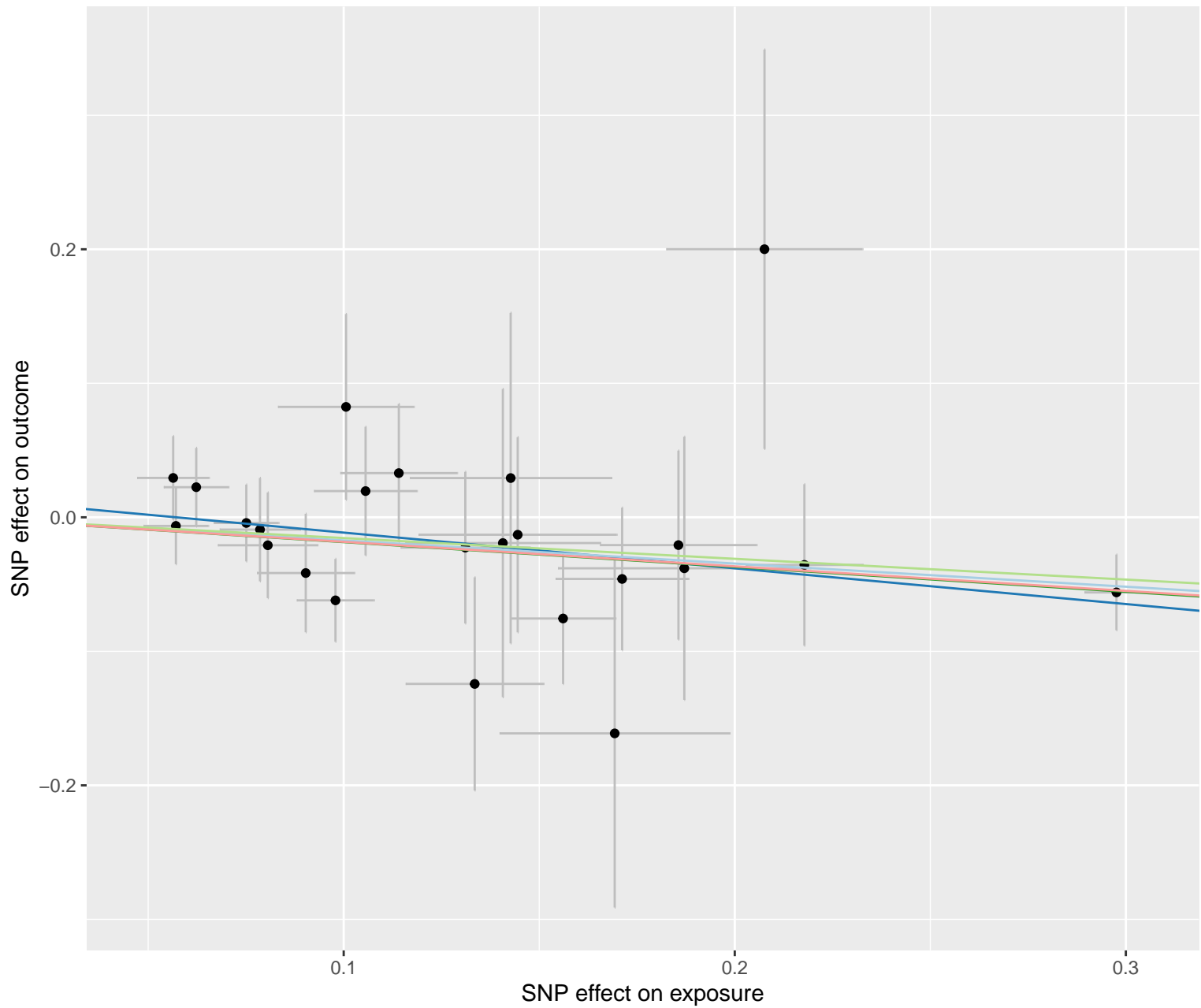

Supplement: Supplementary Data Sheet 1 — Harmonized summary data, forest plots, funnel plots, data sources, harmonization details, and sensitivity analyses for the Mendelian randomization analysis of pyroptosis-related proteins and ulcerative colitis. [file DataSheet1.zip › bdpqtlresult/2597_8_VEGFA_VEGF/scatter.pdf]

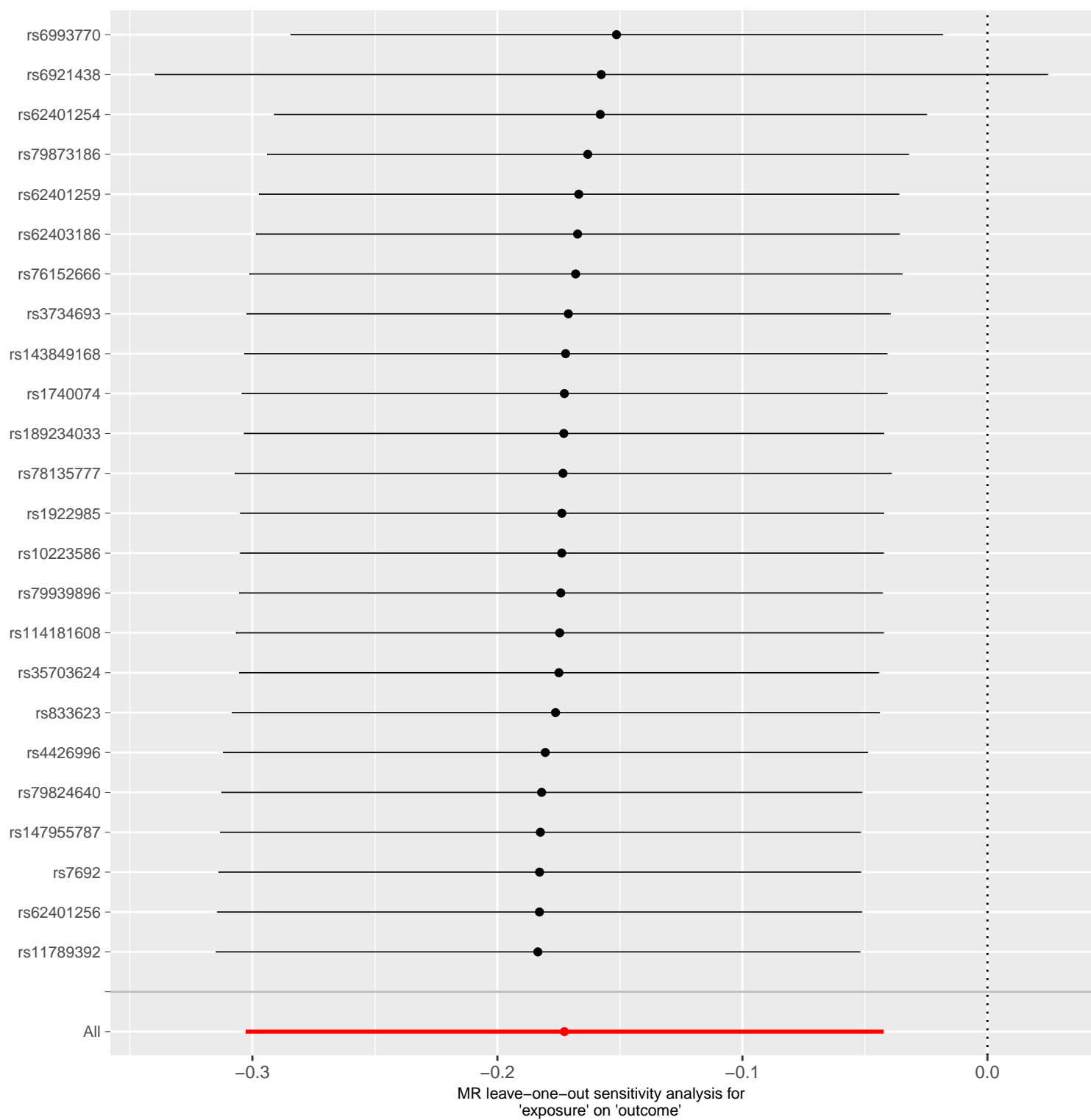

Supplement: Supplementary Data Sheet 1 — Harmonized summary data, forest plots, funnel plots, data sources, harmonization details, and sensitivity analyses for the Mendelian randomization analysis of pyroptosis-related proteins and ulcerative colitis. [file DataSheet1.zip › bdpqtlresult/2597_8_VEGFA_VEGF/sensitivity-analysis.pdf]

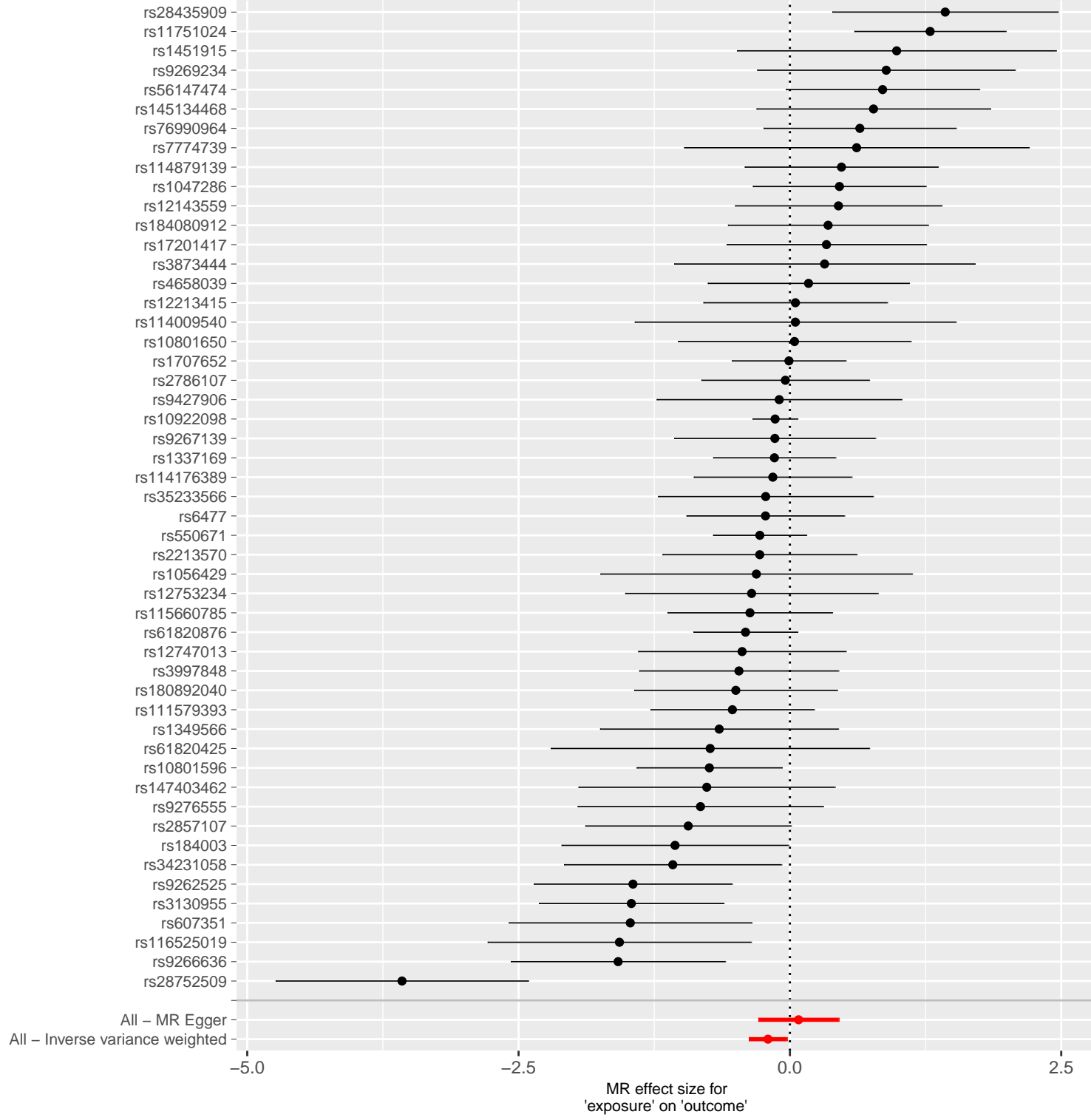

Supplement: Supplementary Data Sheet 1 — Harmonized summary data, forest plots, funnel plots, data sources, harmonization details, and sensitivity analyses for the Mendelian randomization analysis of pyroptosis-related proteins and ulcerative colitis. [file DataSheet1.zip › bdpqtlresult/2693_20_OSM_OSM/forest.pdf]

# MR Method

- Inverse variance weighted
- MR Egger

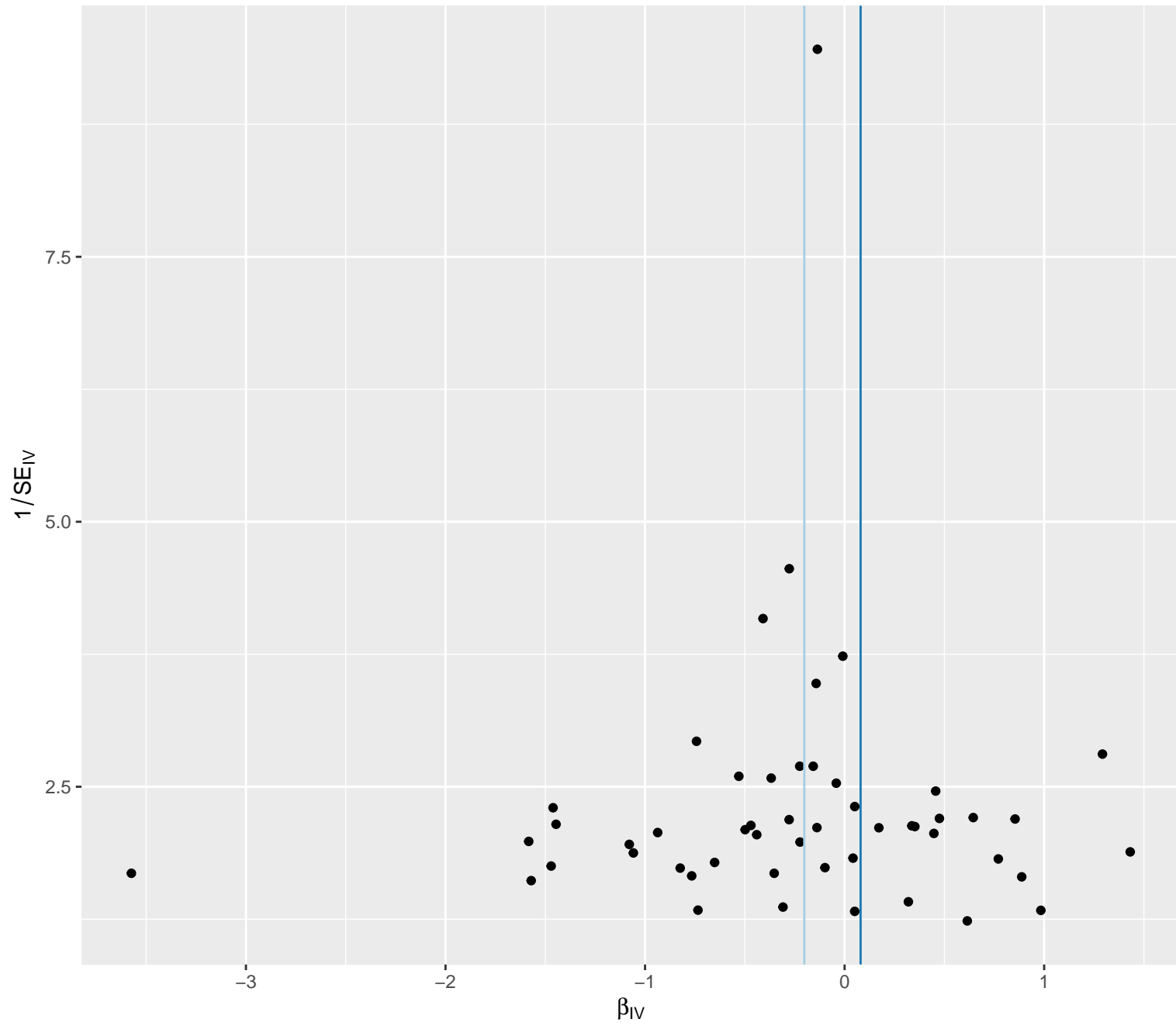

Supplement: Supplementary Data Sheet 1 — Harmonized summary data, forest plots, funnel plots, data sources, harmonization details, and sensitivity analyses for the Mendelian randomization analysis of pyroptosis-related proteins and ulcerative colitis. [file DataSheet1.zip › bdpqtlresult/2693_20_OSM_OSM/funnelplot.pdf]

# MR Test

- Inverse variance weighted
- MR Egger
- Simple mode
- Weighted median
- Weighted mode

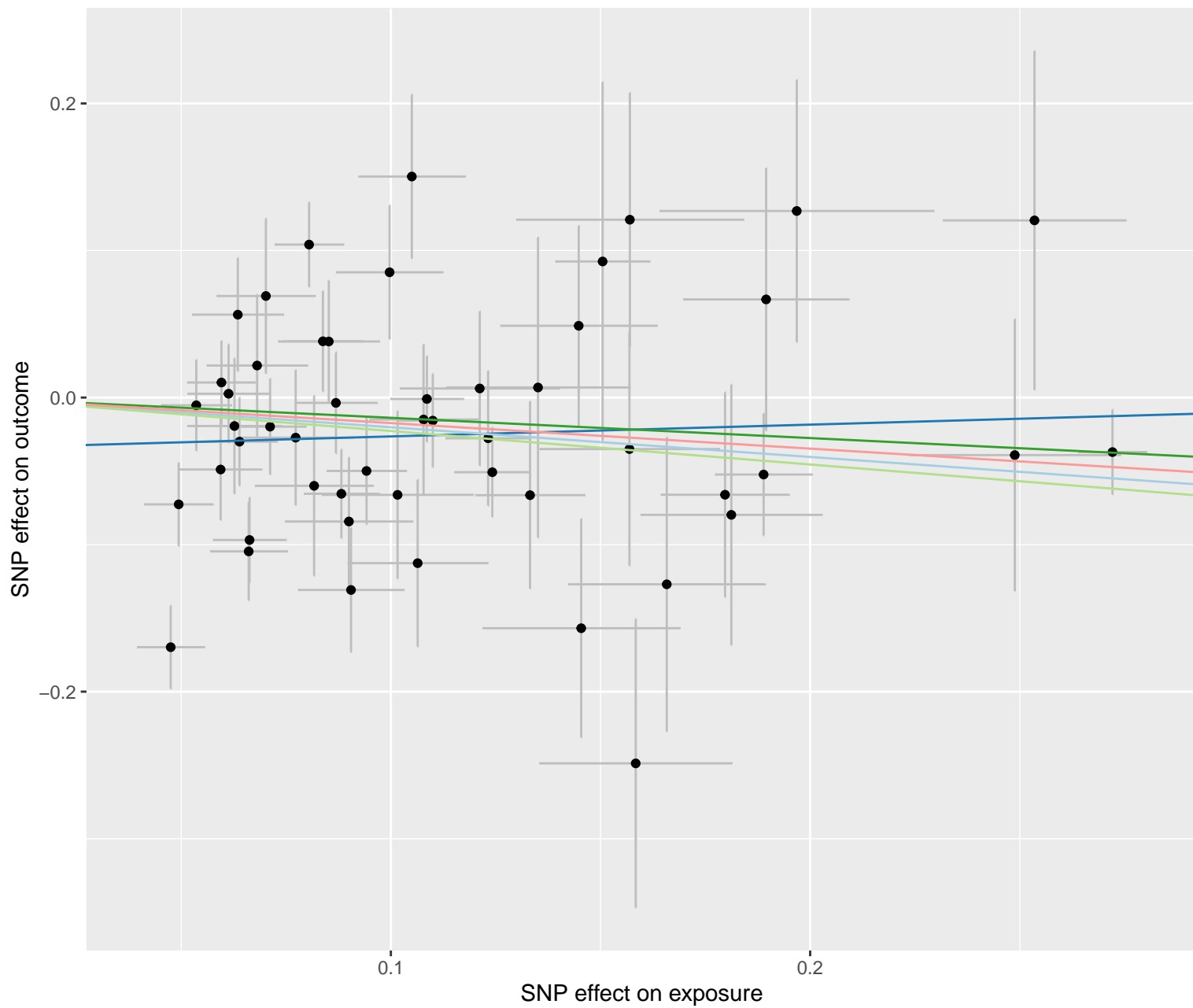

Supplement: Supplementary Data Sheet 1 — Harmonized summary data, forest plots, funnel plots, data sources, harmonization details, and sensitivity analyses for the Mendelian randomization analysis of pyroptosis-related proteins and ulcerative colitis. [file DataSheet1.zip › bdpqtlresult/2693_20_OSM_OSM/scatter.pdf]

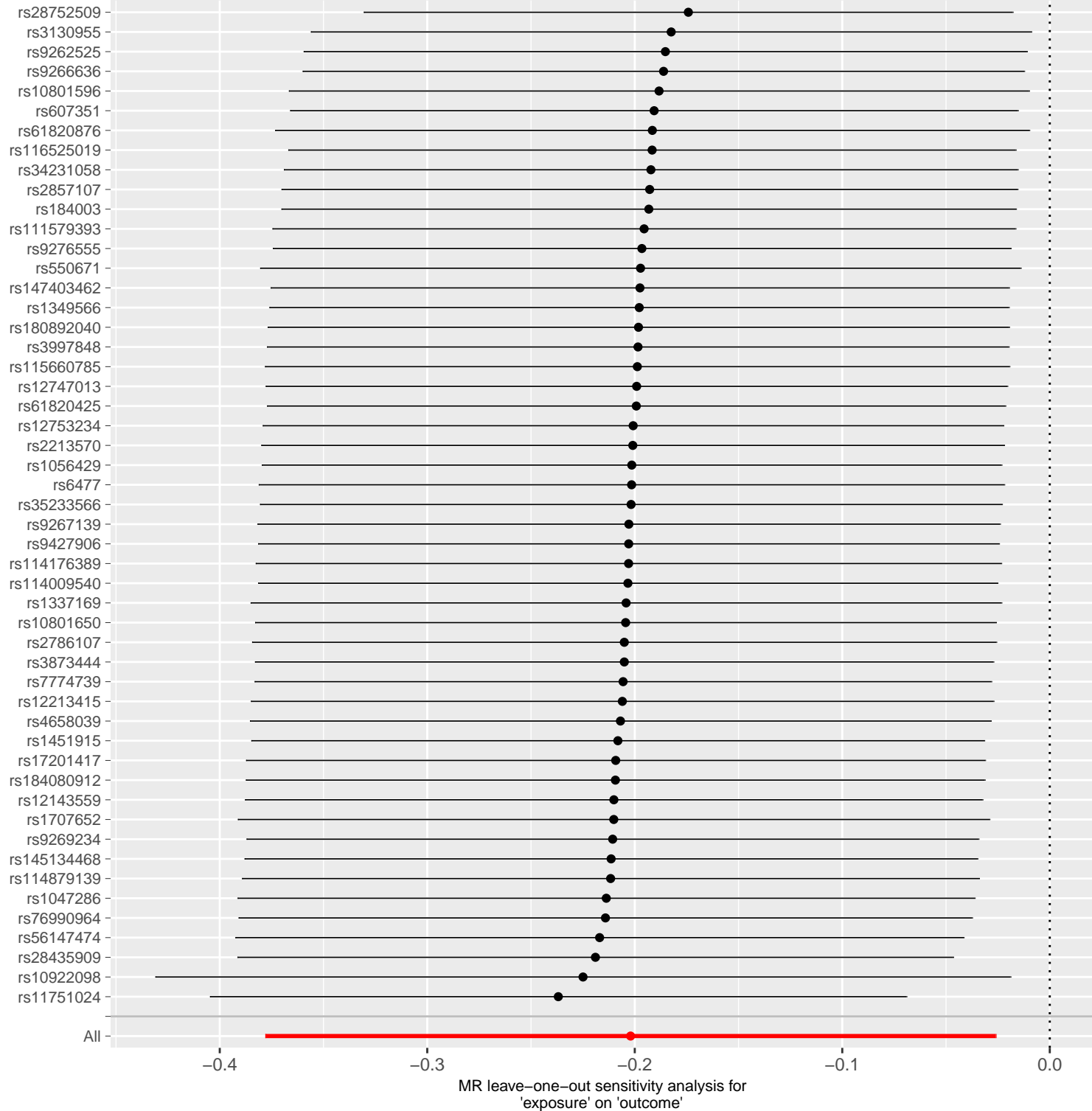

Supplement: Supplementary Data Sheet 1 — Harmonized summary data, forest plots, funnel plots, data sources, harmonization details, and sensitivity analyses for the Mendelian randomization analysis of pyroptosis-related proteins and ulcerative colitis. [file DataSheet1.zip › bdpqtlresult/2693_20_OSM_OSM/sensitivity-analysis.pdf]

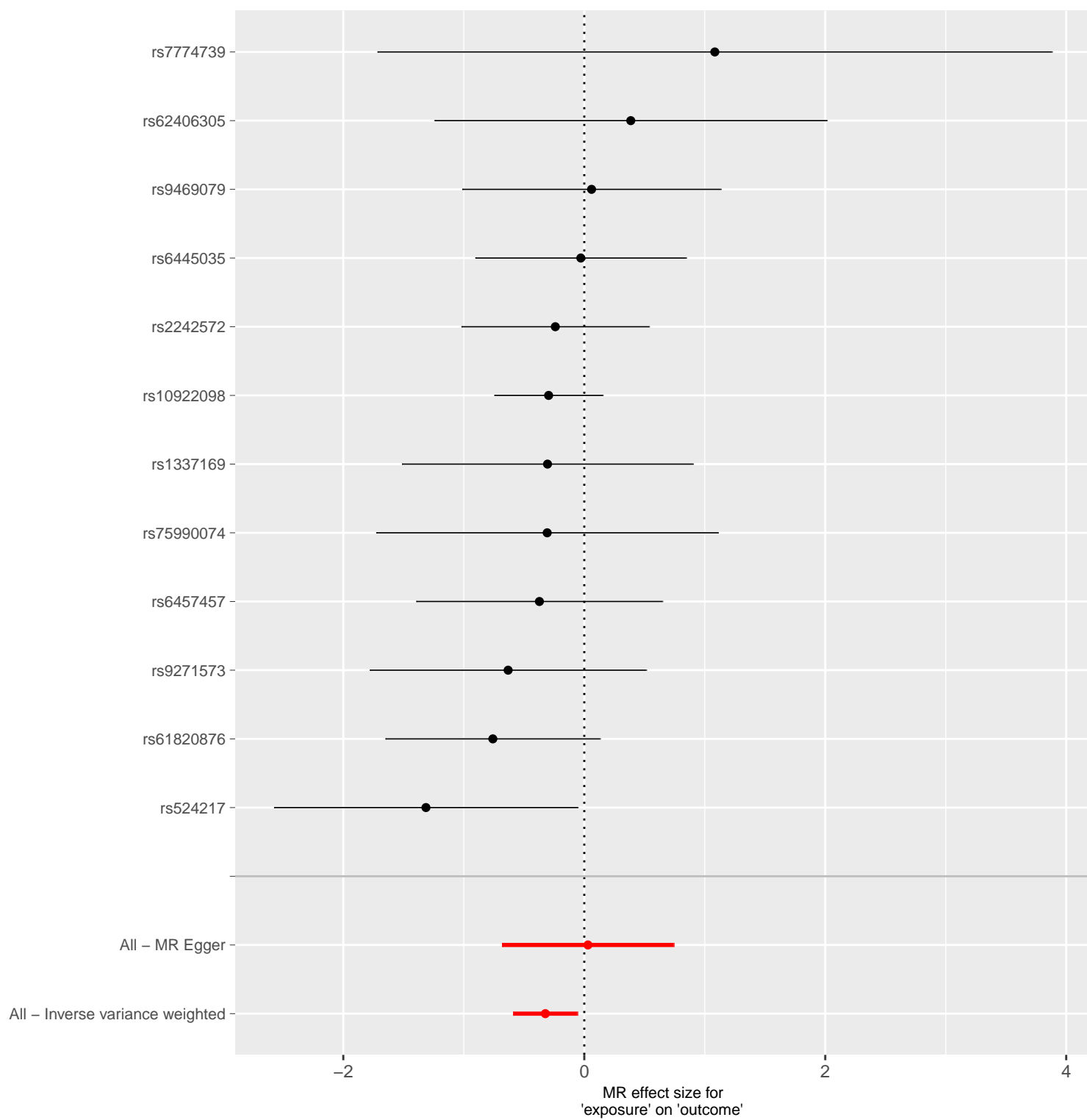

Supplement: Supplementary Data Sheet 1 — Harmonized summary data, forest plots, funnel plots, data sources, harmonization details, and sensitivity analyses for the Mendelian randomization analysis of pyroptosis-related proteins and ulcerative colitis. [file DataSheet1.zip › bdpqtlresult/3072_4_IL13_IL_13/forest.pdf]

# MR Method

- Inverse variance weighted
- MR Egger

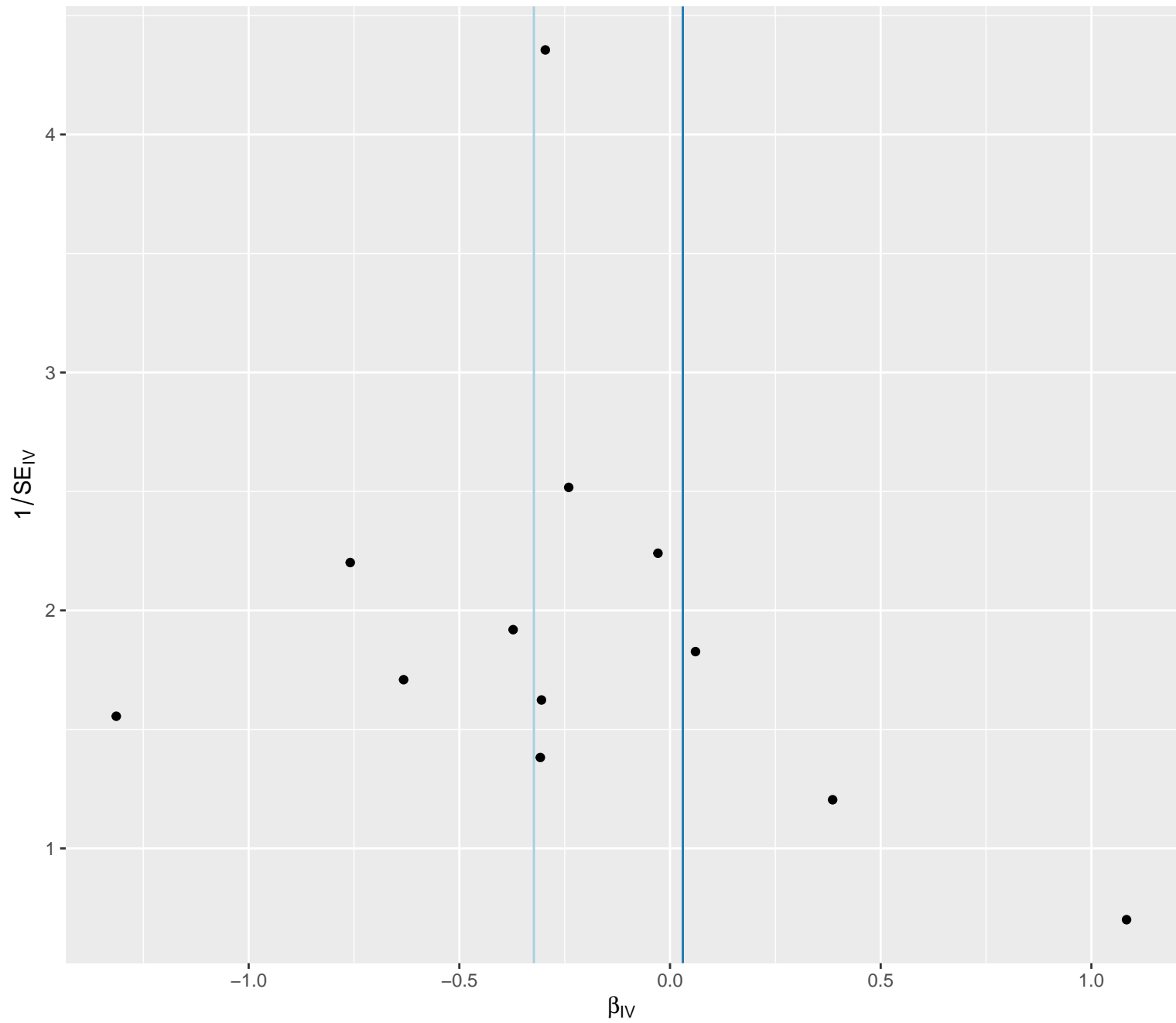

Supplement: Supplementary Data Sheet 1 — Harmonized summary data, forest plots, funnel plots, data sources, harmonization details, and sensitivity analyses for the Mendelian randomization analysis of pyroptosis-related proteins and ulcerative colitis. [file DataSheet1.zip › bdpqtlresult/3072_4_IL13_IL_13/funnelplot.pdf]

# MR Test

- Inverse variance weighted
- MR Egger
- Simple mode
- Weighted median
- Weighted mode

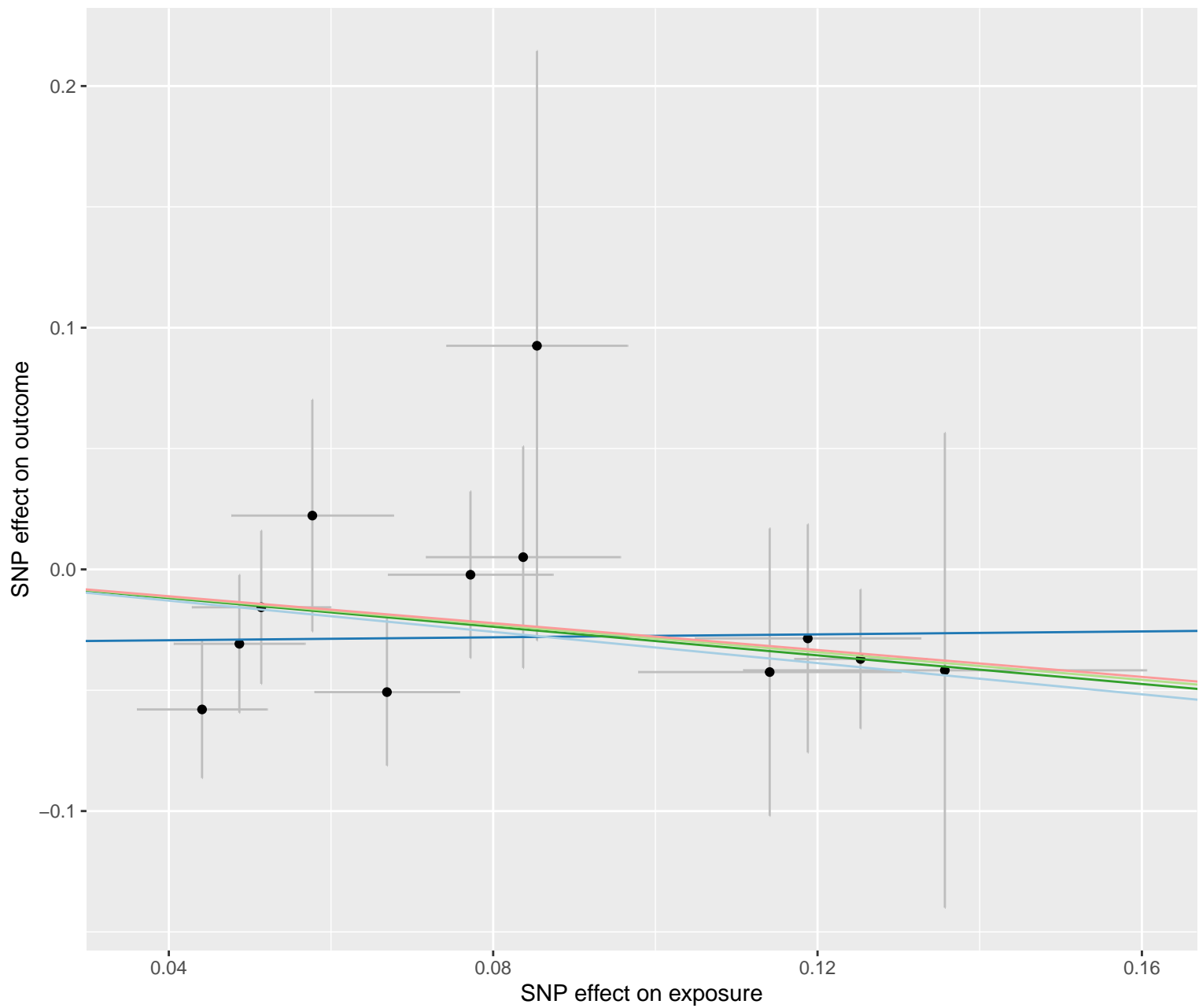

Supplement: Supplementary Data Sheet 1 — Harmonized summary data, forest plots, funnel plots, data sources, harmonization details, and sensitivity analyses for the Mendelian randomization analysis of pyroptosis-related proteins and ulcerative colitis. [file DataSheet1.zip › bdpqtlresult/3072_4_IL13_IL_13/scatter.pdf]

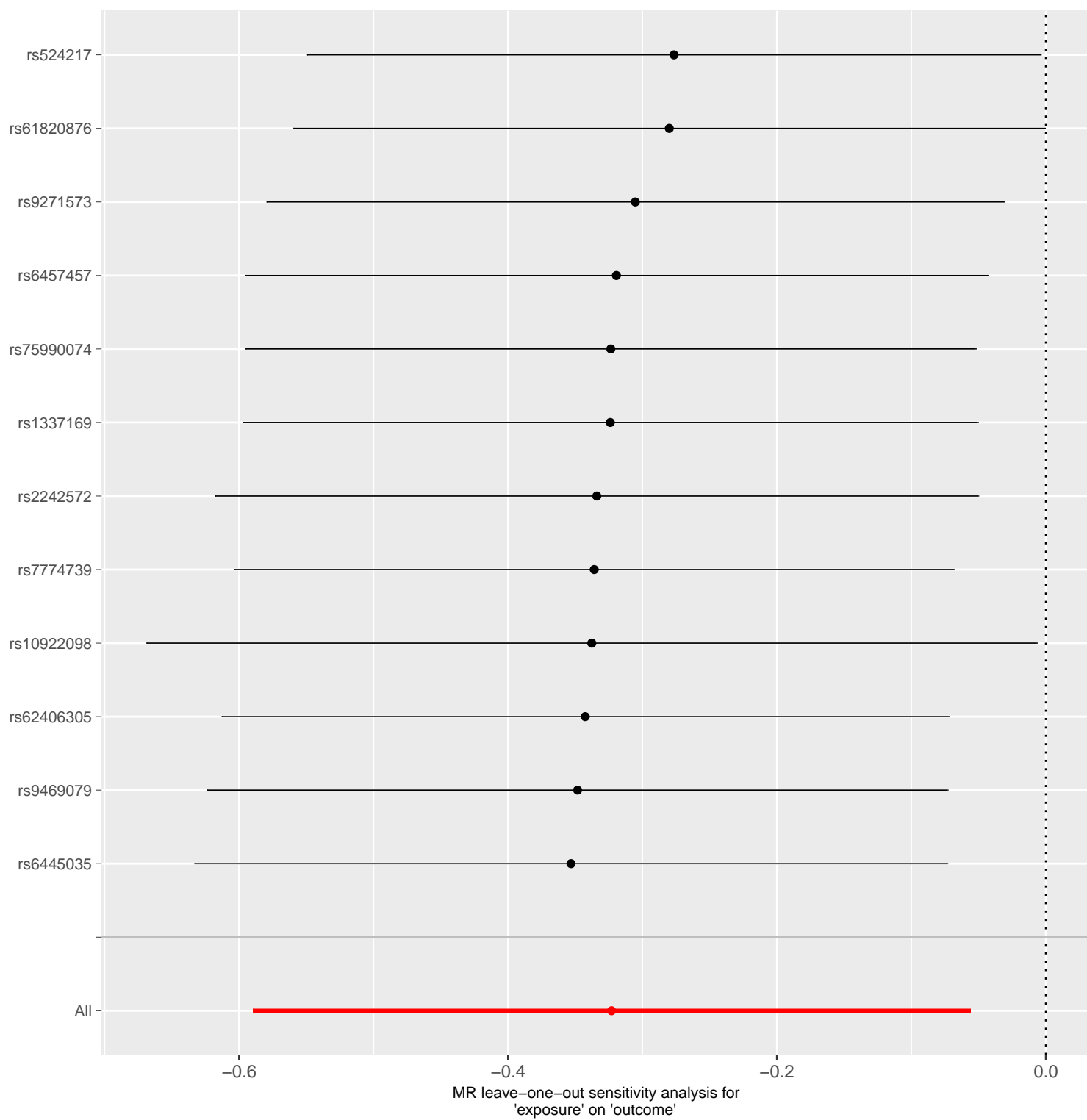

Supplement: Supplementary Data Sheet 1 — Harmonized summary data, forest plots, funnel plots, data sources, harmonization details, and sensitivity analyses for the Mendelian randomization analysis of pyroptosis-related proteins and ulcerative colitis. [file DataSheet1.zip › bdpqtlresult/3072_4_IL13_IL_13/sensitivity-analysis.pdf]

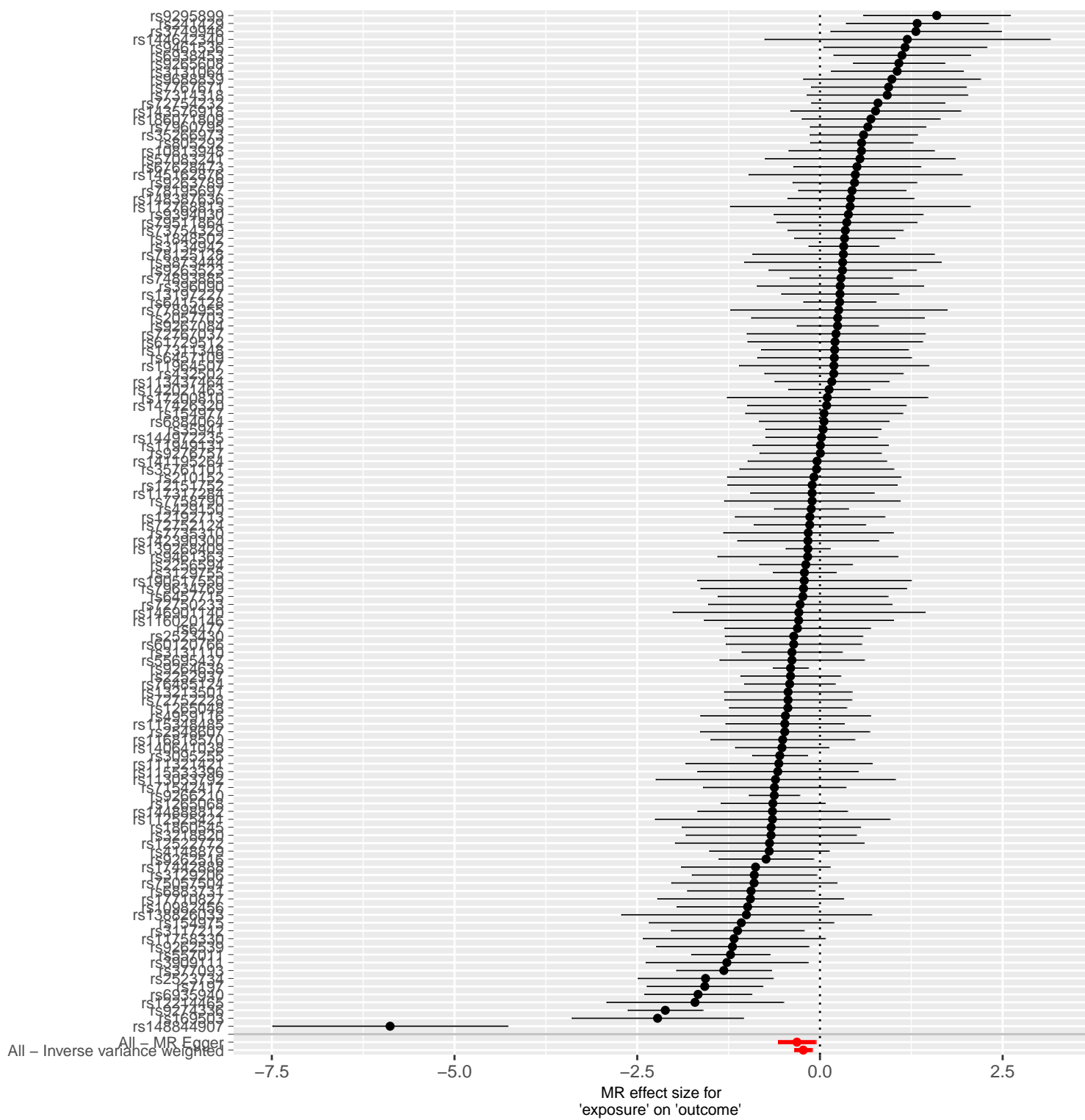

Supplement: Supplementary Data Sheet 1 — Harmonized summary data, forest plots, funnel plots, data sources, harmonization details, and sensitivity analyses for the Mendelian randomization analysis of pyroptosis-related proteins and ulcerative colitis. [file DataSheet1.zip › bdpqtlresult/3440_7_GZMA_granzyme_A/forest.pdf]

# MR Method

- Inverse variance weighted
- MR Egger

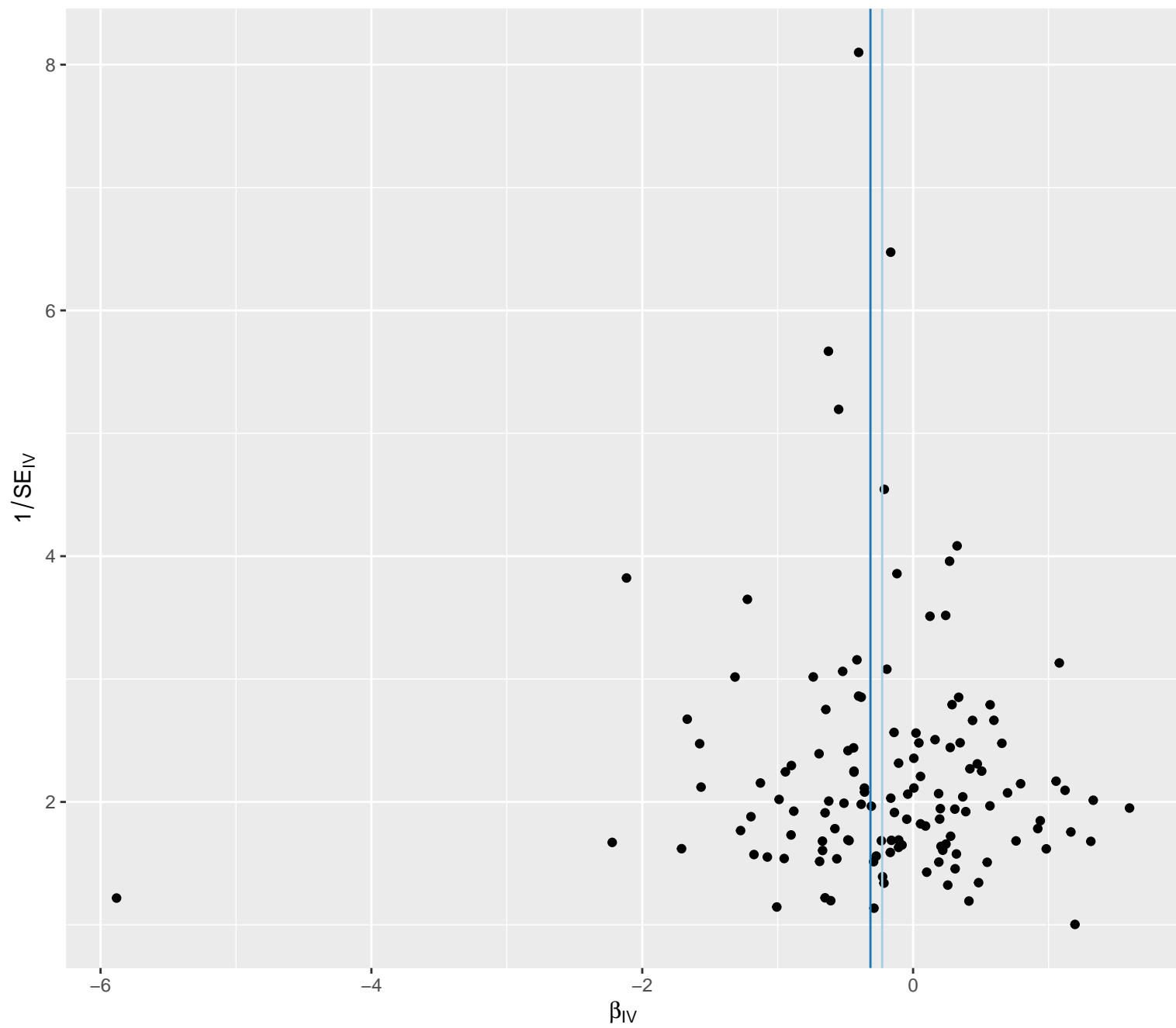

Supplement: Supplementary Data Sheet 1 — Harmonized summary data, forest plots, funnel plots, data sources, harmonization details, and sensitivity analyses for the Mendelian randomization analysis of pyroptosis-related proteins and ulcerative colitis. [file DataSheet1.zip › bdpqtlresult/3440_7_GZMA_granzyme_A/funnelplot.pdf]

# MR Test

- Inverse variance weighted
- MR Egger
- Simple mode
- Weighted median
- Weighted mode

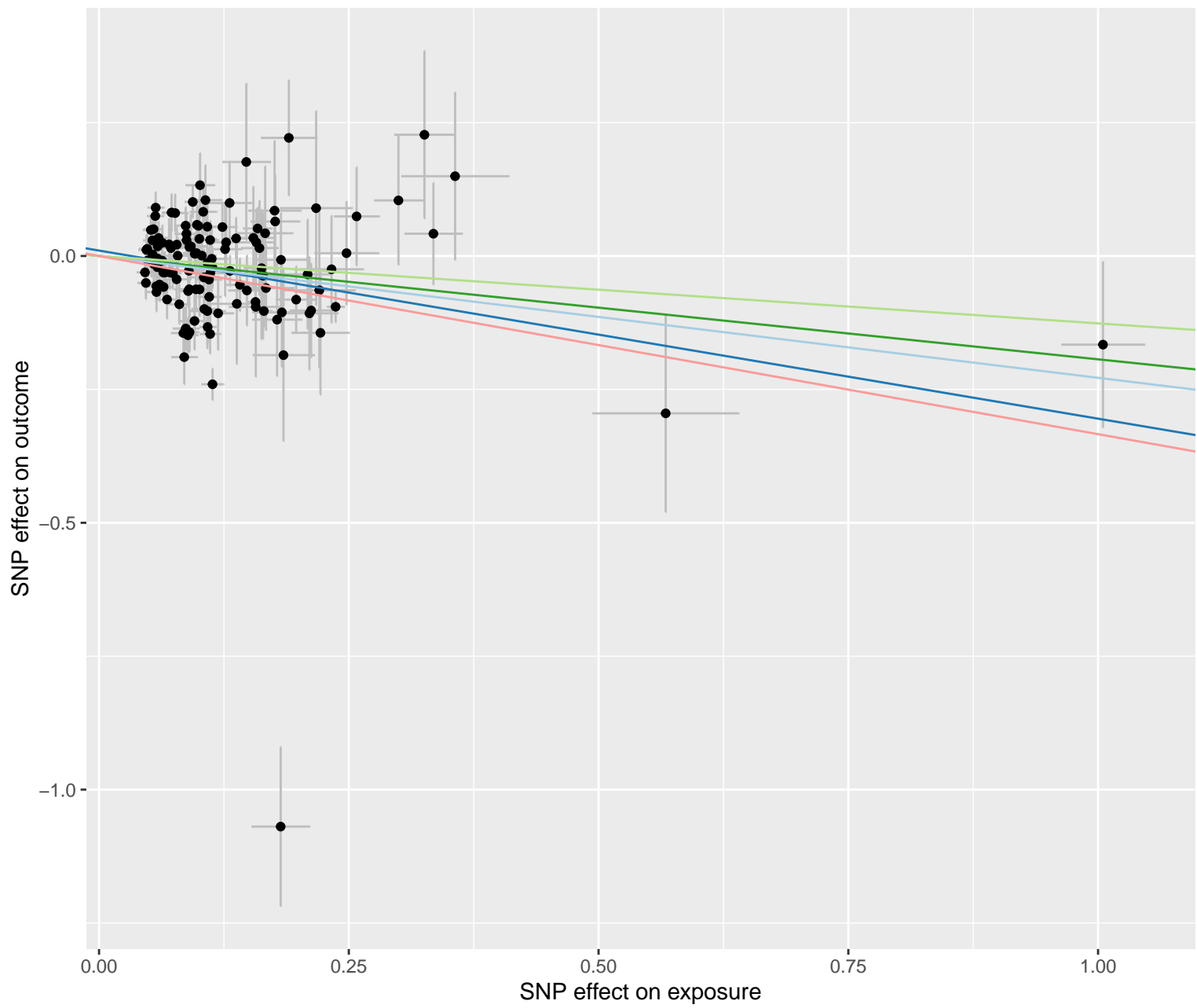

Supplement: Supplementary Data Sheet 1 — Harmonized summary data, forest plots, funnel plots, data sources, harmonization details, and sensitivity analyses for the Mendelian randomization analysis of pyroptosis-related proteins and ulcerative colitis. [file DataSheet1.zip › bdpqtlresult/3440_7_GZMA_granzyme_A/scatter.pdf]

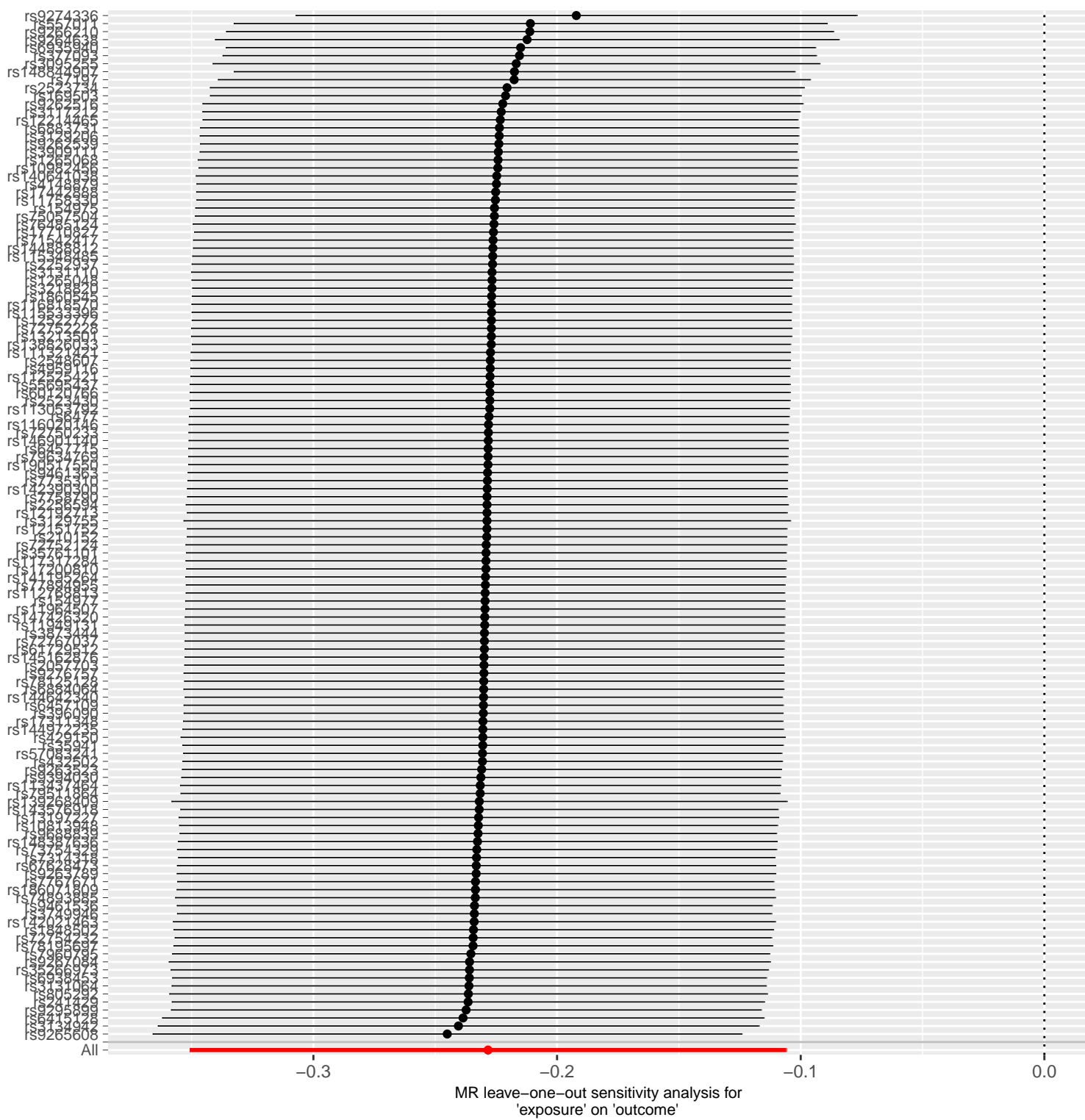

Supplement: Supplementary Data Sheet 1 — Harmonized summary data, forest plots, funnel plots, data sources, harmonization details, and sensitivity analyses for the Mendelian randomization analysis of pyroptosis-related proteins and ulcerative colitis. [file DataSheet1.zip › bdpqtlresult/3440_7_GZMA_granzyme_A/sensitivity-analysis.pdf]

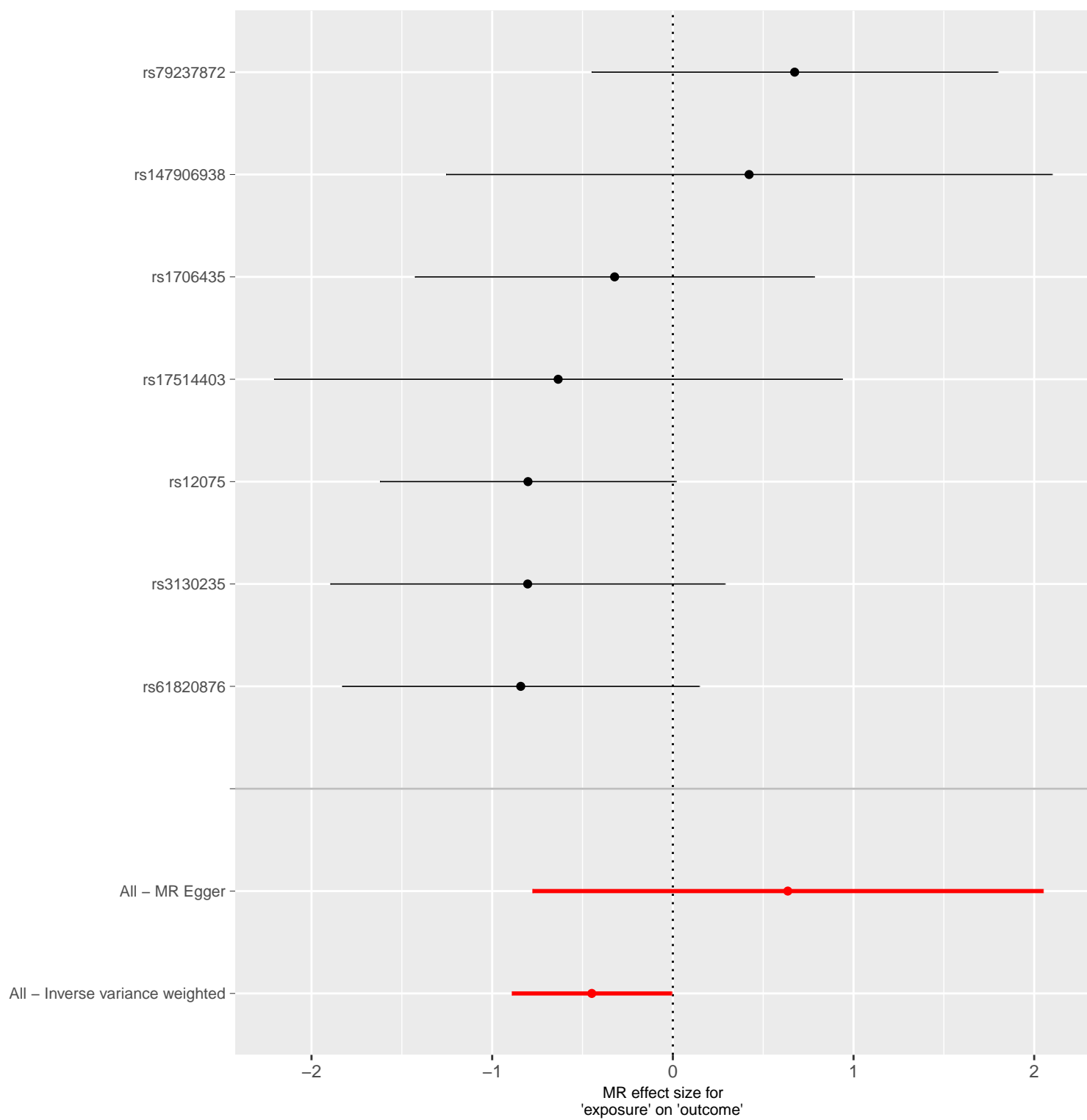

Supplement: Supplementary Data Sheet 1 — Harmonized summary data, forest plots, funnel plots, data sources, harmonization details, and sensitivity analyses for the Mendelian randomization analysis of pyroptosis-related proteins and ulcerative colitis. [file DataSheet1.zip › bdpqtlresult/3447_64_CXCL8_IL_8/forest.pdf]

# MR Method

- Inverse variance weighted
- MR Egger

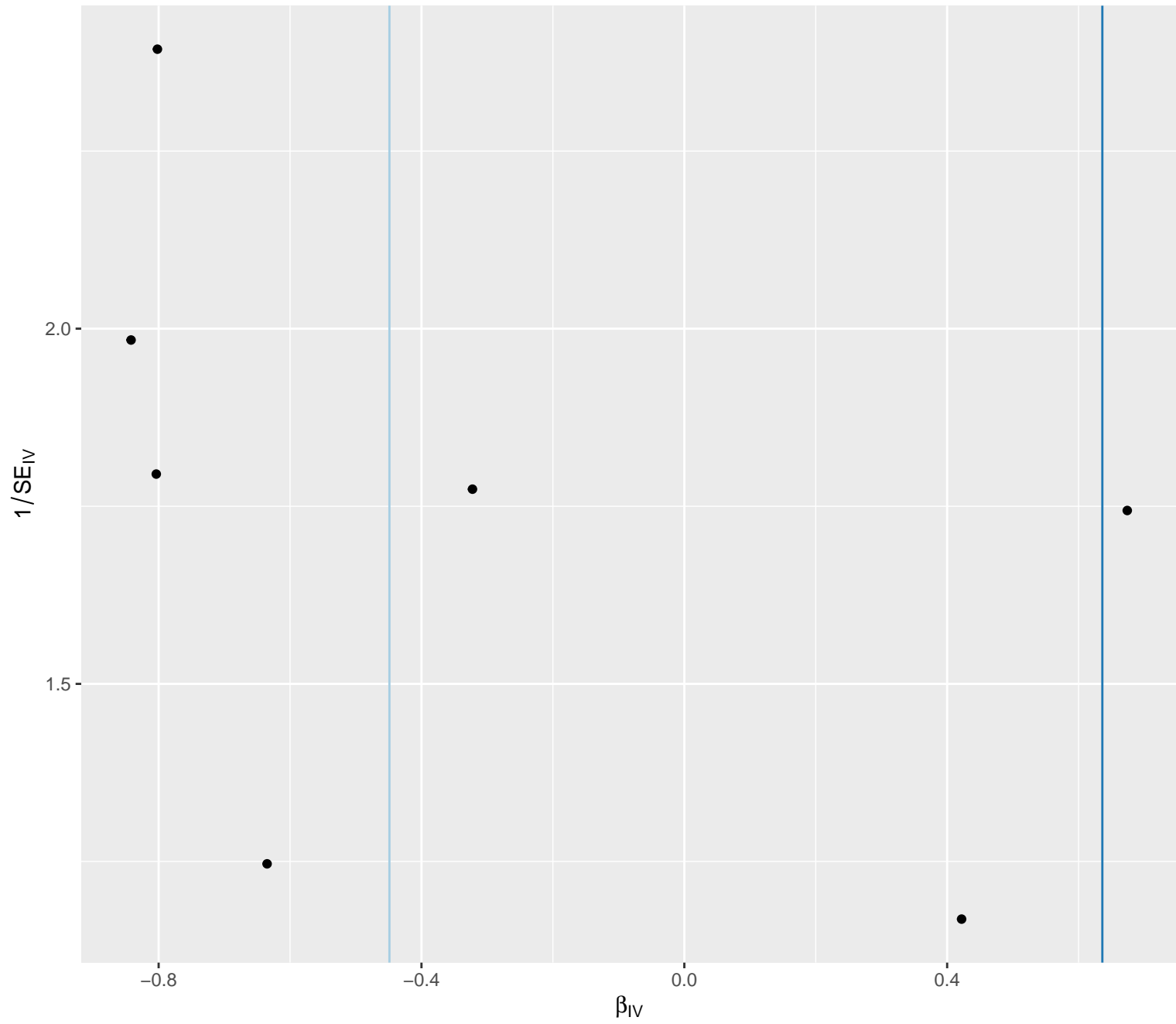

Supplement: Supplementary Data Sheet 1 — Harmonized summary data, forest plots, funnel plots, data sources, harmonization details, and sensitivity analyses for the Mendelian randomization analysis of pyroptosis-related proteins and ulcerative colitis. [file DataSheet1.zip › bdpqtlresult/3447_64_CXCL8_IL_8/funnelplot.pdf]

# MR Test

- Inverse variance weighted
- MR Egger
- Simple mode
- Weighted median
- Weighted mode

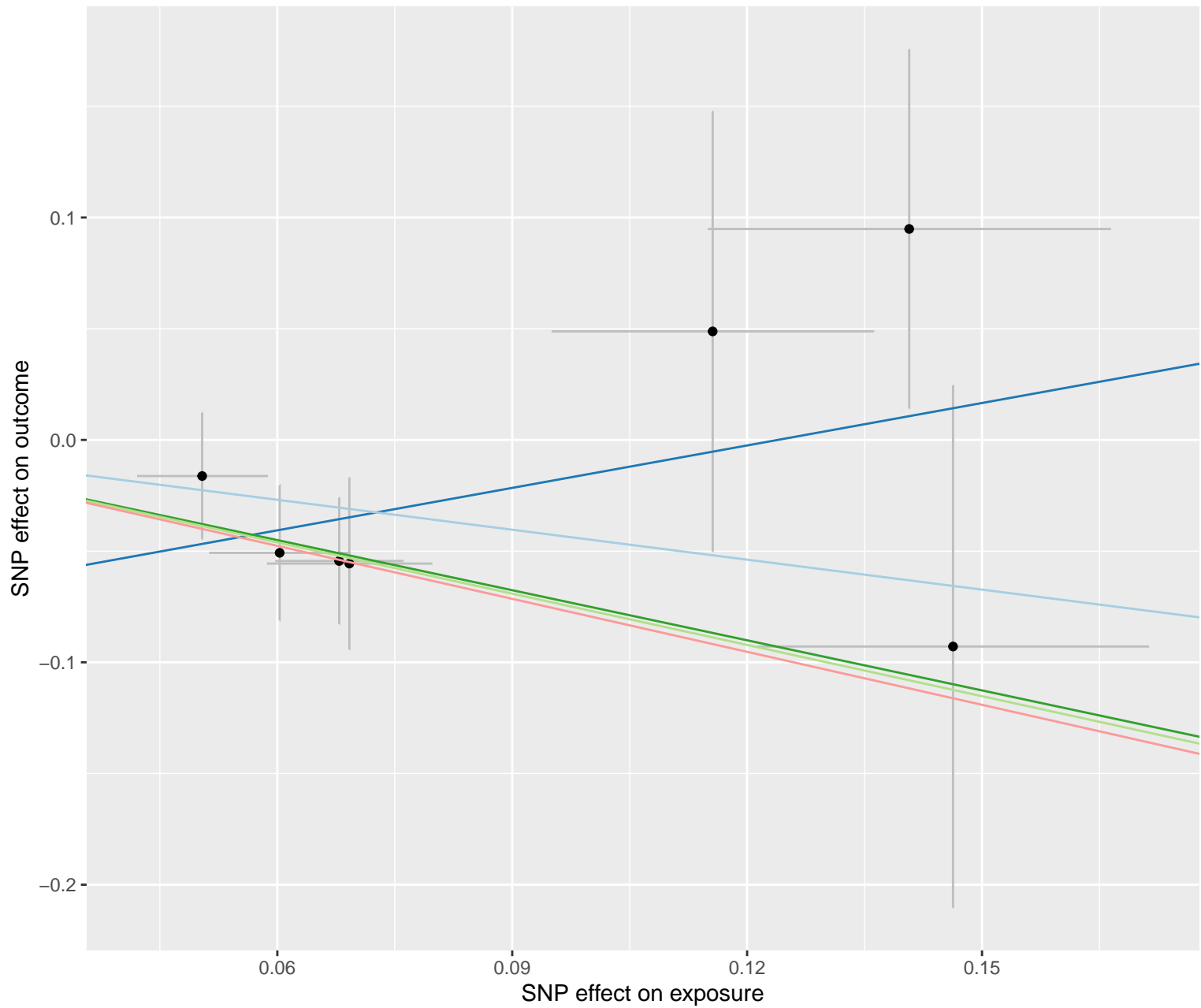

Supplement: Supplementary Data Sheet 1 — Harmonized summary data, forest plots, funnel plots, data sources, harmonization details, and sensitivity analyses for the Mendelian randomization analysis of pyroptosis-related proteins and ulcerative colitis. [file DataSheet1.zip › bdpqtlresult/3447_64_CXCL8_IL_8/scatter.pdf]

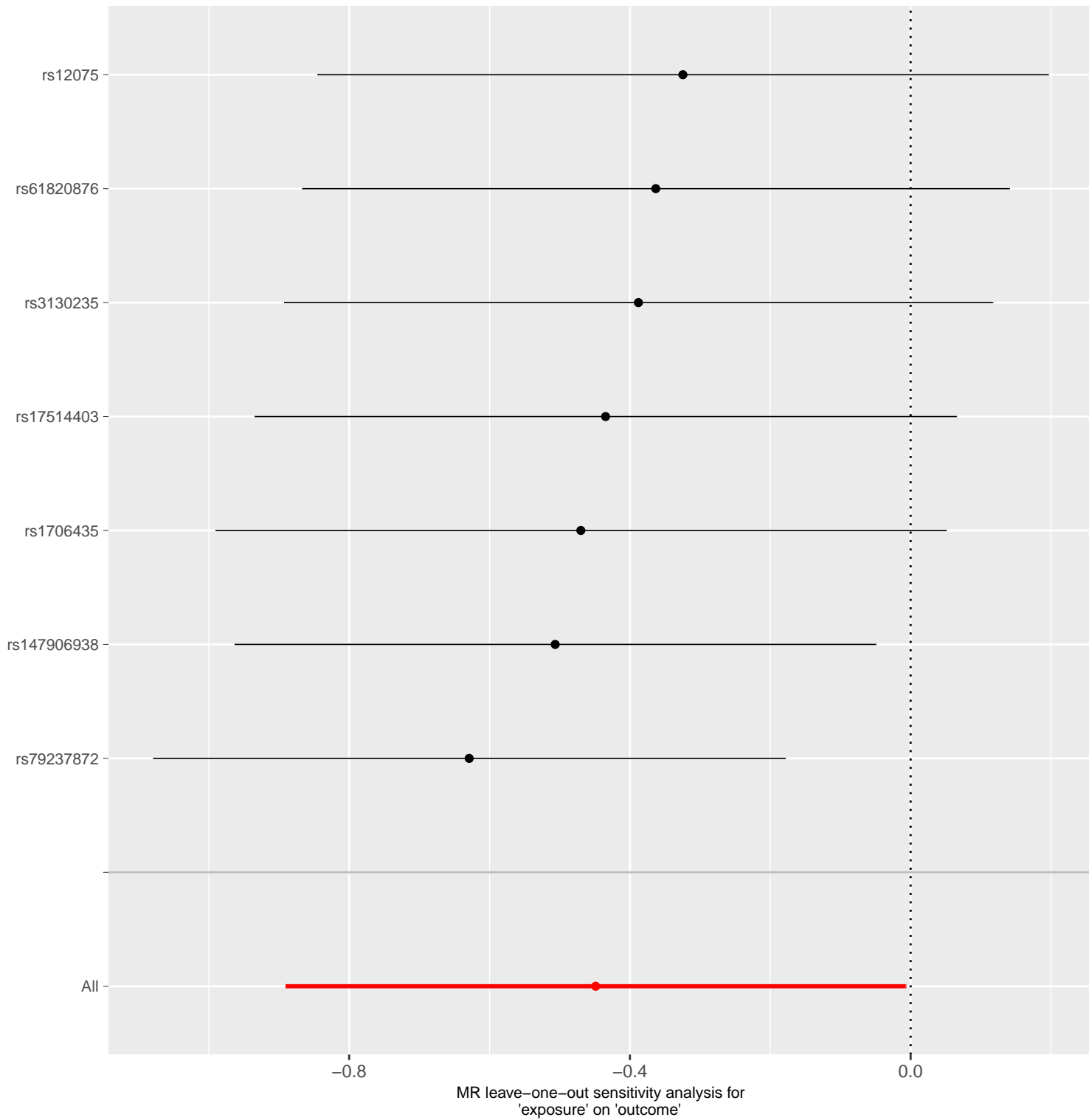

Supplement: Supplementary Data Sheet 1 — Harmonized summary data, forest plots, funnel plots, data sources, harmonization details, and sensitivity analyses for the Mendelian randomization analysis of pyroptosis-related proteins and ulcerative colitis. [file DataSheet1.zip › bdpqtlresult/3447_64_CXCL8_IL_8/sensitivity-analysis.pdf]

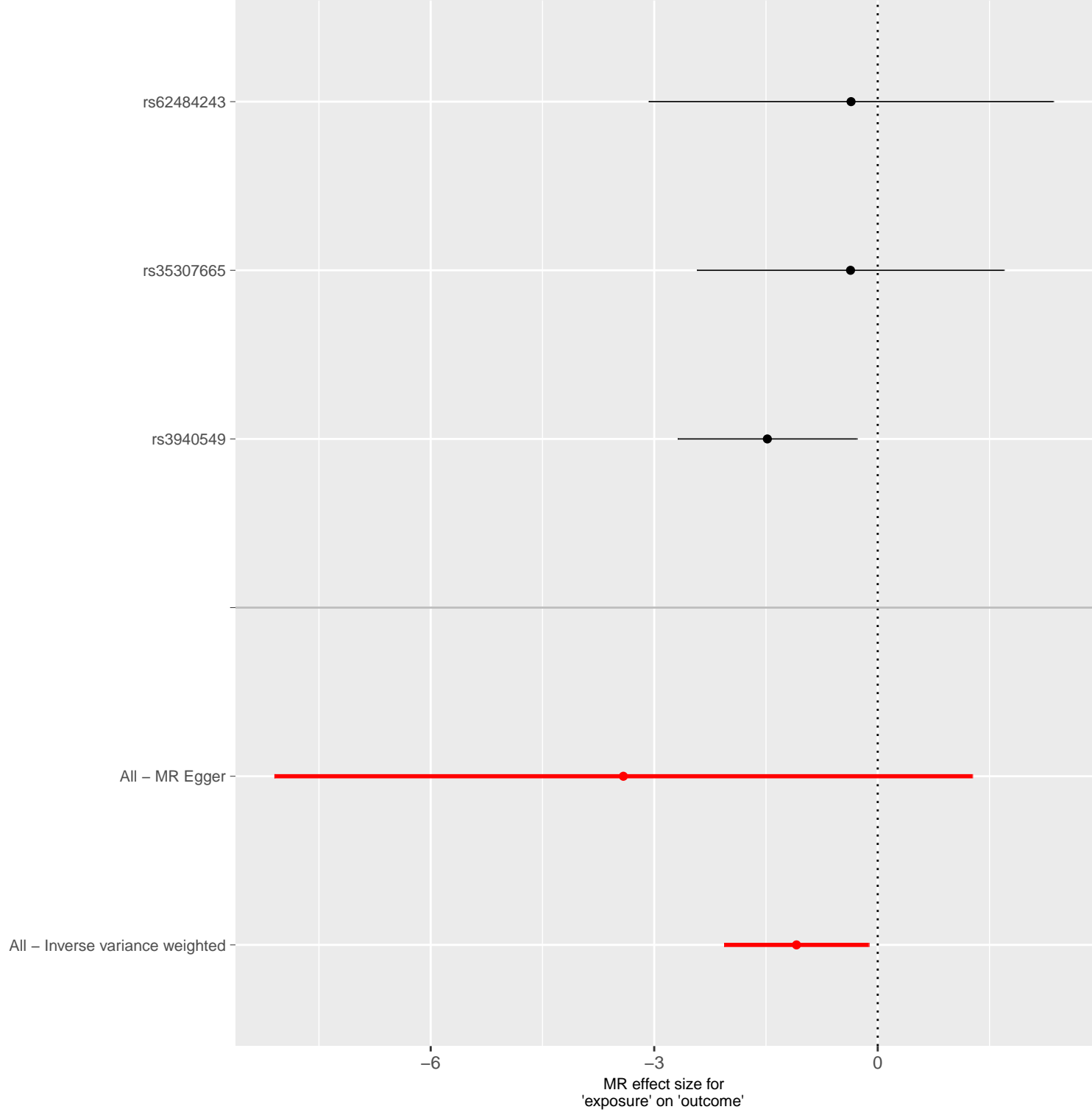

Supplement: Supplementary Data Sheet 2 — Full GCST identifiers, taxonomic labels, and Mendelian randomization statistics for the gut microbial traits associated with ulcerative colitis. [file DataSheet2.zip › GM_result/GCST90032180/forest.pdf]

# MR Method

- Inverse variance weighted
- MR Egger

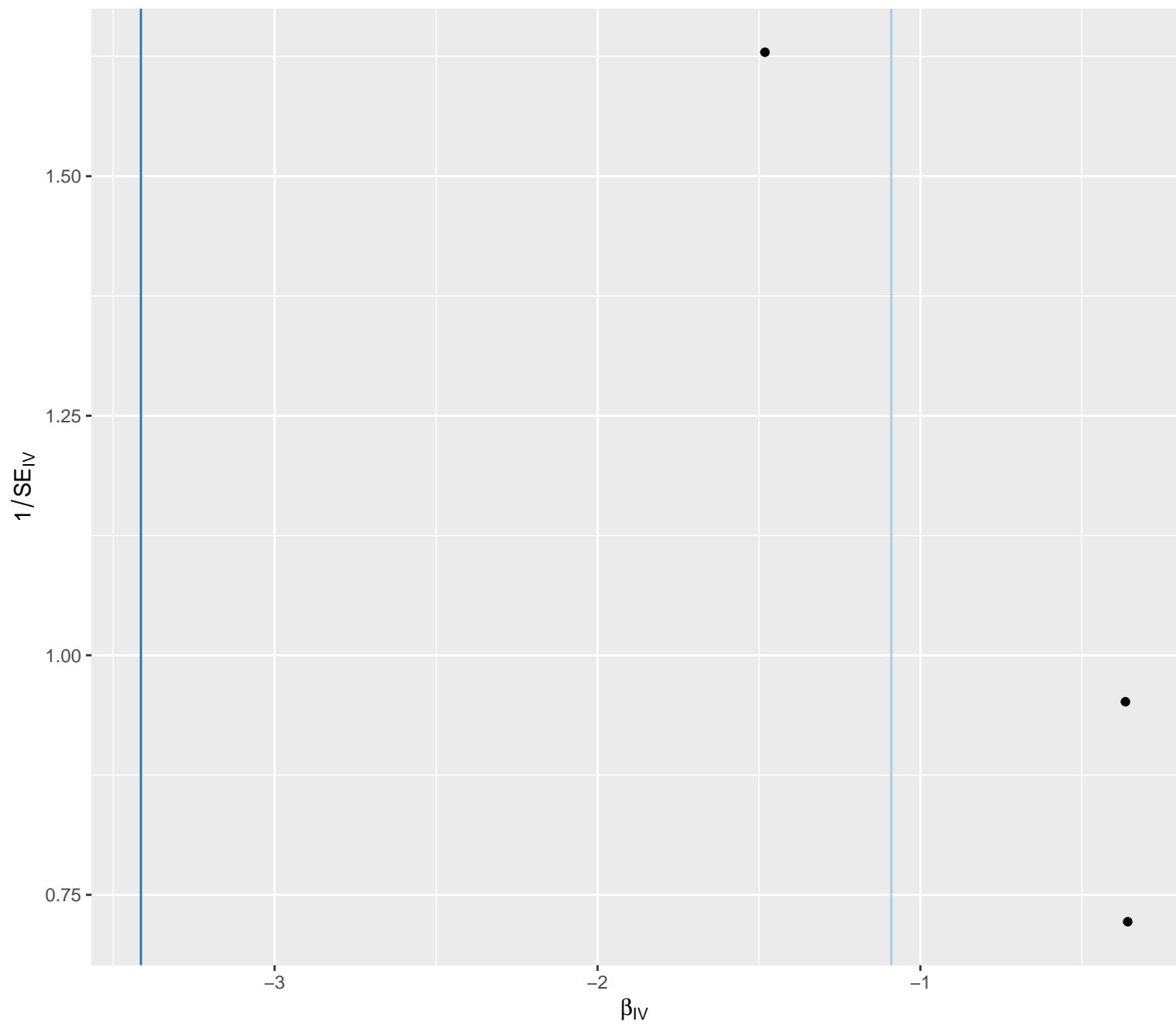

Supplement: Supplementary Data Sheet 2 — Full GCST identifiers, taxonomic labels, and Mendelian randomization statistics for the gut microbial traits associated with ulcerative colitis. [file DataSheet2.zip › GM_result/GCST90032180/funnelplot.pdf]

# MR Test

- Inverse variance weighted
- MR Egger
- Simple mode
- Weighted median
- Weighted mode

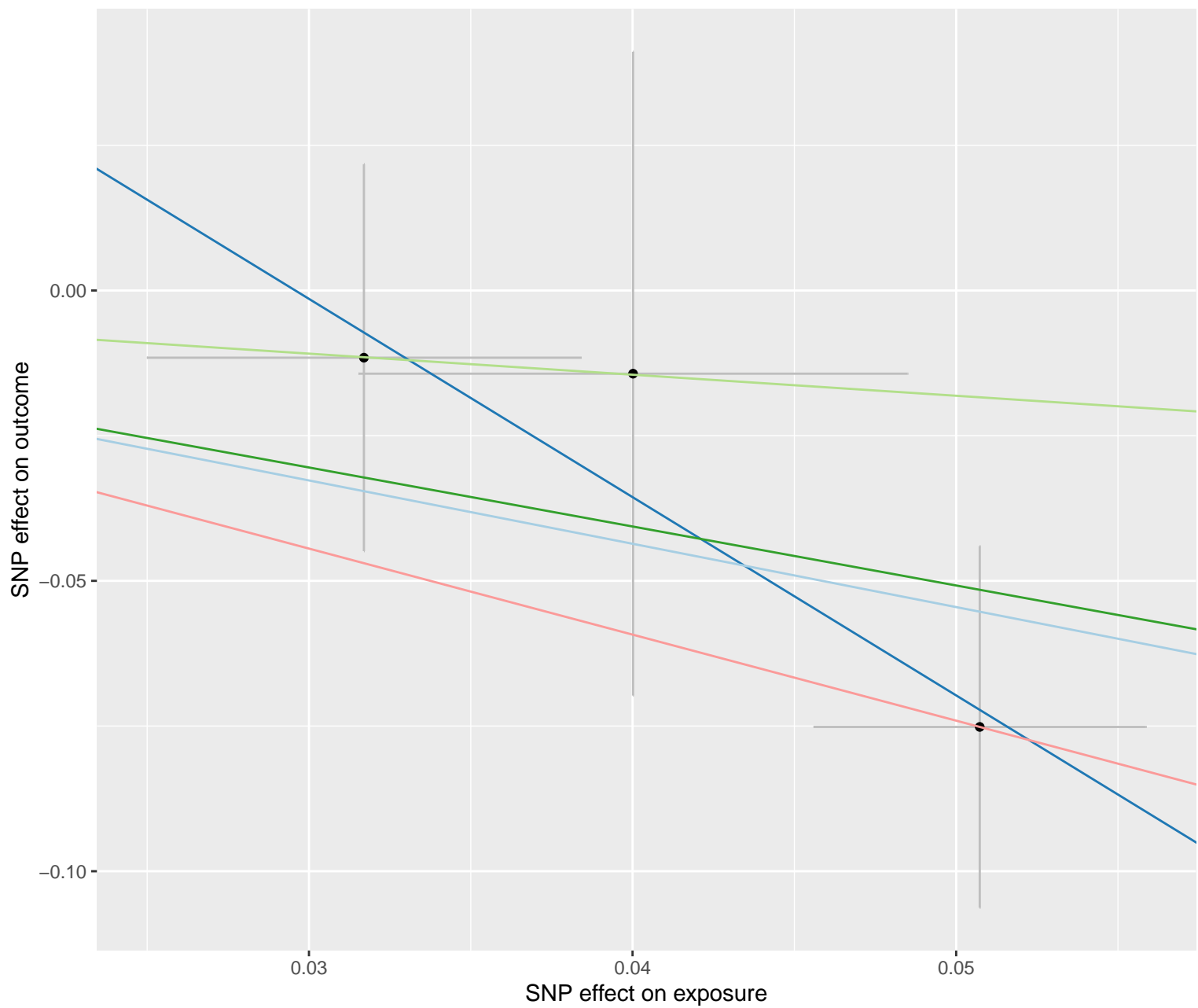

Supplement: Supplementary Data Sheet 2 — Full GCST identifiers, taxonomic labels, and Mendelian randomization statistics for the gut microbial traits associated with ulcerative colitis. [file DataSheet2.zip › GM_result/GCST90032180/scatter.pdf]

rs3940549

rs62484243

rs35307665

All

-2

-1

0

1

MR leave-one-out sensitivity analysis for  
'exposure' on 'outcome'

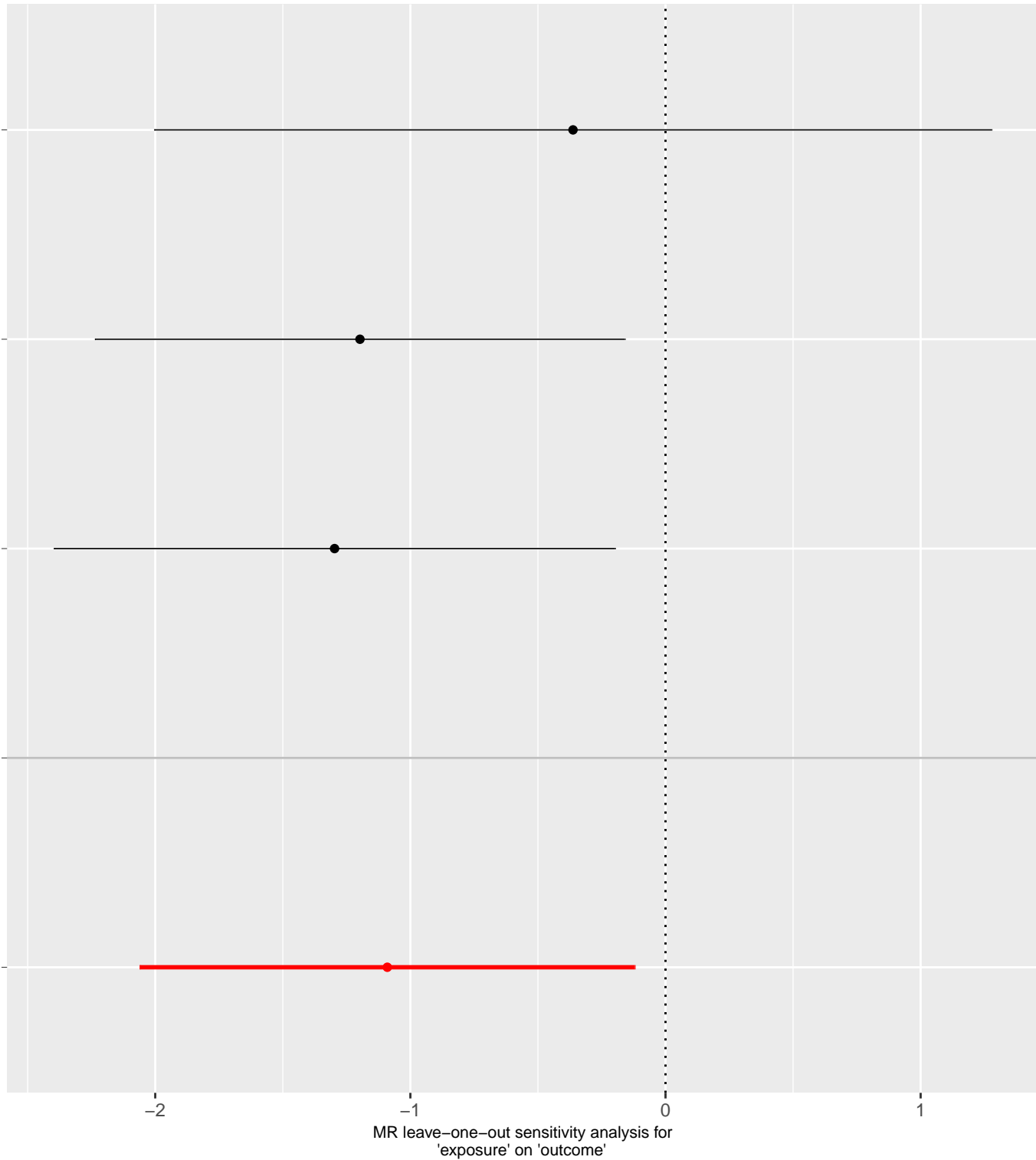

Supplement: Supplementary Data Sheet 2 — Full GCST identifiers, taxonomic labels, and Mendelian randomization statistics for the gut microbial traits associated with ulcerative colitis. [file DataSheet2.zip › GM_result/GCST90032180/sensitivity-analysis.pdf]

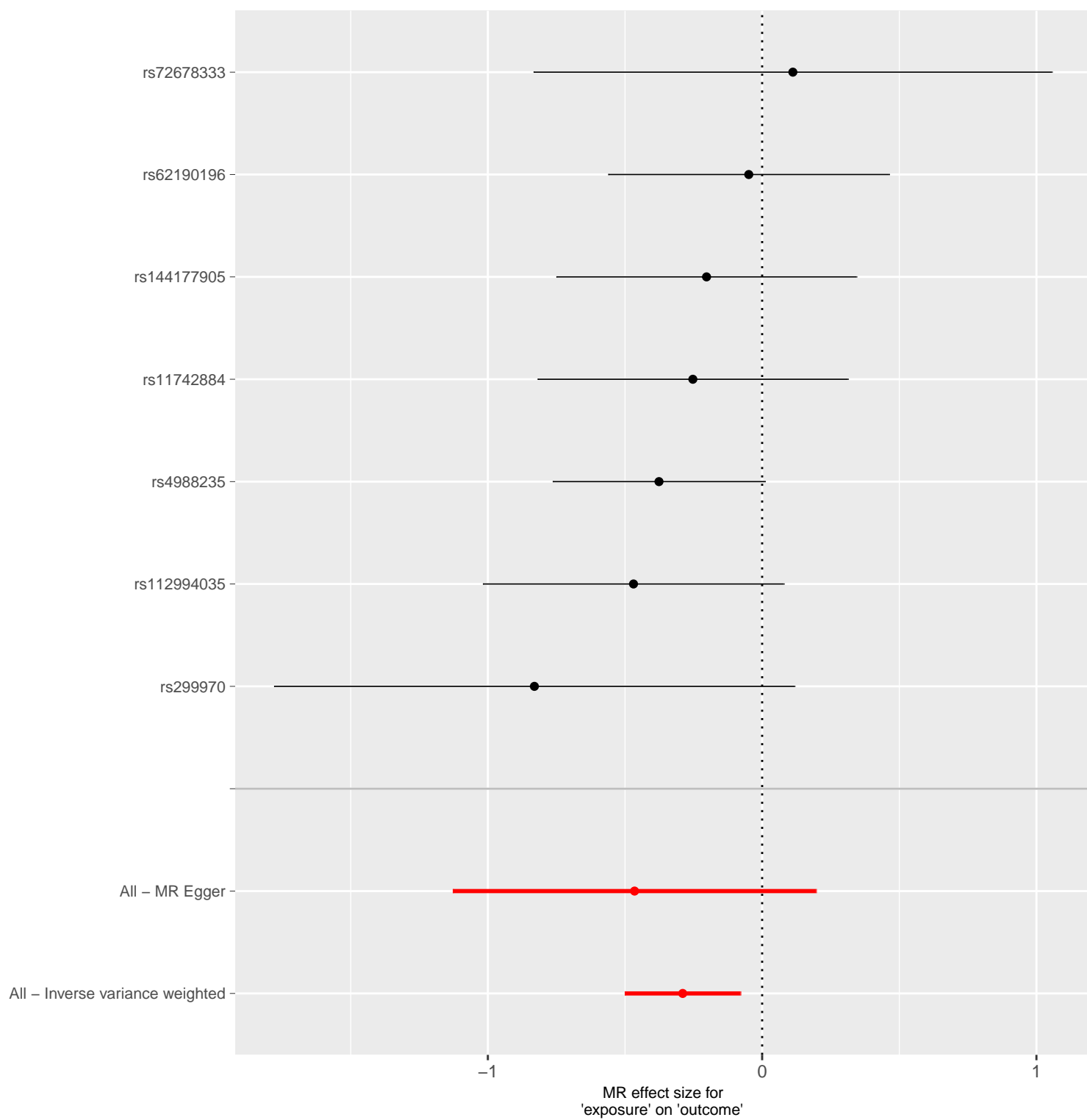

Supplement: Supplementary Data Sheet 2 — Full GCST identifiers, taxonomic labels, and Mendelian randomization statistics for the gut microbial traits associated with ulcerative colitis. [file DataSheet2.zip › GM_result/GCST90032222/forest.pdf]

# MR Method

- Inverse variance weighted
- MR Egger

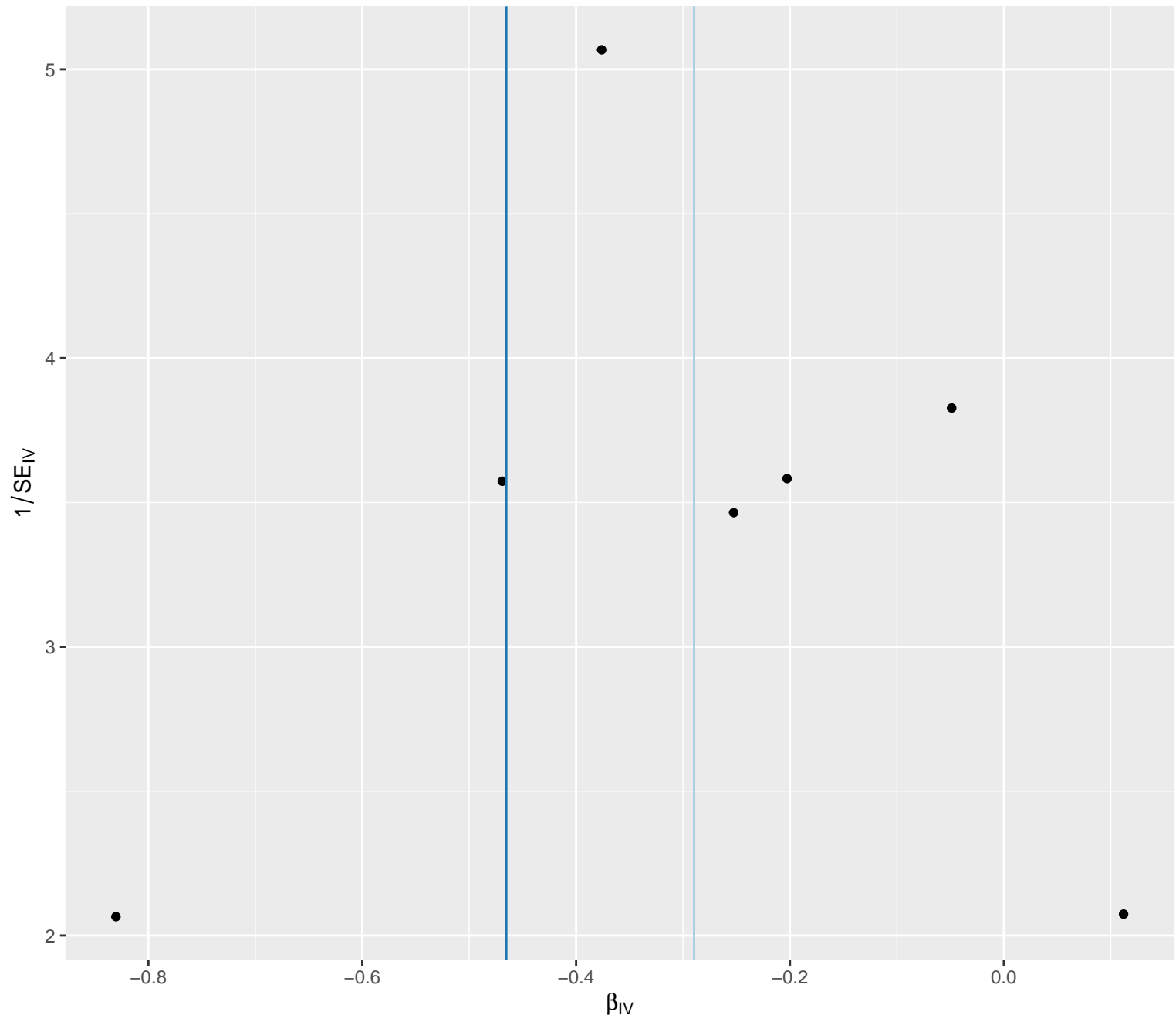

Supplement: Supplementary Data Sheet 2 — Full GCST identifiers, taxonomic labels, and Mendelian randomization statistics for the gut microbial traits associated with ulcerative colitis. [file DataSheet2.zip › GM_result/GCST90032222/funnelplot.pdf]

# MR Test

- Inverse variance weighted
- MR Egger
- Simple mode
- Weighted median
- Weighted mode

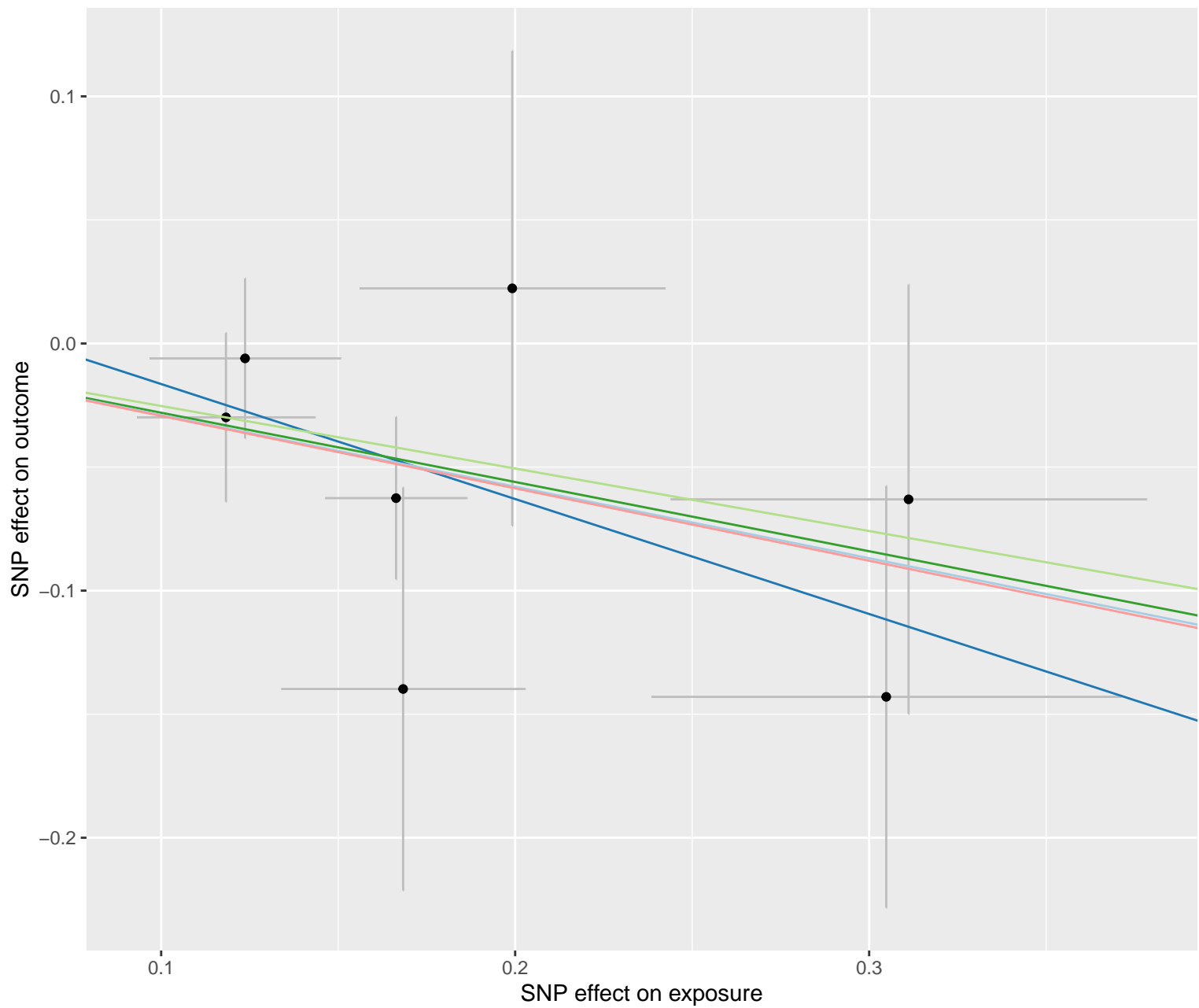

Supplement: Supplementary Data Sheet 2 — Full GCST identifiers, taxonomic labels, and Mendelian randomization statistics for the gut microbial traits associated with ulcerative colitis. [file DataSheet2.zip › GM_result/GCST90032222/scatter.pdf]

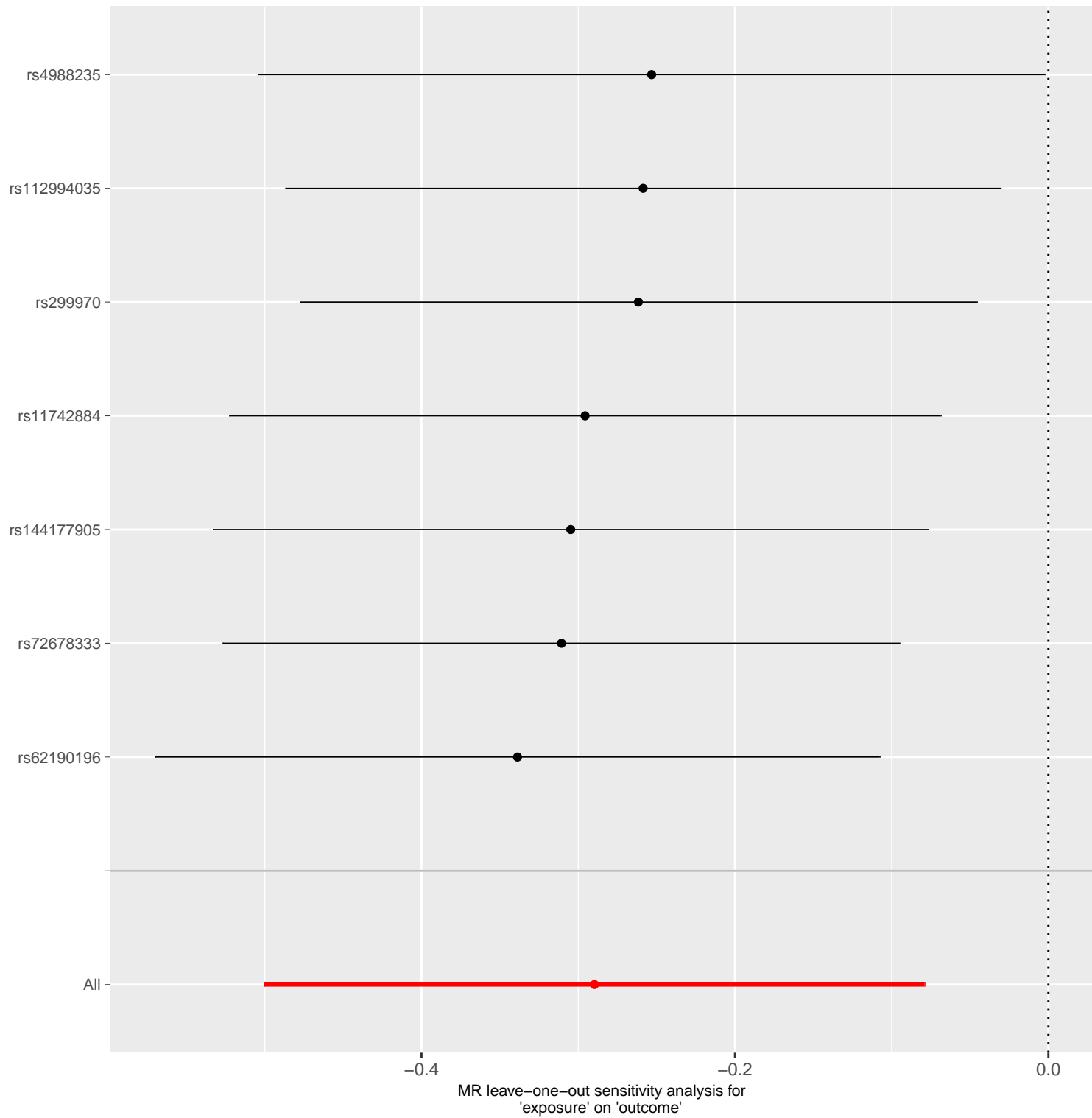

Supplement: Supplementary Data Sheet 2 — Full GCST identifiers, taxonomic labels, and Mendelian randomization statistics for the gut microbial traits associated with ulcerative colitis. [file DataSheet2.zip › GM_result/GCST90032222/sensitivity-analysis.pdf]

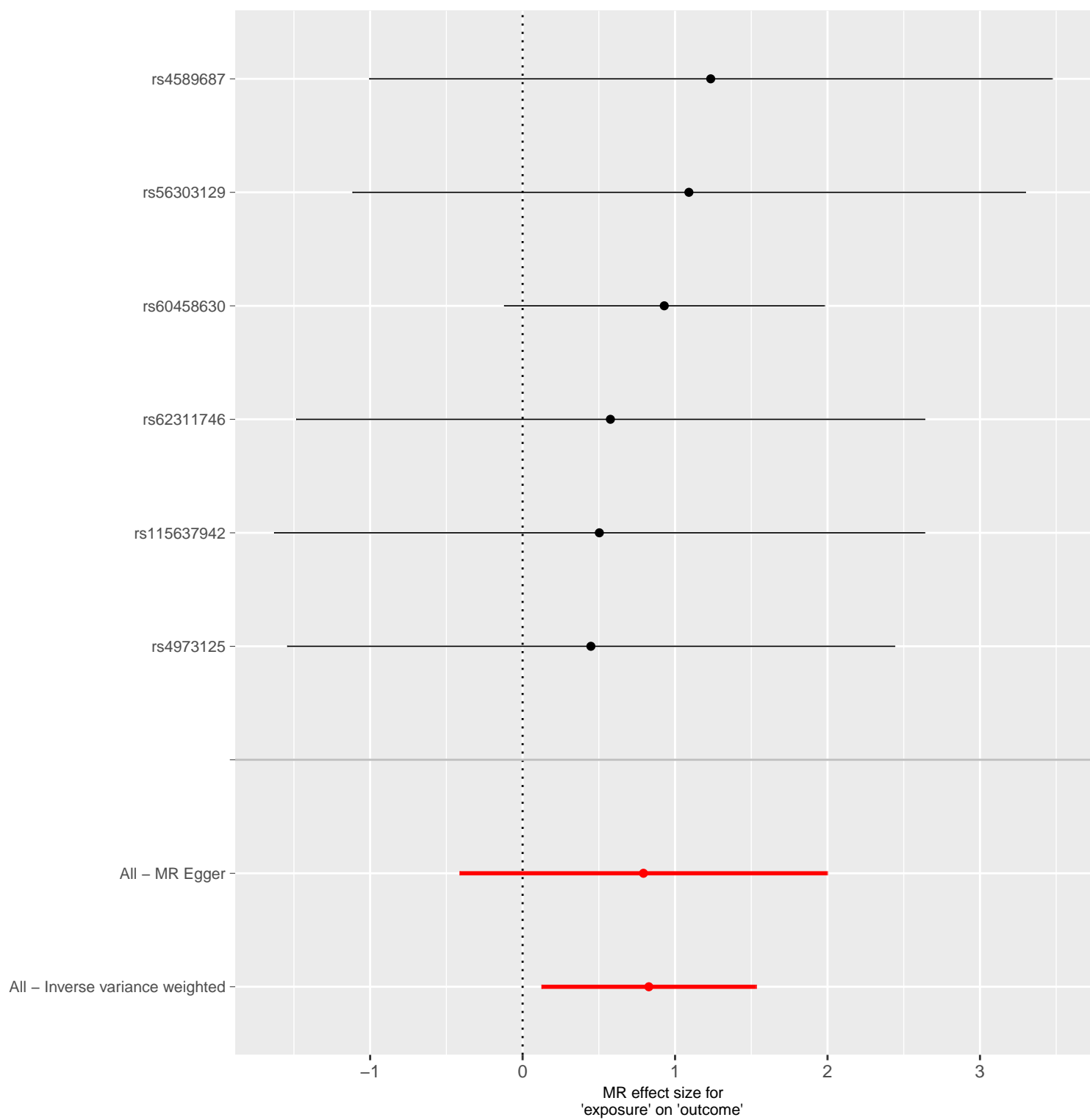

Supplement: Supplementary Data Sheet 2 — Full GCST identifiers, taxonomic labels, and Mendelian randomization statistics for the gut microbial traits associated with ulcerative colitis. [file DataSheet2.zip › GM_result/GCST90032239/forest.pdf]

# MR Method

- Inverse variance weighted
- MR Egger

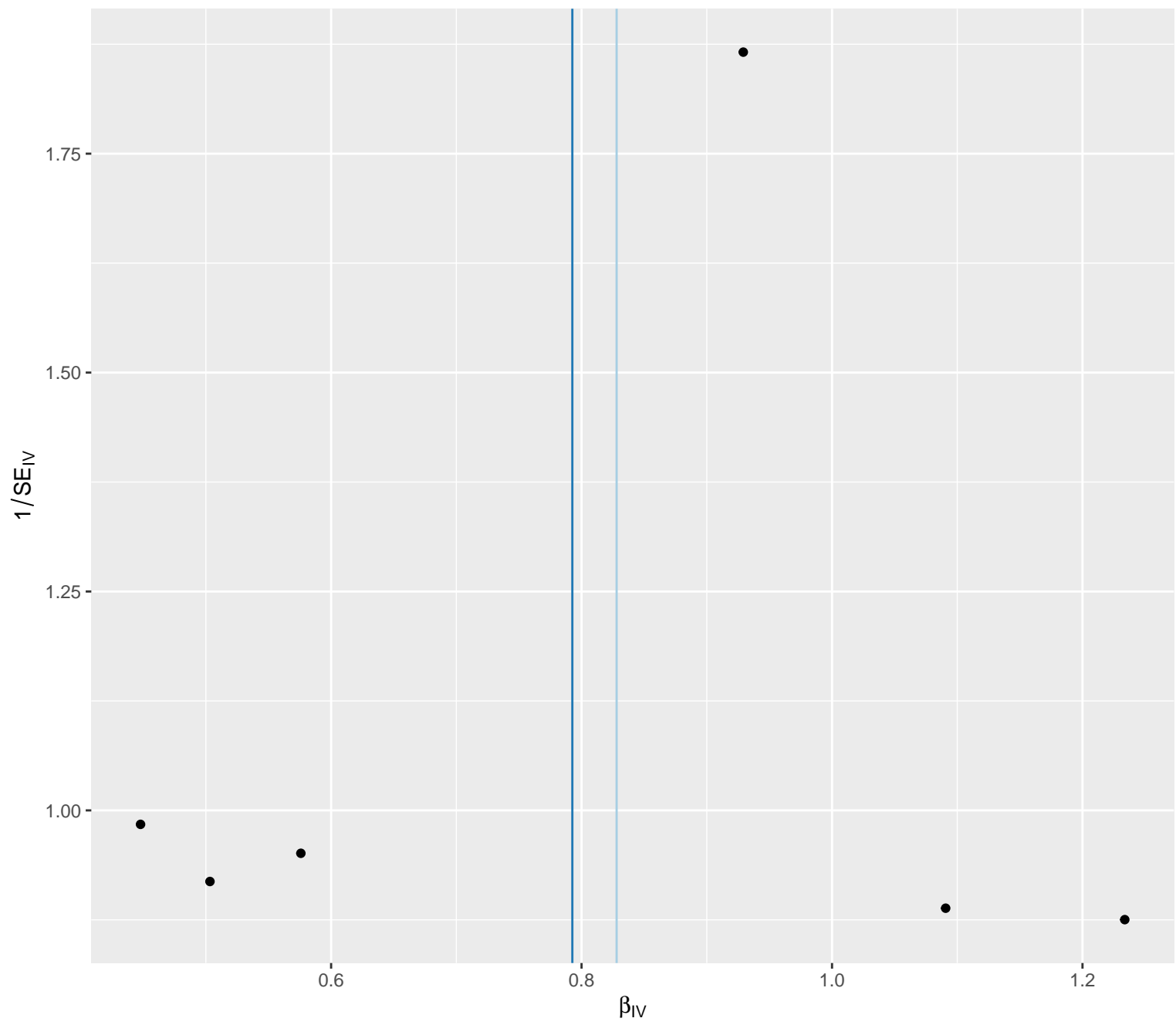

Supplement: Supplementary Data Sheet 2 — Full GCST identifiers, taxonomic labels, and Mendelian randomization statistics for the gut microbial traits associated with ulcerative colitis. [file DataSheet2.zip › GM_result/GCST90032239/funnelplot.pdf]

# MR Test

- Inverse variance weighted
- MR Egger
- Simple mode
- Weighted median
- Weighted mode

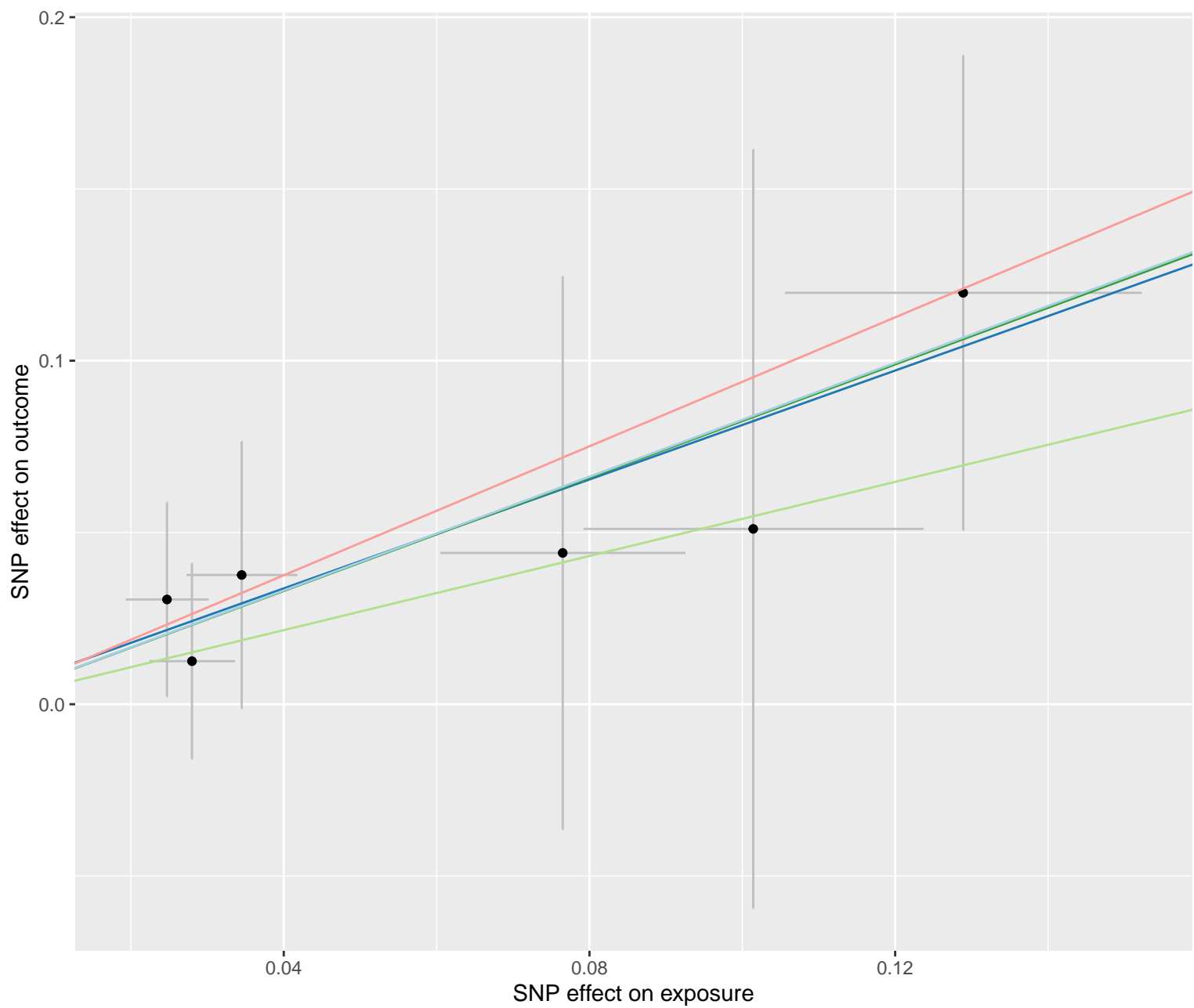

Supplement: Supplementary Data Sheet 2 — Full GCST identifiers, taxonomic labels, and Mendelian randomization statistics for the gut microbial traits associated with ulcerative colitis. [file DataSheet2.zip › GM_result/GCST90032239/scatter.pdf]

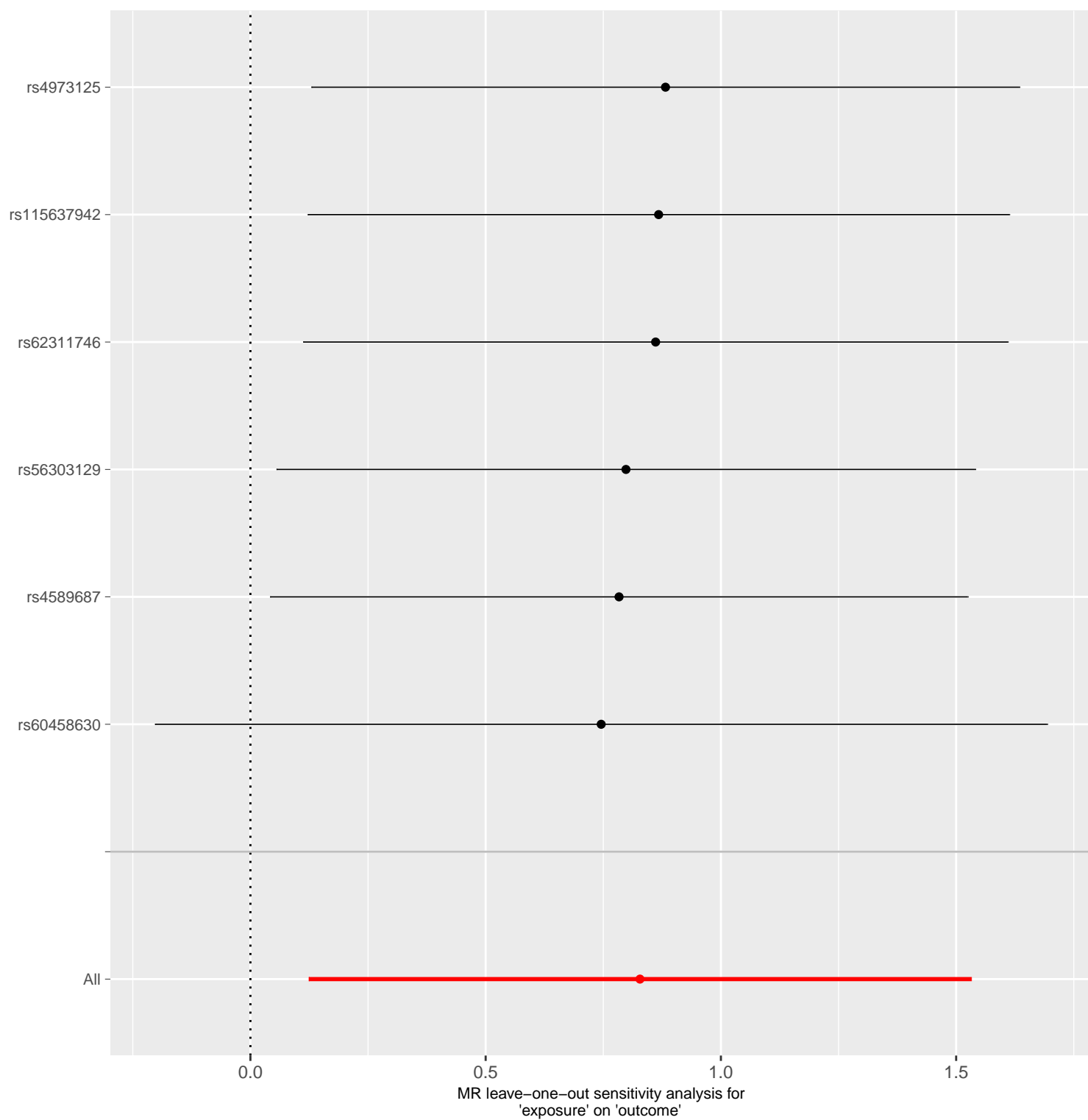

Supplement: Supplementary Data Sheet 2 — Full GCST identifiers, taxonomic labels, and Mendelian randomization statistics for the gut microbial traits associated with ulcerative colitis. [file DataSheet2.zip › GM_result/GCST90032239/sensitivity-analysis.pdf]

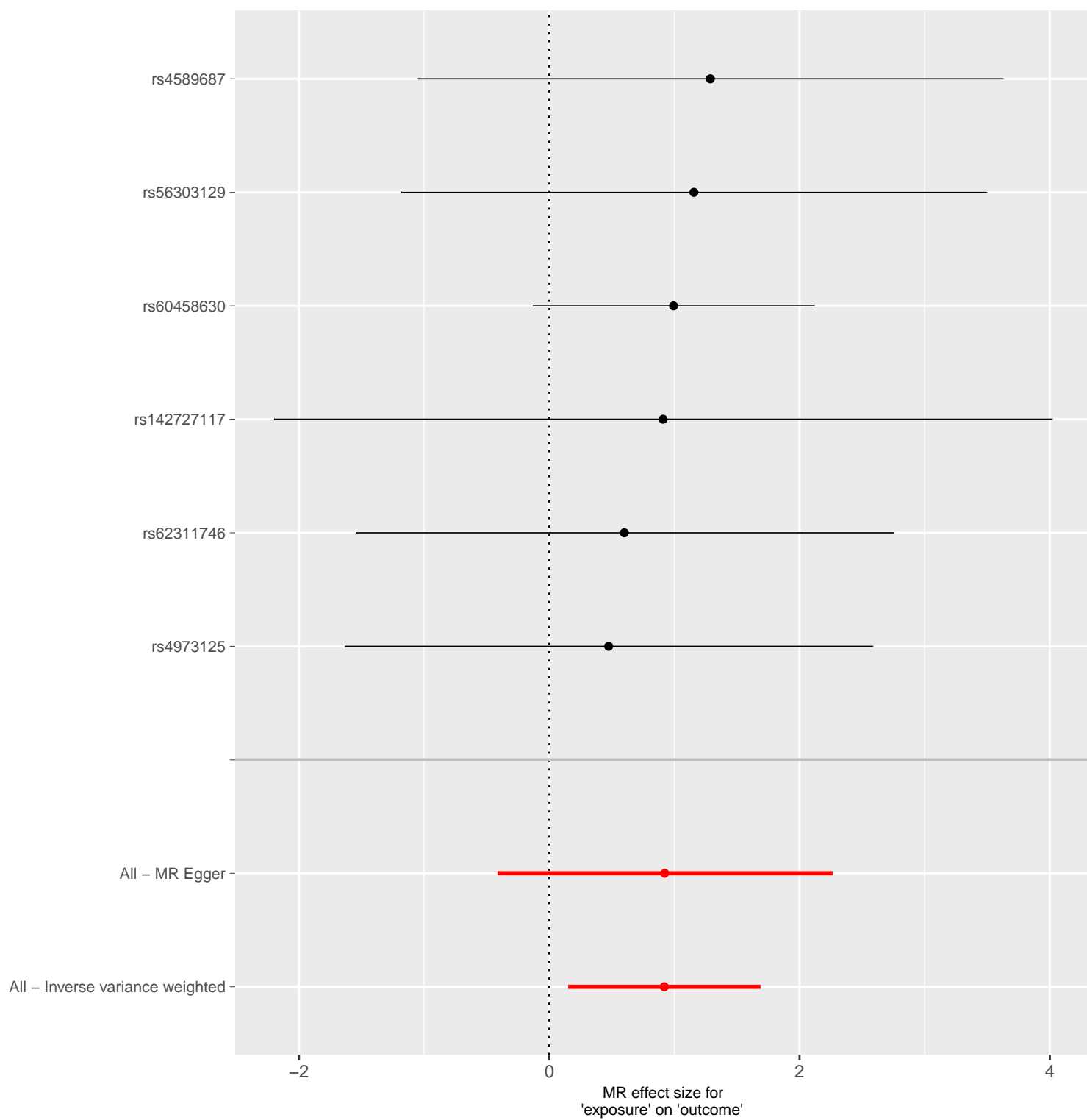

Supplement: Supplementary Data Sheet 2 — Full GCST identifiers, taxonomic labels, and Mendelian randomization statistics for the gut microbial traits associated with ulcerative colitis. [file DataSheet2.zip › GM_result/GCST90032240/forest.pdf]

# MR Method

- Inverse variance weighted
- MR Egger

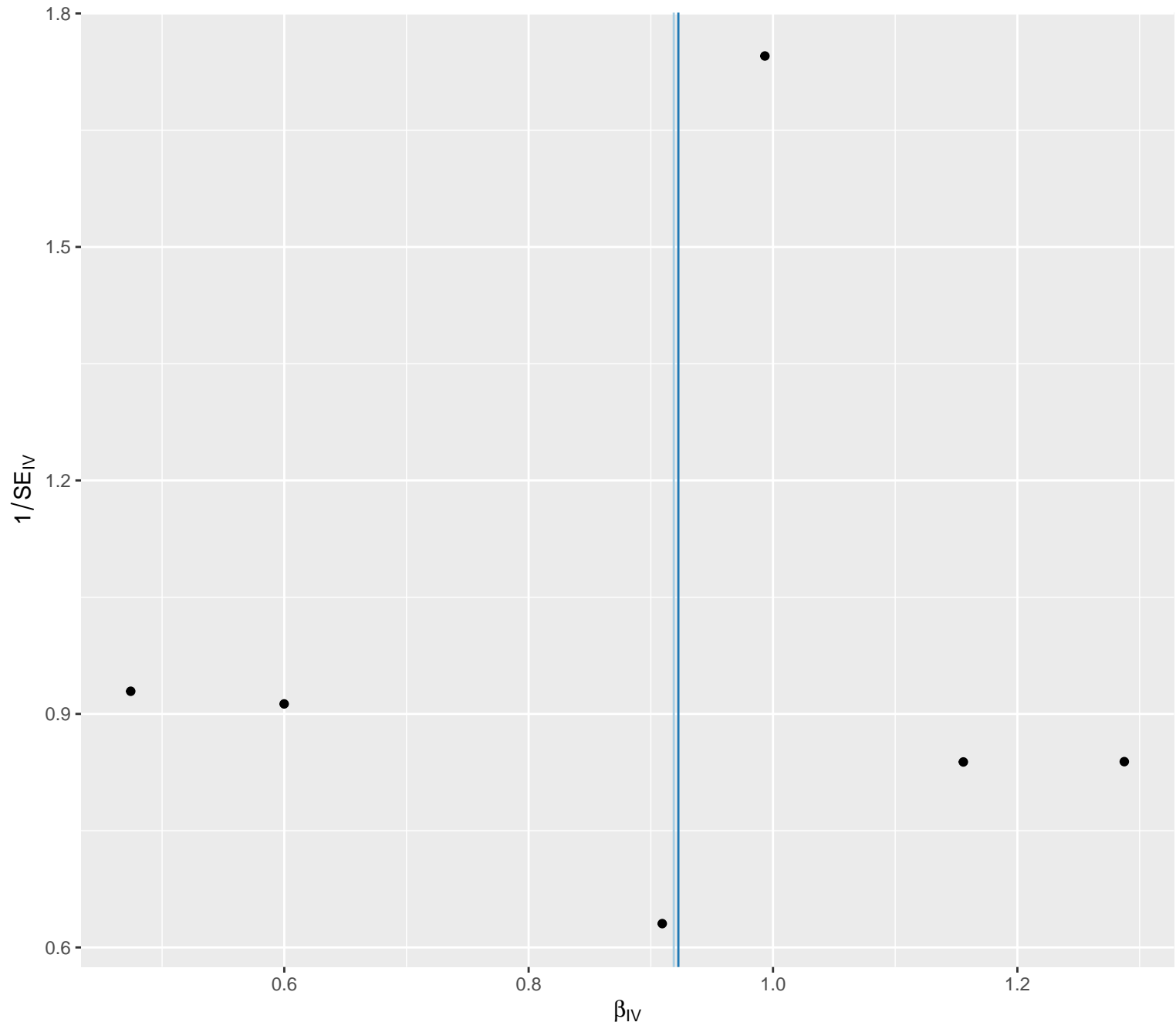

Supplement: Supplementary Data Sheet 2 — Full GCST identifiers, taxonomic labels, and Mendelian randomization statistics for the gut microbial traits associated with ulcerative colitis. [file DataSheet2.zip › GM_result/GCST90032240/funnelplot.pdf]

# MR Test

- Inverse variance weighted
- MR Egger
- Simple mode
- Weighted median
- Weighted mode

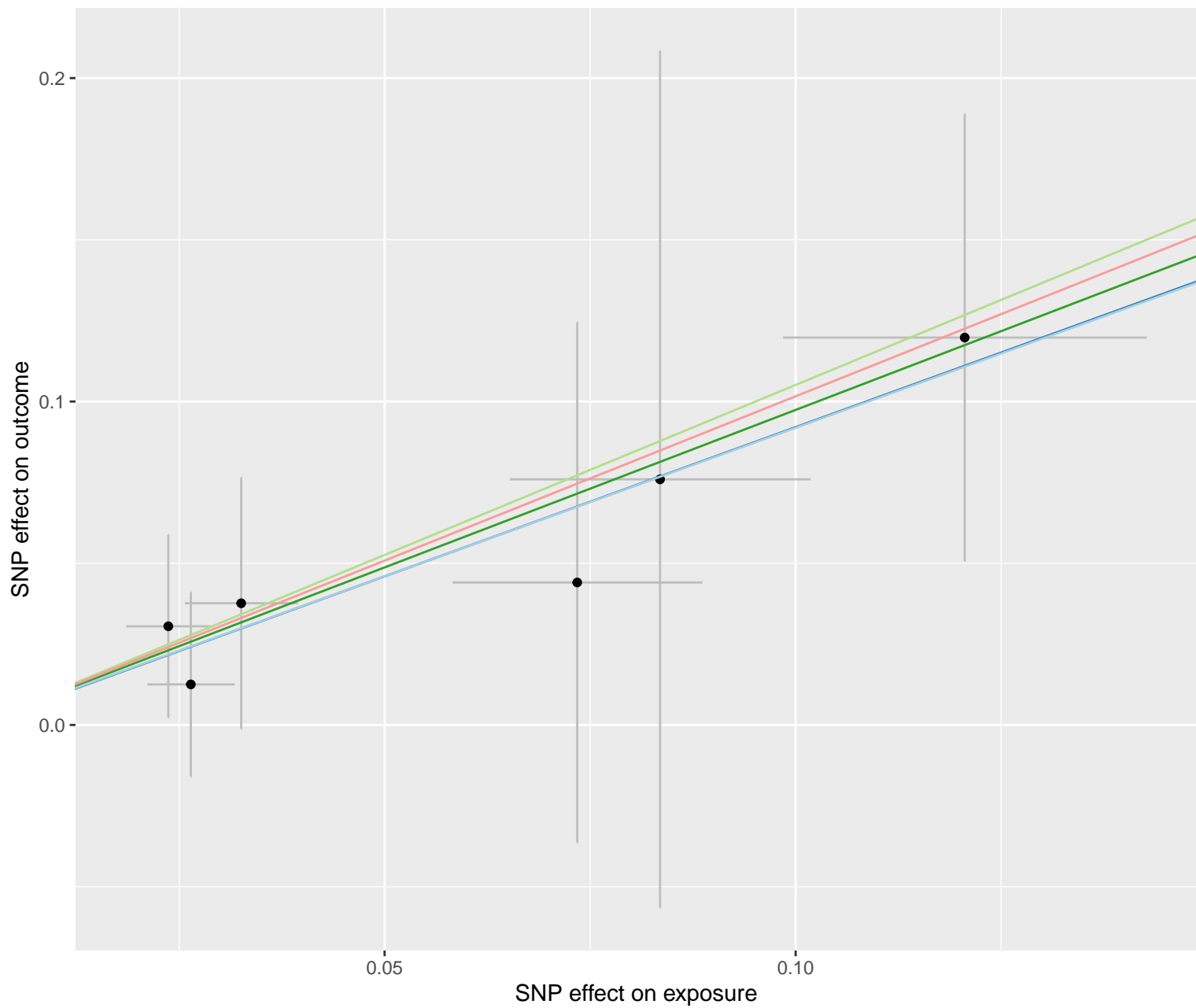

Supplement: Supplementary Data Sheet 2 — Full GCST identifiers, taxonomic labels, and Mendelian randomization statistics for the gut microbial traits associated with ulcerative colitis. [file DataSheet2.zip › GM_result/GCST90032240/scatter.pdf]

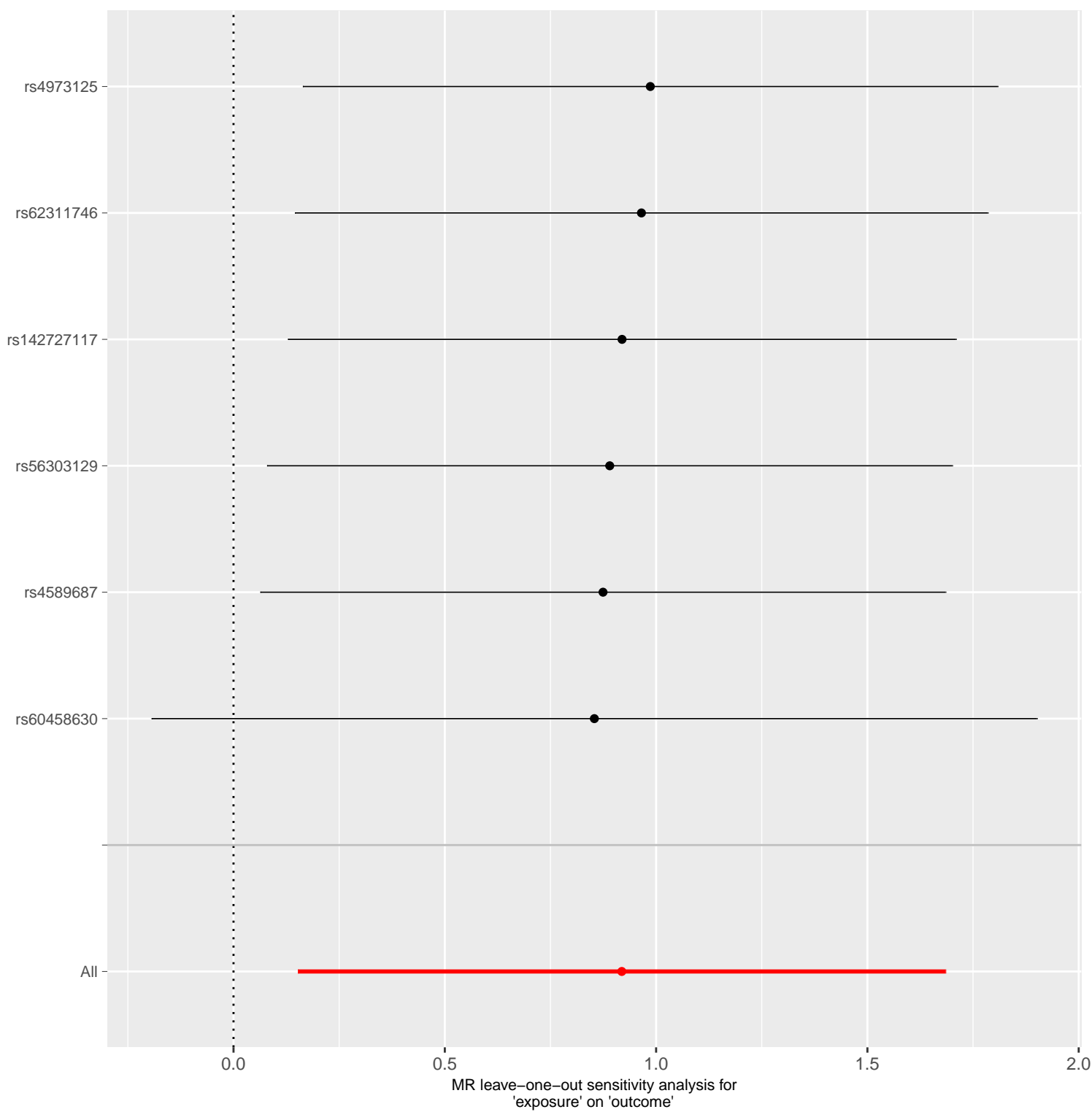

Supplement: Supplementary Data Sheet 2 — Full GCST identifiers, taxonomic labels, and Mendelian randomization statistics for the gut microbial traits associated with ulcerative colitis. [file DataSheet2.zip › GM_result/GCST90032240/sensitivity-analysis.pdf]

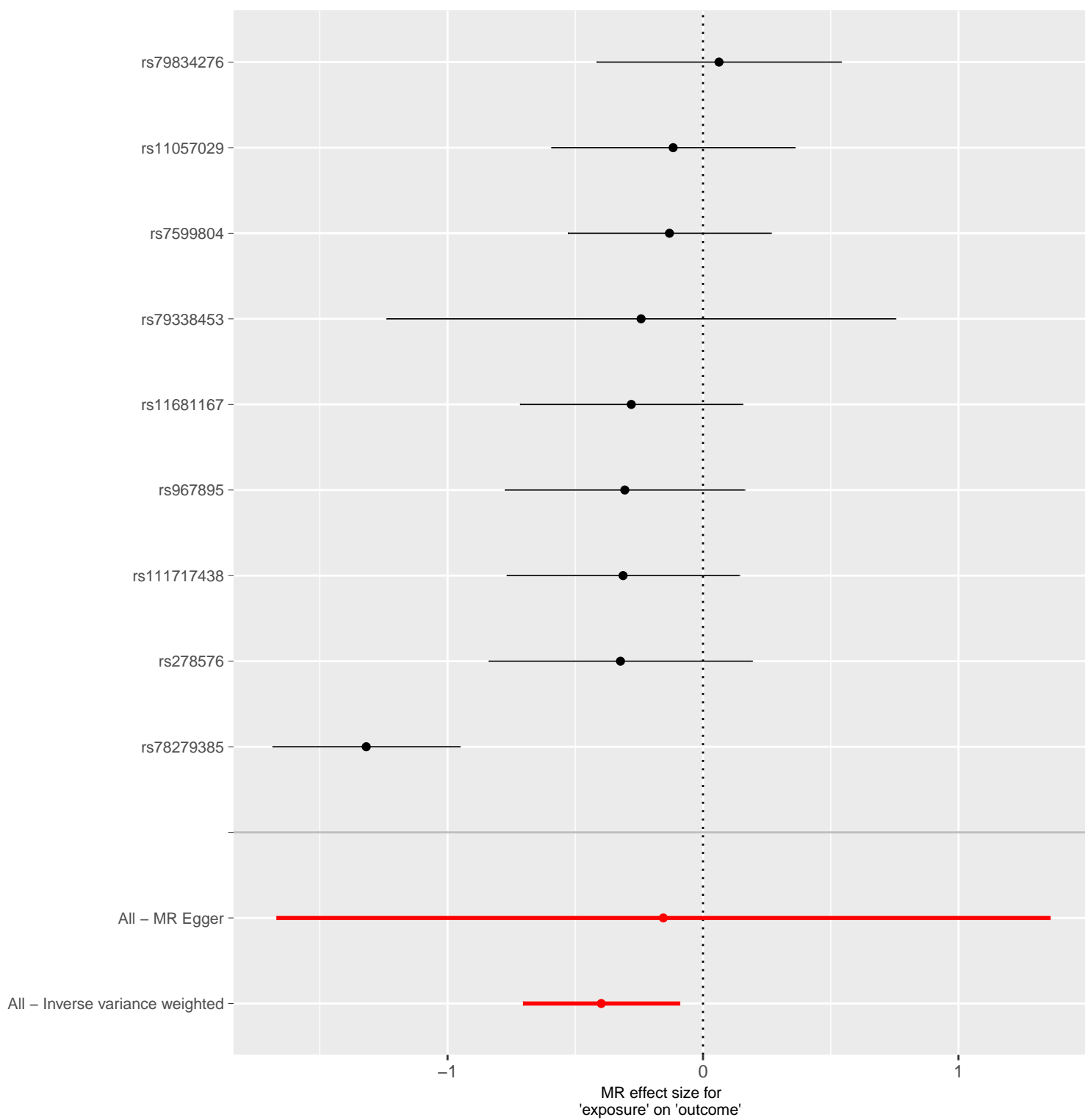

Supplement: Supplementary Data Sheet 2 — Full GCST identifiers, taxonomic labels, and Mendelian randomization statistics for the gut microbial traits associated with ulcerative colitis. [file DataSheet2.zip › GM_result/GCST90032262/forest.pdf]

# MR Method

- Inverse variance weighted
- MR Egger

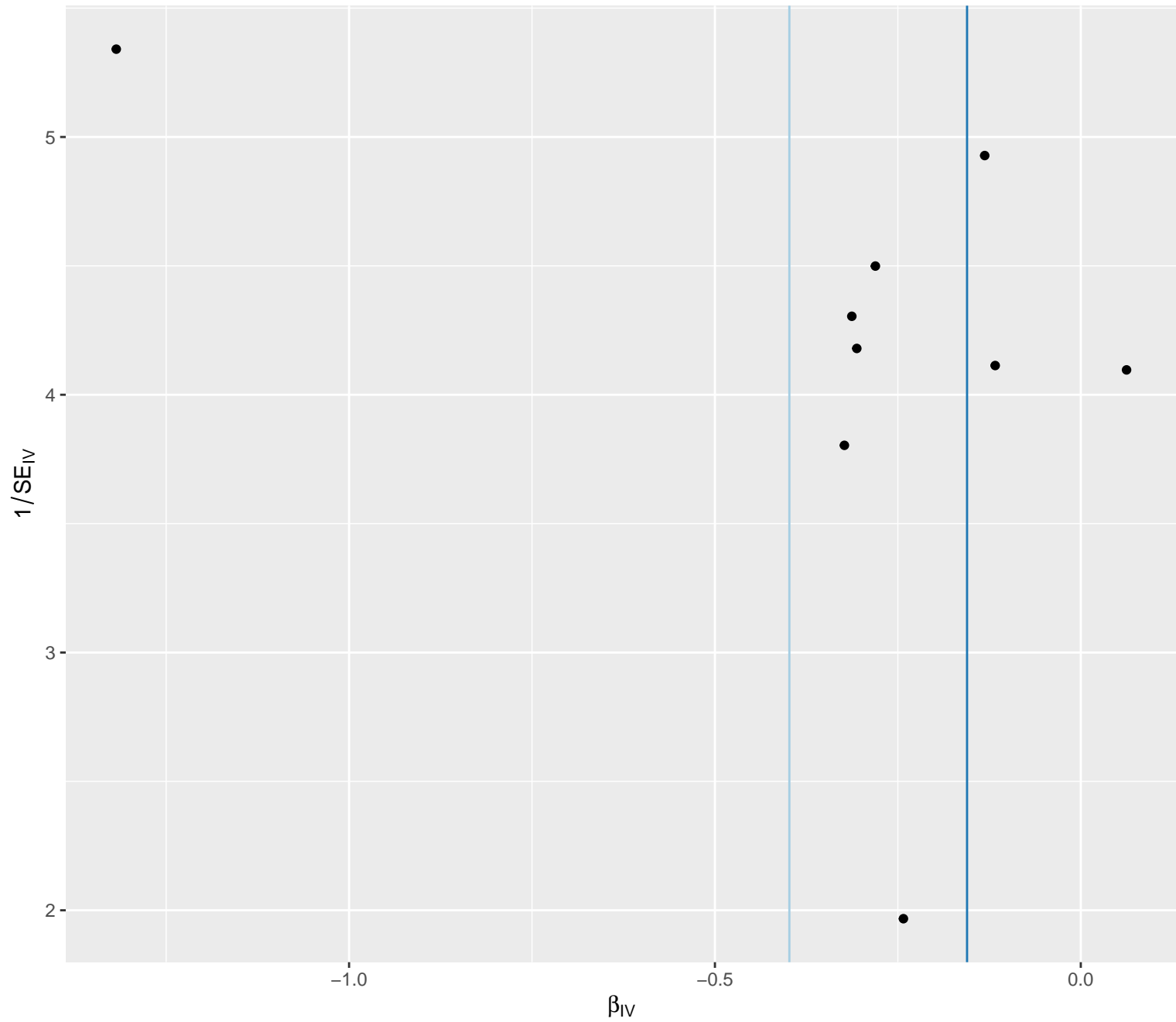

Supplement: Supplementary Data Sheet 2 — Full GCST identifiers, taxonomic labels, and Mendelian randomization statistics for the gut microbial traits associated with ulcerative colitis. [file DataSheet2.zip › GM_result/GCST90032262/funnelplot.pdf]

# MR Test

- Inverse variance weighted
- MR Egger
- Simple mode
- Weighted median
- Weighted mode

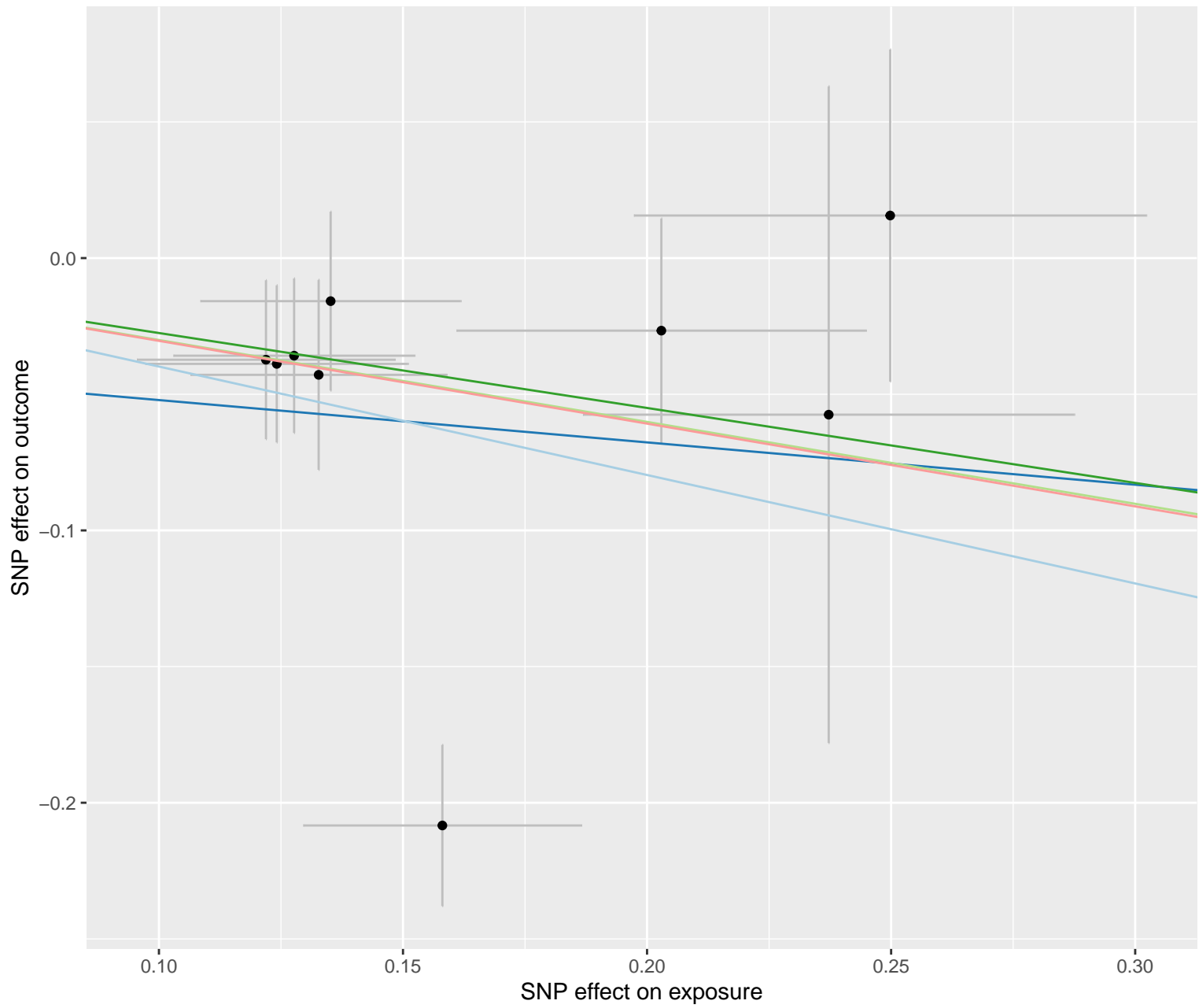

Supplement: Supplementary Data Sheet 2 — Full GCST identifiers, taxonomic labels, and Mendelian randomization statistics for the gut microbial traits associated with ulcerative colitis. [file DataSheet2.zip › GM_result/GCST90032262/scatter.pdf]

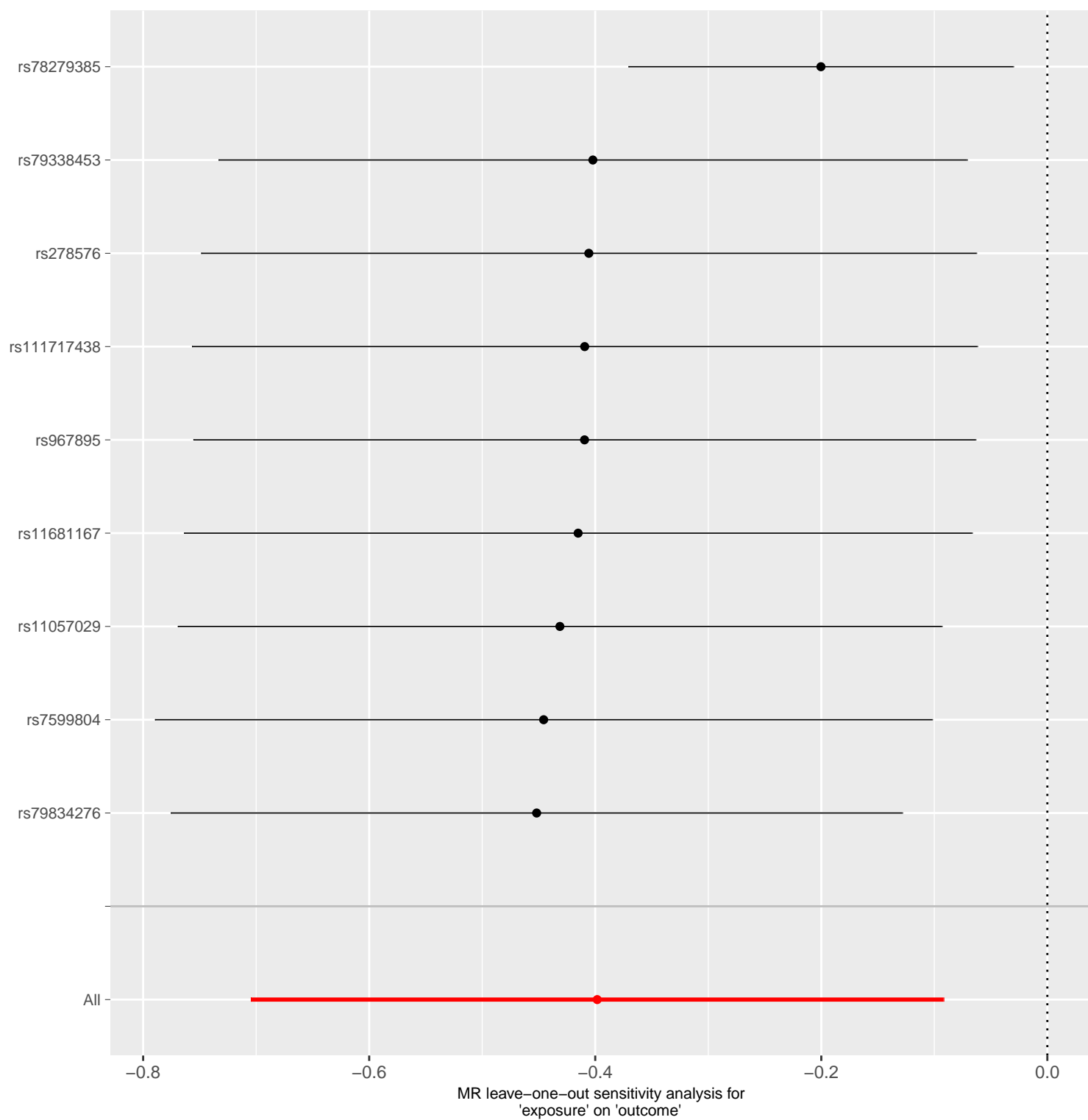

Supplement: Supplementary Data Sheet 2 — Full GCST identifiers, taxonomic labels, and Mendelian randomization statistics for the gut microbial traits associated with ulcerative colitis. [file DataSheet2.zip › GM_result/GCST90032262/sensitivity-analysis.pdf]

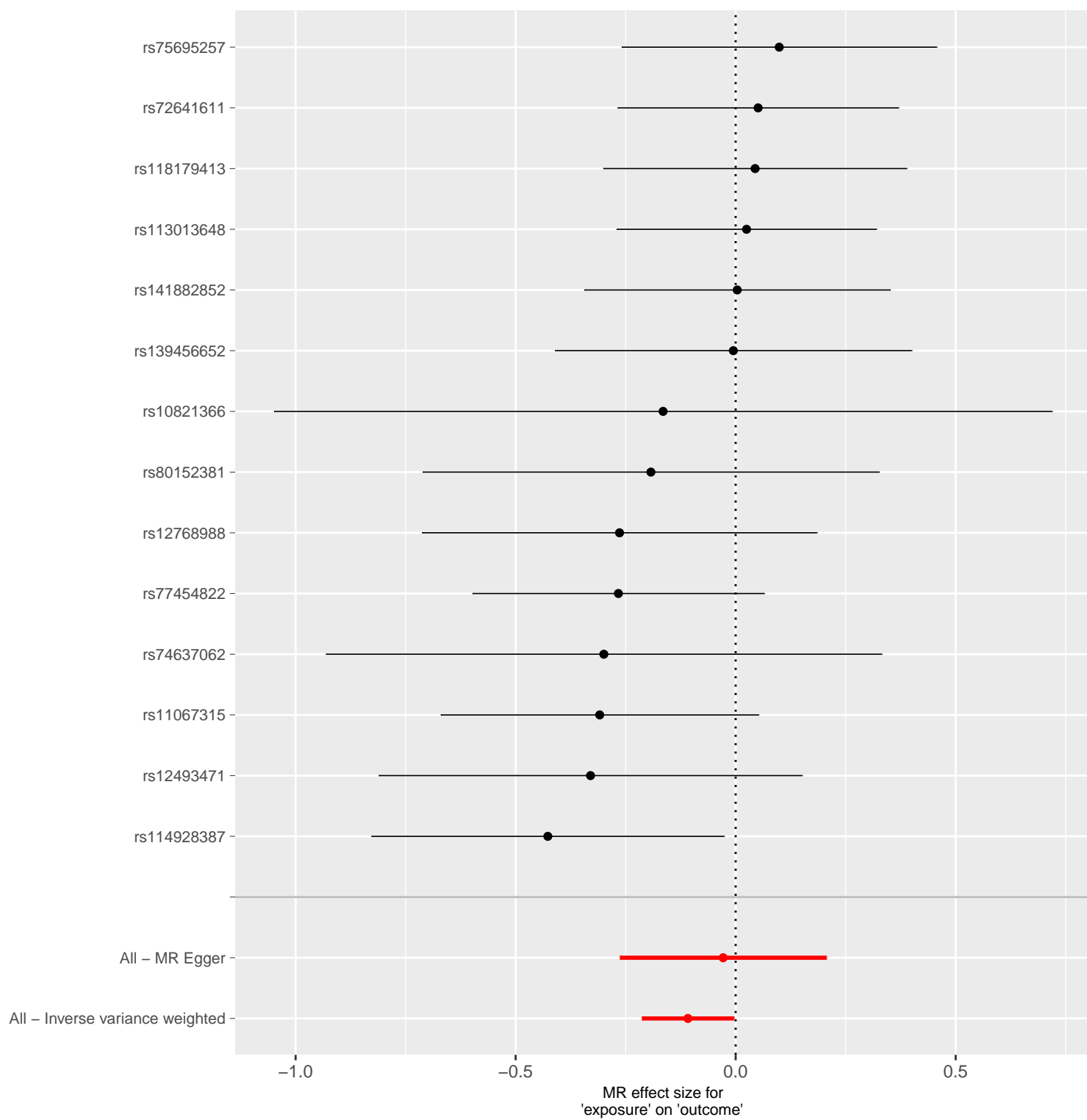

Supplement: Supplementary Data Sheet 2 — Full GCST identifiers, taxonomic labels, and Mendelian randomization statistics for the gut microbial traits associated with ulcerative colitis. [file DataSheet2.zip › GM_result/GCST90032290/forest.pdf]
