# Supplementary figures and images for: Integrative Mendelian randomization and experimental validation prioritize KLF4 in the gut microbiota–pyroptosis–barrier axis of ulcerative colitis (part 2 of 3)
Source: Front Immunol. 2026 Mar 16;17:1773990. doi: 10.3389/fimmu.2026.1773990 (PMC13033518; doi:10.3389/fimmu.2026.1773990)

# MR Method

- Inverse variance weighted
- MR Egger

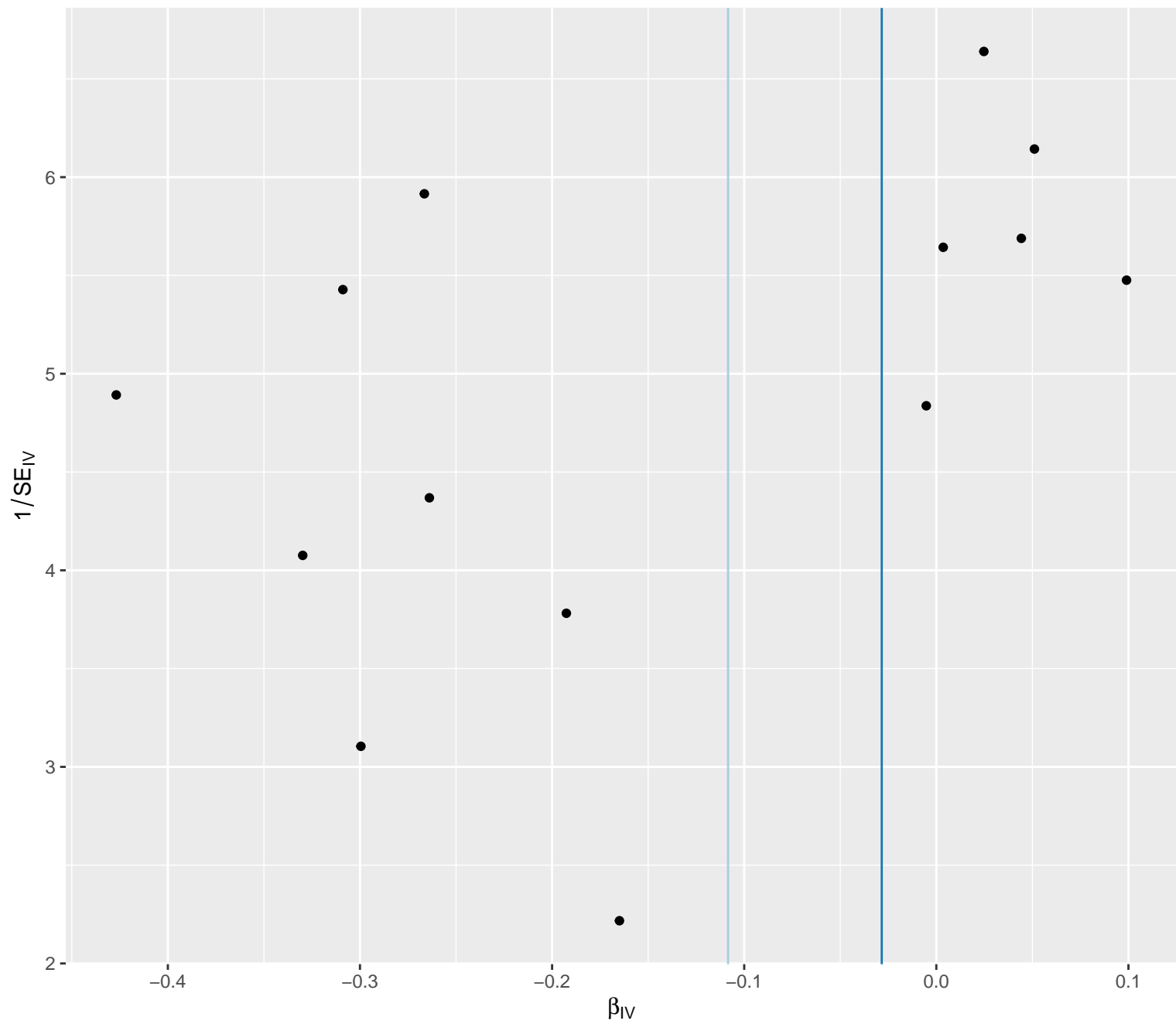

Supplement: Supplementary Data Sheet 2 — Full GCST identifiers, taxonomic labels, and Mendelian randomization statistics for the gut microbial traits associated with ulcerative colitis. [file DataSheet2.zip › GM_result/GCST90032290/funnelplot.pdf]

# MR Test

- Inverse variance weighted
- MR Egger
- Simple mode
- Weighted median
- Weighted mode

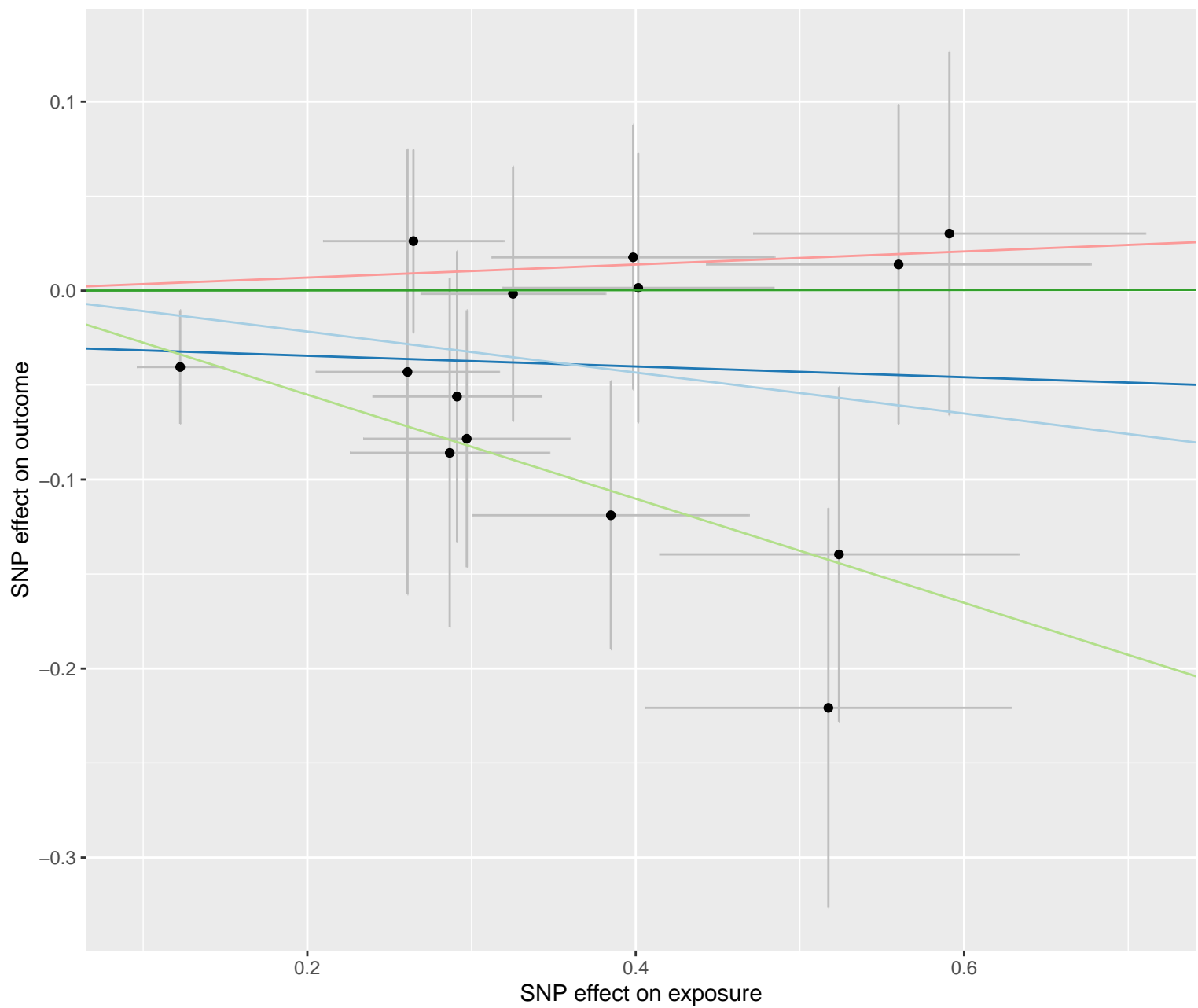

Supplement: Supplementary Data Sheet 2 — Full GCST identifiers, taxonomic labels, and Mendelian randomization statistics for the gut microbial traits associated with ulcerative colitis. [file DataSheet2.zip › GM_result/GCST90032290/scatter.pdf]

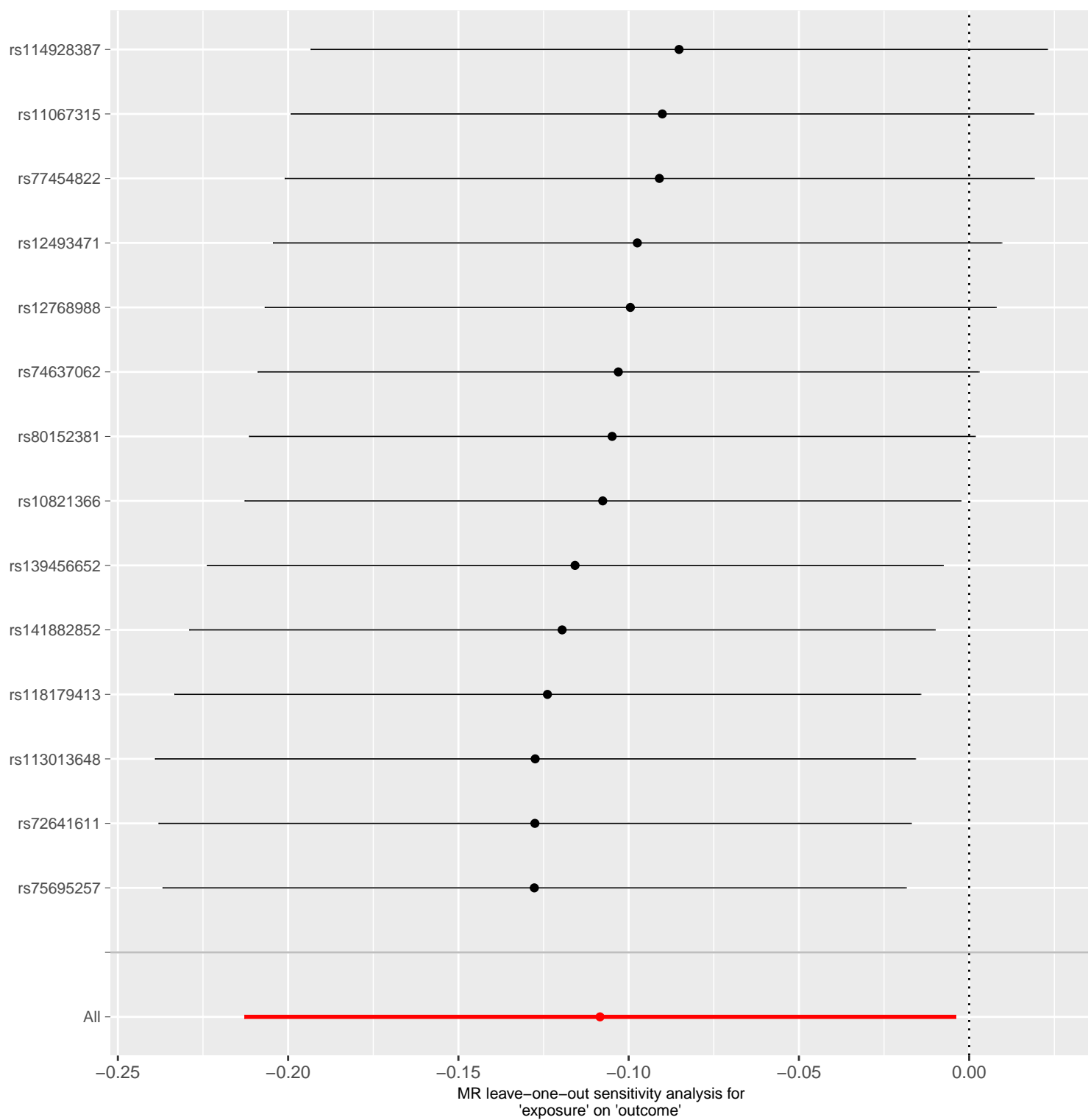

Supplement: Supplementary Data Sheet 2 — Full GCST identifiers, taxonomic labels, and Mendelian randomization statistics for the gut microbial traits associated with ulcerative colitis. [file DataSheet2.zip › GM_result/GCST90032290/sensitivity-analysis.pdf]

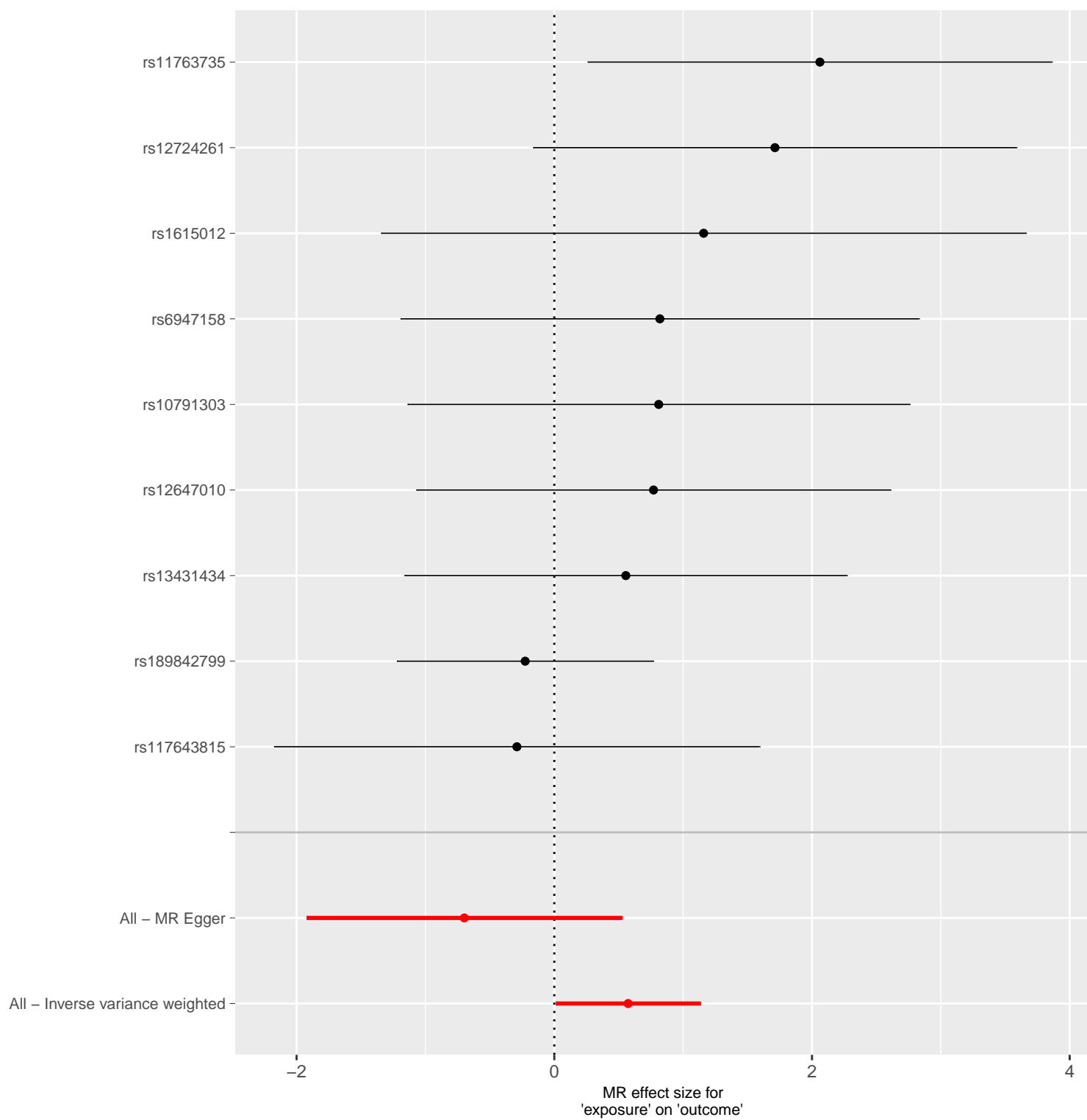

Supplement: Supplementary Data Sheet 2 — Full GCST identifiers, taxonomic labels, and Mendelian randomization statistics for the gut microbial traits associated with ulcerative colitis. [file DataSheet2.zip › GM_result/GCST90032313/forest.pdf]

# MR Method

- Inverse variance weighted
- MR Egger

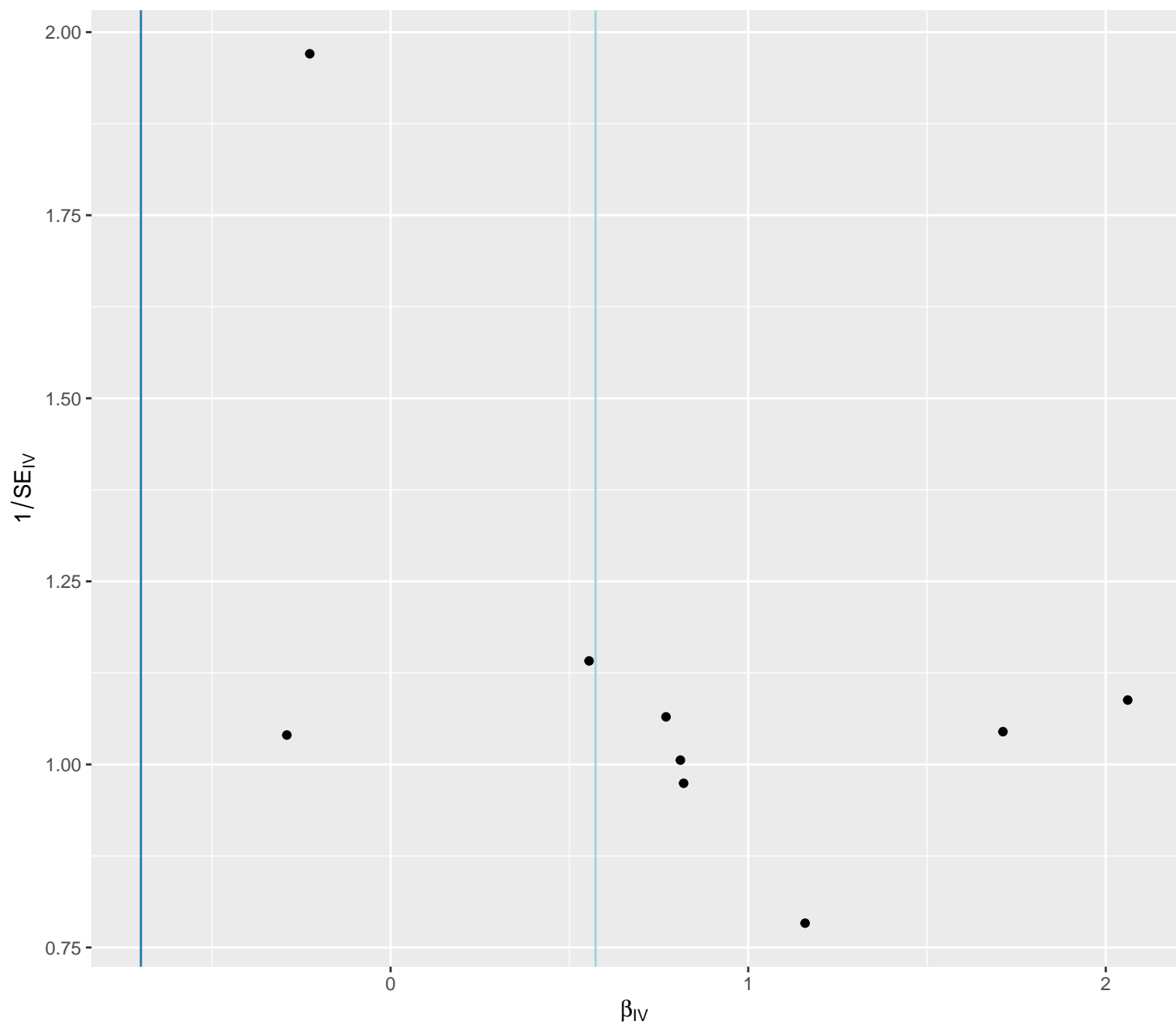

Supplement: Supplementary Data Sheet 2 — Full GCST identifiers, taxonomic labels, and Mendelian randomization statistics for the gut microbial traits associated with ulcerative colitis. [file DataSheet2.zip › GM_result/GCST90032313/funnelplot.pdf]

# MR Test

- Inverse variance weighted
- MR Egger
- Simple mode
- Weighted median
- Weighted mode

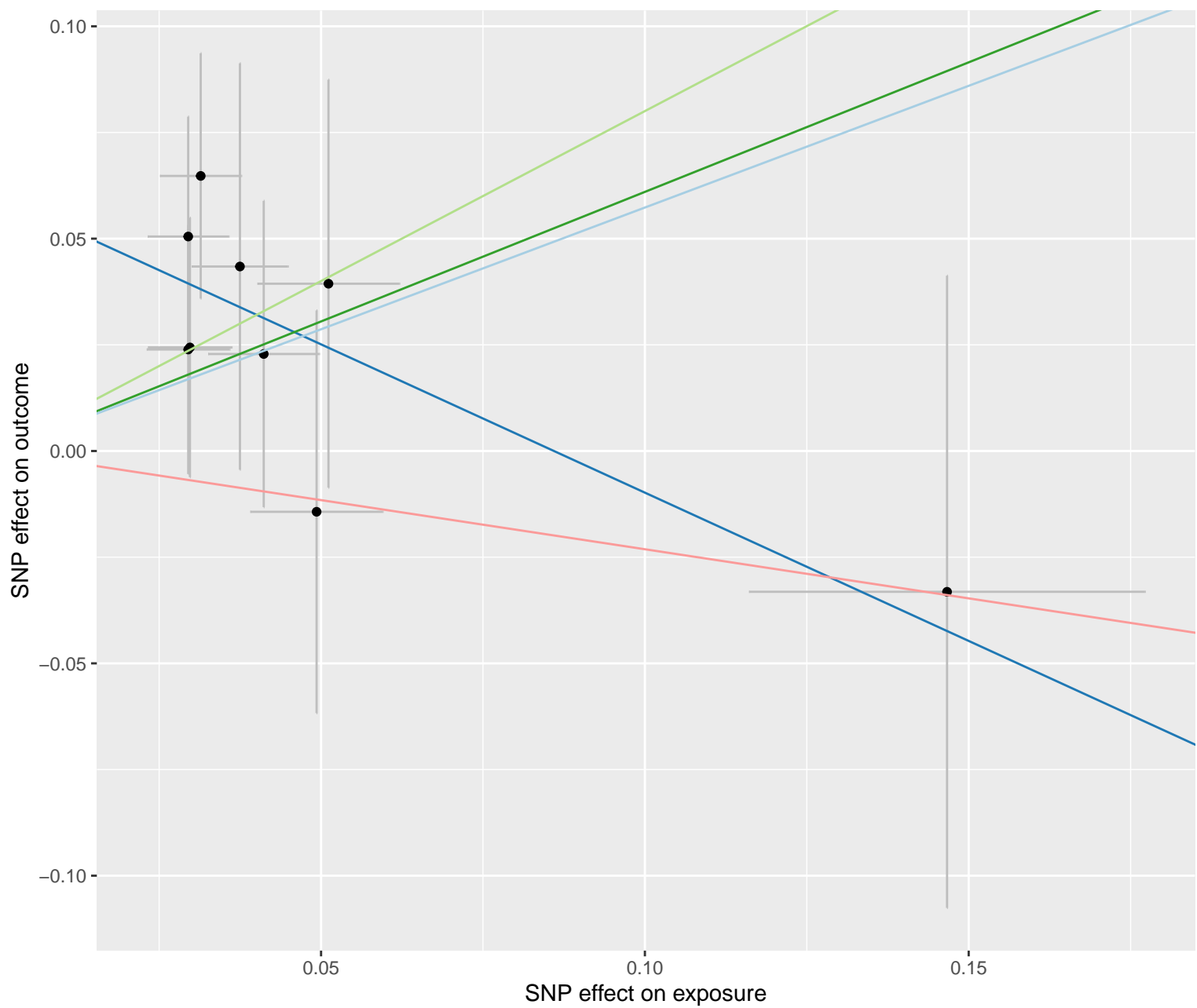

Supplement: Supplementary Data Sheet 2 — Full GCST identifiers, taxonomic labels, and Mendelian randomization statistics for the gut microbial traits associated with ulcerative colitis. [file DataSheet2.zip › GM_result/GCST90032313/scatter.pdf]

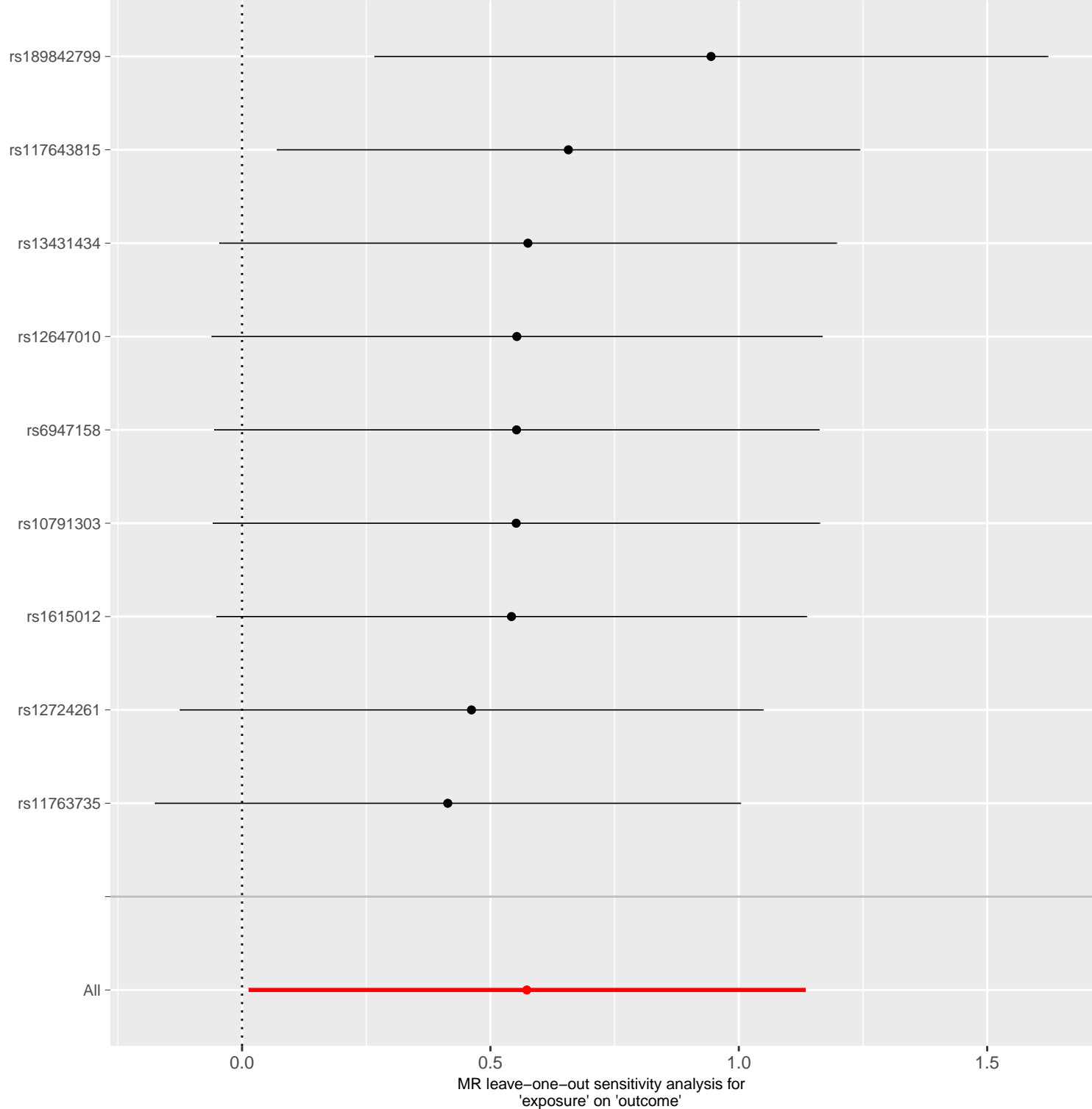

Supplement: Supplementary Data Sheet 2 — Full GCST identifiers, taxonomic labels, and Mendelian randomization statistics for the gut microbial traits associated with ulcerative colitis. [file DataSheet2.zip › GM_result/GCST90032313/sensitivity-analysis.pdf]

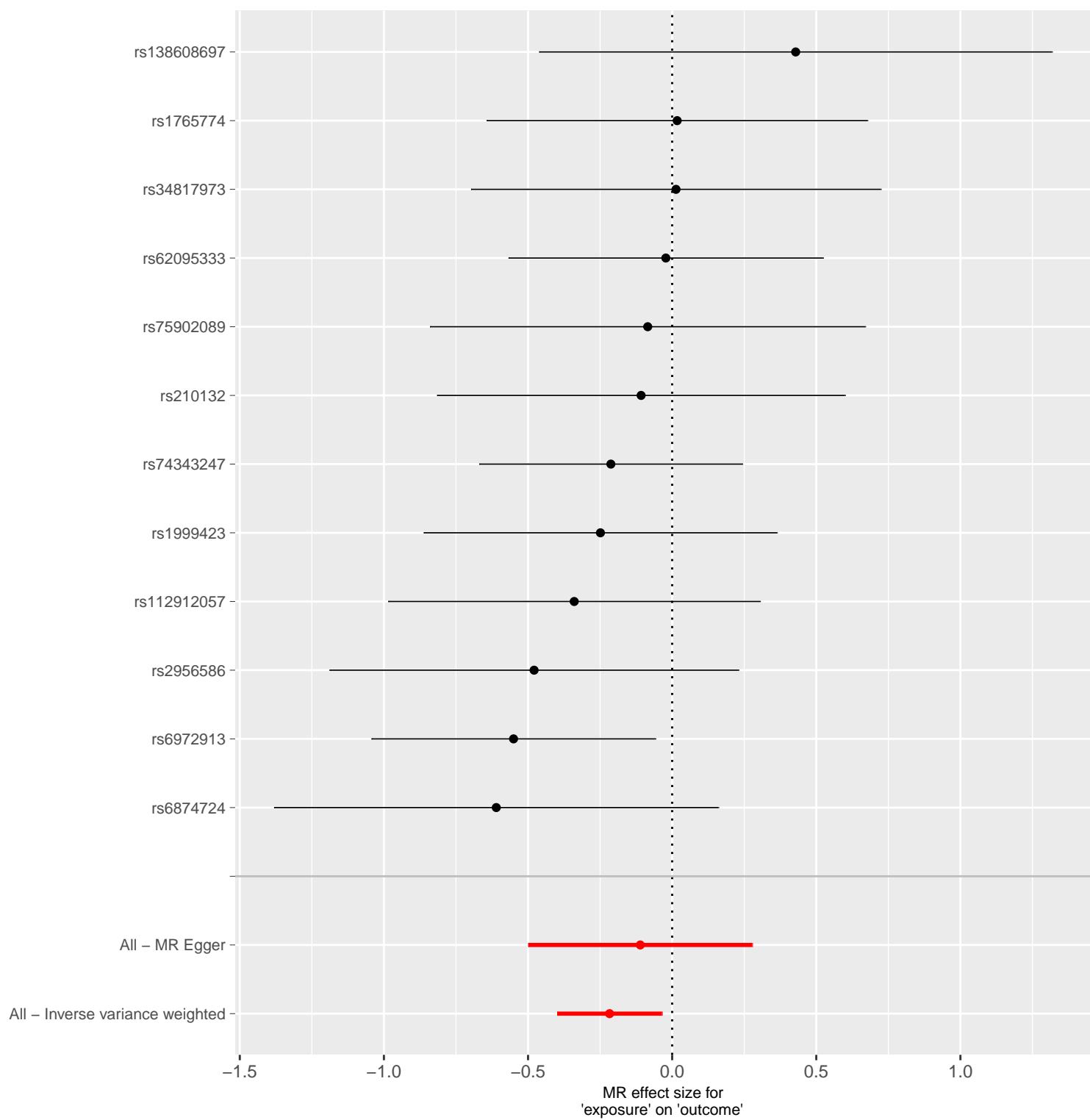

Supplement: Supplementary Data Sheet 2 — Full GCST identifiers, taxonomic labels, and Mendelian randomization statistics for the gut microbial traits associated with ulcerative colitis. [file DataSheet2.zip › GM_result/GCST90032344/forest.pdf]

# MR Method

- Inverse variance weighted
- MR Egger

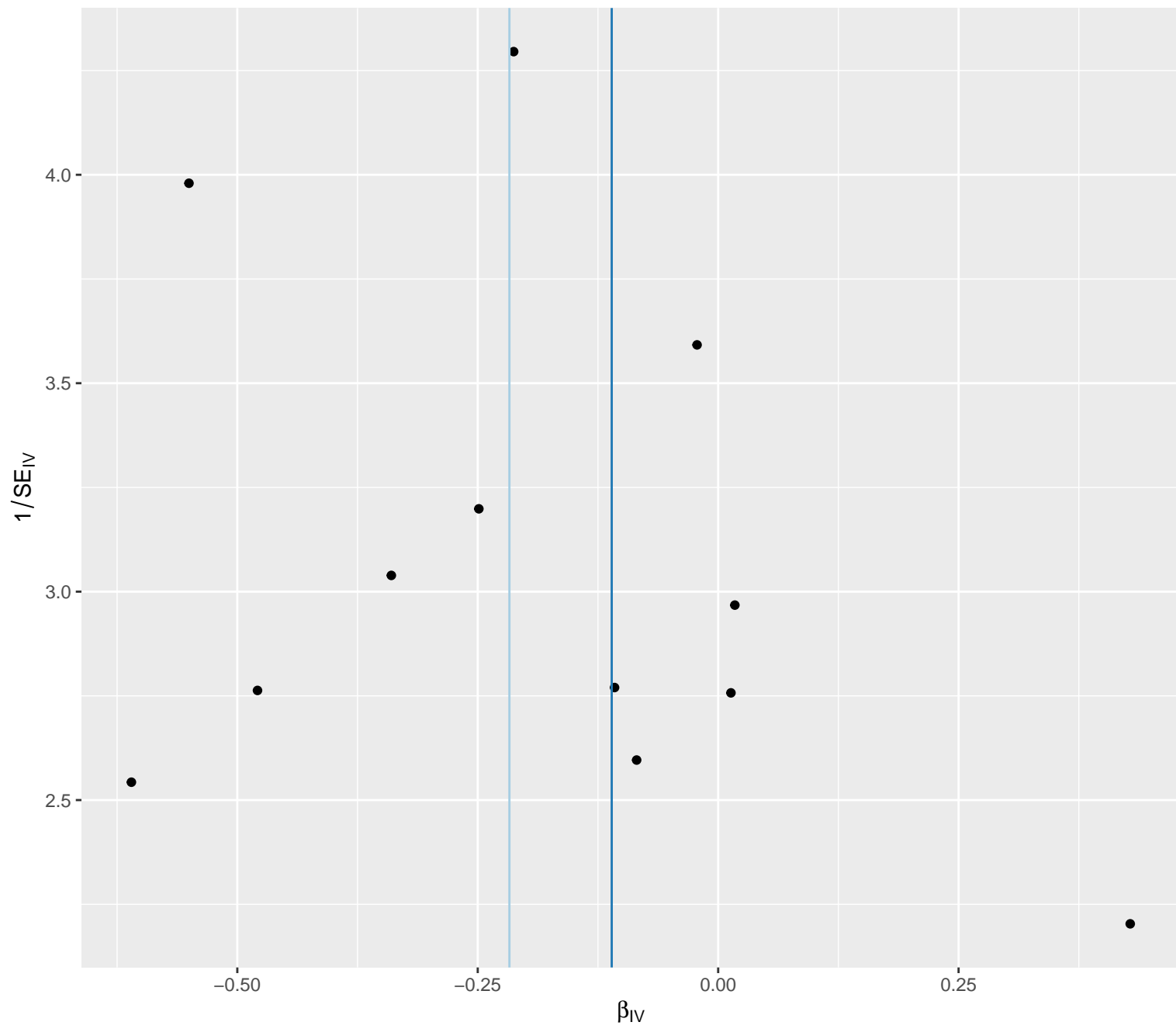

Supplement: Supplementary Data Sheet 2 — Full GCST identifiers, taxonomic labels, and Mendelian randomization statistics for the gut microbial traits associated with ulcerative colitis. [file DataSheet2.zip › GM_result/GCST90032344/funnelplot.pdf]

# MR Test

- Inverse variance weighted
- MR Egger
- Simple mode
- Weighted median
- Weighted mode

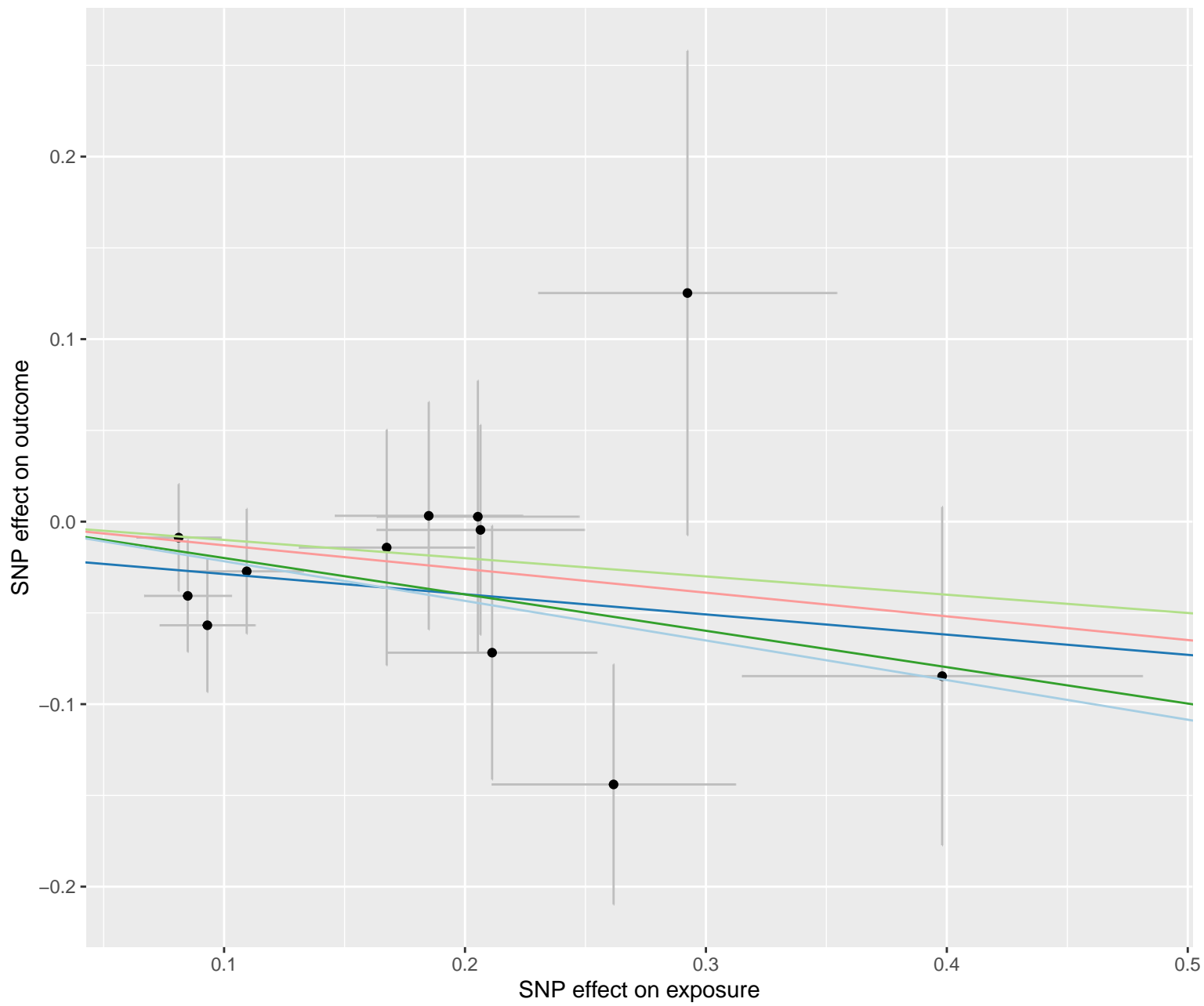

Supplement: Supplementary Data Sheet 2 — Full GCST identifiers, taxonomic labels, and Mendelian randomization statistics for the gut microbial traits associated with ulcerative colitis. [file DataSheet2.zip › GM_result/GCST90032344/scatter.pdf]

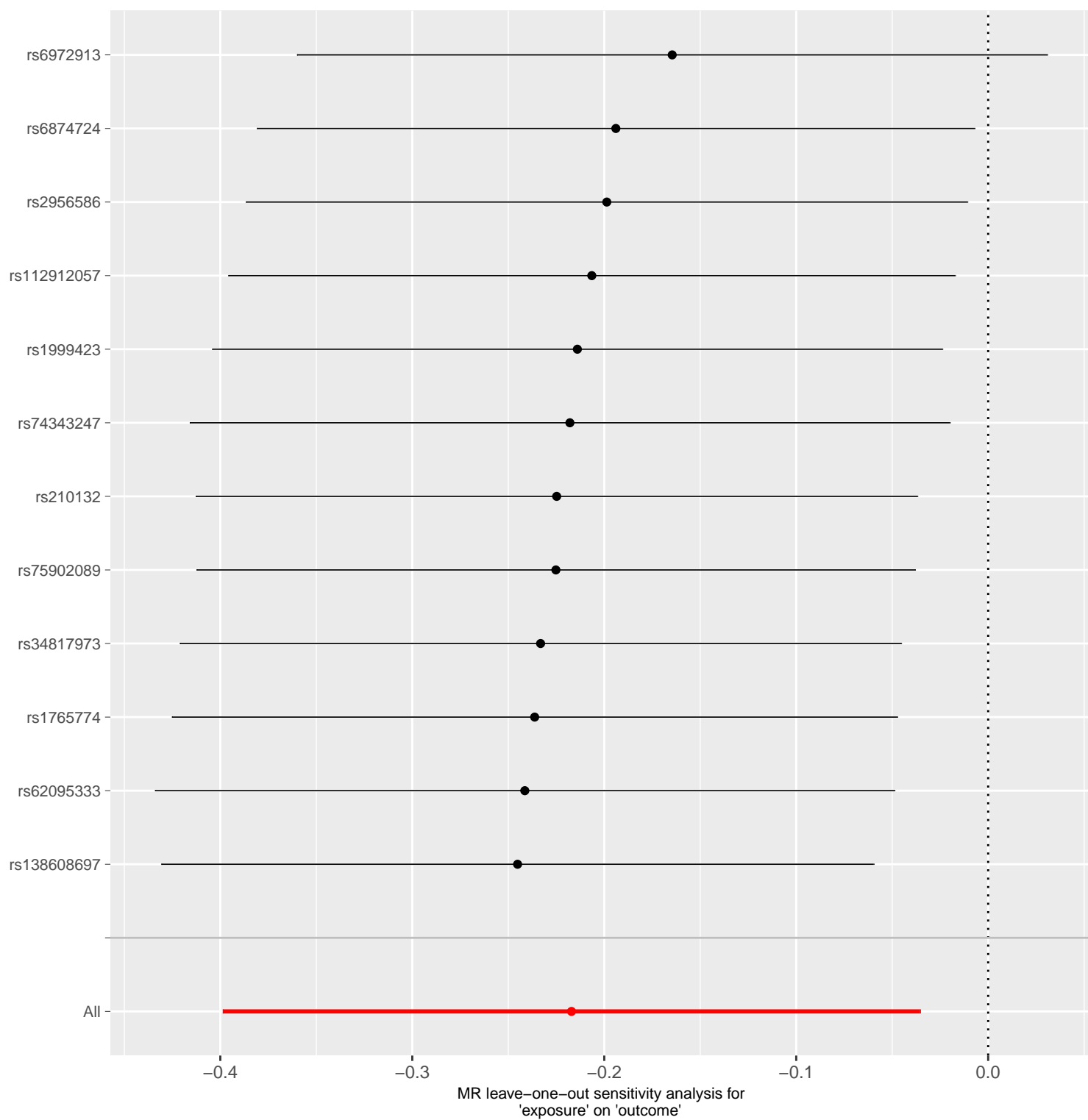

Supplement: Supplementary Data Sheet 2 — Full GCST identifiers, taxonomic labels, and Mendelian randomization statistics for the gut microbial traits associated with ulcerative colitis. [file DataSheet2.zip › GM_result/GCST90032344/sensitivity-analysis.pdf]

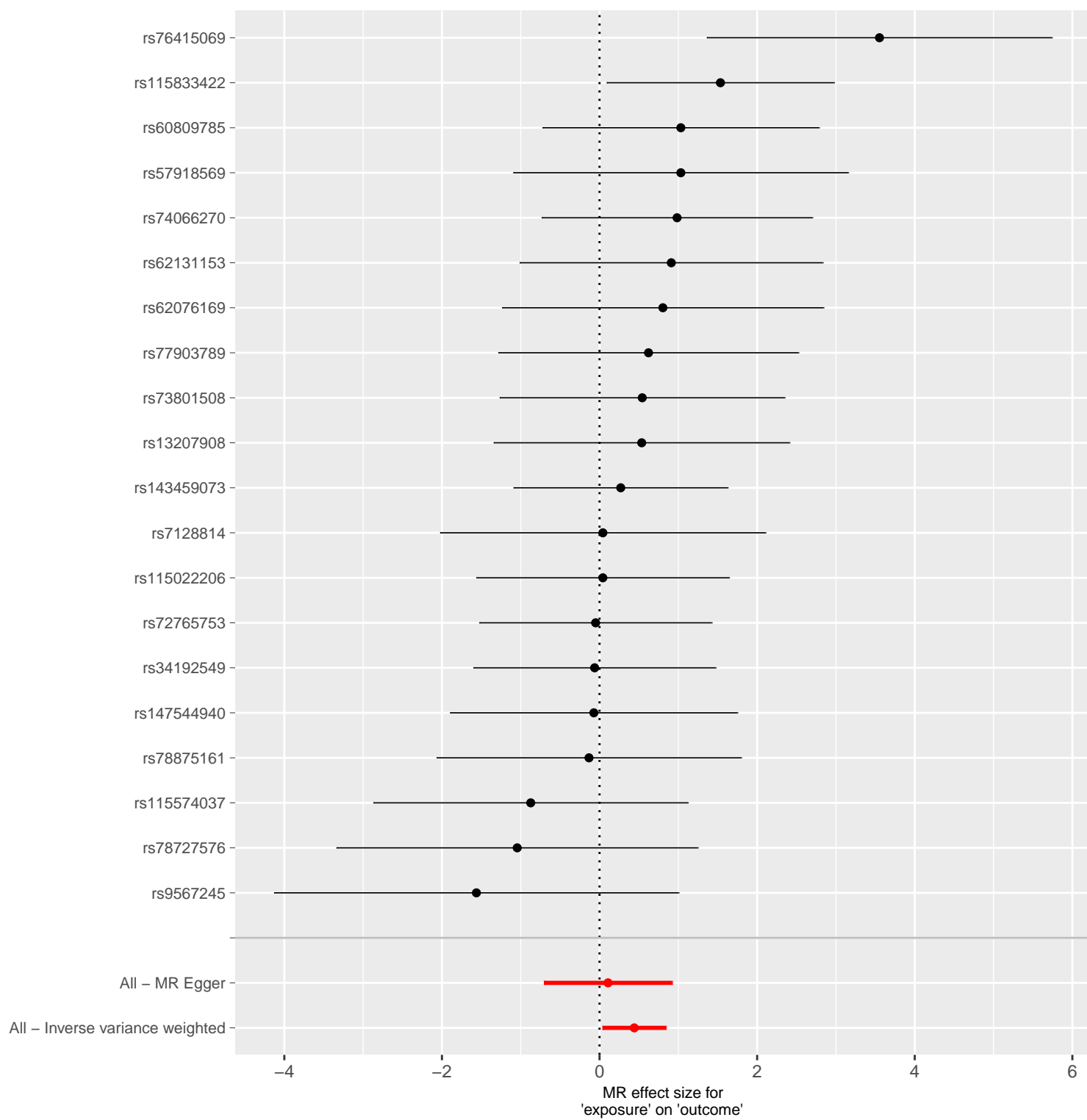

Supplement: Supplementary Data Sheet 2 — Full GCST identifiers, taxonomic labels, and Mendelian randomization statistics for the gut microbial traits associated with ulcerative colitis. [file DataSheet2.zip › GM_result/GCST90032362/forest.pdf]

# MR Method

- Inverse variance weighted
- MR Egger

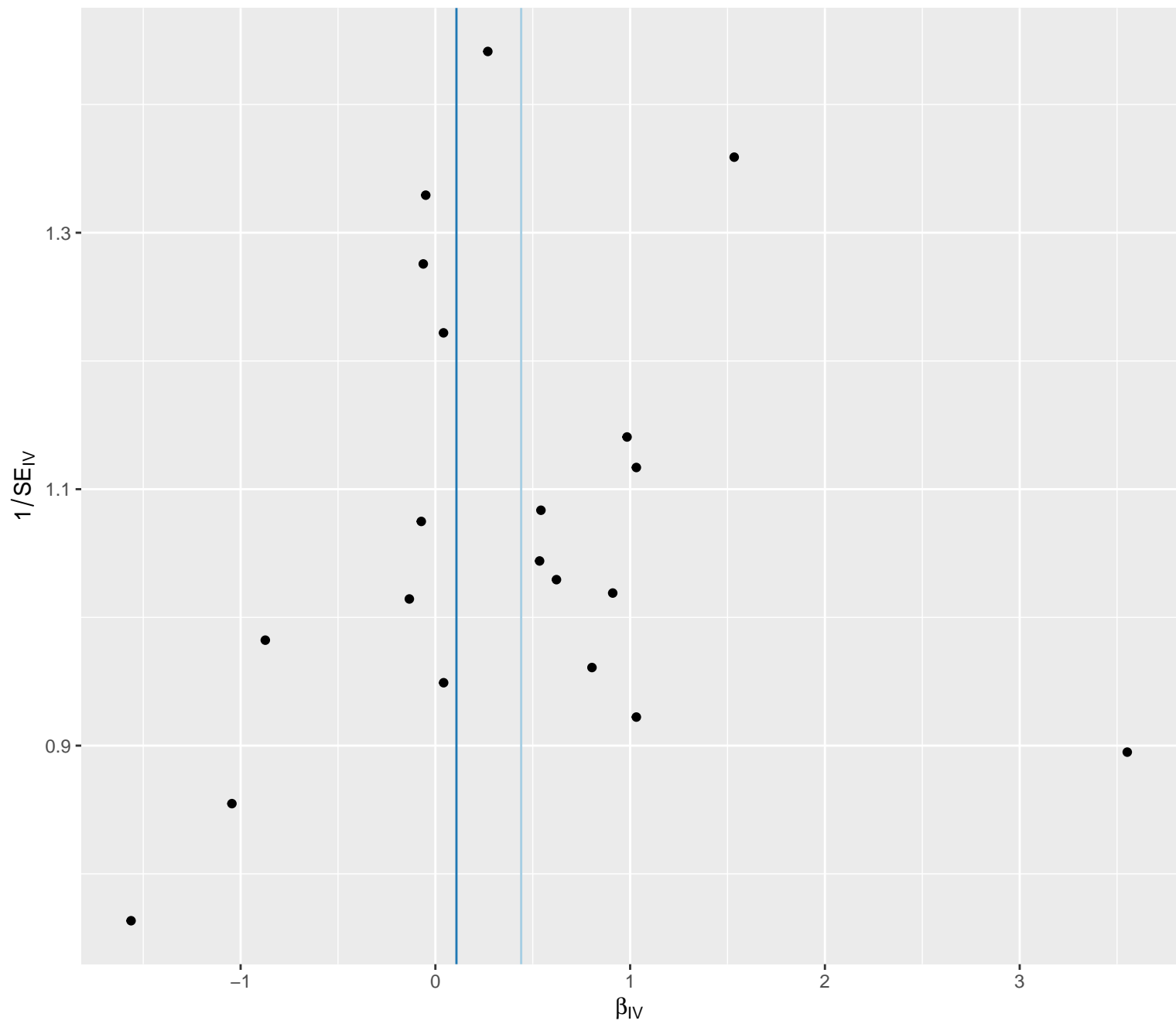

Supplement: Supplementary Data Sheet 2 — Full GCST identifiers, taxonomic labels, and Mendelian randomization statistics for the gut microbial traits associated with ulcerative colitis. [file DataSheet2.zip › GM_result/GCST90032362/funnelplot.pdf]

# MR Test

- Inverse variance weighted
- MR Egger
- Simple mode
- Weighted median
- Weighted mode

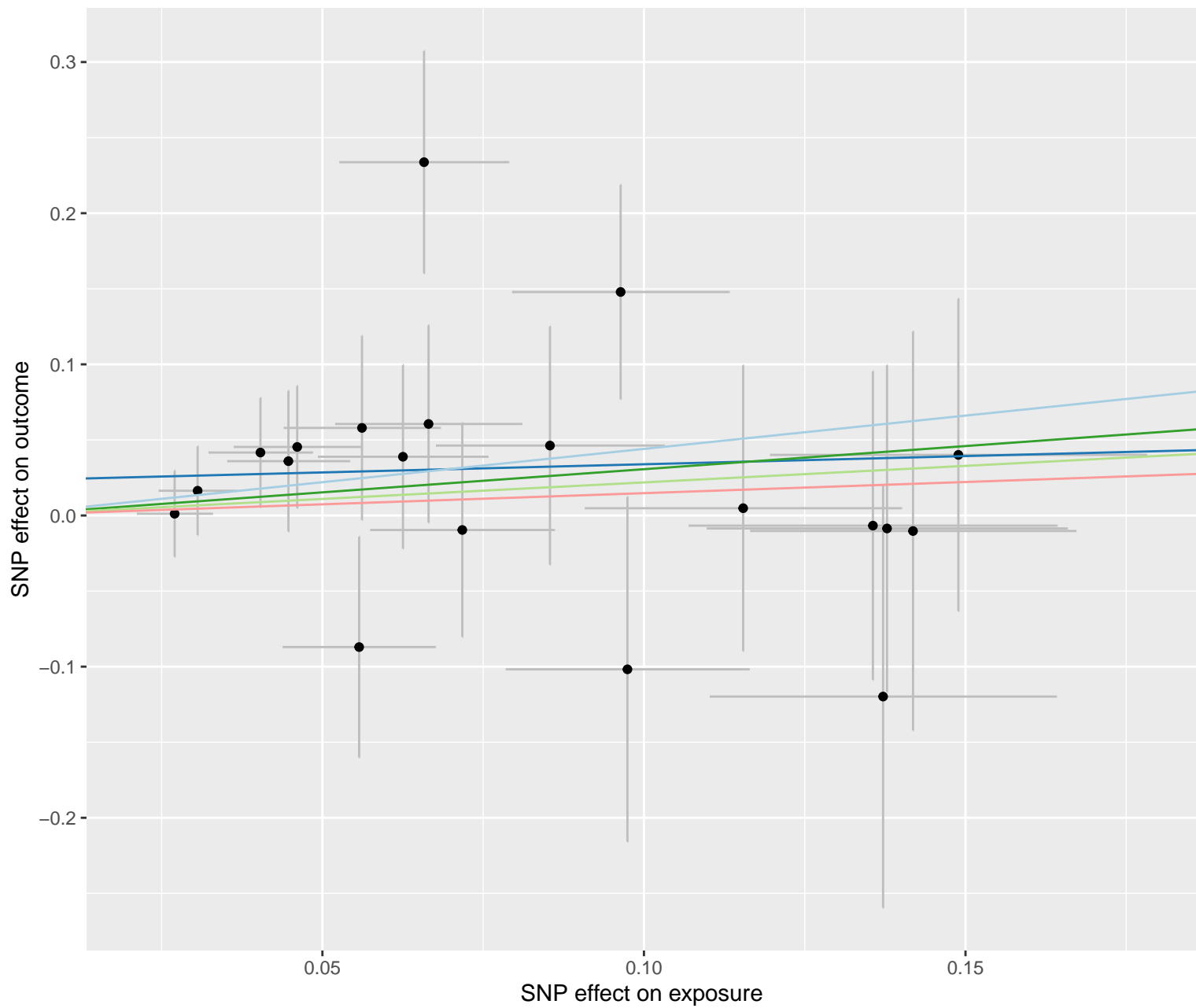

Supplement: Supplementary Data Sheet 2 — Full GCST identifiers, taxonomic labels, and Mendelian randomization statistics for the gut microbial traits associated with ulcerative colitis. [file DataSheet2.zip › GM_result/GCST90032362/scatter.pdf]

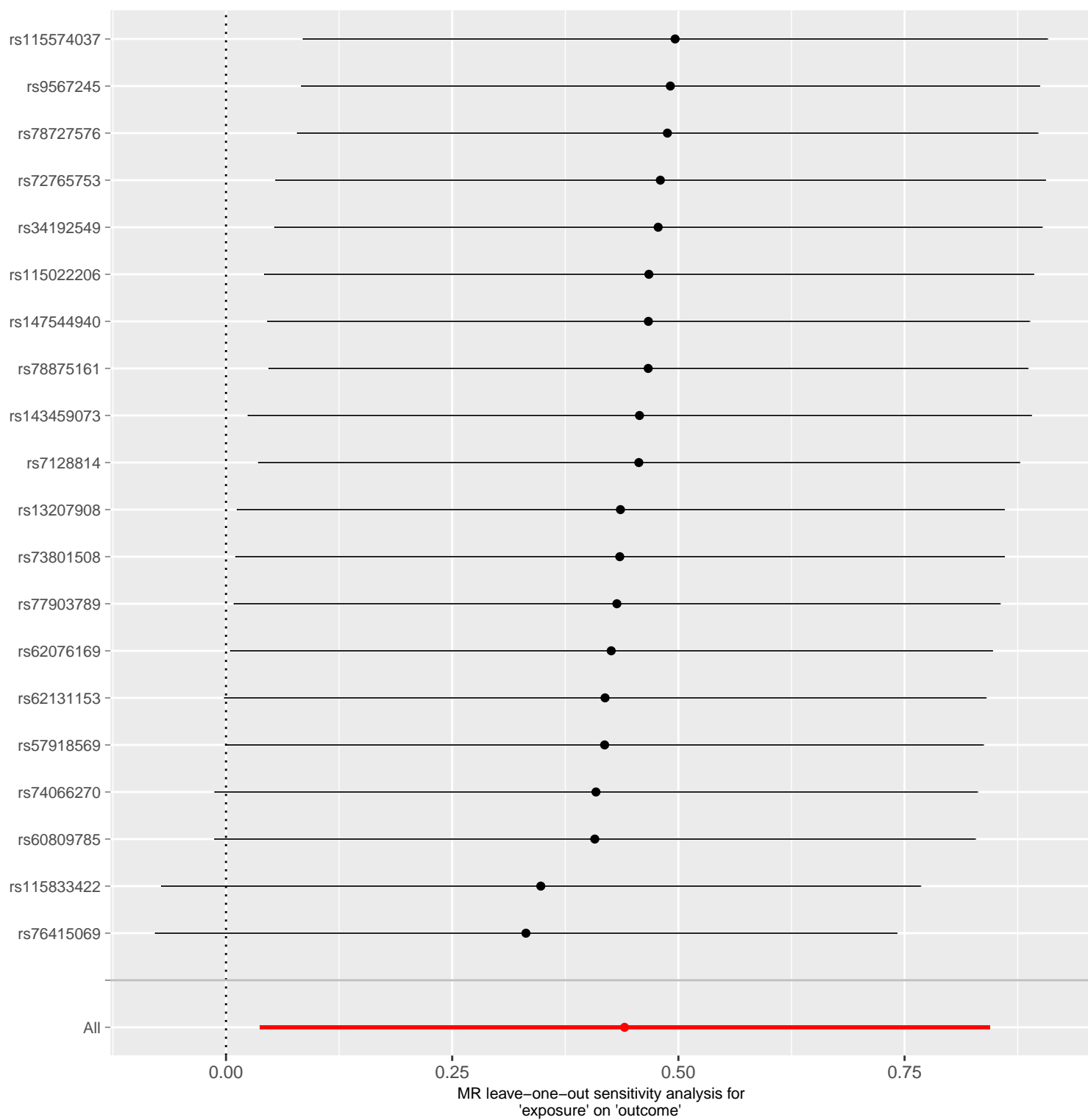

Supplement: Supplementary Data Sheet 2 — Full GCST identifiers, taxonomic labels, and Mendelian randomization statistics for the gut microbial traits associated with ulcerative colitis. [file DataSheet2.zip › GM_result/GCST90032362/sensitivity-analysis.pdf]

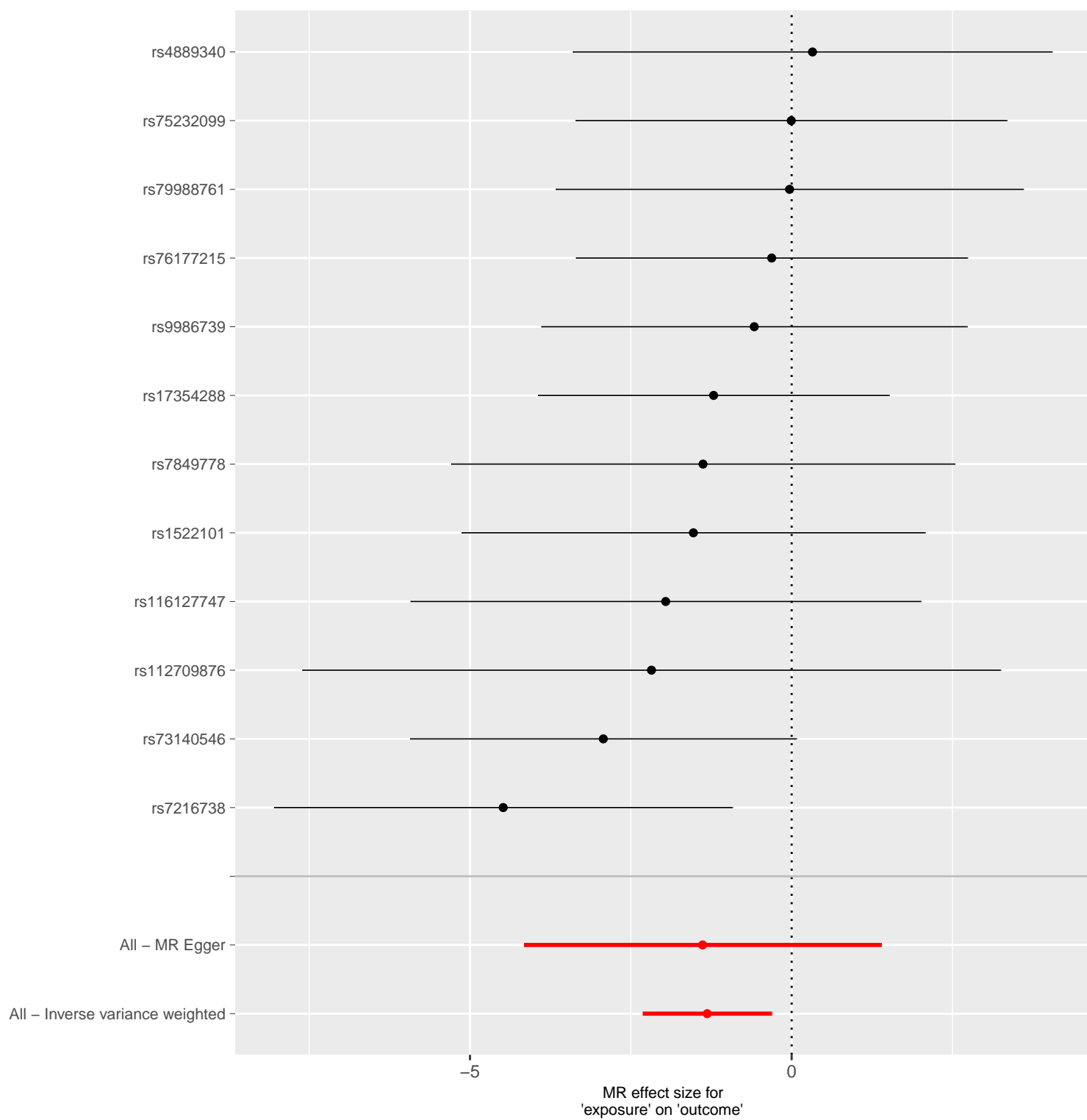

Supplement: Supplementary Data Sheet 2 — Full GCST identifiers, taxonomic labels, and Mendelian randomization statistics for the gut microbial traits associated with ulcerative colitis. [file DataSheet2.zip › GM_result/GCST90032367/forest.pdf]

# MR Method

- Inverse variance weighted
- MR Egger

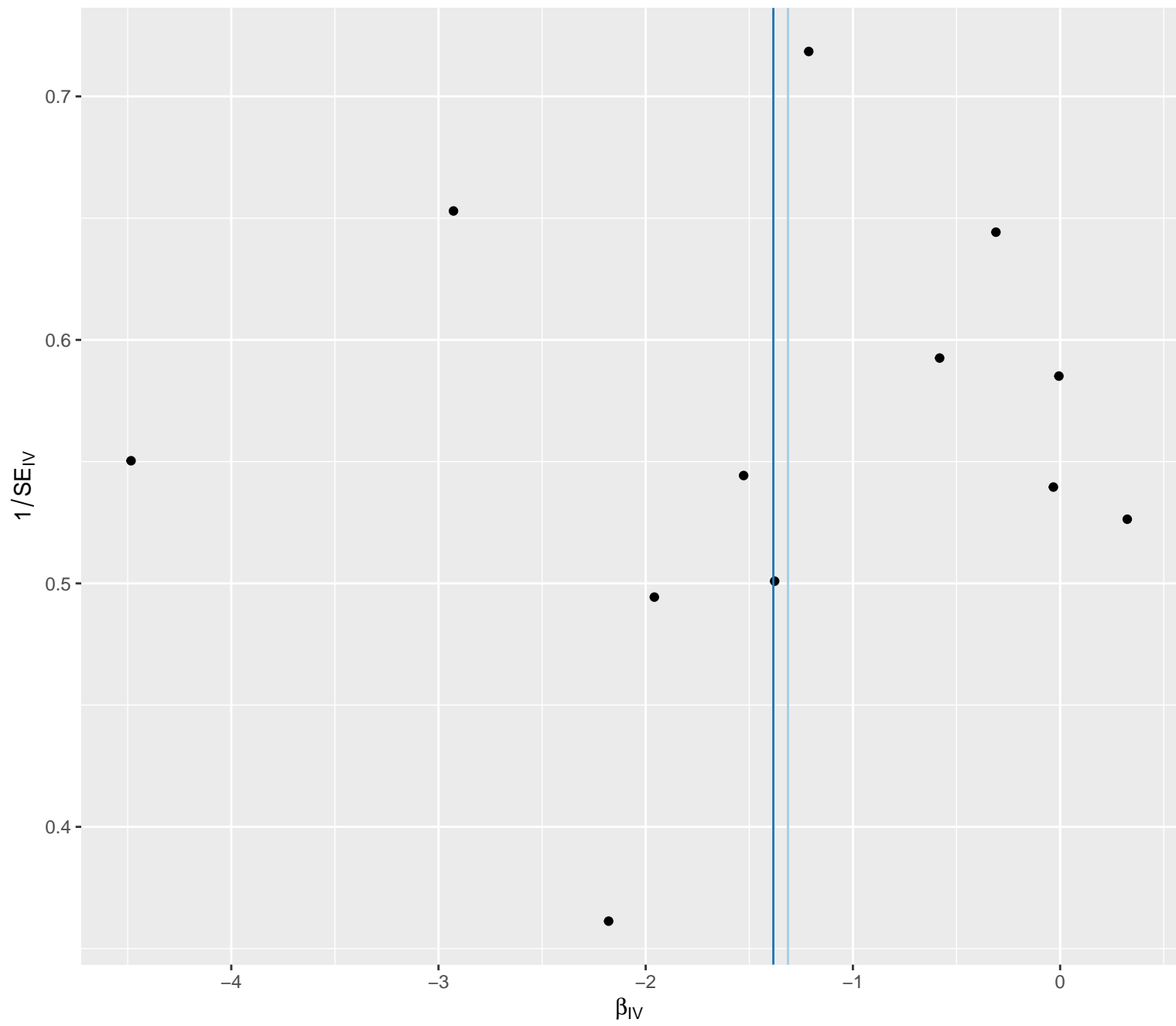

Supplement: Supplementary Data Sheet 2 — Full GCST identifiers, taxonomic labels, and Mendelian randomization statistics for the gut microbial traits associated with ulcerative colitis. [file DataSheet2.zip › GM_result/GCST90032367/funnelplot.pdf]

# MR Test

- Inverse variance weighted
- MR Egger
- Simple mode
- Weighted median
- Weighted mode

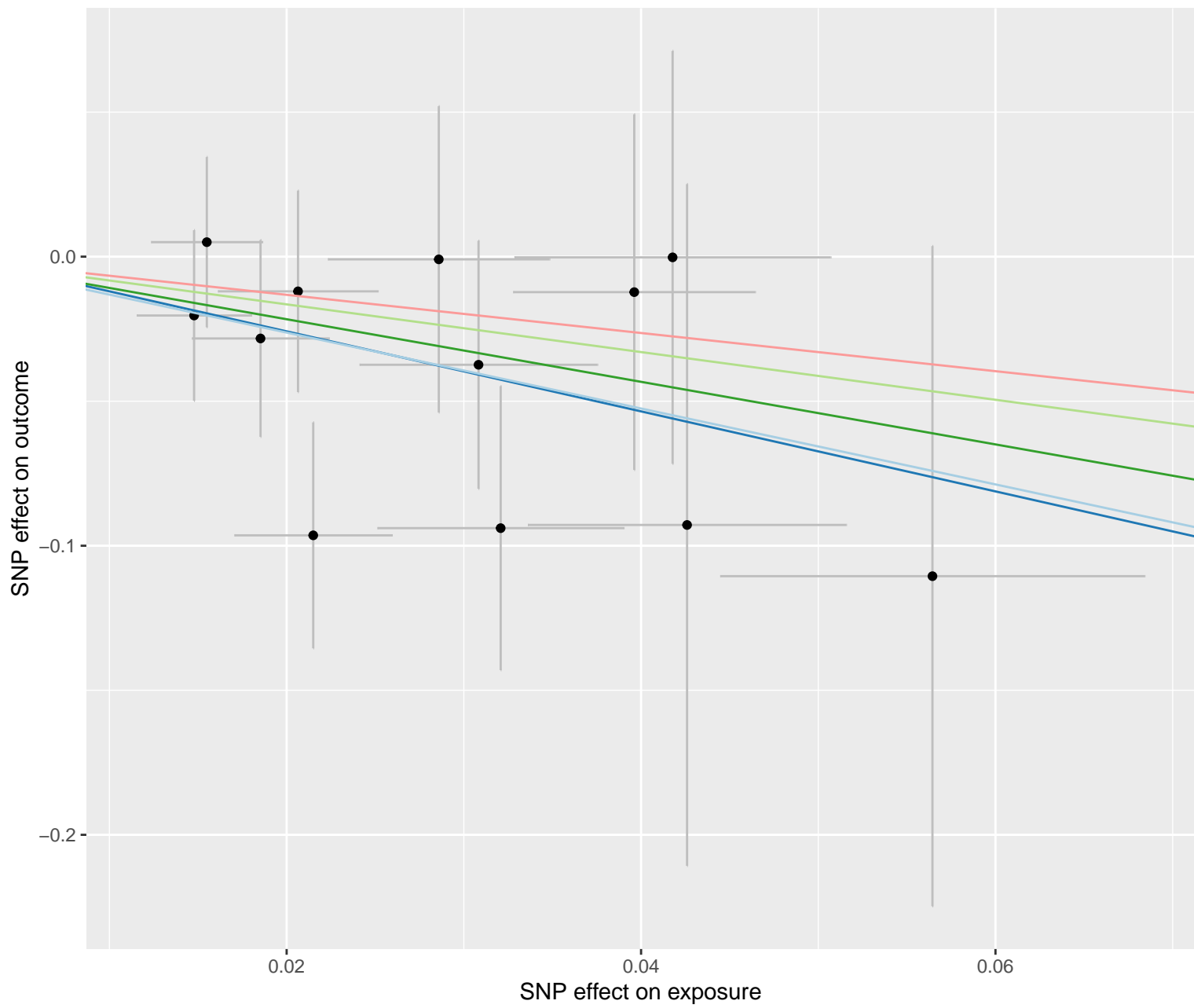

Supplement: Supplementary Data Sheet 2 — Full GCST identifiers, taxonomic labels, and Mendelian randomization statistics for the gut microbial traits associated with ulcerative colitis. [file DataSheet2.zip › GM_result/GCST90032367/scatter.pdf]

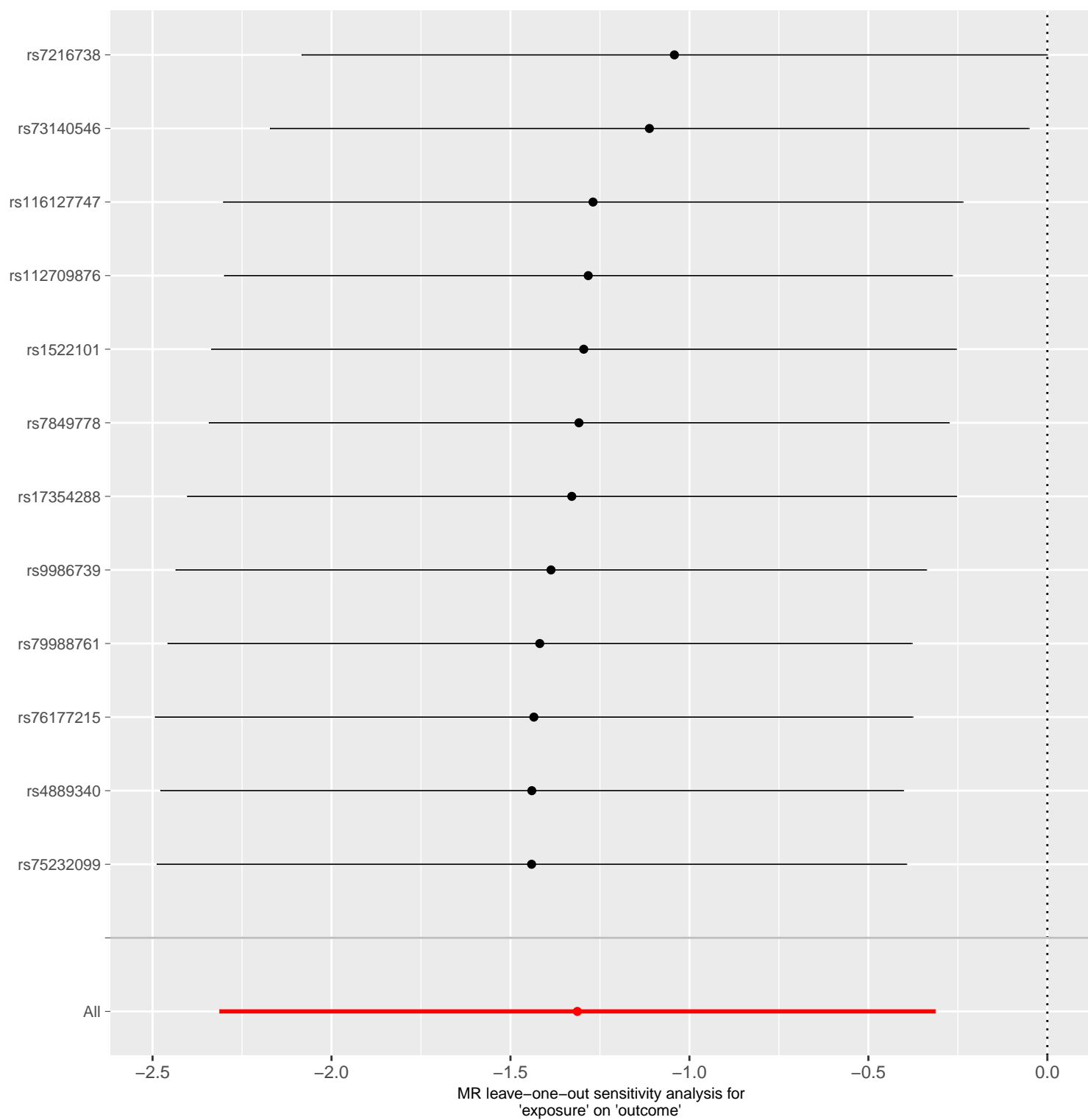

Supplement: Supplementary Data Sheet 2 — Full GCST identifiers, taxonomic labels, and Mendelian randomization statistics for the gut microbial traits associated with ulcerative colitis. [file DataSheet2.zip › GM_result/GCST90032367/sensitivity-analysis.pdf]

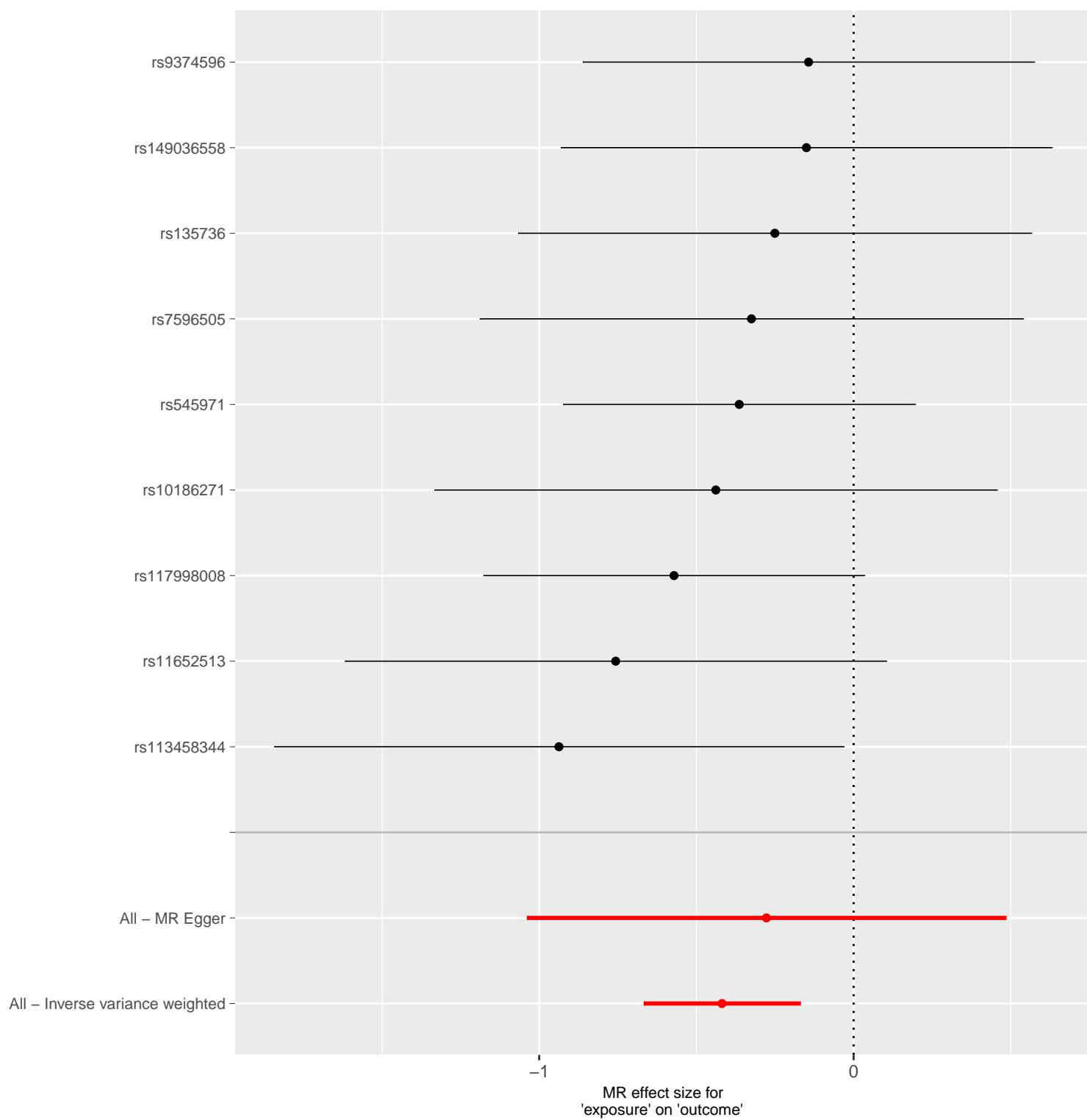

Supplement: Supplementary Data Sheet 2 — Full GCST identifiers, taxonomic labels, and Mendelian randomization statistics for the gut microbial traits associated with ulcerative colitis. [file DataSheet2.zip › GM_result/GCST90032382/forest.pdf]

# MR Method

- Inverse variance weighted
- MR Egger

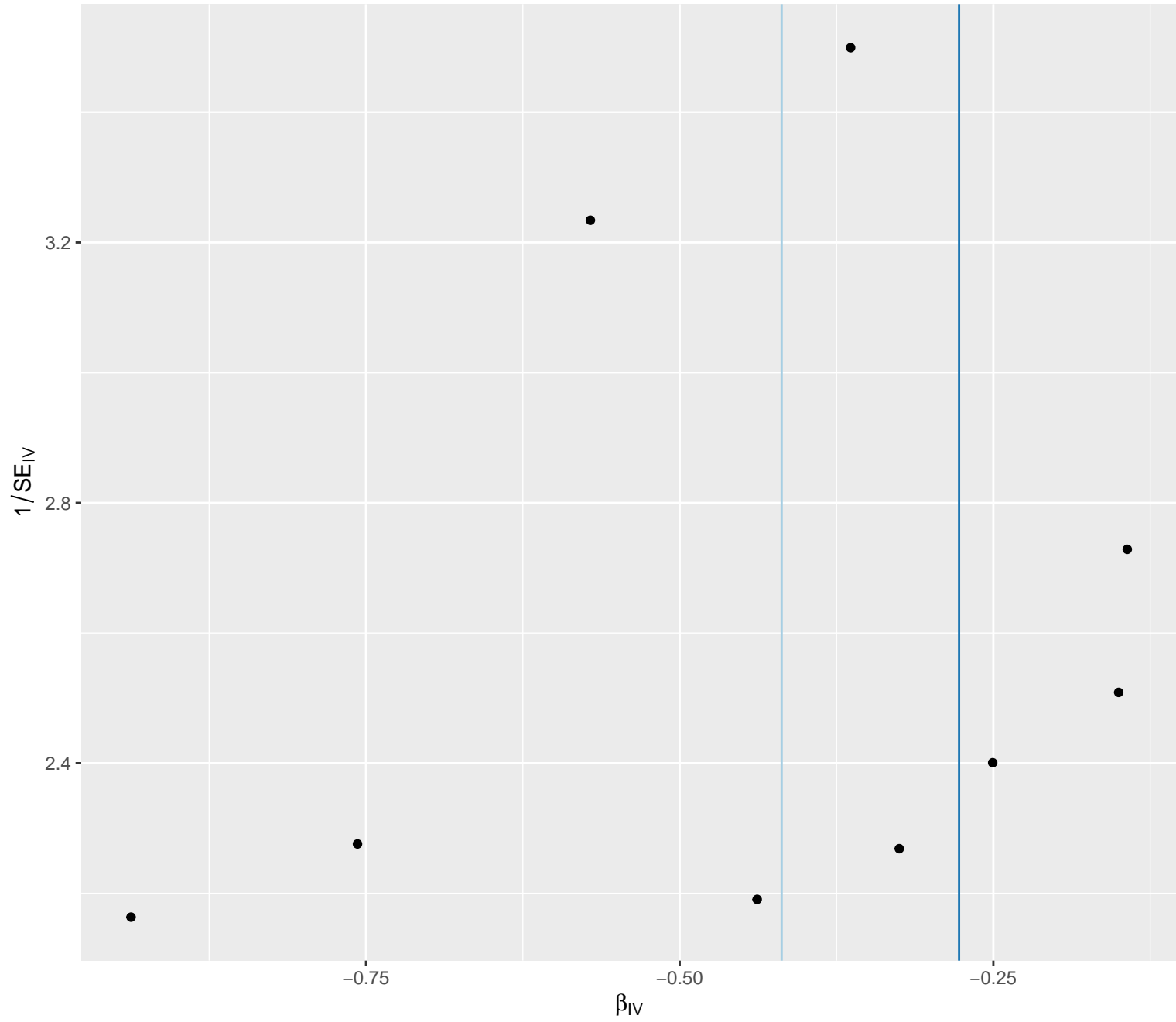

Supplement: Supplementary Data Sheet 2 — Full GCST identifiers, taxonomic labels, and Mendelian randomization statistics for the gut microbial traits associated with ulcerative colitis. [file DataSheet2.zip › GM_result/GCST90032382/funnelplot.pdf]

# MR Test

- Inverse variance weighted
- MR Egger
- Simple mode
- Weighted median
- Weighted mode

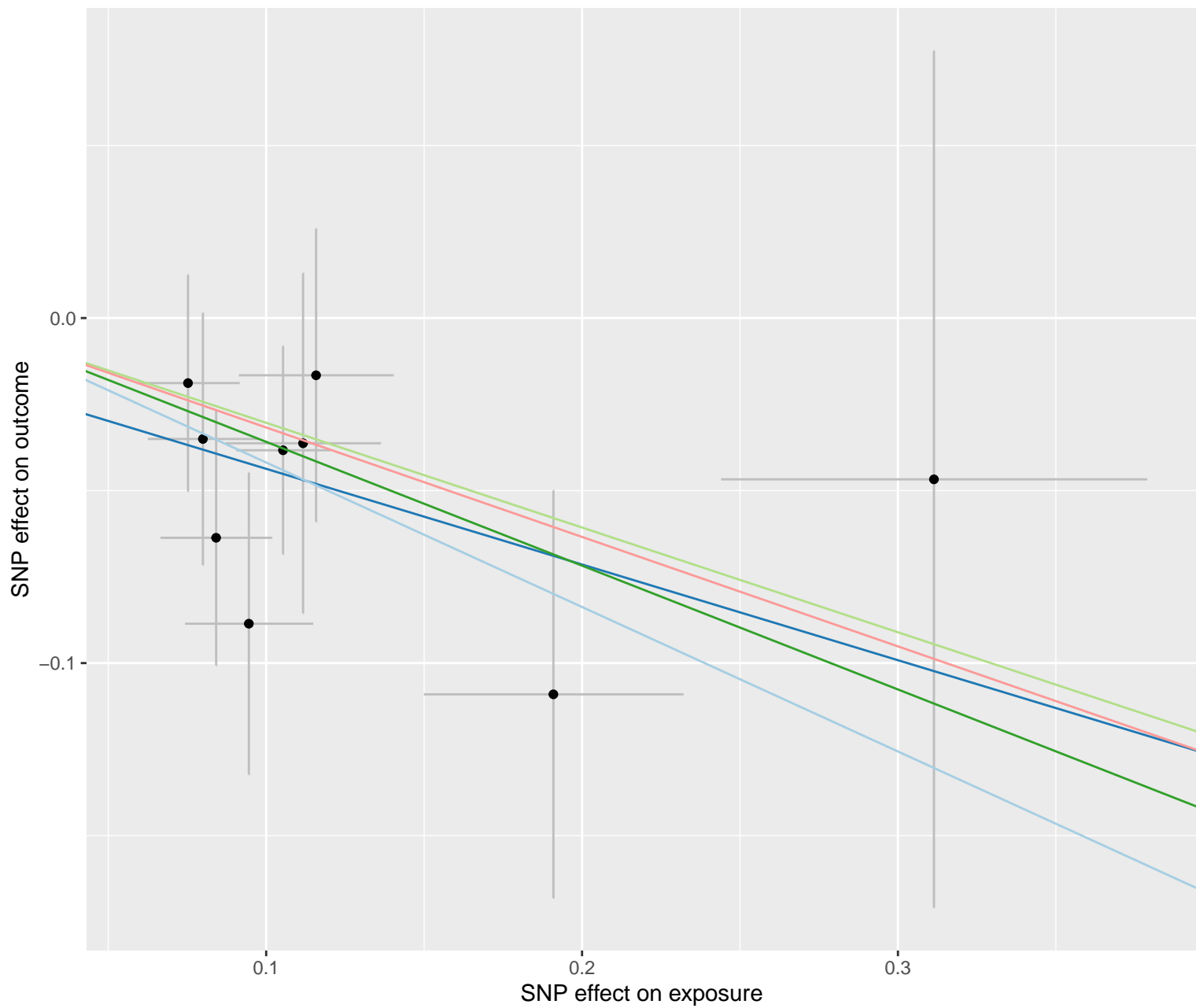

Supplement: Supplementary Data Sheet 2 — Full GCST identifiers, taxonomic labels, and Mendelian randomization statistics for the gut microbial traits associated with ulcerative colitis. [file DataSheet2.zip › GM_result/GCST90032382/scatter.pdf]

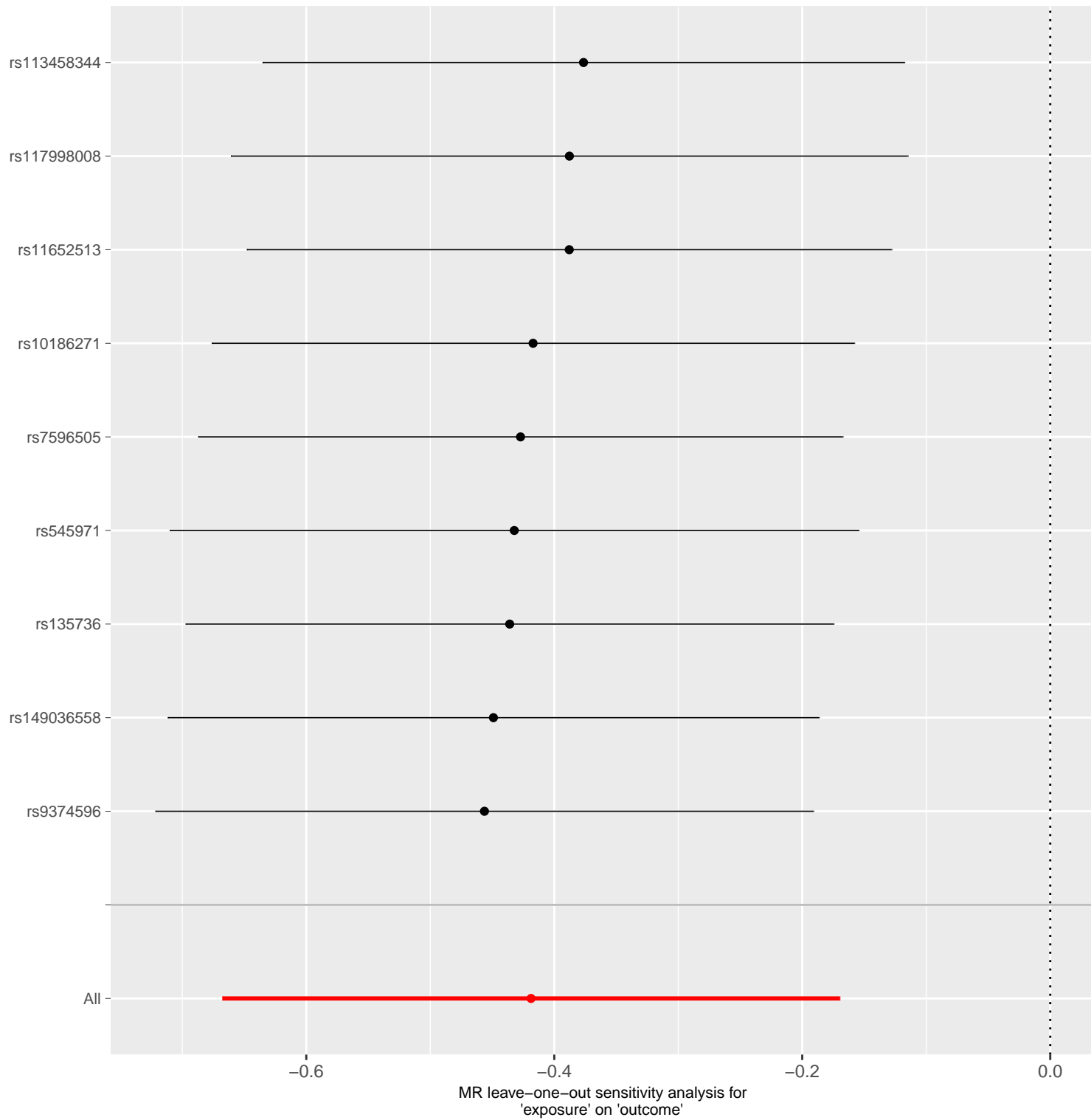

Supplement: Supplementary Data Sheet 2 — Full GCST identifiers, taxonomic labels, and Mendelian randomization statistics for the gut microbial traits associated with ulcerative colitis. [file DataSheet2.zip › GM_result/GCST90032382/sensitivity-analysis.pdf]

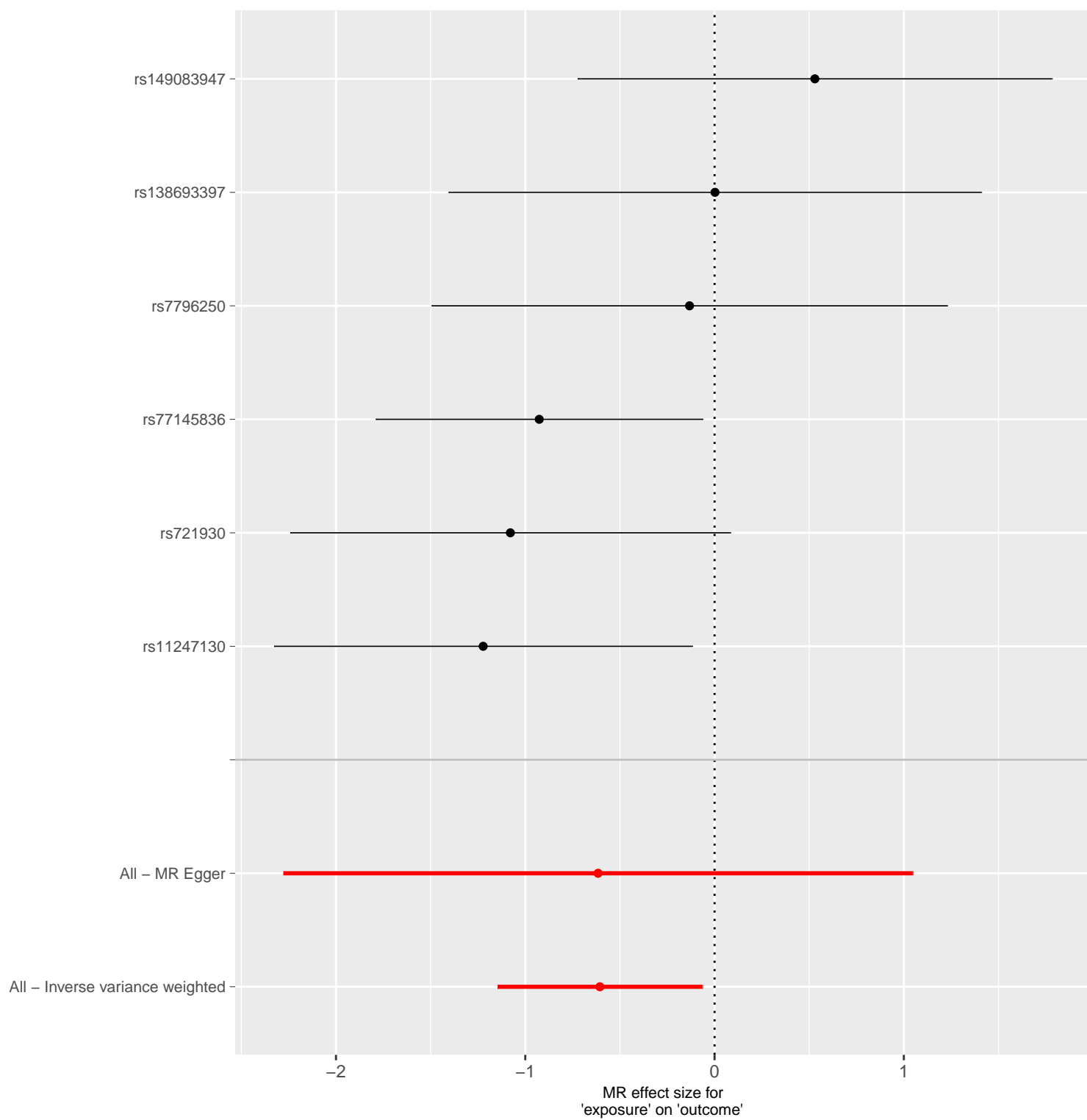

Supplement: Supplementary Data Sheet 2 — Full GCST identifiers, taxonomic labels, and Mendelian randomization statistics for the gut microbial traits associated with ulcerative colitis. [file DataSheet2.zip › GM_result/GCST90032385/forest.pdf]

# MR Method

Inverse variance weighted

MR Egger

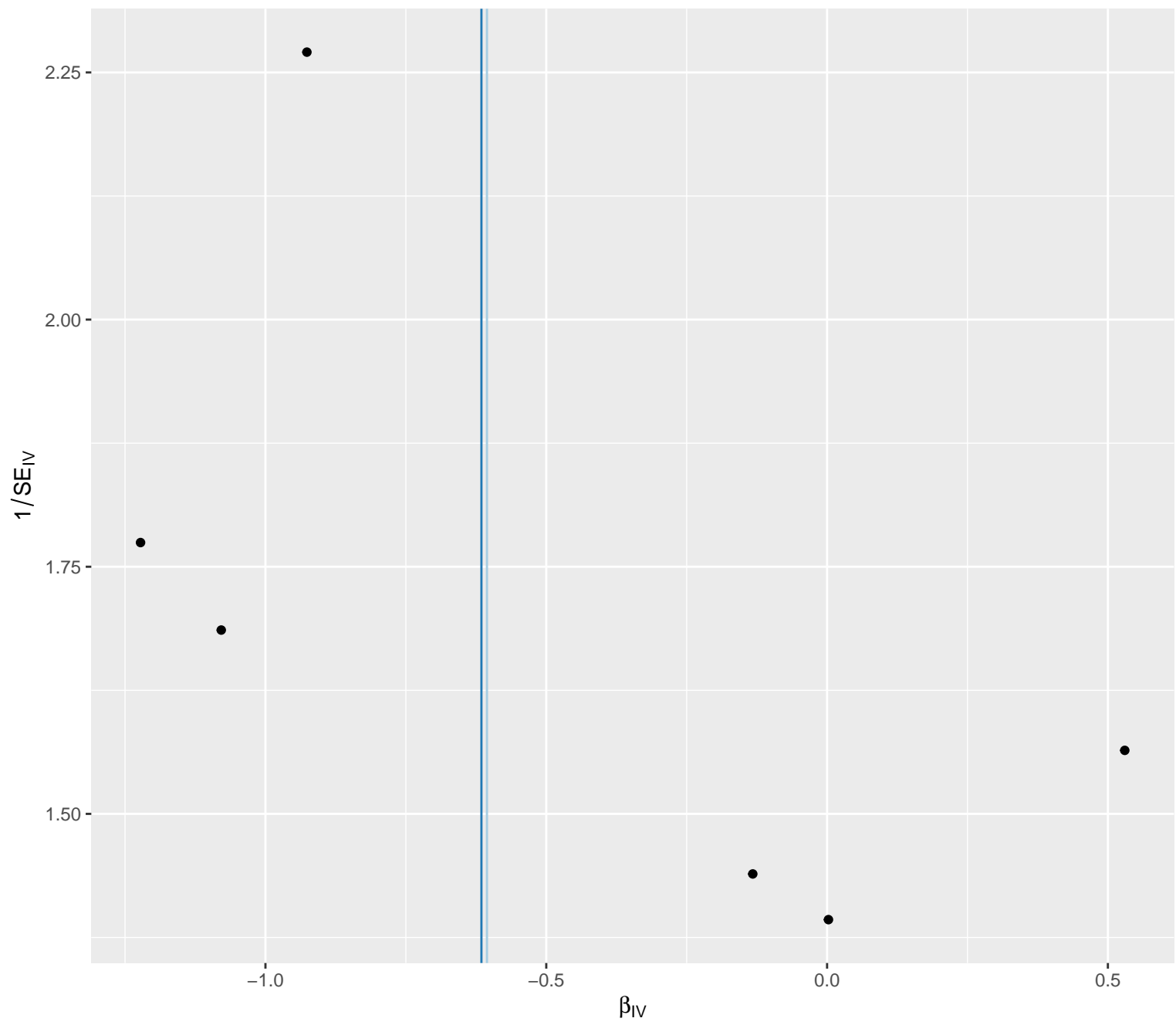

Supplement: Supplementary Data Sheet 2 — Full GCST identifiers, taxonomic labels, and Mendelian randomization statistics for the gut microbial traits associated with ulcerative colitis. [file DataSheet2.zip › GM_result/GCST90032385/funnelplot.pdf]

# MR Test

- Inverse variance weighted
- MR Egger
- Simple mode
- Weighted median
- Weighted mode

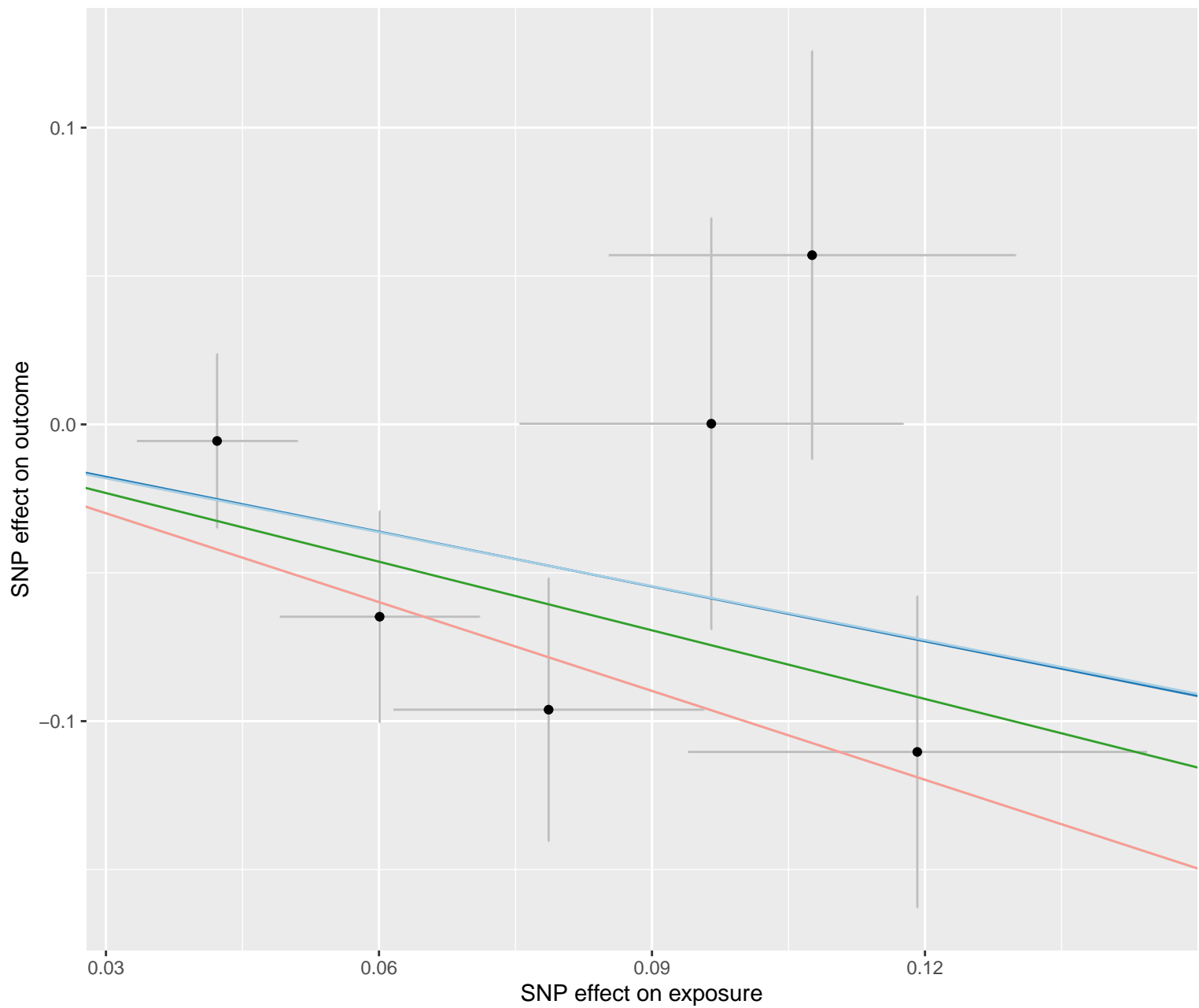

Supplement: Supplementary Data Sheet 2 — Full GCST identifiers, taxonomic labels, and Mendelian randomization statistics for the gut microbial traits associated with ulcerative colitis. [file DataSheet2.zip › GM_result/GCST90032385/scatter.pdf]

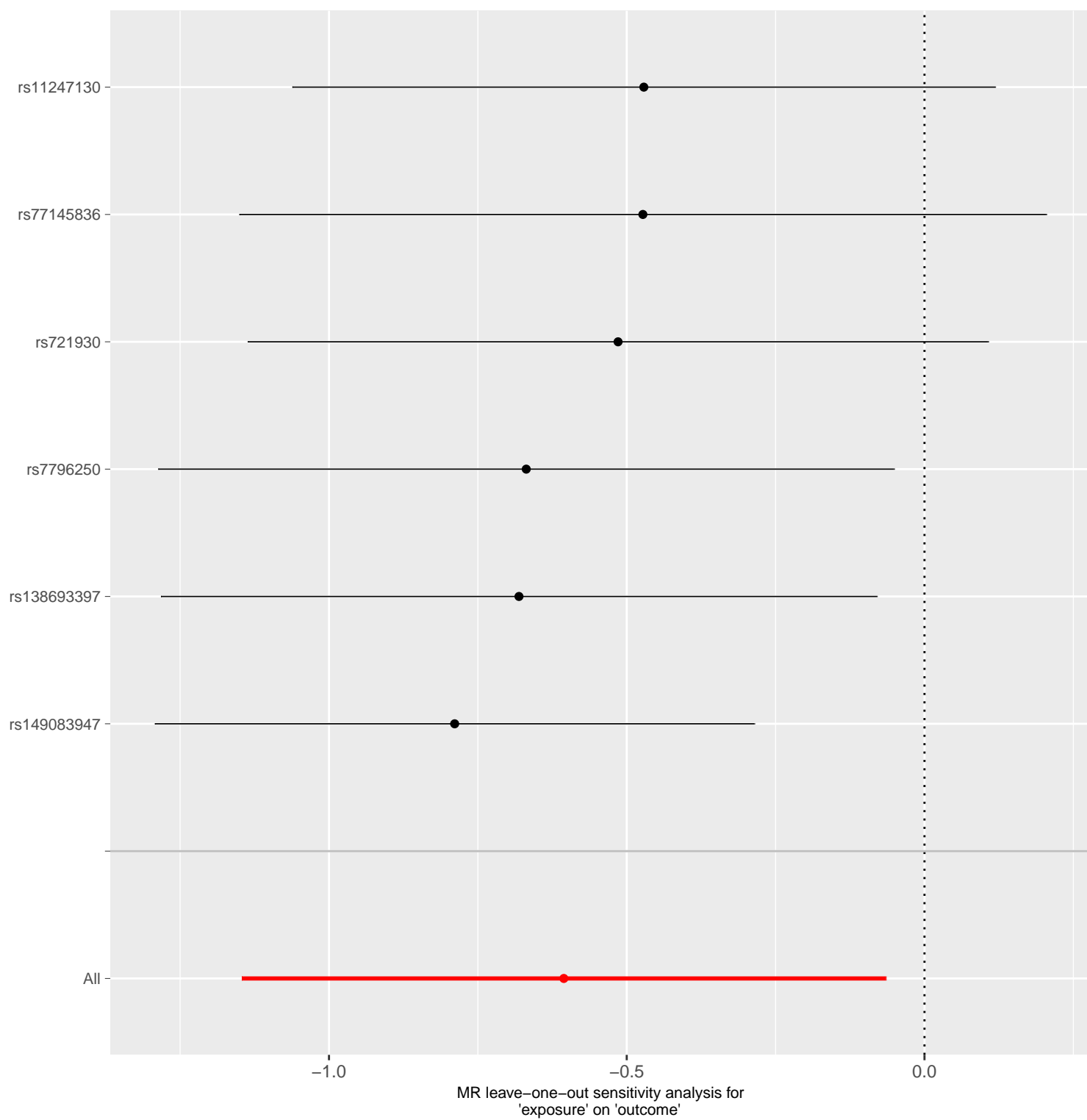

Supplement: Supplementary Data Sheet 2 — Full GCST identifiers, taxonomic labels, and Mendelian randomization statistics for the gut microbial traits associated with ulcerative colitis. [file DataSheet2.zip › GM_result/GCST90032385/sensitivity-analysis.pdf]

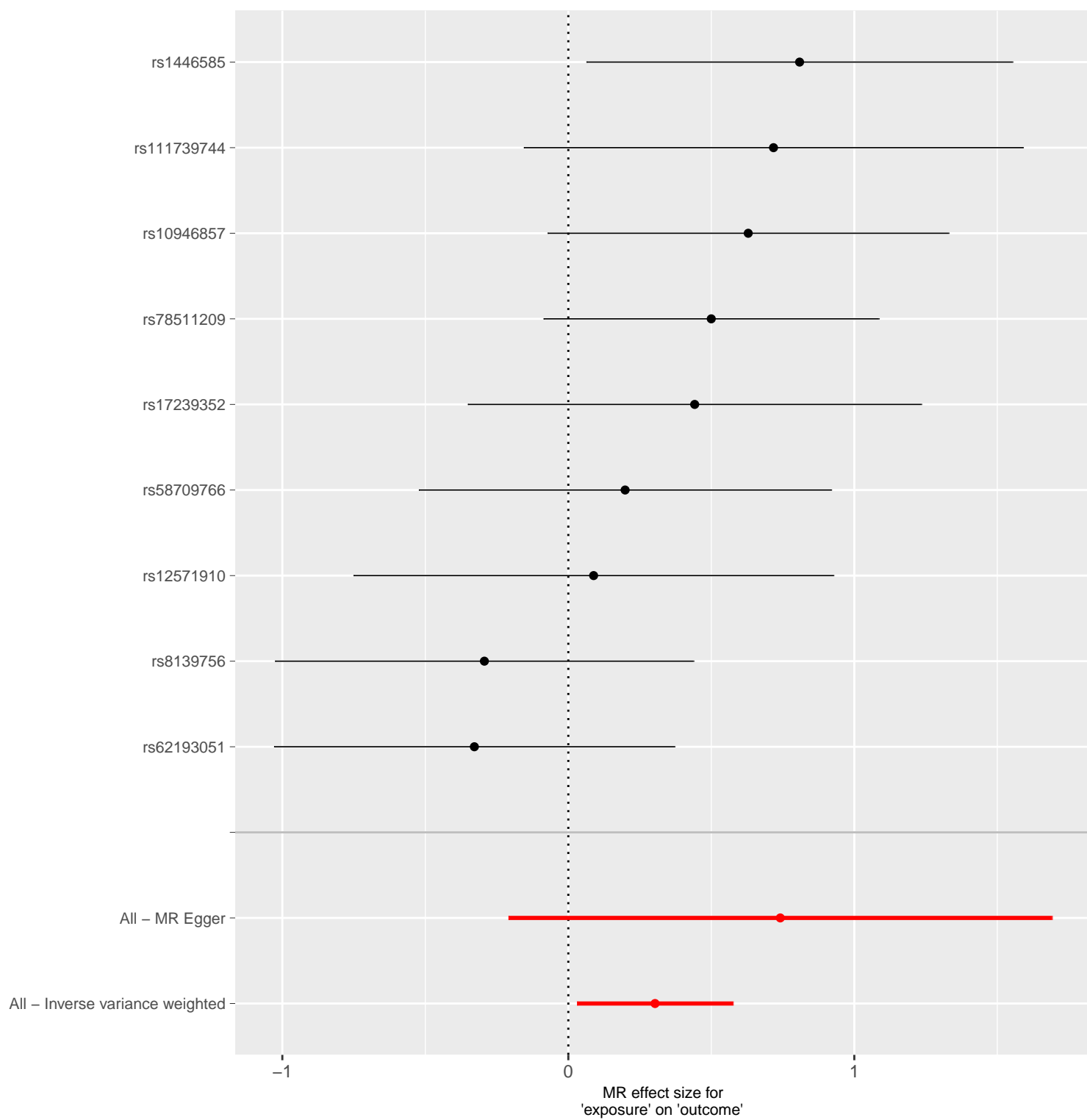

Supplement: Supplementary Data Sheet 2 — Full GCST identifiers, taxonomic labels, and Mendelian randomization statistics for the gut microbial traits associated with ulcerative colitis. [file DataSheet2.zip › GM_result/GCST90032431/forest.pdf]

# MR Method

Inverse variance weighted  
MR Egger

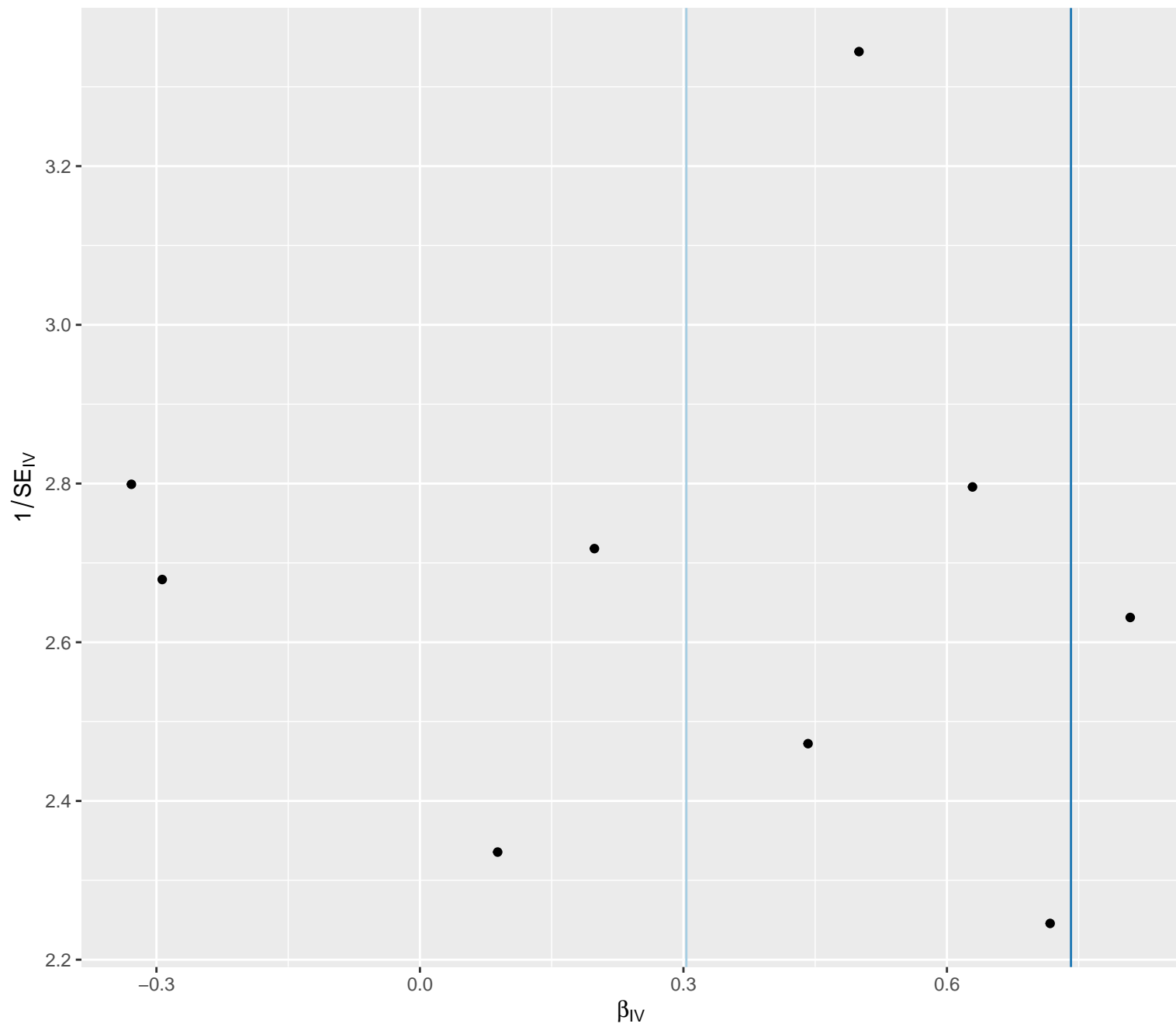

Supplement: Supplementary Data Sheet 2 — Full GCST identifiers, taxonomic labels, and Mendelian randomization statistics for the gut microbial traits associated with ulcerative colitis. [file DataSheet2.zip › GM_result/GCST90032431/funnelplot.pdf]

# MR Test

- Inverse variance weighted
- MR Egger
- Simple mode
- Weighted median
- Weighted mode

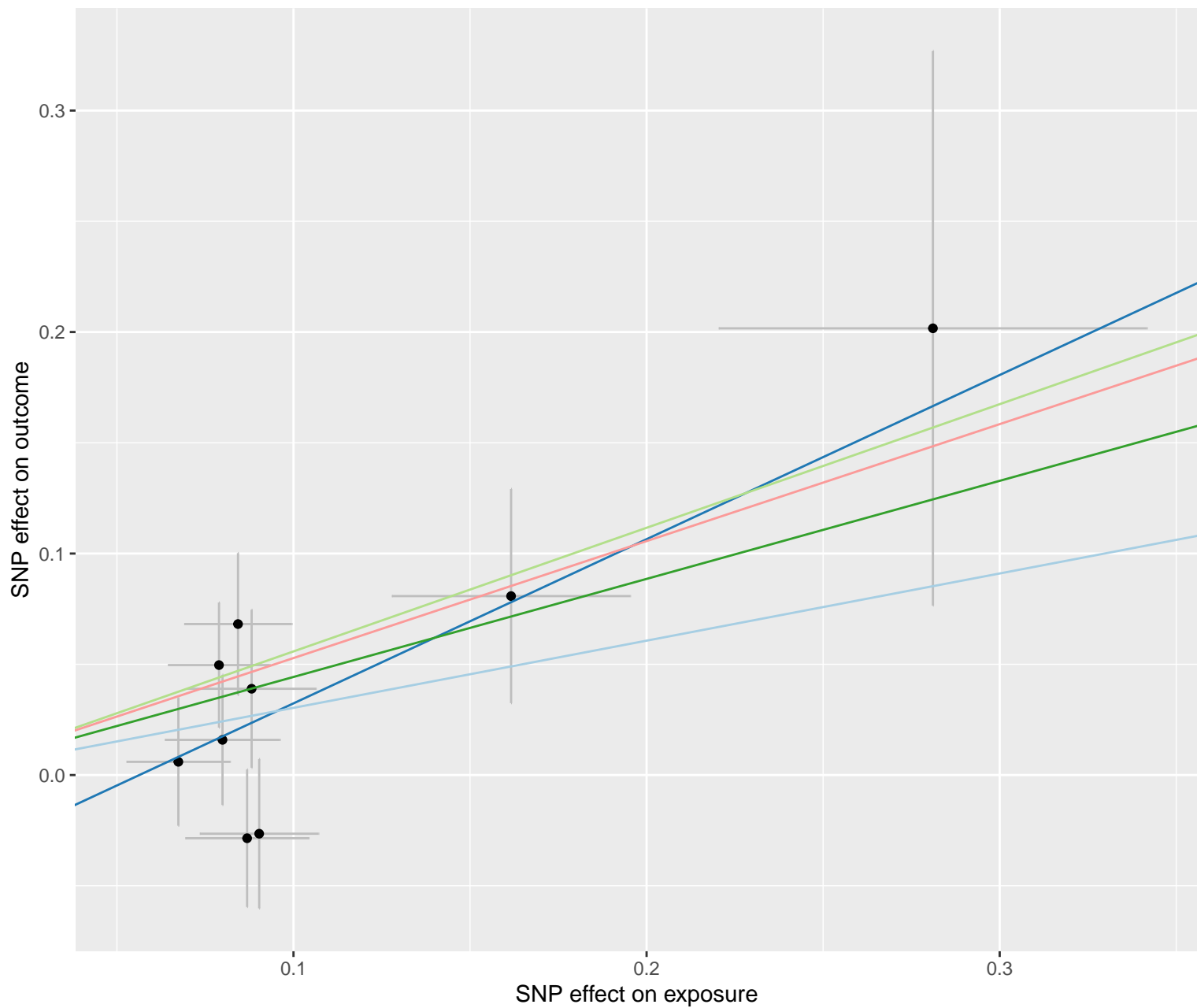

Supplement: Supplementary Data Sheet 2 — Full GCST identifiers, taxonomic labels, and Mendelian randomization statistics for the gut microbial traits associated with ulcerative colitis. [file DataSheet2.zip › GM_result/GCST90032431/scatter.pdf]

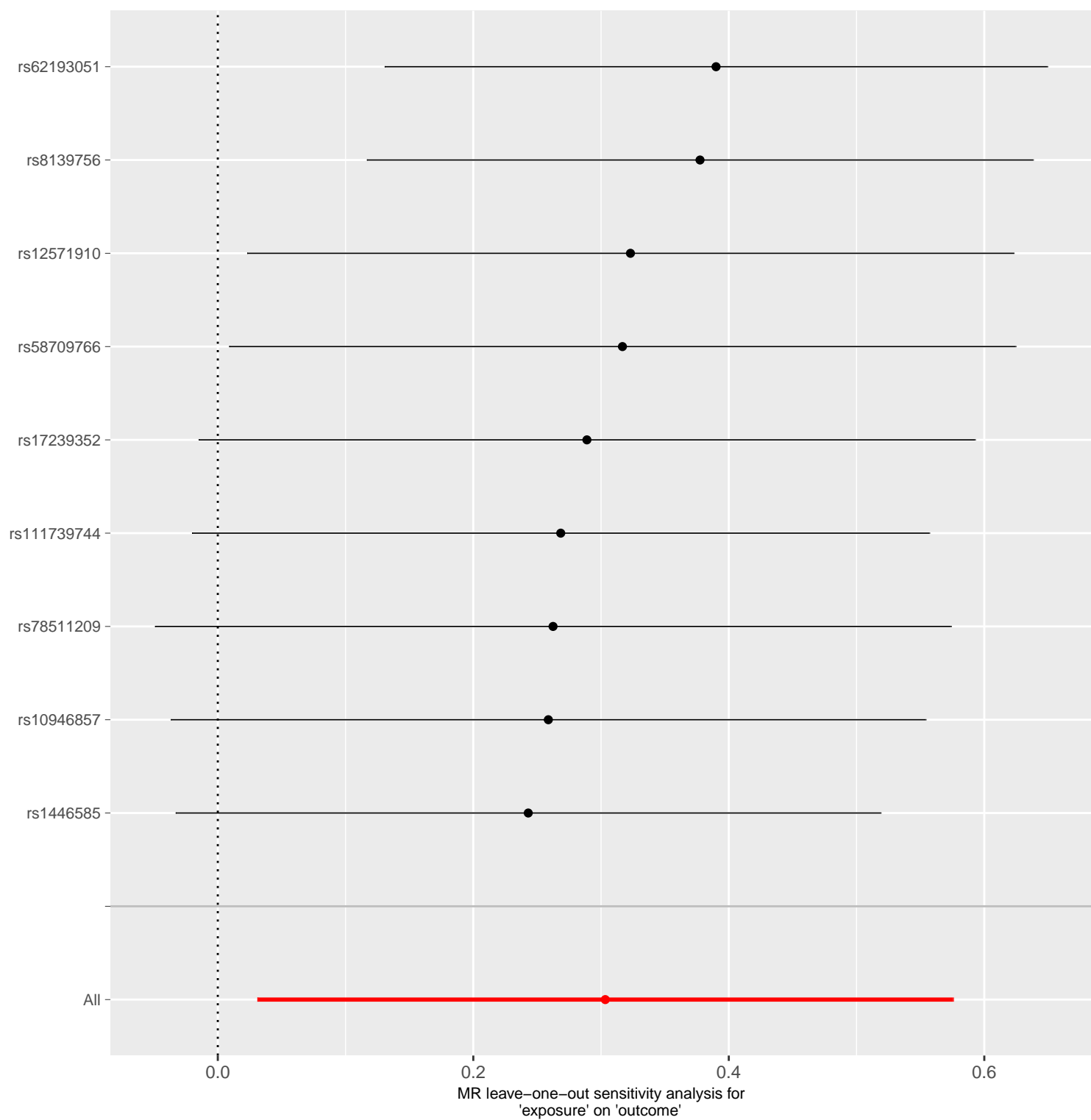

Supplement: Supplementary Data Sheet 2 — Full GCST identifiers, taxonomic labels, and Mendelian randomization statistics for the gut microbial traits associated with ulcerative colitis. [file DataSheet2.zip › GM_result/GCST90032431/sensitivity-analysis.pdf]

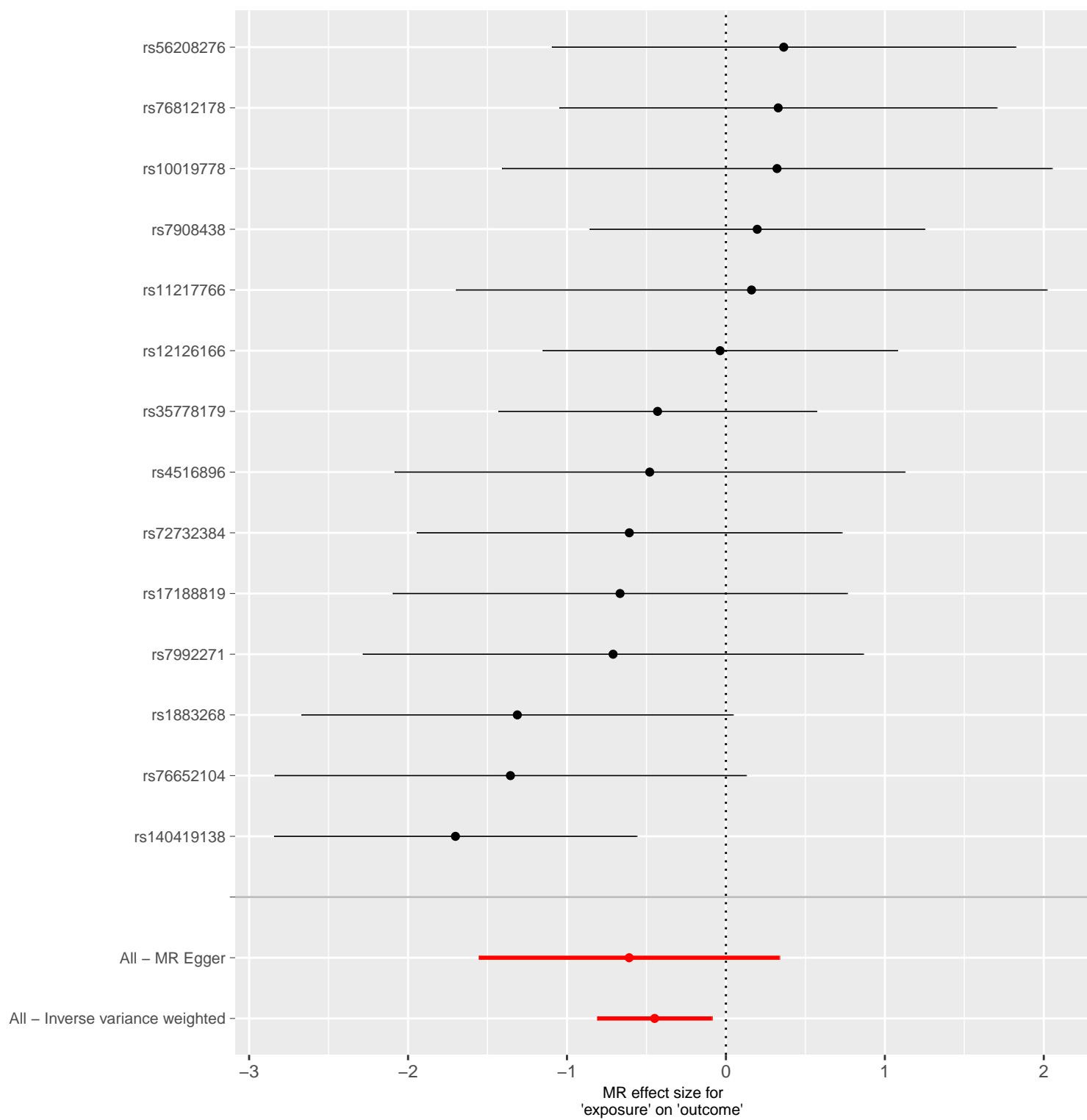

Supplement: Supplementary Data Sheet 2 — Full GCST identifiers, taxonomic labels, and Mendelian randomization statistics for the gut microbial traits associated with ulcerative colitis. [file DataSheet2.zip › GM_result/GCST90032455/forest.pdf]

# MR Method

- Inverse variance weighted
- MR Egger

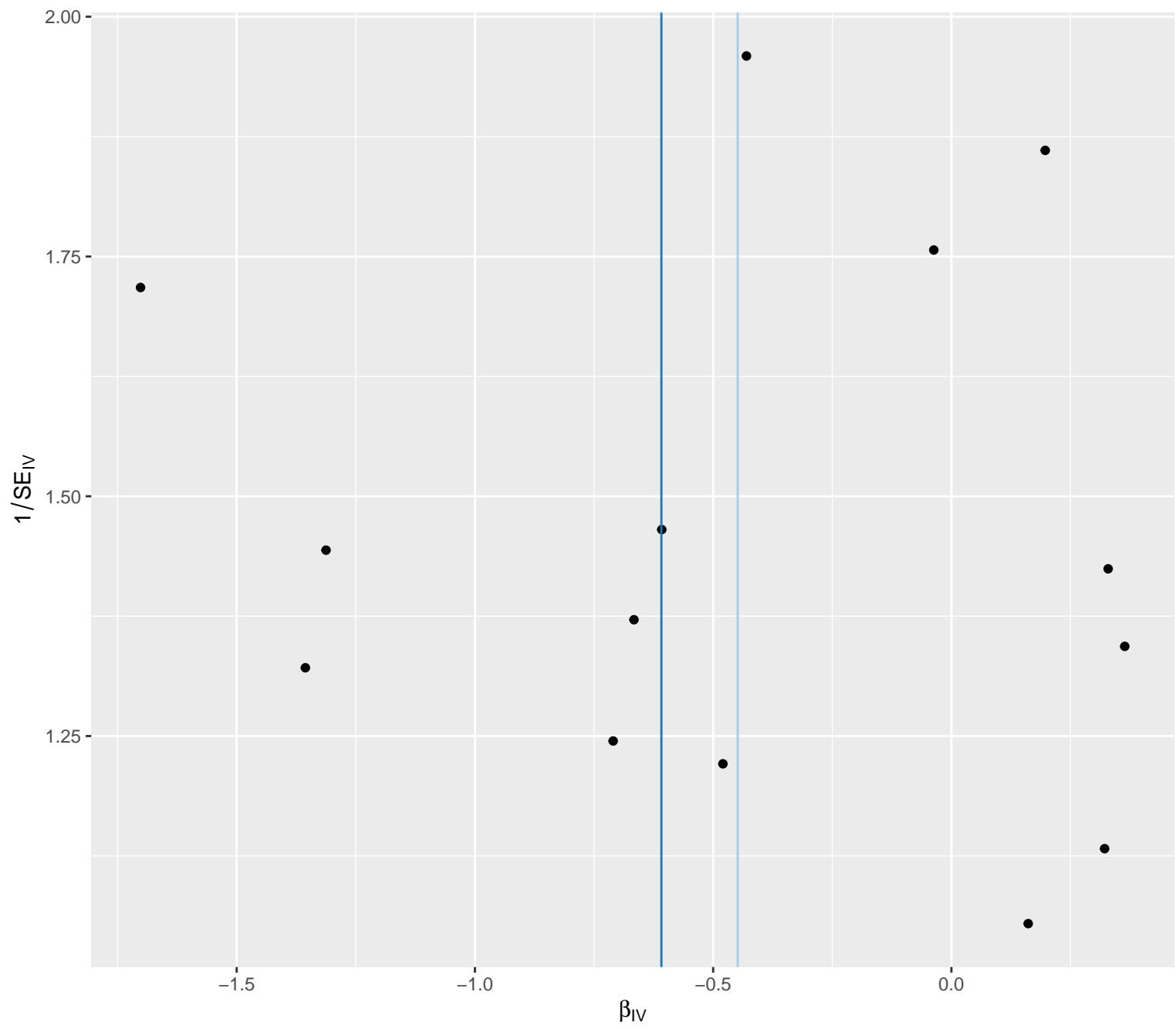

Supplement: Supplementary Data Sheet 2 — Full GCST identifiers, taxonomic labels, and Mendelian randomization statistics for the gut microbial traits associated with ulcerative colitis. [file DataSheet2.zip › GM_result/GCST90032455/funnelplot.pdf]

# MR Test

- Inverse variance weighted
- MR Egger
- Simple mode
- Weighted median
- Weighted mode

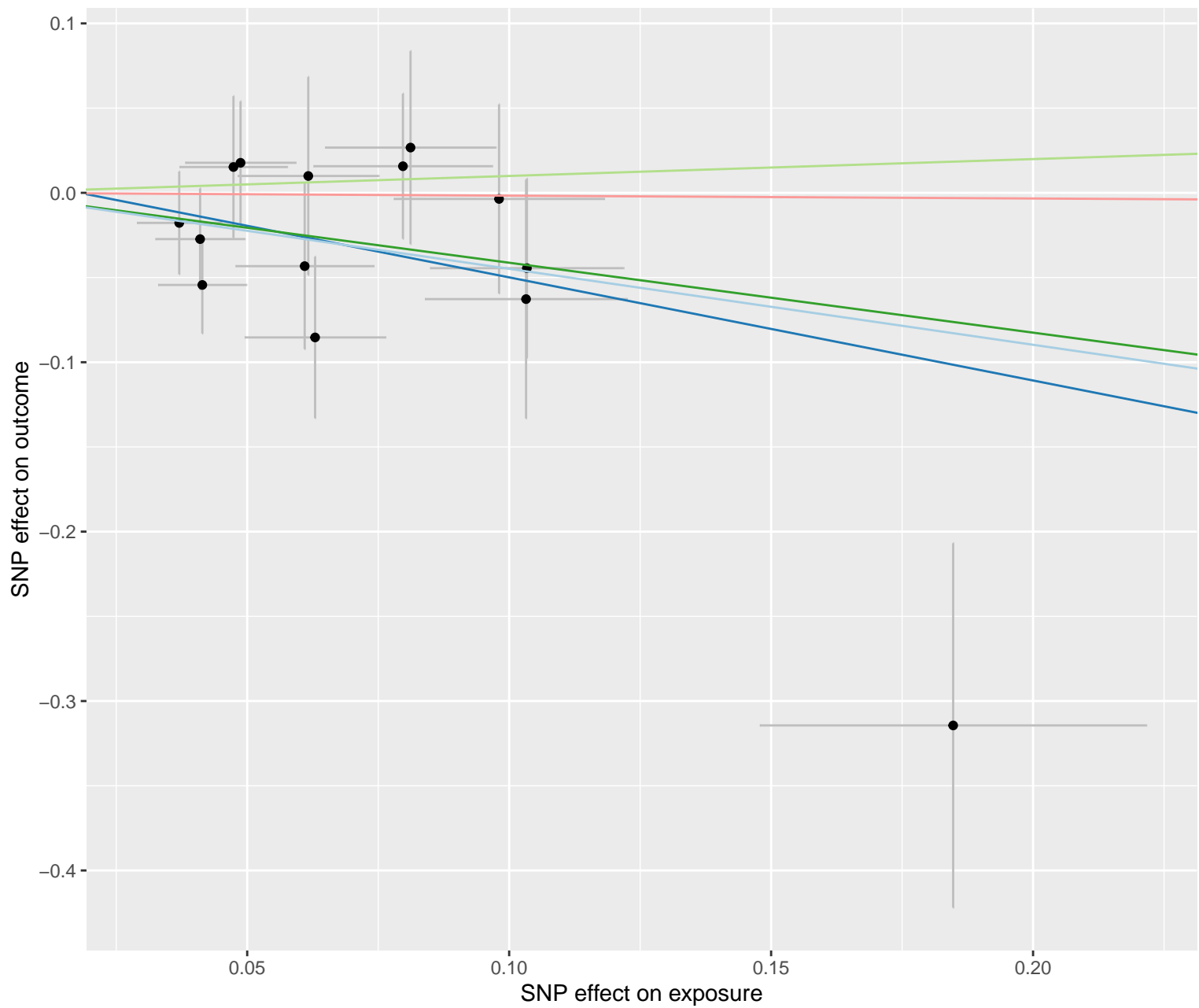

Supplement: Supplementary Data Sheet 2 — Full GCST identifiers, taxonomic labels, and Mendelian randomization statistics for the gut microbial traits associated with ulcerative colitis. [file DataSheet2.zip › GM_result/GCST90032455/scatter.pdf]

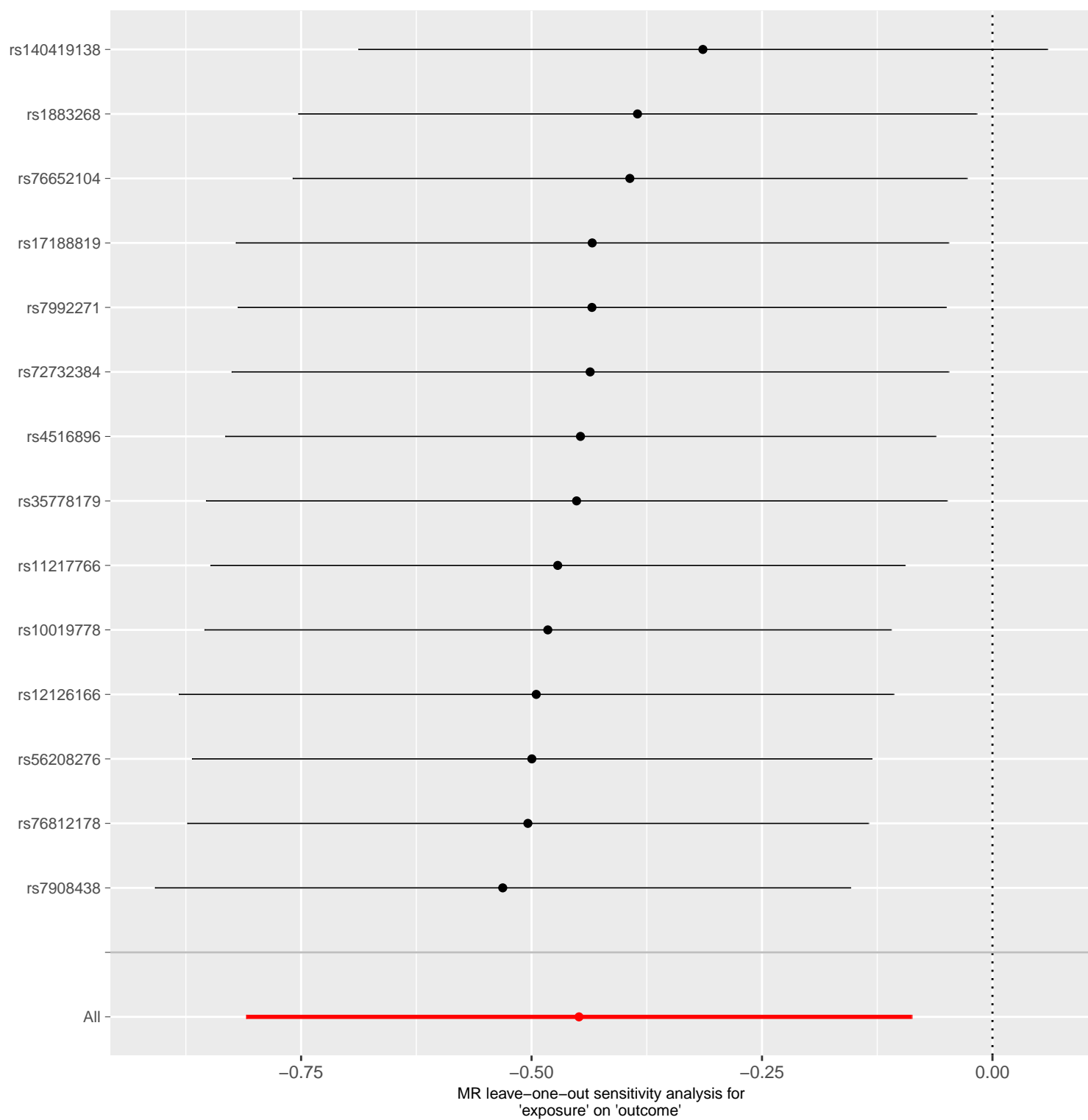

Supplement: Supplementary Data Sheet 2 — Full GCST identifiers, taxonomic labels, and Mendelian randomization statistics for the gut microbial traits associated with ulcerative colitis. [file DataSheet2.zip › GM_result/GCST90032455/sensitivity-analysis.pdf]

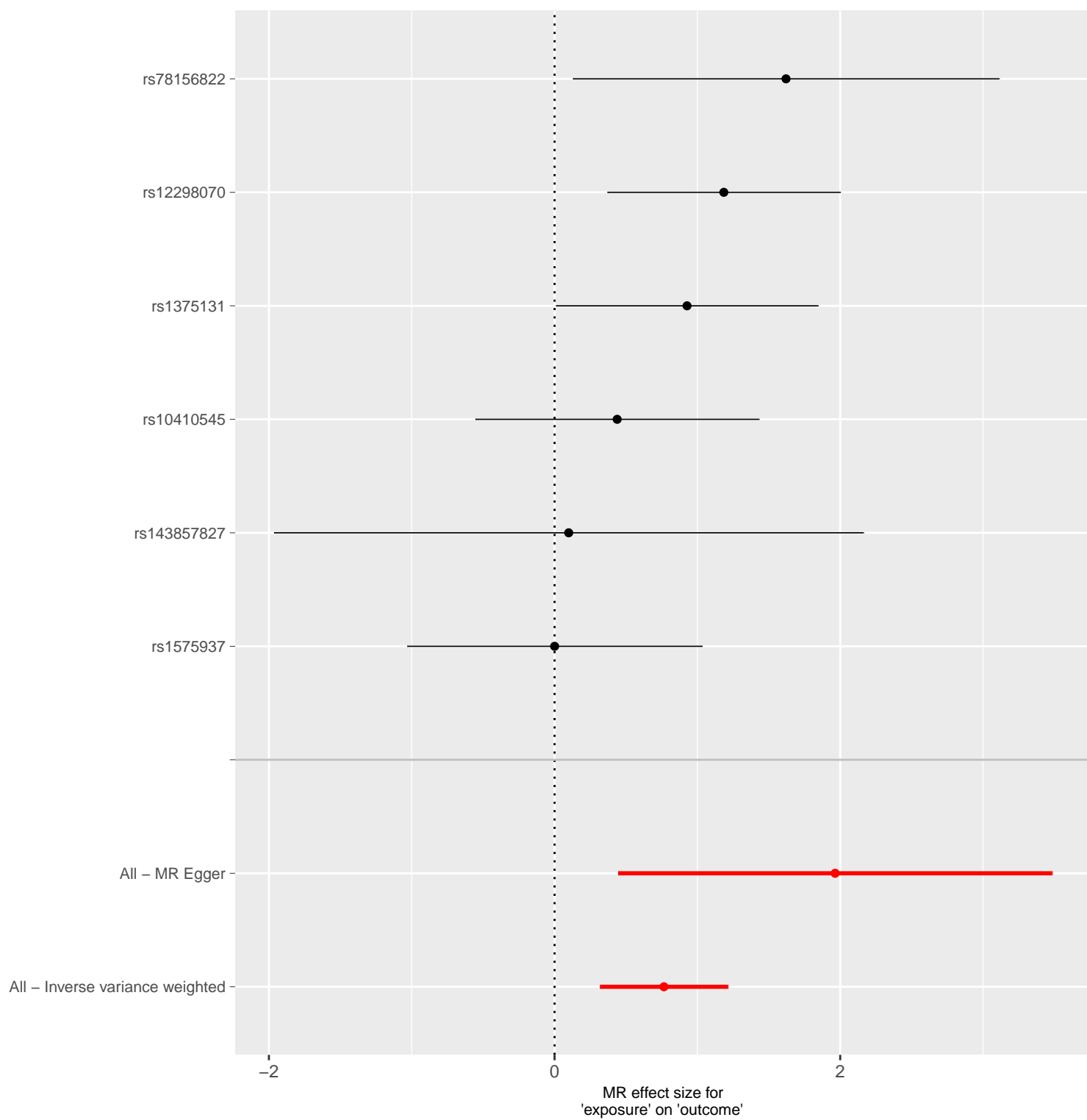

Supplement: Supplementary Data Sheet 2 — Full GCST identifiers, taxonomic labels, and Mendelian randomization statistics for the gut microbial traits associated with ulcerative colitis. [file DataSheet2.zip › GM_result/GCST90032466/forest.pdf]

# MR Method

- Inverse variance weighted
- MR Egger

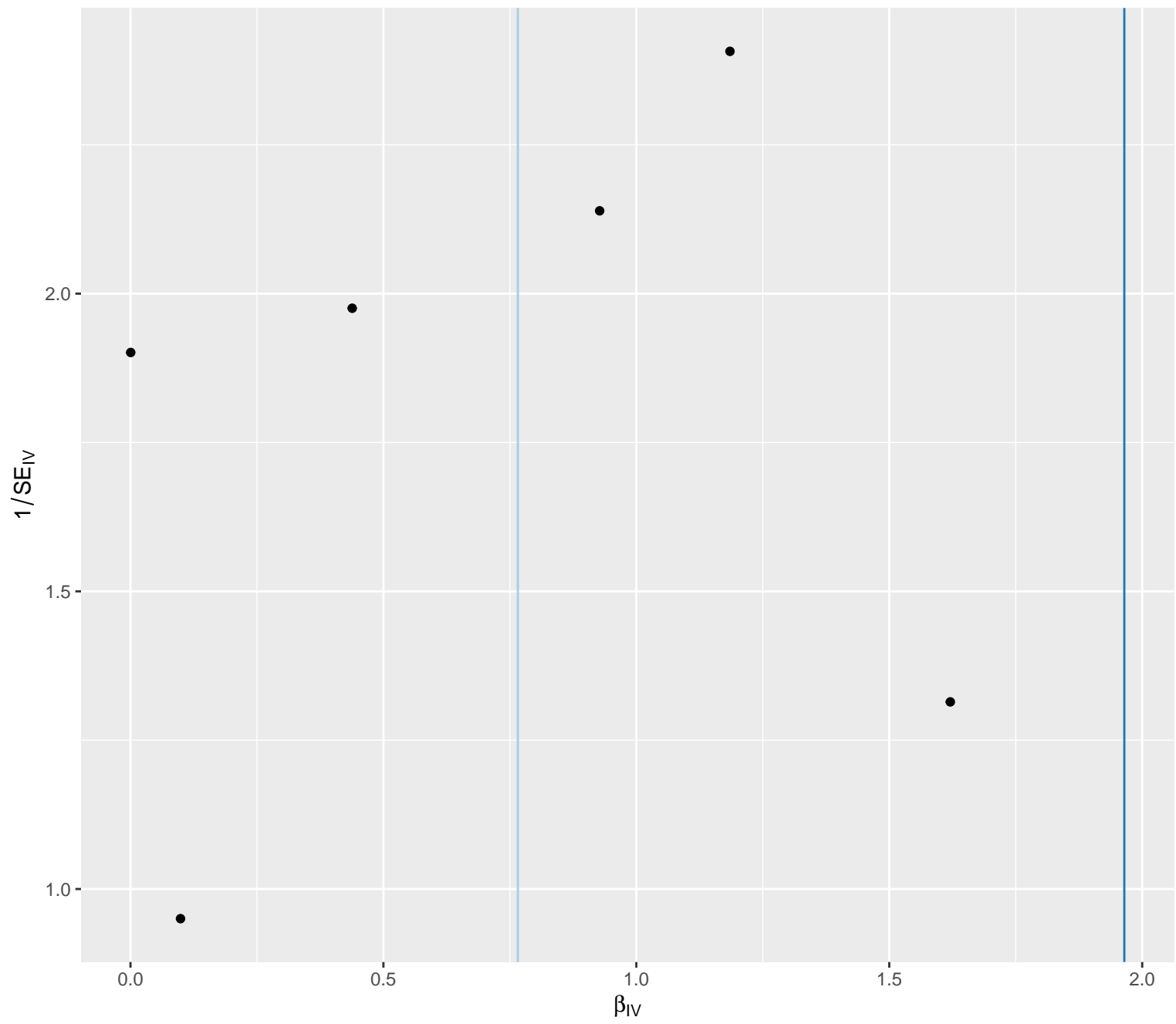

Supplement: Supplementary Data Sheet 2 — Full GCST identifiers, taxonomic labels, and Mendelian randomization statistics for the gut microbial traits associated with ulcerative colitis. [file DataSheet2.zip › GM_result/GCST90032466/funnelplot.pdf]

# MR Test

- Inverse variance weighted
- MR Egger
- Simple mode
- Weighted median
- Weighted mode

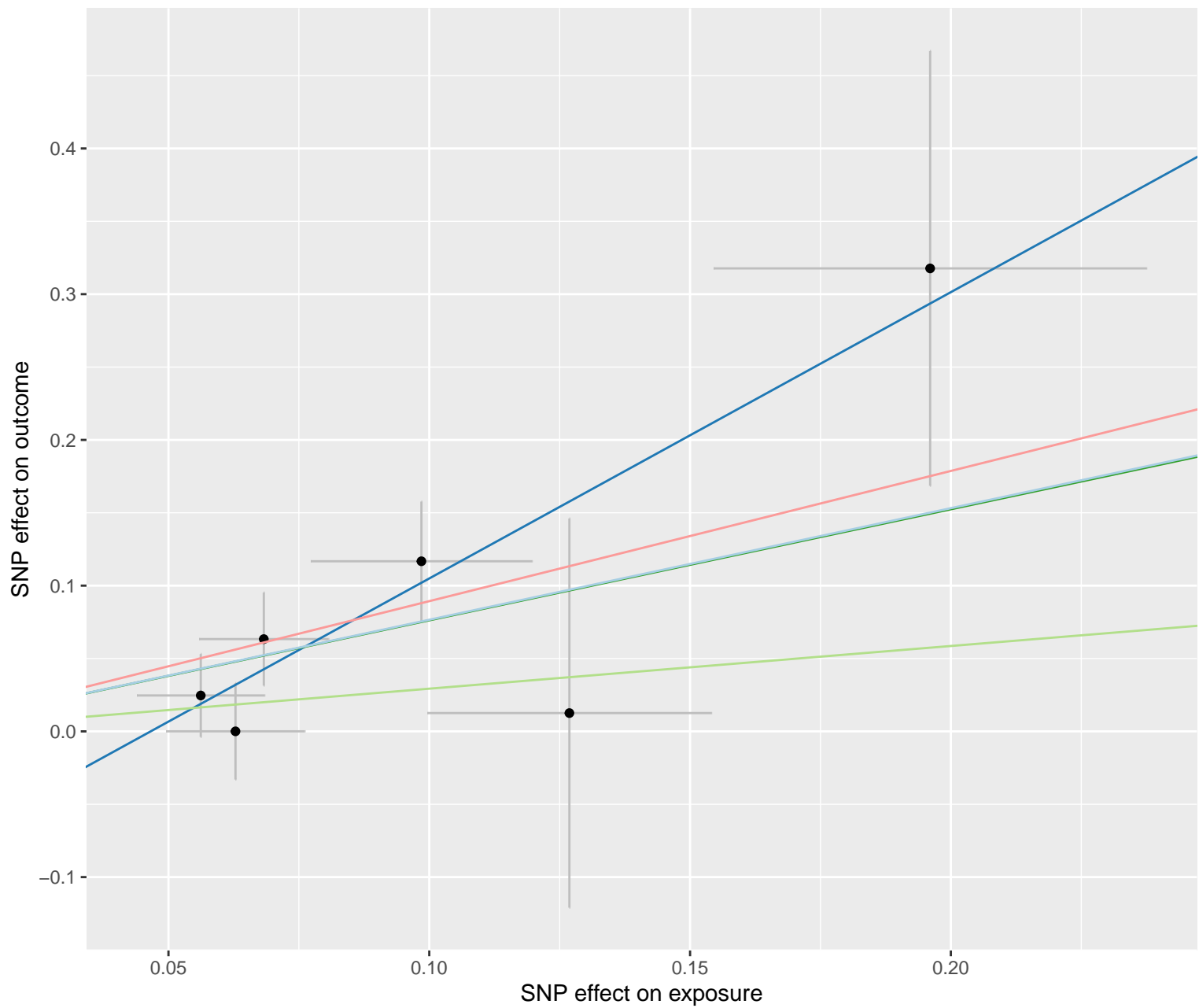

Supplement: Supplementary Data Sheet 2 — Full GCST identifiers, taxonomic labels, and Mendelian randomization statistics for the gut microbial traits associated with ulcerative colitis. [file DataSheet2.zip › GM_result/GCST90032466/scatter.pdf]

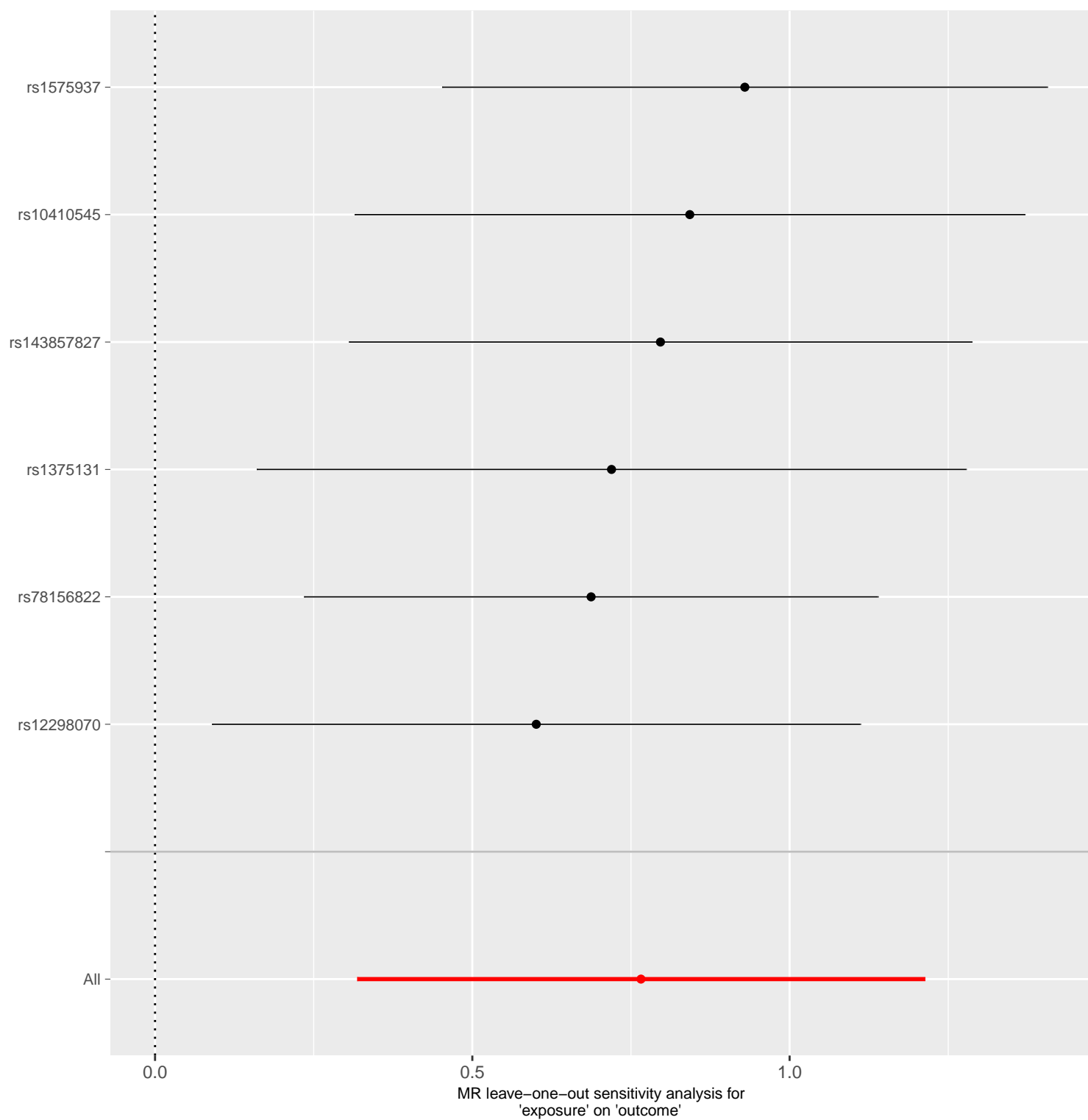

Supplement: Supplementary Data Sheet 2 — Full GCST identifiers, taxonomic labels, and Mendelian randomization statistics for the gut microbial traits associated with ulcerative colitis. [file DataSheet2.zip › GM_result/GCST90032466/sensitivity-analysis.pdf]

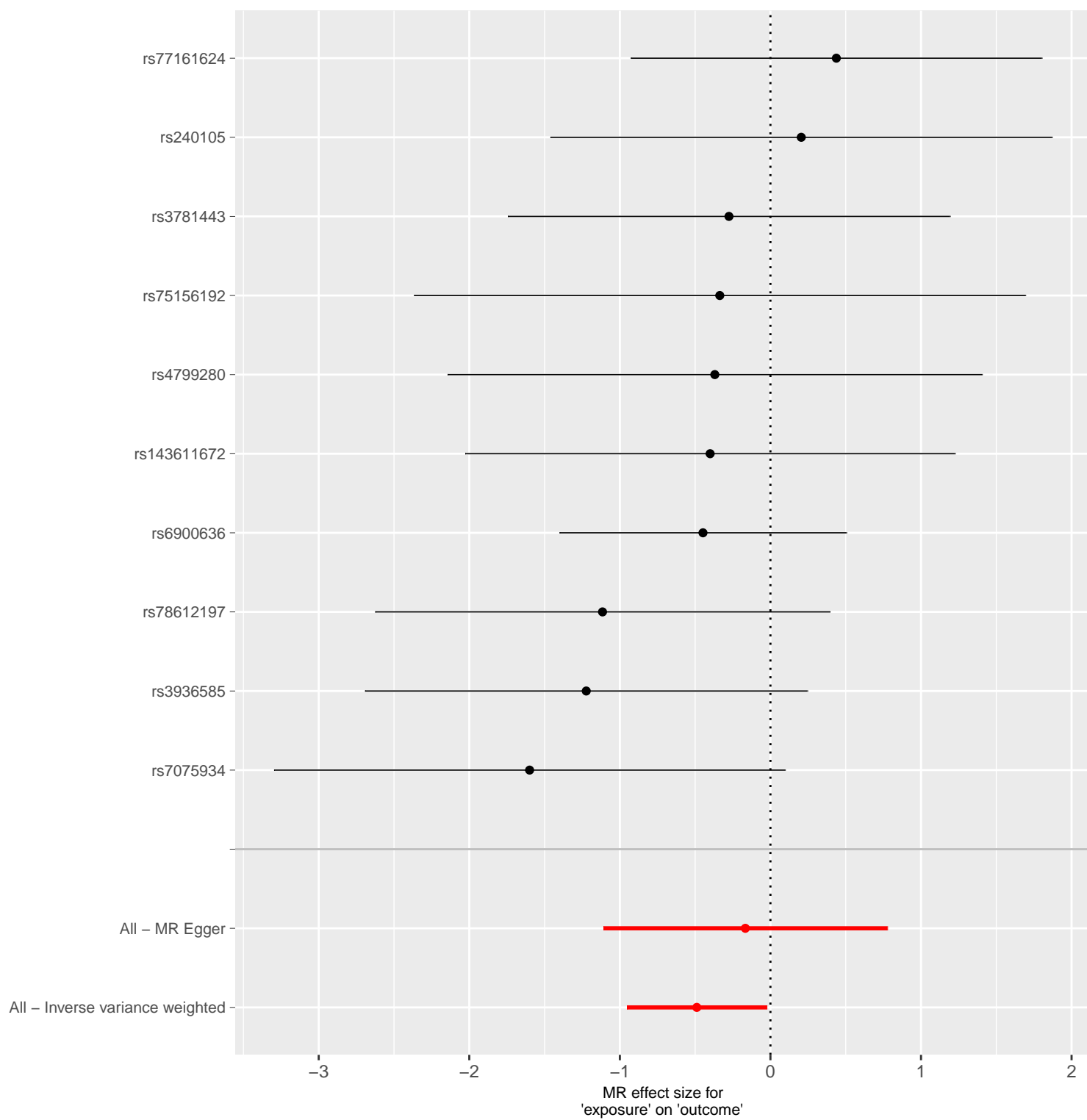

Supplement: Supplementary Data Sheet 2 — Full GCST identifiers, taxonomic labels, and Mendelian randomization statistics for the gut microbial traits associated with ulcerative colitis. [file DataSheet2.zip › GM_result/GCST90032474/forest.pdf]

# MR Method

- Inverse variance weighted
- MR Egger

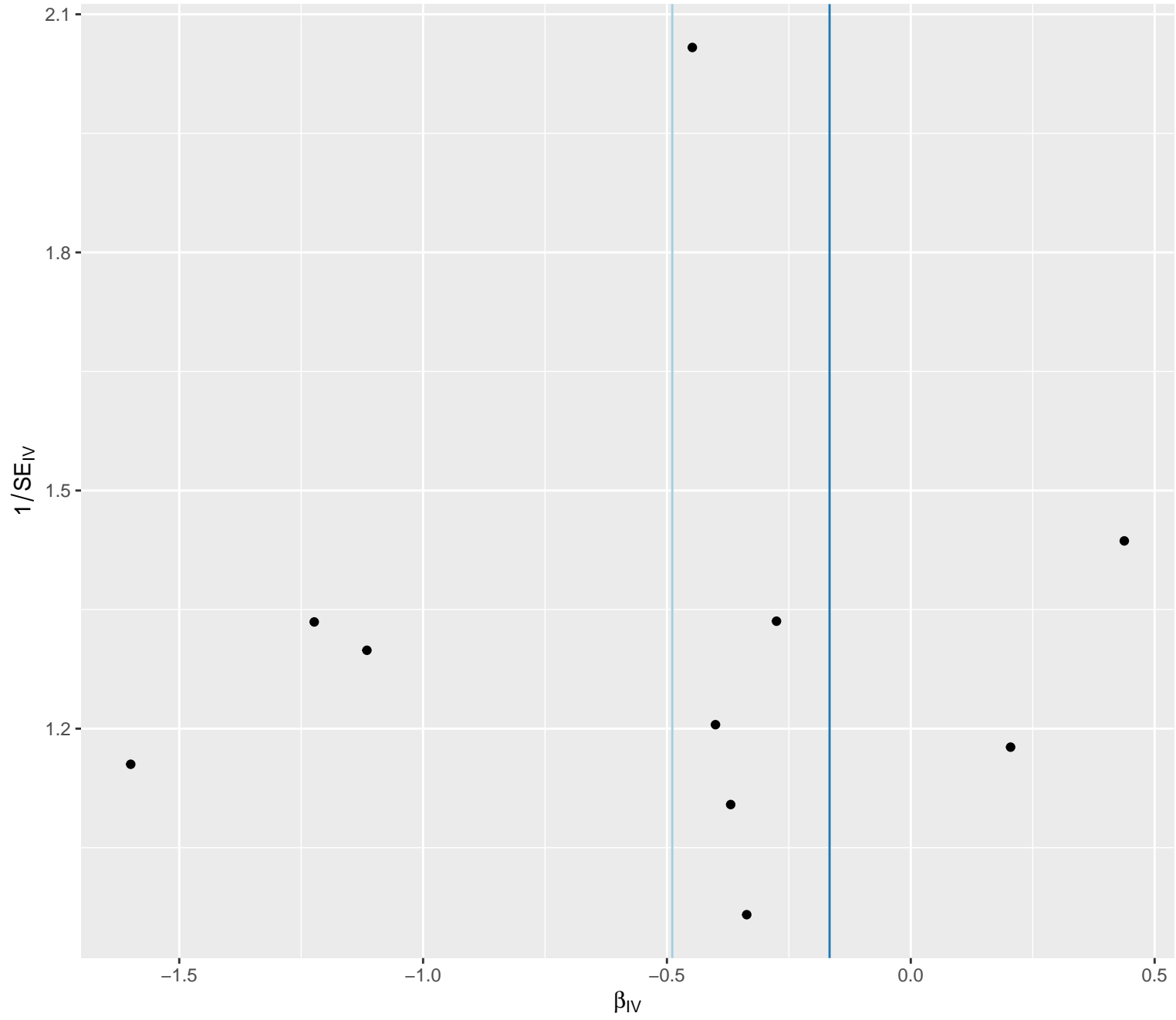

Supplement: Supplementary Data Sheet 2 — Full GCST identifiers, taxonomic labels, and Mendelian randomization statistics for the gut microbial traits associated with ulcerative colitis. [file DataSheet2.zip › GM_result/GCST90032474/funnelplot.pdf]

# MR Test

- Inverse variance weighted
- MR Egger
- Simple mode
- Weighted median
- Weighted mode

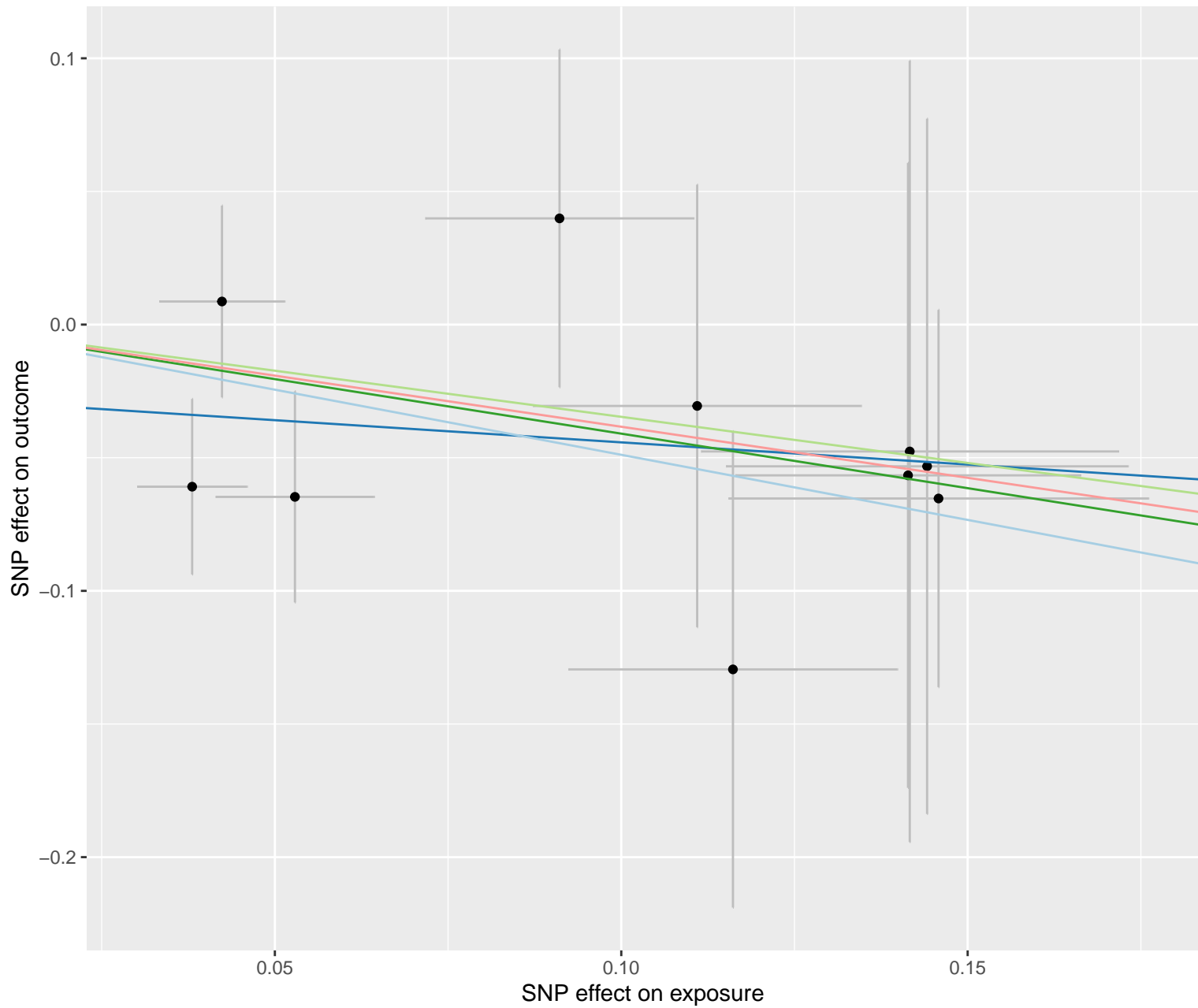

Supplement: Supplementary Data Sheet 2 — Full GCST identifiers, taxonomic labels, and Mendelian randomization statistics for the gut microbial traits associated with ulcerative colitis. [file DataSheet2.zip › GM_result/GCST90032474/scatter.pdf]

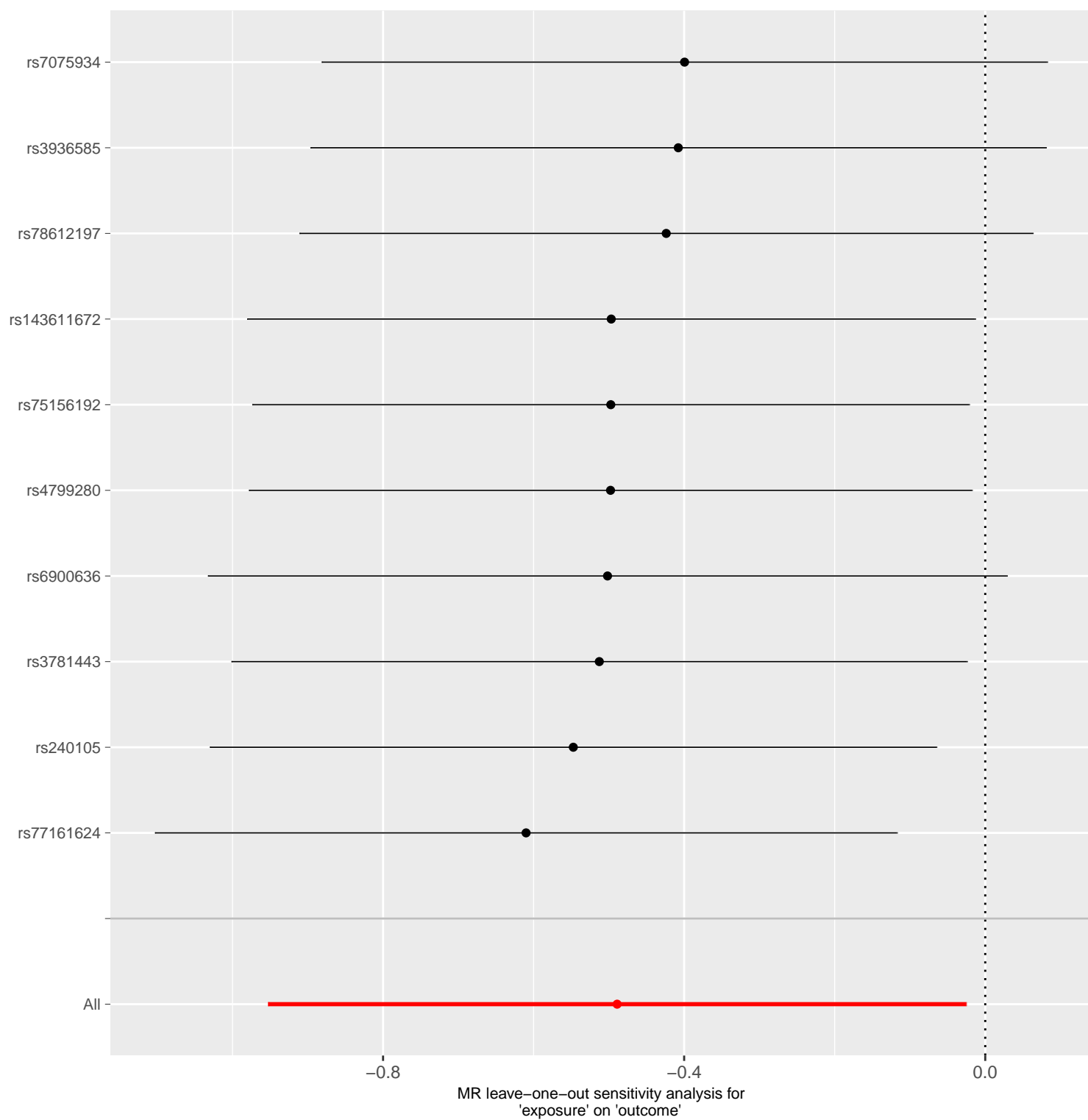

Supplement: Supplementary Data Sheet 2 — Full GCST identifiers, taxonomic labels, and Mendelian randomization statistics for the gut microbial traits associated with ulcerative colitis. [file DataSheet2.zip › GM_result/GCST90032474/sensitivity-analysis.pdf]

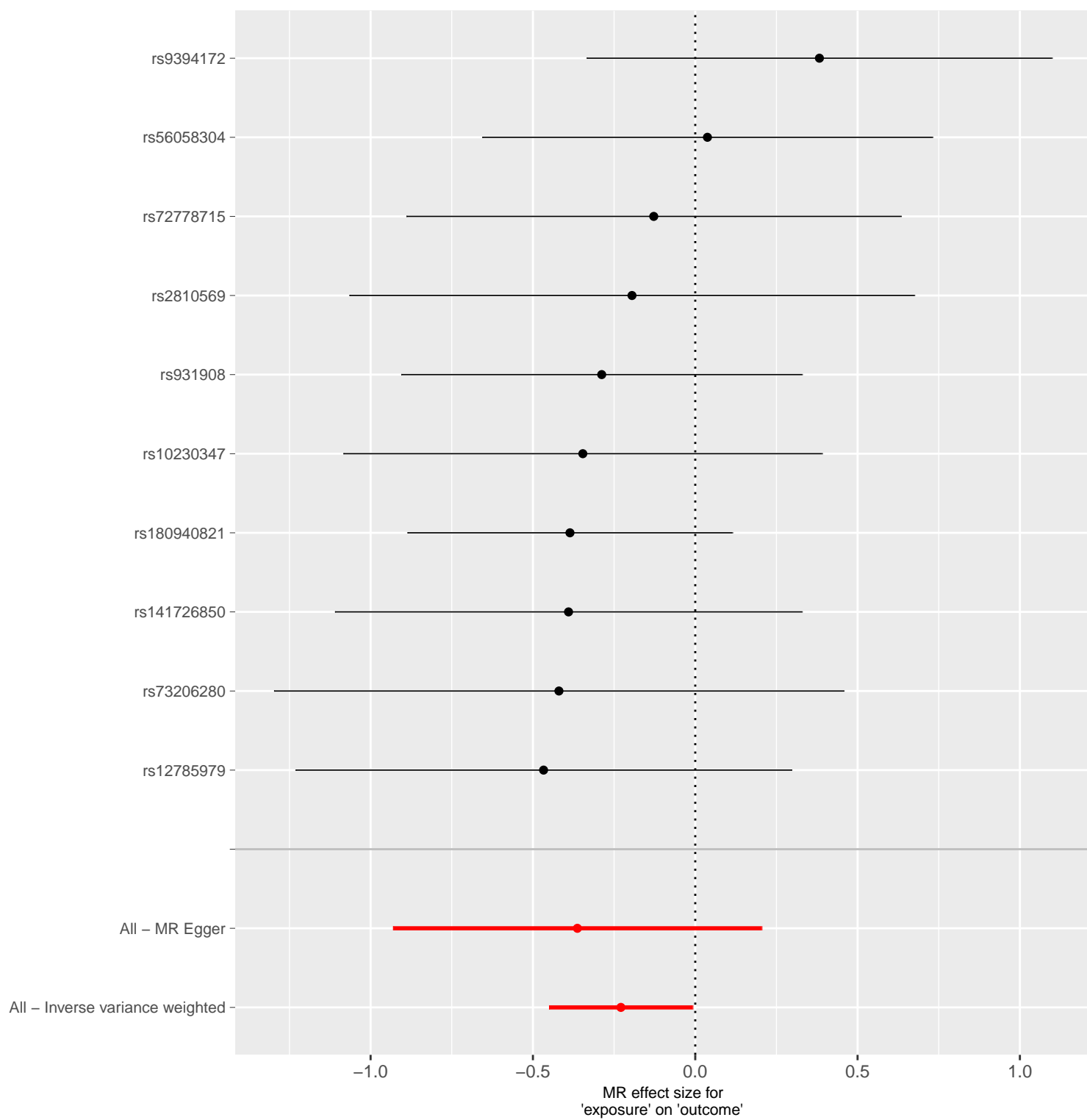

Supplement: Supplementary Data Sheet 2 — Full GCST identifiers, taxonomic labels, and Mendelian randomization statistics for the gut microbial traits associated with ulcerative colitis. [file DataSheet2.zip › GM_result/GCST90032519/forest.pdf]

# MR Method

- Inverse variance weighted
- MR Egger

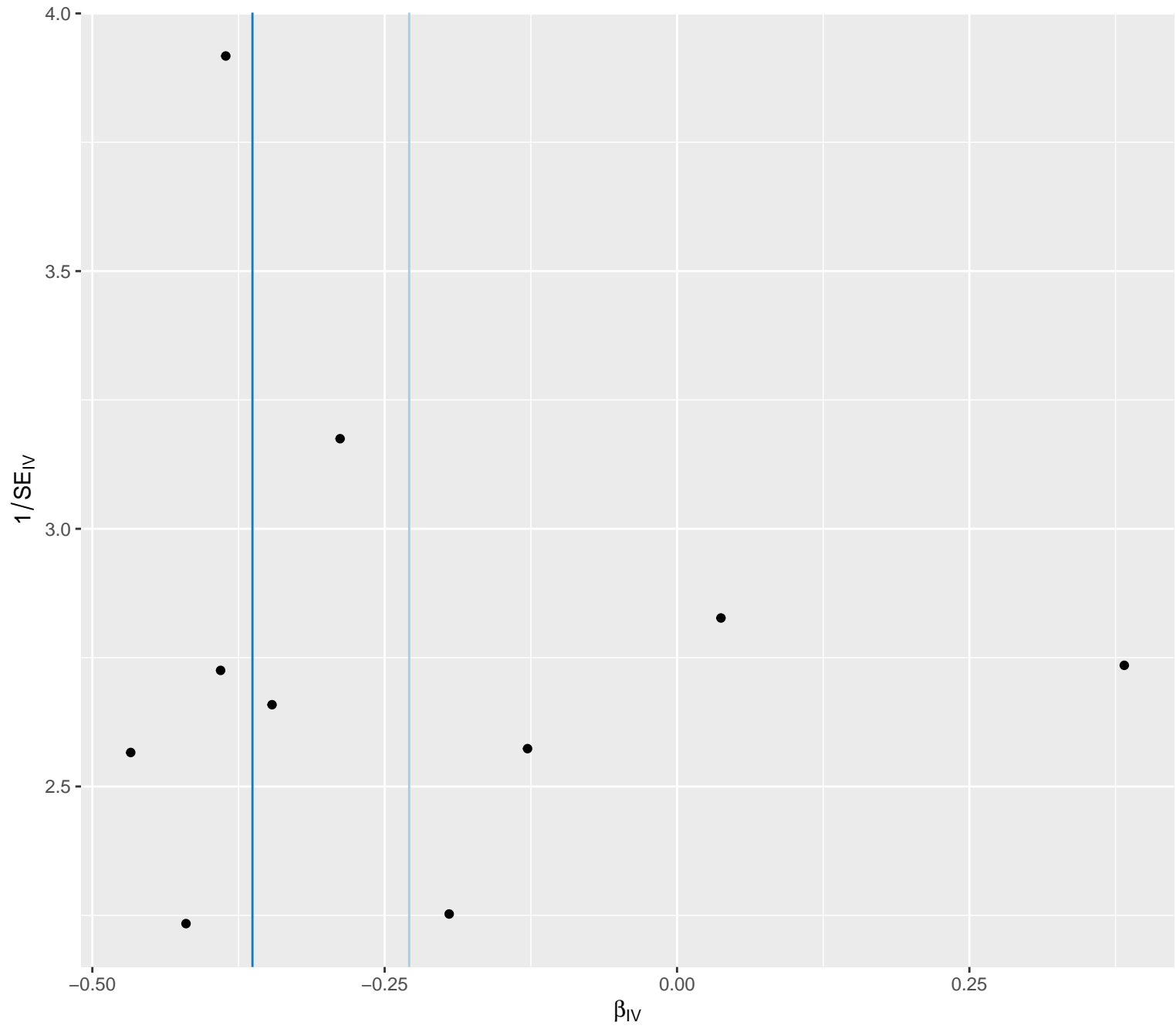

Supplement: Supplementary Data Sheet 2 — Full GCST identifiers, taxonomic labels, and Mendelian randomization statistics for the gut microbial traits associated with ulcerative colitis. [file DataSheet2.zip › GM_result/GCST90032519/funnelplot.pdf]

# MR Test

- Inverse variance weighted
- MR Egger
- Simple mode
- Weighted median
- Weighted mode

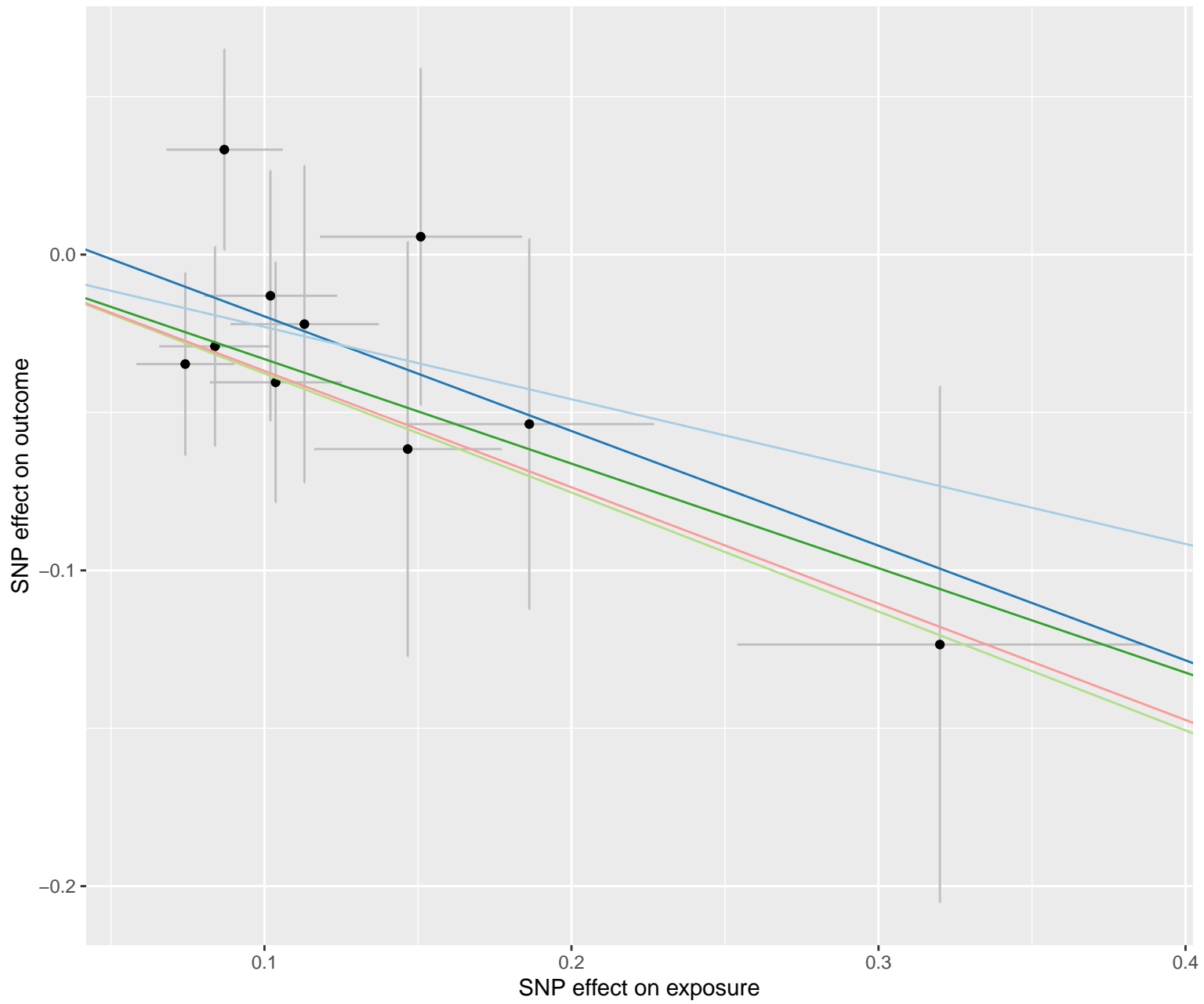

Supplement: Supplementary Data Sheet 2 — Full GCST identifiers, taxonomic labels, and Mendelian randomization statistics for the gut microbial traits associated with ulcerative colitis. [file DataSheet2.zip › GM_result/GCST90032519/scatter.pdf]

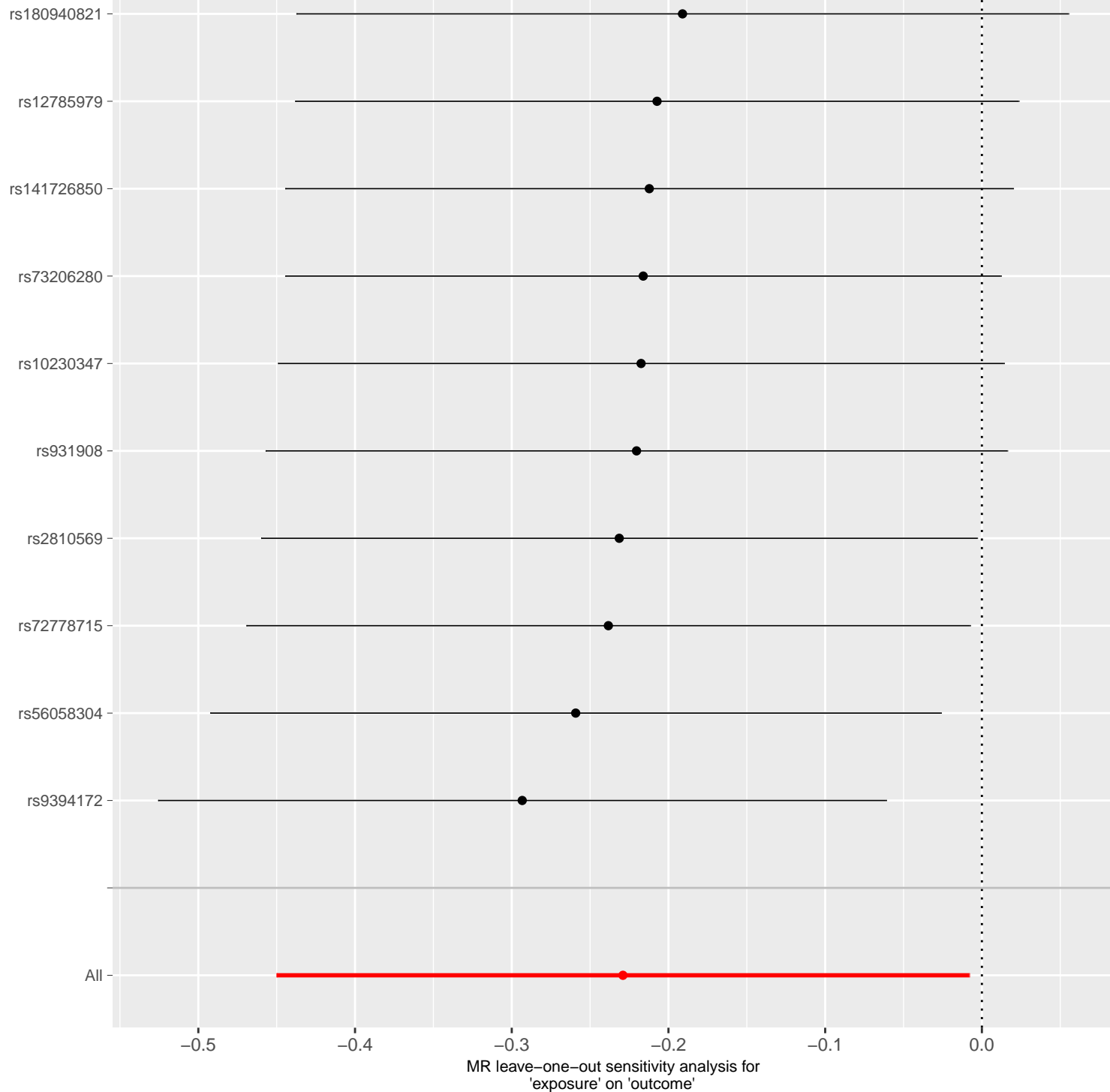

Supplement: Supplementary Data Sheet 2 — Full GCST identifiers, taxonomic labels, and Mendelian randomization statistics for the gut microbial traits associated with ulcerative colitis. [file DataSheet2.zip › GM_result/GCST90032519/sensitivity-analysis.pdf]

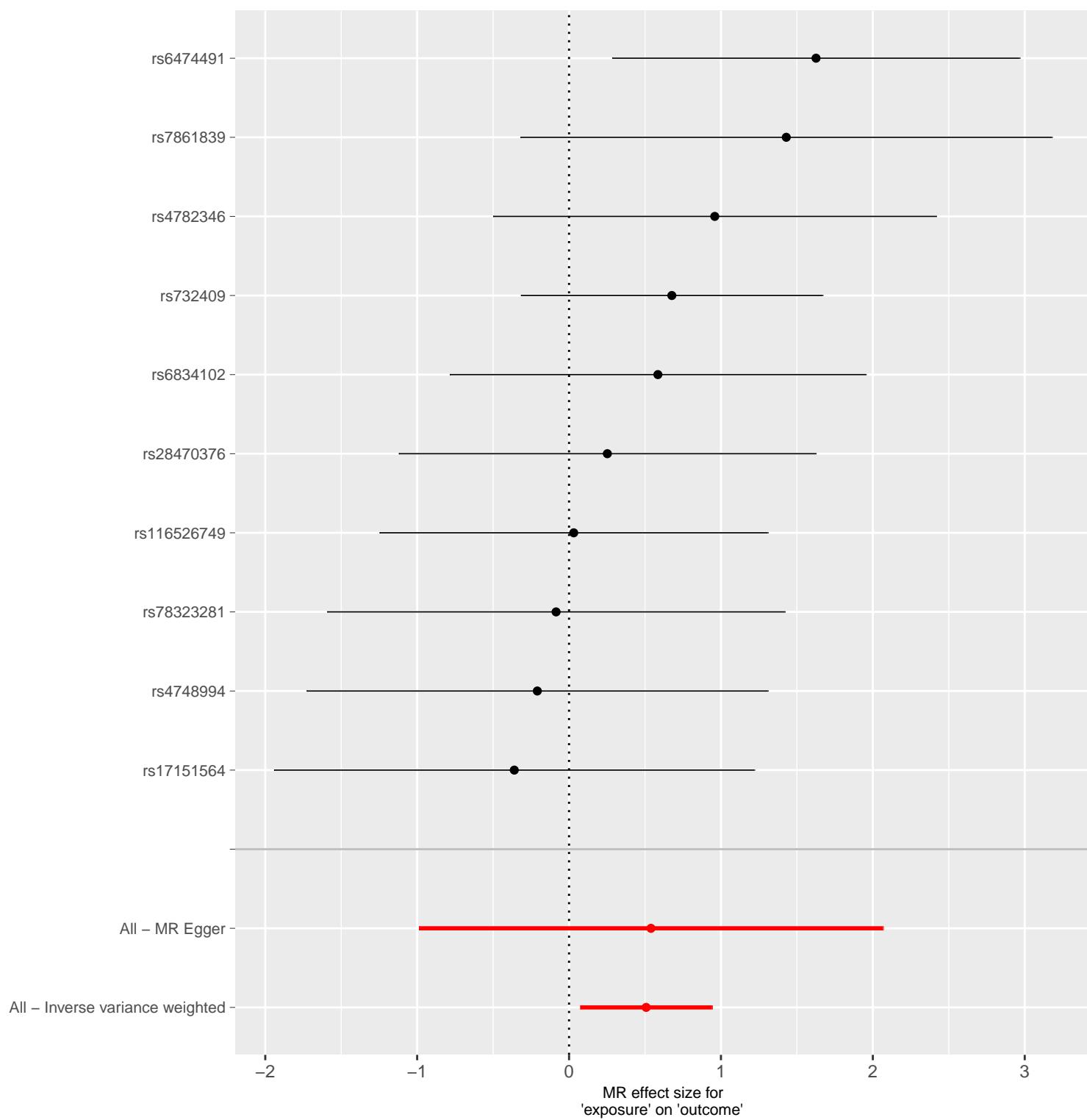

Supplement: Supplementary Data Sheet 2 — Full GCST identifiers, taxonomic labels, and Mendelian randomization statistics for the gut microbial traits associated with ulcerative colitis. [file DataSheet2.zip › GM_result/GCST90032527/forest.pdf]

# MR Method

Inverse variance weighted

MR Egger

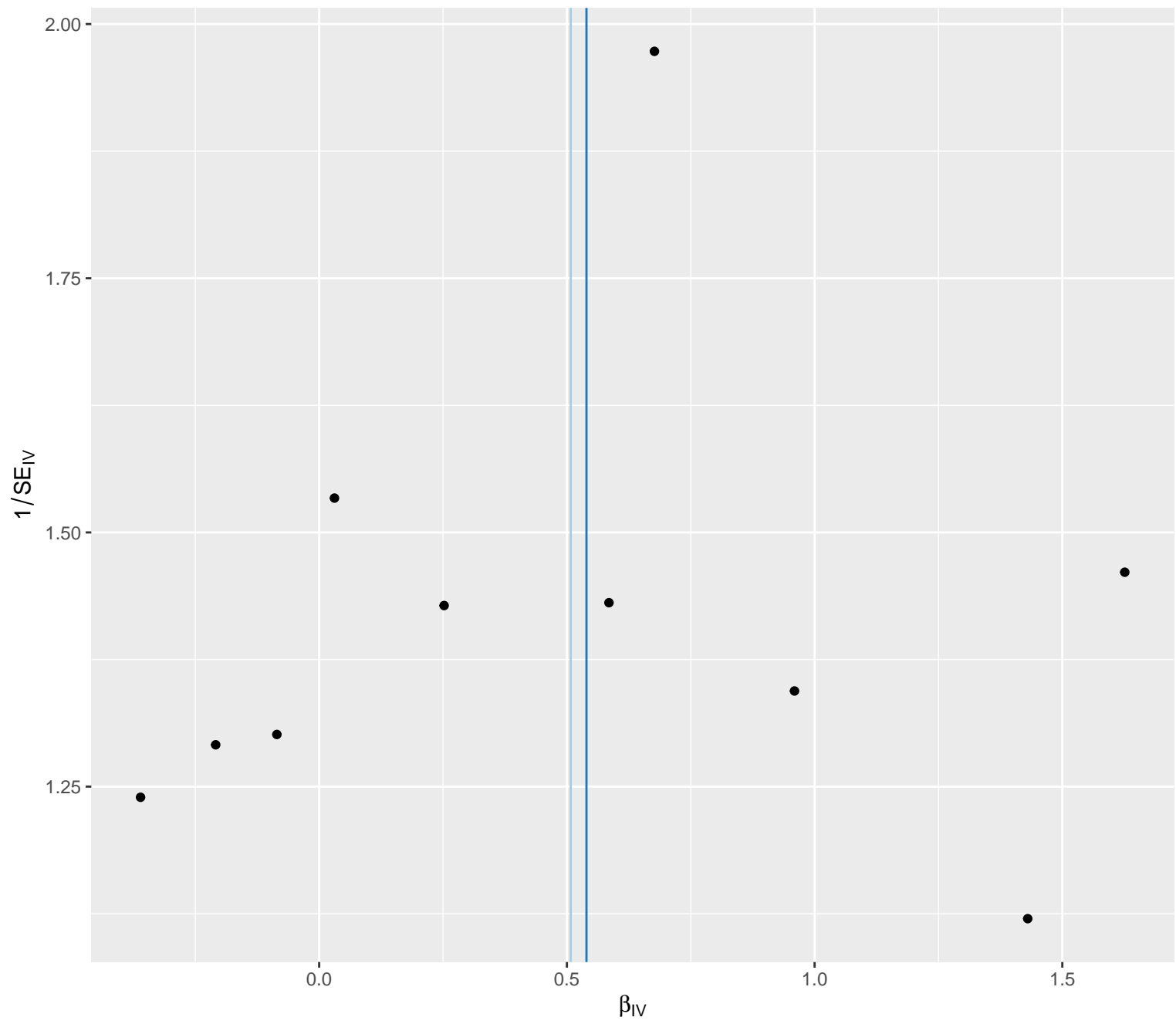

Supplement: Supplementary Data Sheet 2 — Full GCST identifiers, taxonomic labels, and Mendelian randomization statistics for the gut microbial traits associated with ulcerative colitis. [file DataSheet2.zip › GM_result/GCST90032527/funnelplot.pdf]

# MR Test

- Inverse variance weighted
- MR Egger
- Simple mode
- Weighted median
- Weighted mode

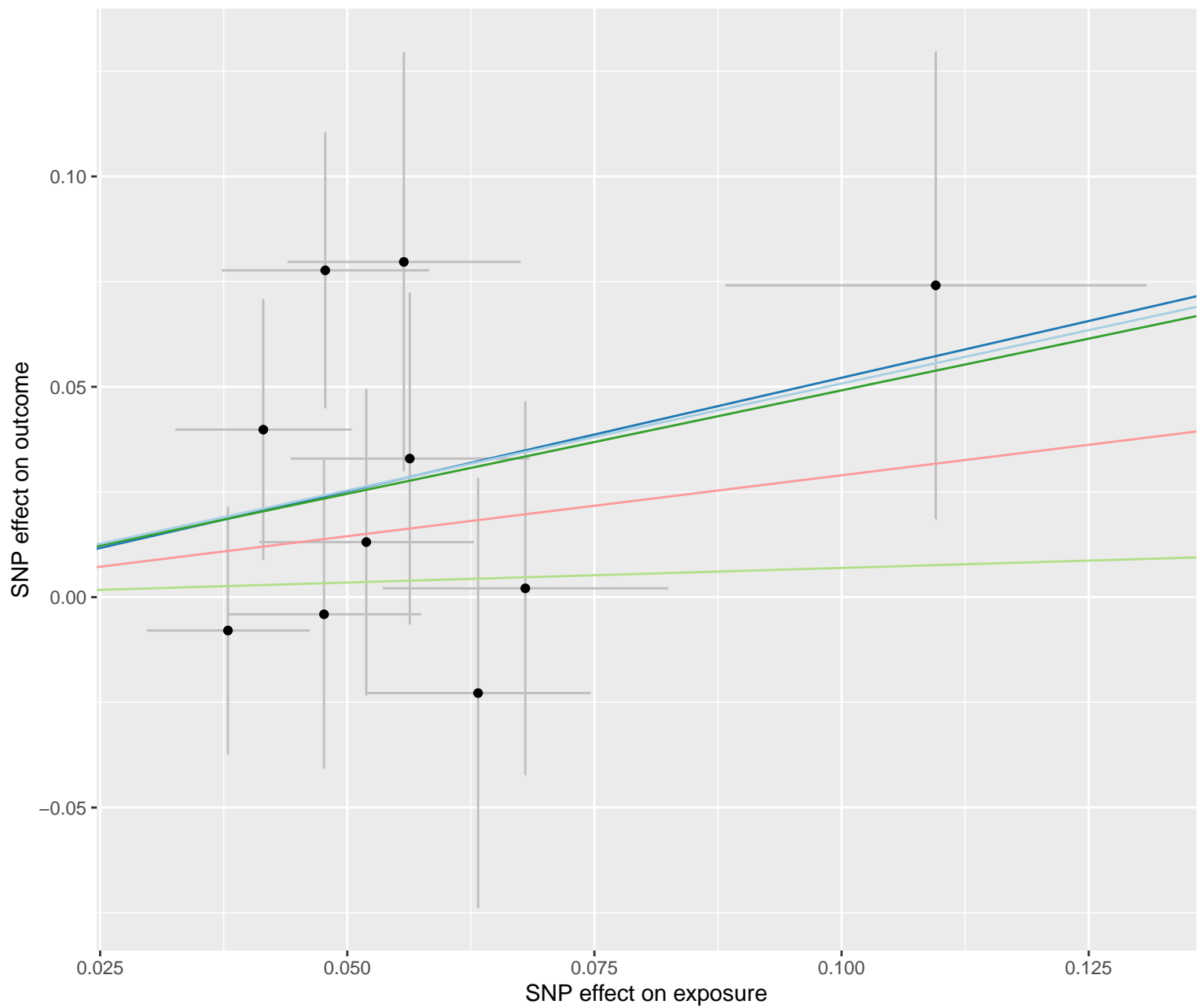

Supplement: Supplementary Data Sheet 2 — Full GCST identifiers, taxonomic labels, and Mendelian randomization statistics for the gut microbial traits associated with ulcerative colitis. [file DataSheet2.zip › GM_result/GCST90032527/scatter.pdf]

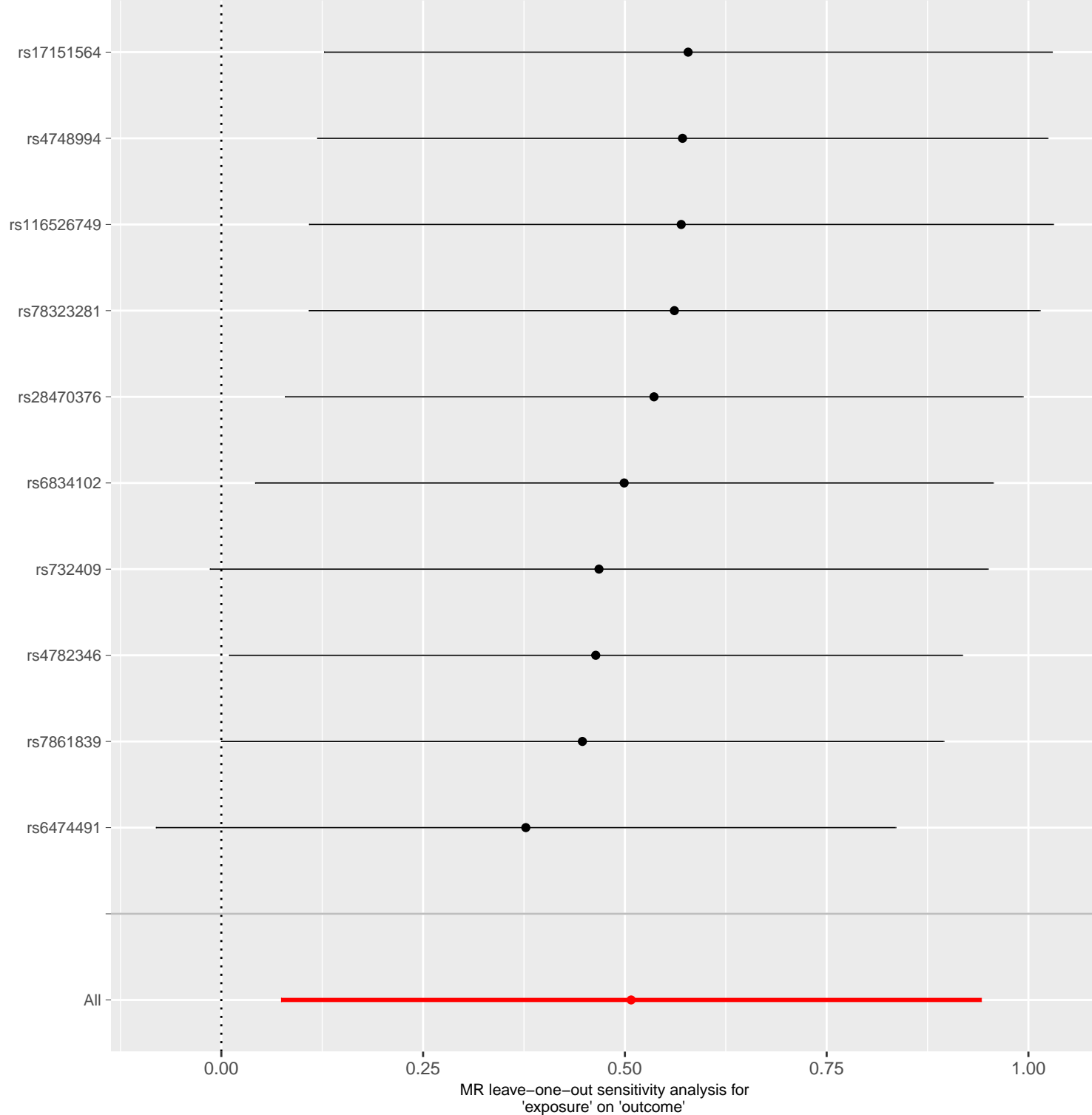

Supplement: Supplementary Data Sheet 2 — Full GCST identifiers, taxonomic labels, and Mendelian randomization statistics for the gut microbial traits associated with ulcerative colitis. [file DataSheet2.zip › GM_result/GCST90032527/sensitivity-analysis.pdf]

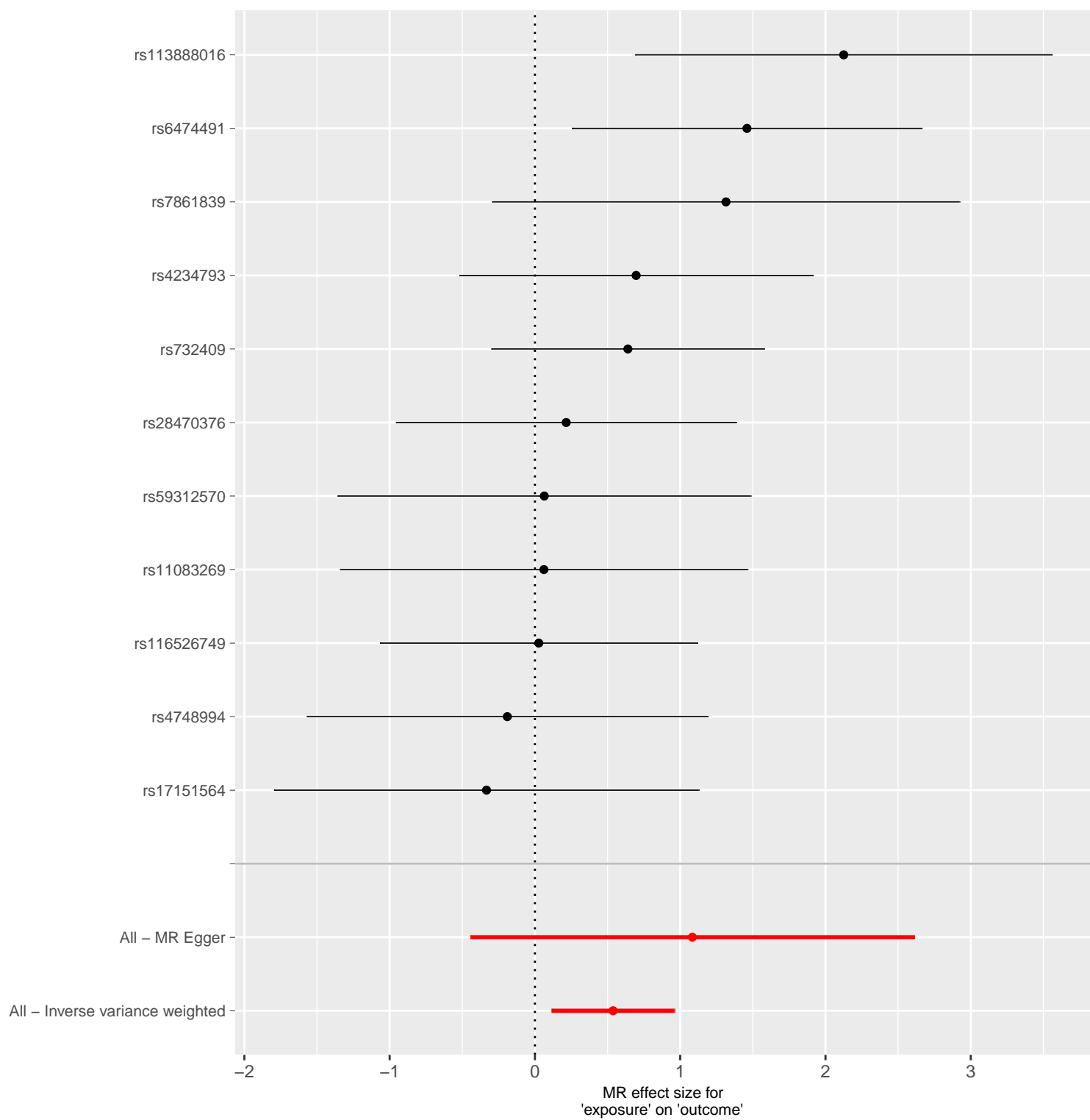

Supplement: Supplementary Data Sheet 2 — Full GCST identifiers, taxonomic labels, and Mendelian randomization statistics for the gut microbial traits associated with ulcerative colitis. [file DataSheet2.zip › GM_result/GCST90032528/forest.pdf]

# MR Method

- Inverse variance weighted
- MR Egger

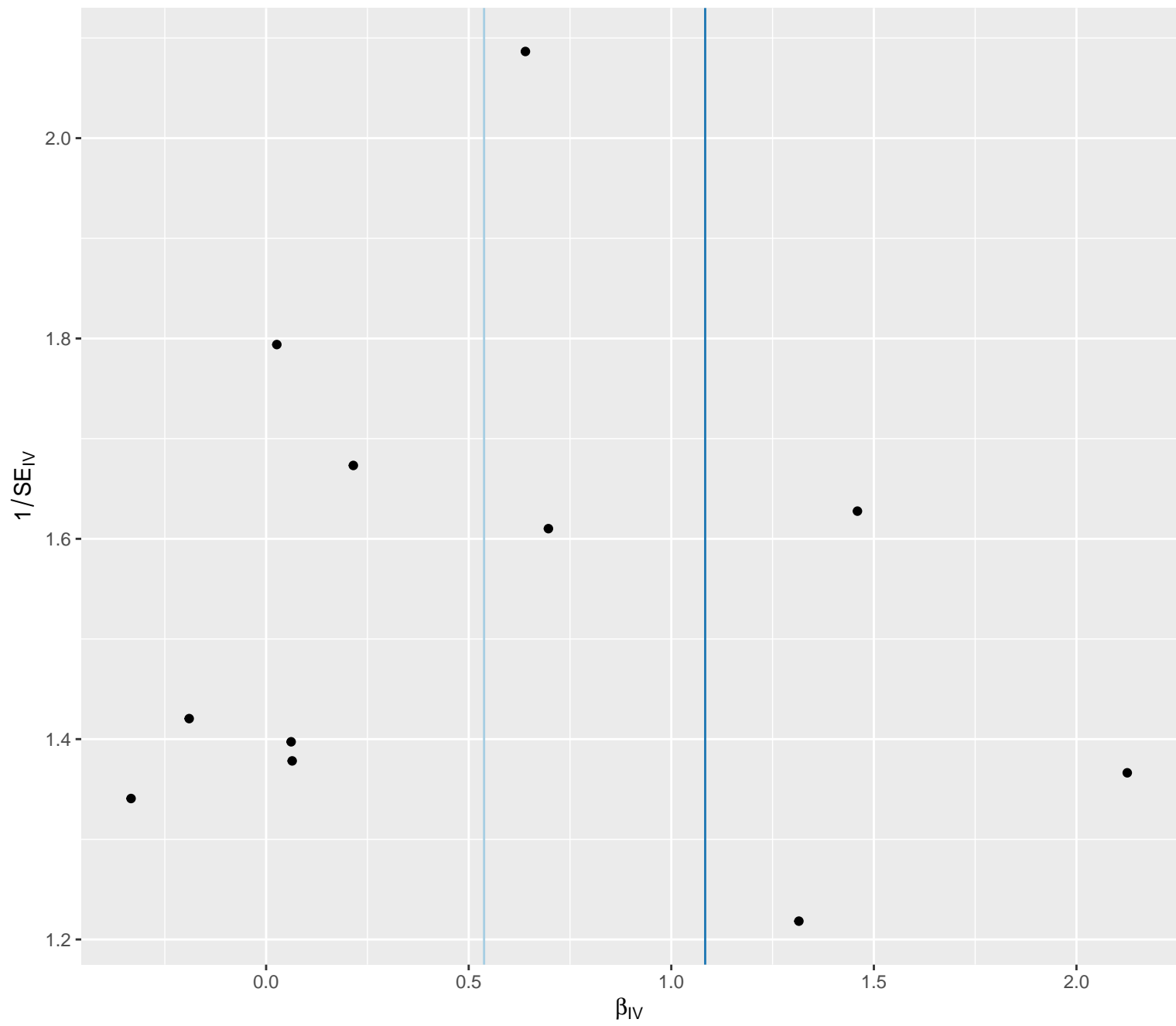

Supplement: Supplementary Data Sheet 2 — Full GCST identifiers, taxonomic labels, and Mendelian randomization statistics for the gut microbial traits associated with ulcerative colitis. [file DataSheet2.zip › GM_result/GCST90032528/funnelplot.pdf]

# MR Test

- Inverse variance weighted
- MR Egger
- Simple mode
- Weighted median
- Weighted mode

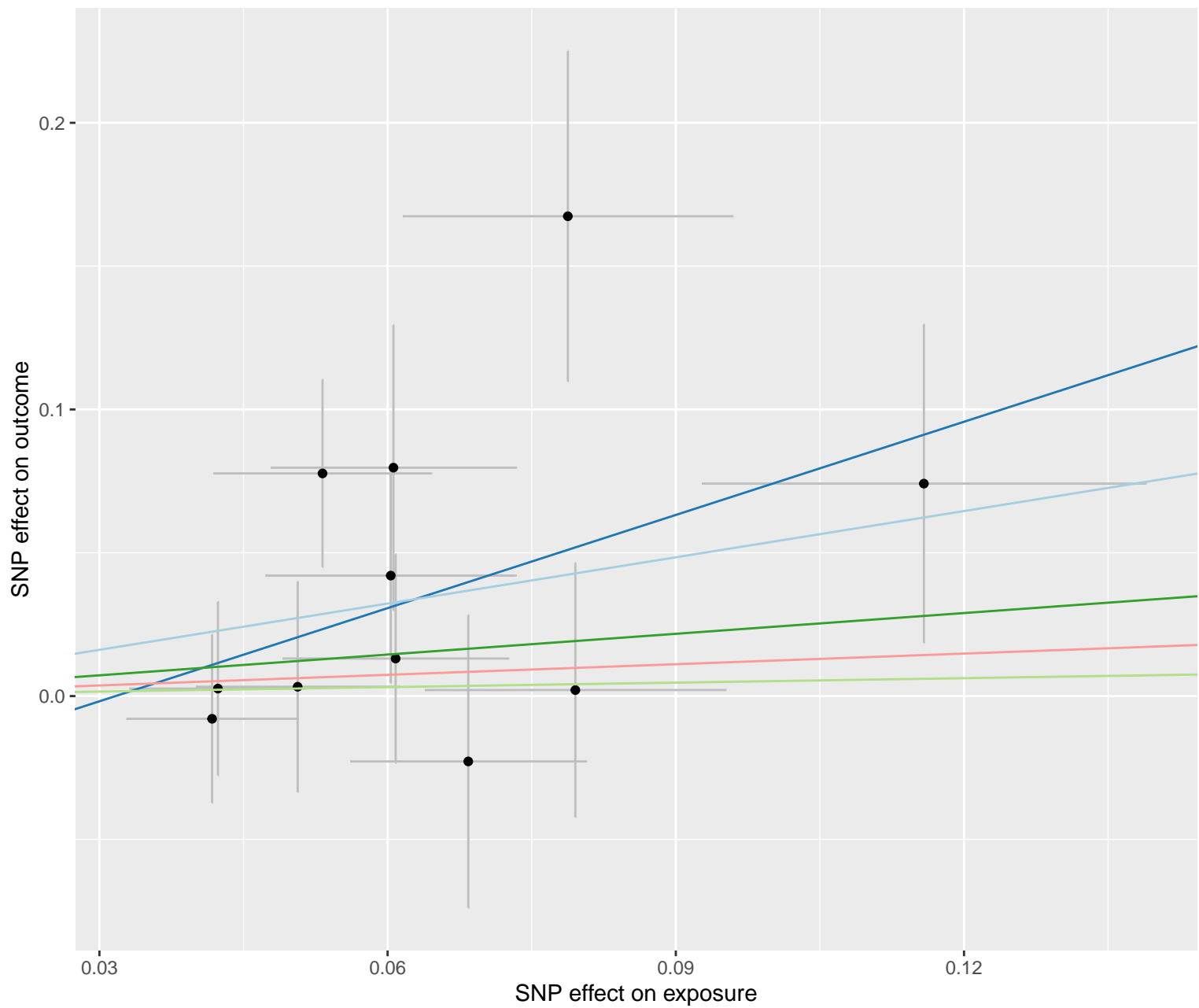

Supplement: Supplementary Data Sheet 2 — Full GCST identifiers, taxonomic labels, and Mendelian randomization statistics for the gut microbial traits associated with ulcerative colitis. [file DataSheet2.zip › GM_result/GCST90032528/scatter.pdf]

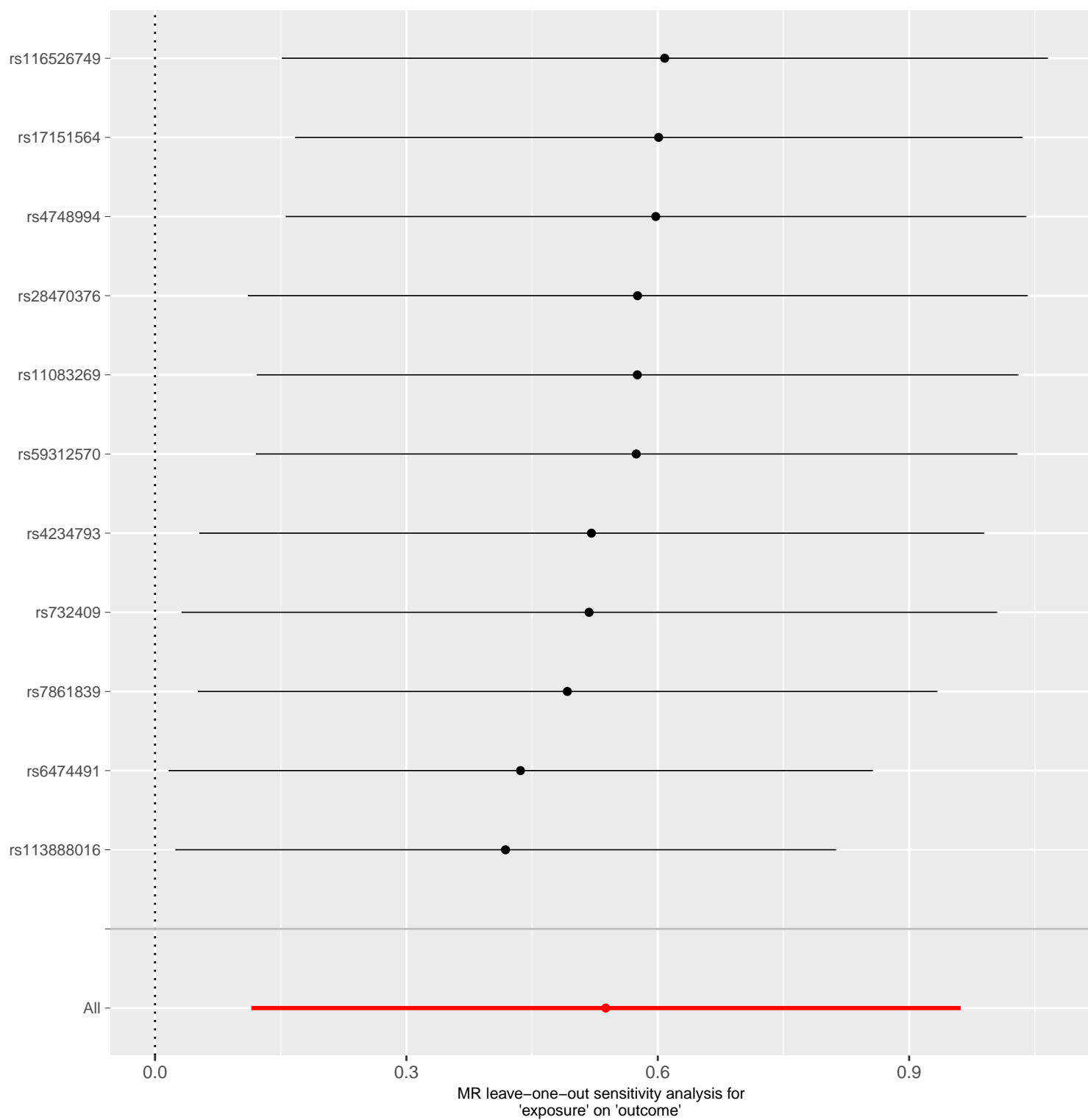

Supplement: Supplementary Data Sheet 2 — Full GCST identifiers, taxonomic labels, and Mendelian randomization statistics for the gut microbial traits associated with ulcerative colitis. [file DataSheet2.zip › GM_result/GCST90032528/sensitivity-analysis.pdf]

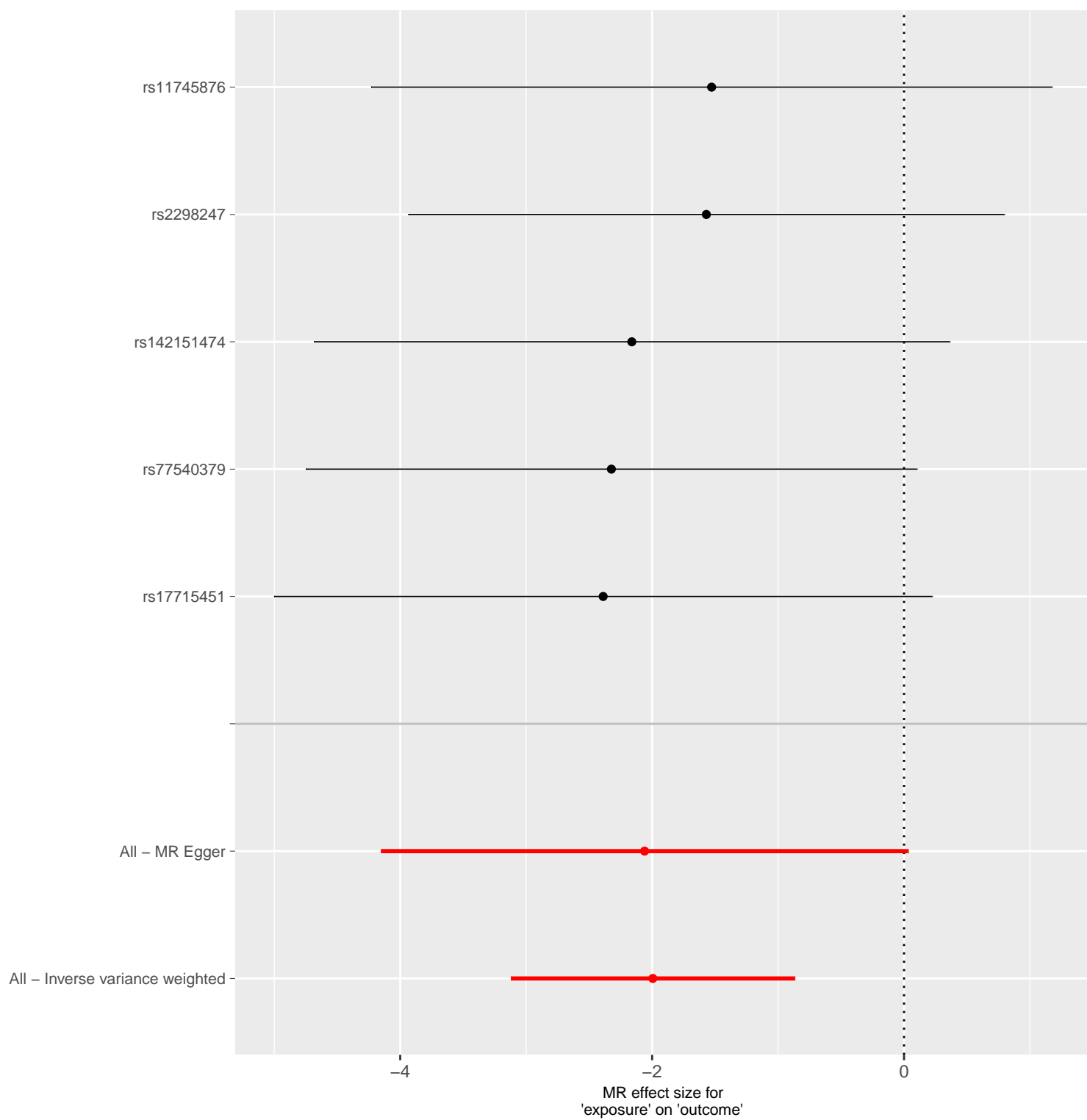

Supplement: Supplementary Data Sheet 2 — Full GCST identifiers, taxonomic labels, and Mendelian randomization statistics for the gut microbial traits associated with ulcerative colitis. [file DataSheet2.zip › GM_result/GCST90032583/forest.pdf]

# MR Method

- Inverse variance weighted
- MR Egger

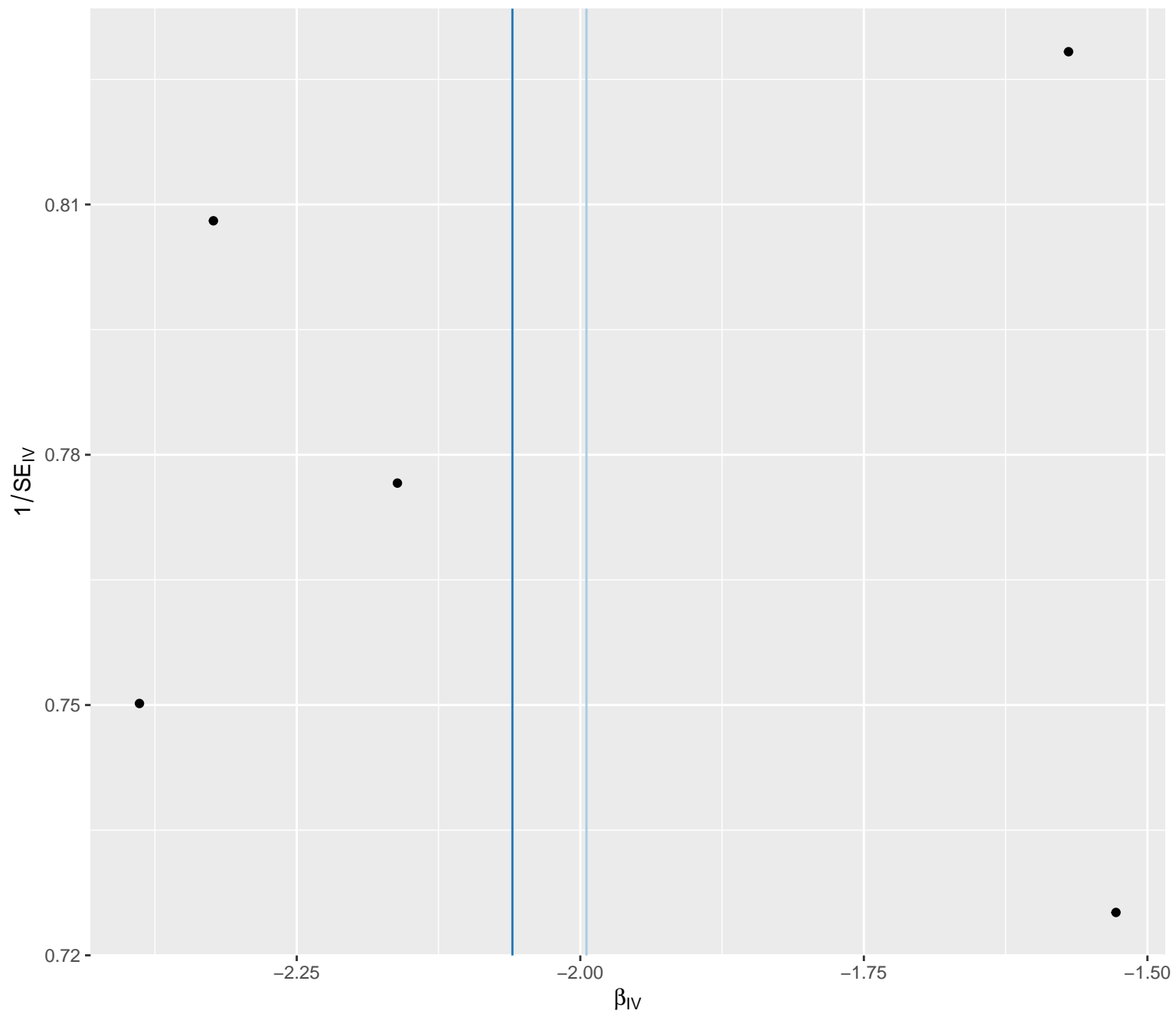

Supplement: Supplementary Data Sheet 2 — Full GCST identifiers, taxonomic labels, and Mendelian randomization statistics for the gut microbial traits associated with ulcerative colitis. [file DataSheet2.zip › GM_result/GCST90032583/funnelplot.pdf]

# MR Test

- Inverse variance weighted
- MR Egger
- Simple mode
- Weighted median
- Weighted mode

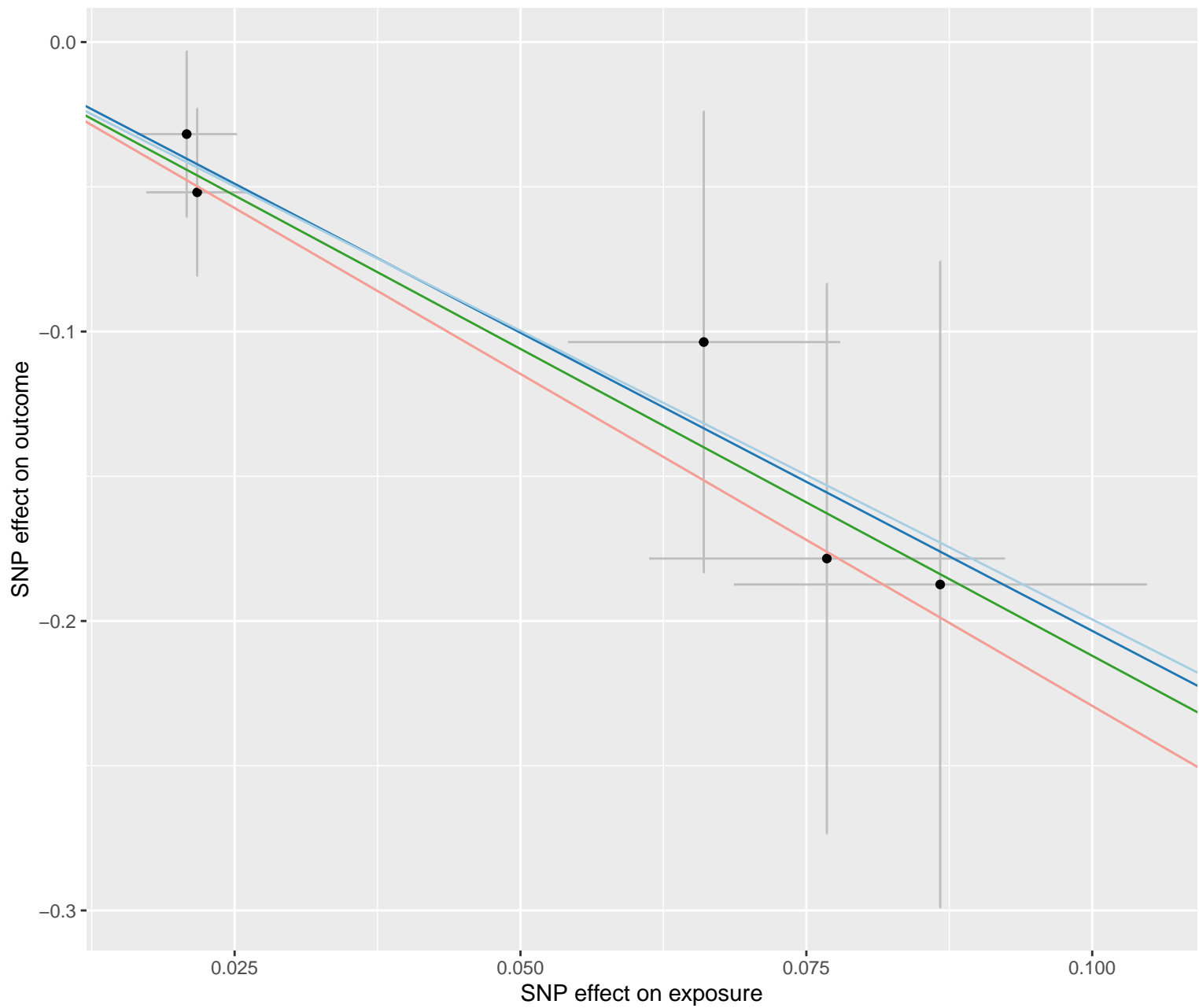

Supplement: Supplementary Data Sheet 2 — Full GCST identifiers, taxonomic labels, and Mendelian randomization statistics for the gut microbial traits associated with ulcerative colitis. [file DataSheet2.zip › GM_result/GCST90032583/scatter.pdf]

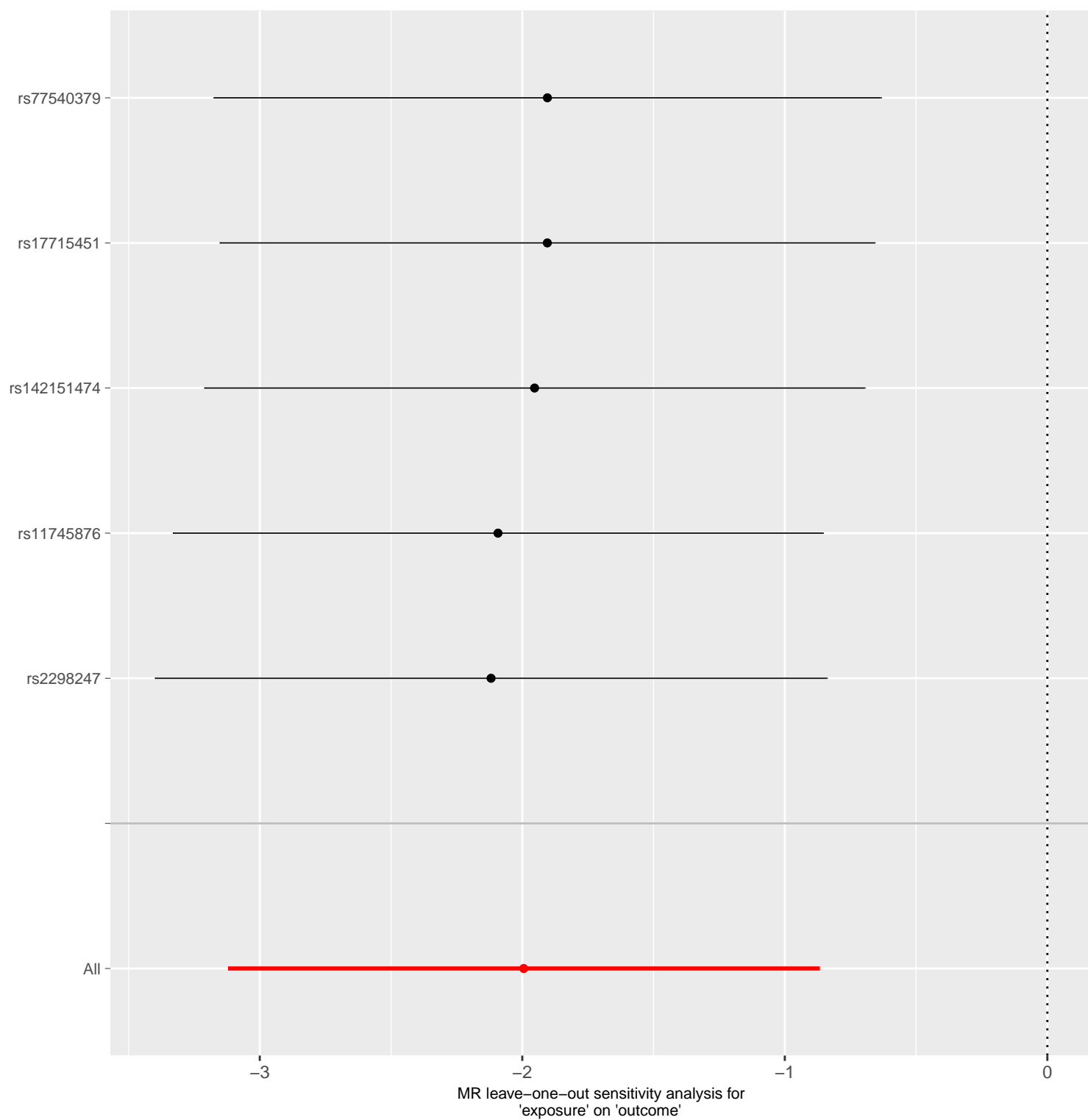

Supplement: Supplementary Data Sheet 2 — Full GCST identifiers, taxonomic labels, and Mendelian randomization statistics for the gut microbial traits associated with ulcerative colitis. [file DataSheet2.zip › GM_result/GCST90032583/sensitivity-analysis.pdf]

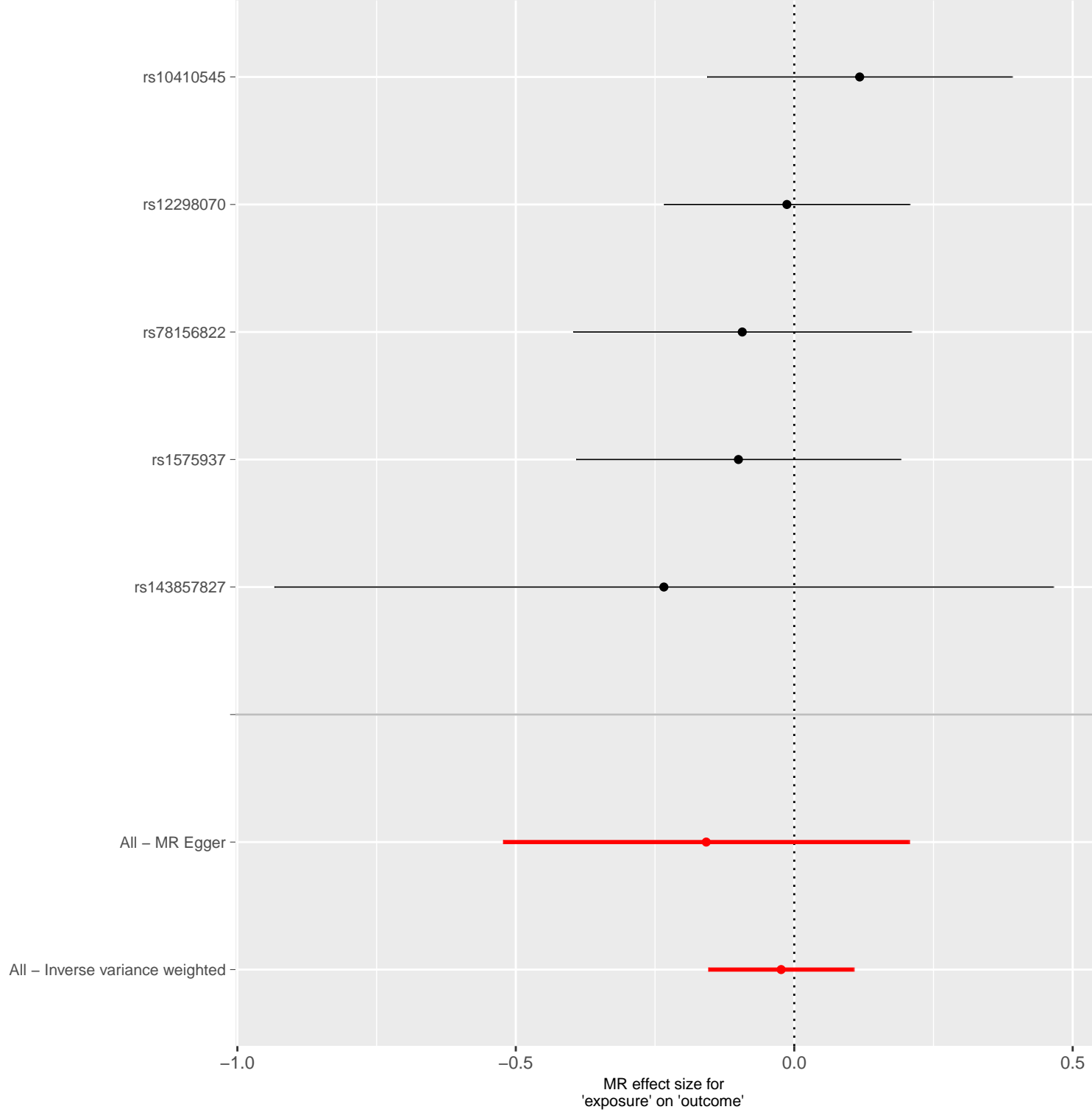

Supplement: Supplementary Data Sheet 3 — Full results of the pairwise Mendelian randomization analyses between ulcerative colitis-associated microbial taxa and ulcerative colitis-associated pyroptosis proteins, used for the downstream mediation analysis. [file DataSheet3.zip › GM_bd_fer_result/GCST90032466+11067_13_BGLAP_Osteocalcin/forest.pdf]

# MR Method

- Inverse variance weighted
- MR Egger

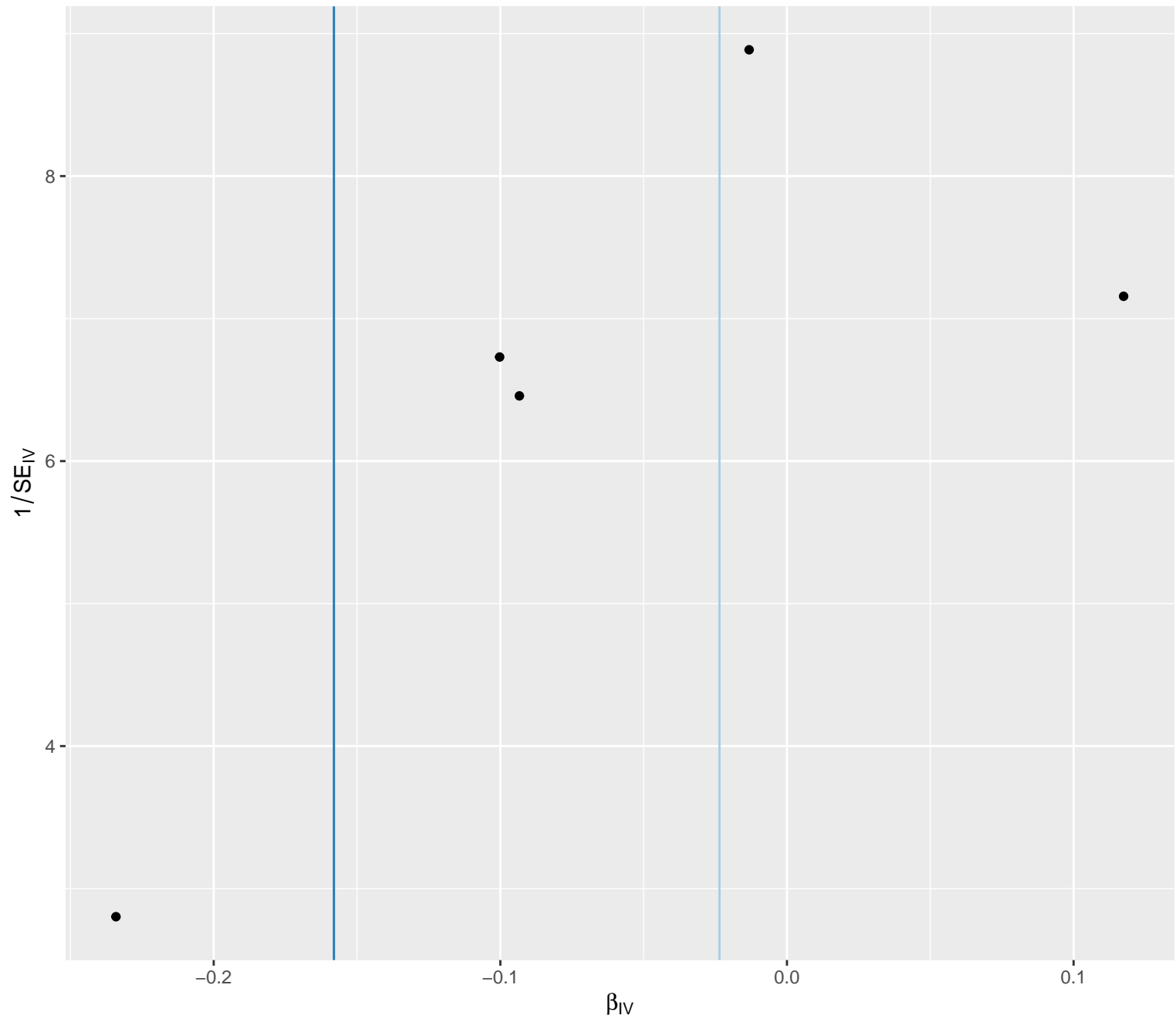

Supplement: Supplementary Data Sheet 3 — Full results of the pairwise Mendelian randomization analyses between ulcerative colitis-associated microbial taxa and ulcerative colitis-associated pyroptosis proteins, used for the downstream mediation analysis. [file DataSheet3.zip › GM_bd_fer_result/GCST90032466+11067_13_BGLAP_Osteocalcin/funnelplot.pdf]

# MR Test

- Inverse variance weighted
- MR Egger
- Simple mode
- Weighted median
- Weighted mode

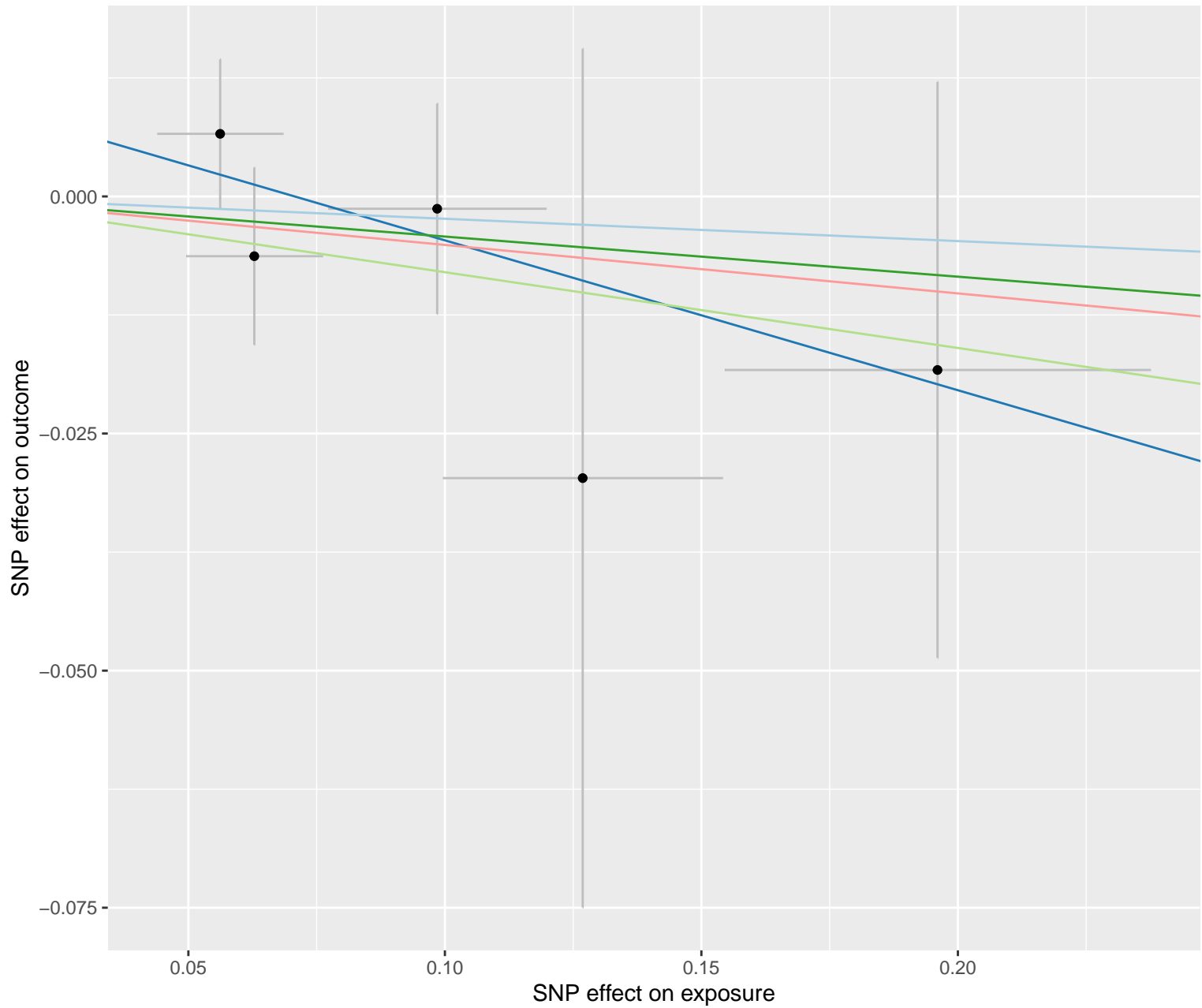

Supplement: Supplementary Data Sheet 3 — Full results of the pairwise Mendelian randomization analyses between ulcerative colitis-associated microbial taxa and ulcerative colitis-associated pyroptosis proteins, used for the downstream mediation analysis. [file DataSheet3.zip › GM_bd_fer_result/GCST90032466+11067_13_BGLAP_Osteocalcin/scatter.pdf]

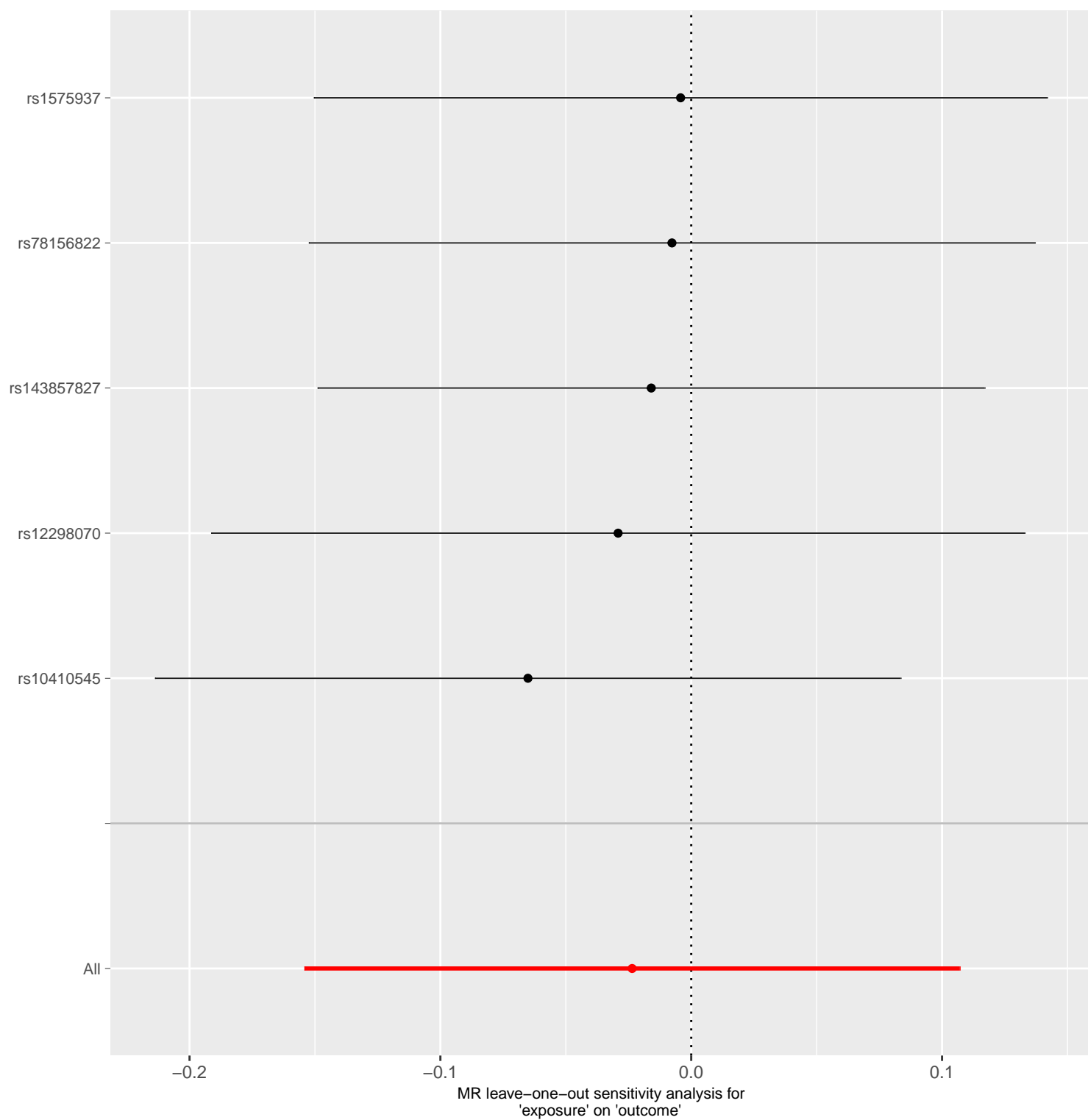

Supplement: Supplementary Data Sheet 3 — Full results of the pairwise Mendelian randomization analyses between ulcerative colitis-associated microbial taxa and ulcerative colitis-associated pyroptosis proteins, used for the downstream mediation analysis. [file DataSheet3.zip › GM_bd_fer_result/GCST90032466+11067_13_BGLAP_Osteocalcin/sensitivity-analysis.pdf]

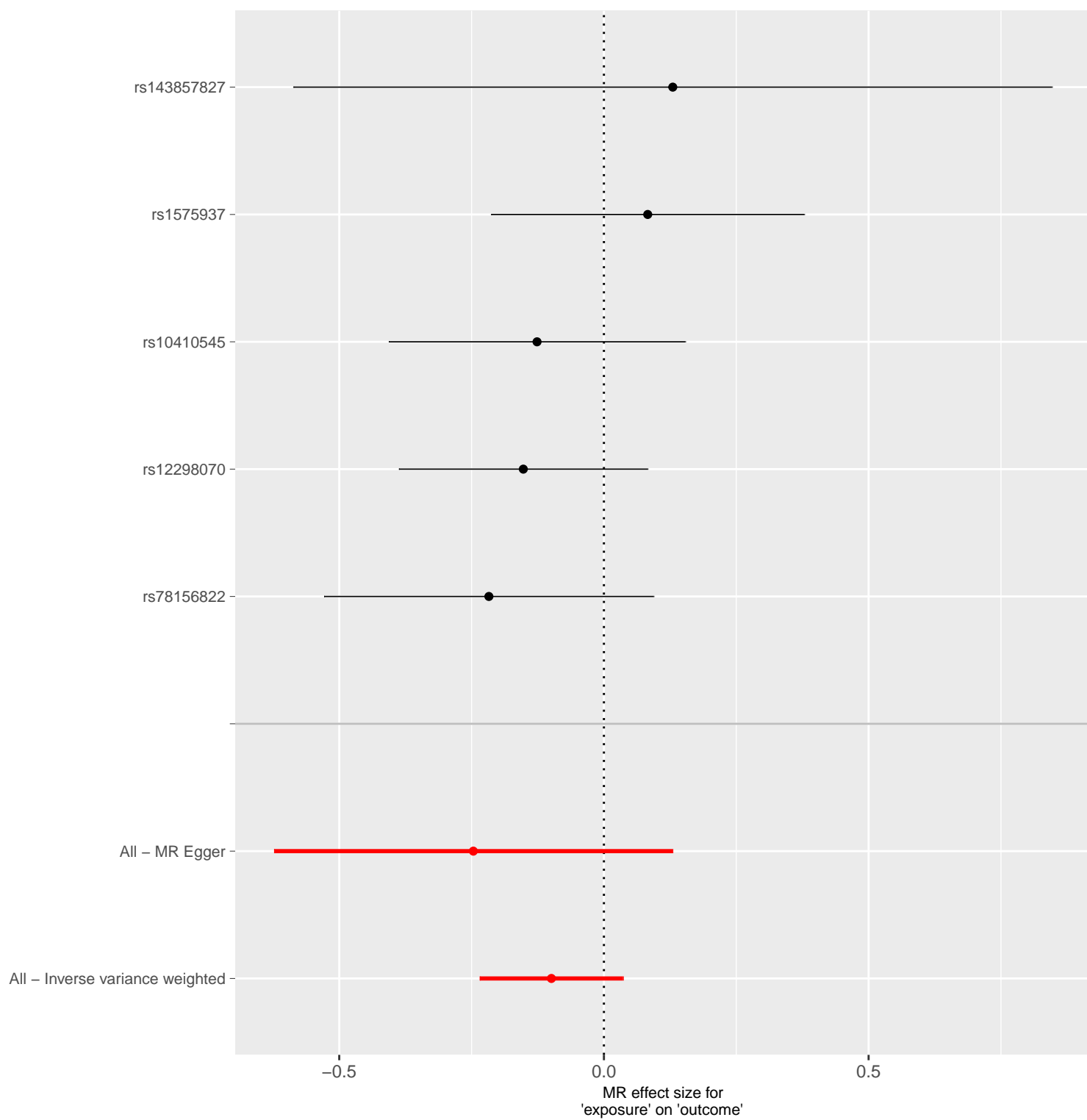

Supplement: Supplementary Data Sheet 3 — Full results of the pairwise Mendelian randomization analyses between ulcerative colitis-associated microbial taxa and ulcerative colitis-associated pyroptosis proteins, used for the downstream mediation analysis. [file DataSheet3.zip › GM_bd_fer_result/GCST90032466+12332_7_EEF2K_EF2K/forest.pdf]

# MR Method

- Inverse variance weighted
- MR Egger

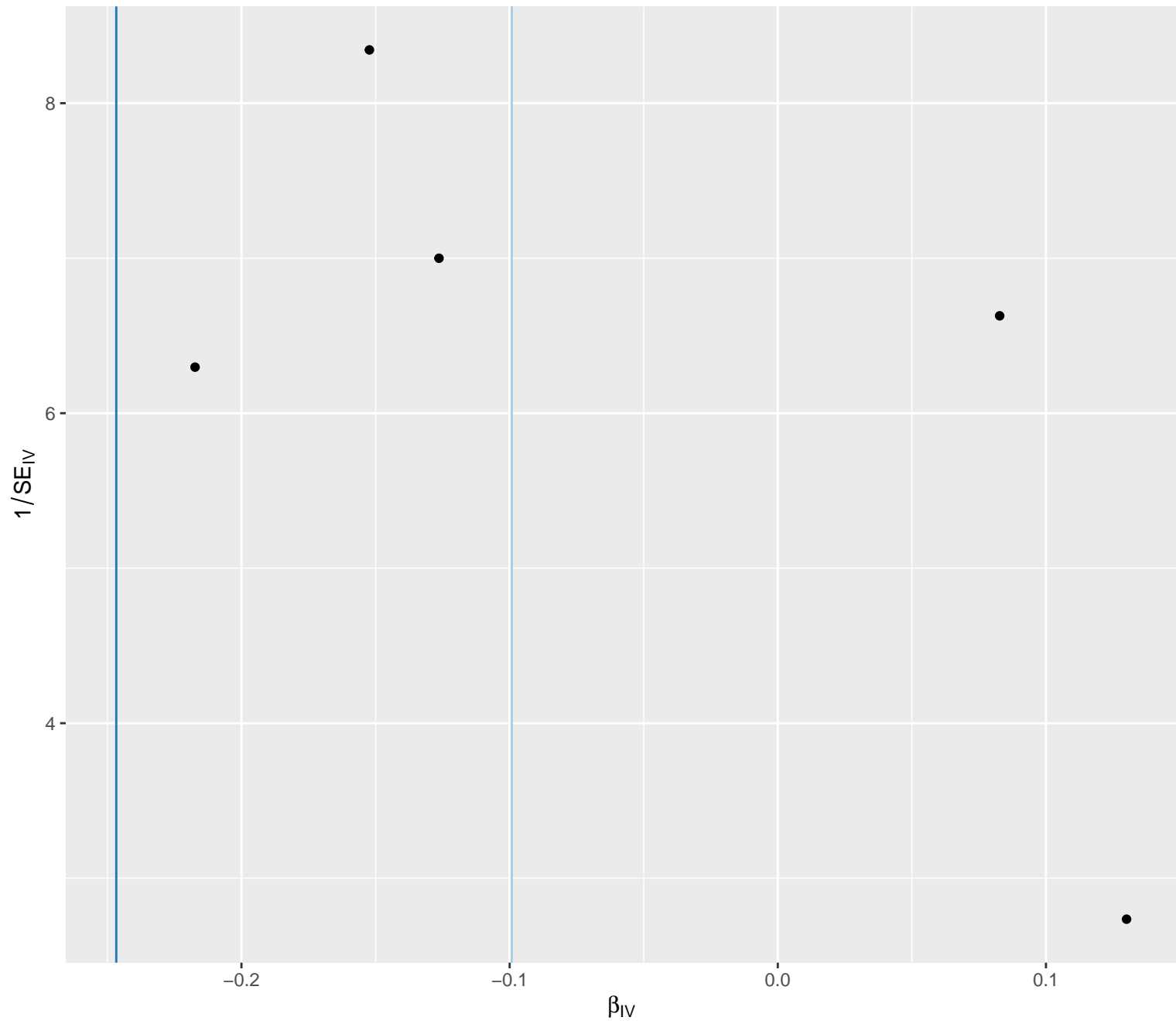

Supplement: Supplementary Data Sheet 3 — Full results of the pairwise Mendelian randomization analyses between ulcerative colitis-associated microbial taxa and ulcerative colitis-associated pyroptosis proteins, used for the downstream mediation analysis. [file DataSheet3.zip › GM_bd_fer_result/GCST90032466+12332_7_EEF2K_EF2K/funnelplot.pdf]

# MR Test

- Inverse variance weighted
- MR Egger
- Simple mode
- Weighted median
- Weighted mode

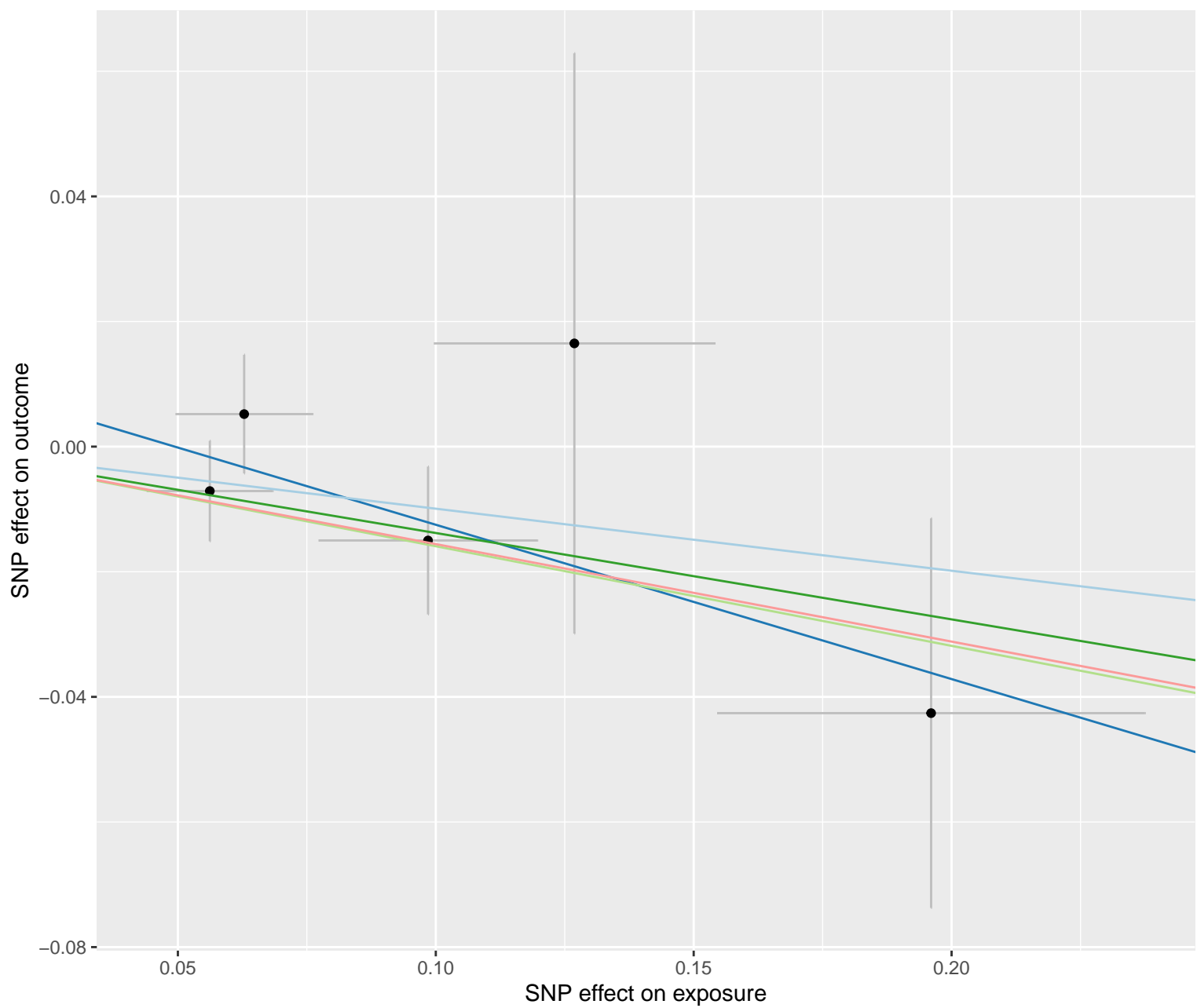

Supplement: Supplementary Data Sheet 3 — Full results of the pairwise Mendelian randomization analyses between ulcerative colitis-associated microbial taxa and ulcerative colitis-associated pyroptosis proteins, used for the downstream mediation analysis. [file DataSheet3.zip › GM_bd_fer_result/GCST90032466+12332_7_EEF2K_EF2K/scatter.pdf]

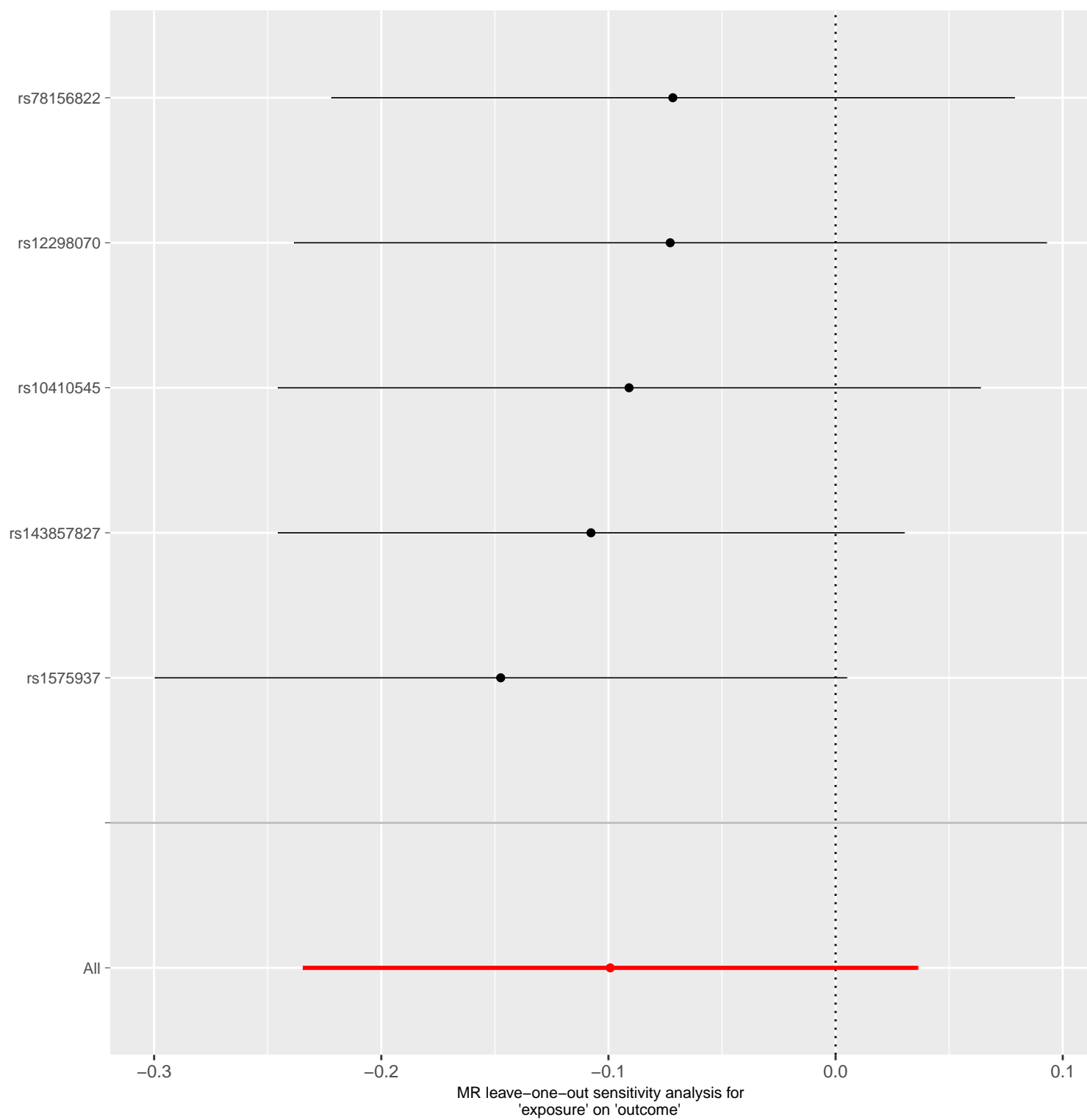

Supplement: Supplementary Data Sheet 3 — Full results of the pairwise Mendelian randomization analyses between ulcerative colitis-associated microbial taxa and ulcerative colitis-associated pyroptosis proteins, used for the downstream mediation analysis. [file DataSheet3.zip › GM_bd_fer_result/GCST90032466+12332_7_EEF2K_EF2K/sensitivity-analysis.pdf]

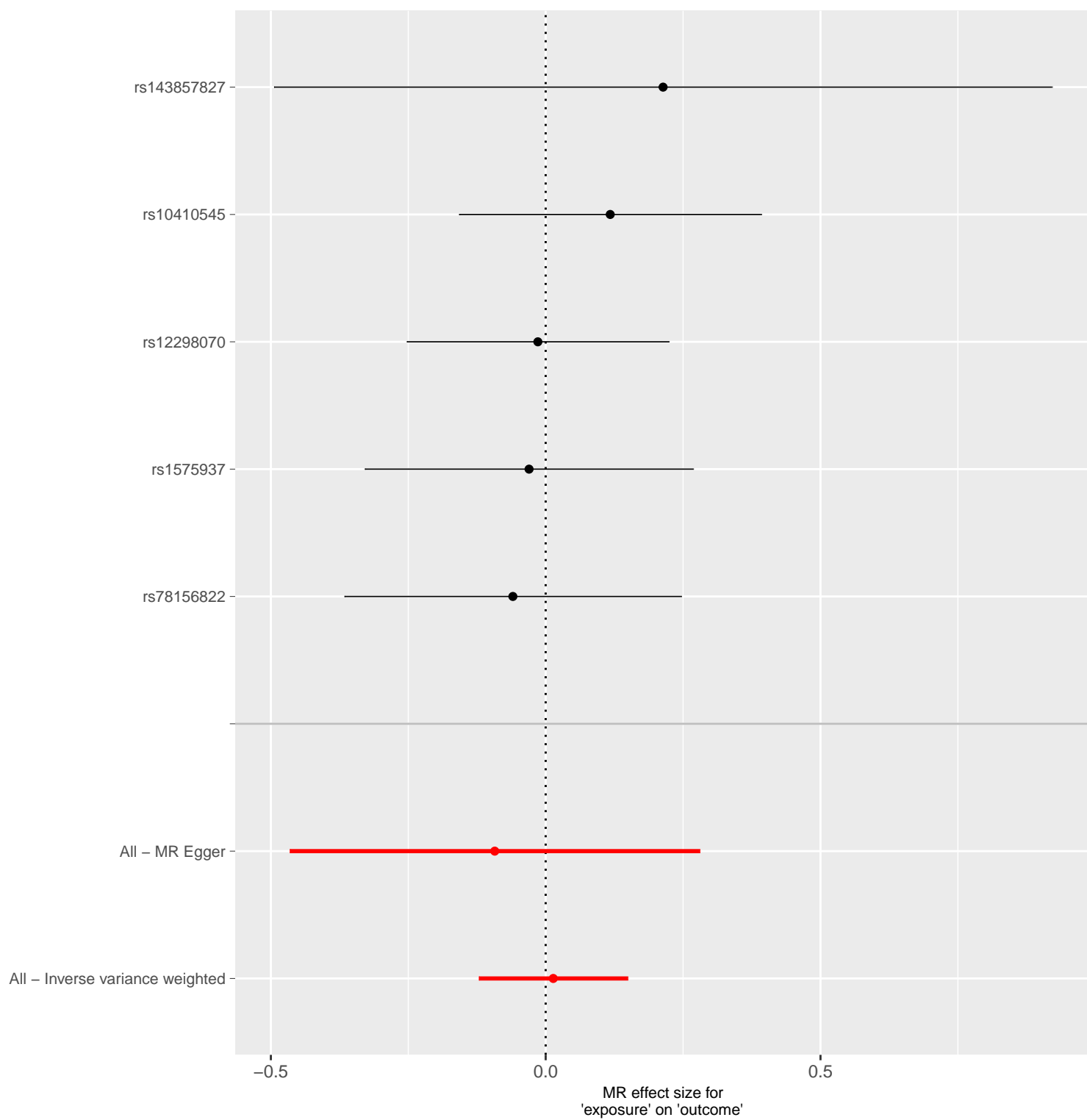

Supplement: Supplementary Data Sheet 3 — Full results of the pairwise Mendelian randomization analyses between ulcerative colitis-associated microbial taxa and ulcerative colitis-associated pyroptosis proteins, used for the downstream mediation analysis. [file DataSheet3.zip › GM_bd_fer_result/GCST90032466+12439_67_IRF9_ISGF3/forest.pdf]

# MR Method

- Inverse variance weighted
- MR Egger

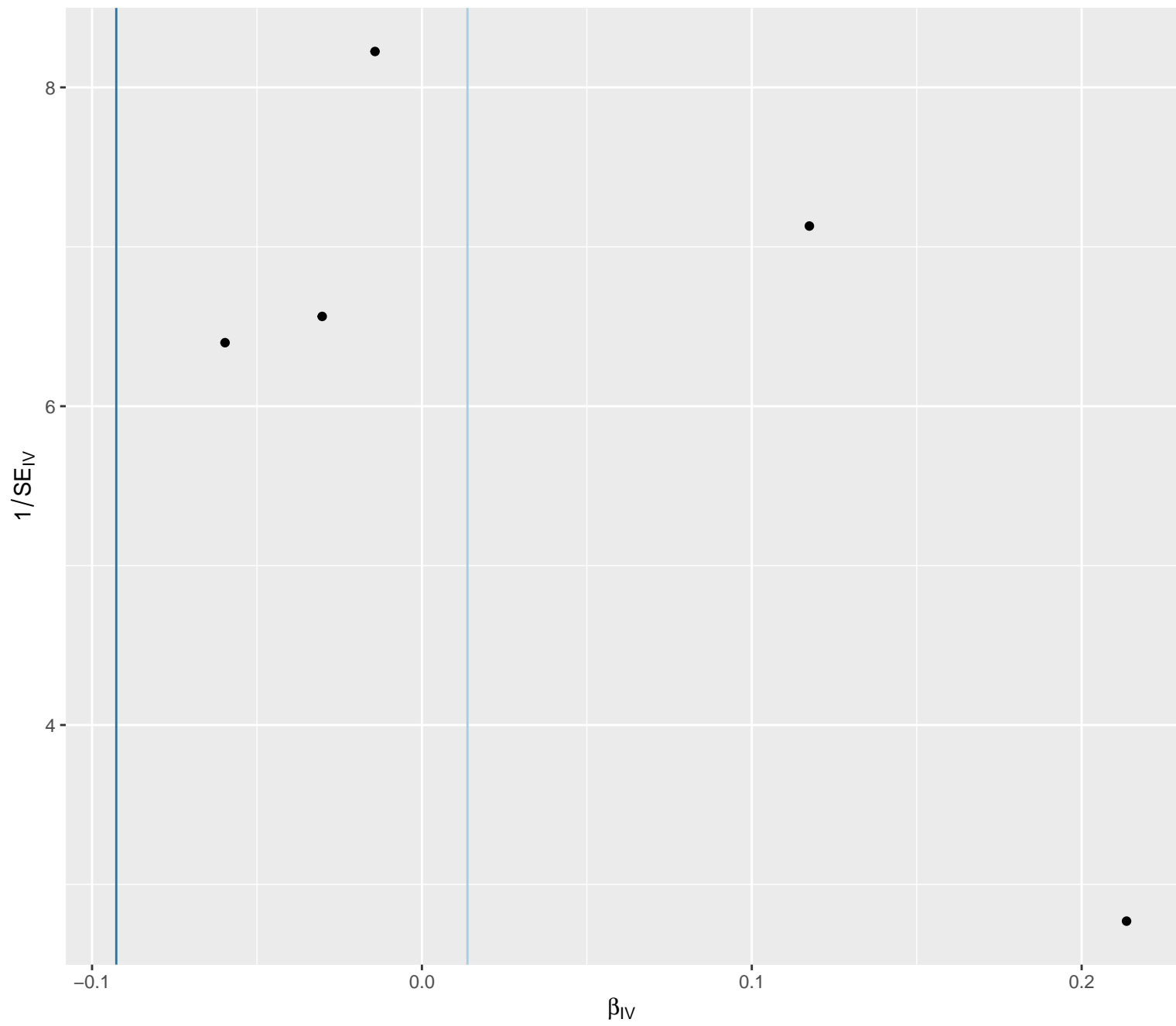

Supplement: Supplementary Data Sheet 3 — Full results of the pairwise Mendelian randomization analyses between ulcerative colitis-associated microbial taxa and ulcerative colitis-associated pyroptosis proteins, used for the downstream mediation analysis. [file DataSheet3.zip › GM_bd_fer_result/GCST90032466+12439_67_IRF9_ISGF3/funnelplot.pdf]

# MR Test

- Inverse variance weighted
- MR Egger
- Simple mode
- Weighted median
- Weighted mode

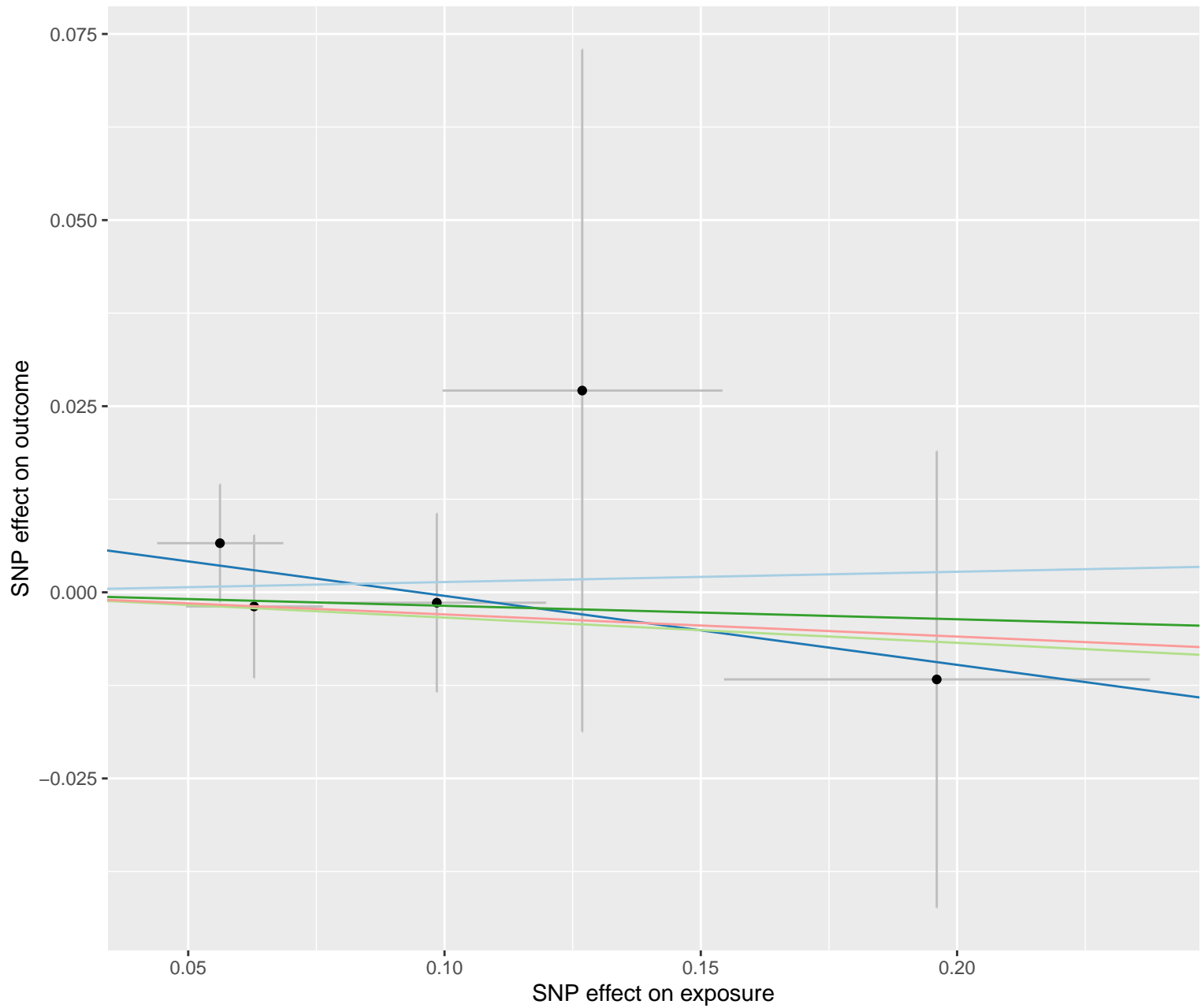

Supplement: Supplementary Data Sheet 3 — Full results of the pairwise Mendelian randomization analyses between ulcerative colitis-associated microbial taxa and ulcerative colitis-associated pyroptosis proteins, used for the downstream mediation analysis. [file DataSheet3.zip › GM_bd_fer_result/GCST90032466+12439_67_IRF9_ISGF3/scatter.pdf]

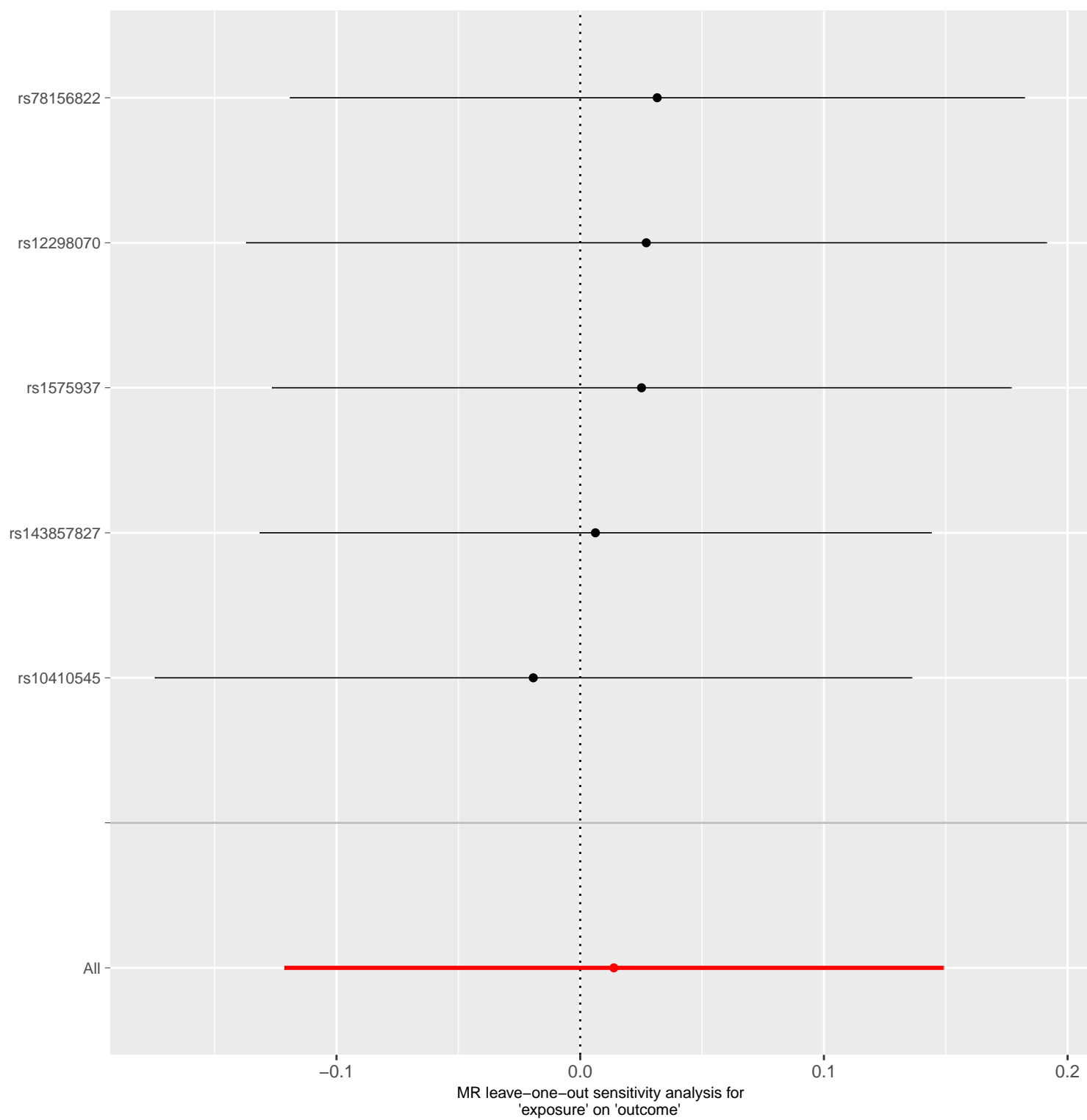

Supplement: Supplementary Data Sheet 3 — Full results of the pairwise Mendelian randomization analyses between ulcerative colitis-associated microbial taxa and ulcerative colitis-associated pyroptosis proteins, used for the downstream mediation analysis. [file DataSheet3.zip › GM_bd_fer_result/GCST90032466+12439_67_IRF9_ISGF3/sensitivity-analysis.pdf]

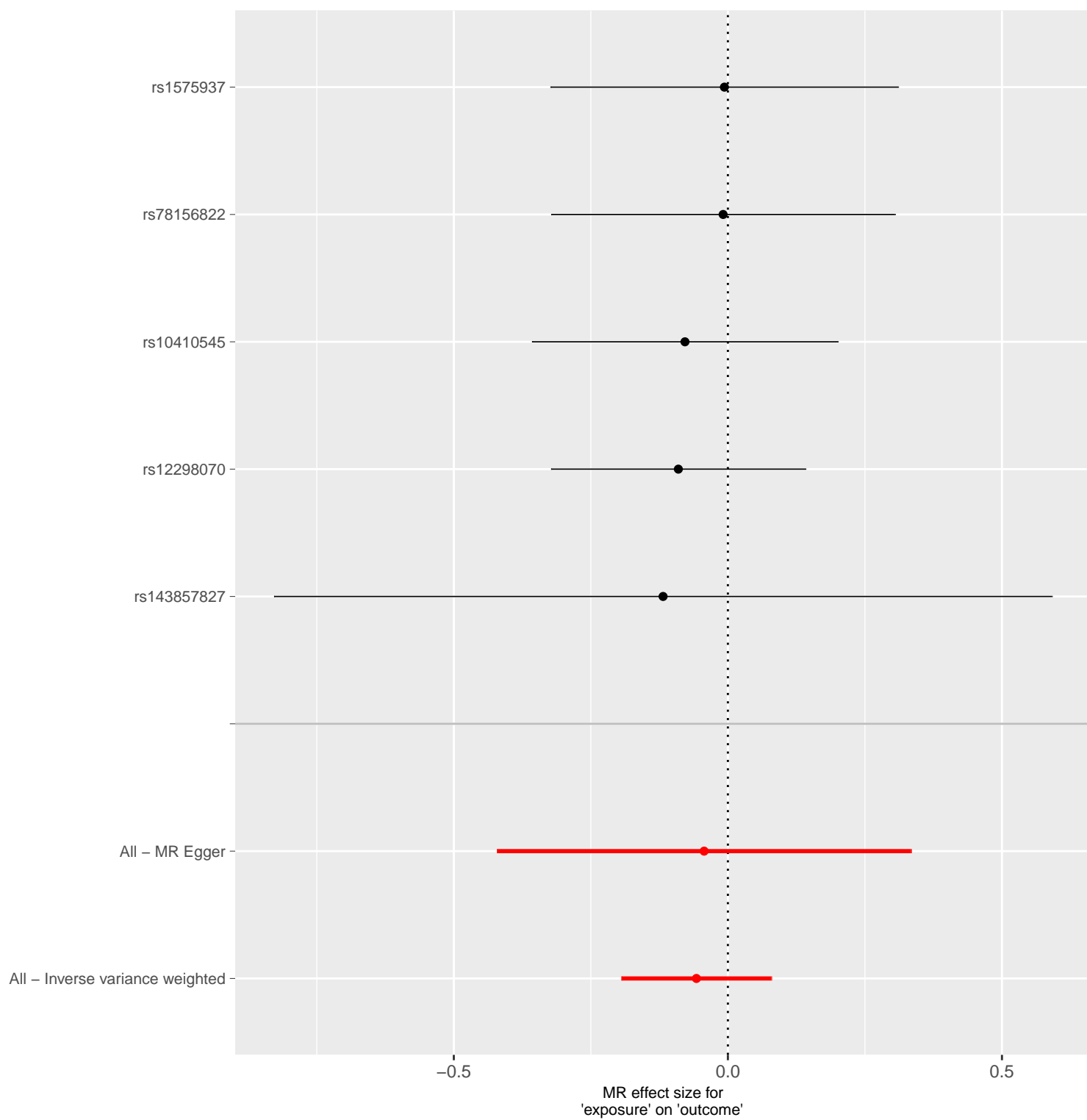

Supplement: Supplementary Data Sheet 3 — Full results of the pairwise Mendelian randomization analyses between ulcerative colitis-associated microbial taxa and ulcerative colitis-associated pyroptosis proteins, used for the downstream mediation analysis. [file DataSheet3.zip › GM_bd_fer_result/GCST90032466+13032_1_BECN1_BECN1/forest.pdf]

# MR Method

- Inverse variance weighted
- MR Egger

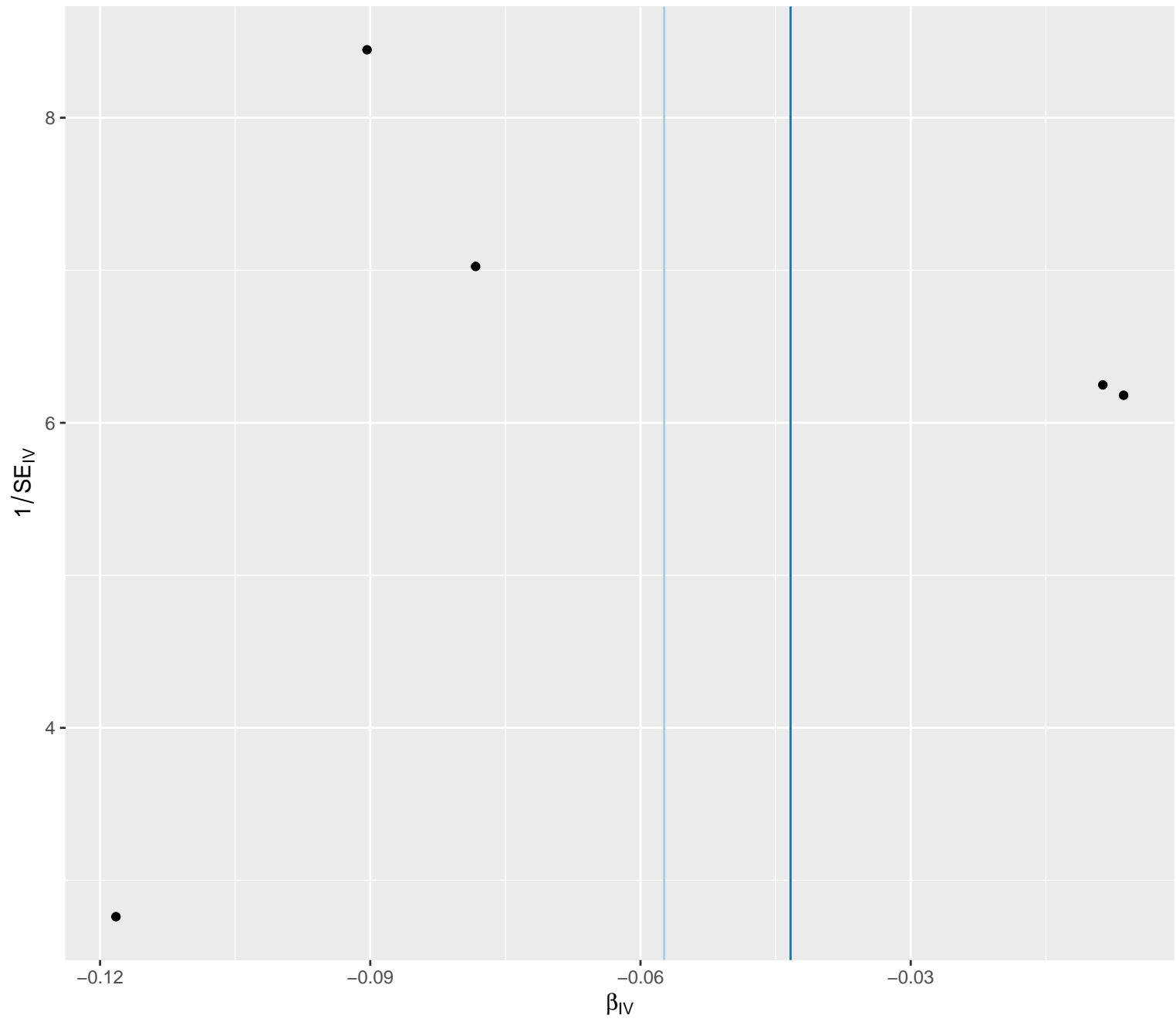

Supplement: Supplementary Data Sheet 3 — Full results of the pairwise Mendelian randomization analyses between ulcerative colitis-associated microbial taxa and ulcerative colitis-associated pyroptosis proteins, used for the downstream mediation analysis. [file DataSheet3.zip › GM_bd_fer_result/GCST90032466+13032_1_BECN1_BECN1/funnelplot.pdf]

# MR Test

- Inverse variance weighted
- MR Egger
- Simple mode
- Weighted median
- Weighted mode

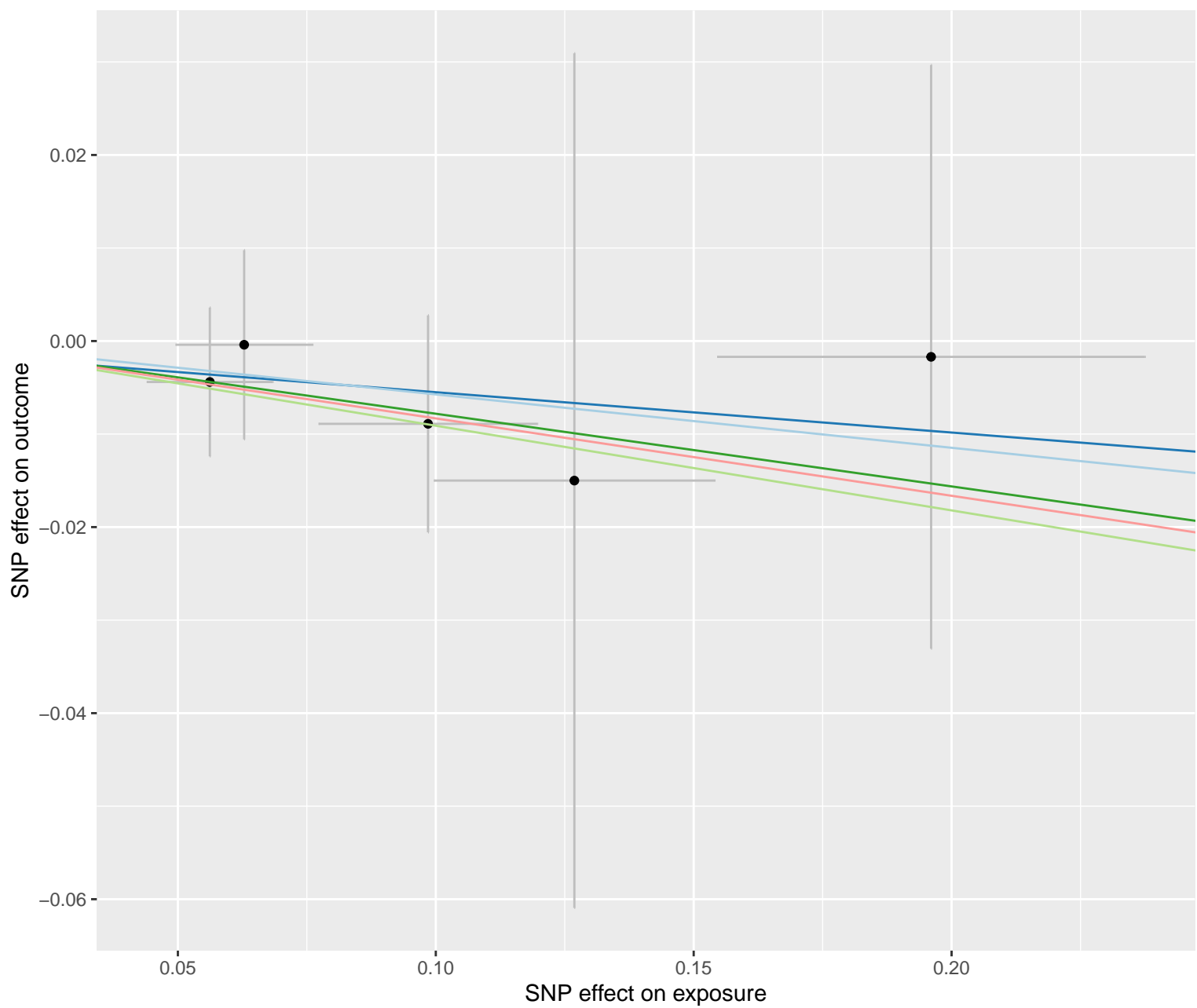

Supplement: Supplementary Data Sheet 3 — Full results of the pairwise Mendelian randomization analyses between ulcerative colitis-associated microbial taxa and ulcerative colitis-associated pyroptosis proteins, used for the downstream mediation analysis. [file DataSheet3.zip › GM_bd_fer_result/GCST90032466+13032_1_BECN1_BECN1/scatter.pdf]

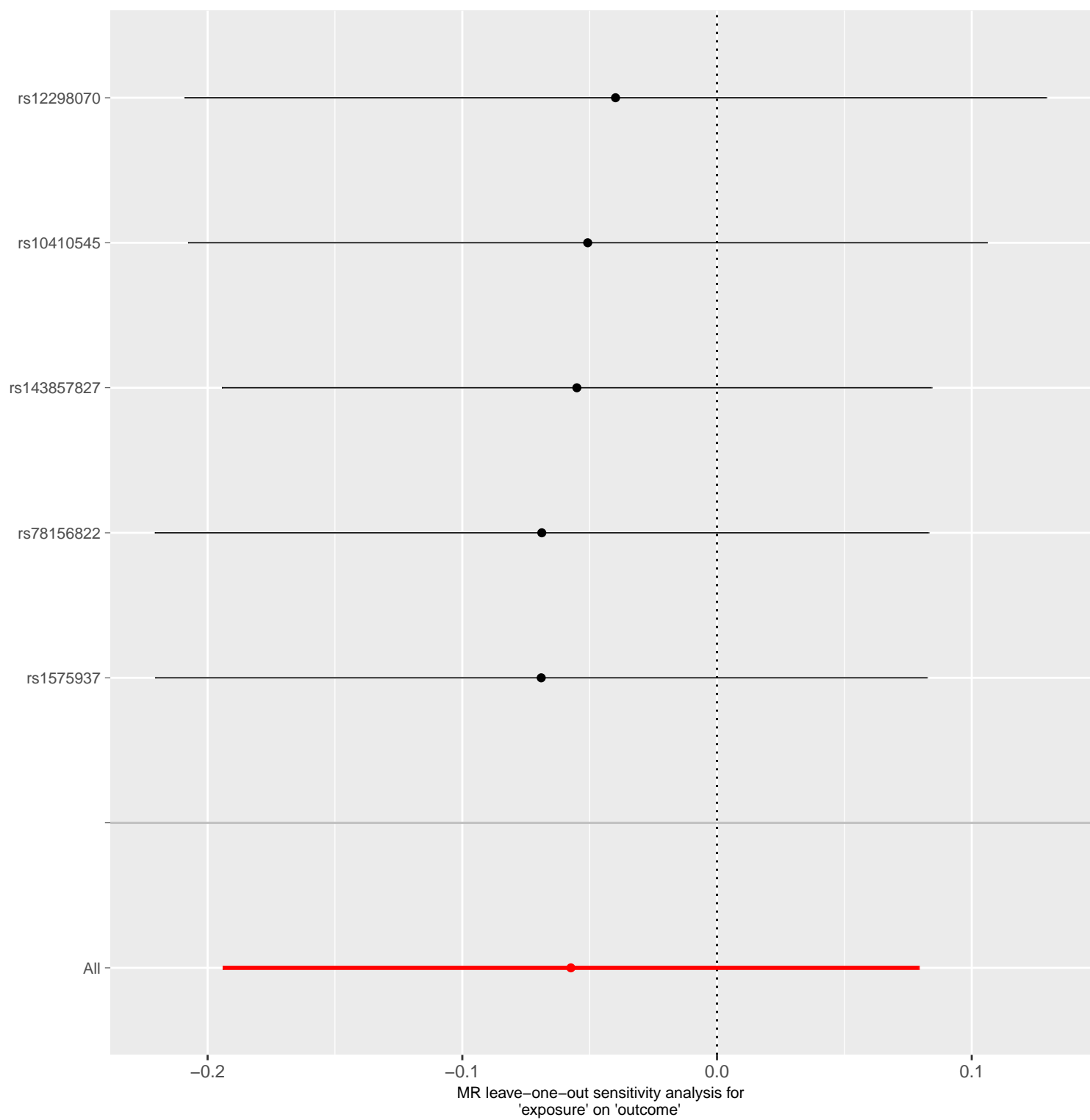

Supplement: Supplementary Data Sheet 3 — Full results of the pairwise Mendelian randomization analyses between ulcerative colitis-associated microbial taxa and ulcerative colitis-associated pyroptosis proteins, used for the downstream mediation analysis. [file DataSheet3.zip › GM_bd_fer_result/GCST90032466+13032_1_BECN1_BECN1/sensitivity-analysis.pdf]

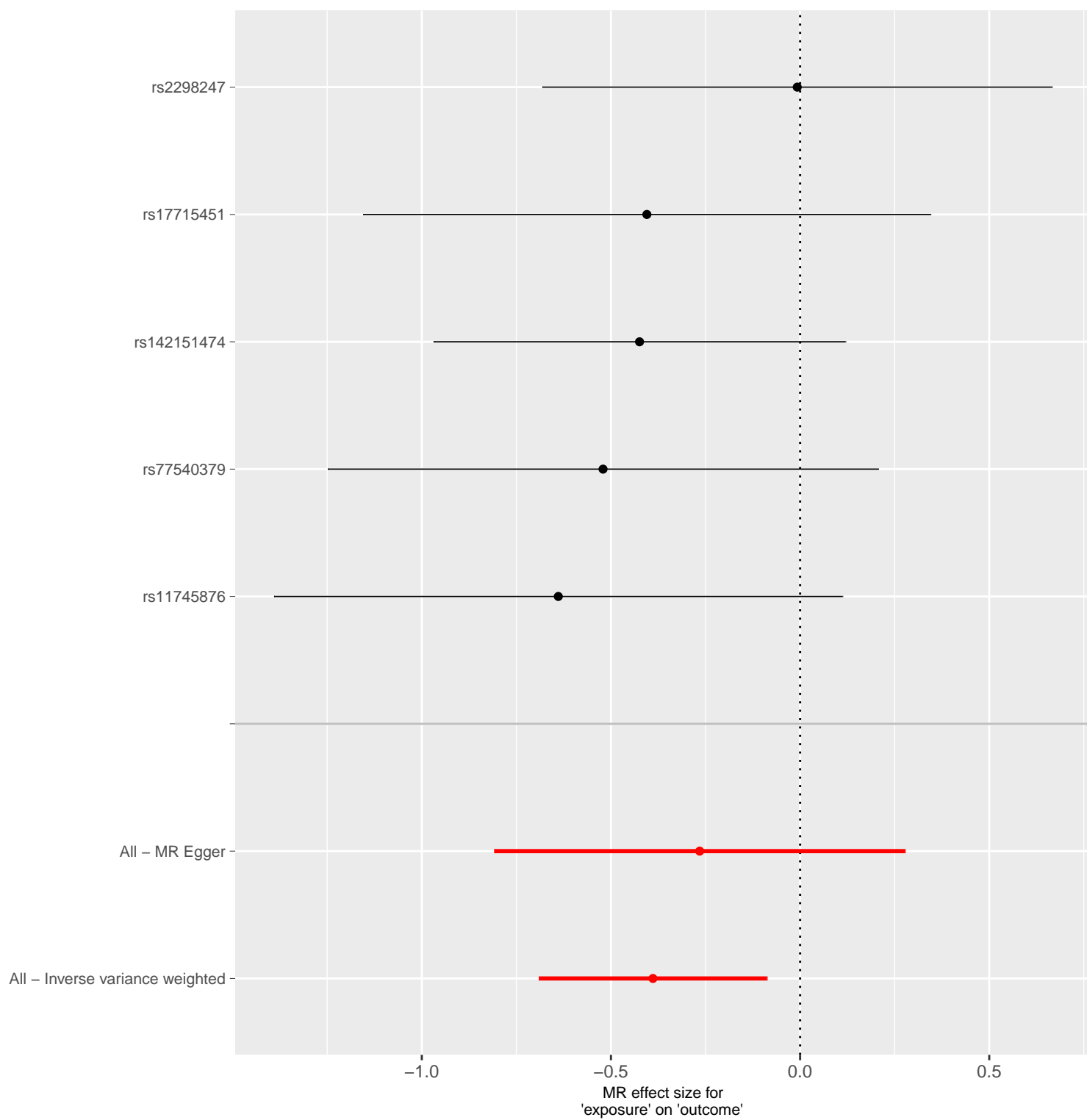

Supplement: Supplementary Data Sheet 3 — Full results of the pairwise Mendelian randomization analyses between ulcerative colitis-associated microbial taxa and ulcerative colitis-associated pyroptosis proteins, used for the downstream mediation analysis. [file DataSheet3.zip › GM_bd_fer_result/GCST90032583+11067_13_BGLAP_Osteocalcin/forest.pdf]

# MR Method

- Inverse variance weighted
- MR Egger

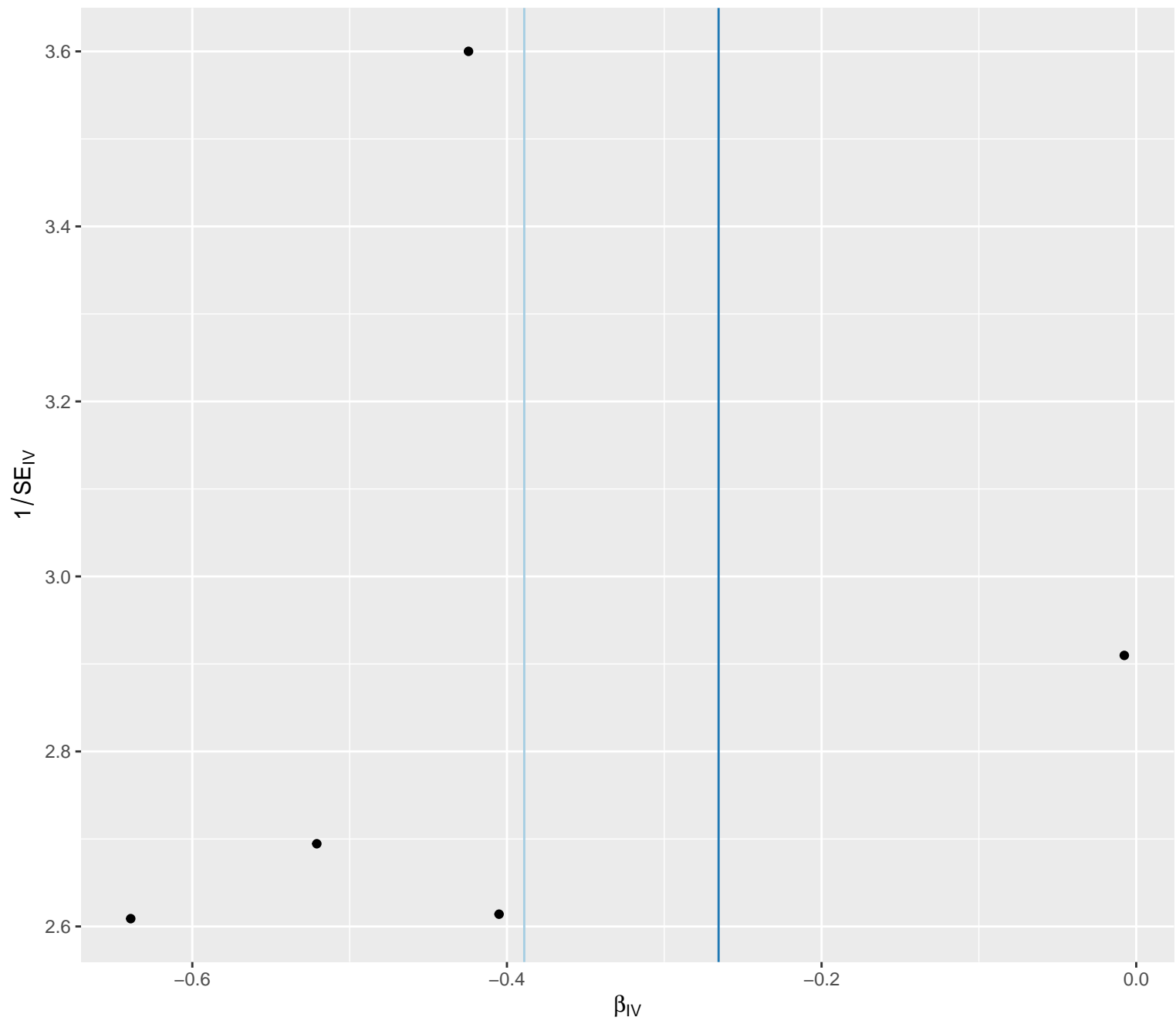

Supplement: Supplementary Data Sheet 3 — Full results of the pairwise Mendelian randomization analyses between ulcerative colitis-associated microbial taxa and ulcerative colitis-associated pyroptosis proteins, used for the downstream mediation analysis. [file DataSheet3.zip › GM_bd_fer_result/GCST90032583+11067_13_BGLAP_Osteocalcin/funnelplot.pdf]

# MR Test

- Inverse variance weighted
- MR Egger
- Simple mode
- Weighted median
- Weighted mode

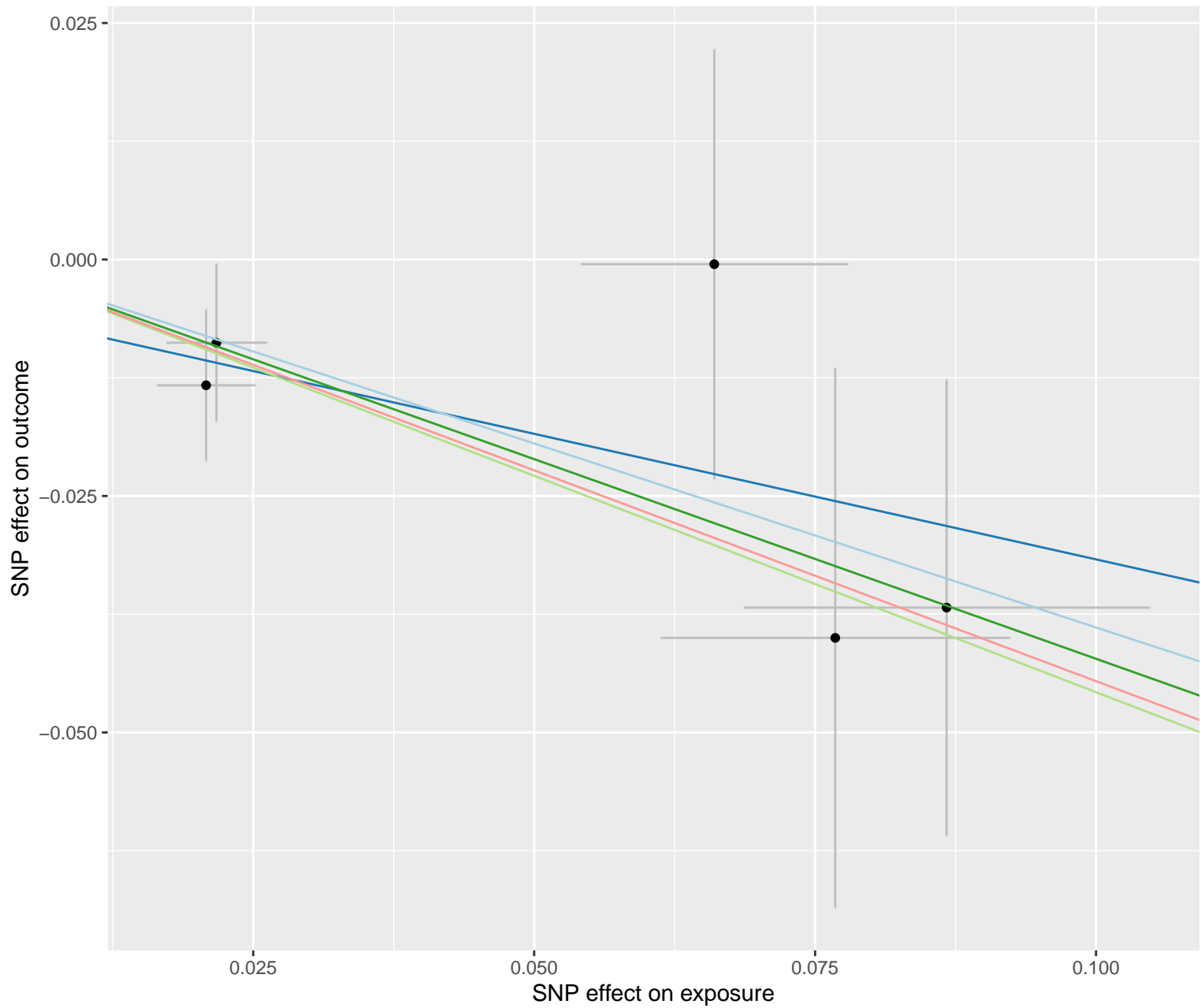

Supplement: Supplementary Data Sheet 3 — Full results of the pairwise Mendelian randomization analyses between ulcerative colitis-associated microbial taxa and ulcerative colitis-associated pyroptosis proteins, used for the downstream mediation analysis. [file DataSheet3.zip › GM_bd_fer_result/GCST90032583+11067_13_BGLAP_Osteocalcin/scatter.pdf]

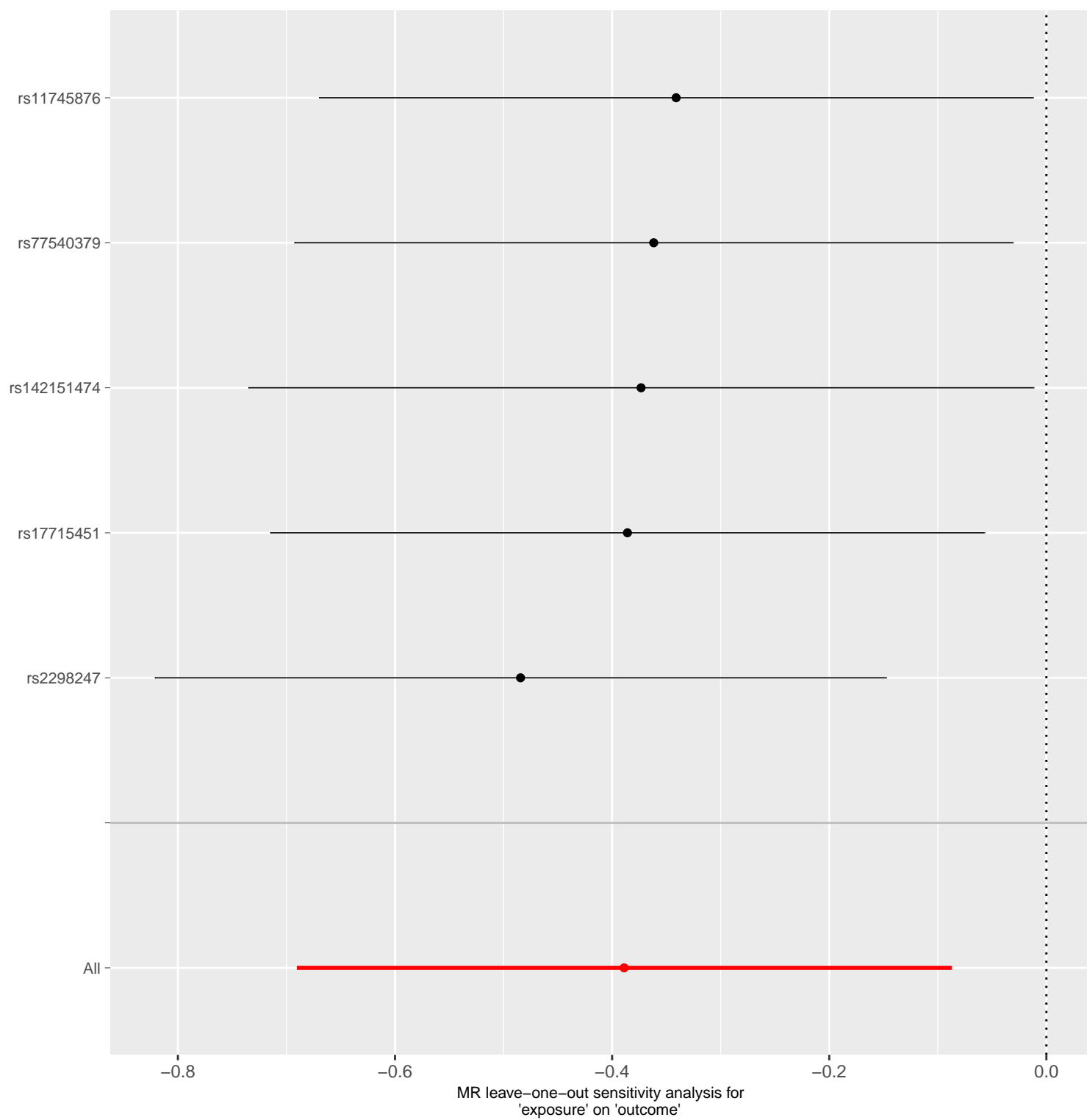

Supplement: Supplementary Data Sheet 3 — Full results of the pairwise Mendelian randomization analyses between ulcerative colitis-associated microbial taxa and ulcerative colitis-associated pyroptosis proteins, used for the downstream mediation analysis. [file DataSheet3.zip › GM_bd_fer_result/GCST90032583+11067_13_BGLAP_Osteocalcin/sensitivity-analysis.pdf]

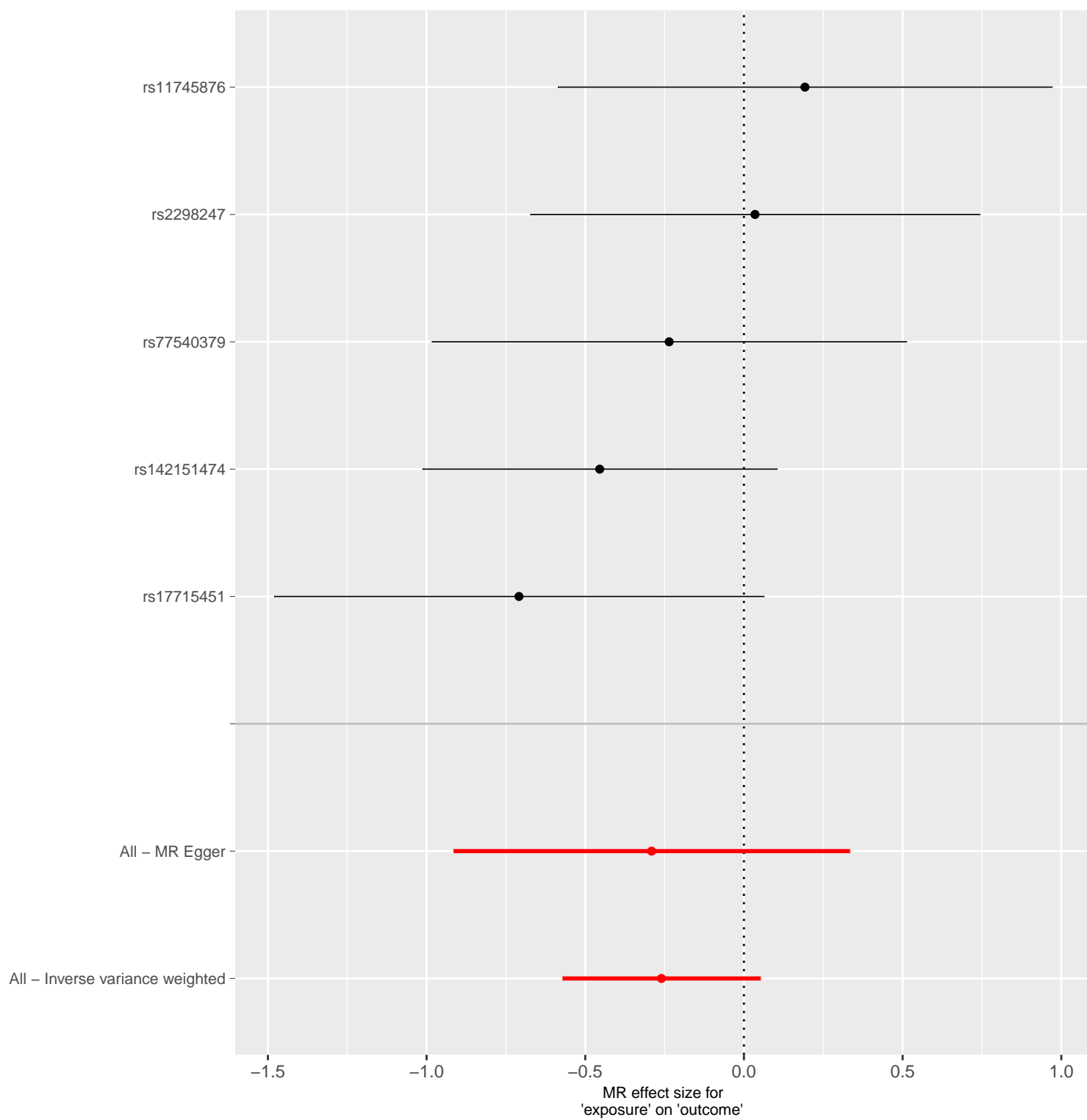

Supplement: Supplementary Data Sheet 3 — Full results of the pairwise Mendelian randomization analyses between ulcerative colitis-associated microbial taxa and ulcerative colitis-associated pyroptosis proteins, used for the downstream mediation analysis. [file DataSheet3.zip › GM_bd_fer_result/GCST90032583+12332_7_EEF2K_EF2K/forest.pdf]

# MR Method

- Inverse variance weighted
- MR Egger

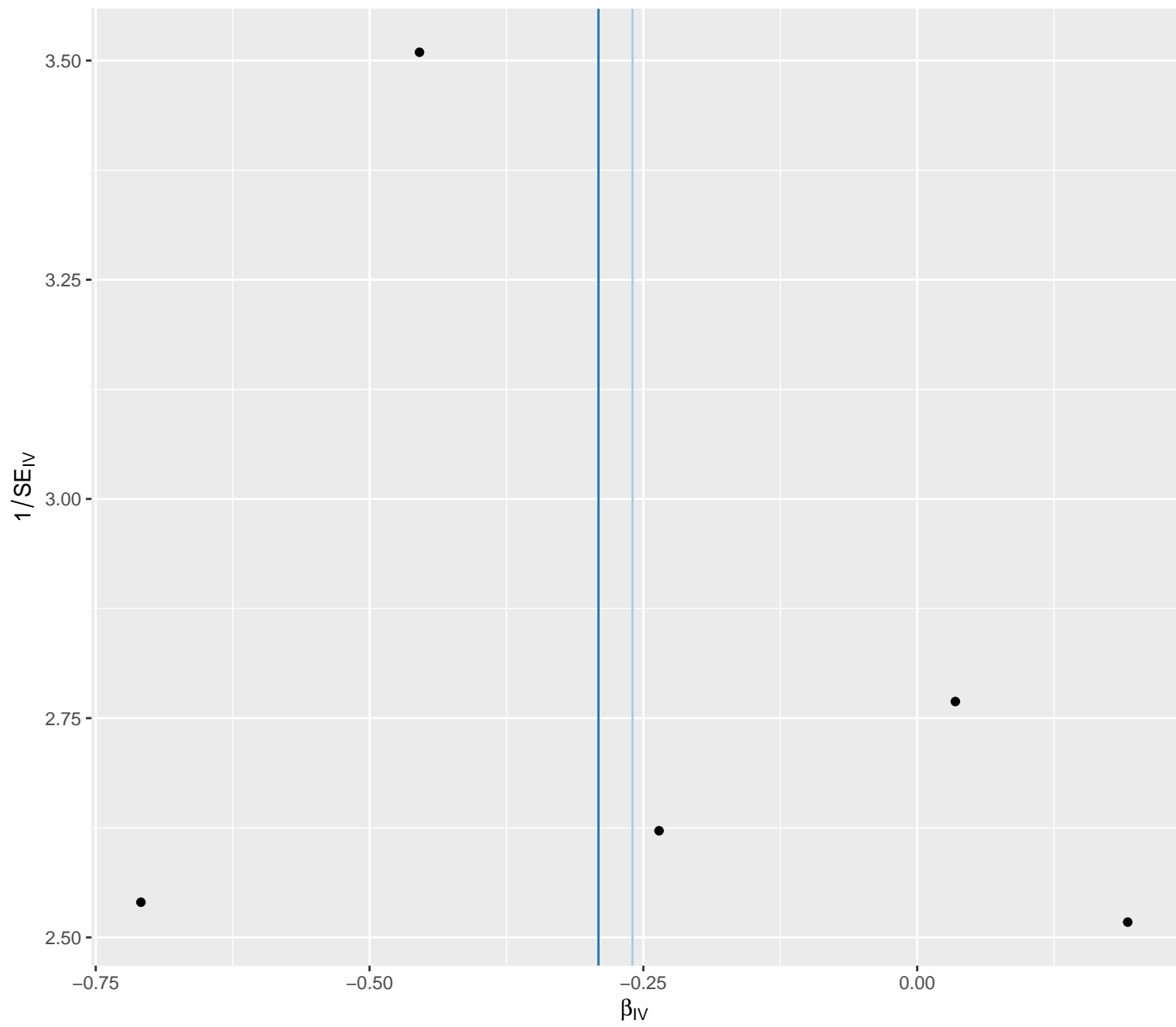

Supplement: Supplementary Data Sheet 3 — Full results of the pairwise Mendelian randomization analyses between ulcerative colitis-associated microbial taxa and ulcerative colitis-associated pyroptosis proteins, used for the downstream mediation analysis. [file DataSheet3.zip › GM_bd_fer_result/GCST90032583+12332_7_EEF2K_EF2K/funnelplot.pdf]

# MR Test

- Inverse variance weighted
- MR Egger
- Simple mode
- Weighted median
- Weighted mode

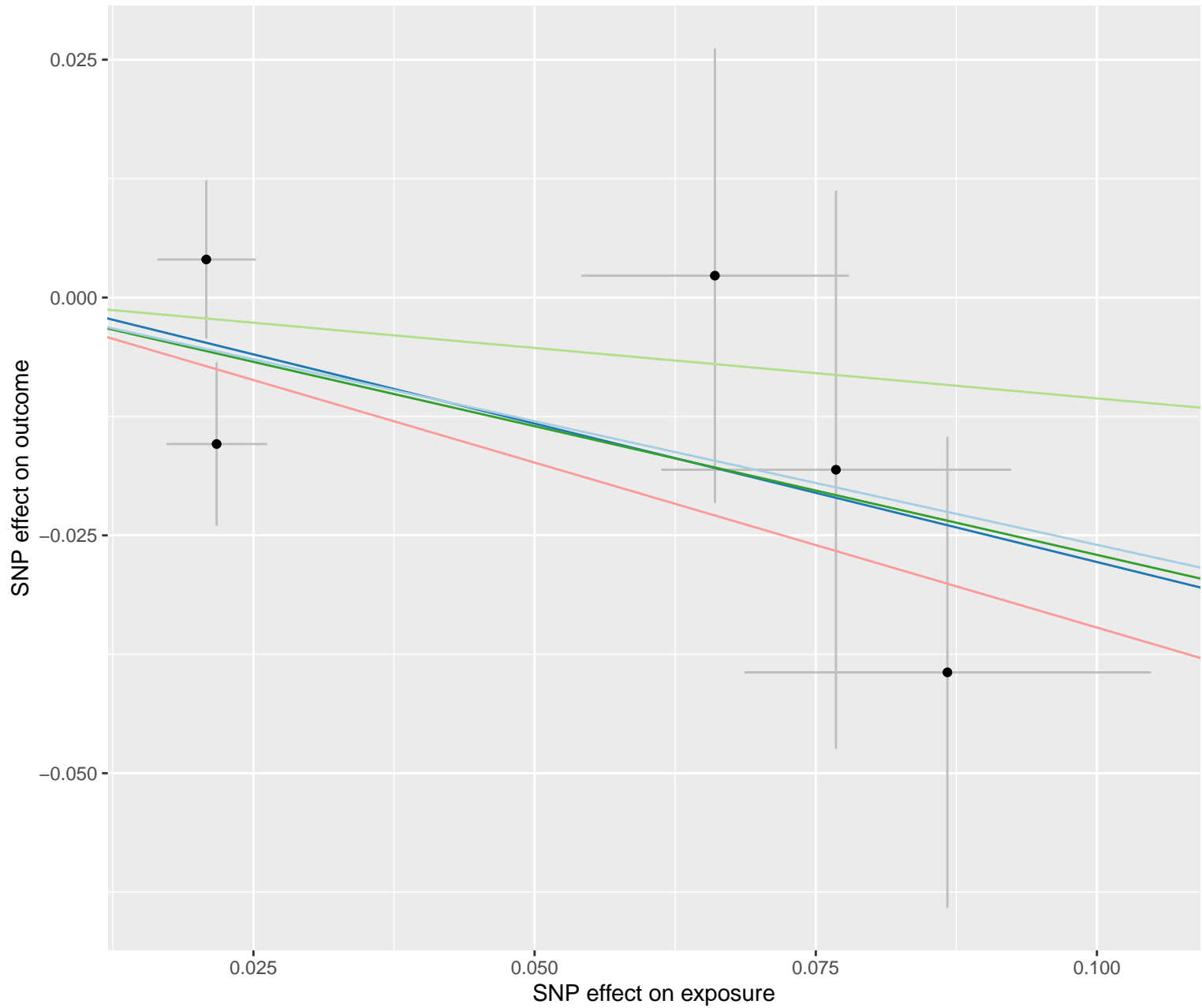

Supplement: Supplementary Data Sheet 3 — Full results of the pairwise Mendelian randomization analyses between ulcerative colitis-associated microbial taxa and ulcerative colitis-associated pyroptosis proteins, used for the downstream mediation analysis. [file DataSheet3.zip › GM_bd_fer_result/GCST90032583+12332_7_EEF2K_EF2K/scatter.pdf]

rs142151474

rs17715451

rs77540379

rs2298247

rs11745876

All

-0.50

-0.25

0.00

0.25

MR leave-one-out sensitivity analysis for  
'exposure' on 'outcome'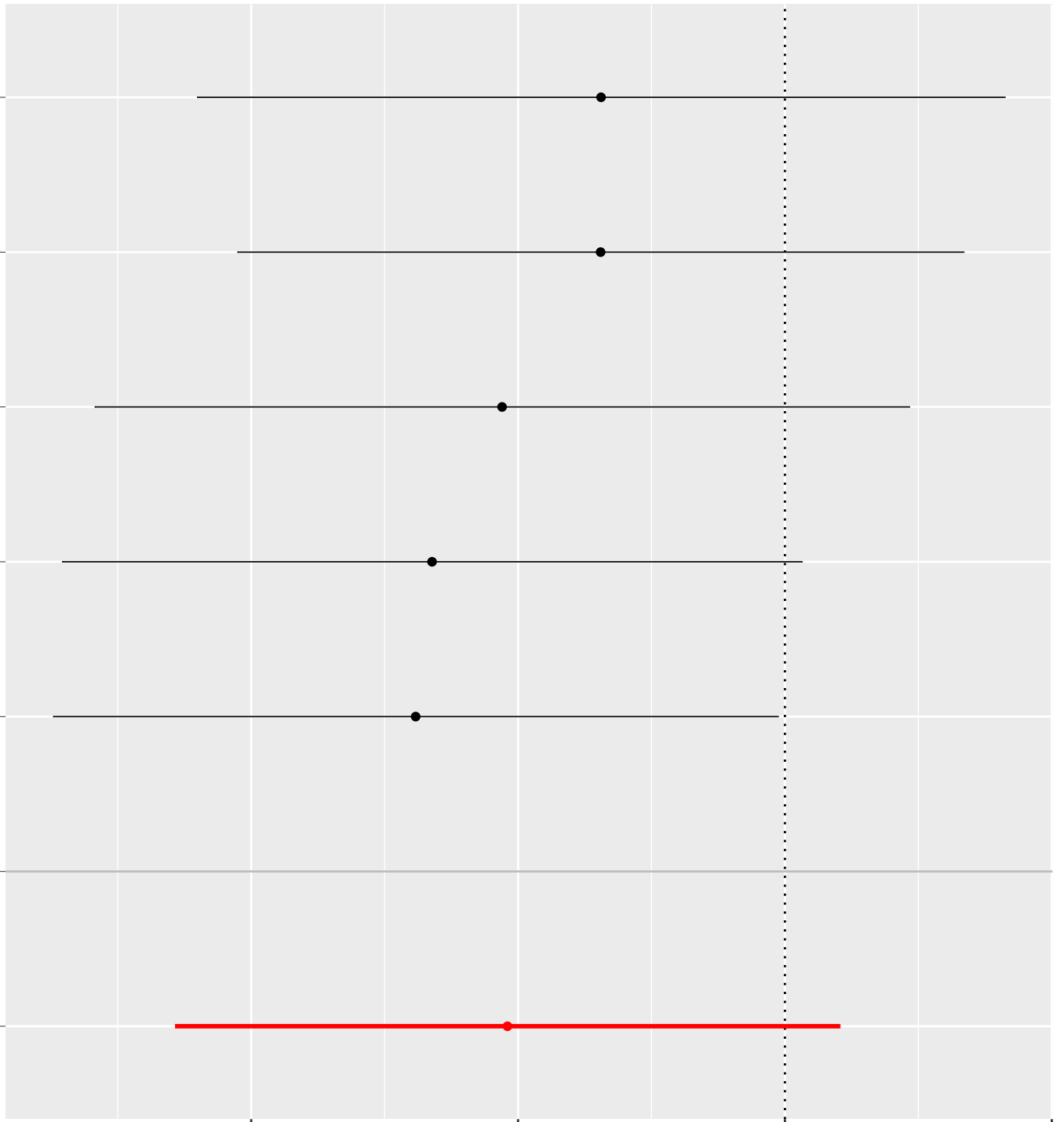

Supplement: Supplementary Data Sheet 3 — Full results of the pairwise Mendelian randomization analyses between ulcerative colitis-associated microbial taxa and ulcerative colitis-associated pyroptosis proteins, used for the downstream mediation analysis. [file DataSheet3.zip › GM_bd_fer_result/GCST90032583+12332_7_EEF2K_EF2K/sensitivity-analysis.pdf]

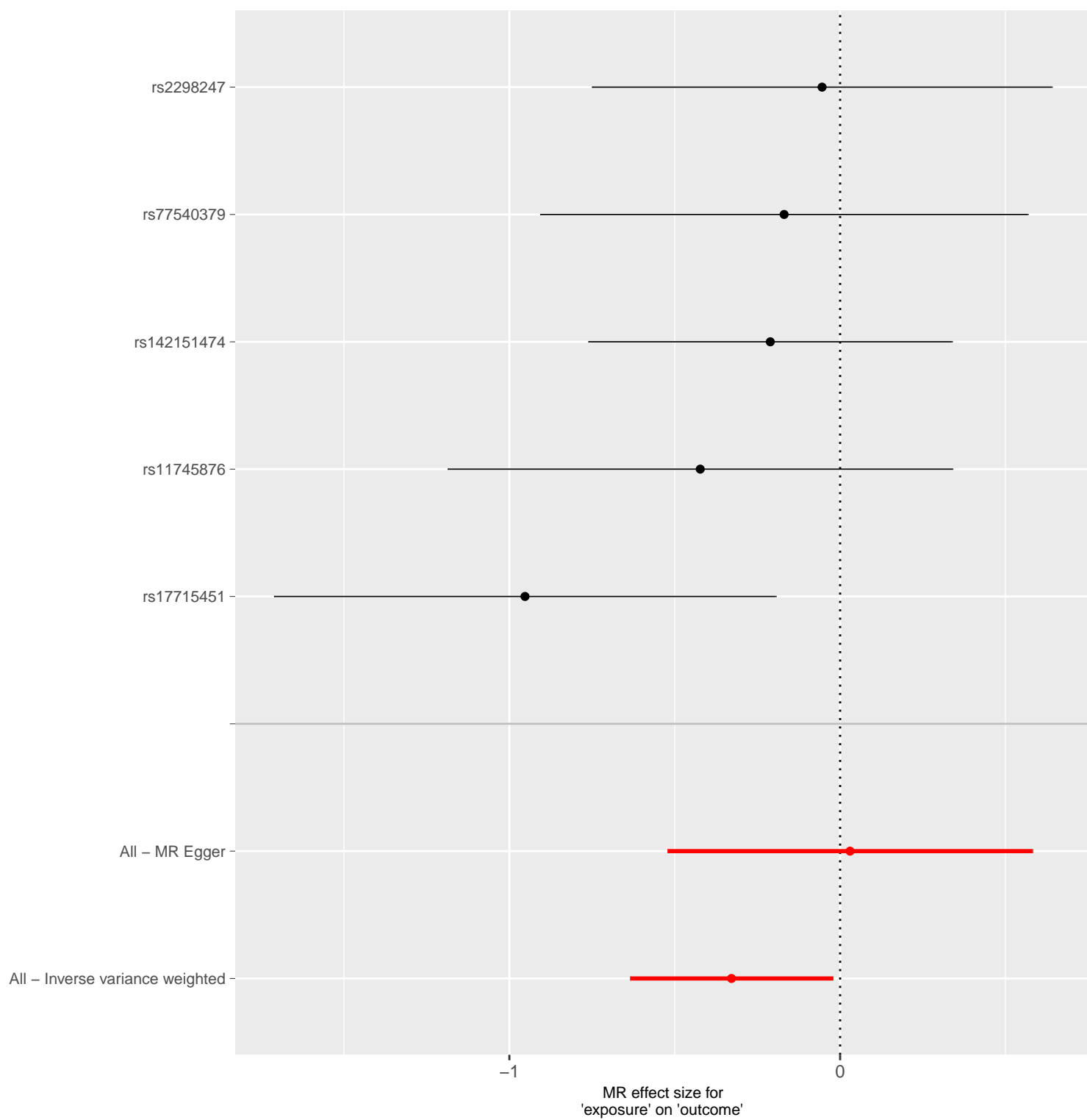

Supplement: Supplementary Data Sheet 3 — Full results of the pairwise Mendelian randomization analyses between ulcerative colitis-associated microbial taxa and ulcerative colitis-associated pyroptosis proteins, used for the downstream mediation analysis. [file DataSheet3.zip › GM_bd_fer_result/GCST90032583+12439_67_IRF9_ISGF3/forest.pdf]

# MR Method

- Inverse variance weighted
- MR Egger

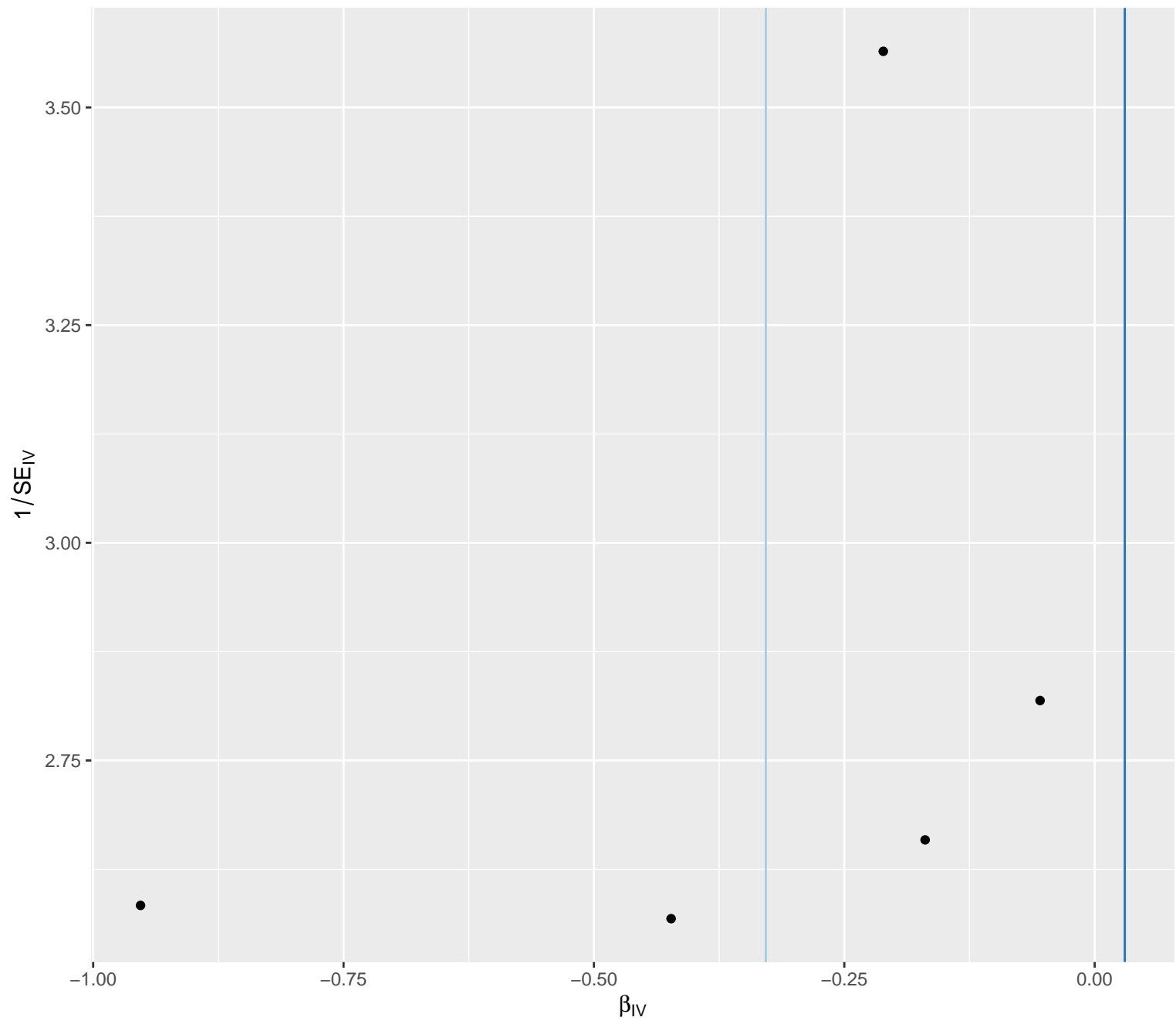

Supplement: Supplementary Data Sheet 3 — Full results of the pairwise Mendelian randomization analyses between ulcerative colitis-associated microbial taxa and ulcerative colitis-associated pyroptosis proteins, used for the downstream mediation analysis. [file DataSheet3.zip › GM_bd_fer_result/GCST90032583+12439_67_IRF9_ISGF3/funnelplot.pdf]

# MR Test

- Inverse variance weighted
- MR Egger
- Simple mode
- Weighted median
- Weighted mode

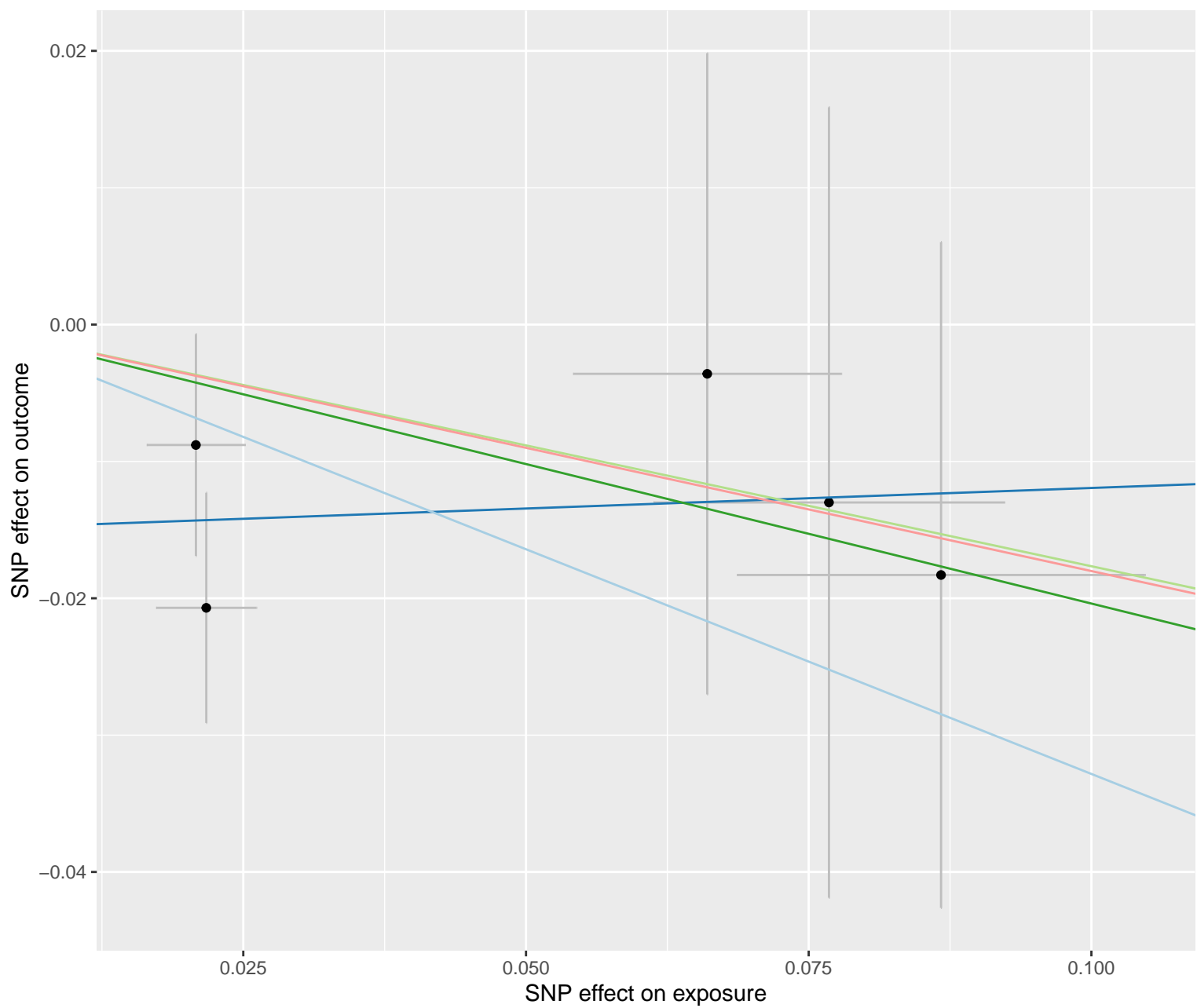

Supplement: Supplementary Data Sheet 3 — Full results of the pairwise Mendelian randomization analyses between ulcerative colitis-associated microbial taxa and ulcerative colitis-associated pyroptosis proteins, used for the downstream mediation analysis. [file DataSheet3.zip › GM_bd_fer_result/GCST90032583+12439_67_IRF9_ISGF3/scatter.pdf]

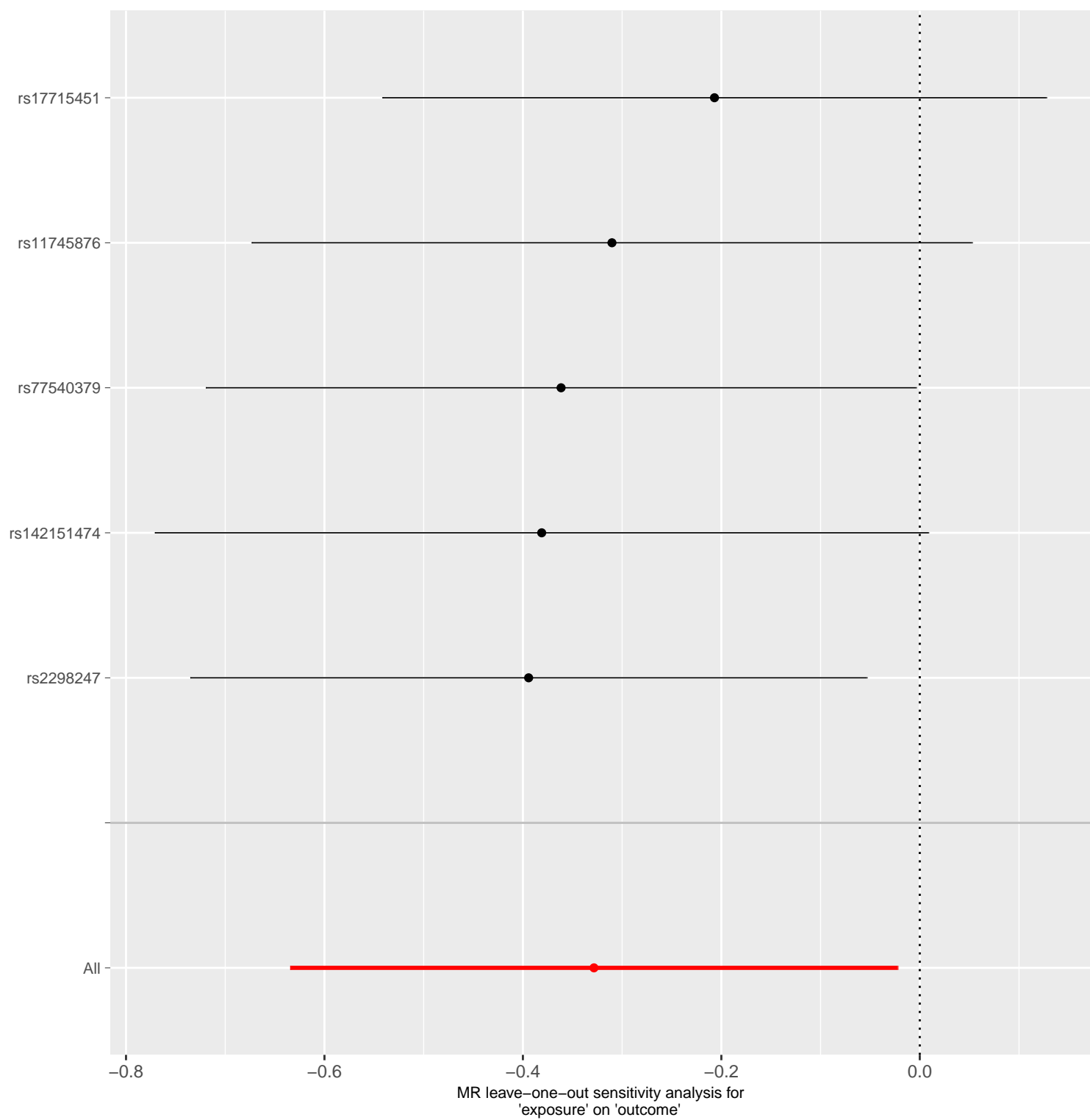

Supplement: Supplementary Data Sheet 3 — Full results of the pairwise Mendelian randomization analyses between ulcerative colitis-associated microbial taxa and ulcerative colitis-associated pyroptosis proteins, used for the downstream mediation analysis. [file DataSheet3.zip › GM_bd_fer_result/GCST90032583+12439_67_IRF9_ISGF3/sensitivity-analysis.pdf]

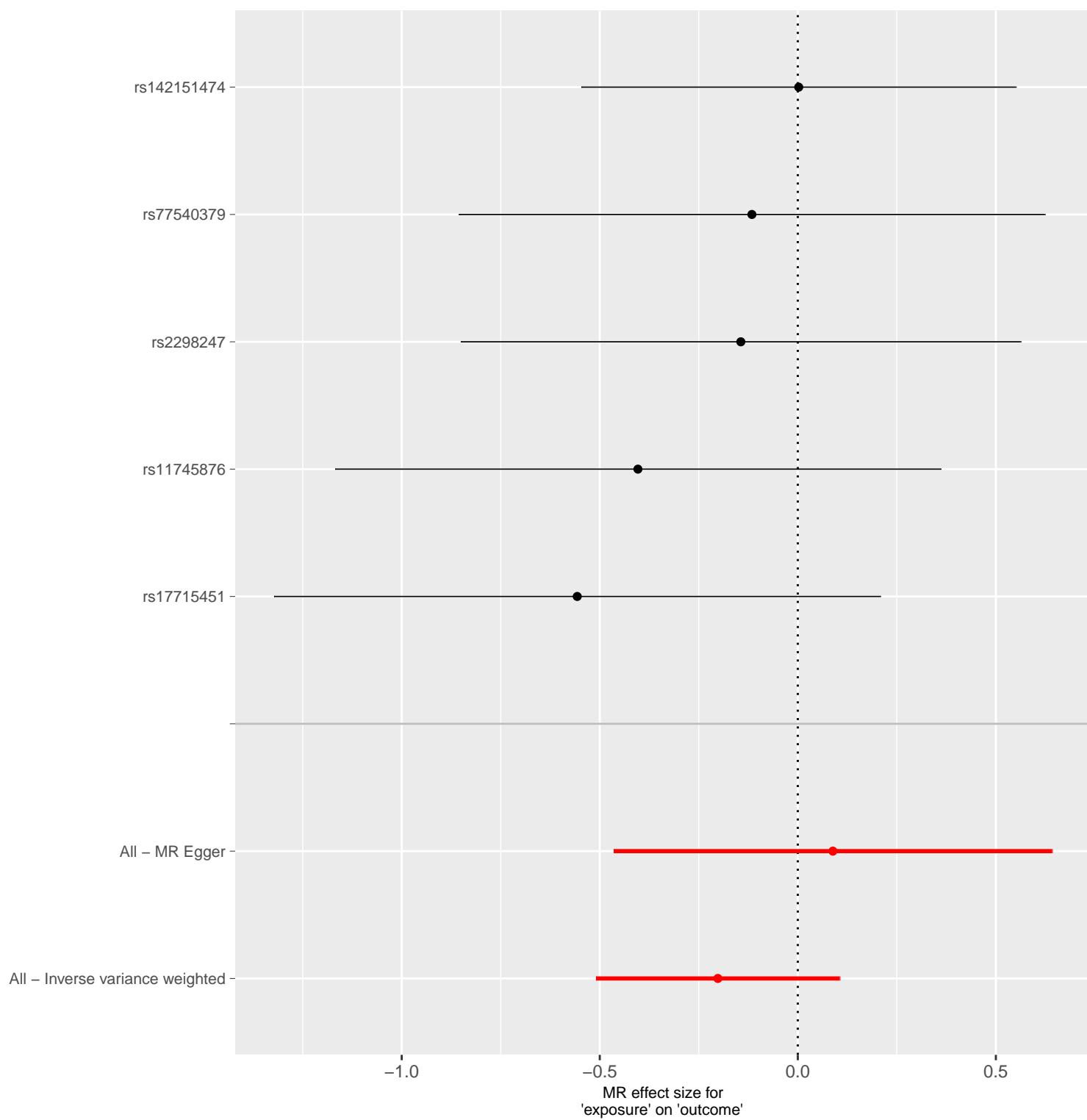

Supplement: Supplementary Data Sheet 3 — Full results of the pairwise Mendelian randomization analyses between ulcerative colitis-associated microbial taxa and ulcerative colitis-associated pyroptosis proteins, used for the downstream mediation analysis. [file DataSheet3.zip › GM_bd_fer_result/GCST90032583+13032_1_BECN1_BECN1/forest.pdf]

# MR Method

- Inverse variance weighted
- MR Egger

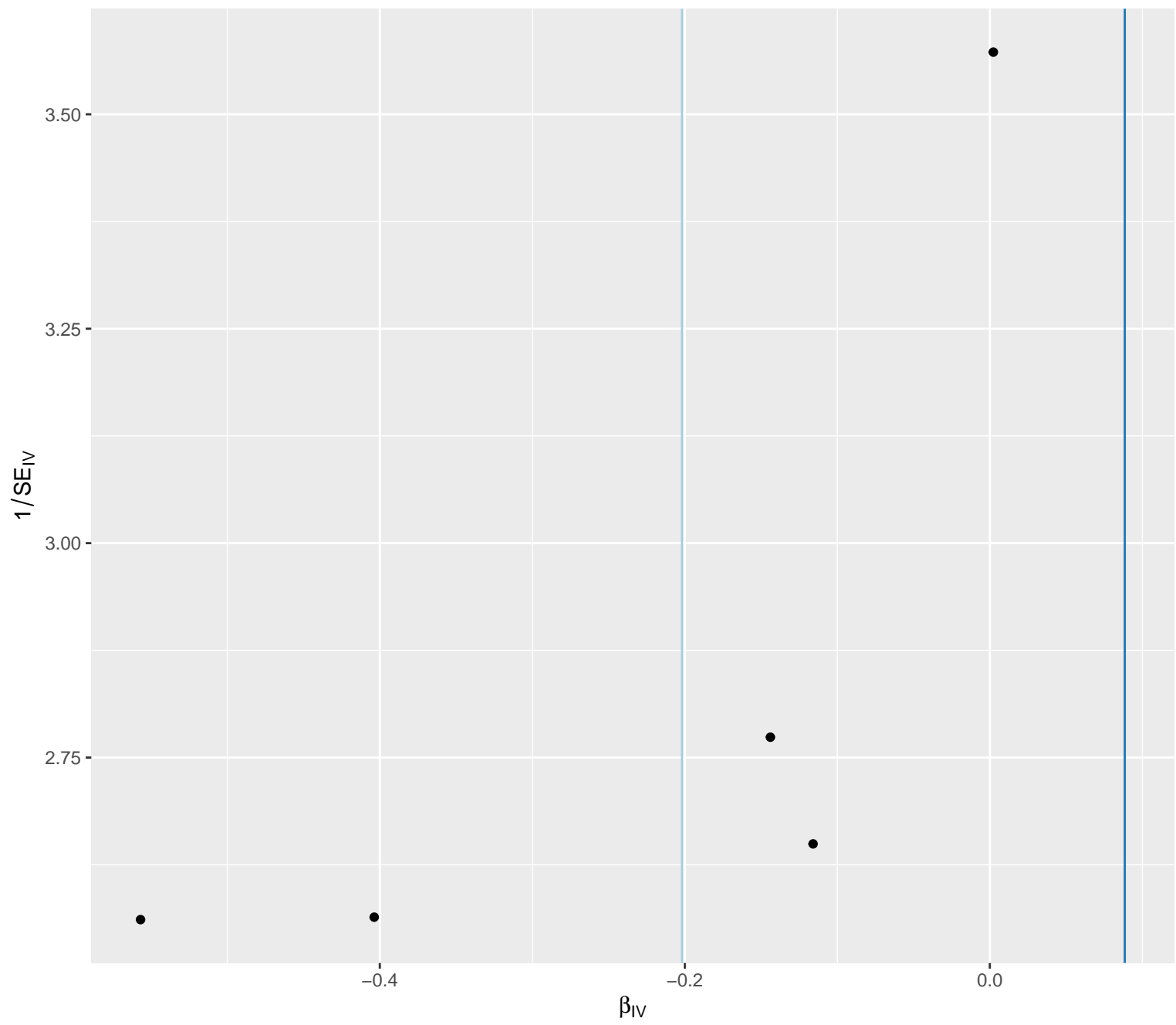

Supplement: Supplementary Data Sheet 3 — Full results of the pairwise Mendelian randomization analyses between ulcerative colitis-associated microbial taxa and ulcerative colitis-associated pyroptosis proteins, used for the downstream mediation analysis. [file DataSheet3.zip › GM_bd_fer_result/GCST90032583+13032_1_BECN1_BECN1/funnelplot.pdf]

# MR Test

- Inverse variance weighted
- MR Egger
- Simple mode
- Weighted median
- Weighted mode

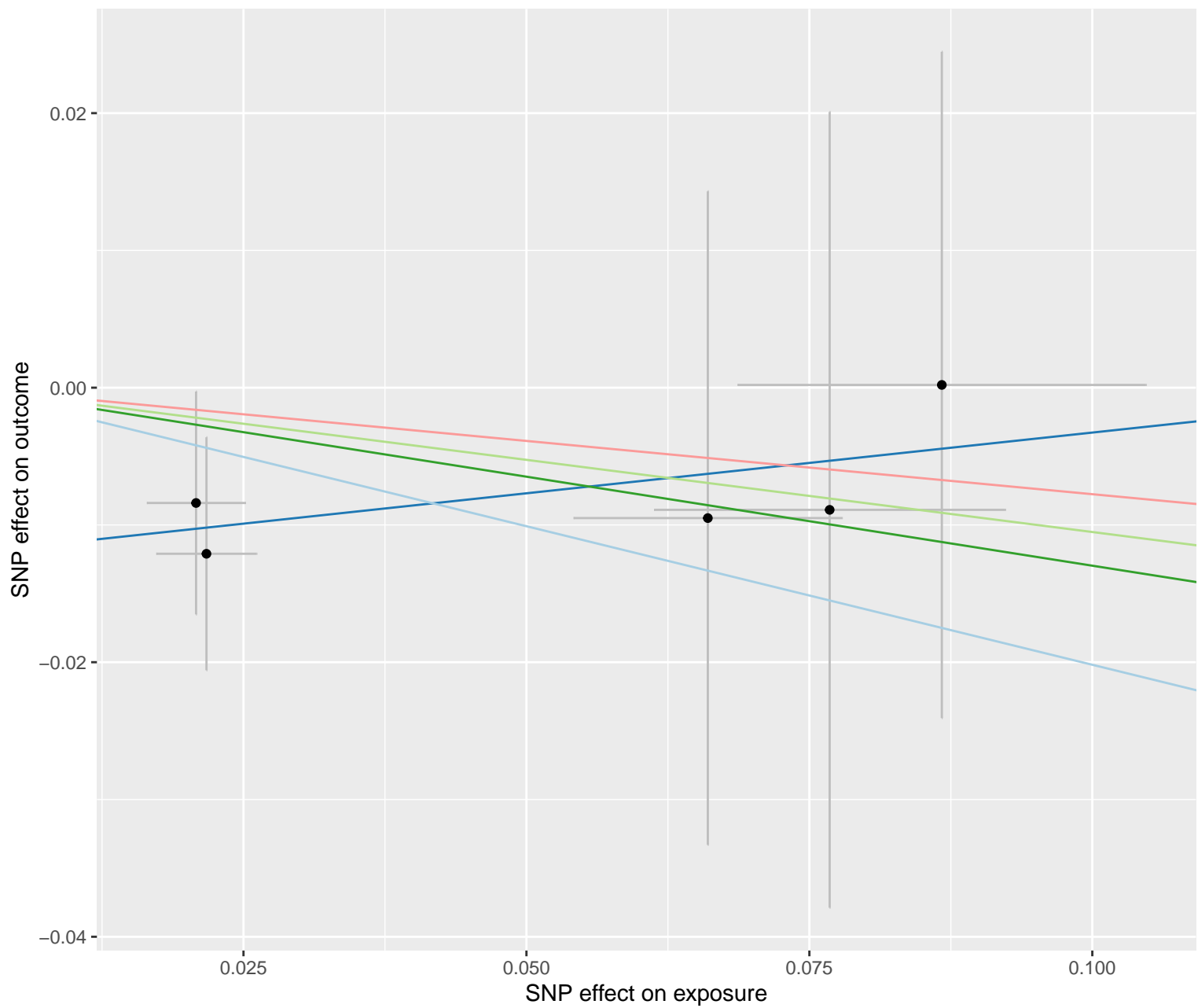

Supplement: Supplementary Data Sheet 3 — Full results of the pairwise Mendelian randomization analyses between ulcerative colitis-associated microbial taxa and ulcerative colitis-associated pyroptosis proteins, used for the downstream mediation analysis. [file DataSheet3.zip › GM_bd_fer_result/GCST90032583+13032_1_BECN1_BECN1/scatter.pdf]

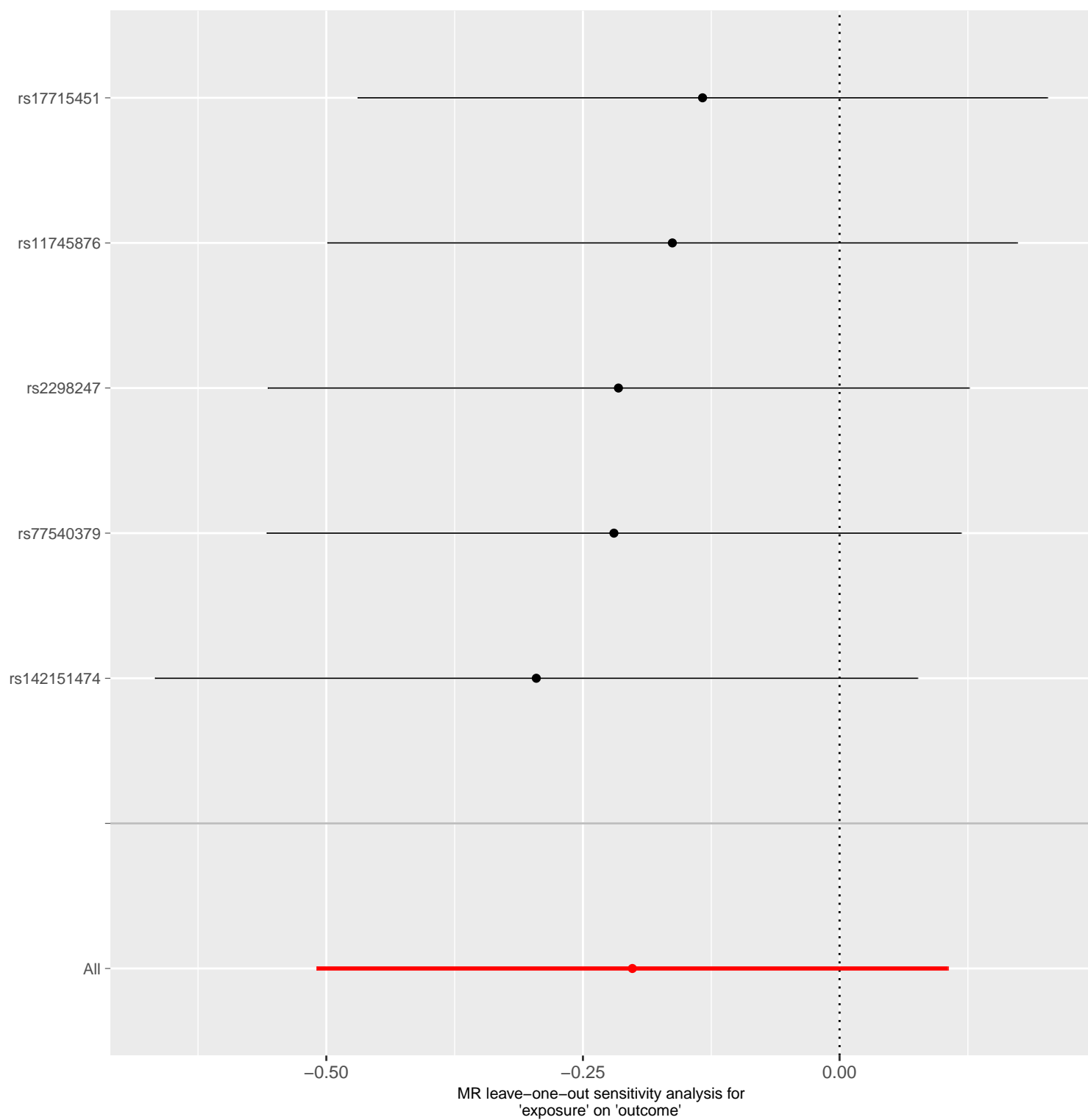

Supplement: Supplementary Data Sheet 3 — Full results of the pairwise Mendelian randomization analyses between ulcerative colitis-associated microbial taxa and ulcerative colitis-associated pyroptosis proteins, used for the downstream mediation analysis. [file DataSheet3.zip › GM_bd_fer_result/GCST90032583+13032_1_BECN1_BECN1/sensitivity-analysis.pdf]

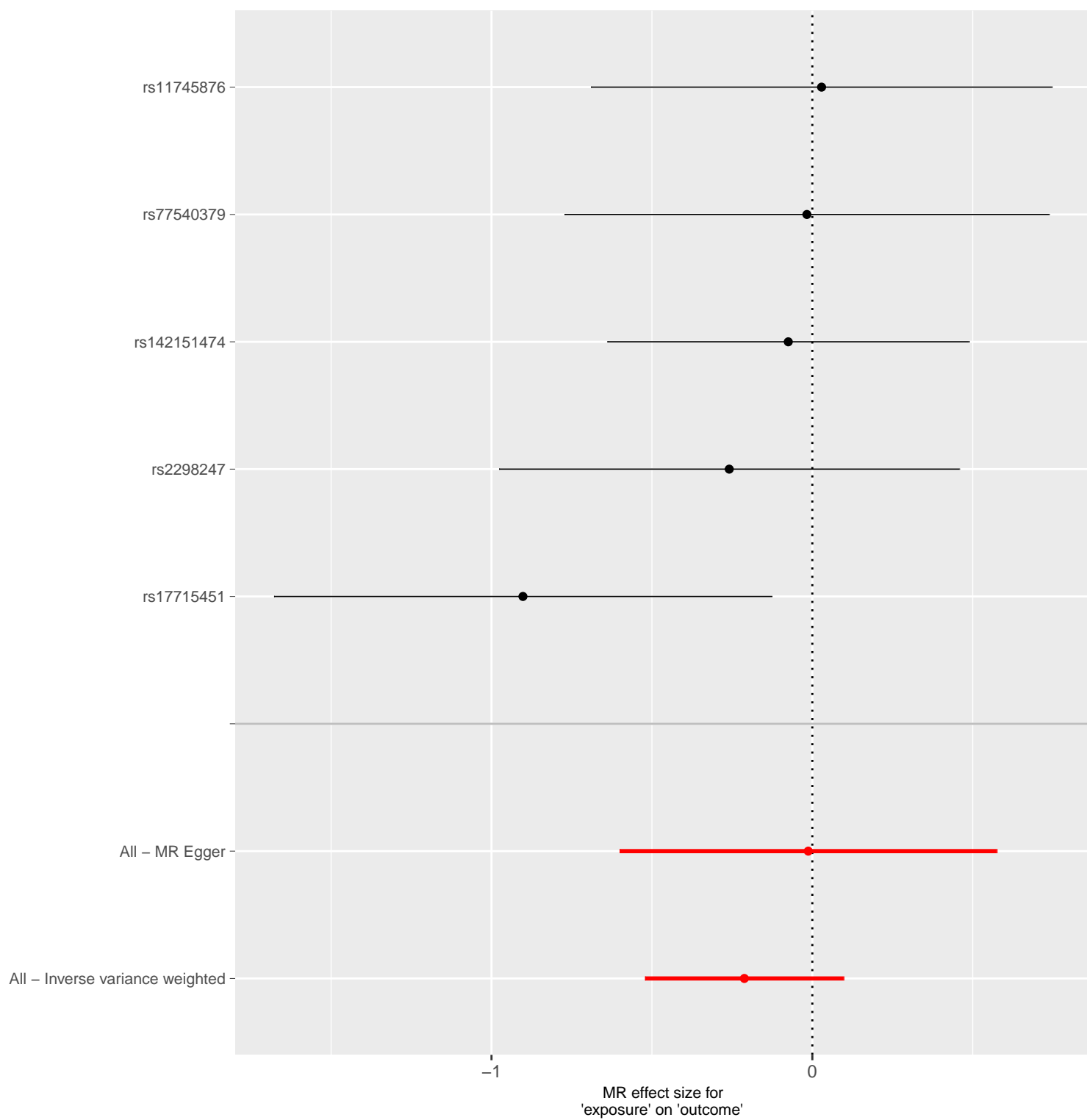

Supplement: Supplementary Data Sheet 3 — Full results of the pairwise Mendelian randomization analyses between ulcerative colitis-associated microbial taxa and ulcerative colitis-associated pyroptosis proteins, used for the downstream mediation analysis. [file DataSheet3.zip › GM_bd_fer_result/GCST90032583+13105_7_SNAP25_SNP25/forest.pdf]

# MR Method

- Inverse variance weighted
- MR Egger

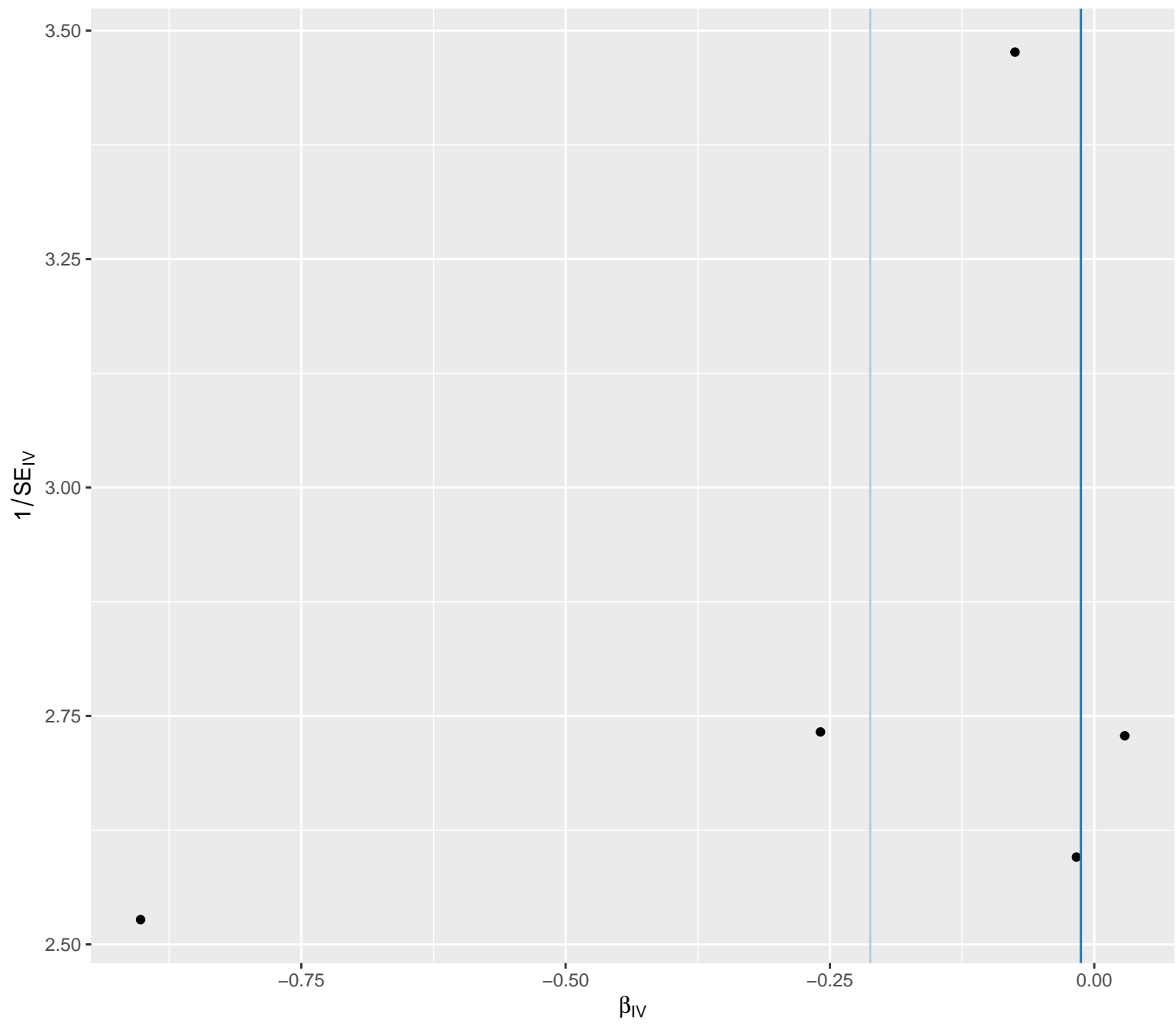

Supplement: Supplementary Data Sheet 3 — Full results of the pairwise Mendelian randomization analyses between ulcerative colitis-associated microbial taxa and ulcerative colitis-associated pyroptosis proteins, used for the downstream mediation analysis. [file DataSheet3.zip › GM_bd_fer_result/GCST90032583+13105_7_SNAP25_SNP25/funnelplot.pdf]

# MR Test

- Inverse variance weighted
- MR Egger
- Simple mode
- Weighted median
- Weighted mode

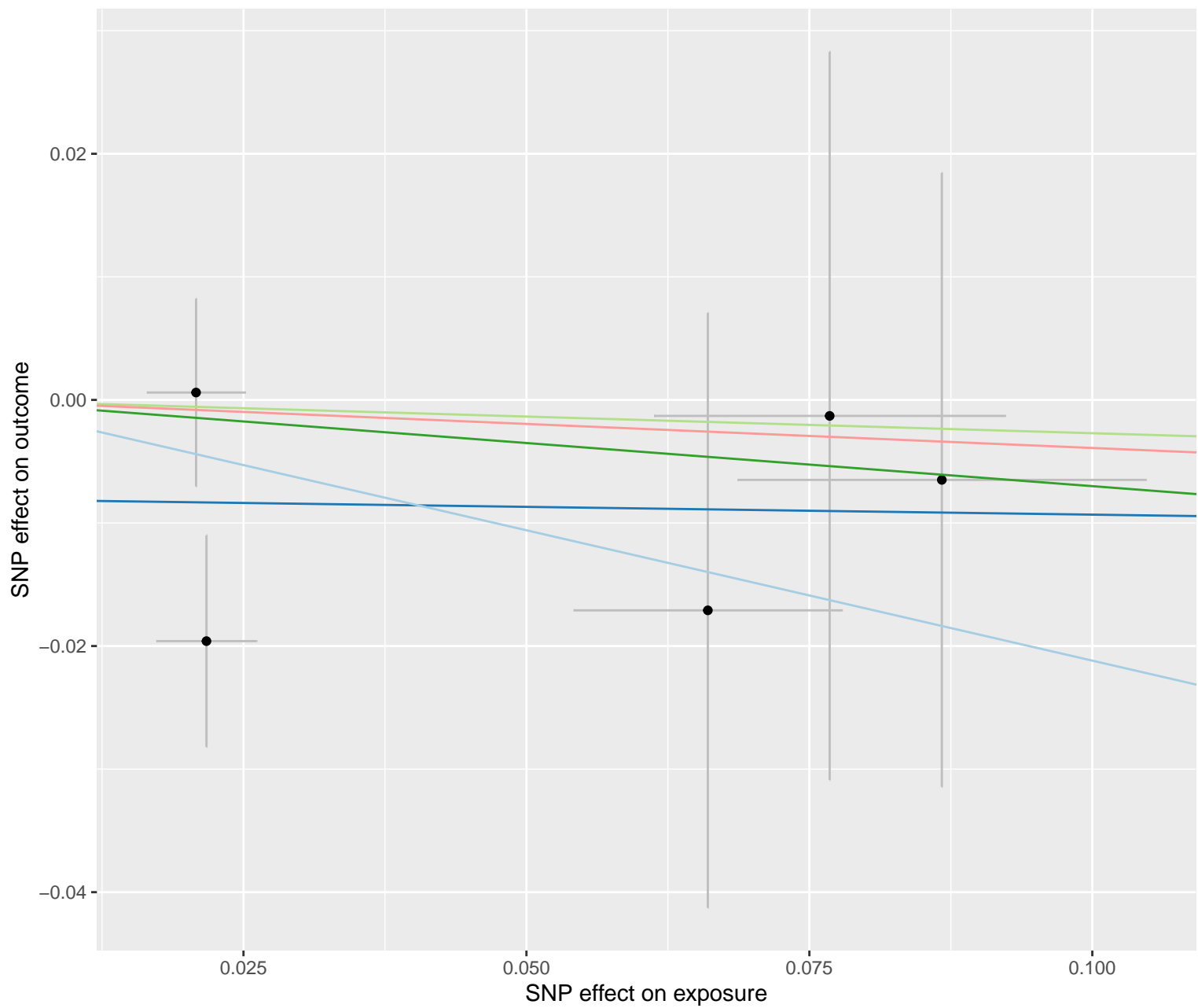

Supplement: Supplementary Data Sheet 3 — Full results of the pairwise Mendelian randomization analyses between ulcerative colitis-associated microbial taxa and ulcerative colitis-associated pyroptosis proteins, used for the downstream mediation analysis. [file DataSheet3.zip › GM_bd_fer_result/GCST90032583+13105_7_SNAP25_SNP25/scatter.pdf]

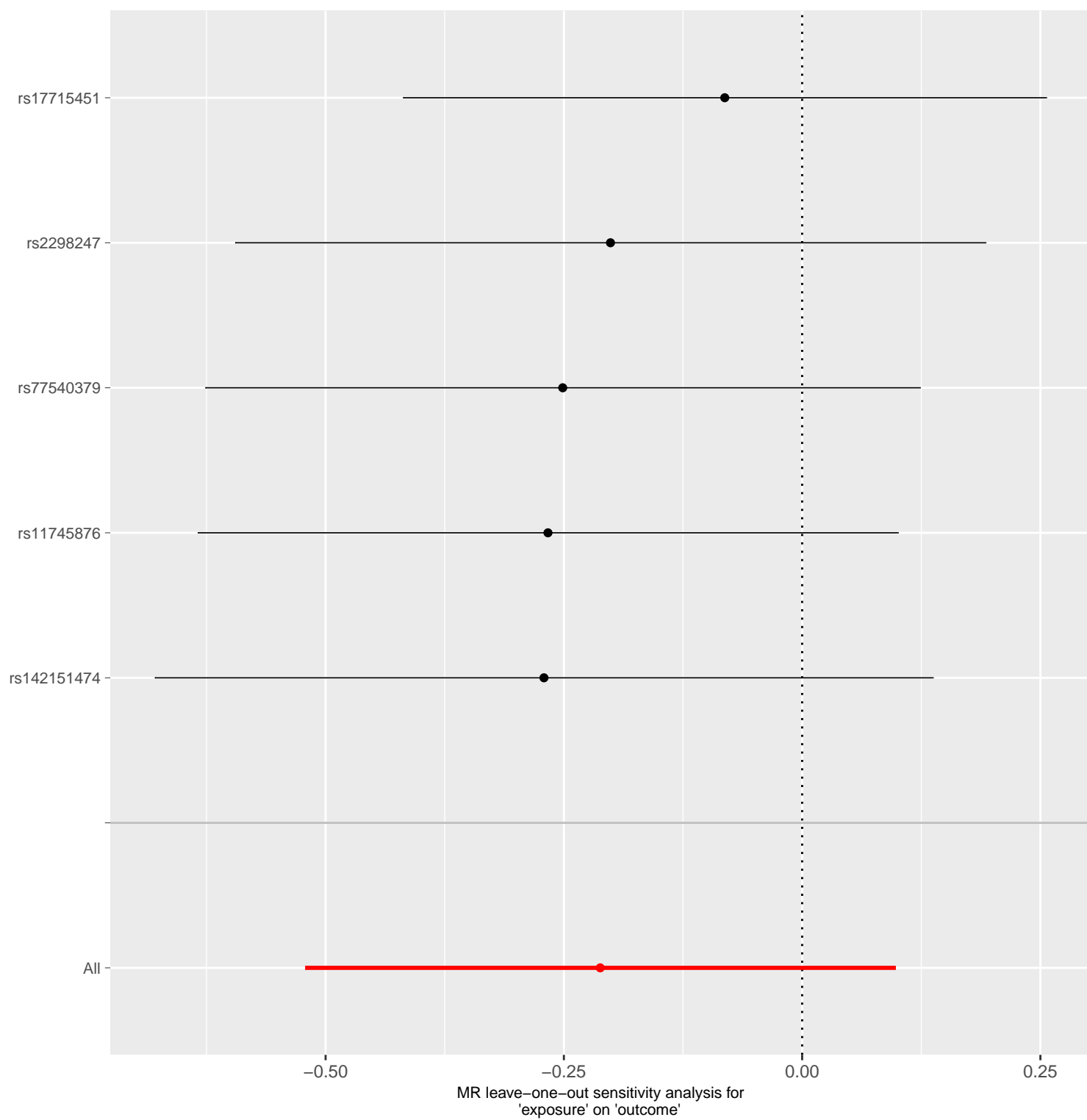

Supplement: Supplementary Data Sheet 3 — Full results of the pairwise Mendelian randomization analyses between ulcerative colitis-associated microbial taxa and ulcerative colitis-associated pyroptosis proteins, used for the downstream mediation analysis. [file DataSheet3.zip › GM_bd_fer_result/GCST90032583+13105_7_SNAP25_SNP25/sensitivity-analysis.pdf]

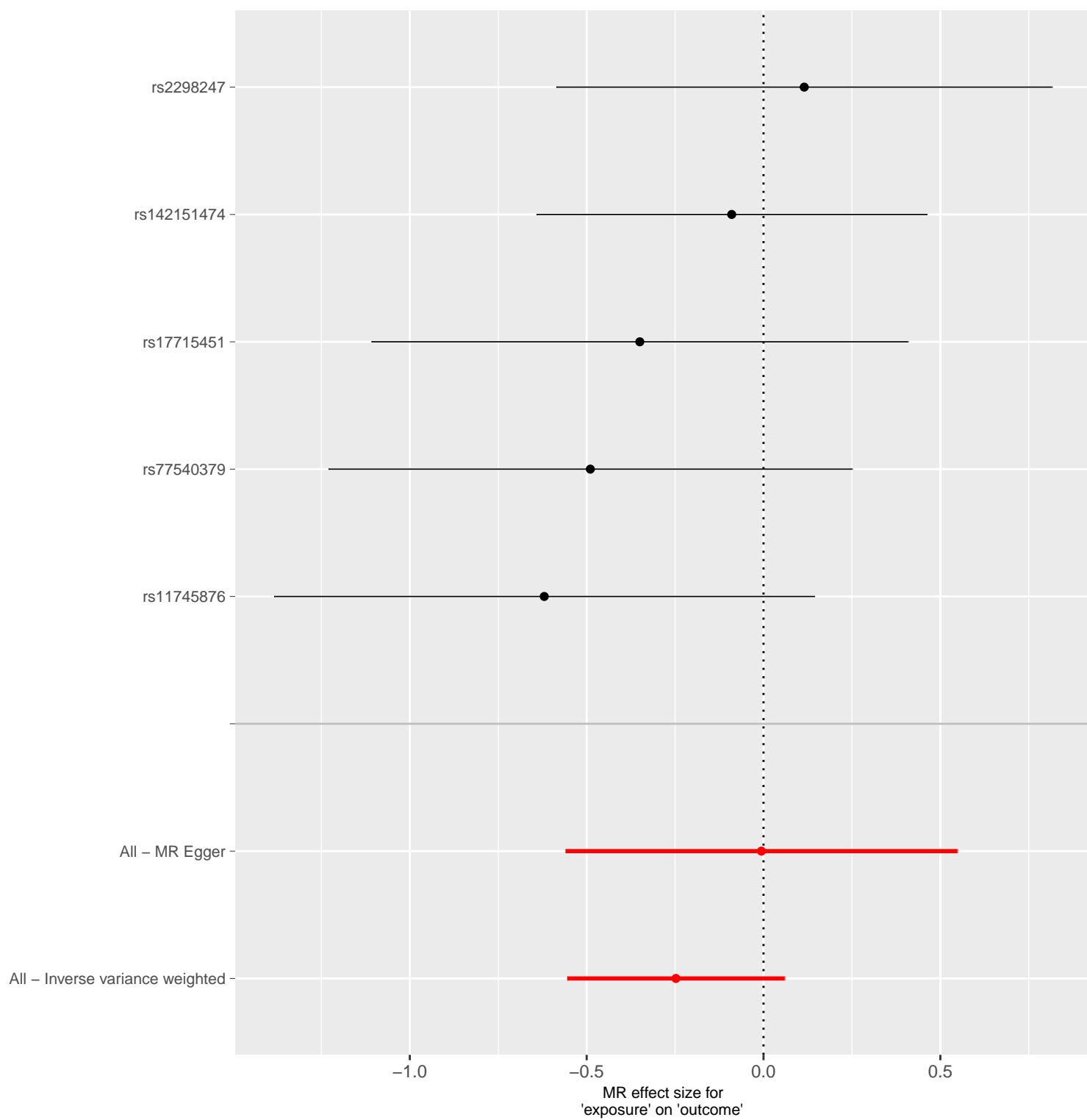

Supplement: Supplementary Data Sheet 3 — Full results of the pairwise Mendelian randomization analyses between ulcerative colitis-associated microbial taxa and ulcerative colitis-associated pyroptosis proteins, used for the downstream mediation analysis. [file DataSheet3.zip › GM_bd_fer_result/GCST90032583+13985_12_SMURF2_SMUF2/forest.pdf]

# MR Method

- Inverse variance weighted
- MR Egger

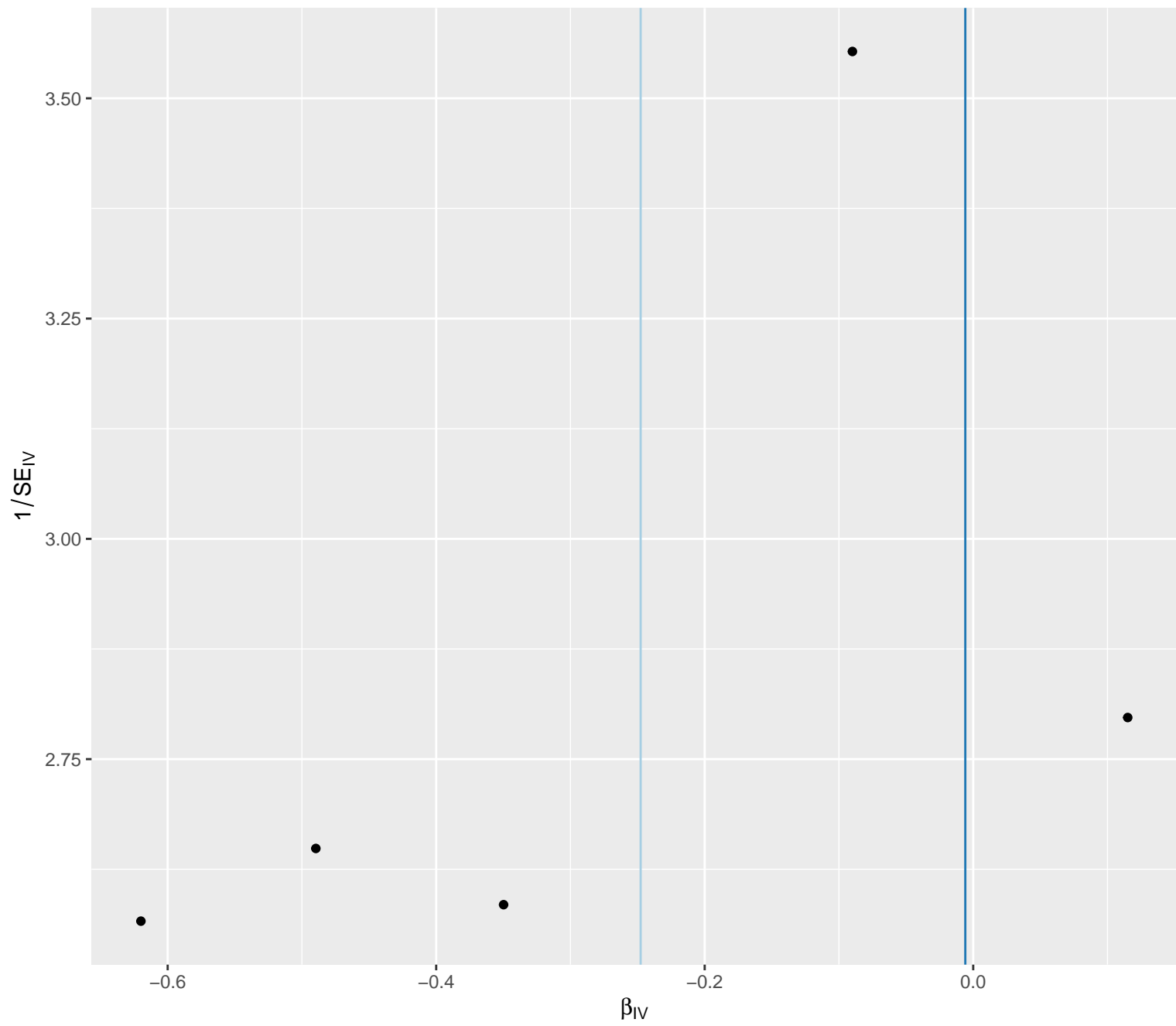

Supplement: Supplementary Data Sheet 3 — Full results of the pairwise Mendelian randomization analyses between ulcerative colitis-associated microbial taxa and ulcerative colitis-associated pyroptosis proteins, used for the downstream mediation analysis. [file DataSheet3.zip › GM_bd_fer_result/GCST90032583+13985_12_SMURF2_SMUF2/funnelplot.pdf]

# MR Test

- Inverse variance weighted
- MR Egger
- Simple mode
- Weighted median
- Weighted mode

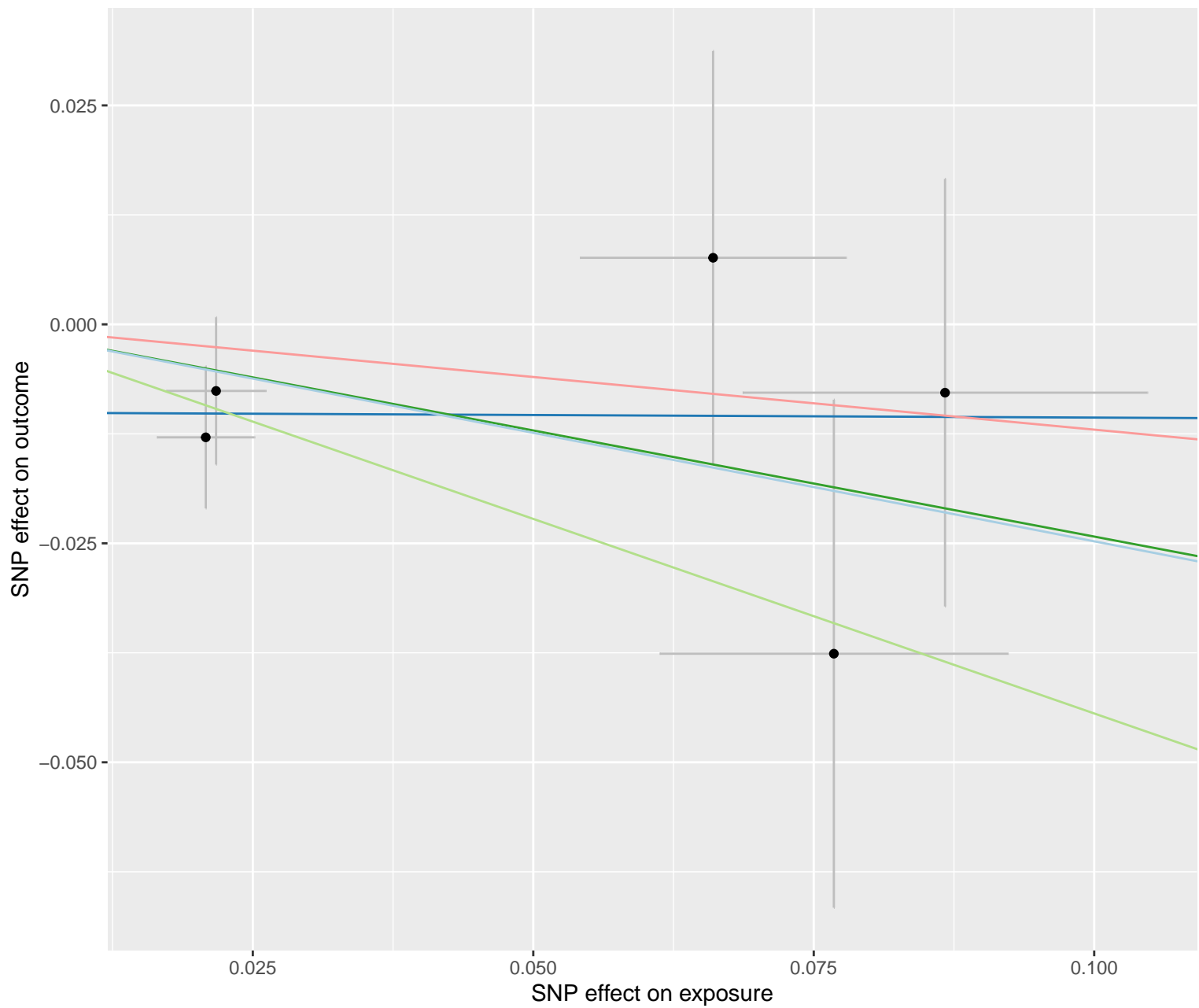

Supplement: Supplementary Data Sheet 3 — Full results of the pairwise Mendelian randomization analyses between ulcerative colitis-associated microbial taxa and ulcerative colitis-associated pyroptosis proteins, used for the downstream mediation analysis. [file DataSheet3.zip › GM_bd_fer_result/GCST90032583+13985_12_SMURF2_SMUF2/scatter.pdf]

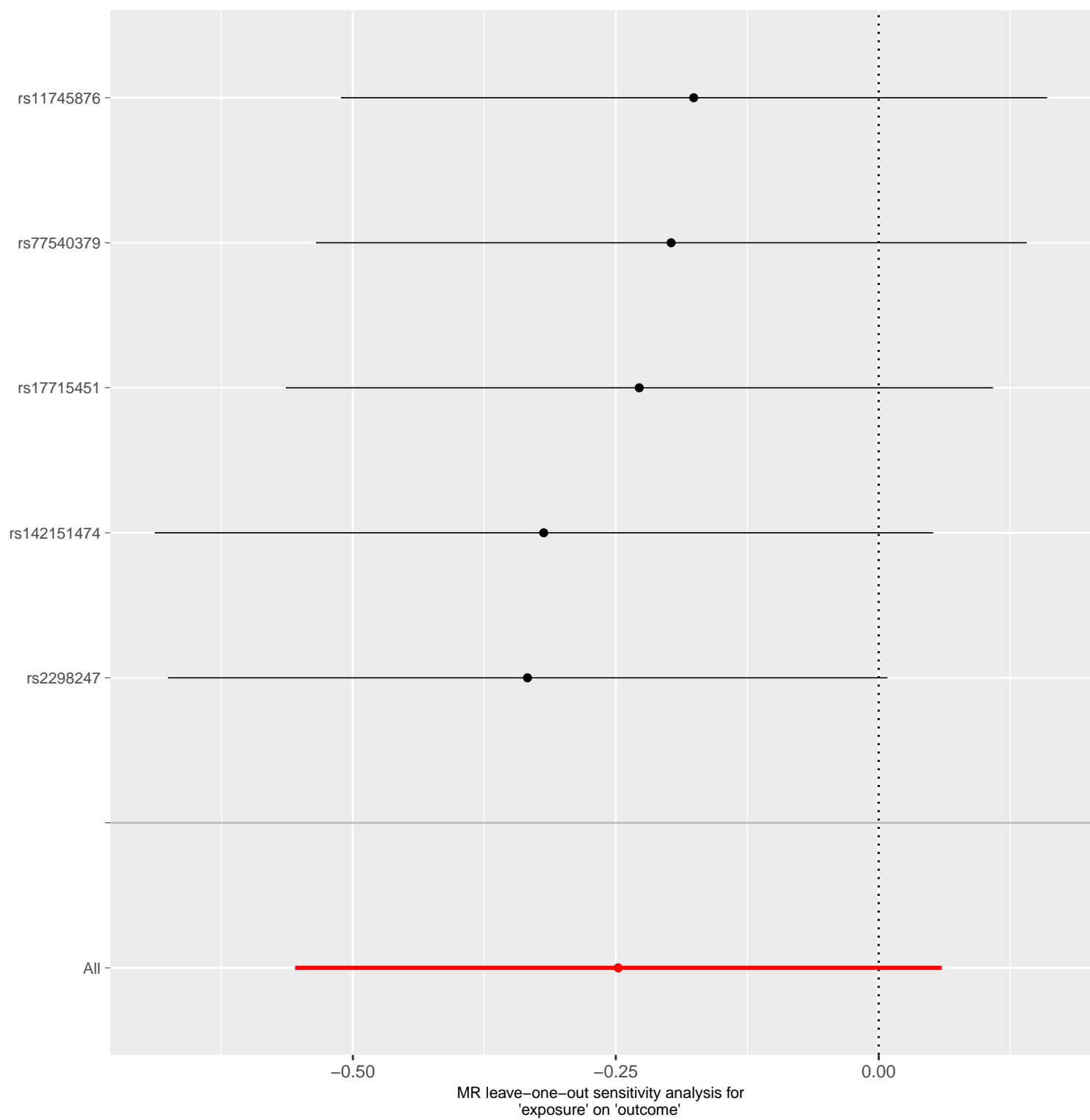

Supplement: Supplementary Data Sheet 3 — Full results of the pairwise Mendelian randomization analyses between ulcerative colitis-associated microbial taxa and ulcerative colitis-associated pyroptosis proteins, used for the downstream mediation analysis. [file DataSheet3.zip › GM_bd_fer_result/GCST90032583+13985_12_SMURF2_SMUF2/sensitivity-analysis.pdf]

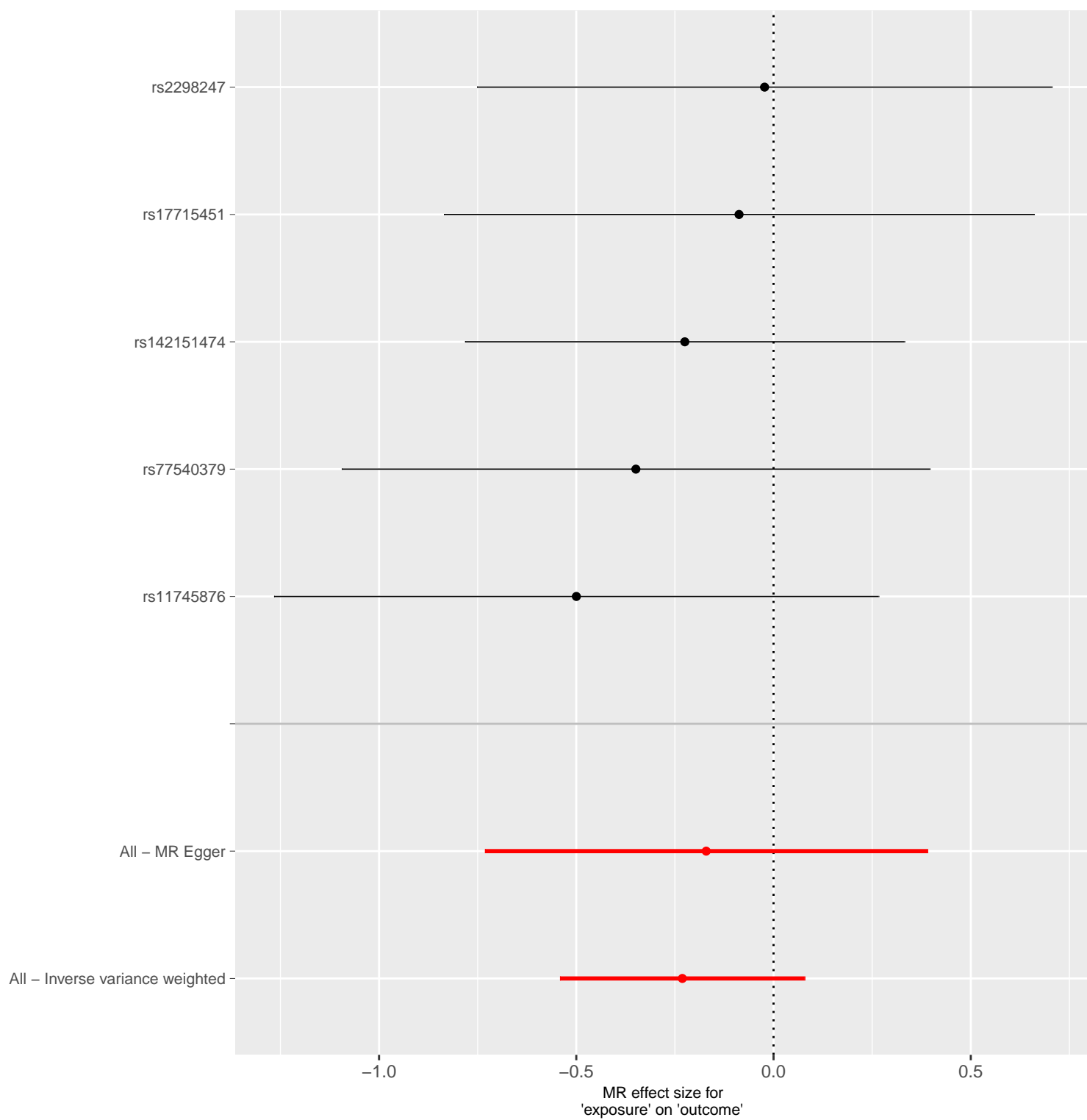

Supplement: Supplementary Data Sheet 3 — Full results of the pairwise Mendelian randomization analyses between ulcerative colitis-associated microbial taxa and ulcerative colitis-associated pyroptosis proteins, used for the downstream mediation analysis. [file DataSheet3.zip › GM_bd_fer_result/GCST90032583+15346_31_IFNG_IFN_g/forest.pdf]
