# Supplementary figures and images for: Integrative Mendelian randomization and experimental validation prioritize KLF4 in the gut microbiota–pyroptosis–barrier axis of ulcerative colitis (part 3 of 3)
Source: Front Immunol. 2026 Mar 16;17:1773990. doi: 10.3389/fimmu.2026.1773990 (PMC13033518; doi:10.3389/fimmu.2026.1773990)

# MR Method

- Inverse variance weighted
- MR Egger

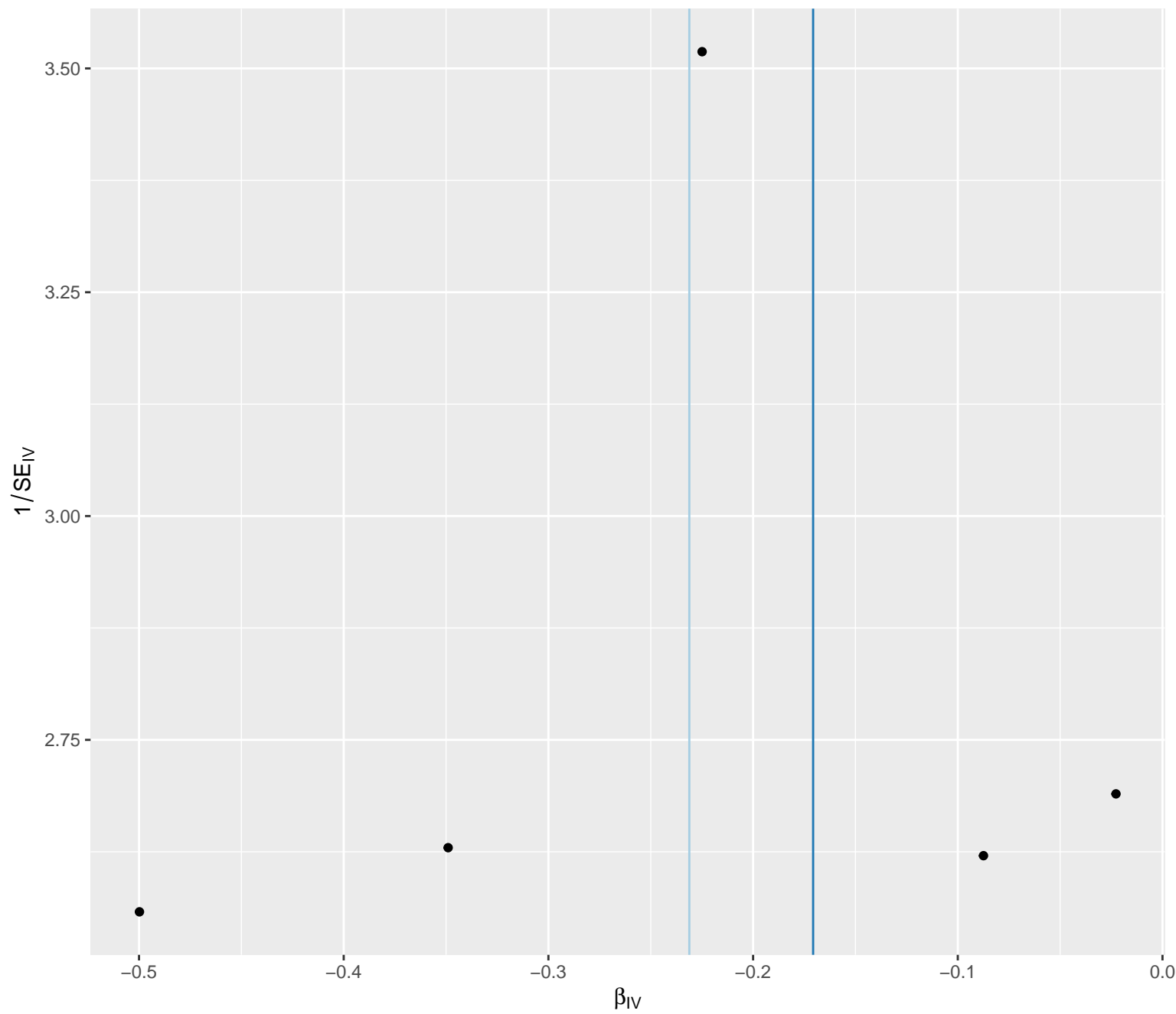

Supplement: Supplementary Data Sheet 3 — Full results of the pairwise Mendelian randomization analyses between ulcerative colitis-associated microbial taxa and ulcerative colitis-associated pyroptosis proteins, used for the downstream mediation analysis. [file DataSheet3.zip › GM_bd_fer_result/GCST90032583+15346_31_IFNG_IFN_g/funnelplot.pdf]

# MR Test

- Inverse variance weighted
- MR Egger
- Simple mode
- Weighted median
- Weighted mode

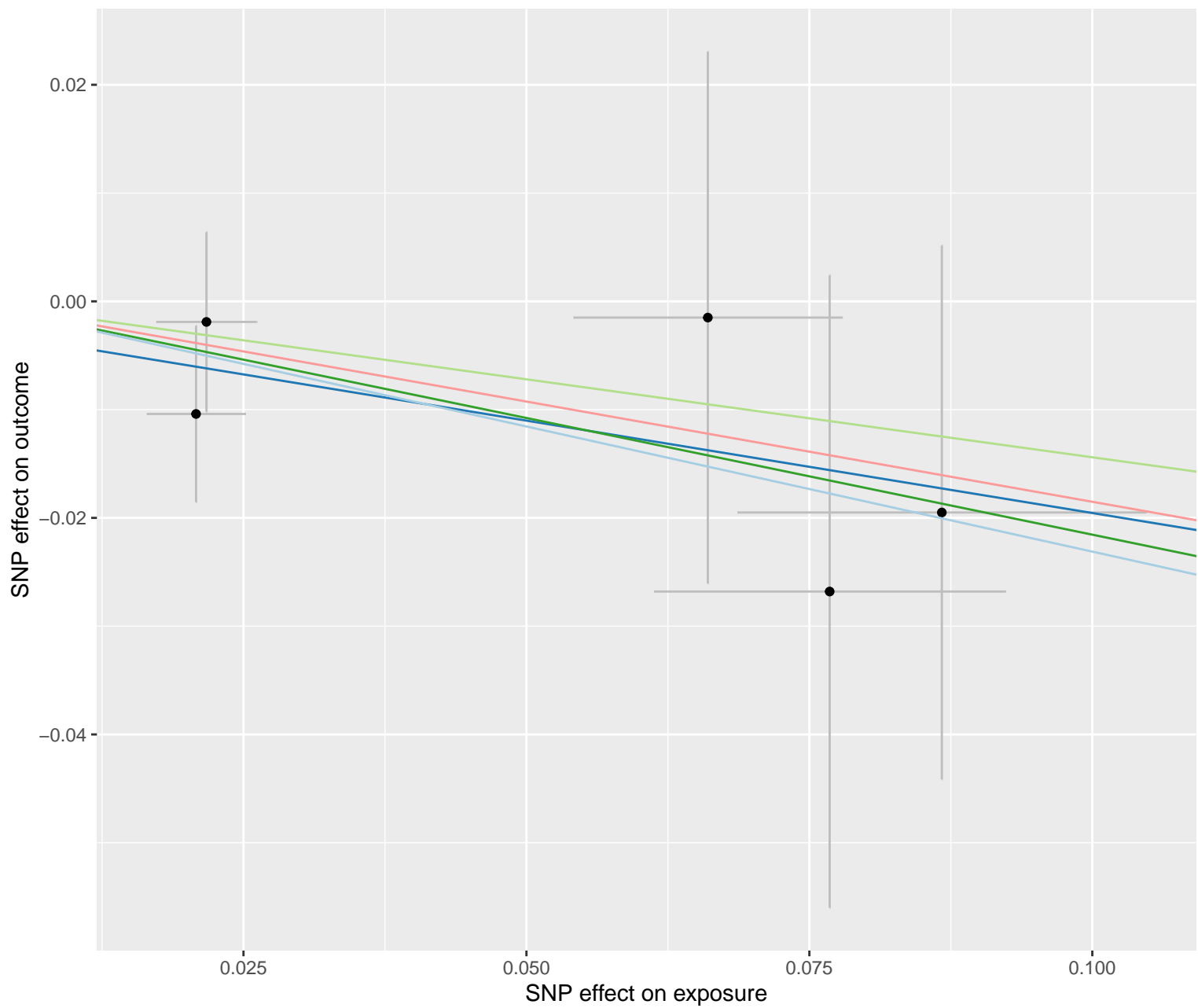

Supplement: Supplementary Data Sheet 3 — Full results of the pairwise Mendelian randomization analyses between ulcerative colitis-associated microbial taxa and ulcerative colitis-associated pyroptosis proteins, used for the downstream mediation analysis. [file DataSheet3.zip › GM_bd_fer_result/GCST90032583+15346_31_IFNG_IFN_g/scatter.pdf]

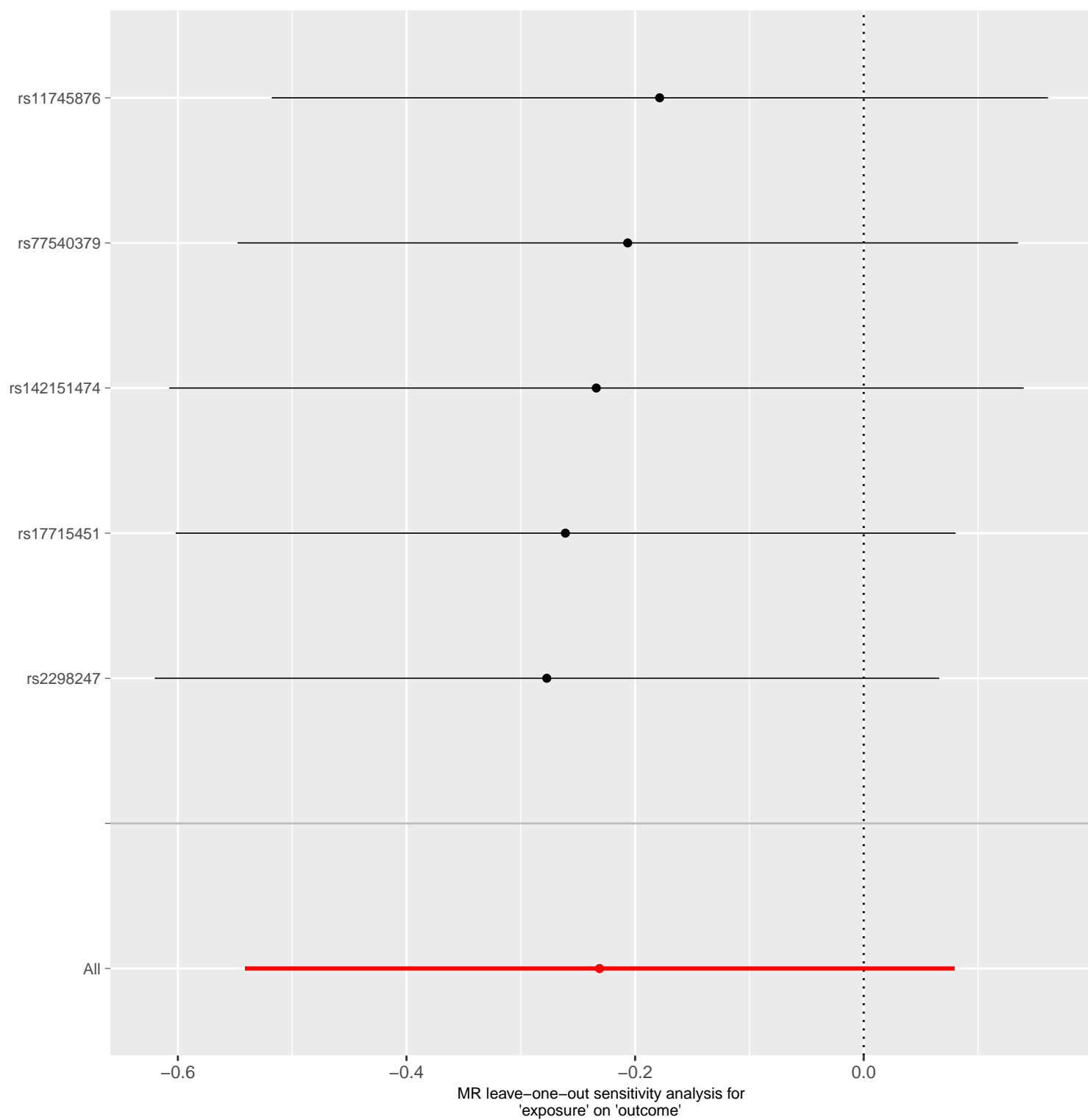

Supplement: Supplementary Data Sheet 3 — Full results of the pairwise Mendelian randomization analyses between ulcerative colitis-associated microbial taxa and ulcerative colitis-associated pyroptosis proteins, used for the downstream mediation analysis. [file DataSheet3.zip › GM_bd_fer_result/GCST90032583+15346_31_IFNG_IFN_g/sensitivity-analysis.pdf]

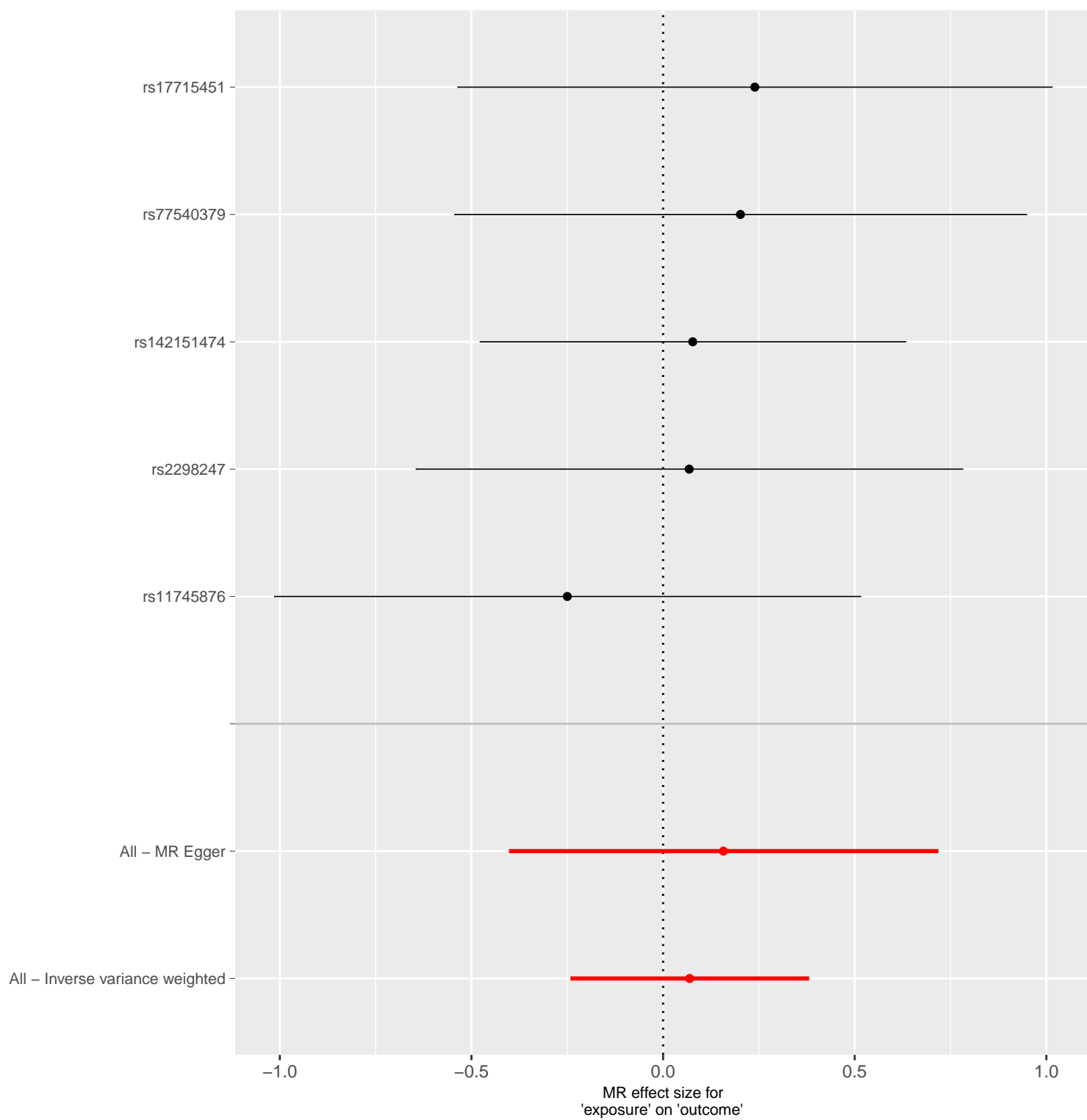

Supplement: Supplementary Data Sheet 3 — Full results of the pairwise Mendelian randomization analyses between ulcerative colitis-associated microbial taxa and ulcerative colitis-associated pyroptosis proteins, used for the downstream mediation analysis. [file DataSheet3.zip › GM_bd_fer_result/GCST90032583+15675_3_CEBPB_CEBPB/forest.pdf]

# MR Method

- Inverse variance weighted
- MR Egger

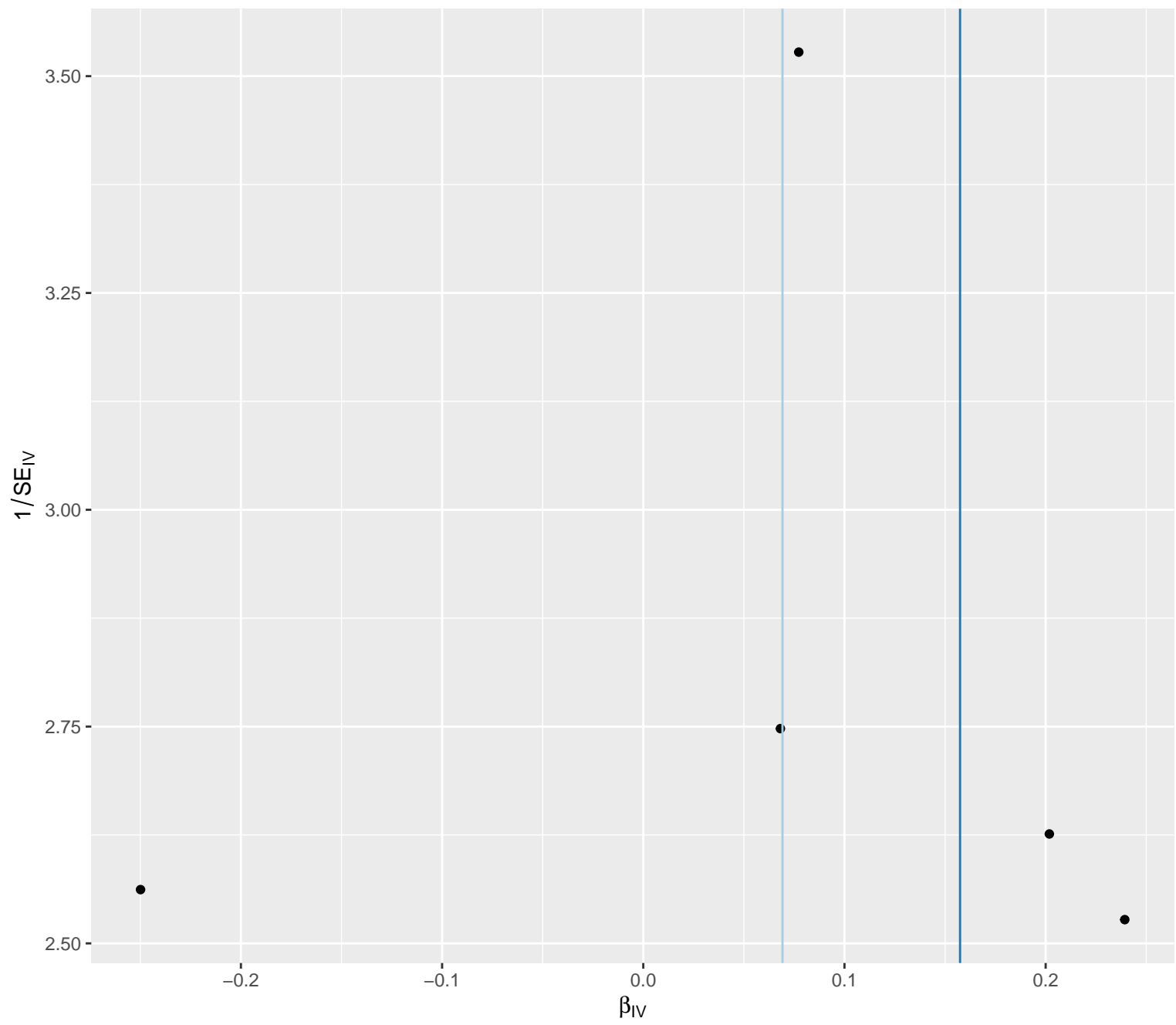

Supplement: Supplementary Data Sheet 3 — Full results of the pairwise Mendelian randomization analyses between ulcerative colitis-associated microbial taxa and ulcerative colitis-associated pyroptosis proteins, used for the downstream mediation analysis. [file DataSheet3.zip › GM_bd_fer_result/GCST90032583+15675_3_CEBPB_CEBPB/funnelplot.pdf]

# MR Test

- Inverse variance weighted
- MR Egger
- Simple mode
- Weighted median
- Weighted mode

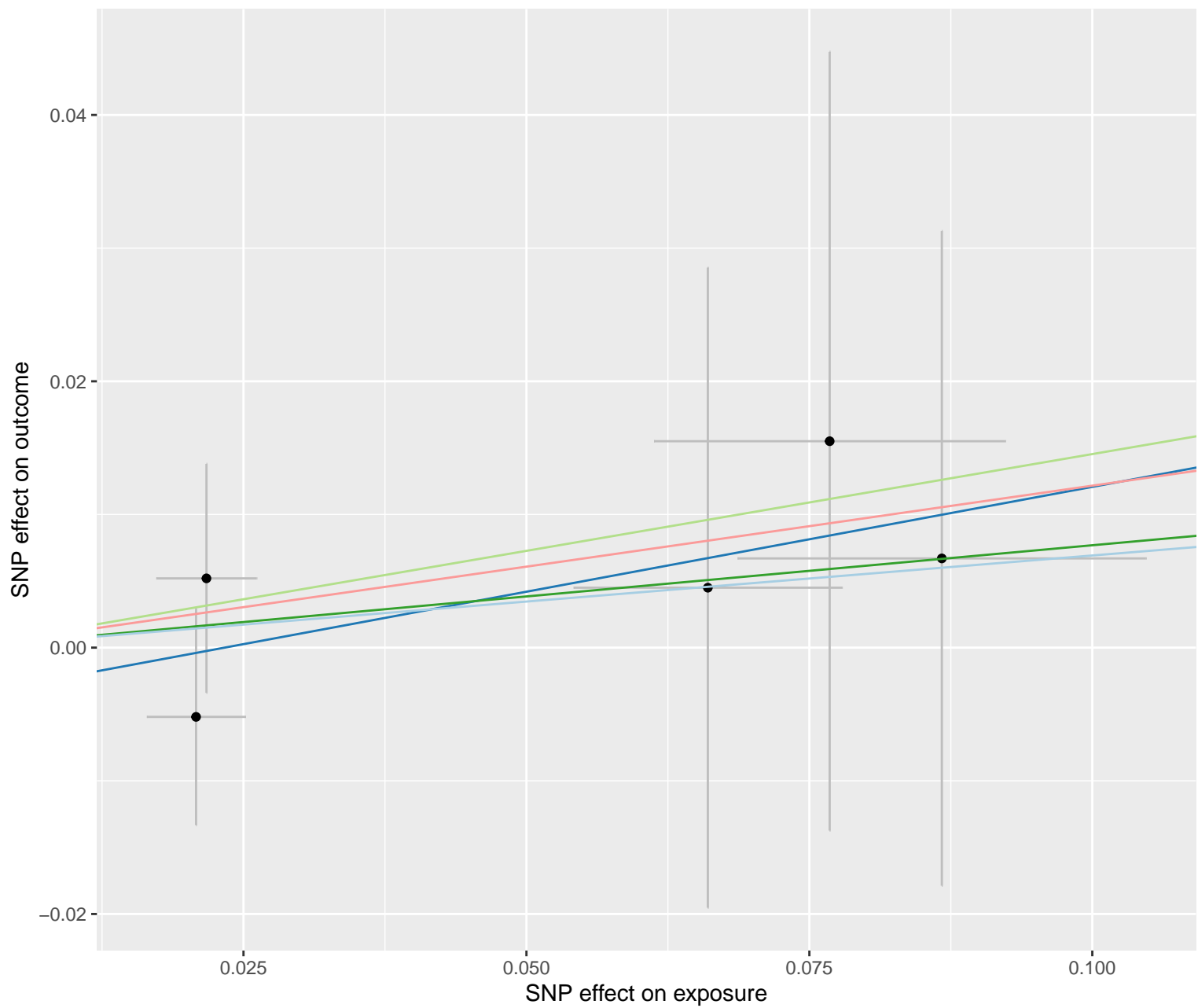

Supplement: Supplementary Data Sheet 3 — Full results of the pairwise Mendelian randomization analyses between ulcerative colitis-associated microbial taxa and ulcerative colitis-associated pyroptosis proteins, used for the downstream mediation analysis. [file DataSheet3.zip › GM_bd_fer_result/GCST90032583+15675_3_CEBPB_CEBPB/scatter.pdf]

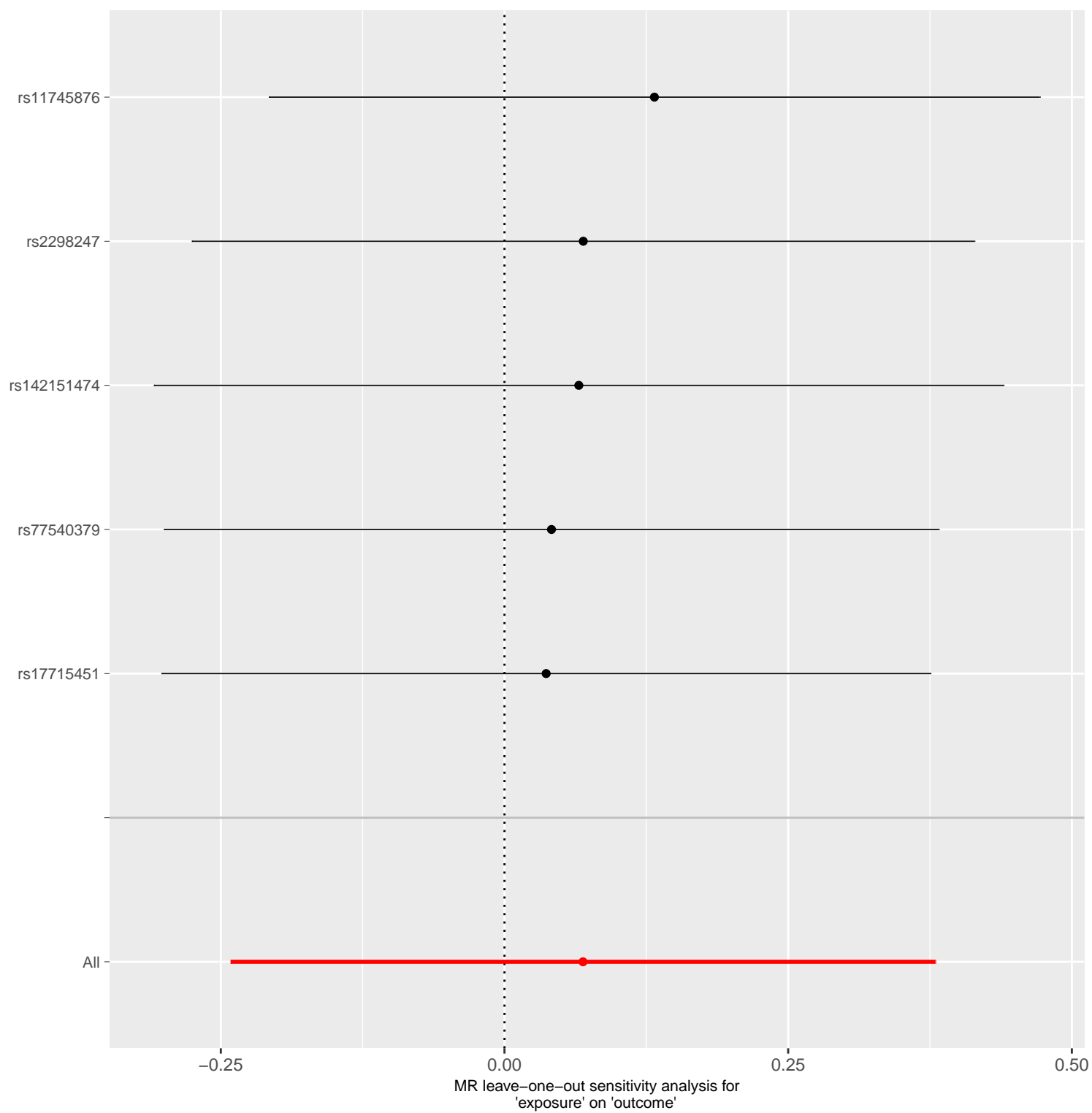

Supplement: Supplementary Data Sheet 3 — Full results of the pairwise Mendelian randomization analyses between ulcerative colitis-associated microbial taxa and ulcerative colitis-associated pyroptosis proteins, used for the downstream mediation analysis. [file DataSheet3.zip › GM_bd_fer_result/GCST90032583+15675_3_CEBPB_CEBPB/sensitivity-analysis.pdf]

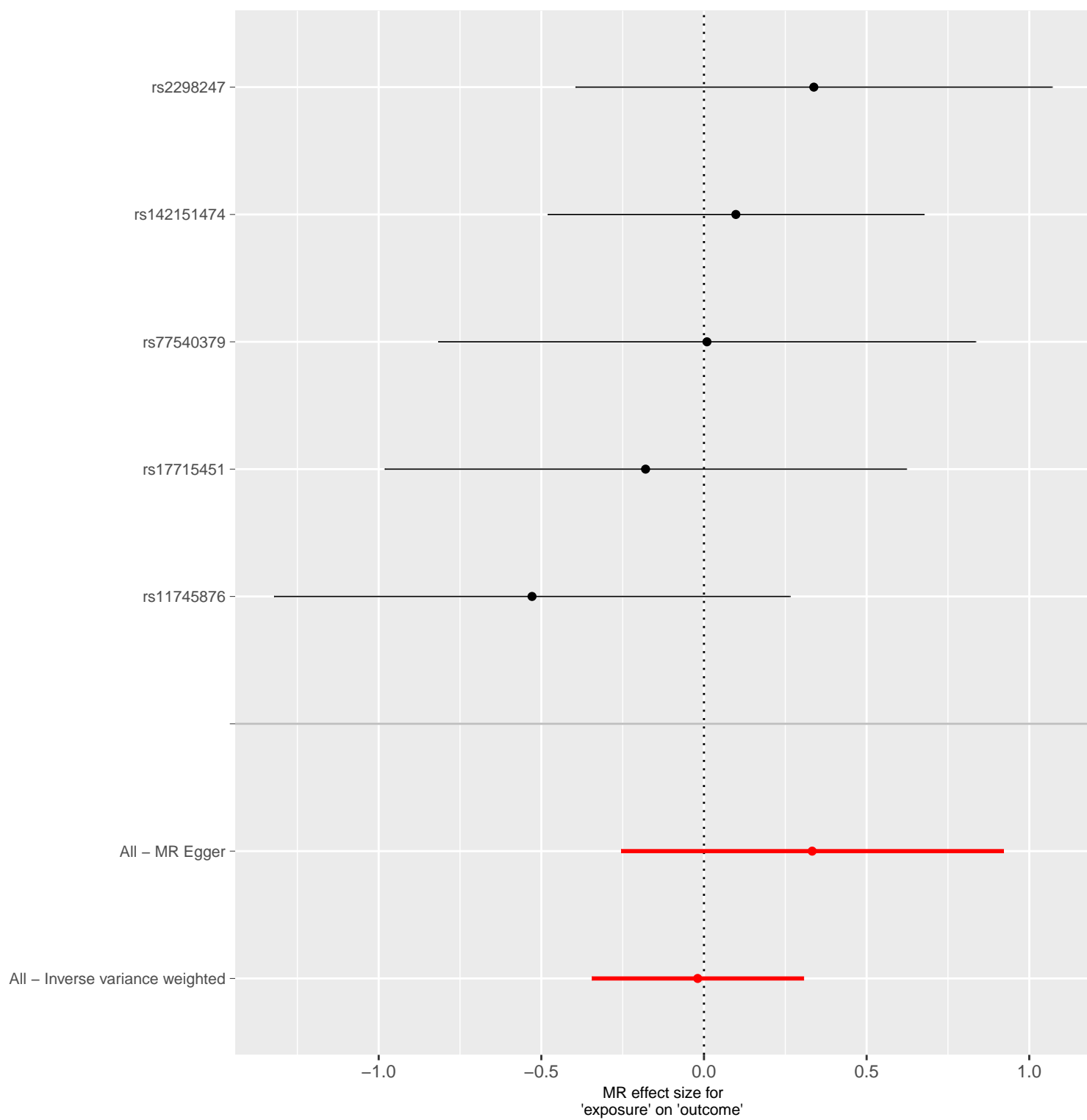

Supplement: Supplementary Data Sheet 3 — Full results of the pairwise Mendelian randomization analyses between ulcerative colitis-associated microbial taxa and ulcerative colitis-associated pyroptosis proteins, used for the downstream mediation analysis. [file DataSheet3.zip › GM_bd_fer_result/GCST90032583+16914_104_CD14_sCD14/forest.pdf]

# MR Method

- Inverse variance weighted
- MR Egger

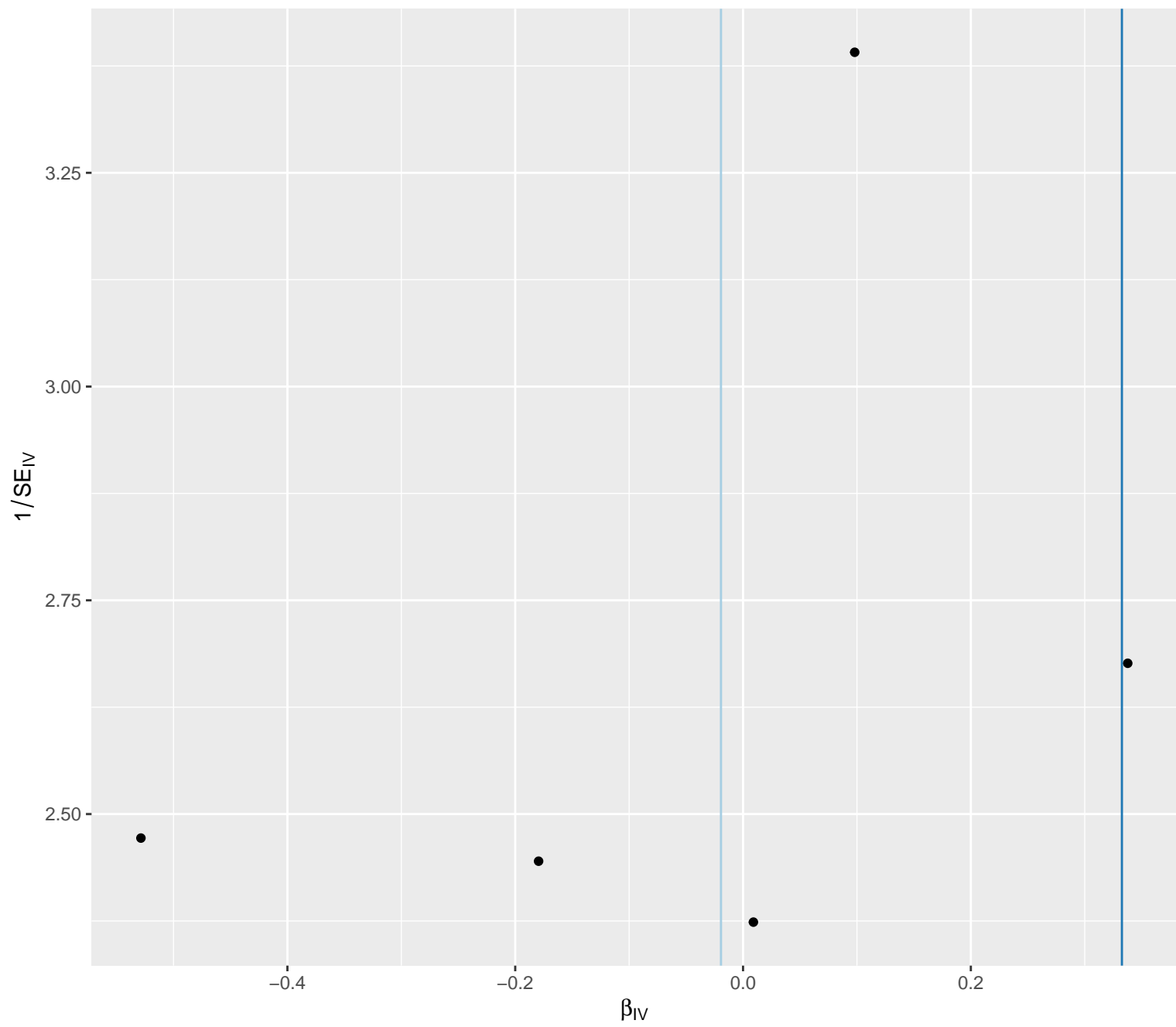

Supplement: Supplementary Data Sheet 3 — Full results of the pairwise Mendelian randomization analyses between ulcerative colitis-associated microbial taxa and ulcerative colitis-associated pyroptosis proteins, used for the downstream mediation analysis. [file DataSheet3.zip › GM_bd_fer_result/GCST90032583+16914_104_CD14_sCD14/funnelplot.pdf]

# MR Test

- Inverse variance weighted
- MR Egger
- Simple mode
- Weighted median
- Weighted mode

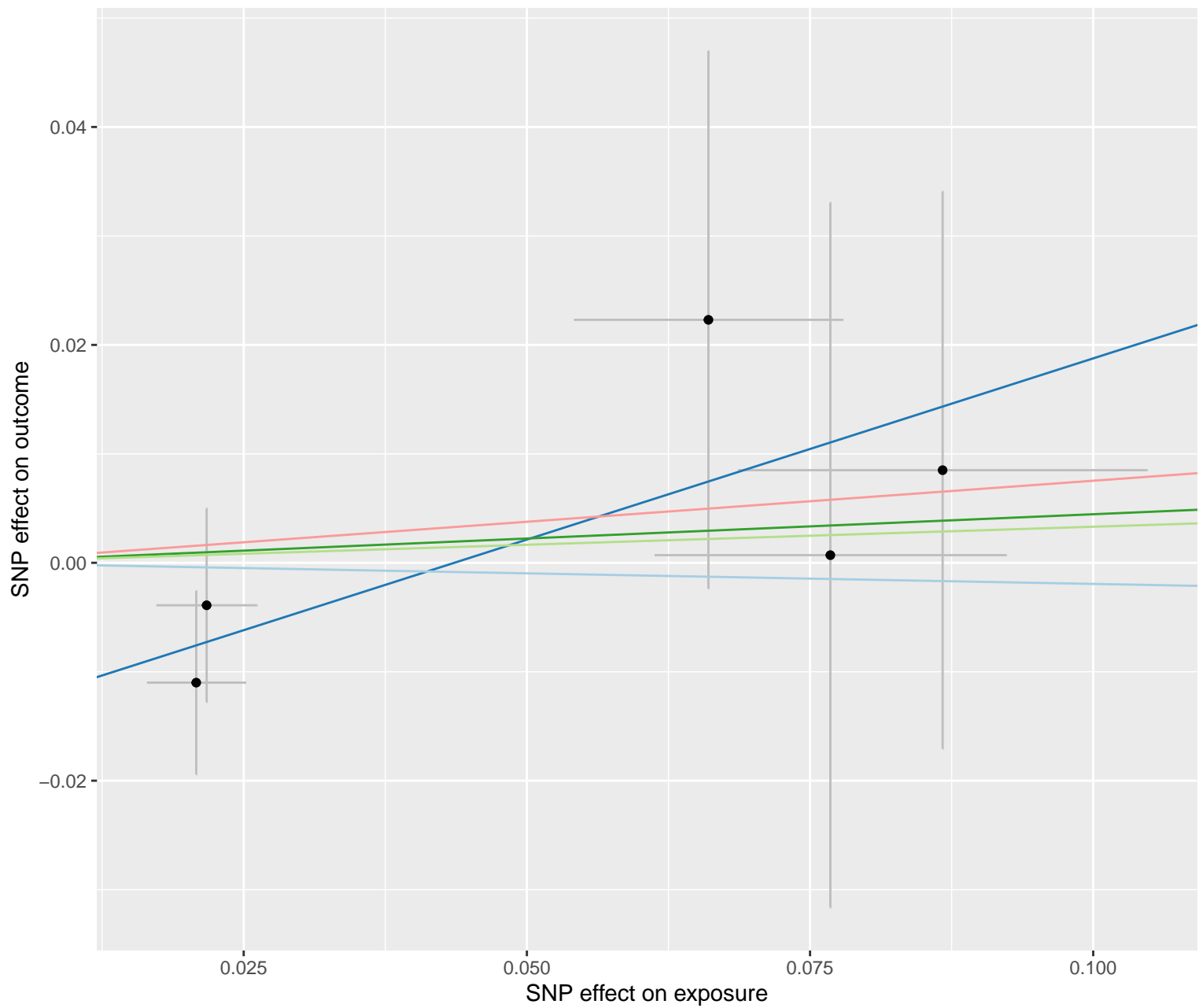

Supplement: Supplementary Data Sheet 3 — Full results of the pairwise Mendelian randomization analyses between ulcerative colitis-associated microbial taxa and ulcerative colitis-associated pyroptosis proteins, used for the downstream mediation analysis. [file DataSheet3.zip › GM_bd_fer_result/GCST90032583+16914_104_CD14_sCD14/scatter.pdf]

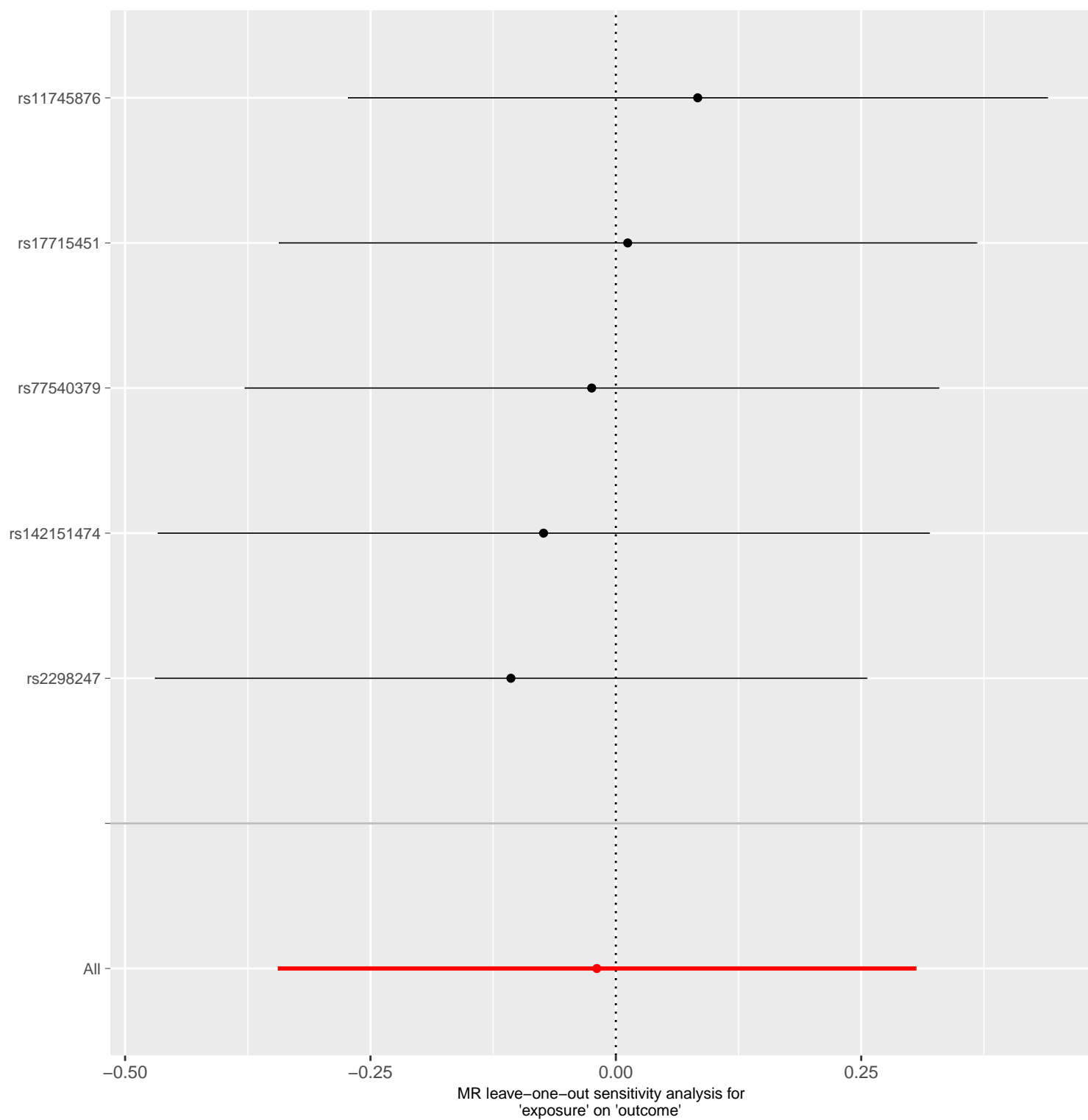

Supplement: Supplementary Data Sheet 3 — Full results of the pairwise Mendelian randomization analyses between ulcerative colitis-associated microbial taxa and ulcerative colitis-associated pyroptosis proteins, used for the downstream mediation analysis. [file DataSheet3.zip › GM_bd_fer_result/GCST90032583+16914_104_CD14_sCD14/sensitivity-analysis.pdf]

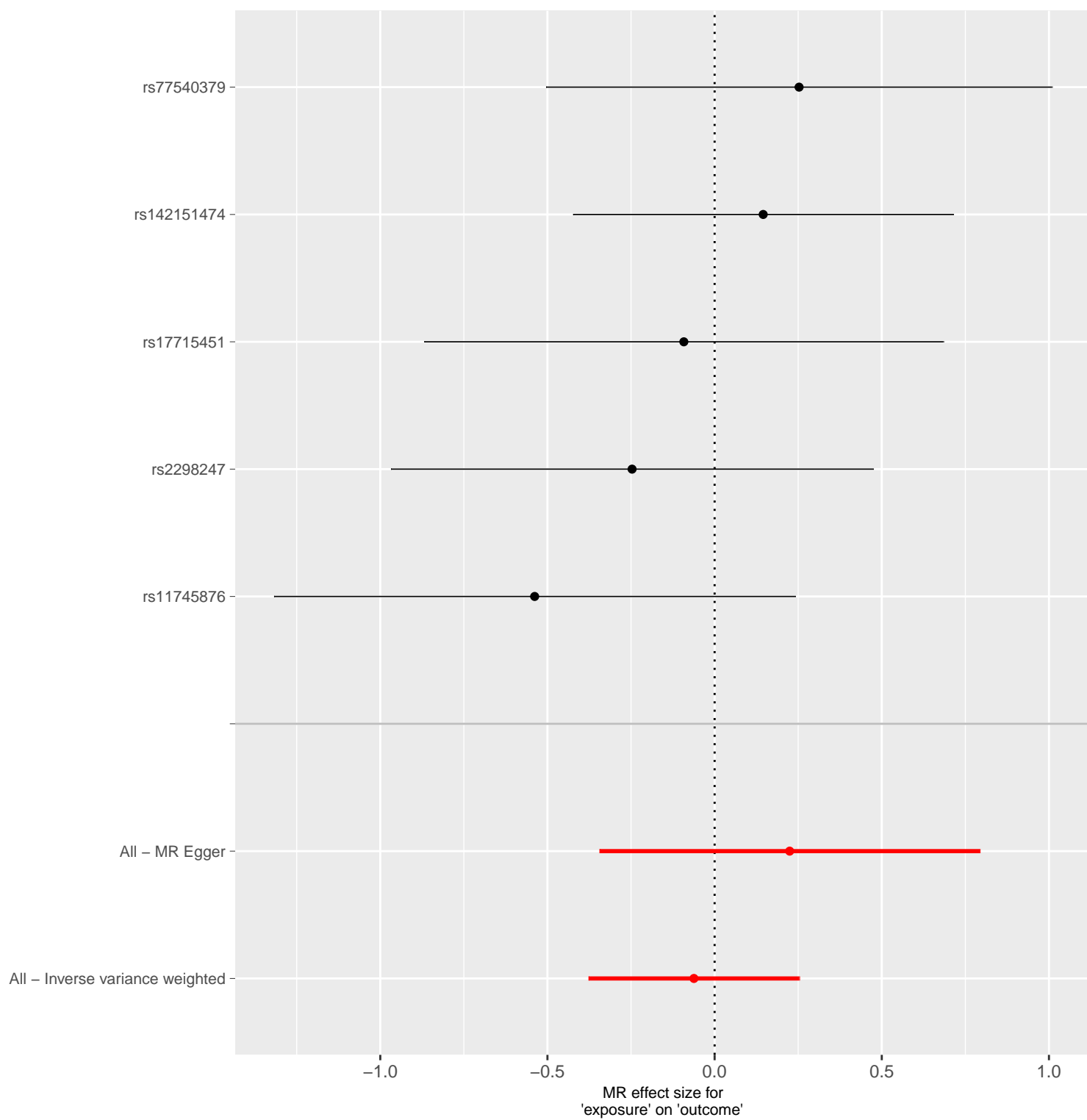

Supplement: Supplementary Data Sheet 3 — Full results of the pairwise Mendelian randomization analyses between ulcerative colitis-associated microbial taxa and ulcerative colitis-associated pyroptosis proteins, used for the downstream mediation analysis. [file DataSheet3.zip › GM_bd_fer_result/GCST90032583+17155_1_VPS28_VPS28_protein_homolog/forest.pdf]

# MR Method

- Inverse variance weighted
- MR Egger

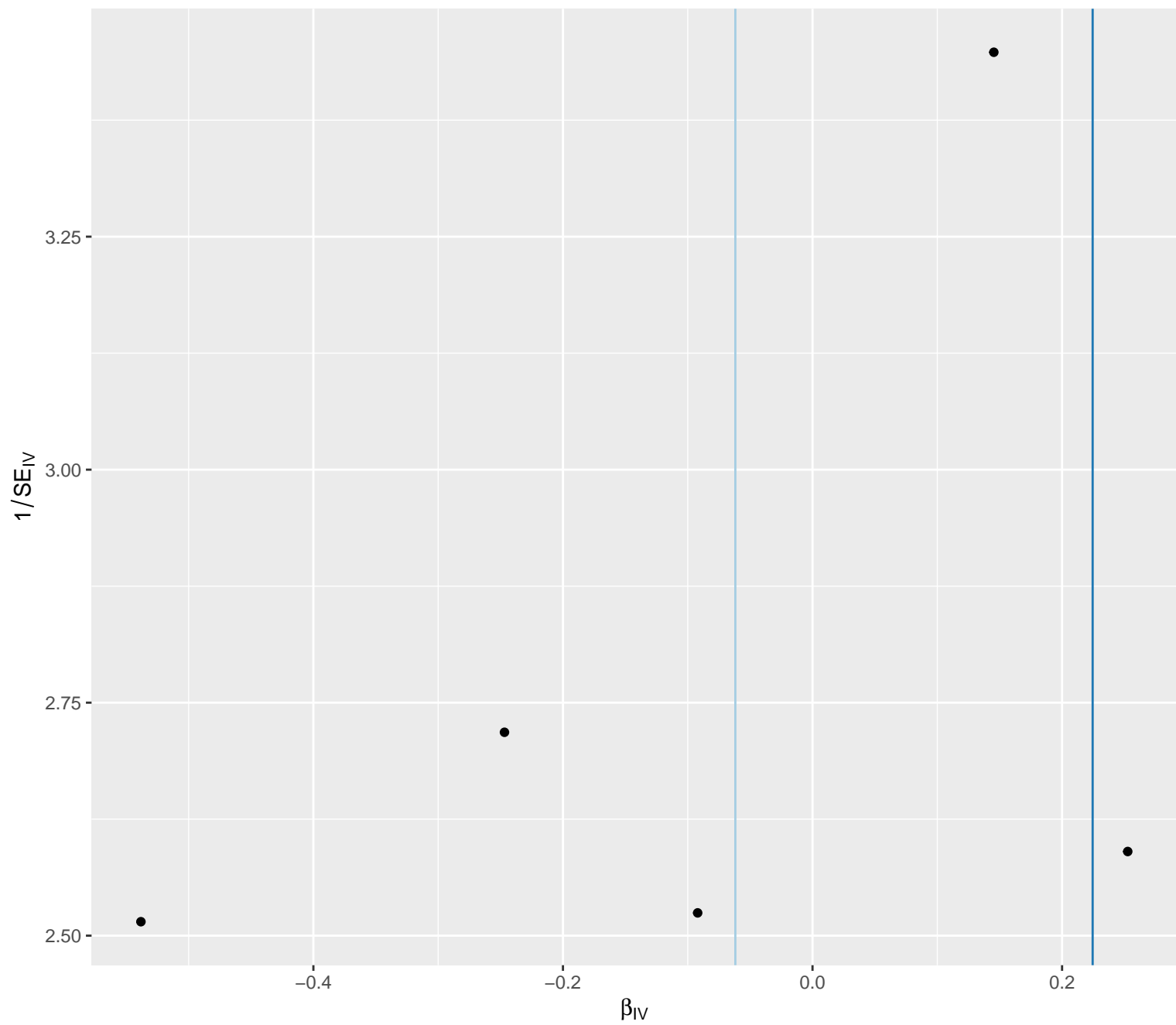

Supplement: Supplementary Data Sheet 3 — Full results of the pairwise Mendelian randomization analyses between ulcerative colitis-associated microbial taxa and ulcerative colitis-associated pyroptosis proteins, used for the downstream mediation analysis. [file DataSheet3.zip › GM_bd_fer_result/GCST90032583+17155_1_VPS28_VPS28_protein_homolog/funnelplot.pdf]

# MR Test

- Inverse variance weighted
- MR Egger
- Simple mode
- Weighted median
- Weighted mode

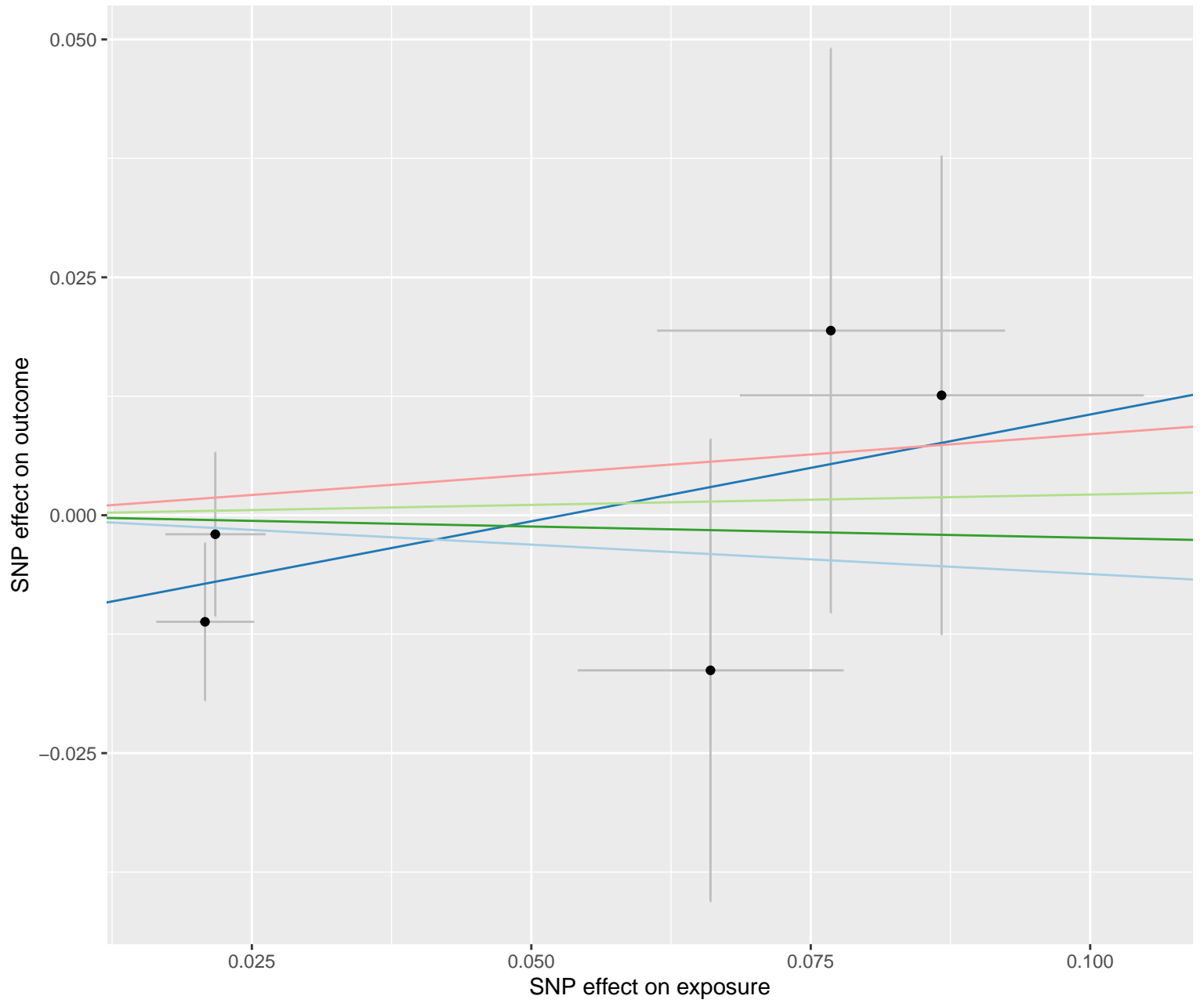

Supplement: Supplementary Data Sheet 3 — Full results of the pairwise Mendelian randomization analyses between ulcerative colitis-associated microbial taxa and ulcerative colitis-associated pyroptosis proteins, used for the downstream mediation analysis. [file DataSheet3.zip › GM_bd_fer_result/GCST90032583+17155_1_VPS28_VPS28_protein_homolog/scatter.pdf]

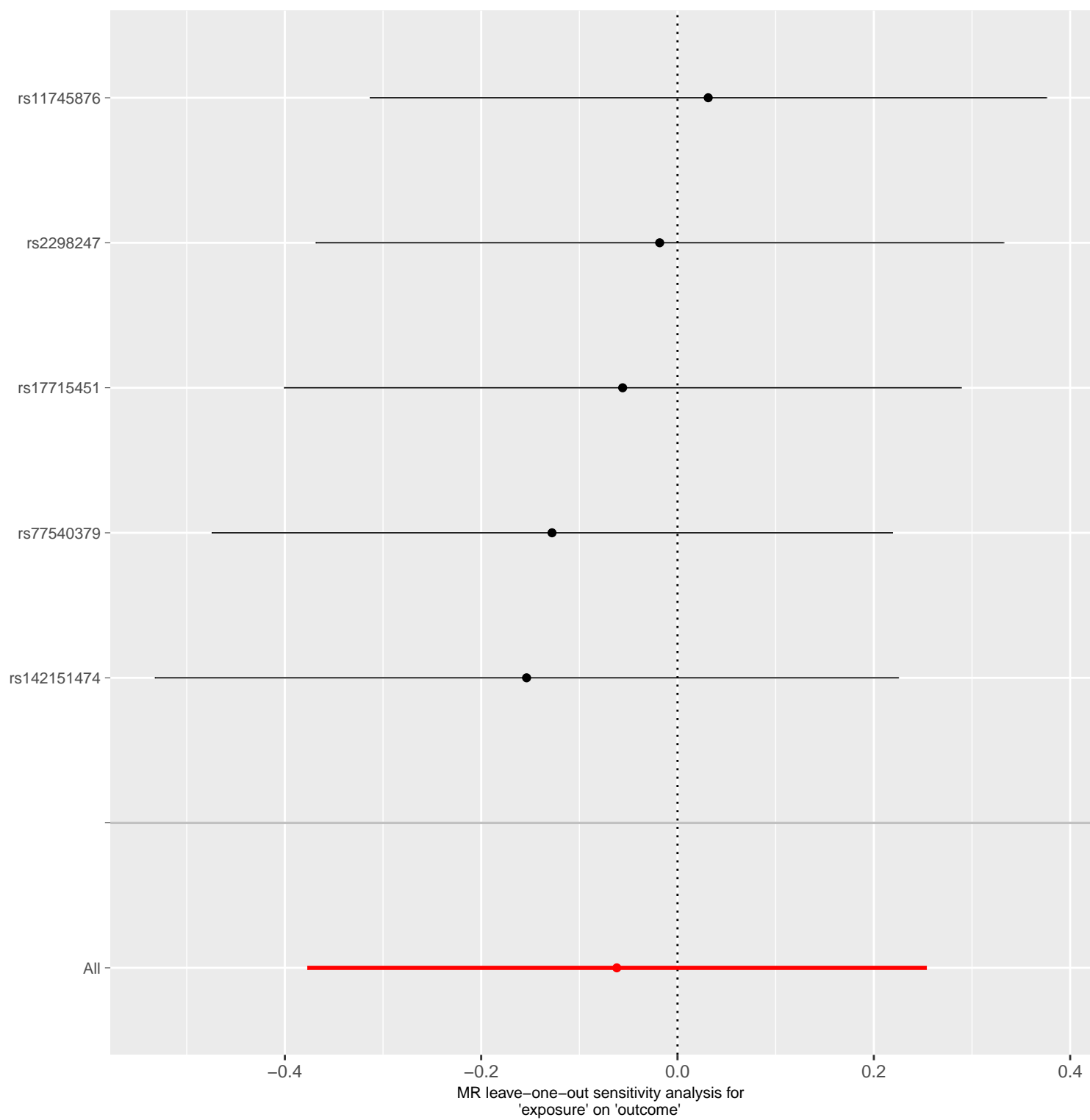

Supplement: Supplementary Data Sheet 3 — Full results of the pairwise Mendelian randomization analyses between ulcerative colitis-associated microbial taxa and ulcerative colitis-associated pyroptosis proteins, used for the downstream mediation analysis. [file DataSheet3.zip › GM_bd_fer_result/GCST90032583+17155_1_VPS28_VPS28_protein_homolog/sensitivity-analysis.pdf]

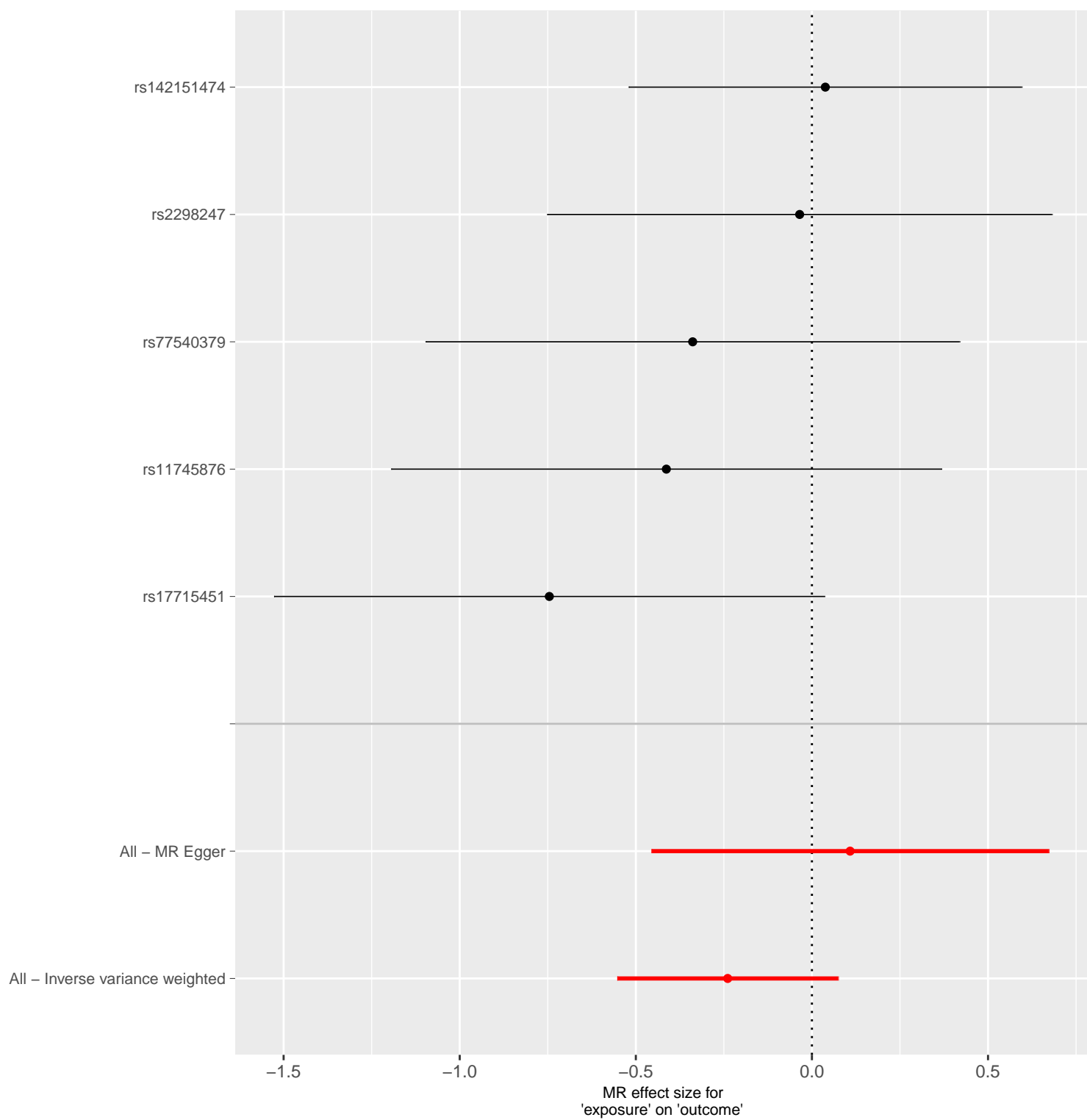

Supplement: Supplementary Data Sheet 3 — Full results of the pairwise Mendelian randomization analyses between ulcerative colitis-associated microbial taxa and ulcerative colitis-associated pyroptosis proteins, used for the downstream mediation analysis. [file DataSheet3.zip › GM_bd_fer_result/GCST90032583+17175_5_MAP2K6_MP2K6/forest.pdf]

# MR Method

- Inverse variance weighted
- MR Egger

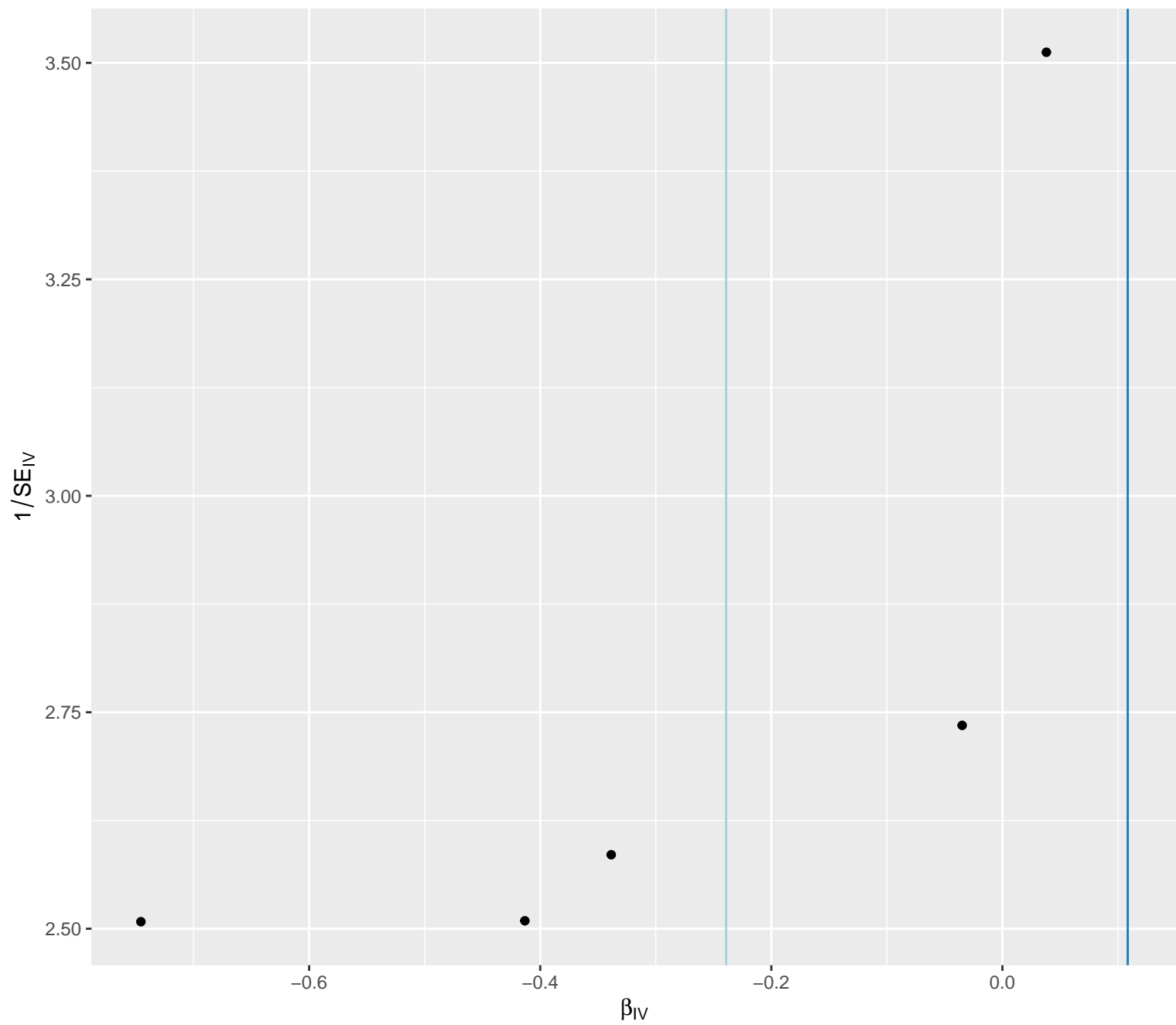

Supplement: Supplementary Data Sheet 3 — Full results of the pairwise Mendelian randomization analyses between ulcerative colitis-associated microbial taxa and ulcerative colitis-associated pyroptosis proteins, used for the downstream mediation analysis. [file DataSheet3.zip › GM_bd_fer_result/GCST90032583+17175_5_MAP2K6_MP2K6/funnelplot.pdf]

# MR Test

- Inverse variance weighted
- MR Egger
- Simple mode
- Weighted median
- Weighted mode

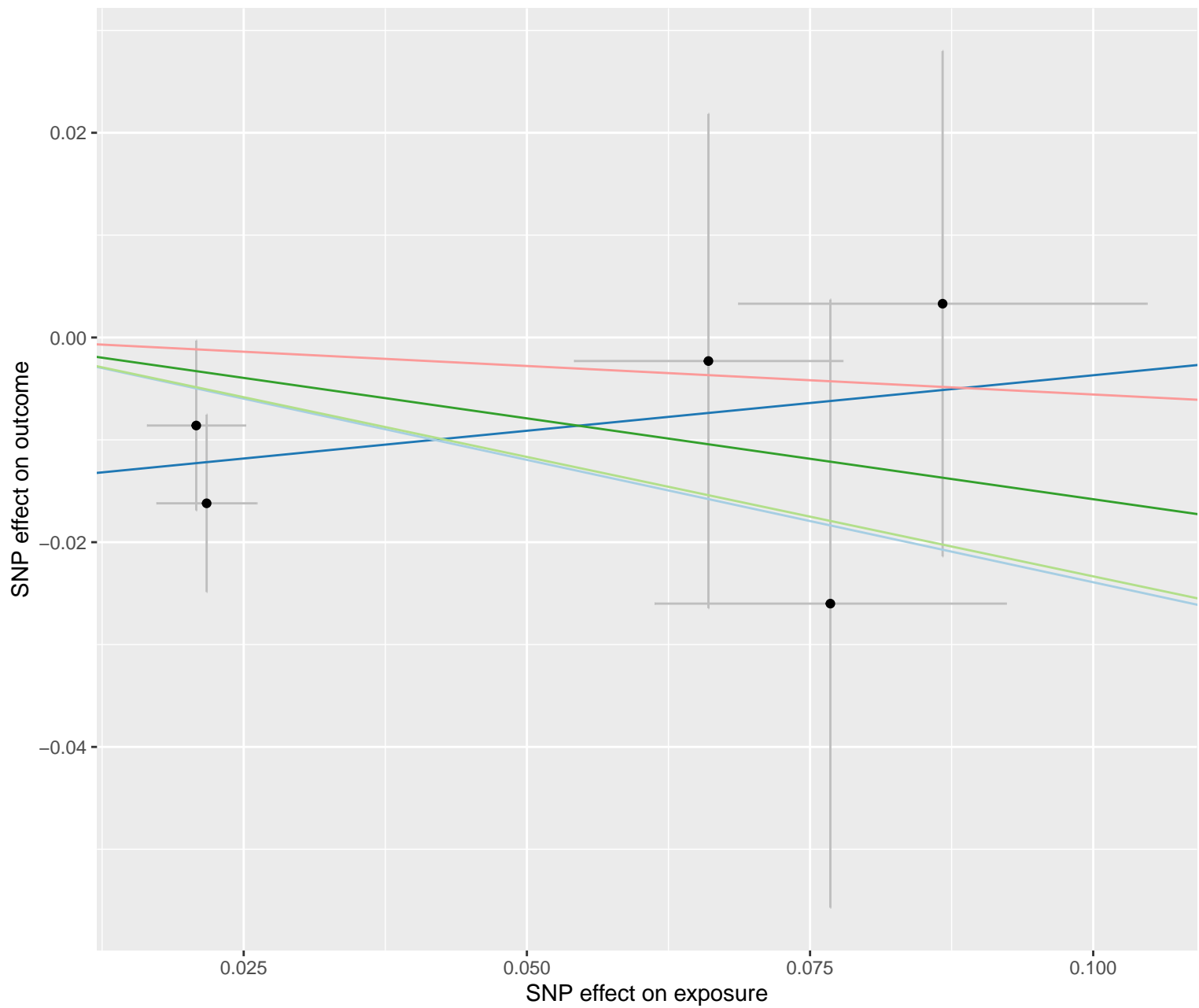

Supplement: Supplementary Data Sheet 3 — Full results of the pairwise Mendelian randomization analyses between ulcerative colitis-associated microbial taxa and ulcerative colitis-associated pyroptosis proteins, used for the downstream mediation analysis. [file DataSheet3.zip › GM_bd_fer_result/GCST90032583+17175_5_MAP2K6_MP2K6/scatter.pdf]

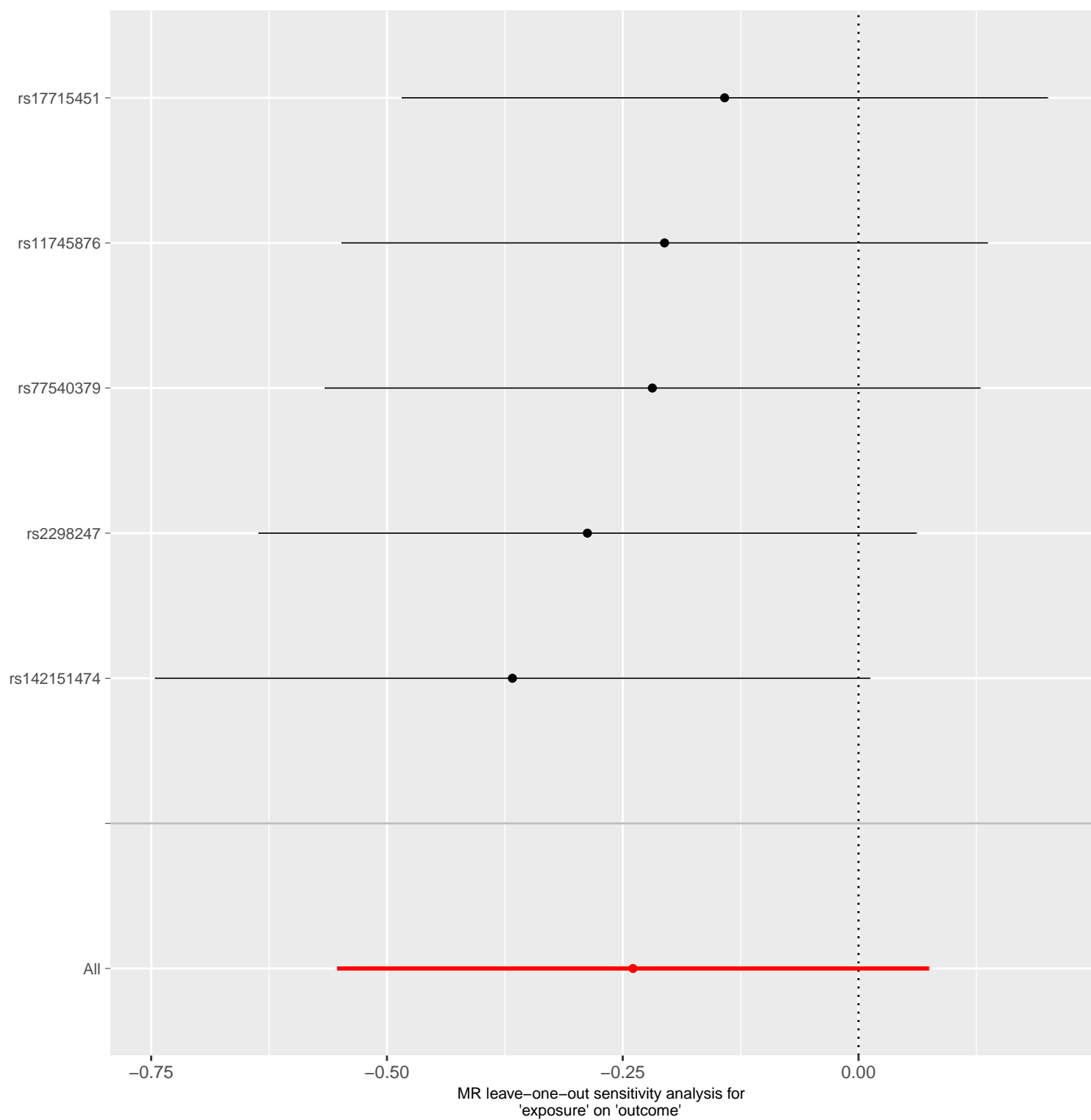

Supplement: Supplementary Data Sheet 3 — Full results of the pairwise Mendelian randomization analyses between ulcerative colitis-associated microbial taxa and ulcerative colitis-associated pyroptosis proteins, used for the downstream mediation analysis. [file DataSheet3.zip › GM_bd_fer_result/GCST90032583+17175_5_MAP2K6_MP2K6/sensitivity-analysis.pdf]

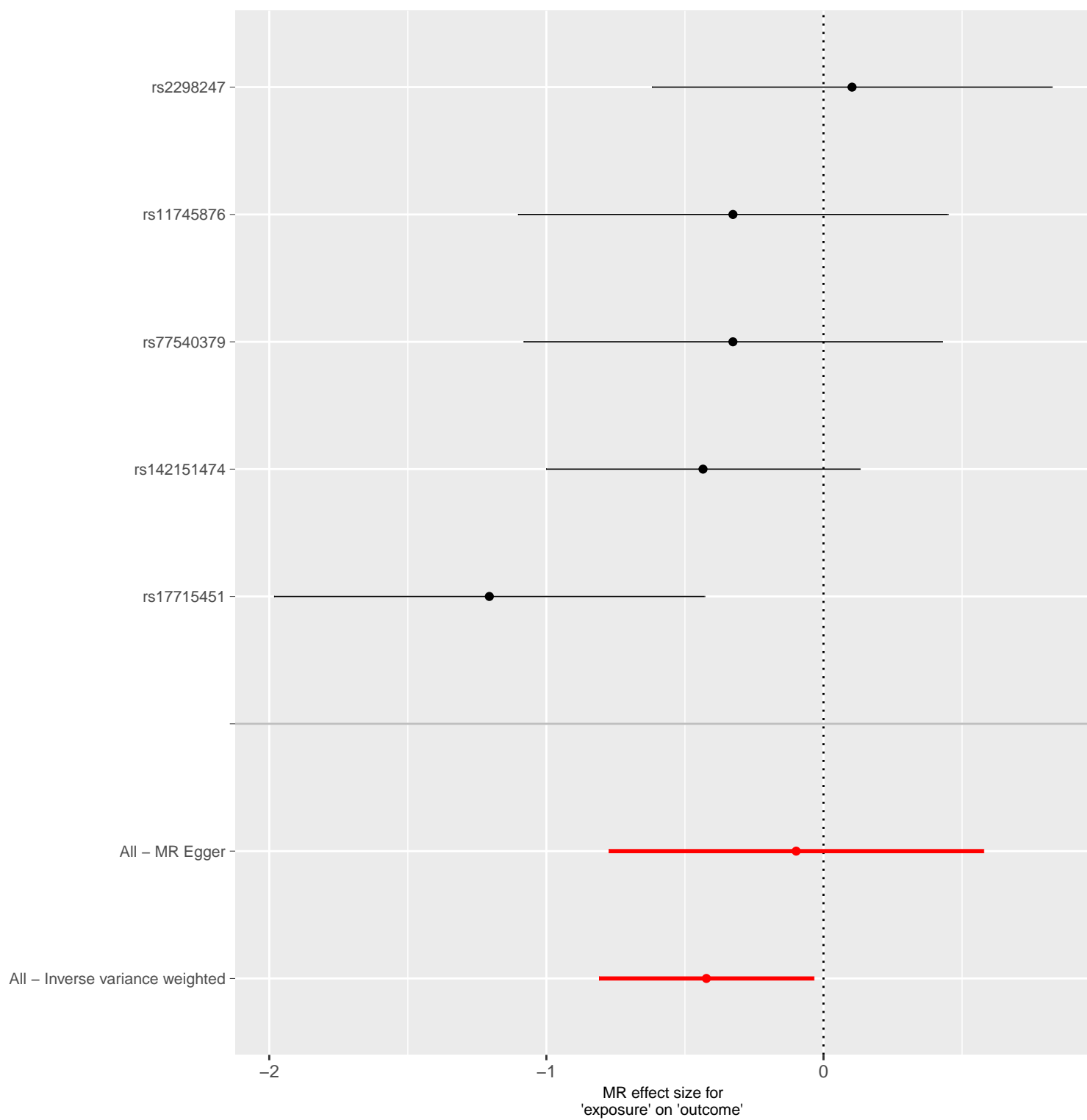

Supplement: Supplementary Data Sheet 3 — Full results of the pairwise Mendelian randomization analyses between ulcerative colitis-associated microbial taxa and ulcerative colitis-associated pyroptosis proteins, used for the downstream mediation analysis. [file DataSheet3.zip › GM_bd_fer_result/GCST90032583+17350_13_CHMP2B_CHM2B/forest.pdf]

# MR Method

Inverse variance weighted

MR Egger

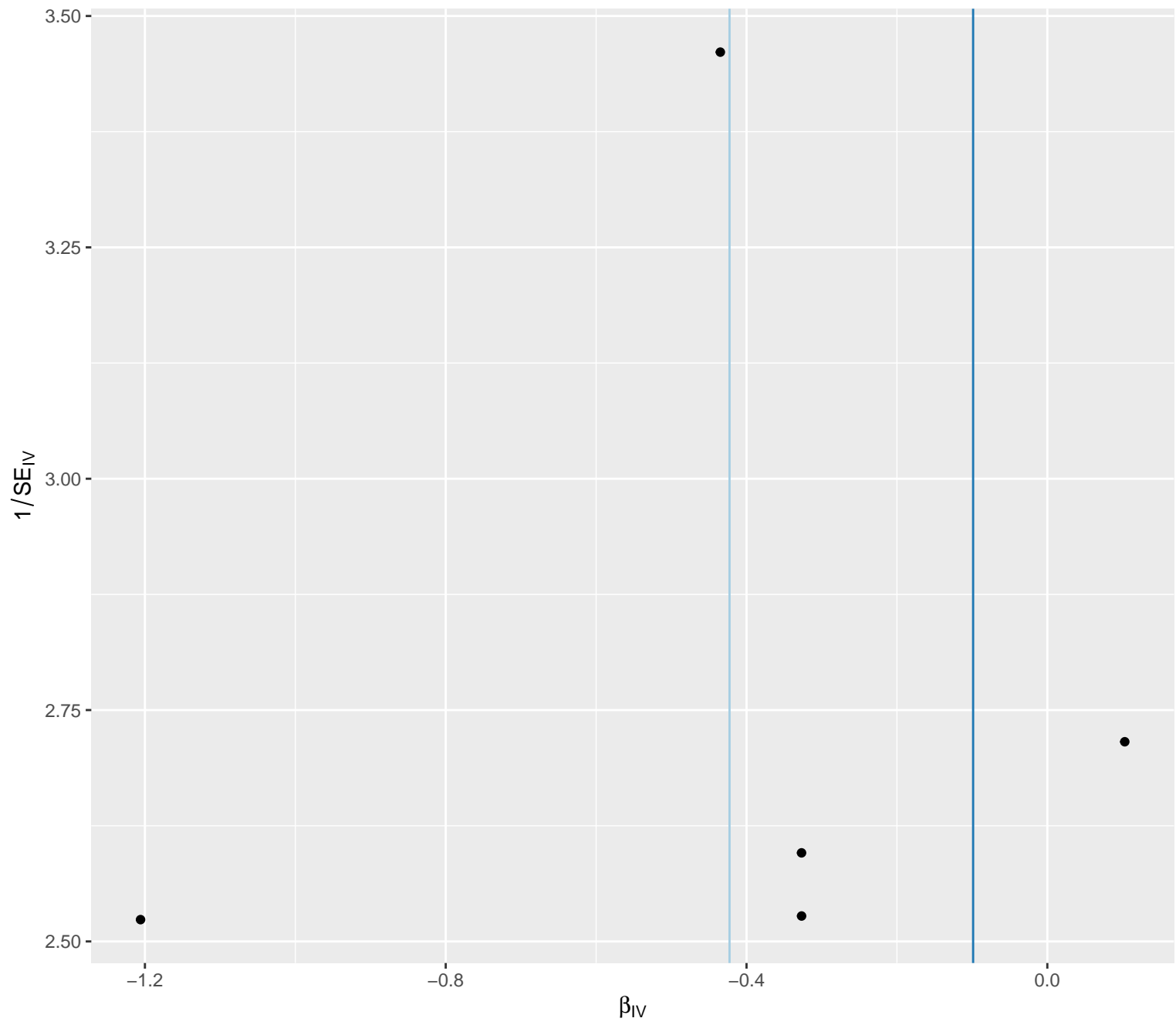

Supplement: Supplementary Data Sheet 3 — Full results of the pairwise Mendelian randomization analyses between ulcerative colitis-associated microbial taxa and ulcerative colitis-associated pyroptosis proteins, used for the downstream mediation analysis. [file DataSheet3.zip › GM_bd_fer_result/GCST90032583+17350_13_CHMP2B_CHM2B/funnelplot.pdf]

# MR Test

- Inverse variance weighted
- MR Egger
- Simple mode
- Weighted median
- Weighted mode

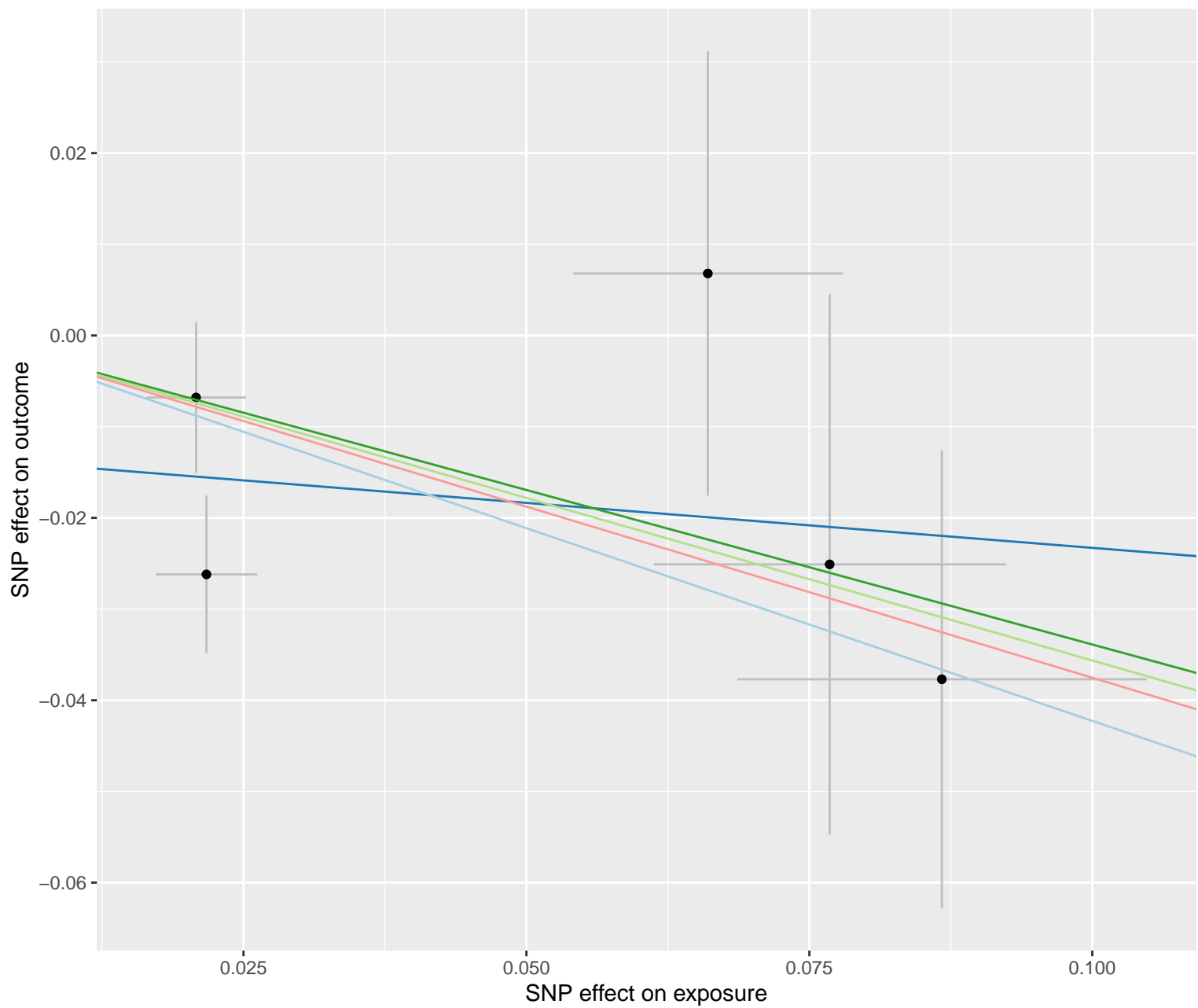

Supplement: Supplementary Data Sheet 3 — Full results of the pairwise Mendelian randomization analyses between ulcerative colitis-associated microbial taxa and ulcerative colitis-associated pyroptosis proteins, used for the downstream mediation analysis. [file DataSheet3.zip › GM_bd_fer_result/GCST90032583+17350_13_CHMP2B_CHM2B/scatter.pdf]

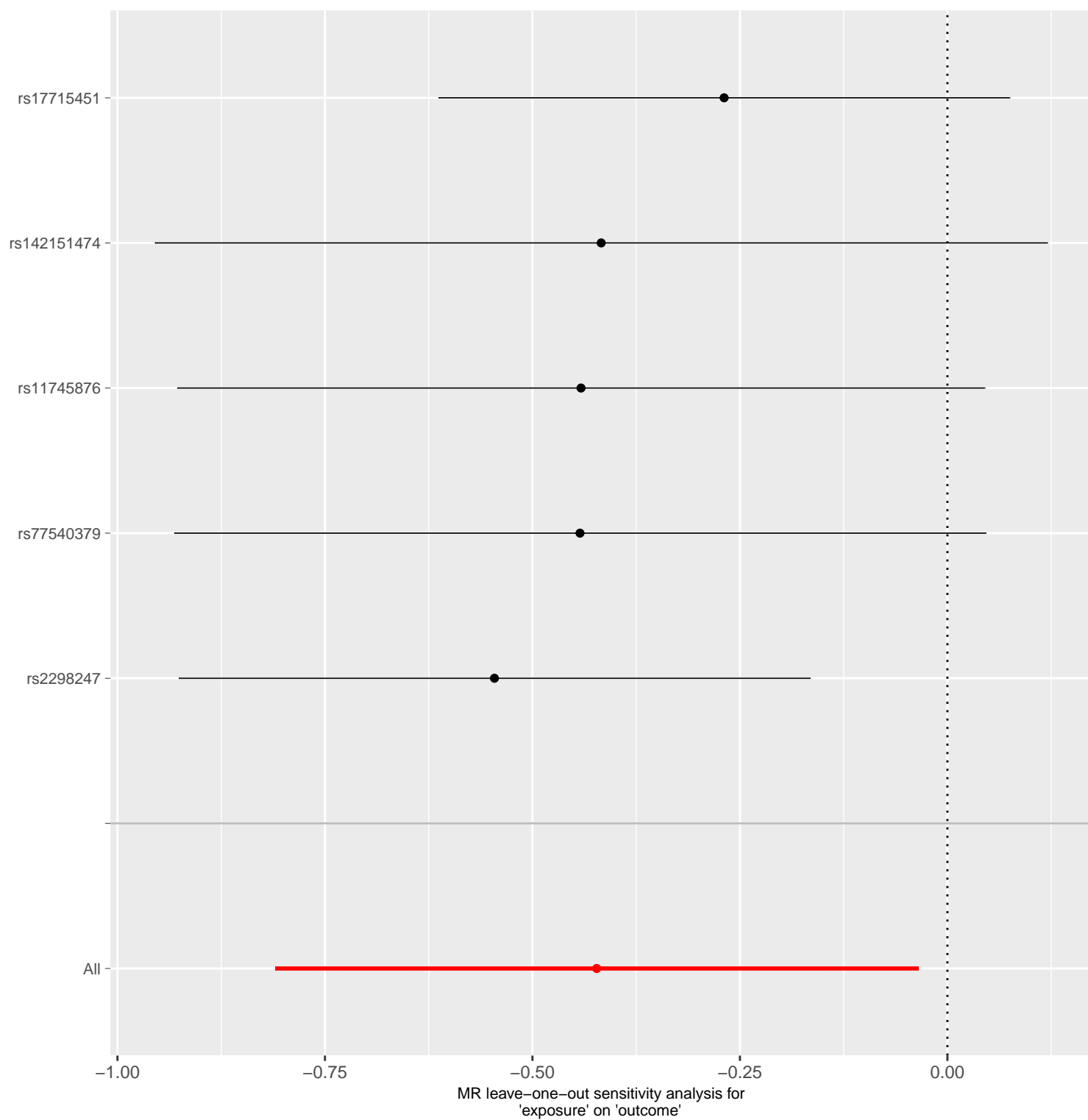

Supplement: Supplementary Data Sheet 3 — Full results of the pairwise Mendelian randomization analyses between ulcerative colitis-associated microbial taxa and ulcerative colitis-associated pyroptosis proteins, used for the downstream mediation analysis. [file DataSheet3.zip › GM_bd_fer_result/GCST90032583+17350_13_CHMP2B_CHM2B/sensitivity-analysis.pdf]

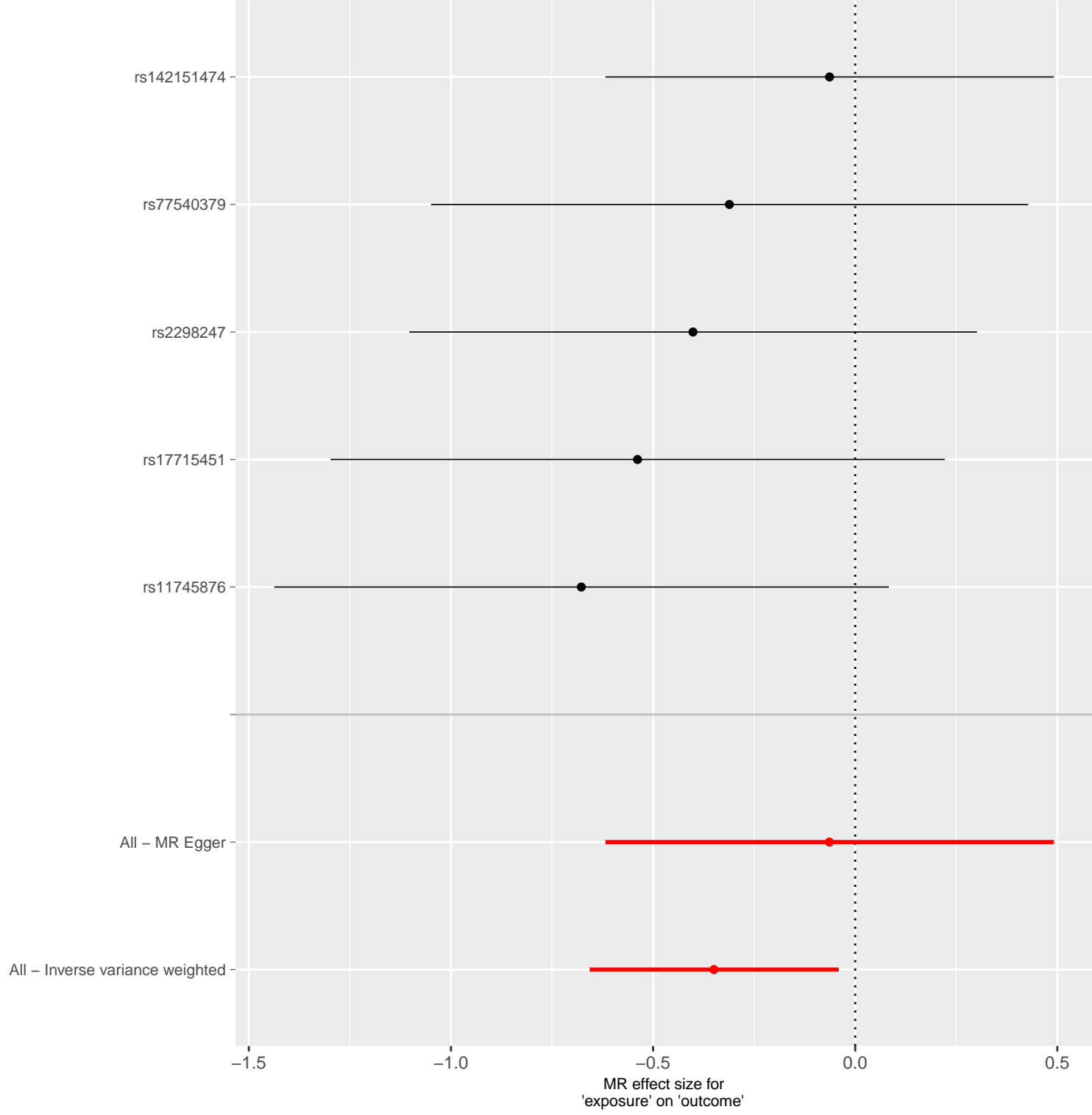

Supplement: Supplementary Data Sheet 3 — Full results of the pairwise Mendelian randomization analyses between ulcerative colitis-associated microbial taxa and ulcerative colitis-associated pyroptosis proteins, used for the downstream mediation analysis. [file DataSheet3.zip › GM_bd_fer_result/GCST90032583+17850_42_KLF4_KLF4/forest.pdf]

# MR Method

- Inverse variance weighted
- MR Egger

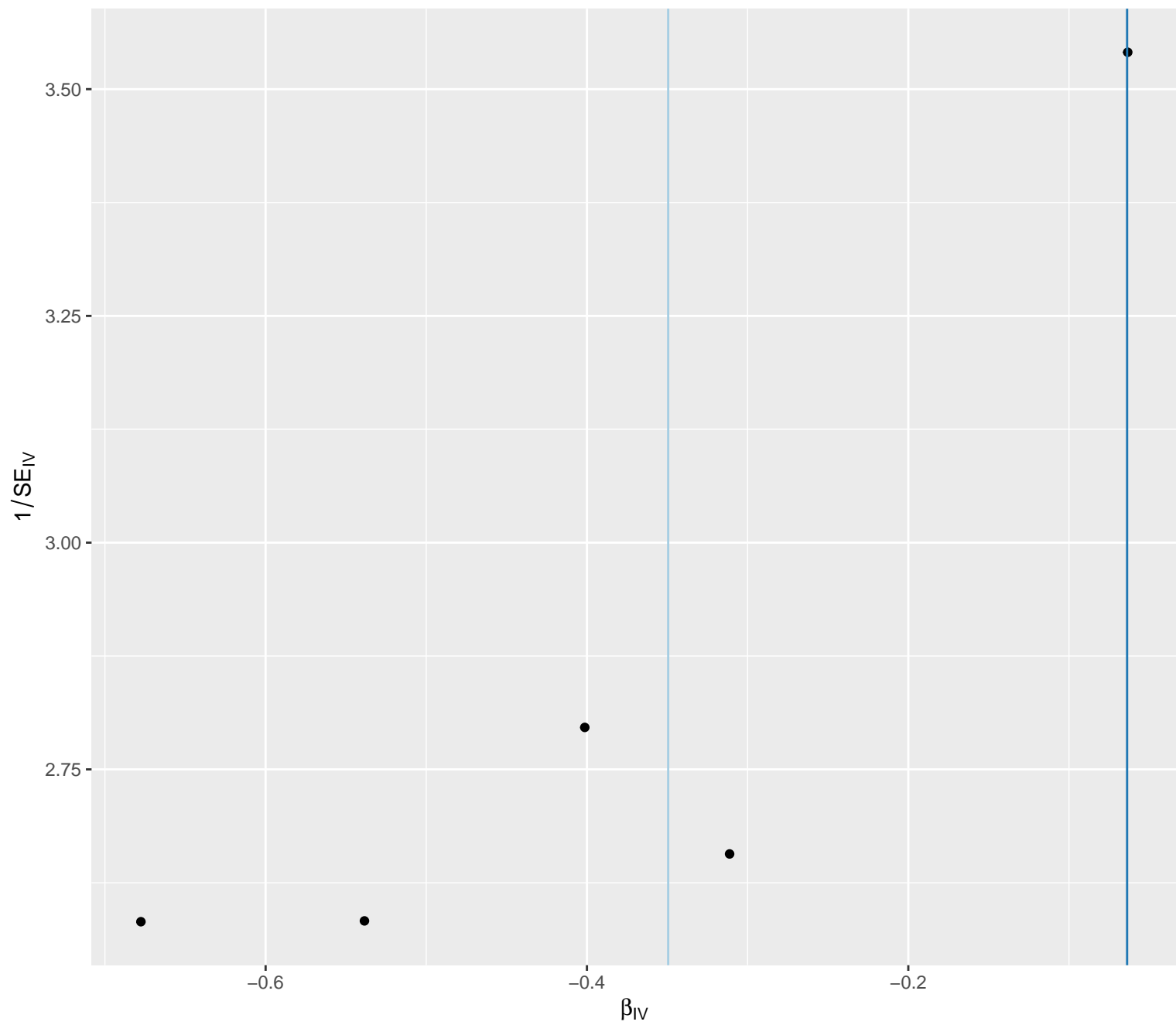

Supplement: Supplementary Data Sheet 3 — Full results of the pairwise Mendelian randomization analyses between ulcerative colitis-associated microbial taxa and ulcerative colitis-associated pyroptosis proteins, used for the downstream mediation analysis. [file DataSheet3.zip › GM_bd_fer_result/GCST90032583+17850_42_KLF4_KLF4/funnelplot.pdf]

# MR Test

- Inverse variance weighted
- MR Egger
- Simple mode
- Weighted median
- Weighted mode

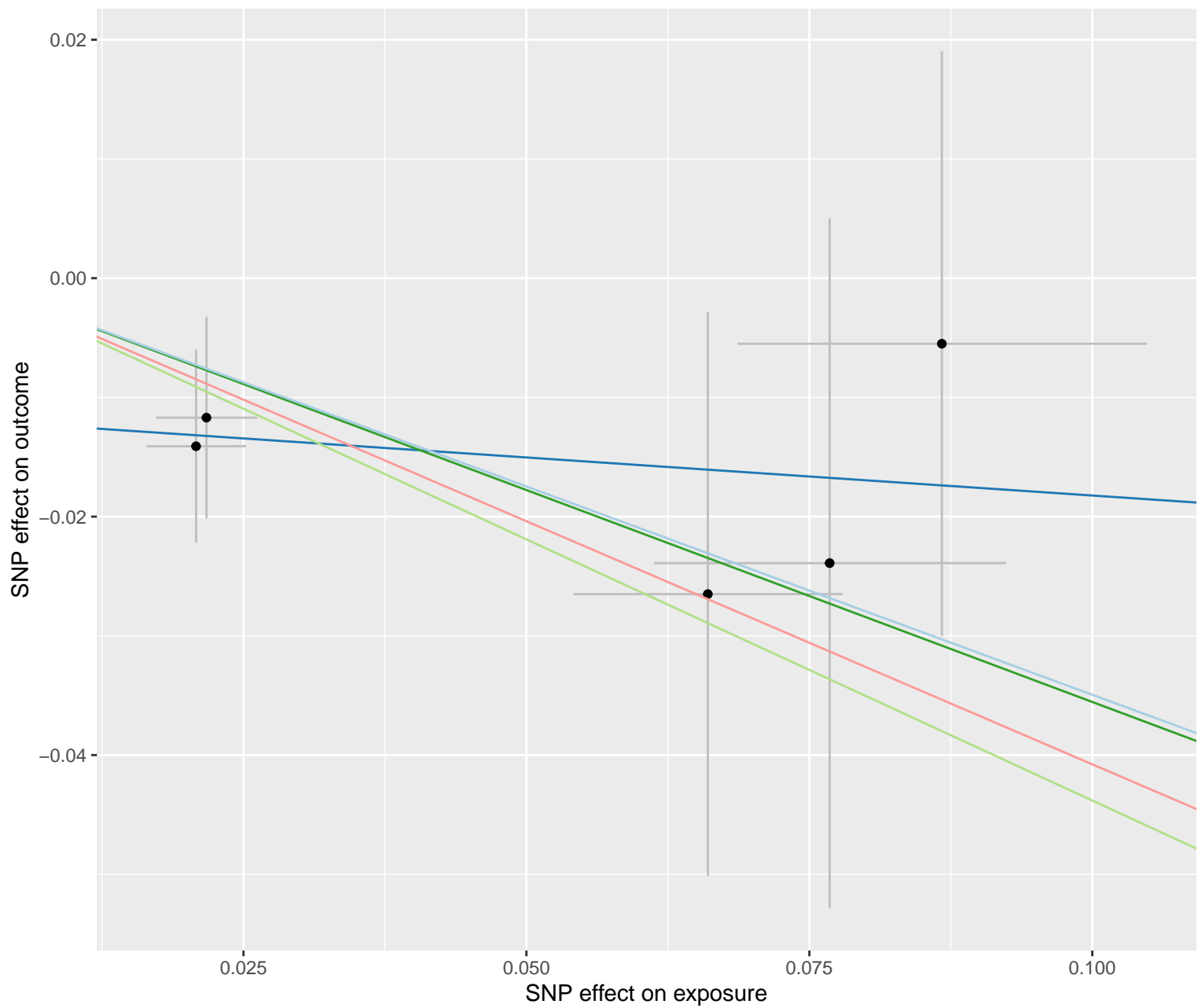

Supplement: Supplementary Data Sheet 3 — Full results of the pairwise Mendelian randomization analyses between ulcerative colitis-associated microbial taxa and ulcerative colitis-associated pyroptosis proteins, used for the downstream mediation analysis. [file DataSheet3.zip › GM_bd_fer_result/GCST90032583+17850_42_KLF4_KLF4/scatter.pdf]

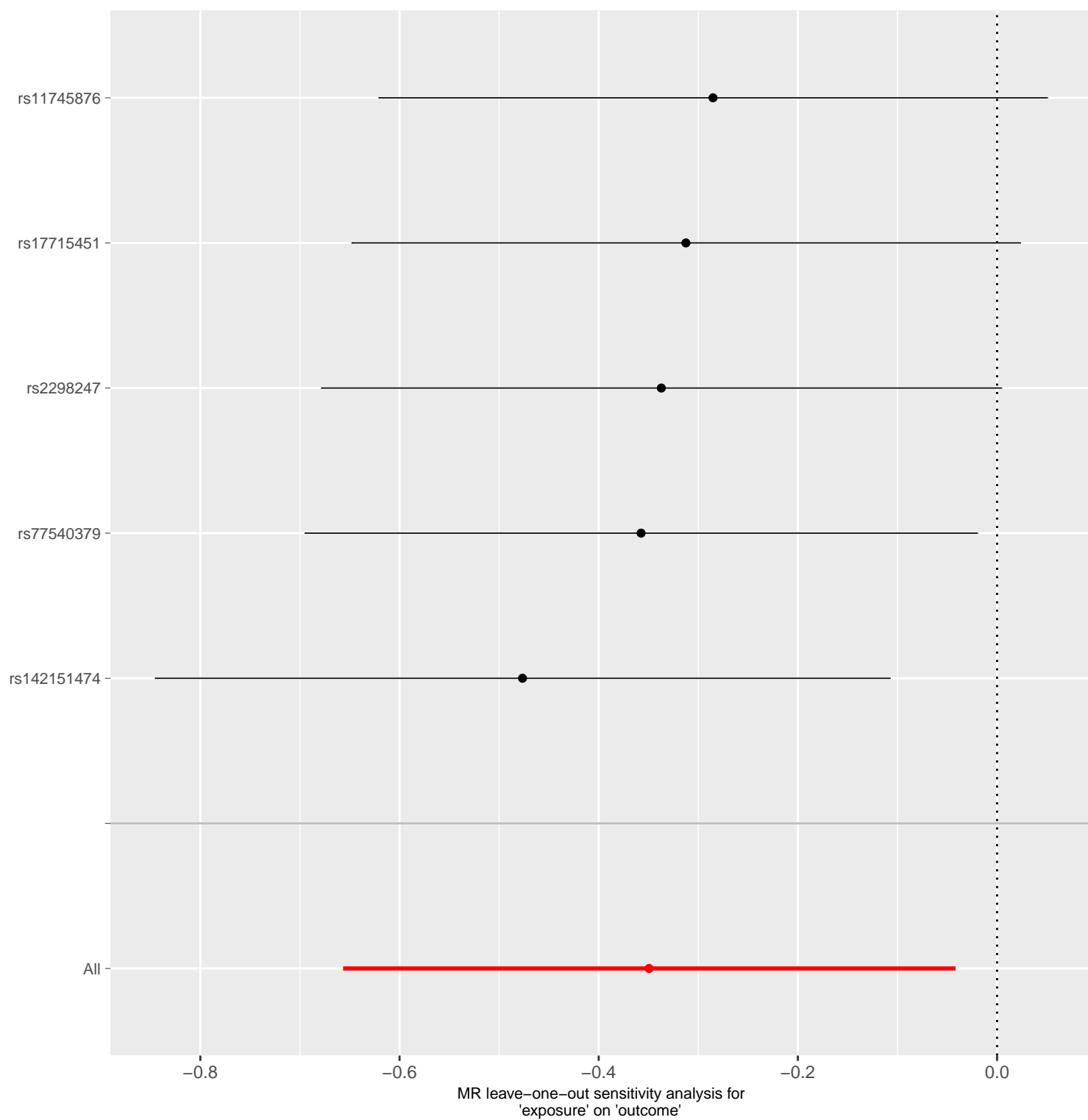

Supplement: Supplementary Data Sheet 3 — Full results of the pairwise Mendelian randomization analyses between ulcerative colitis-associated microbial taxa and ulcerative colitis-associated pyroptosis proteins, used for the downstream mediation analysis. [file DataSheet3.zip › GM_bd_fer_result/GCST90032583+17850_42_KLF4_KLF4/sensitivity-analysis.pdf]

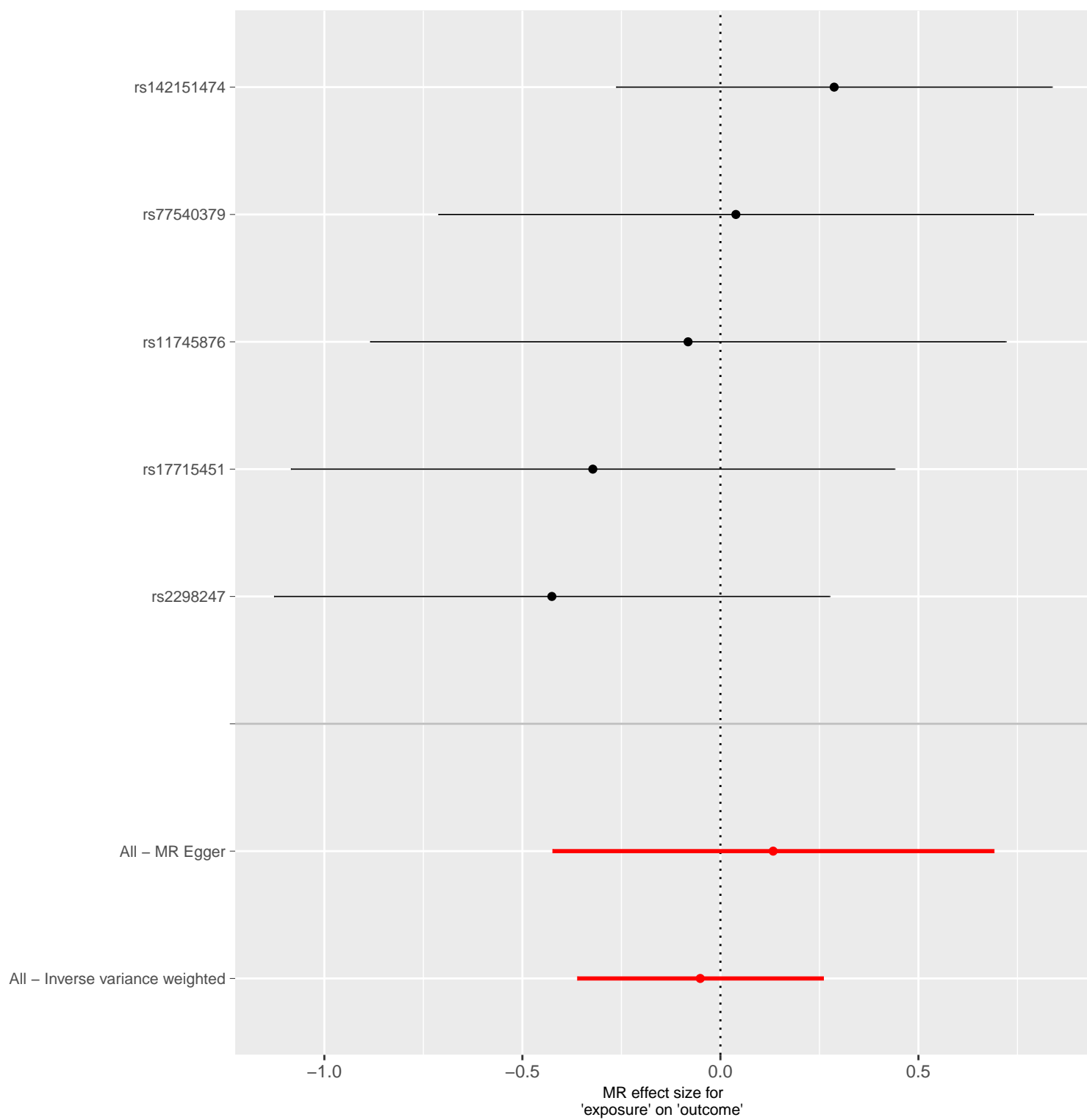

Supplement: Supplementary Data Sheet 3 — Full results of the pairwise Mendelian randomization analyses between ulcerative colitis-associated microbial taxa and ulcerative colitis-associated pyroptosis proteins, used for the downstream mediation analysis. [file DataSheet3.zip › GM_bd_fer_result/GCST90032583+18819_21_PPIC_PPIC/forest.pdf]

# MR Method

- Inverse variance weighted
- MR Egger

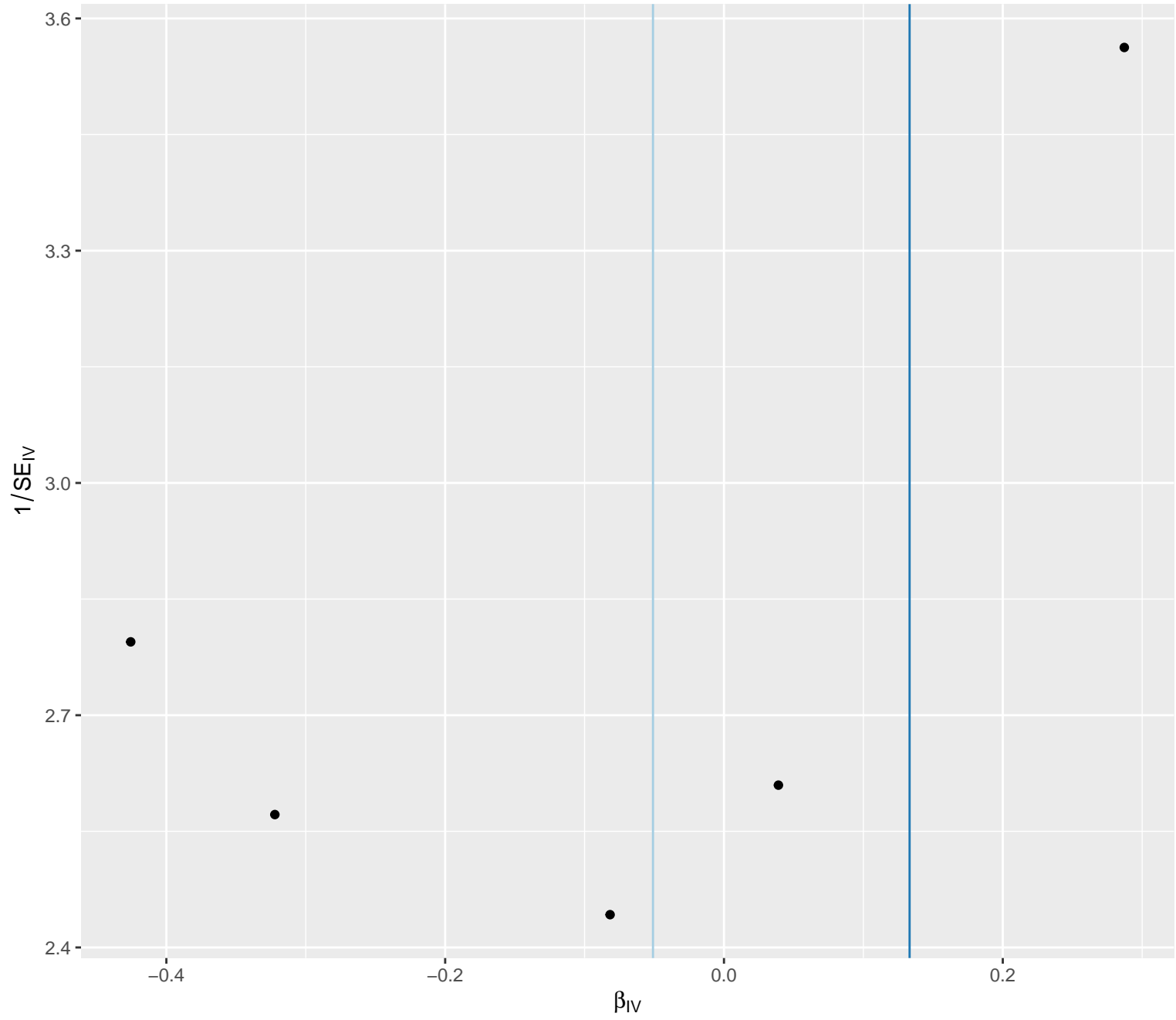

Supplement: Supplementary Data Sheet 3 — Full results of the pairwise Mendelian randomization analyses between ulcerative colitis-associated microbial taxa and ulcerative colitis-associated pyroptosis proteins, used for the downstream mediation analysis. [file DataSheet3.zip › GM_bd_fer_result/GCST90032583+18819_21_PPIC_PPIC/funnelplot.pdf]

# MR Test

- Inverse variance weighted
- MR Egger
- Simple mode
- Weighted median
- Weighted mode

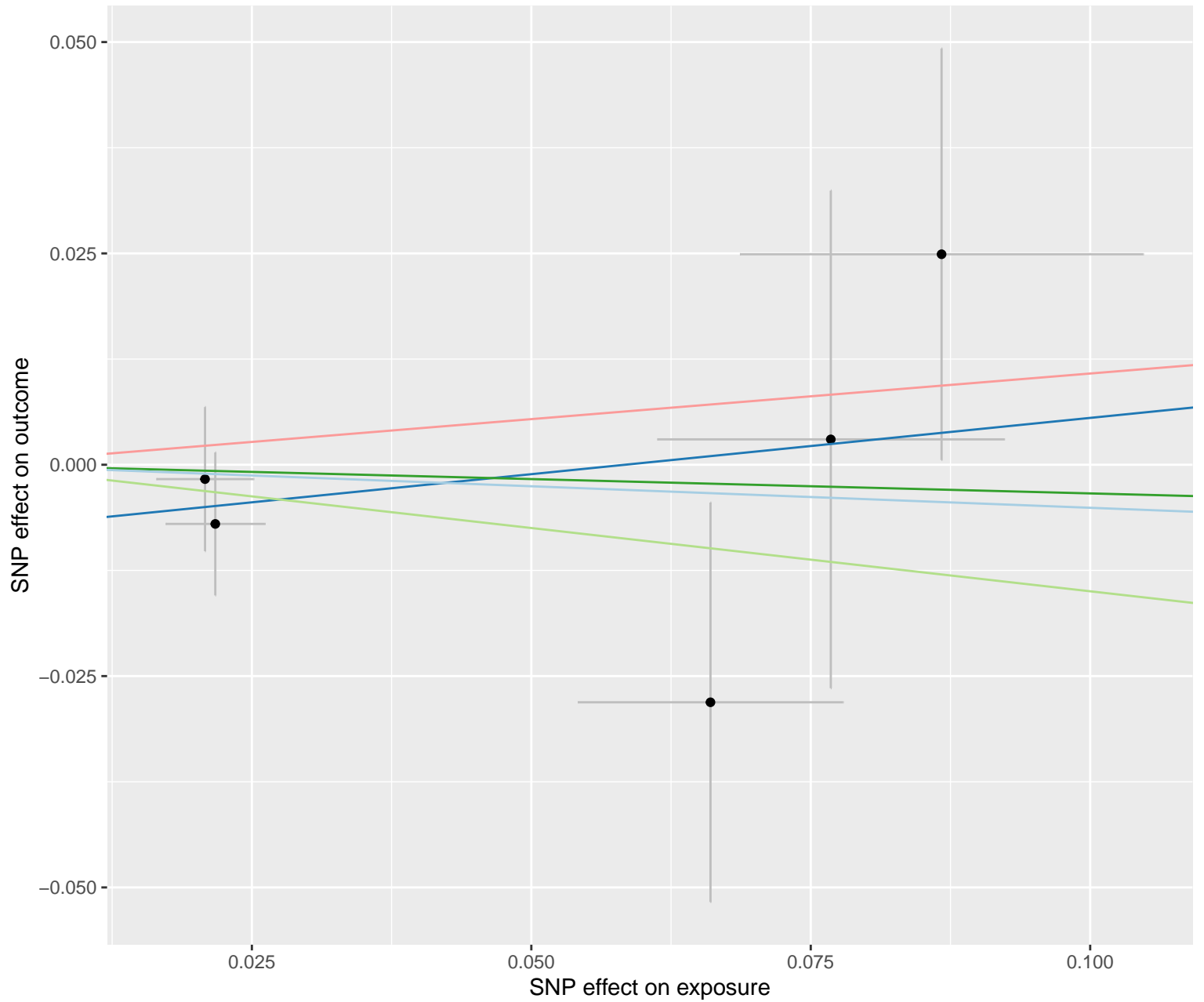

Supplement: Supplementary Data Sheet 3 — Full results of the pairwise Mendelian randomization analyses between ulcerative colitis-associated microbial taxa and ulcerative colitis-associated pyroptosis proteins, used for the downstream mediation analysis. [file DataSheet3.zip › GM_bd_fer_result/GCST90032583+18819_21_PPIC_PPIC/scatter.pdf]

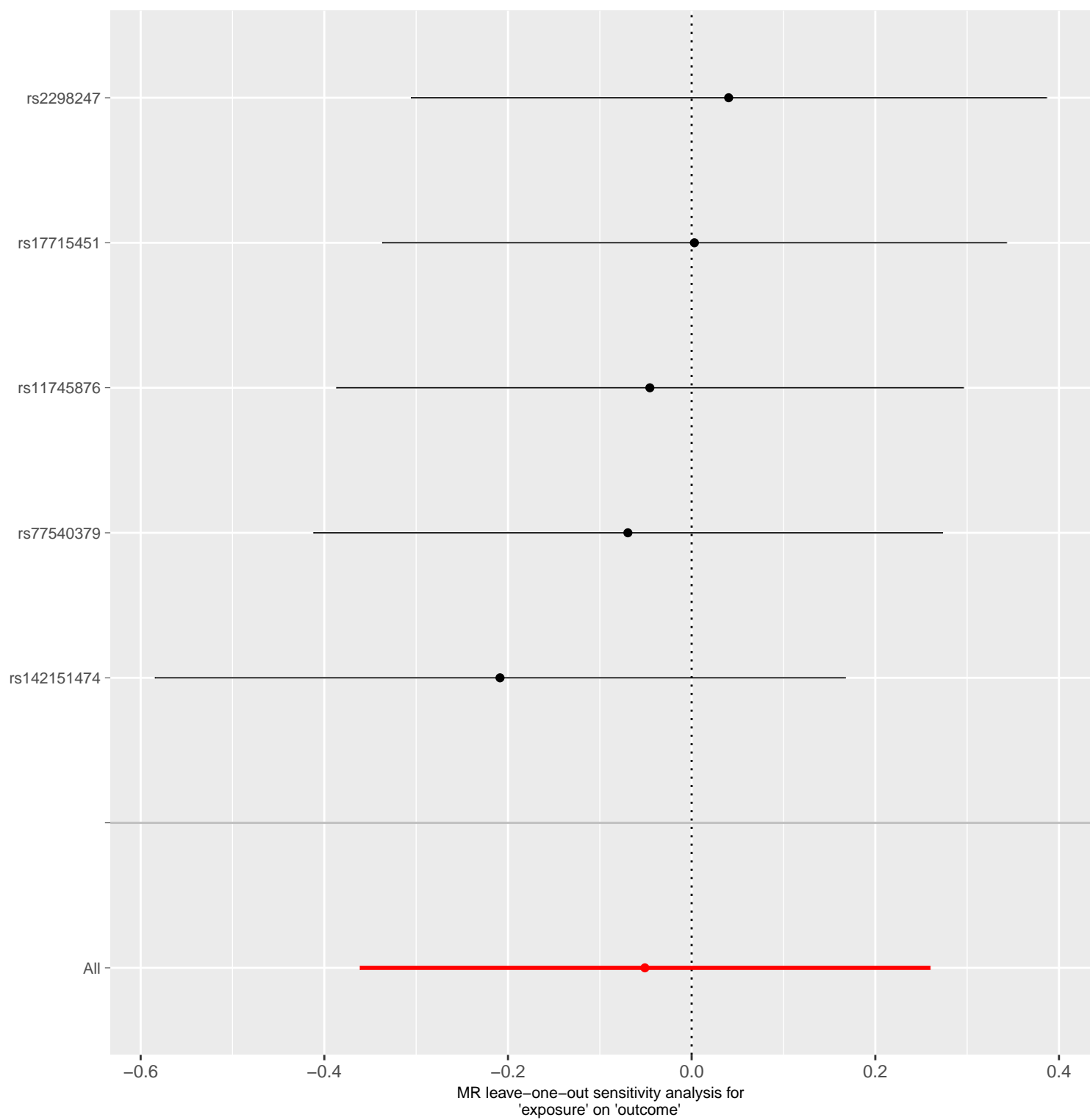

Supplement: Supplementary Data Sheet 3 — Full results of the pairwise Mendelian randomization analyses between ulcerative colitis-associated microbial taxa and ulcerative colitis-associated pyroptosis proteins, used for the downstream mediation analysis. [file DataSheet3.zip › GM_bd_fer_result/GCST90032583+18819_21_PPIC_PPIC/sensitivity-analysis.pdf]

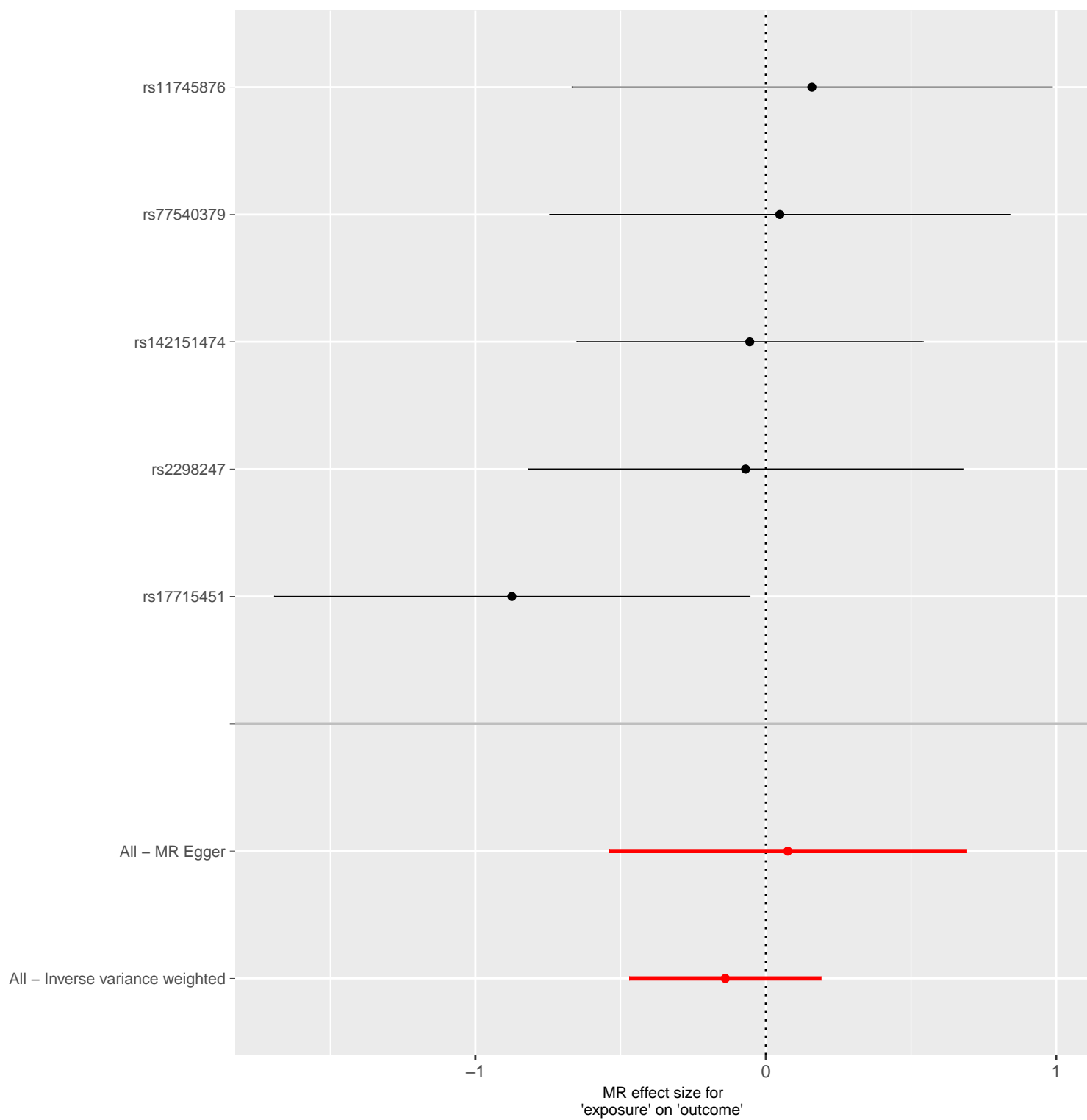

Supplement: Supplementary Data Sheet 3 — Full results of the pairwise Mendelian randomization analyses between ulcerative colitis-associated microbial taxa and ulcerative colitis-associated pyroptosis proteins, used for the downstream mediation analysis. [file DataSheet3.zip › GM_bd_fer_result/GCST90032583+19437_61_VEGFA_L_VEGF165/forest.pdf]

# MR Method

- Inverse variance weighted
- MR Egger

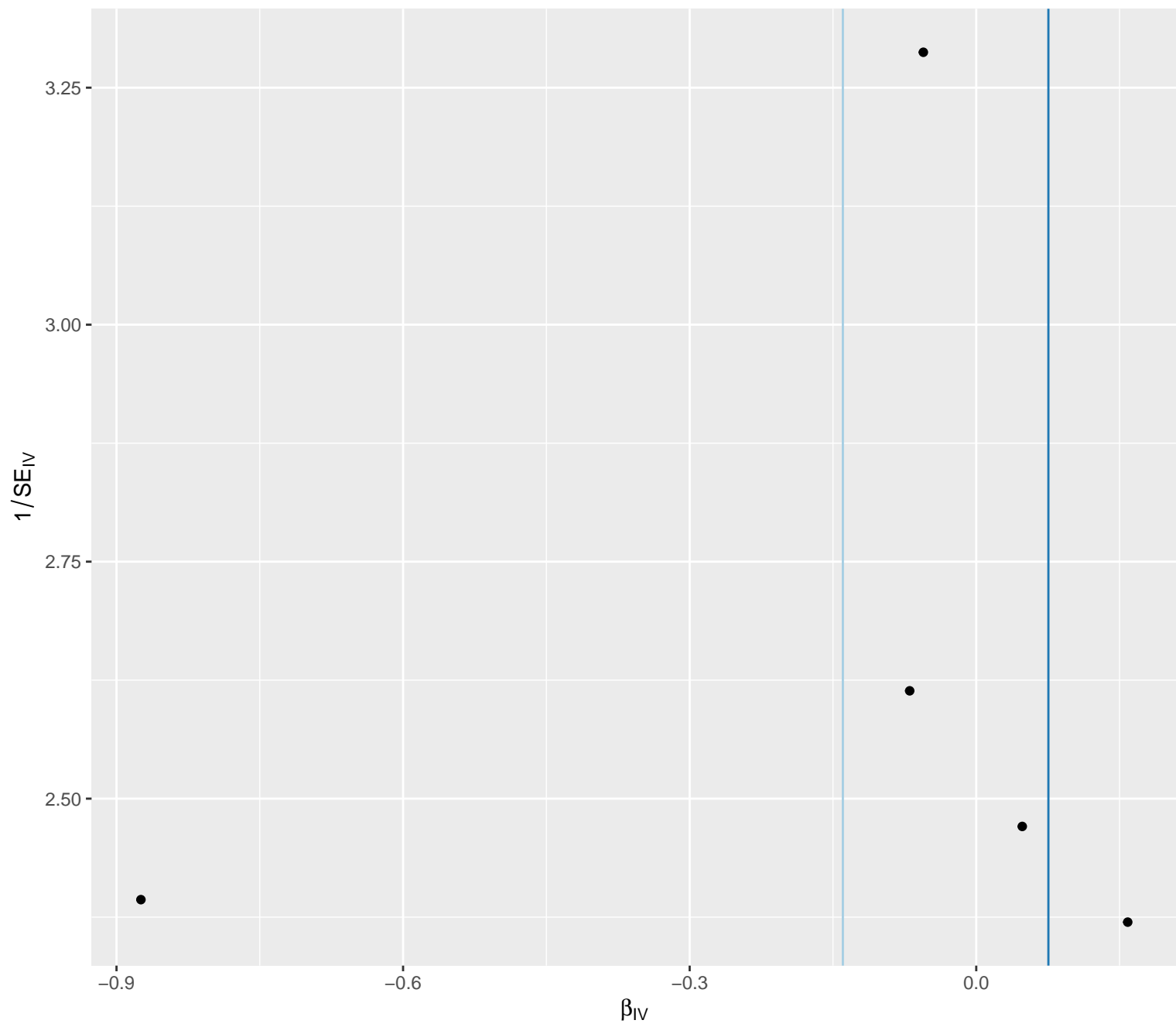

Supplement: Supplementary Data Sheet 3 — Full results of the pairwise Mendelian randomization analyses between ulcerative colitis-associated microbial taxa and ulcerative colitis-associated pyroptosis proteins, used for the downstream mediation analysis. [file DataSheet3.zip › GM_bd_fer_result/GCST90032583+19437_61_VEGFA_L_VEGF165/funnelplot.pdf]

# MR Test

- Inverse variance weighted
- MR Egger
- Simple mode
- Weighted median
- Weighted mode

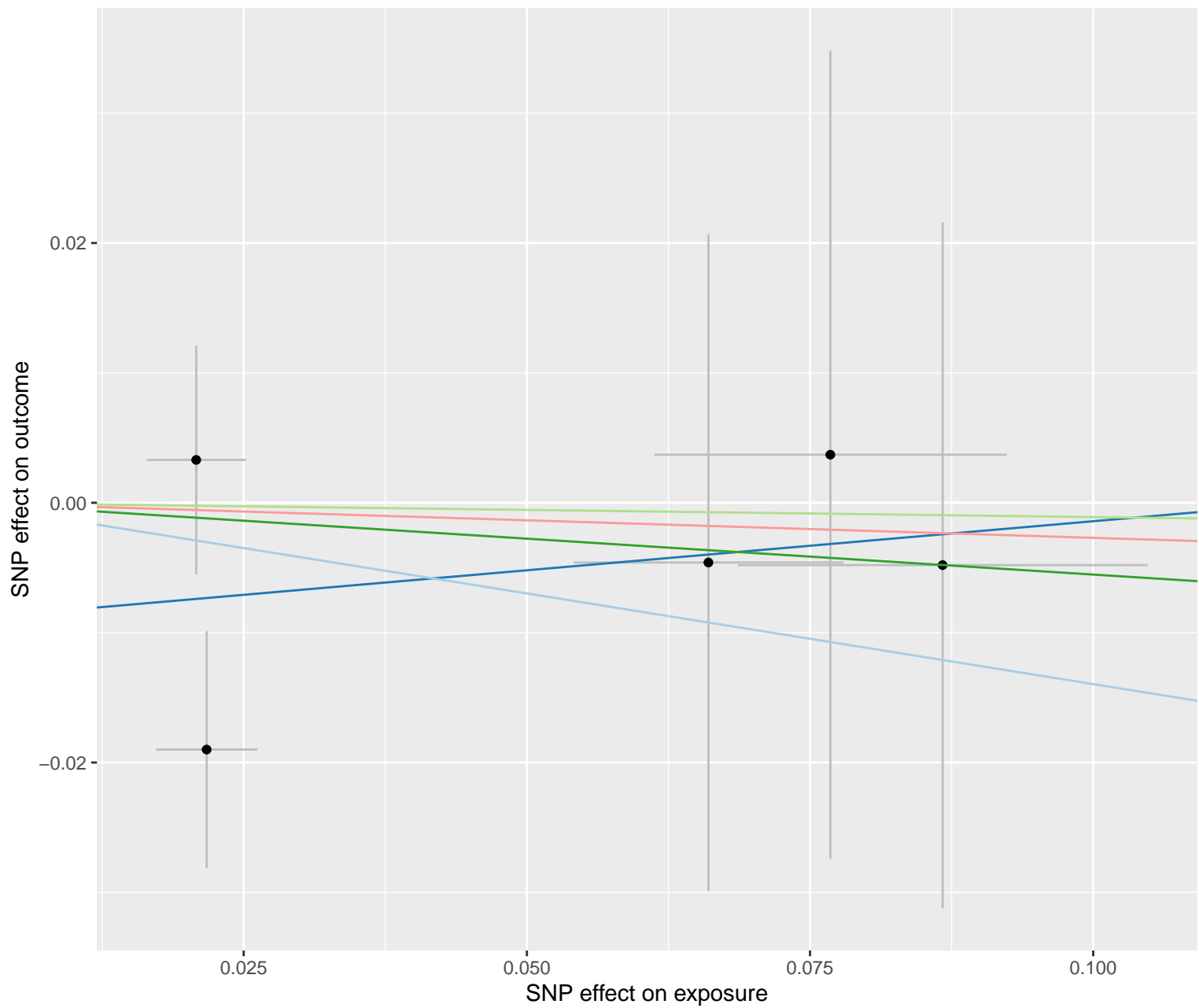

Supplement: Supplementary Data Sheet 3 — Full results of the pairwise Mendelian randomization analyses between ulcerative colitis-associated microbial taxa and ulcerative colitis-associated pyroptosis proteins, used for the downstream mediation analysis. [file DataSheet3.zip › GM_bd_fer_result/GCST90032583+19437_61_VEGFA_L_VEGF165/scatter.pdf]

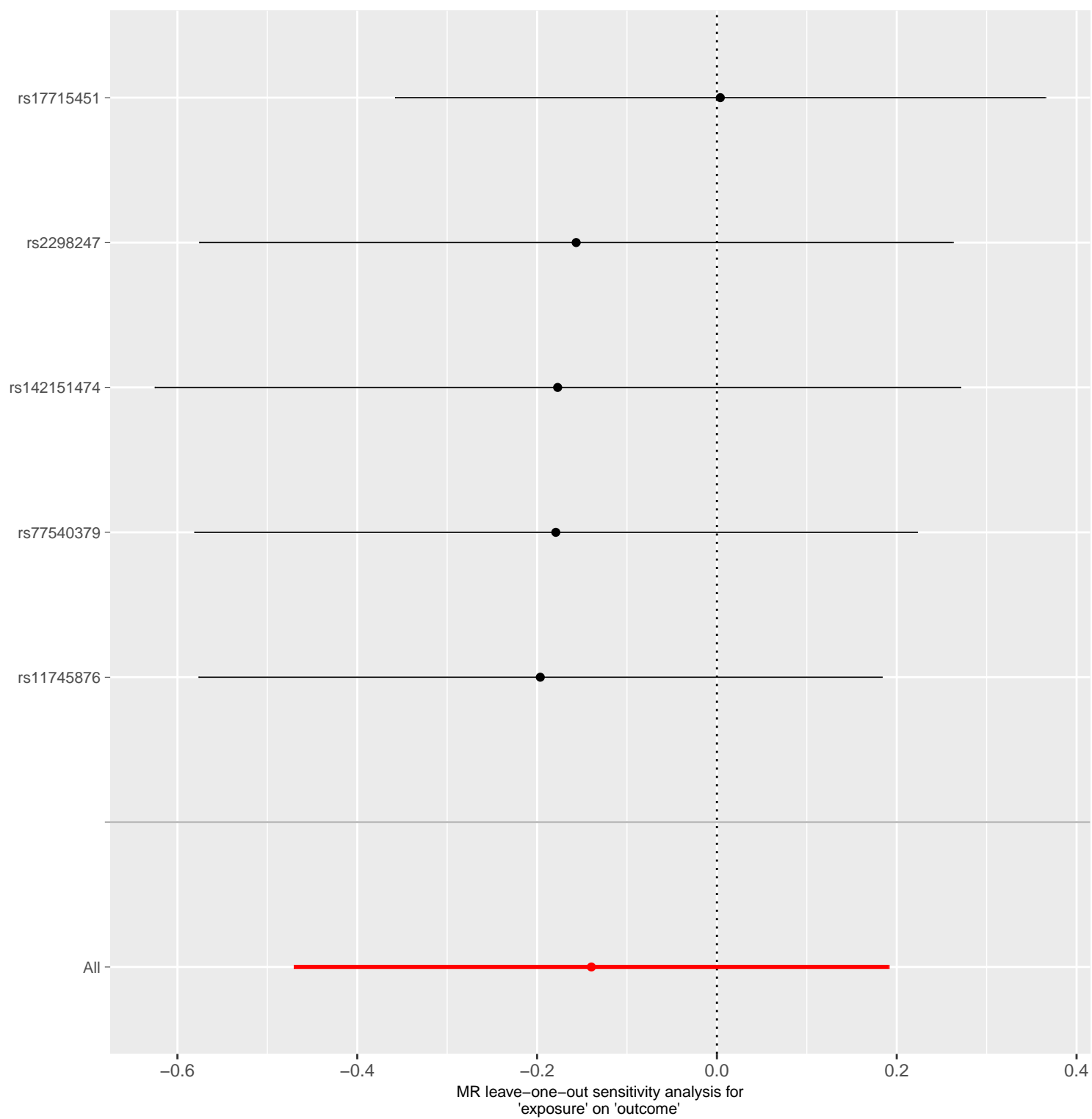

Supplement: Supplementary Data Sheet 3 — Full results of the pairwise Mendelian randomization analyses between ulcerative colitis-associated microbial taxa and ulcerative colitis-associated pyroptosis proteins, used for the downstream mediation analysis. [file DataSheet3.zip › GM_bd_fer_result/GCST90032583+19437_61_VEGFA_L_VEGF165/sensitivity-analysis.pdf]

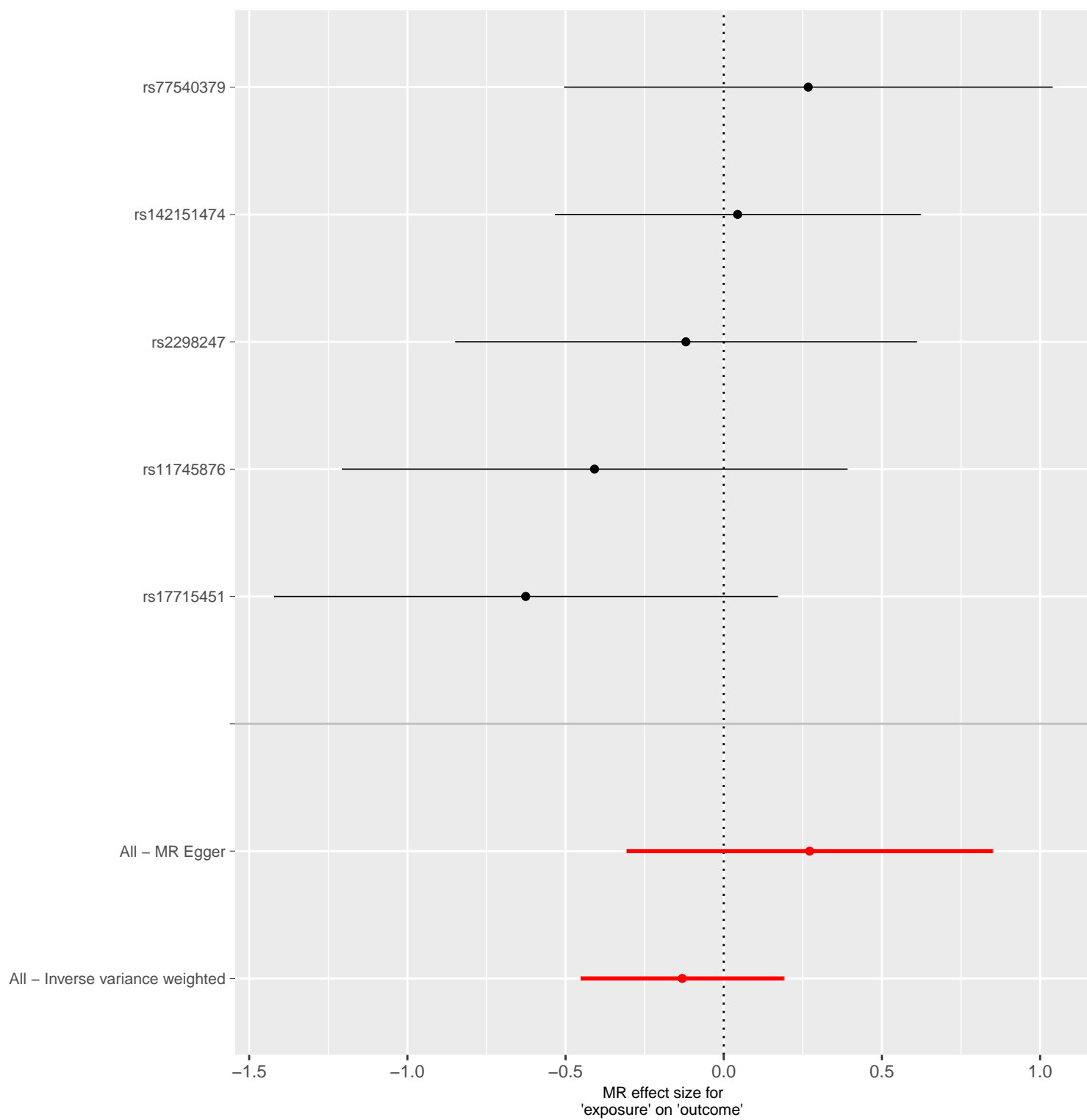

Supplement: Supplementary Data Sheet 3 — Full results of the pairwise Mendelian randomization analyses between ulcerative colitis-associated microbial taxa and ulcerative colitis-associated pyroptosis proteins, used for the downstream mediation analysis. [file DataSheet3.zip › GM_bd_fer_result/GCST90032583+2597_8_VEGFA_VEGF/forest.pdf]

# MR Method

- Inverse variance weighted
- MR Egger

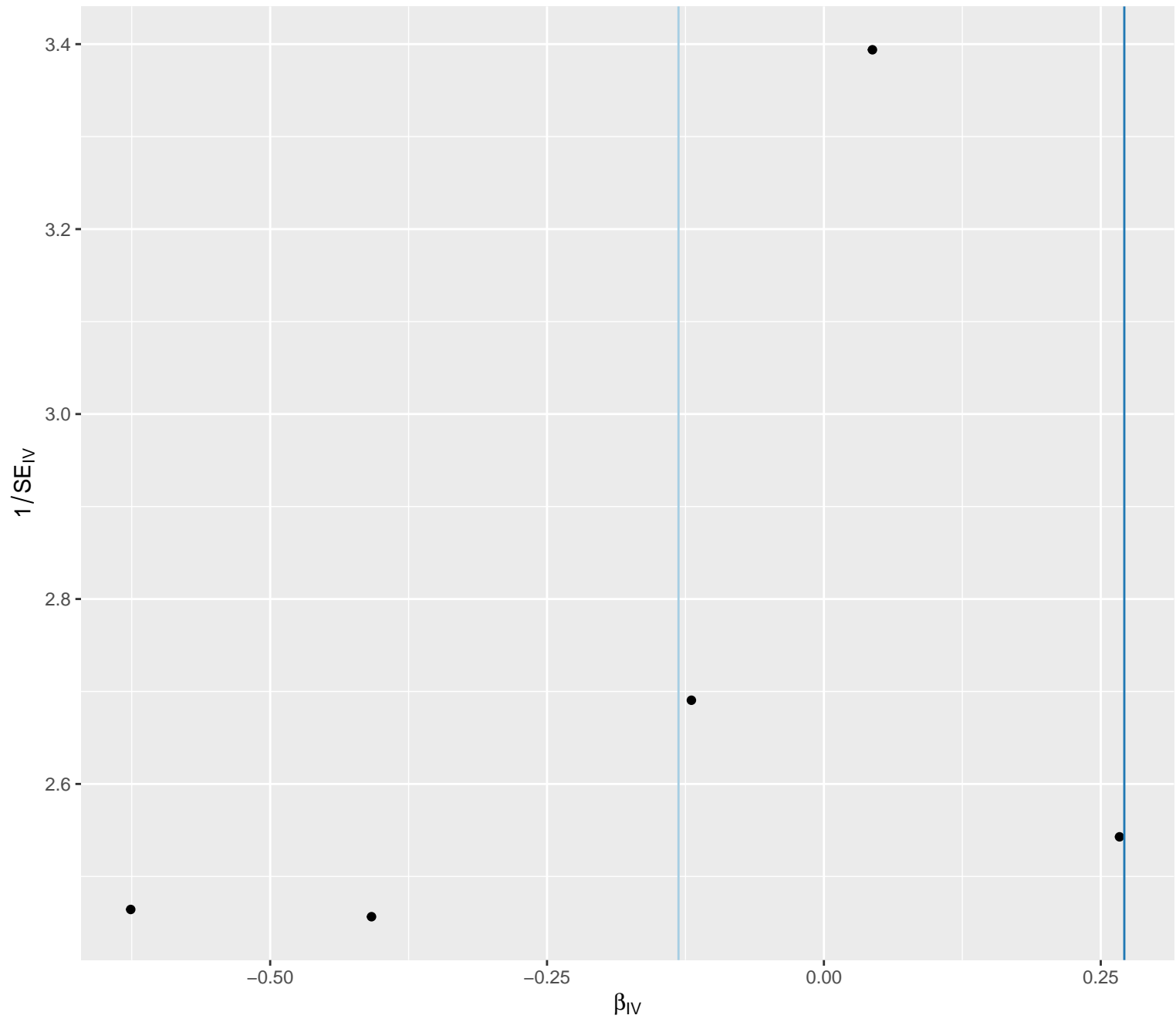

Supplement: Supplementary Data Sheet 3 — Full results of the pairwise Mendelian randomization analyses between ulcerative colitis-associated microbial taxa and ulcerative colitis-associated pyroptosis proteins, used for the downstream mediation analysis. [file DataSheet3.zip › GM_bd_fer_result/GCST90032583+2597_8_VEGFA_VEGF/funnelplot.pdf]

# MR Test

- Inverse variance weighted
- MR Egger
- Simple mode
- Weighted median
- Weighted mode

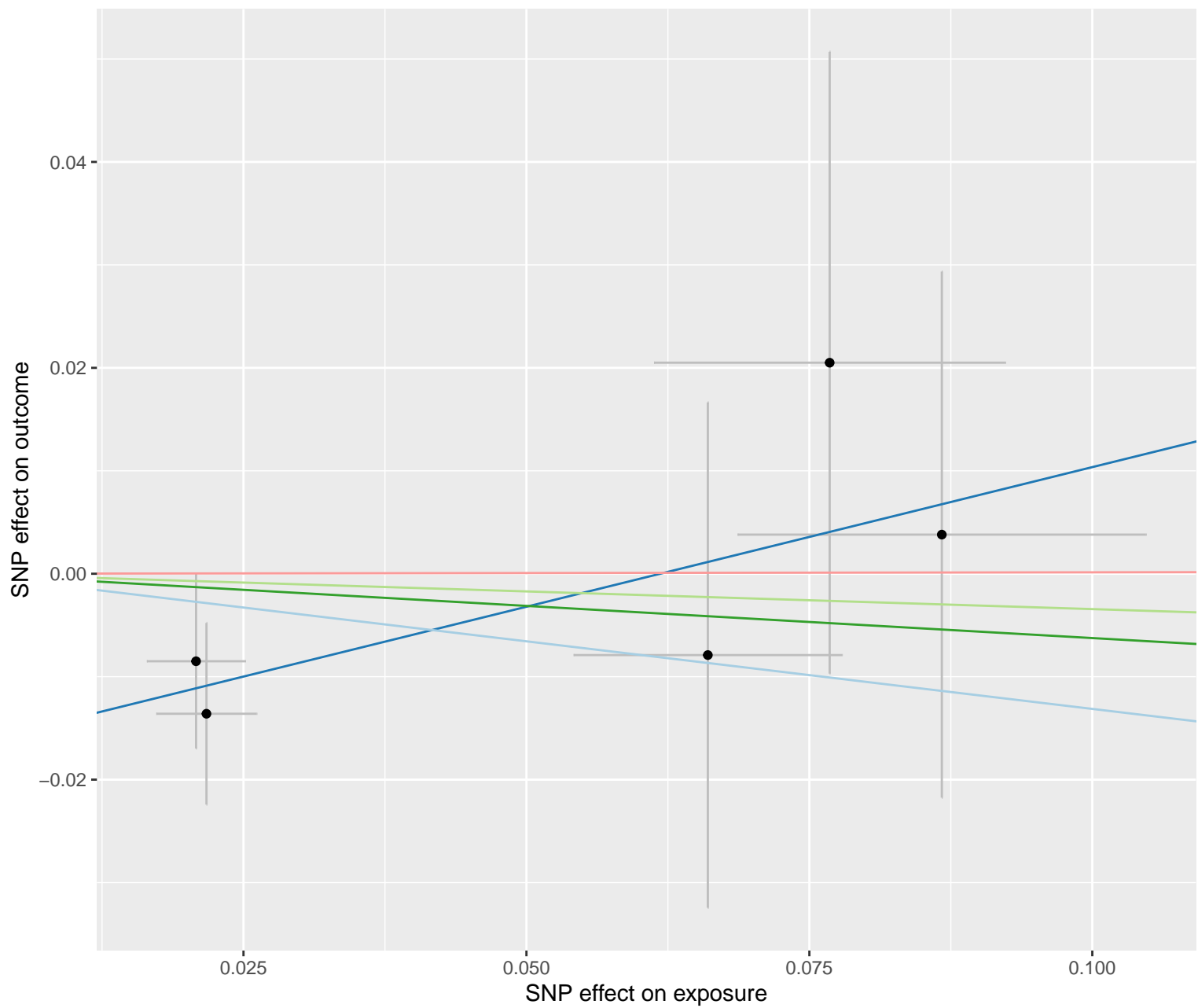

Supplement: Supplementary Data Sheet 3 — Full results of the pairwise Mendelian randomization analyses between ulcerative colitis-associated microbial taxa and ulcerative colitis-associated pyroptosis proteins, used for the downstream mediation analysis. [file DataSheet3.zip › GM_bd_fer_result/GCST90032583+2597_8_VEGFA_VEGF/scatter.pdf]

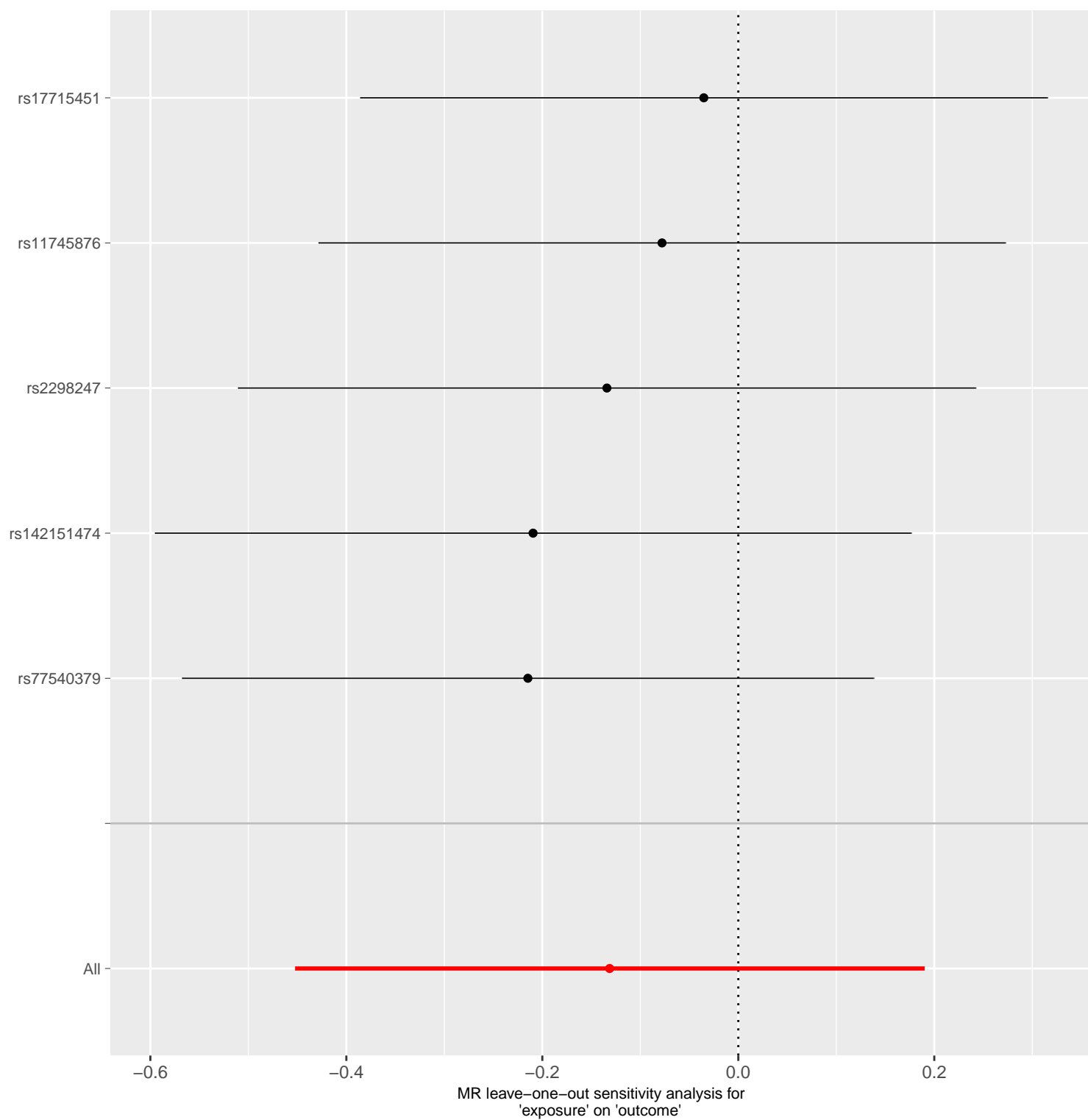

Supplement: Supplementary Data Sheet 3 — Full results of the pairwise Mendelian randomization analyses between ulcerative colitis-associated microbial taxa and ulcerative colitis-associated pyroptosis proteins, used for the downstream mediation analysis. [file DataSheet3.zip › GM_bd_fer_result/GCST90032583+2597_8_VEGFA_VEGF/sensitivity-analysis.pdf]

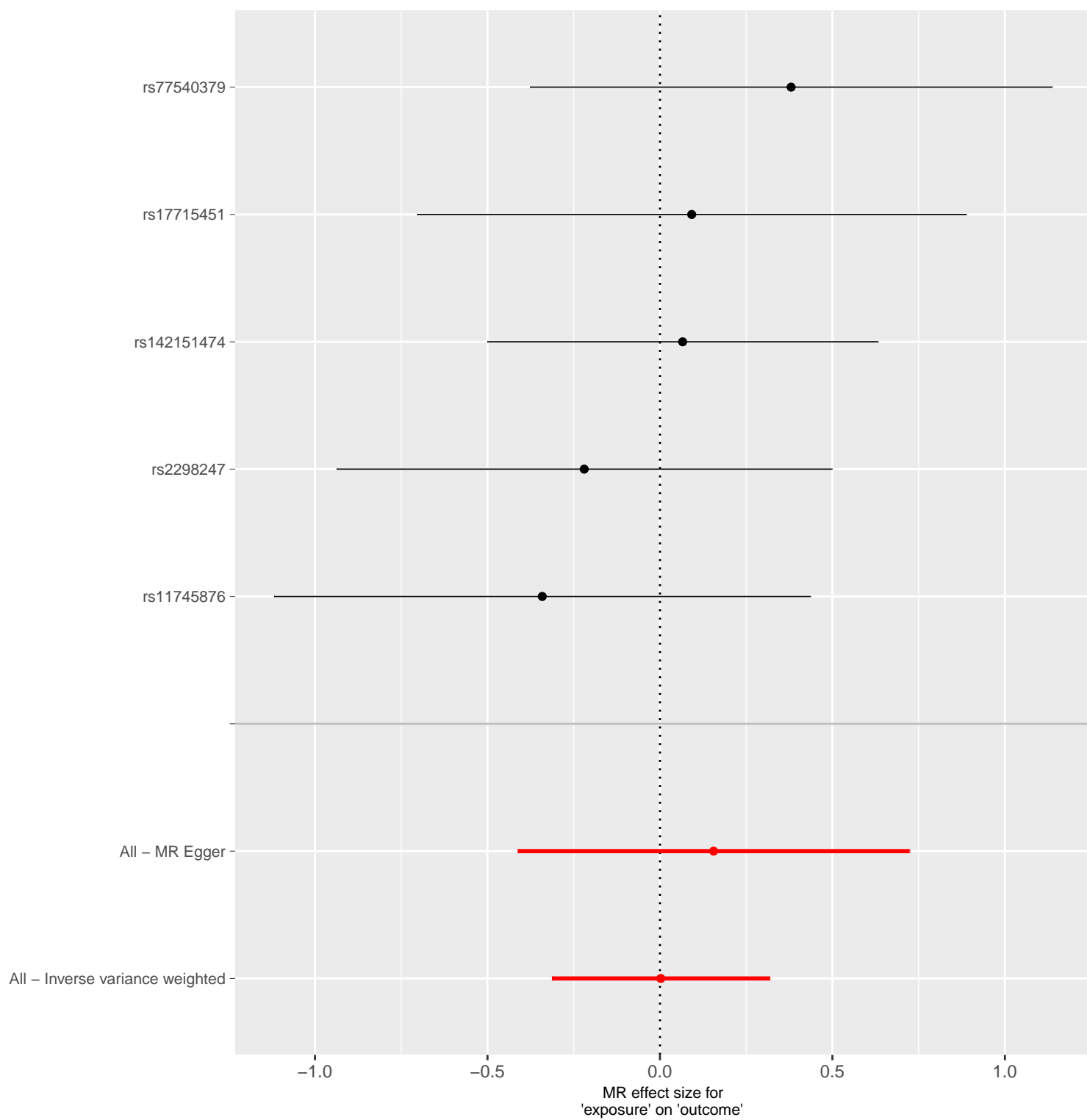

Supplement: Supplementary Data Sheet 3 — Full results of the pairwise Mendelian randomization analyses between ulcerative colitis-associated microbial taxa and ulcerative colitis-associated pyroptosis proteins, used for the downstream mediation analysis. [file DataSheet3.zip › GM_bd_fer_result/GCST90032583+2693_20_OSM_OSM/forest.pdf]

# MR Method

- Inverse variance weighted
- MR Egger

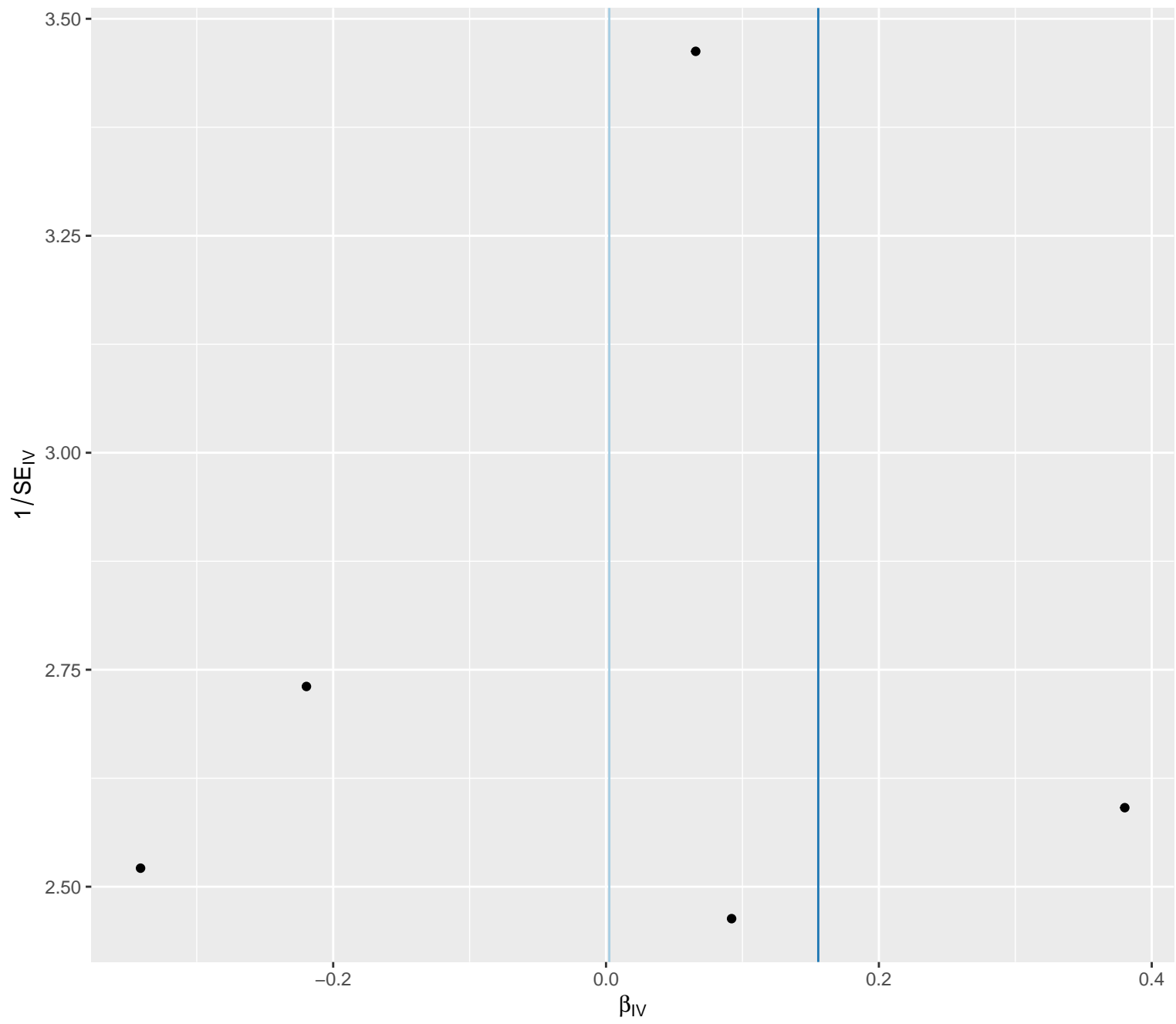

Supplement: Supplementary Data Sheet 3 — Full results of the pairwise Mendelian randomization analyses between ulcerative colitis-associated microbial taxa and ulcerative colitis-associated pyroptosis proteins, used for the downstream mediation analysis. [file DataSheet3.zip › GM_bd_fer_result/GCST90032583+2693_20_OSM_OSM/funnelplot.pdf]

# MR Test

- Inverse variance weighted
- MR Egger
- Simple mode
- Weighted median
- Weighted mode

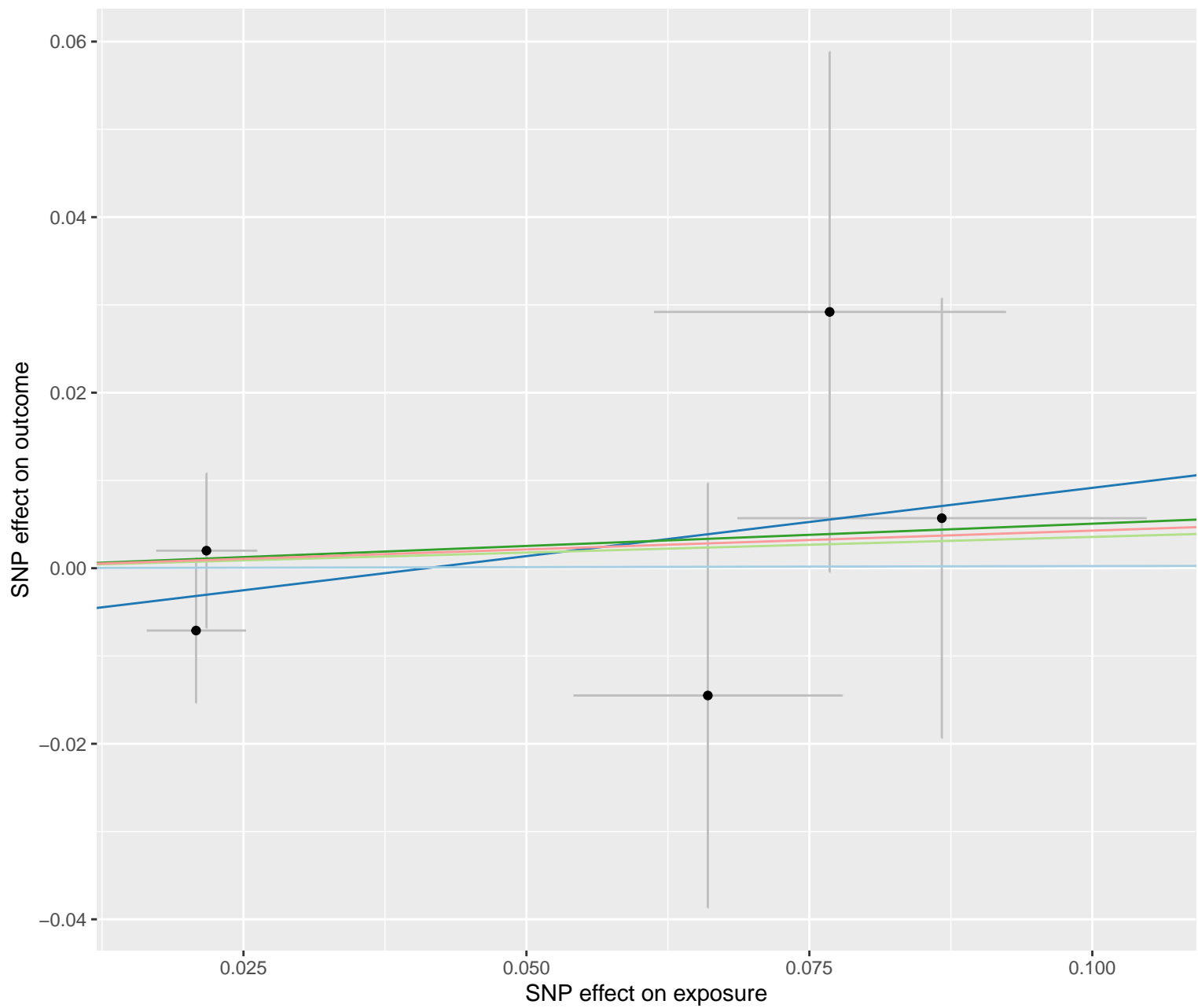

Supplement: Supplementary Data Sheet 3 — Full results of the pairwise Mendelian randomization analyses between ulcerative colitis-associated microbial taxa and ulcerative colitis-associated pyroptosis proteins, used for the downstream mediation analysis. [file DataSheet3.zip › GM_bd_fer_result/GCST90032583+2693_20_OSM_OSM/scatter.pdf]

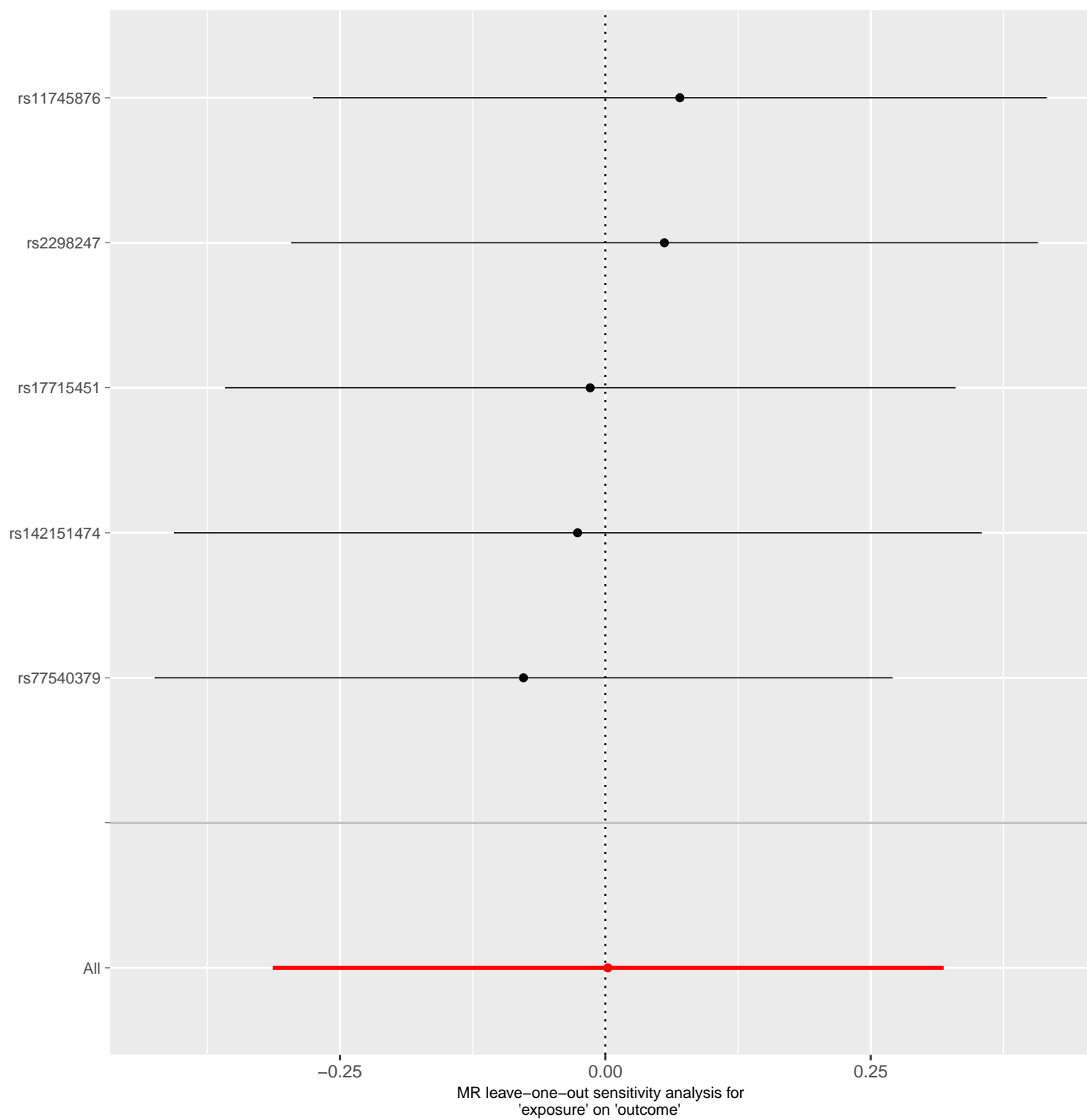

Supplement: Supplementary Data Sheet 3 — Full results of the pairwise Mendelian randomization analyses between ulcerative colitis-associated microbial taxa and ulcerative colitis-associated pyroptosis proteins, used for the downstream mediation analysis. [file DataSheet3.zip › GM_bd_fer_result/GCST90032583+2693_20_OSM_OSM/sensitivity-analysis.pdf]

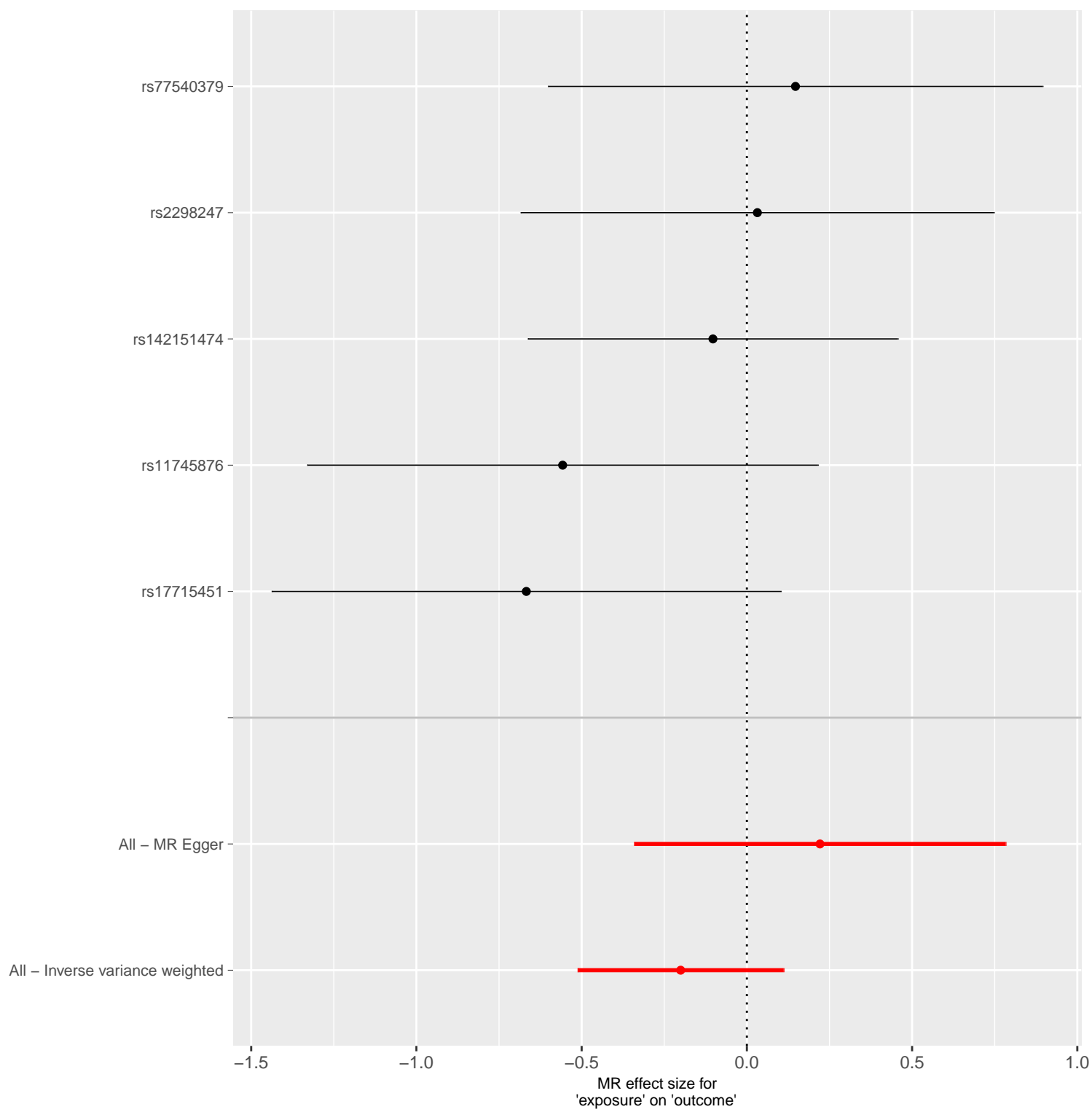

Supplement: Supplementary Data Sheet 3 — Full results of the pairwise Mendelian randomization analyses between ulcerative colitis-associated microbial taxa and ulcerative colitis-associated pyroptosis proteins, used for the downstream mediation analysis. [file DataSheet3.zip › GM_bd_fer_result/GCST90032583+3072_4_IL13_IL_13/forest.pdf]

# MR Method

- Inverse variance weighted
- MR Egger

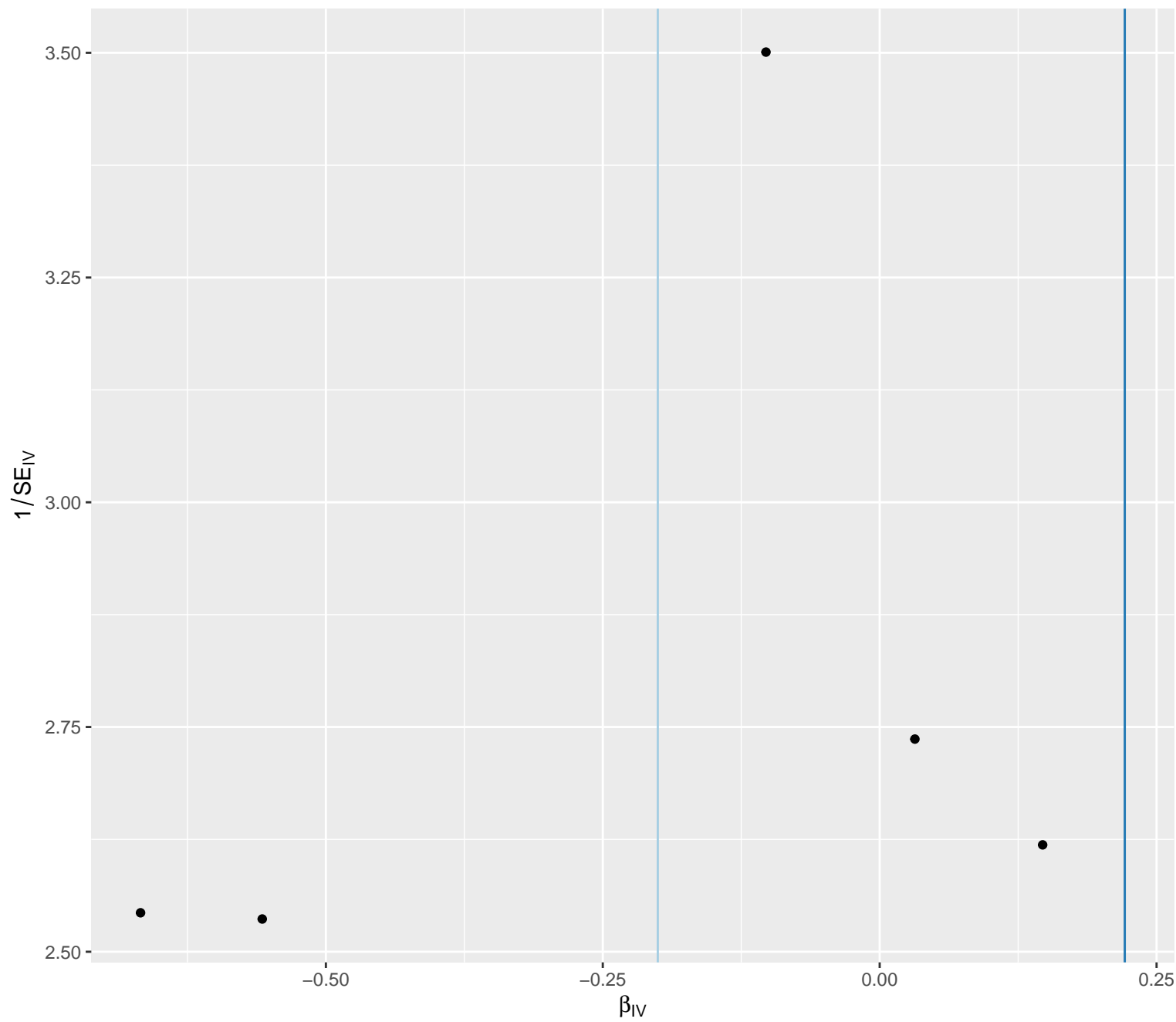

Supplement: Supplementary Data Sheet 3 — Full results of the pairwise Mendelian randomization analyses between ulcerative colitis-associated microbial taxa and ulcerative colitis-associated pyroptosis proteins, used for the downstream mediation analysis. [file DataSheet3.zip › GM_bd_fer_result/GCST90032583+3072_4_IL13_IL_13/funnelplot.pdf]

# MR Test

- Inverse variance weighted
- MR Egger
- Simple mode
- Weighted median
- Weighted mode

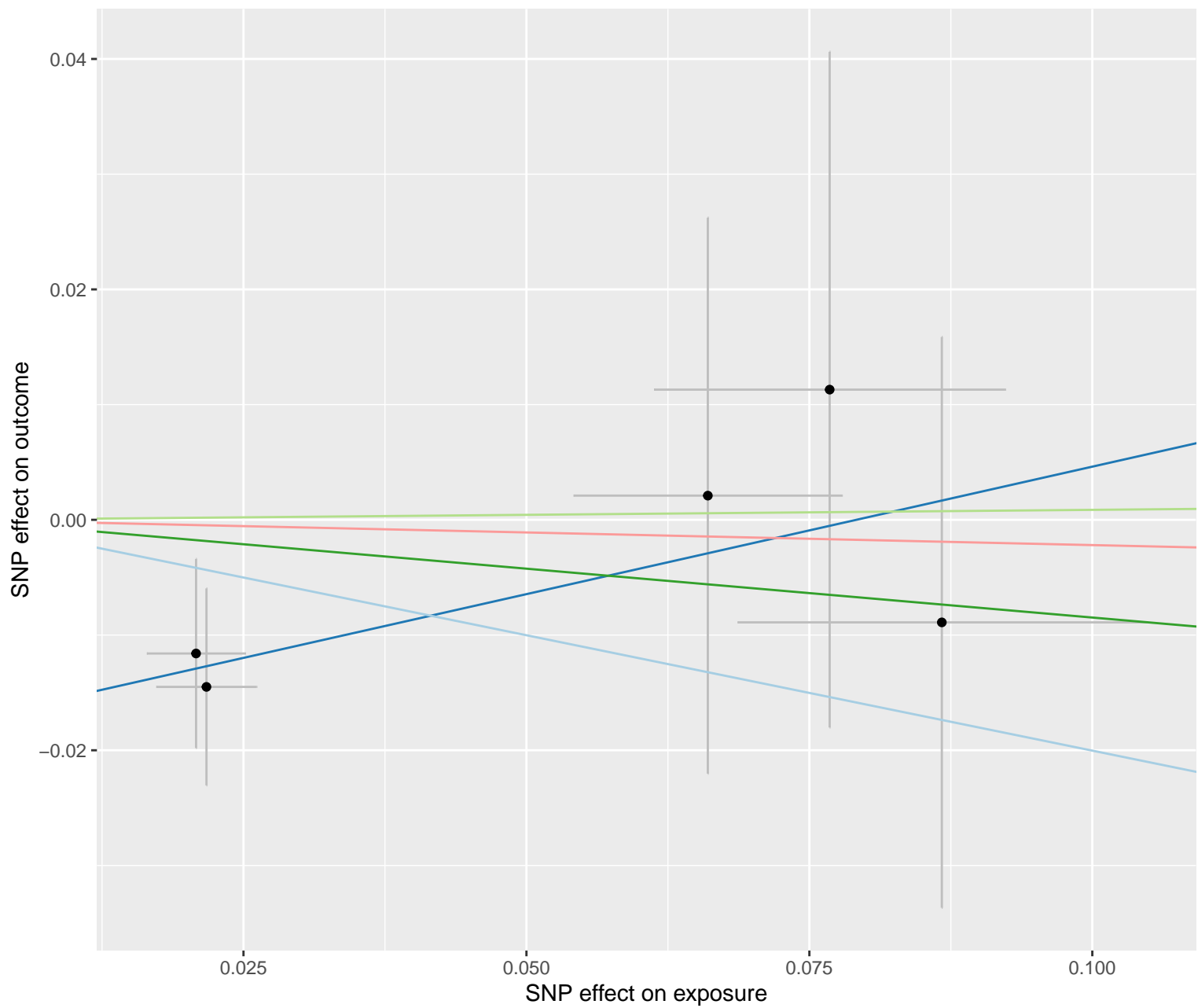

Supplement: Supplementary Data Sheet 3 — Full results of the pairwise Mendelian randomization analyses between ulcerative colitis-associated microbial taxa and ulcerative colitis-associated pyroptosis proteins, used for the downstream mediation analysis. [file DataSheet3.zip › GM_bd_fer_result/GCST90032583+3072_4_IL13_IL_13/scatter.pdf]

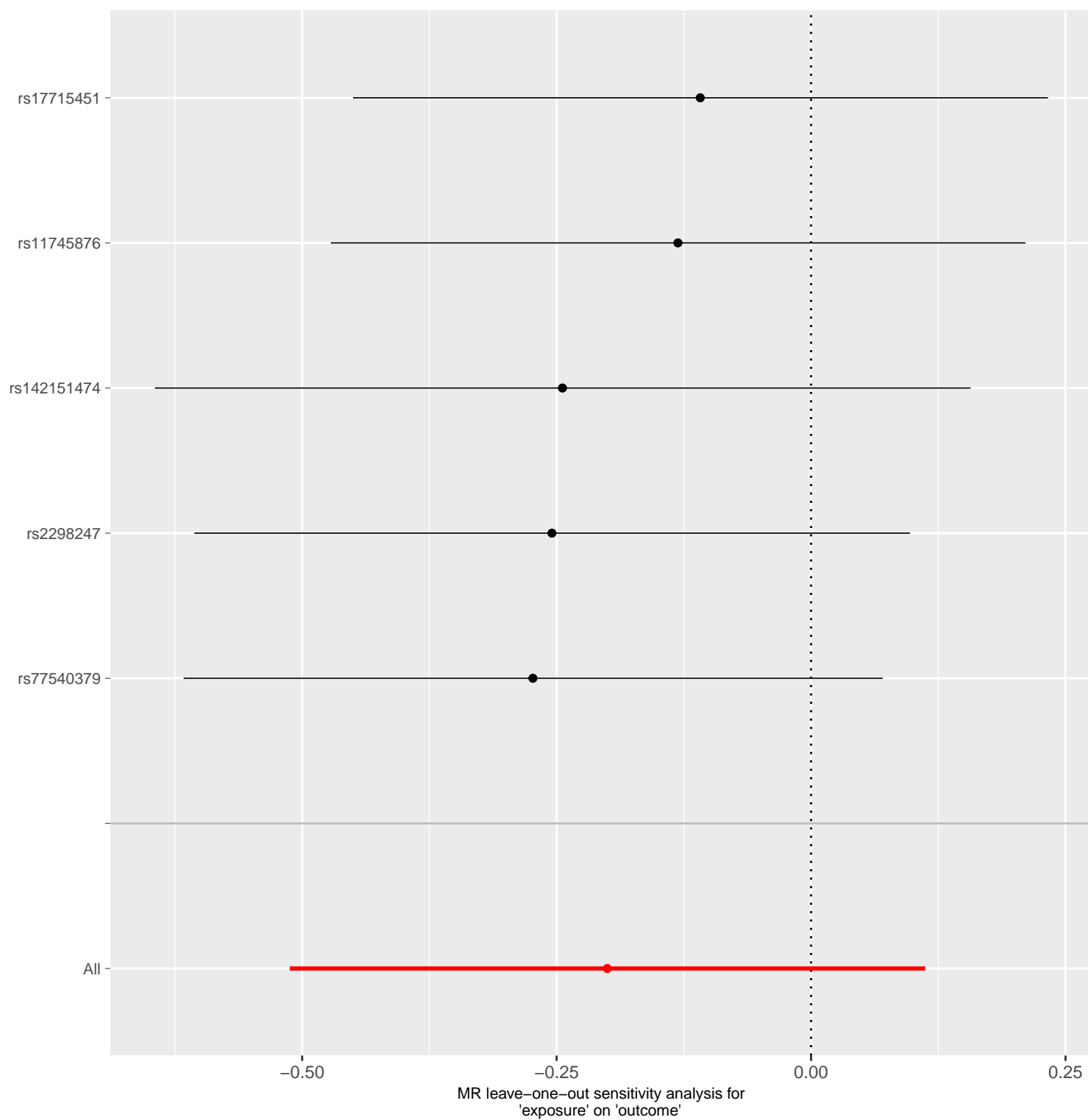

Supplement: Supplementary Data Sheet 3 — Full results of the pairwise Mendelian randomization analyses between ulcerative colitis-associated microbial taxa and ulcerative colitis-associated pyroptosis proteins, used for the downstream mediation analysis. [file DataSheet3.zip › GM_bd_fer_result/GCST90032583+3072_4_IL13_IL_13/sensitivity-analysis.pdf]

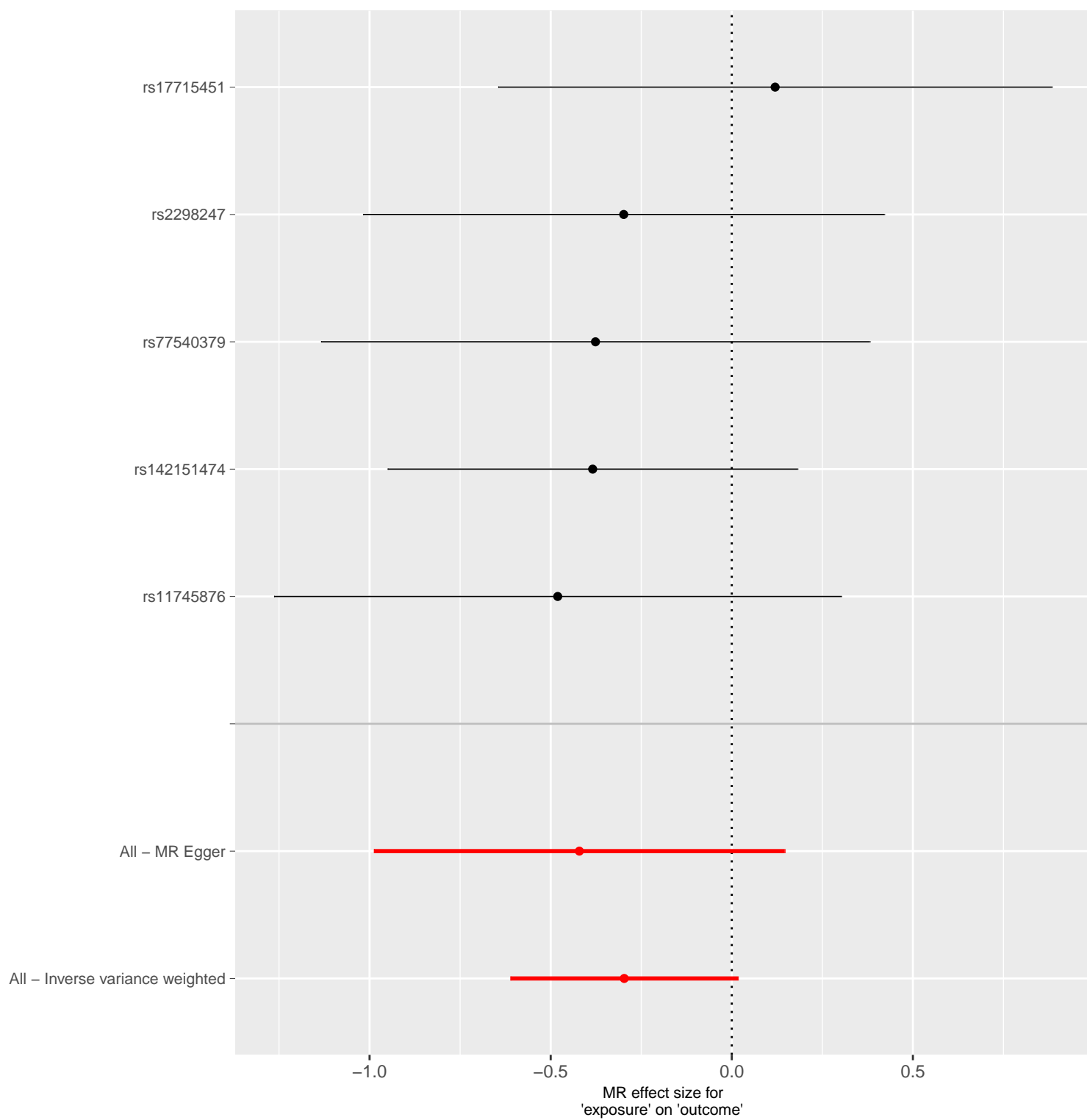

Supplement: Supplementary Data Sheet 3 — Full results of the pairwise Mendelian randomization analyses between ulcerative colitis-associated microbial taxa and ulcerative colitis-associated pyroptosis proteins, used for the downstream mediation analysis. [file DataSheet3.zip › GM_bd_fer_result/GCST90032583+3440_7_GZMA_granzyme_A/forest.pdf]
