# Supplementary material for: Integrative Mendelian randomization and experimental validation prioritize KLF4 in the gut microbiota–pyroptosis–barrier axis of ulcerative colitis
Source: Front Immunol. 2026 Mar 16;17:1773990. doi: 10.3389/fimmu.2026.1773990 (PMC13033518; doi:10.3389/fimmu.2026.1773990)
Supplement: Supplementary Data Sheet 1 — Harmonized summary data, forest plots, funnel plots, data sources, harmonization details, and sensitivity analyses for the Mendelian randomization analysis of pyroptosis-related proteins and ulcerative colitis. [file forest.pdf › bdpqtlresult/19437_61_VEGFA_L_VEGF165/forest.pdf]

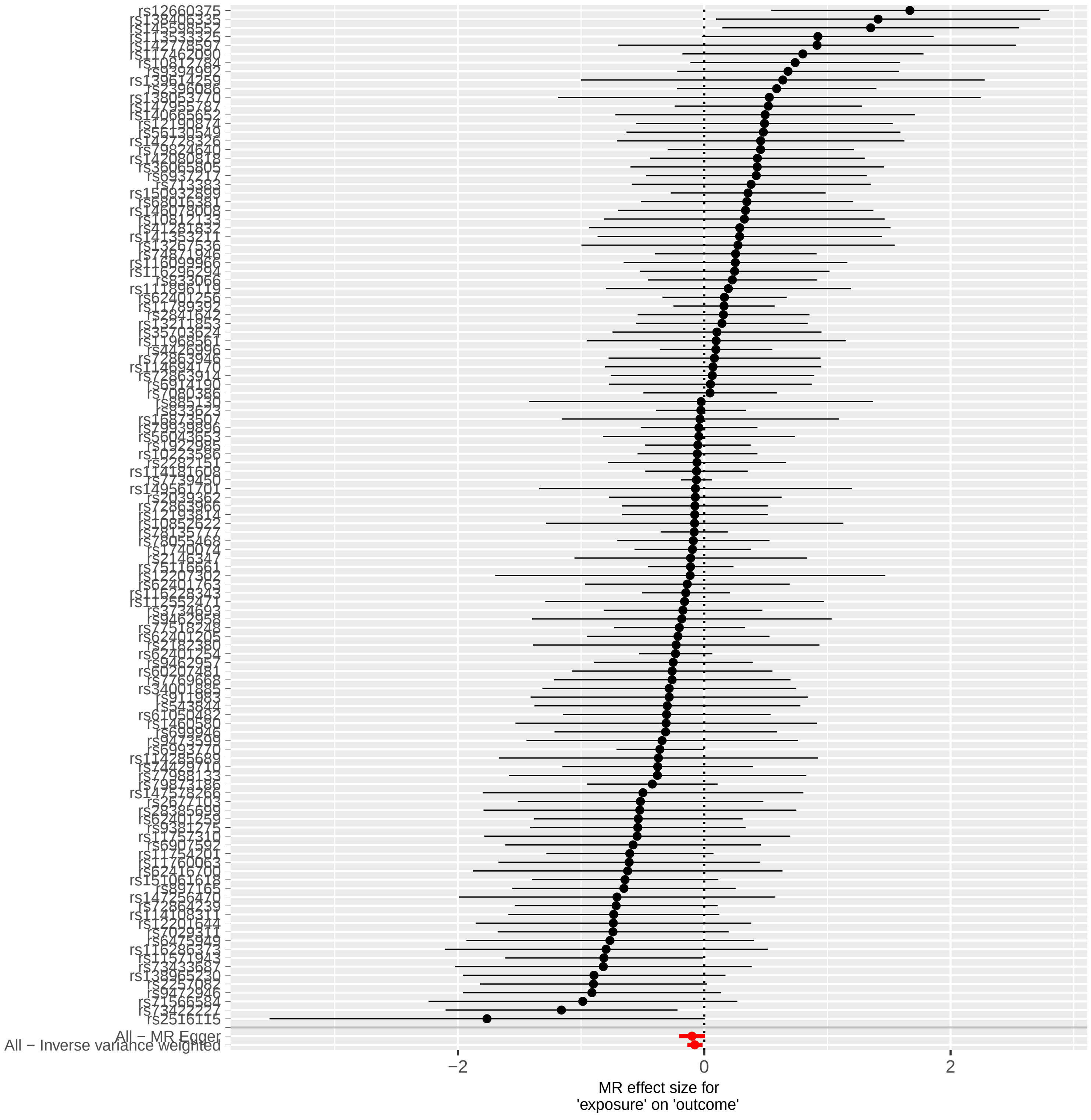
rs12660375
rs2516115
All − MR Egger
All − Inverse variance weighted
−2
0
2
MR effect size for
'exposure' on 'outcome'
