# Supplementary material for: Integrative Mendelian randomization and experimental validation prioritize KLF4 in the gut microbiota–pyroptosis–barrier axis of ulcerative colitis
Source: Front Immunol. 2026 Mar 16;17:1773990. doi: 10.3389/fimmu.2026.1773990 (PMC13033518; doi:10.3389/fimmu.2026.1773990)
Supplement: Supplementary Data Sheet 1 — Harmonized summary data, forest plots, funnel plots, data sources, harmonization details, and sensitivity analyses for the Mendelian randomization analysis of pyroptosis-related proteins and ulcerative colitis. [file DataSheet1.zip › bdpqtlresult/3719_2_CDKN1B_p27Kip1/sensitivity-analysis.pdf]

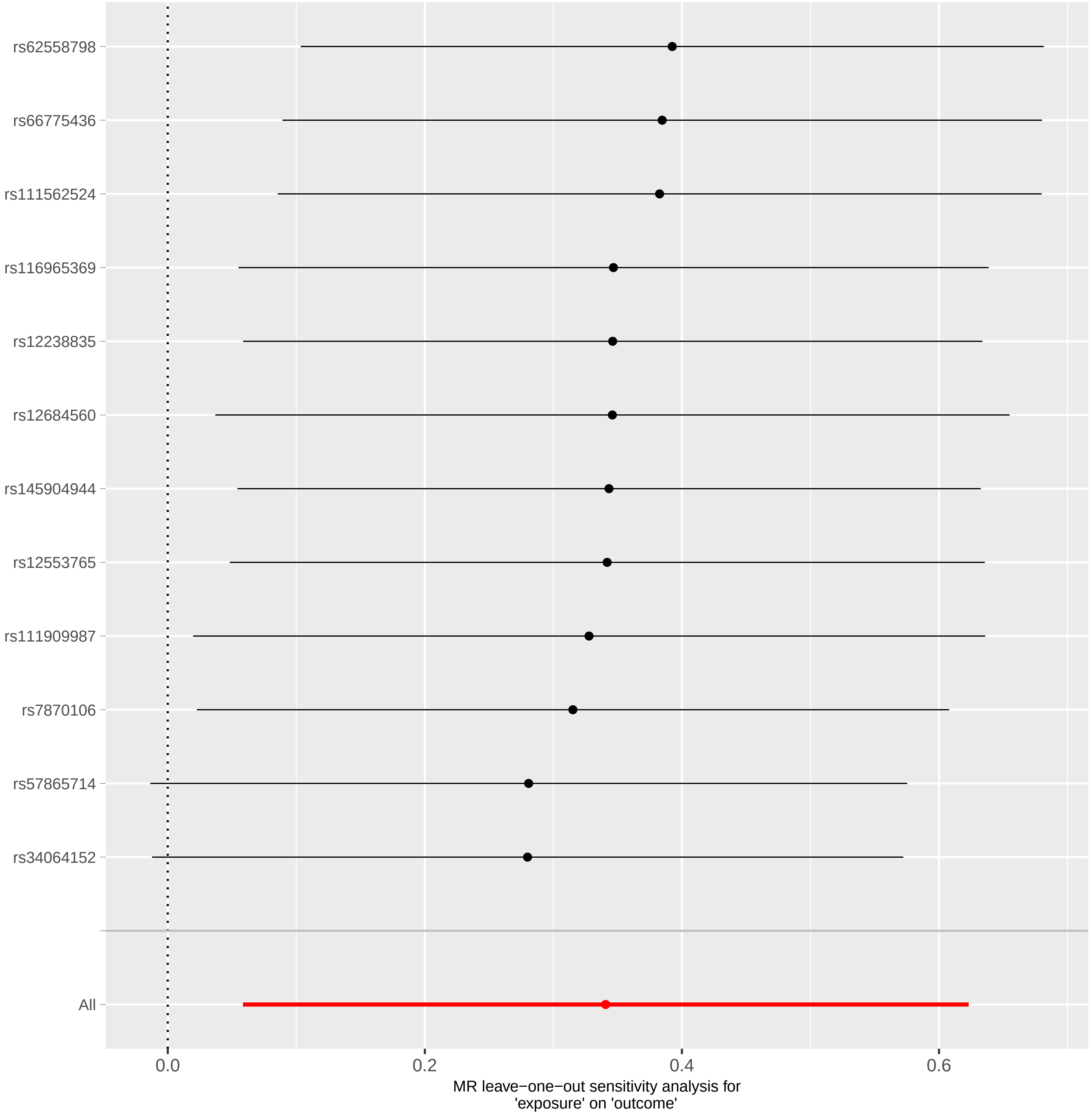
rs62558798
rs66775436
rs111562524
rs116965369
rs12238835
rs12684560
rs145904944
rs12553765
rs111909987
rs7870106
rs57865714
rs34064152
All
0.0
0.2
0.4
0.6
MR leave−one−out sensitivity analysis for
'exposure' on 'outcome'
