# Supplementary material for: Integrative Mendelian randomization and experimental validation prioritize KLF4 in the gut microbiota–pyroptosis–barrier axis of ulcerative colitis
Source: Front Immunol. 2026 Mar 16;17:1773990. doi: 10.3389/fimmu.2026.1773990 (PMC13033518; doi:10.3389/fimmu.2026.1773990)
Supplement: Supplementary Data Sheet 1 — Harmonized summary data, forest plots, funnel plots, data sources, harmonization details, and sensitivity analyses for the Mendelian randomization analysis of pyroptosis-related proteins and ulcerative colitis. [file DataSheet1.zip › bdpqtlresult/3831_21_PTEN_pTEN/funnelplot.pdf]

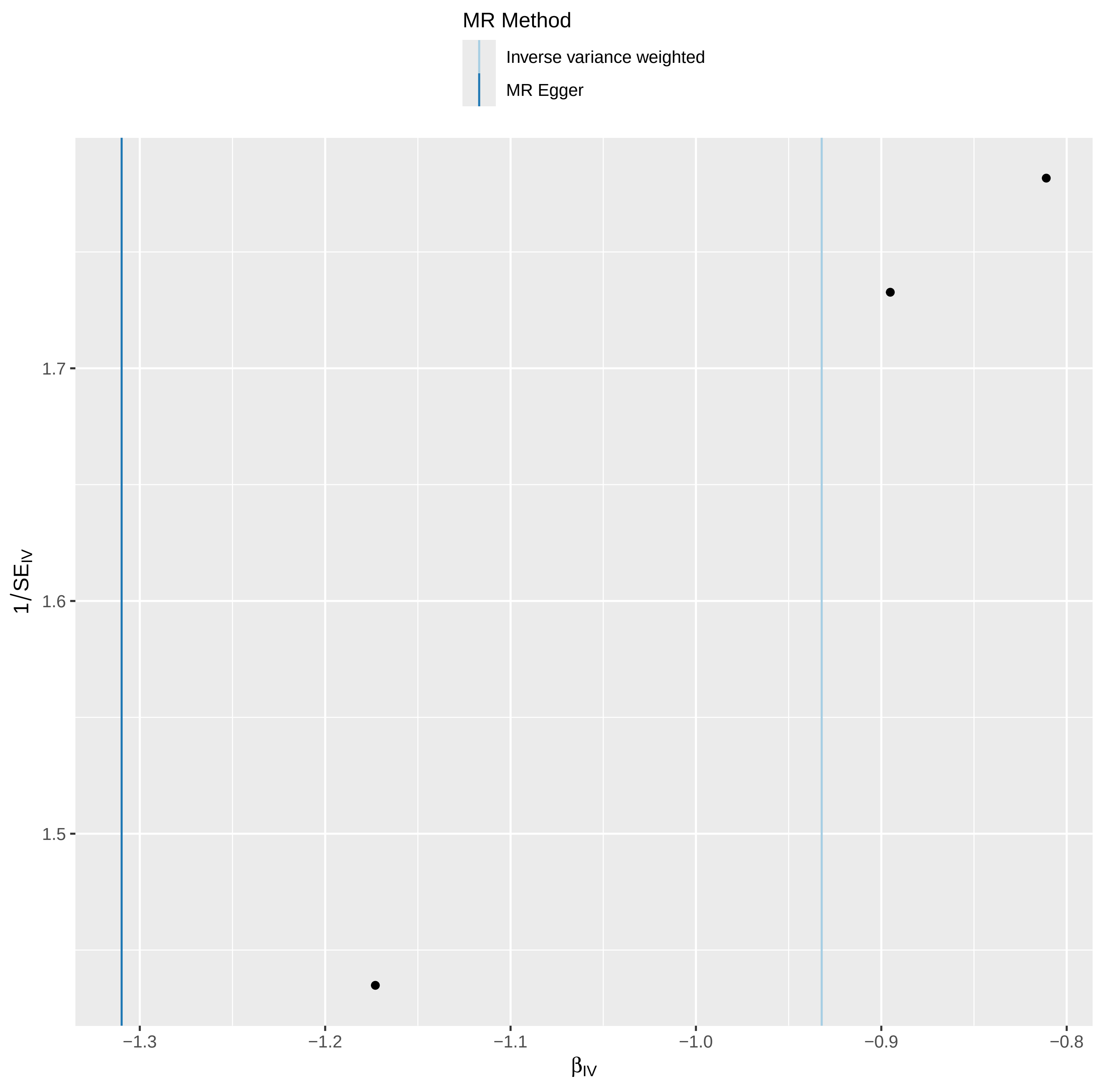

MR Method
Inverse variance weighted
MR Egger
1.7
1.6
1.5
1/SE$_{IV}$
−1.3
−1.2
−1.1
−1.0
−0.9
−0.8
β$_{IV}$
