# Supplementary material for: Integrative Mendelian randomization and experimental validation prioritize KLF4 in the gut microbiota–pyroptosis–barrier axis of ulcerative colitis
Source: Front Immunol. 2026 Mar 16;17:1773990. doi: 10.3389/fimmu.2026.1773990 (PMC13033518; doi:10.3389/fimmu.2026.1773990)
Supplement: Supplementary Data Sheet 1 — Harmonized summary data, forest plots, funnel plots, data sources, harmonization details, and sensitivity analyses for the Mendelian randomization analysis of pyroptosis-related proteins and ulcerative colitis. [file DataSheet1.zip › bdpqtlresult/4867_15_VEGFA_VEGF121/forest.pdf]

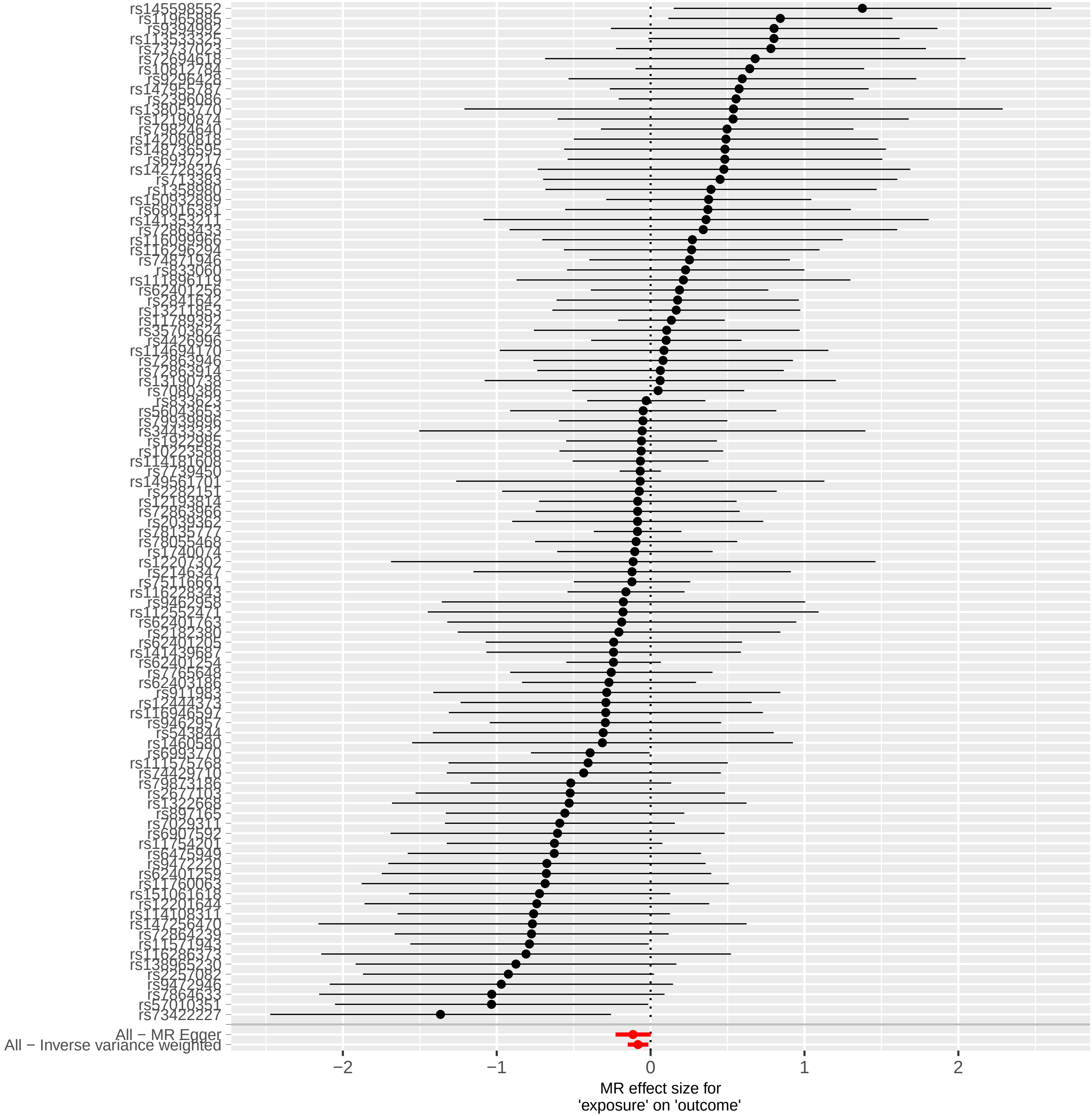
rs145598552
rs11965885
rs9394992
rs113533325
rs73737023
rs72694618
rs10812784
rs9296428
rs147955787
rs2396086
rs138053770
rs12190874
rs79824640
rs142080818
rs148736595
rs6937217
rs142728326
rs713383
rs1358980
rs150932899
rs68016381
rs141353211
rs72863433
rs116099966
rs116296294
rs74871946
rs833060
rs111896119
rs62401256
rs2841642
rs13211853
rs11789392
rs35703624
rs4426996
rs114694170
rs72863946
rs72863914
rs13190738
rs7080386
rs833623
rs56043653
rs79939896
rs34433332
rs1922985
rs10223586
rs114181608
rs7739450
rs149561701
rs2282151
rs12193814
rs72863966
rs2039362
rs78135777
rs78055468
rs1740074
rs12207302
rs2146347
rs75116661
rs116228343
rs9462958
rs112552471
rs62401763
rs2182380
rs62401205
rs141439687
rs62401254
rs7765648
rs62403186
rs911983
rs12444373
rs116946597
rs9462957
rs543844
rs1460580
rs6993770
rs111575768
rs74429710
rs79873186
rs2677103
rs1322668
rs897165
rs7029311
rs6907592
rs11754201
rs6475949
rs9472220
rs62401259
rs11760063
rs151061618
rs12201644
rs114108311
rs147256470
rs72864239
rs11571943
rs116286373
rs138965230
rs2257082
rs9472946
rs7864633
rs57010351
rs73422227
All − MR Egger
All − Inverse variance weighted
−2
−1
0
1
2
MR effect size for
'exposure' on 'outcome'
