# Supplementary material for: Integrative Mendelian randomization and experimental validation prioritize KLF4 in the gut microbiota–pyroptosis–barrier axis of ulcerative colitis
Source: Front Immunol. 2026 Mar 16;17:1773990. doi: 10.3389/fimmu.2026.1773990 (PMC13033518; doi:10.3389/fimmu.2026.1773990)
Supplement: Supplementary Data Sheet 1 — Harmonized summary data, forest plots, funnel plots, data sources, harmonization details, and sensitivity analyses for the Mendelian randomization analysis of pyroptosis-related proteins and ulcerative colitis. [file DataSheet1.zip › bdpqtlresult/4911_49_GSTP1_Glutathione_S_transferase_Pi/sensitivity-analysis.pdf]

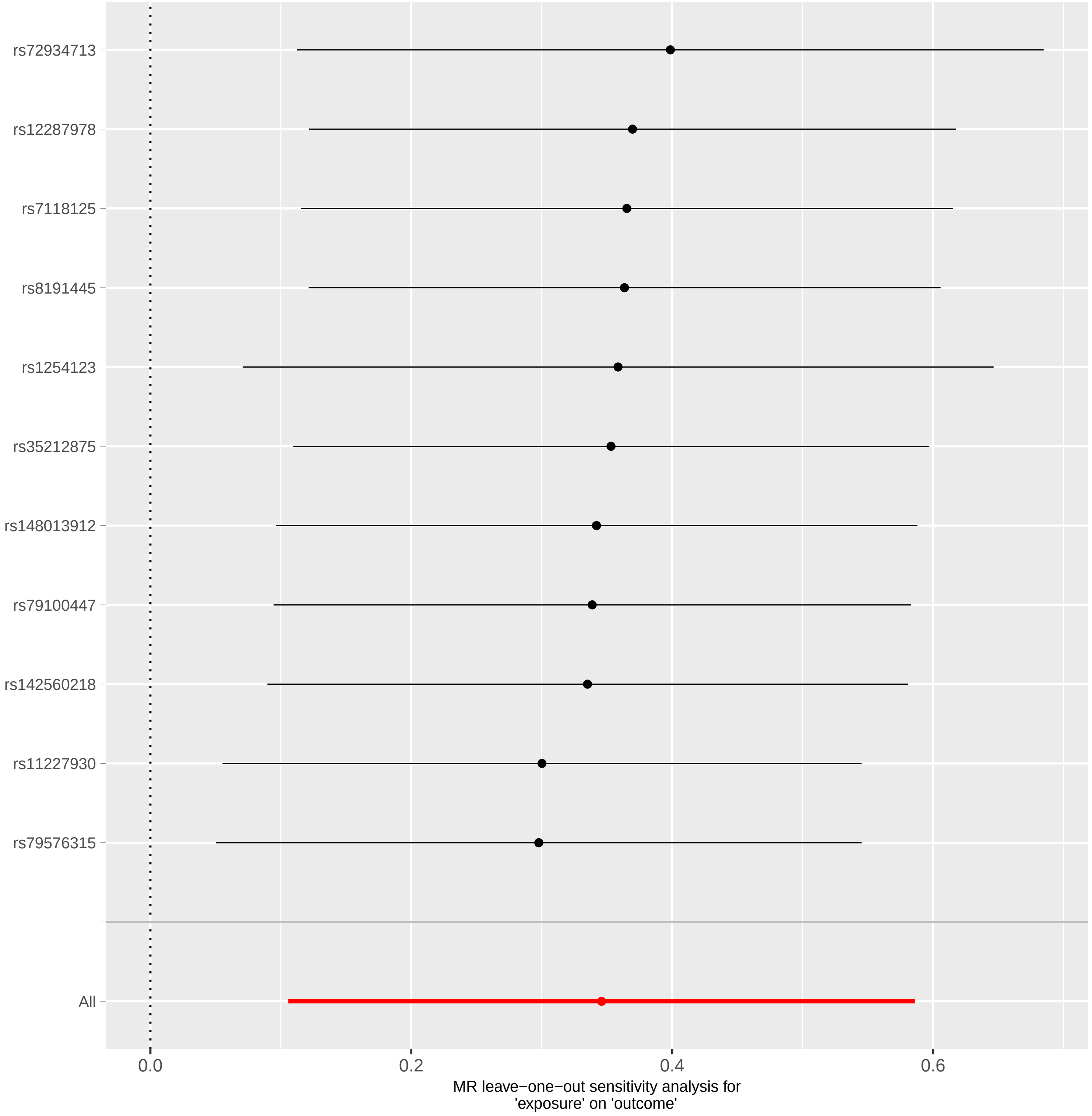
rs72934713
rs12287978
rs7118125
rs8191445
rs1254123
rs35212875
rs148013912
rs79100447
rs142560218
rs11227930
rs79576315
All
0.0
0.2
0.4
0.6
MR leave−one−out sensitivity analysis for
'exposure' on 'outcome'
