# Supplementary material for: Integrative Mendelian randomization and experimental validation prioritize KLF4 in the gut microbiota–pyroptosis–barrier axis of ulcerative colitis
Source: Front Immunol. 2026 Mar 16;17:1773990. doi: 10.3389/fimmu.2026.1773990 (PMC13033518; doi:10.3389/fimmu.2026.1773990)
Supplement: Supplementary Data Sheet 1 — Harmonized summary data, forest plots, funnel plots, data sources, harmonization details, and sensitivity analyses for the Mendelian randomization analysis of pyroptosis-related proteins and ulcerative colitis. [file DataSheet1.zip › bdpqtlresult/5231_79_PCSK9_PCSK9/forest.pdf]

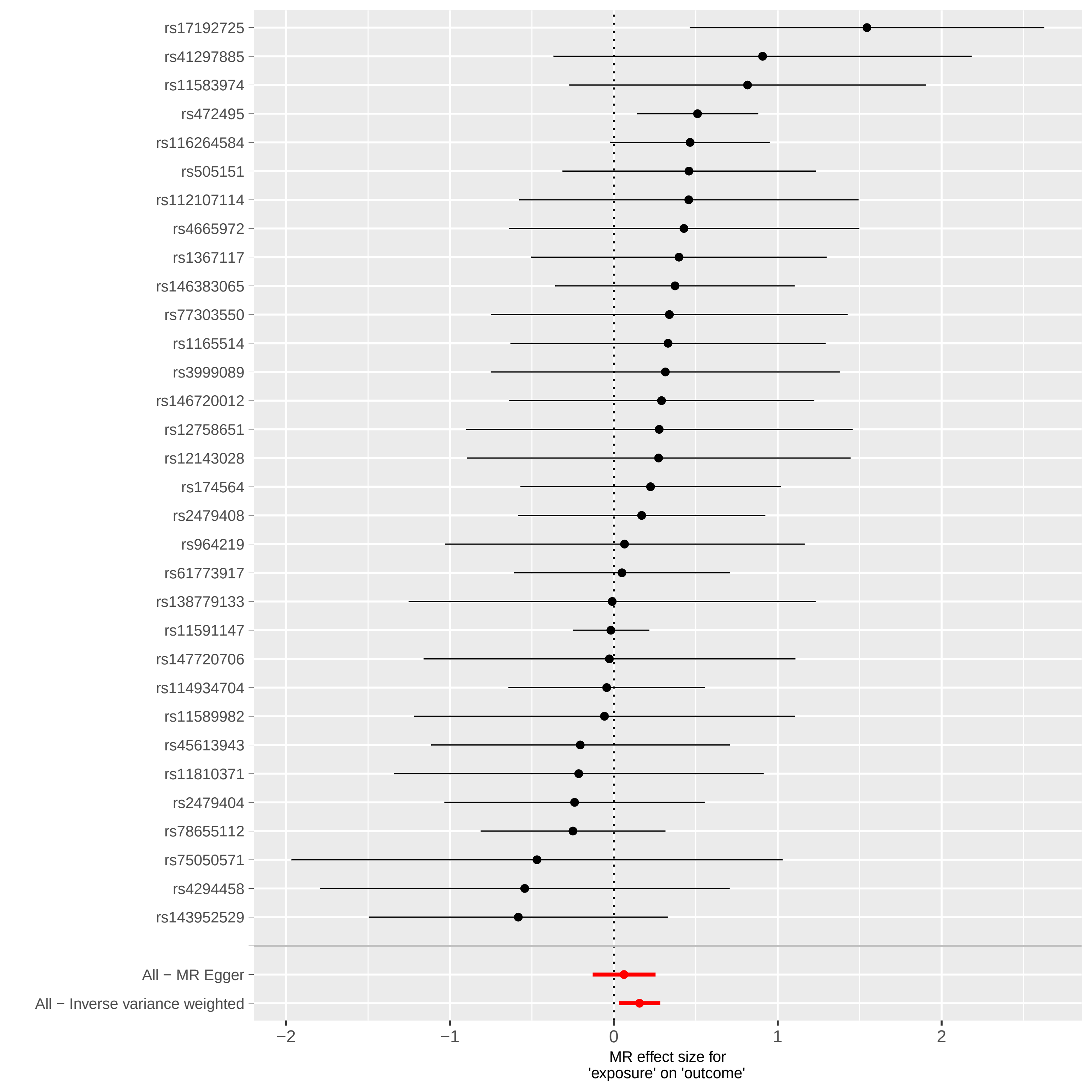

rs17192725
rs41297885
rs11583974
rs472495
rs116264584
rs505151
rs112107114
rs4665972
rs1367117
rs146383065
rs77303550
rs1165514
rs3999089
rs146720012
rs12758651
rs12143028
rs174564
rs2479408
rs964219
rs61773917
rs138779133
rs11591147
rs147720706
rs114934704
rs11589982
rs45613943
rs11810371
rs2479404
rs78655112
rs75050571
rs4294458
rs143952529
All − MR Egger
All − Inverse variance weighted
−2
−1
0
1
2
MR effect size for
'exposure' on 'outcome'
