# Supplementary material for: Integrative Mendelian randomization and experimental validation prioritize KLF4 in the gut microbiota–pyroptosis–barrier axis of ulcerative colitis
Source: Front Immunol. 2026 Mar 16;17:1773990. doi: 10.3389/fimmu.2026.1773990 (PMC13033518; doi:10.3389/fimmu.2026.1773990)
Supplement: Supplementary Data Sheet 1 — Harmonized summary data, forest plots, funnel plots, data sources, harmonization details, and sensitivity analyses for the Mendelian randomization analysis of pyroptosis-related proteins and ulcerative colitis. [file DataSheet1.zip › bdpqtlresult/5231_79_PCSK9_PCSK9/sensitivity-analysis.pdf]

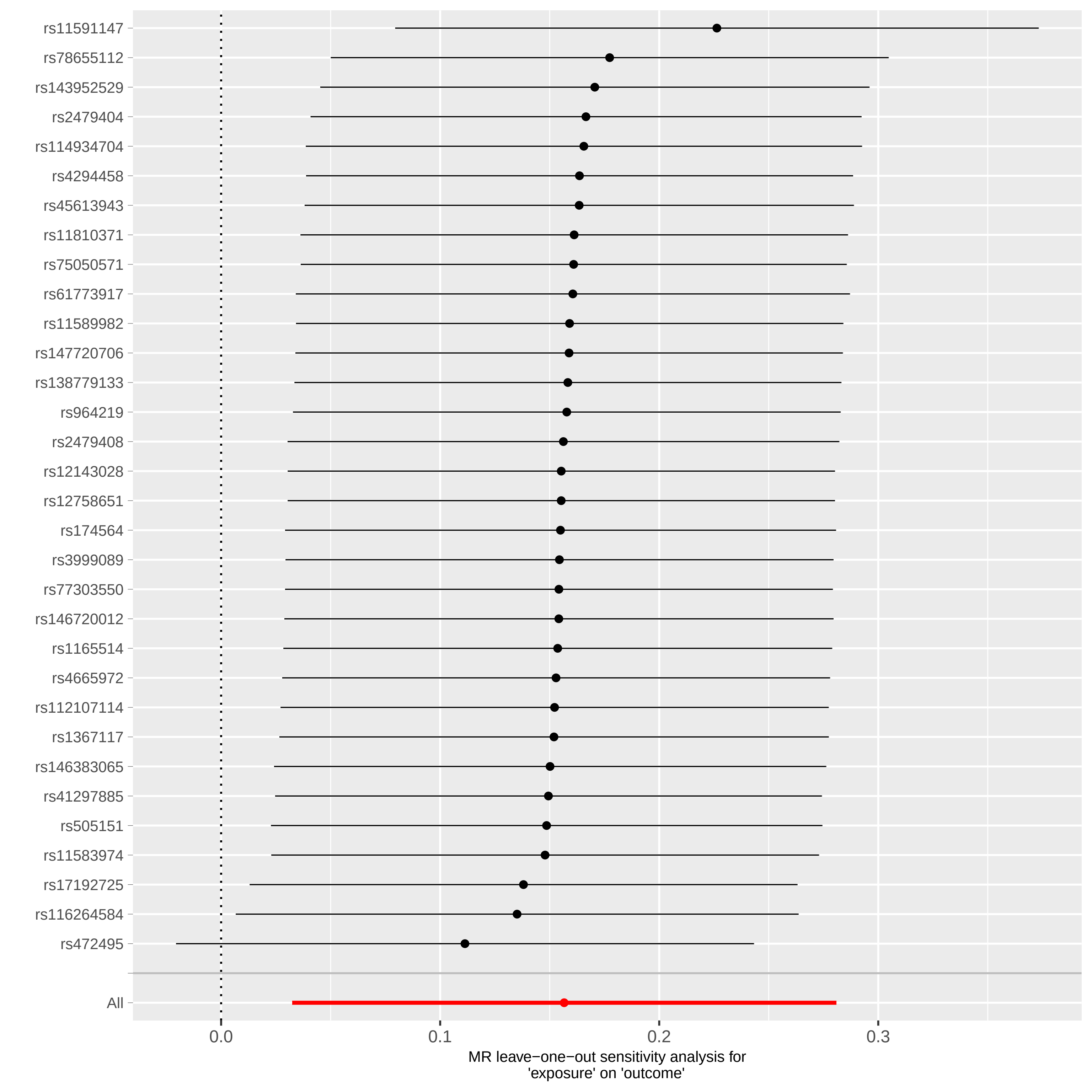
rs11591147
rs78655112
rs143952529
rs2479404
rs114934704
rs4294458
rs45613943
rs11810371
rs75050571
rs61773917
rs11589982
rs147720706
rs138779133
rs964219
rs2479408
rs12143028
rs12758651
rs174564
rs3999089
rs77303550
rs146720012
rs1165514
rs4665972
rs112107114
rs1367117
rs146383065
rs41297885
rs505151
rs11583974
rs17192725
rs116264584
rs472495
All
0.0
0.1
0.2
0.3
MR leave−one−out sensitivity analysis for
'exposure' on 'outcome'
