# Supplementary material for: Integrative Mendelian randomization and experimental validation prioritize KLF4 in the gut microbiota–pyroptosis–barrier axis of ulcerative colitis
Source: Front Immunol. 2026 Mar 16;17:1773990. doi: 10.3389/fimmu.2026.1773990 (PMC13033518; doi:10.3389/fimmu.2026.1773990)
Supplement: Supplementary Data Sheet 1 — Harmonized summary data, forest plots, funnel plots, data sources, harmonization details, and sensitivity analyses for the Mendelian randomization analysis of pyroptosis-related proteins and ulcerative colitis. [file DataSheet1.zip › bdpqtlresult/6020_52_UTS2_Urotensin_II/forest.pdf]

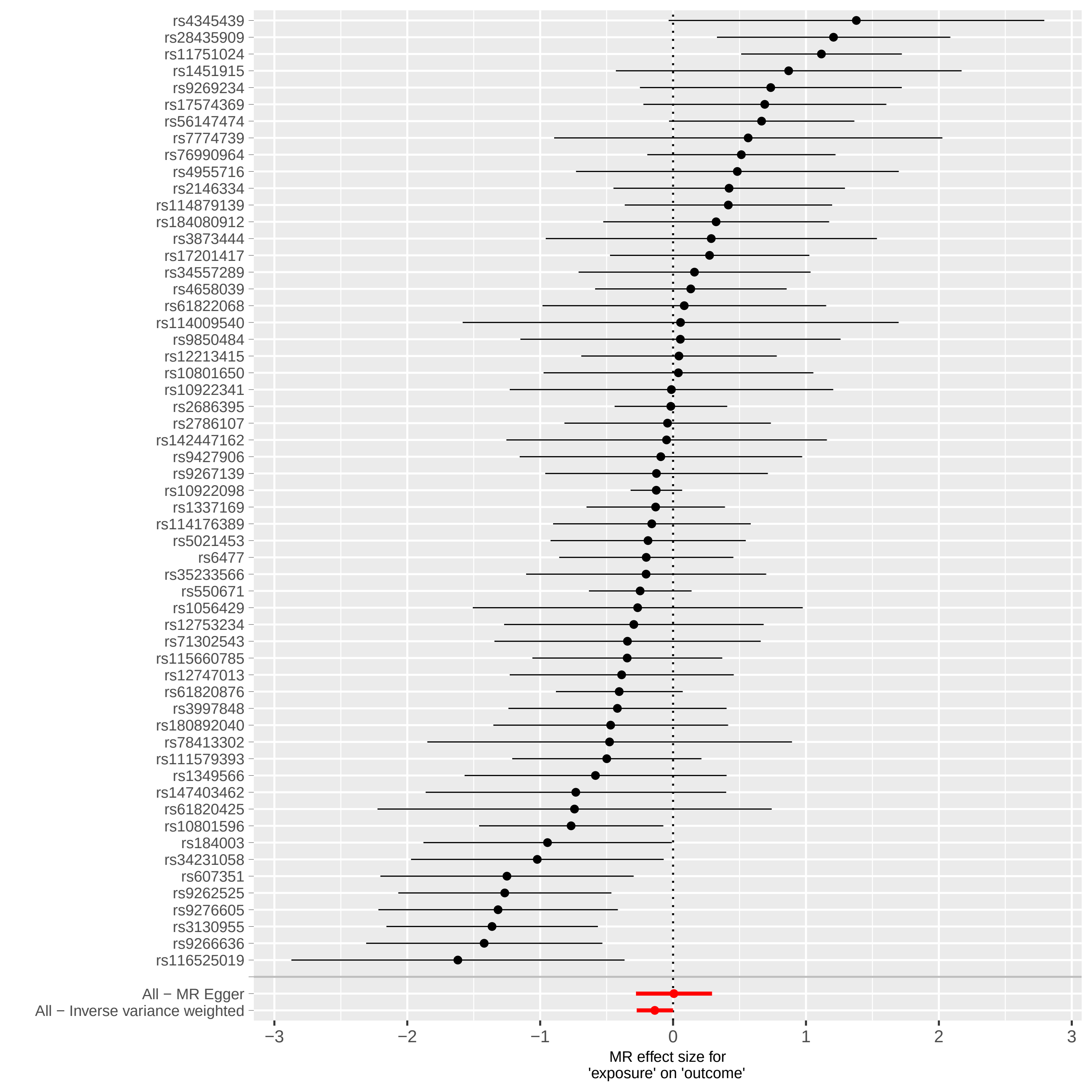
rs4345439
rs28435909
rs11751024
rs1451915
rs9269234
rs17574369
rs56147474
rs7774739
rs76990964
rs4955716
rs2146334
rs114879139
rs184080912
rs3873444
rs17201417
rs34557289
rs4658039
rs61822068
rs114009540
rs9850484
rs12213415
rs10801650
rs10922341
rs2686395
rs2786107
rs142447162
rs9427906
rs9267139
rs10922098
rs1337169
rs114176389
rs5021453
rs6477
rs35233566
rs550671
rs1056429
rs12753234
rs71302543
rs115660785
rs12747013
rs61820876
rs3997848
rs180892040
rs78413302
rs111579393
rs1349566
rs147403462
rs61820425
rs10801596
rs184003
rs34231058
rs607351
rs9262525
rs9276605
rs3130955
rs9266636
rs116525019
All − MR Egger
All − Inverse variance weighted
−3
−2
−1
0
1
2
3
MR effect size for
'exposure' on 'outcome'
