# Supplementary material for: Integrative Mendelian randomization and experimental validation prioritize KLF4 in the gut microbiota–pyroptosis–barrier axis of ulcerative colitis
Source: Front Immunol. 2026 Mar 16;17:1773990. doi: 10.3389/fimmu.2026.1773990 (PMC13033518; doi:10.3389/fimmu.2026.1773990)
Supplement: Supplementary Data Sheet 1 — Harmonized summary data, forest plots, funnel plots, data sources, harmonization details, and sensitivity analyses for the Mendelian randomization analysis of pyroptosis-related proteins and ulcerative colitis. [file DataSheet1.zip › bdpqtlresult/6020_52_UTS2_Urotensin_II/funnelplot.pdf]

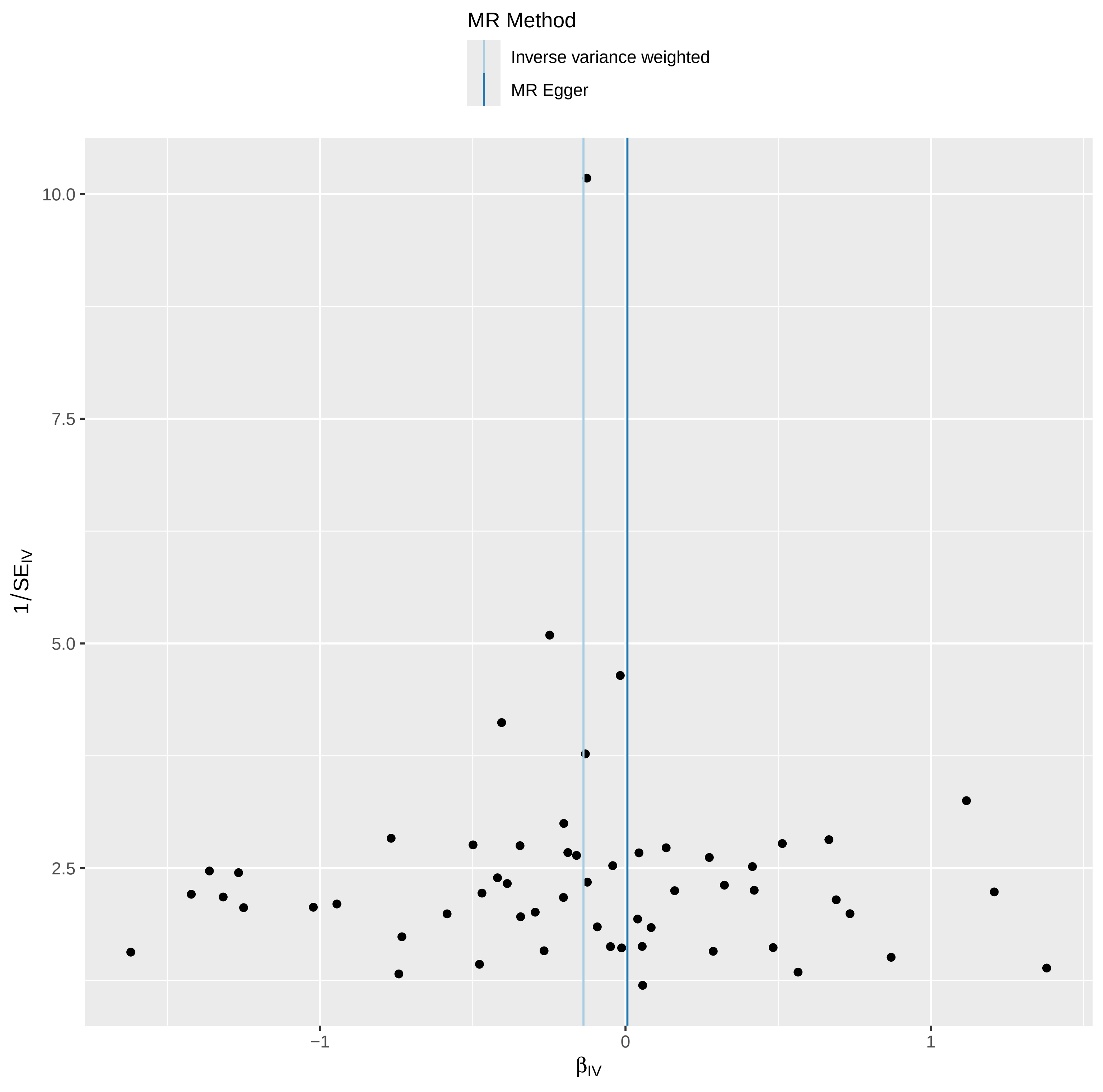
MR Method
Inverse variance weighted
MR Egger
10.0
7.5
5.0
2.5
−1
0
1
$1/SE_{IV}$
β_{IV}
