# Supplementary material for: Integrative Mendelian randomization and experimental validation prioritize KLF4 in the gut microbiota–pyroptosis–barrier axis of ulcerative colitis
Source: Front Immunol. 2026 Mar 16;17:1773990. doi: 10.3389/fimmu.2026.1773990 (PMC13033518; doi:10.3389/fimmu.2026.1773990)
Supplement: Supplementary Data Sheet 1 — Harmonized summary data, forest plots, funnel plots, data sources, harmonization details, and sensitivity analyses for the Mendelian randomization analysis of pyroptosis-related proteins and ulcerative colitis. [file DataSheet1.zip › bdpqtlresult/6020_52_UTS2_Urotensin_II/sensitivity-analysis.pdf]

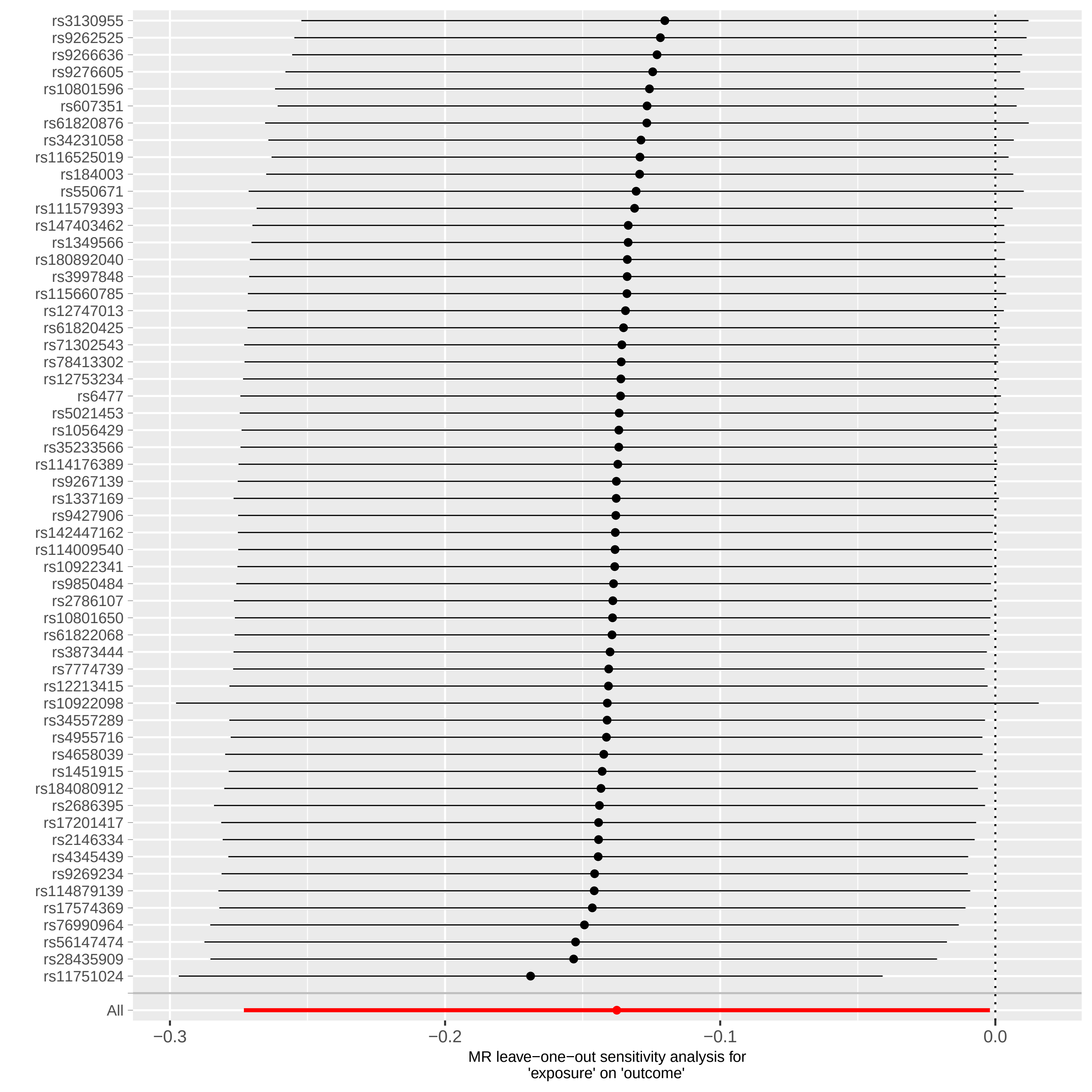
rs3130955
rs9262525
rs9266636
rs9276605
rs10801596
rs607351
rs61820876
rs34231058
rs116525019
rs184003
rs550671
rs111579393
rs147403462
rs1349566
rs180892040
rs3997848
rs115660785
rs12747013
rs61820425
rs71302543
rs78413302
rs12753234
rs6477
rs5021453
rs1056429
rs35233566
rs114176389
rs9267139
rs1337169
rs9427906
rs142447162
rs114009540
rs10922341
rs9850484
rs2786107
rs10801650
rs61822068
rs3873444
rs7774739
rs12213415
rs10922098
rs34557289
rs4955716
rs4658039
rs1451915
rs184080912
rs2686395
rs17201417
rs2146334
rs4345439
rs9269234
rs114879139
rs17574369
rs76990964
rs56147474
rs28435909
rs11751024
All
−0.3
−0.2
−0.1
0.0
MR leave−one−out sensitivity analysis for
'exposure' on 'outcome'
