# Supplementary material for: Integrative Mendelian randomization and experimental validation prioritize KLF4 in the gut microbiota–pyroptosis–barrier axis of ulcerative colitis
Source: Front Immunol. 2026 Mar 16;17:1773990. doi: 10.3389/fimmu.2026.1773990 (PMC13033518; doi:10.3389/fimmu.2026.1773990)
Supplement: Supplementary Data Sheet 1 — Harmonized summary data, forest plots, funnel plots, data sources, harmonization details, and sensitivity analyses for the Mendelian randomization analysis of pyroptosis-related proteins and ulcerative colitis. [file DataSheet1.zip › bdpqtlresult/8231_122_FLT1_VEGF_sR1/forest.pdf]

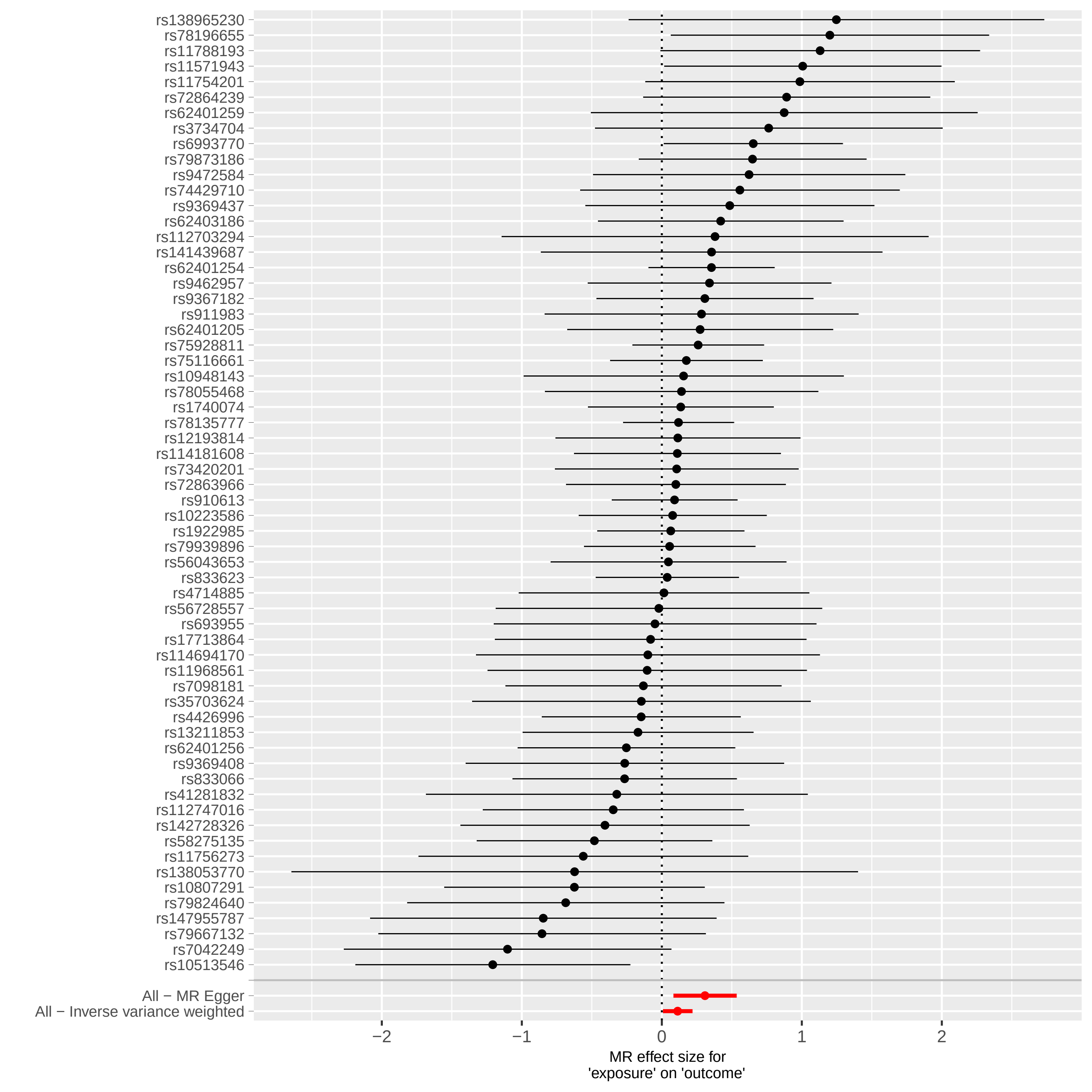
rs138965230
rs78196655
rs11788193
rs11571943
rs11754201
rs72864239
rs62401259
rs3734704
rs6993770
rs79873186
rs9472584
rs74429710
rs9369437
rs62403186
rs112703294
rs141439687
rs62401254
rs9462957
rs9367182
rs911983
rs62401205
rs75928811
rs75116661
rs10948143
rs78055468
rs1740074
rs78135777
rs12193814
rs114181608
rs73420201
rs72863966
rs910613
rs10223586
rs1922985
rs79939896
rs56043653
rs833623
rs4714885
rs56728557
rs693955
rs17713864
rs114694170
rs11968561
rs7098181
rs35703624
rs4426996
rs13211853
rs62401256
rs9369408
rs833066
rs41281832
rs112747016
rs142728326
rs58275135
rs11756273
rs138053770
rs10807291
rs79824640
rs147955787
rs79667132
rs7042249
rs10513546
All − MR Egger
All − Inverse variance weighted
−2
−1
0
1
2
MR effect size for
'exposure' on 'outcome'
