# Supplementary material for: Integrative Mendelian randomization and experimental validation prioritize KLF4 in the gut microbiota–pyroptosis–barrier axis of ulcerative colitis
Source: Front Immunol. 2026 Mar 16;17:1773990. doi: 10.3389/fimmu.2026.1773990 (PMC13033518; doi:10.3389/fimmu.2026.1773990)
Supplement: Supplementary Data Sheet 1 — Harmonized summary data, forest plots, funnel plots, data sources, harmonization details, and sensitivity analyses for the Mendelian randomization analysis of pyroptosis-related proteins and ulcerative colitis. [file DataSheet1.zip › bdpqtlresult/8231_122_FLT1_VEGF_sR1/sensitivity-analysis.pdf]

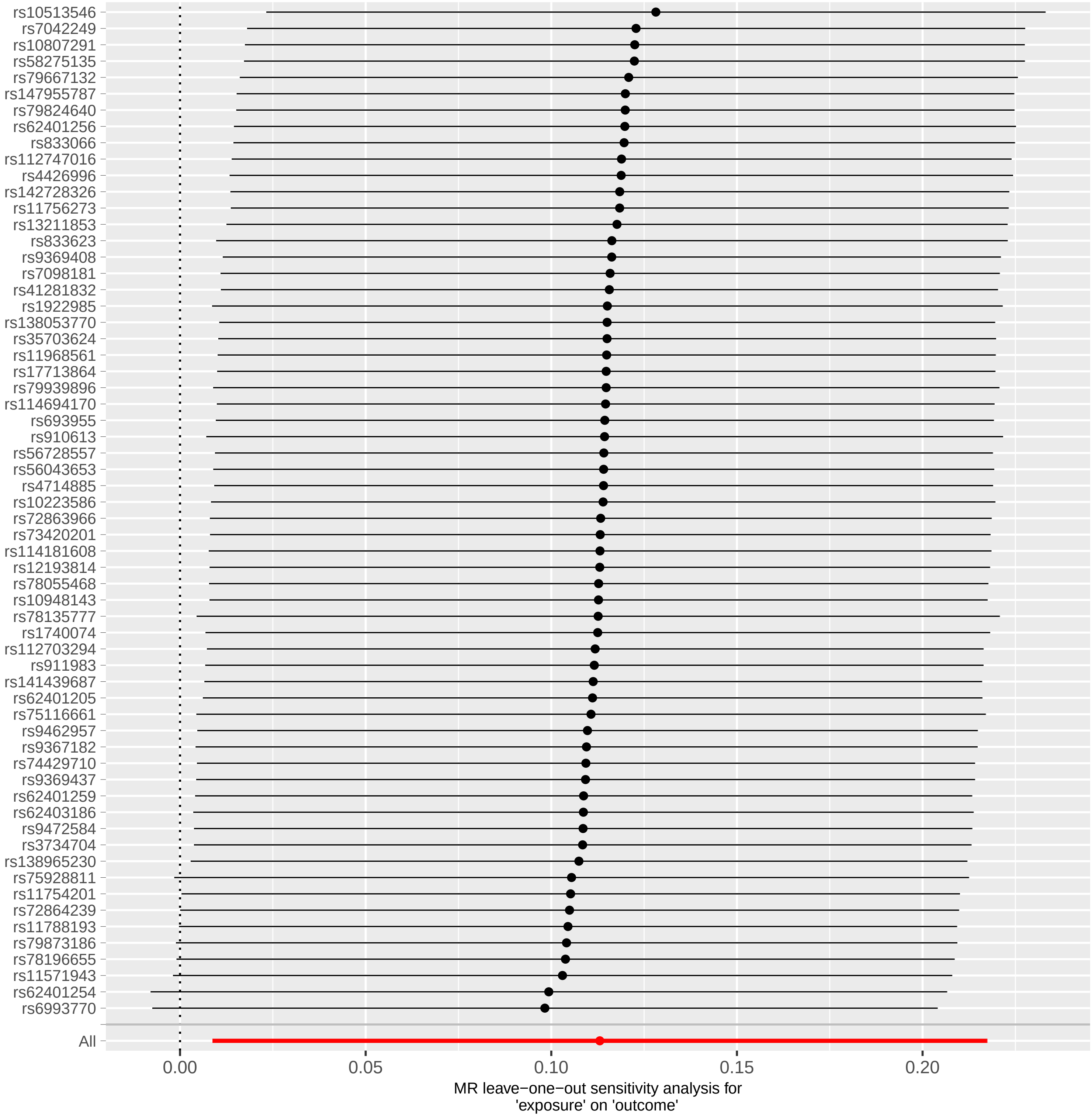
rs10513546
rs7042249
rs10807291
rs58275135
rs79667132
rs147955787
rs79824640
rs62401256
rs833066
rs112747016
rs4426996
rs142728326
rs11756273
rs13211853
rs833623
rs9369408
rs7098181
rs41281832
rs1922985
rs138053770
rs35703624
rs11968561
rs17713864
rs79939896
rs114694170
rs693955
rs910613
rs56728557
rs56043653
rs4714885
rs10223586
rs72863966
rs73420201
rs114181608
rs12193814
rs78055468
rs10948143
rs78135777
rs1740074
rs112703294
rs911983
rs141439687
rs62401205
rs75116661
rs9462957
rs9367182
rs74429710
rs9369437
rs62401259
rs62403186
rs9472584
rs3734704
rs138965230
rs75928811
rs11754201
rs72864239
rs11788193
rs79873186
rs78196655
rs11571943
rs62401254
rs6993770
All
0.00
0.05
0.10
0.15
0.20
MR leave−one−out sensitivity analysis for
'exposure' on 'outcome'
