# Supplementary material for: Integrative Mendelian randomization and experimental validation prioritize KLF4 in the gut microbiota–pyroptosis–barrier axis of ulcerative colitis
Source: Front Immunol. 2026 Mar 16;17:1773990. doi: 10.3389/fimmu.2026.1773990 (PMC13033518; doi:10.3389/fimmu.2026.1773990)
Supplement: Supplementary Data Sheet 1 — Harmonized summary data, forest plots, funnel plots, data sources, harmonization details, and sensitivity analyses for the Mendelian randomization analysis of pyroptosis-related proteins and ulcerative colitis. [file DataSheet1.zip › bdpqtlresult/8424_269_CTNNB1_b_Catenin/forest.pdf]

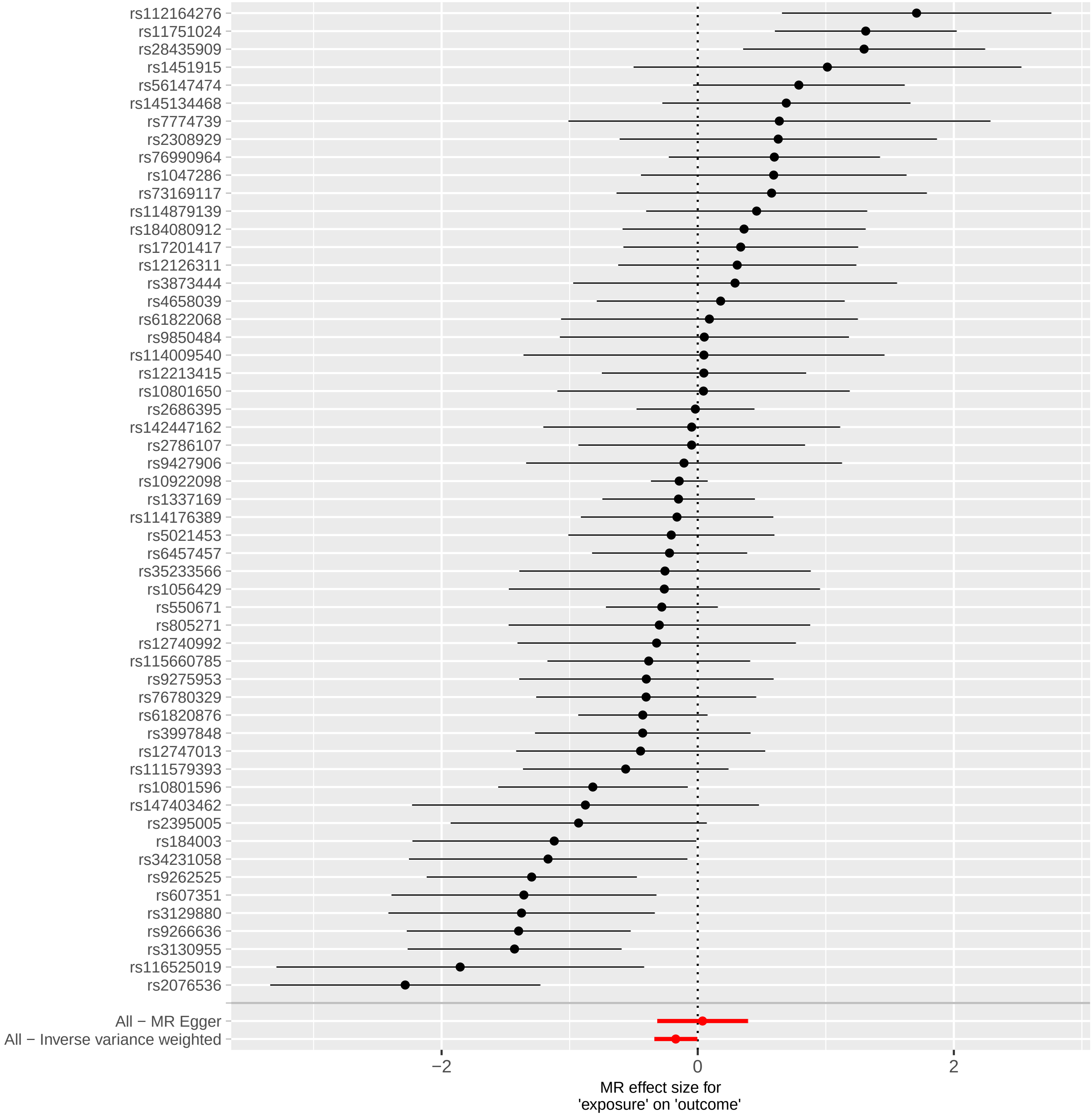
rs112164276
rs11751024
rs28435909
rs1451915
rs56147474
rs145134468
rs7774739
rs2308929
rs76990964
rs1047286
rs73169117
rs114879139
rs184080912
rs17201417
rs12126311
rs3873444
rs4658039
rs61822068
rs9850484
rs114009540
rs12213415
rs10801650
rs2686395
rs142447162
rs2786107
rs9427906
rs10922098
rs1337169
rs114176389
rs5021453
rs6457457
rs35233566
rs1056429
rs550671
rs805271
rs12740992
rs115660785
rs9275953
rs76780329
rs61820876
rs3997848
rs12747013
rs111579393
rs10801596
rs147403462
rs2395005
rs184003
rs34231058
rs9262525
rs607351
rs3129880
rs9266636
rs3130955
rs116525019
rs2076536
All − MR Egger
All − Inverse variance weighted
−2
0
2
MR effect size for
'exposure' on 'outcome'
