# Supplementary material for: Integrative Mendelian randomization and experimental validation prioritize KLF4 in the gut microbiota–pyroptosis–barrier axis of ulcerative colitis
Source: Front Immunol. 2026 Mar 16;17:1773990. doi: 10.3389/fimmu.2026.1773990 (PMC13033518; doi:10.3389/fimmu.2026.1773990)
Supplement: Supplementary Data Sheet 1 — Harmonized summary data, forest plots, funnel plots, data sources, harmonization details, and sensitivity analyses for the Mendelian randomization analysis of pyroptosis-related proteins and ulcerative colitis. [file DataSheet1.zip › bdpqtlresult/8424_269_CTNNB1_b_Catenin/sensitivity-analysis.pdf]

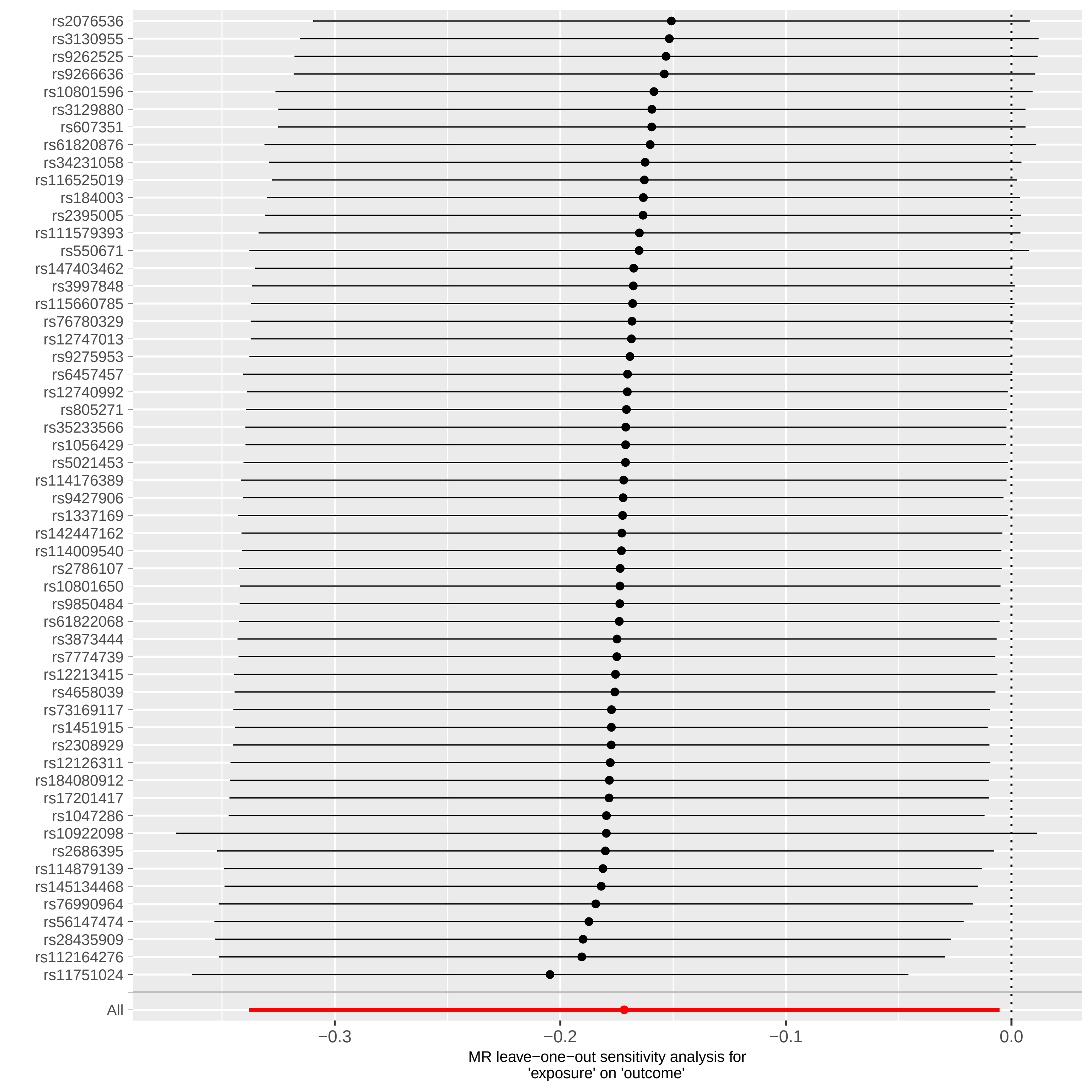
rs2076536
rs3130955
rs9262525
rs9266636
rs10801596
rs3129880
rs607351
rs61820876
rs34231058
rs116525019
rs184003
rs2395005
rs111579393
rs550671
rs147403462
rs3997848
rs115660785
rs76780329
rs12747013
rs9275953
rs6457457
rs12740992
rs805271
rs35233566
rs1056429
rs5021453
rs114176389
rs9427906
rs1337169
rs142447162
rs114009540
rs2786107
rs10801650
rs9850484
rs61822068
rs3873444
rs7774739
rs12213415
rs4658039
rs73169117
rs1451915
rs2308929
rs12126311
rs184080912
rs17201417
rs1047286
rs10922098
rs2686395
rs114879139
rs145134468
rs76990964
rs56147474
rs28435909
rs112164276
rs11751024
All
−0.3
−0.2
−0.1
0.0
MR leave−one−out sensitivity analysis for
'exposure' on 'outcome'
