# Supplementary material for: Integrative Mendelian randomization and experimental validation prioritize KLF4 in the gut microbiota–pyroptosis–barrier axis of ulcerative colitis
Source: Front Immunol. 2026 Mar 16;17:1773990. doi: 10.3389/fimmu.2026.1773990 (PMC13033518; doi:10.3389/fimmu.2026.1773990)
Supplement: Supplementary Data Sheet 1 — Harmonized summary data, forest plots, funnel plots, data sources, harmonization details, and sensitivity analyses for the Mendelian randomization analysis of pyroptosis-related proteins and ulcerative colitis. [file DataSheet1.zip › bdpqtlresult/8969_49_CD14_CD14/forest.pdf]

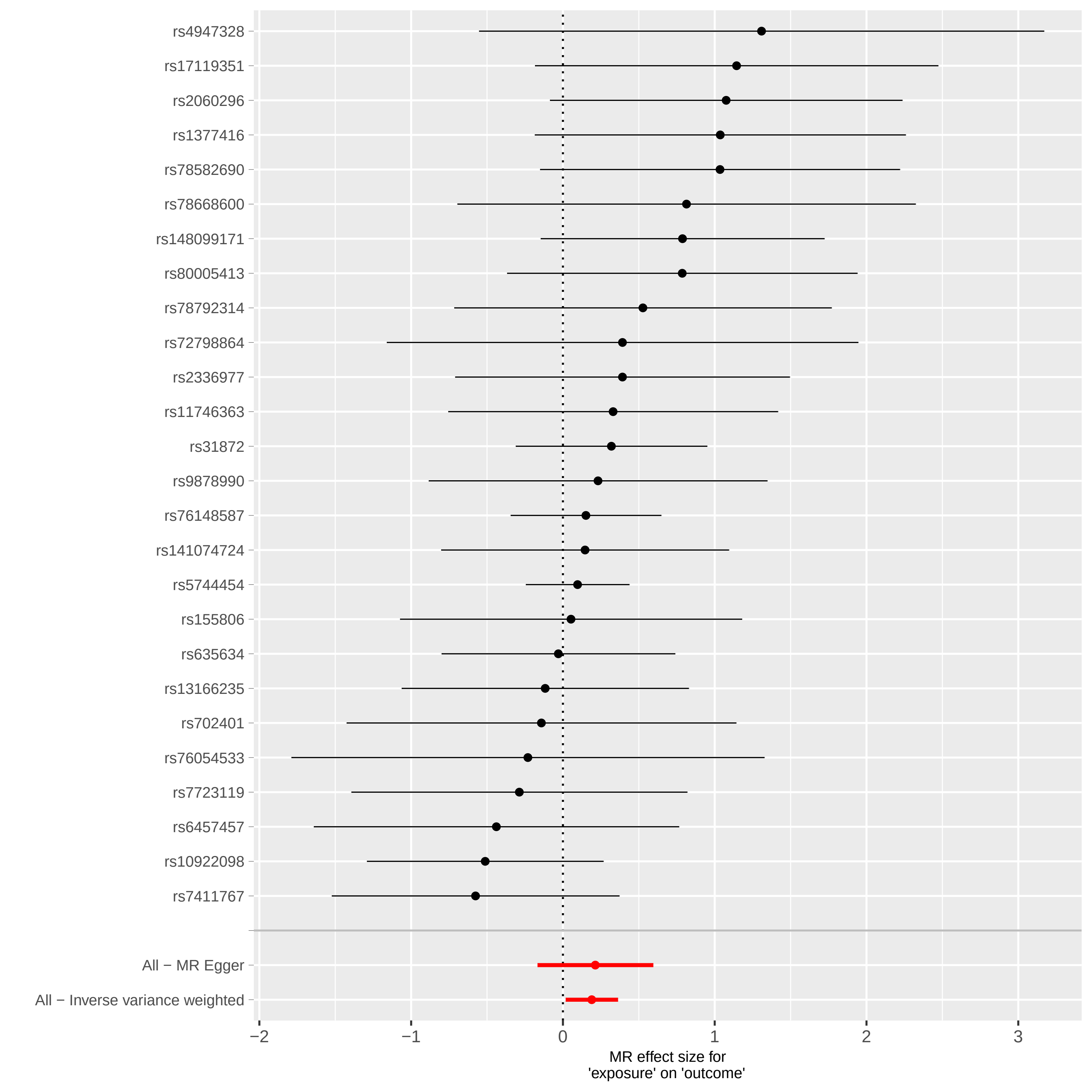

rs4947328
rs17119351
rs2060296
rs1377416
rs78582690
rs78668600
rs148099171
rs80005413
rs78792314
rs72798864
rs2336977
rs11746363
rs31872
rs9878990
rs76148587
rs141074724
rs5744454
rs155806
rs635634
rs13166235
rs702401
rs76054533
rs7723119
rs6457457
rs10922098
rs7411767
All − MR Egger
All − Inverse variance weighted
−2
−1
0
1
2
3
MR effect size for
'exposure' on 'outcome'
