# Supplementary material for: Integrative Mendelian randomization and experimental validation prioritize KLF4 in the gut microbiota–pyroptosis–barrier axis of ulcerative colitis
Source: Front Immunol. 2026 Mar 16;17:1773990. doi: 10.3389/fimmu.2026.1773990 (PMC13033518; doi:10.3389/fimmu.2026.1773990)
Supplement: Supplementary Data Sheet 1 — Harmonized summary data, forest plots, funnel plots, data sources, harmonization details, and sensitivity analyses for the Mendelian randomization analysis of pyroptosis-related proteins and ulcerative colitis. [file DataSheet1.zip › bdpqtlresult/8969_49_CD14_CD14/sensitivity-analysis.pdf]

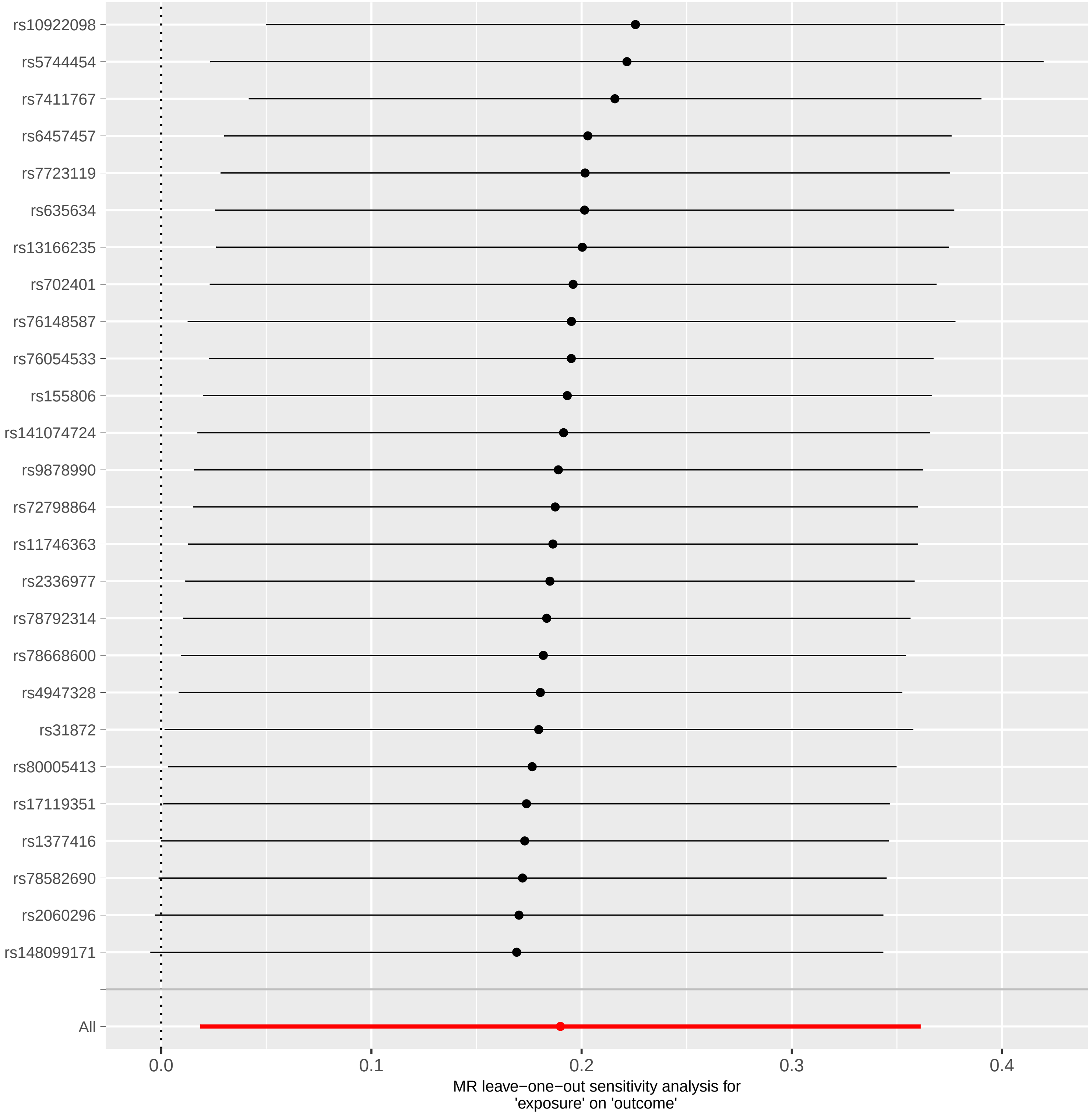
rs10922098
rs5744454
rs7411767
rs6457457
rs7723119
rs635634
rs13166235
rs702401
rs76148587
rs76054533
rs155806
rs141074724
rs9878990
rs72798864
rs11746363
rs2336977
rs78792314
rs78668600
rs4947328
rs31872
rs80005413
rs17119351
rs1377416
rs78582690
rs2060296
rs148099171
All
0.0
0.1
0.2
0.3
0.4
MR leave−one−out sensitivity analysis for
'exposure' on 'outcome'
