# Supplementary material for: Integrative Mendelian randomization and experimental validation prioritize KLF4 in the gut microbiota–pyroptosis–barrier axis of ulcerative colitis
Source: Front Immunol. 2026 Mar 16;17:1773990. doi: 10.3389/fimmu.2026.1773990 (PMC13033518; doi:10.3389/fimmu.2026.1773990)
Supplement: Supplementary Data Sheet 1 — Harmonized summary data, forest plots, funnel plots, data sources, harmonization details, and sensitivity analyses for the Mendelian randomization analysis of pyroptosis-related proteins and ulcerative colitis. [file DataSheet1.zip › bdpqtlresult/9215_117_USP25_UBP25/forest.pdf]

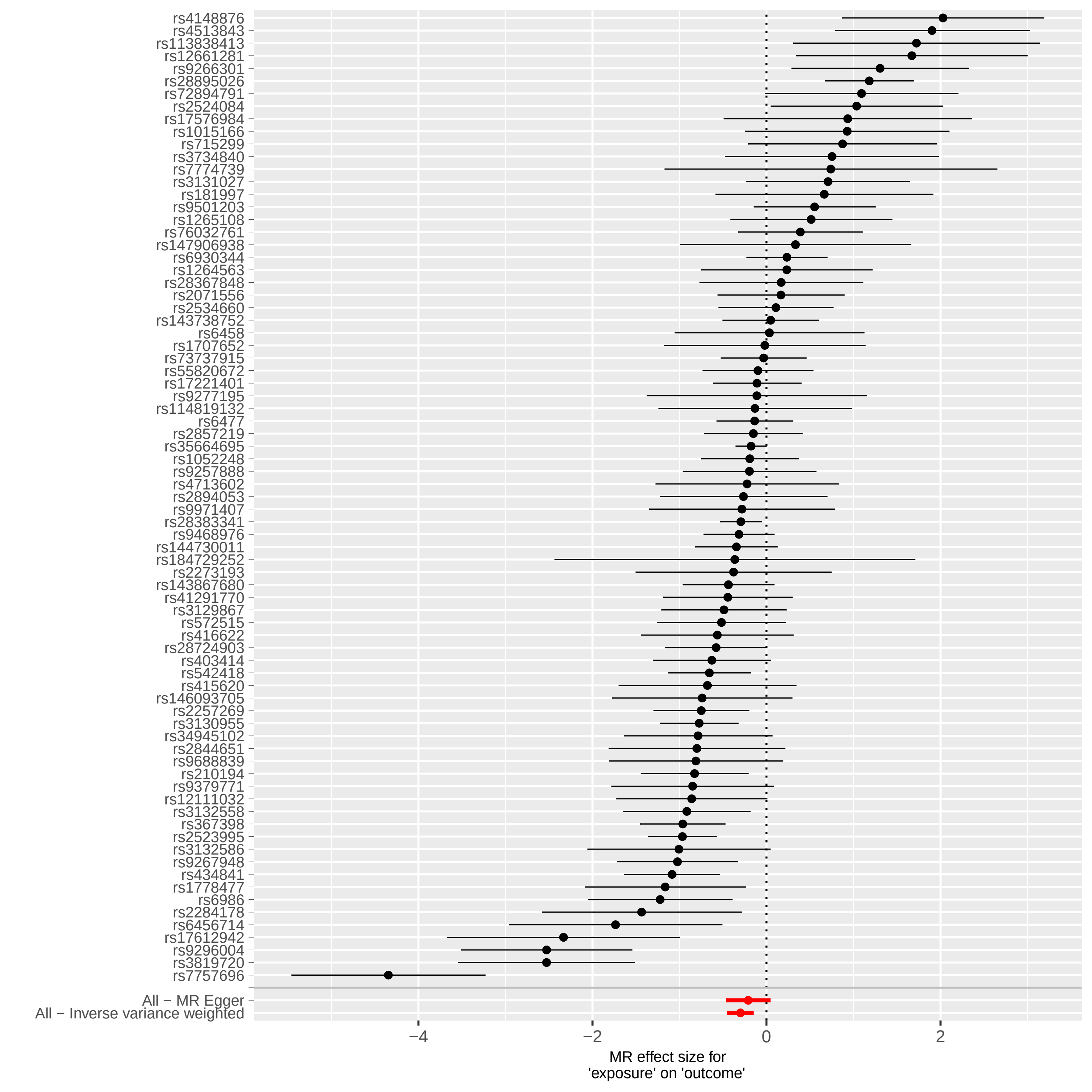
rs4148876
rs4513843
rs113838413
rs12661281
rs9266301
rs28895026
rs72894791
rs2524084
rs17576984
rs1015166
rs715299
rs3734840
rs7774739
rs3131027
rs181997
rs9501203
rs1265108
rs76032761
rs147906938
rs6930344
rs1264563
rs28367848
rs2071556
rs2534660
rs143738752
rs6458
rs1707652
rs73737915
rs55820672
rs17221401
rs9277195
rs114819132
rs6477
rs2857219
rs35664695
rs1052248
rs9257888
rs4713602
rs2894053
rs9971407
rs28383341
rs9468976
rs144730011
rs184729252
rs2273193
rs143867680
rs41291770
rs3129867
rs572515
rs416622
rs28724903
rs403414
rs542418
rs415620
rs146093705
rs2257269
rs3130955
rs34945102
rs2844651
rs9688839
rs210194
rs9379771
rs12111032
rs3132558
rs367398
rs2523995
rs3132586
rs9267948
rs434841
rs1778477
rs6986
rs2284178
rs6456714
rs17612942
rs9296004
rs3819720
rs7757696
All − MR Egger
All − Inverse variance weighted
−4
−2
0
2
MR effect size for
'exposure' on 'outcome'
