# Supplementary material for: Integrative Mendelian randomization and experimental validation prioritize KLF4 in the gut microbiota–pyroptosis–barrier axis of ulcerative colitis
Source: Front Immunol. 2026 Mar 16;17:1773990. doi: 10.3389/fimmu.2026.1773990 (PMC13033518; doi:10.3389/fimmu.2026.1773990)
Supplement: Supplementary Data Sheet 1 — Harmonized summary data, forest plots, funnel plots, data sources, harmonization details, and sensitivity analyses for the Mendelian randomization analysis of pyroptosis-related proteins and ulcerative colitis. [file DataSheet1.zip › bdpqtlresult/9215_117_USP25_UBP25/scatter.pdf]

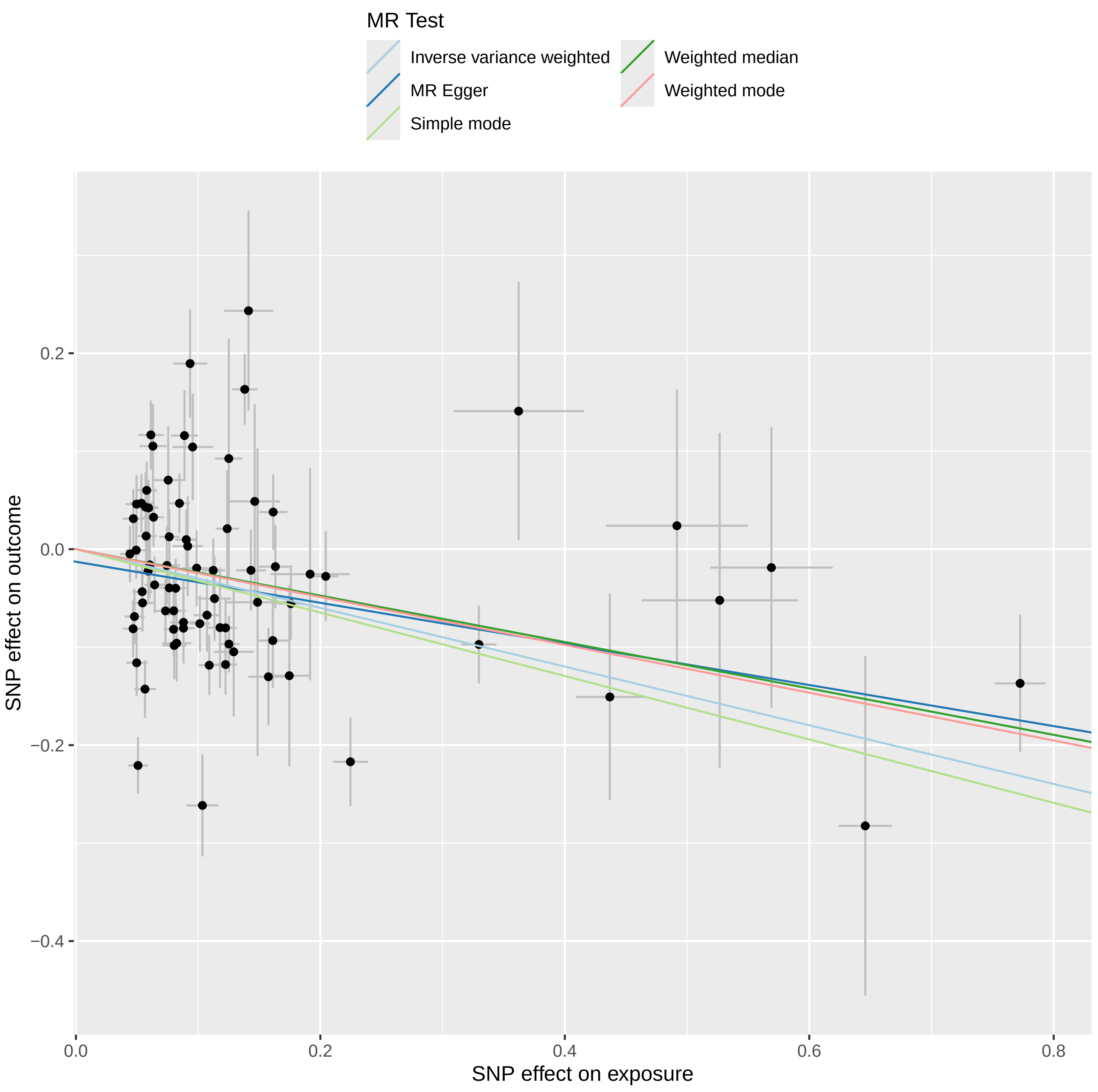
MR Test
Inverse variance weighted
MR Egger
Simple mode
Weighted median
Weighted mode
0.2
0.0
−0.2
−0.4
SNP effect on outcome
0.0
0.2
0.4
0.6
0.8
SNP effect on exposure
