# Supplementary material for: Integrative Mendelian randomization and experimental validation prioritize KLF4 in the gut microbiota–pyroptosis–barrier axis of ulcerative colitis
Source: Front Immunol. 2026 Mar 16;17:1773990. doi: 10.3389/fimmu.2026.1773990 (PMC13033518; doi:10.3389/fimmu.2026.1773990)
Supplement: Supplementary Data Sheet 1 — Harmonized summary data, forest plots, funnel plots, data sources, harmonization details, and sensitivity analyses for the Mendelian randomization analysis of pyroptosis-related proteins and ulcerative colitis. [file DataSheet1.zip › bdpqtlresult/9215_117_USP25_UBP25/sensitivity-analysis.pdf]

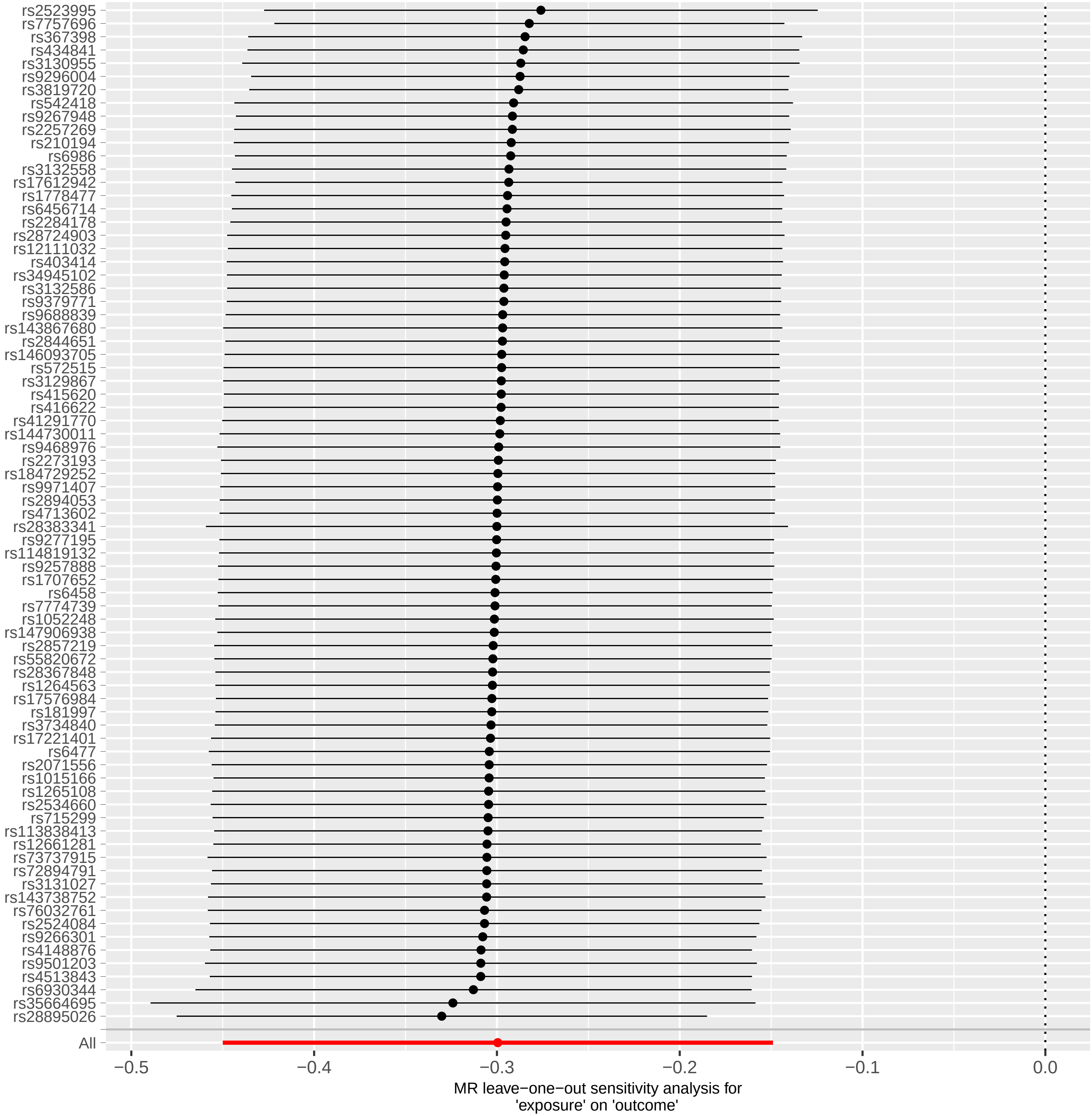
rs2523995
rs7757696
rs367398
rs434841
rs3130955
rs9296004
rs3819720
rs542418
rs9267948
rs2257269
rs210194
rs6986
rs3132558
rs17612942
rs1778477
rs6456714
rs2284178
rs28724903
rs12111032
rs403414
rs34945102
rs3132586
rs9379771
rs9688839
rs143867680
rs2844651
rs146093705
rs572515
rs3129867
rs415620
rs416622
rs41291770
rs144730011
rs9468976
rs2273193
rs184729252
rs9971407
rs2894053
rs4713602
rs28383341
rs9277195
rs114819132
rs9257888
rs1707652
rs6458
rs7774739
rs1052248
rs147906938
rs2857219
rs55820672
rs28367848
rs1264563
rs17576984
rs181997
rs3734840
rs17221401
rs6477
rs2071556
rs1015166
rs1265108
rs2534660
rs715299
rs113838413
rs12661281
rs73737915
rs72894791
rs3131027
rs143738752
rs76032761
rs2524084
rs9266301
rs4148876
rs9501203
rs4513843
rs6930344
rs35664695
rs28895026
All
−0.5
−0.4
−0.3
−0.2
−0.1
0.0
MR leave−one−out sensitivity analysis for
'exposure' on 'outcome'
