# Supplementary material for: Integrative Mendelian randomization and experimental validation prioritize KLF4 in the gut microbiota–pyroptosis–barrier axis of ulcerative colitis
Source: Front Immunol. 2026 Mar 16;17:1773990. doi: 10.3389/fimmu.2026.1773990 (PMC13033518; doi:10.3389/fimmu.2026.1773990)
Supplement: Supplementary Data Sheet 1 — Harmonized summary data, forest plots, funnel plots, data sources, harmonization details, and sensitivity analyses for the Mendelian randomization analysis of pyroptosis-related proteins and ulcerative colitis. [file DataSheet1.zip › bdpqtlresult/9312_8_AZGP1_AZGP1/forest.pdf]

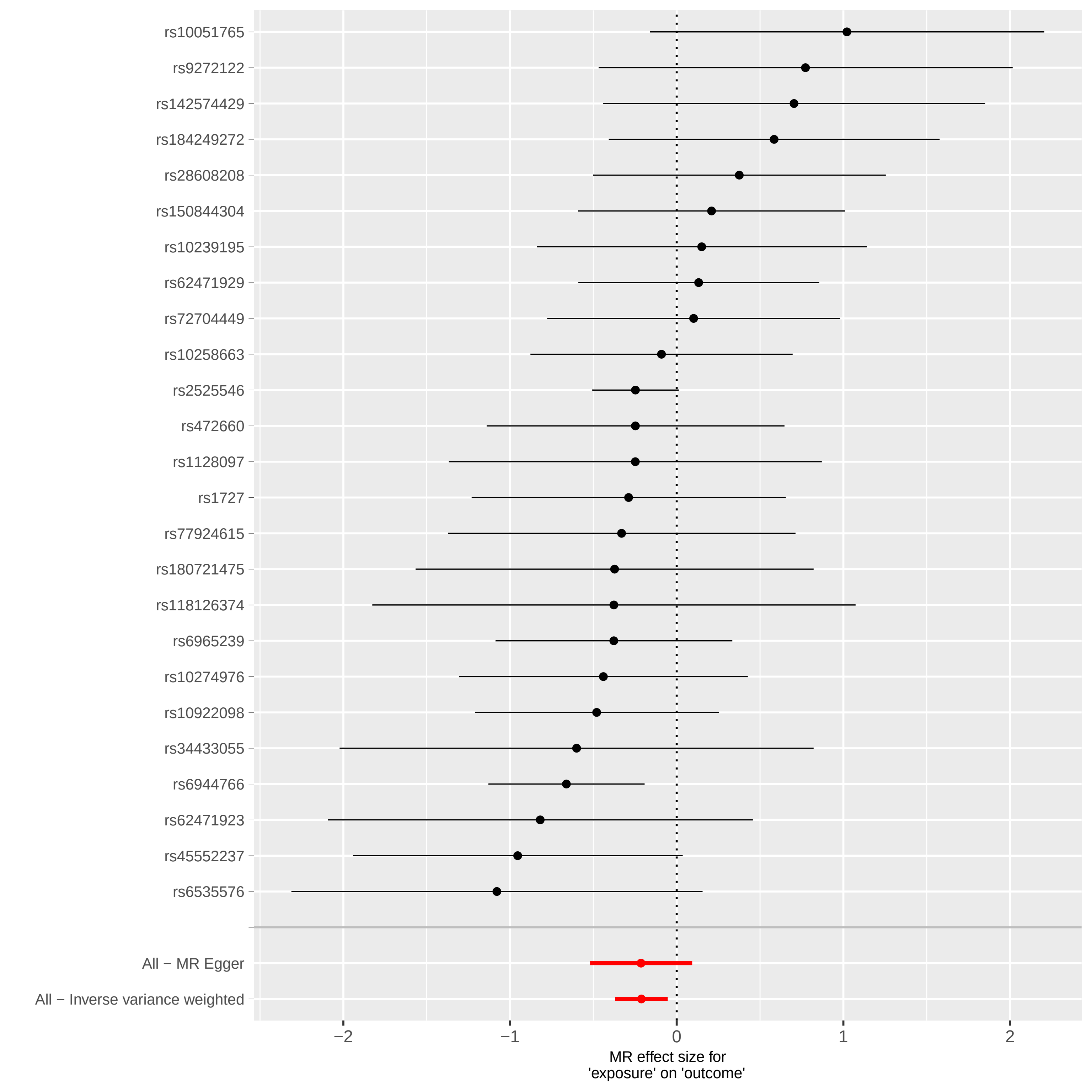

rs10051765
rs9272122
rs142574429
rs184249272
rs28608208
rs150844304
rs10239195
rs62471929
rs72704449
rs10258663
rs2525546
rs472660
rs1128097
rs1727
rs77924615
rs180721475
rs118126374
rs6965239
rs10274976
rs10922098
rs34433055
rs6944766
rs62471923
rs45552237
rs6535576
All − MR Egger
All − Inverse variance weighted
−2
−1
0
1
2
MR effect size for
'exposure' on 'outcome'
