# Supplementary material for: Integrative Mendelian randomization and experimental validation prioritize KLF4 in the gut microbiota–pyroptosis–barrier axis of ulcerative colitis
Source: Front Immunol. 2026 Mar 16;17:1773990. doi: 10.3389/fimmu.2026.1773990 (PMC13033518; doi:10.3389/fimmu.2026.1773990)
Supplement: Supplementary Data Sheet 1 — Harmonized summary data, forest plots, funnel plots, data sources, harmonization details, and sensitivity analyses for the Mendelian randomization analysis of pyroptosis-related proteins and ulcerative colitis. [file DataSheet1.zip › bdpqtlresult/9312_8_AZGP1_AZGP1/sensitivity-analysis.pdf]

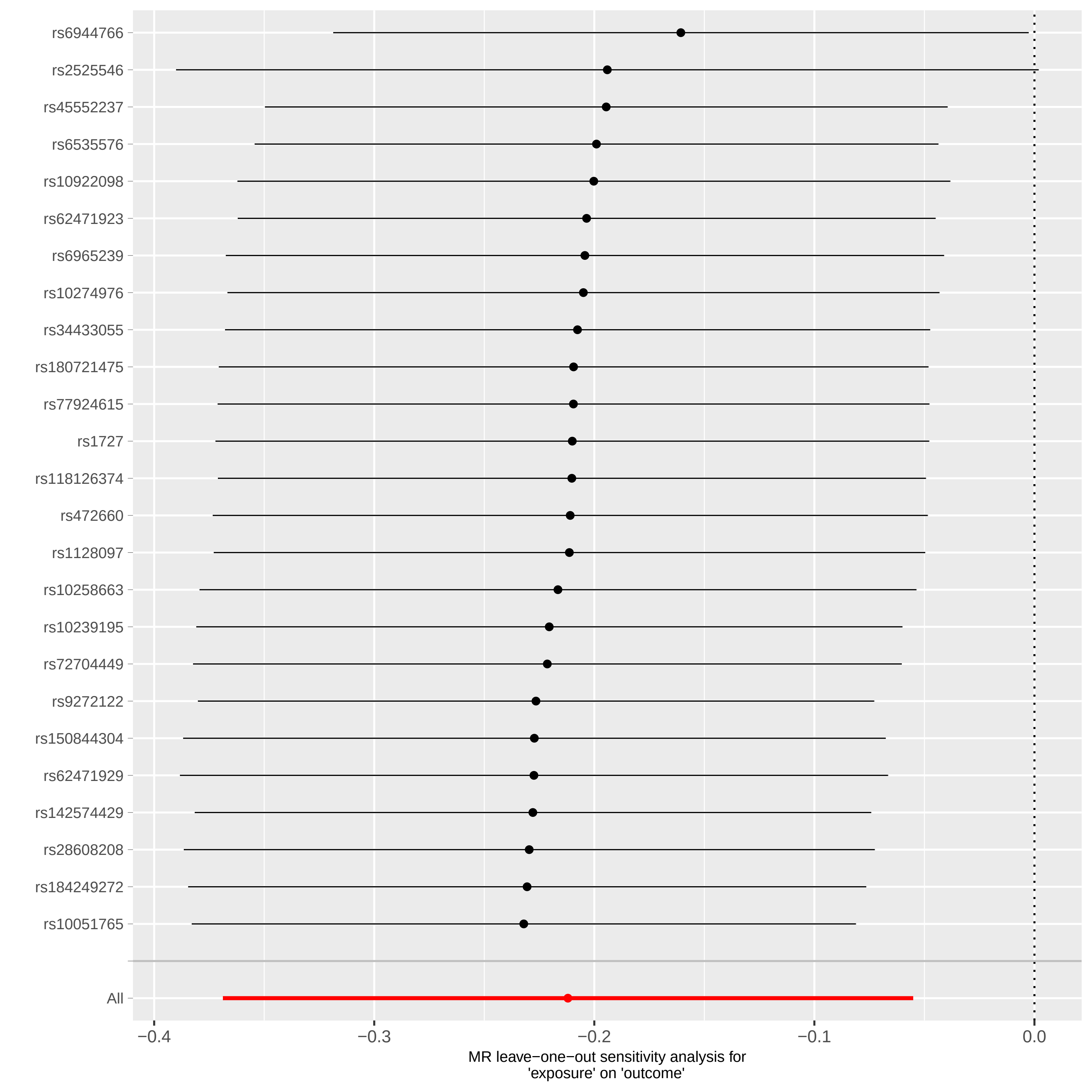
rs6944766
rs2525546
rs45552237
rs6535576
rs10922098
rs62471923
rs6965239
rs10274976
rs34433055
rs180721475
rs77924615
rs1727
rs118126374
rs472660
rs1128097
rs10258663
rs10239195
rs72704449
rs9272122
rs150844304
rs62471929
rs142574429
rs28608208
rs184249272
rs10051765
All
−0.4
−0.3
−0.2
−0.1
0.0
MR leave−one−out sensitivity analysis for
'exposure' on 'outcome'
