# Supplementary material for: Integrative Mendelian randomization and experimental validation prioritize KLF4 in the gut microbiota–pyroptosis–barrier axis of ulcerative colitis
Source: Front Immunol. 2026 Mar 16;17:1773990. doi: 10.3389/fimmu.2026.1773990 (PMC13033518; doi:10.3389/fimmu.2026.1773990)
Supplement: Supplementary Data Sheet 1 — Harmonized summary data, forest plots, funnel plots, data sources, harmonization details, and sensitivity analyses for the Mendelian randomization analysis of pyroptosis-related proteins and ulcerative colitis. [file DataSheet1.zip › bdpqtlresult/9337_43_TAC1_TKN1/forest.pdf]

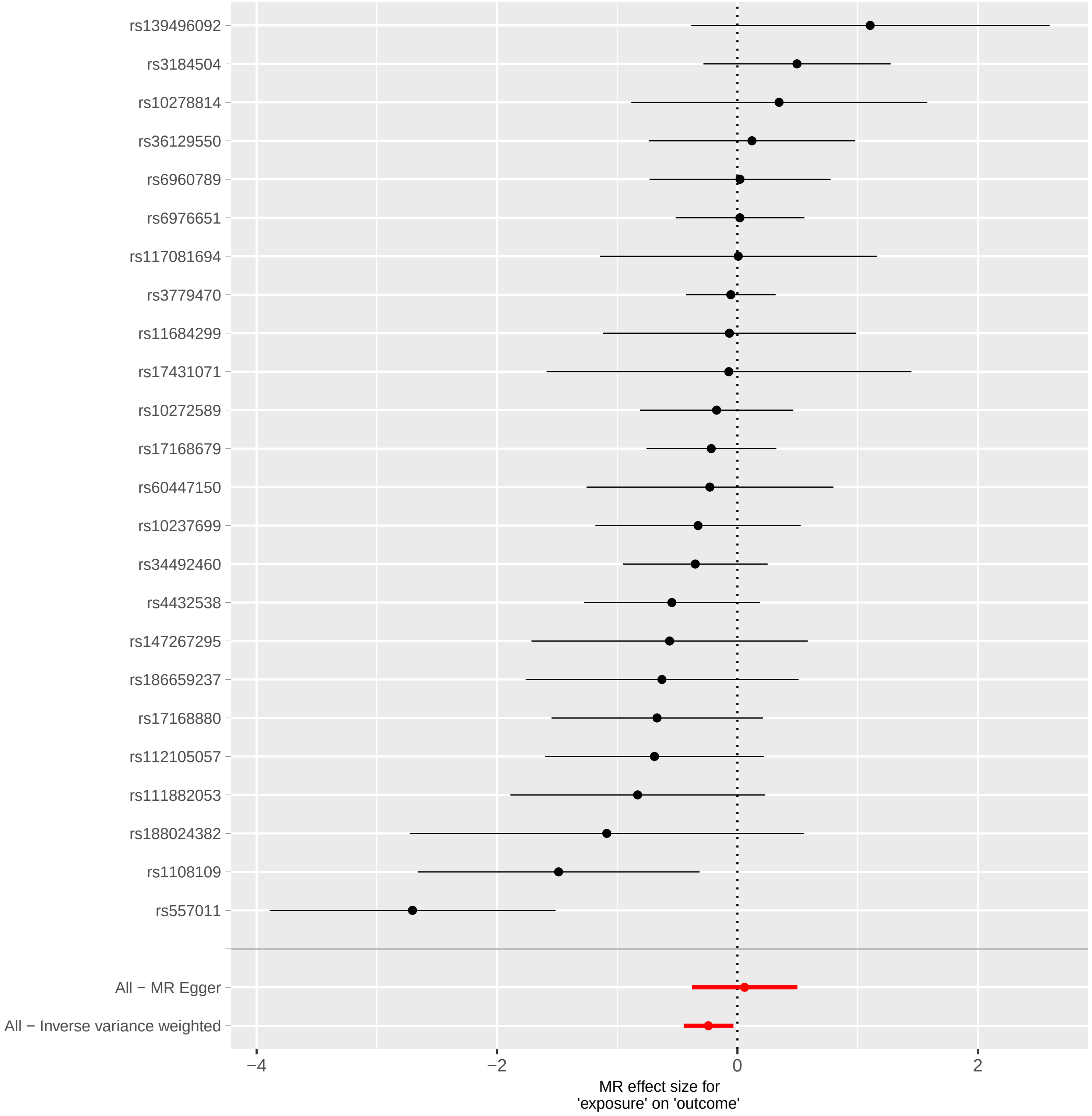

rs139496092
rs3184504
rs10278814
rs36129550
rs6960789
rs6976651
rs117081694
rs3779470
rs11684299
rs17431071
rs10272589
rs17168679
rs60447150
rs10237699
rs34492460
rs4432538
rs147267295
rs186659237
rs17168880
rs112105057
rs111882053
rs188024382
rs1108109
rs557011
All − MR Egger
All − Inverse variance weighted
−4
−2
0
2
MR effect size for
'exposure' on 'outcome'
