# Supplementary material for: Integrative Mendelian randomization and experimental validation prioritize KLF4 in the gut microbiota–pyroptosis–barrier axis of ulcerative colitis
Source: Front Immunol. 2026 Mar 16;17:1773990. doi: 10.3389/fimmu.2026.1773990 (PMC13033518; doi:10.3389/fimmu.2026.1773990)
Supplement: Supplementary Data Sheet 1 — Harmonized summary data, forest plots, funnel plots, data sources, harmonization details, and sensitivity analyses for the Mendelian randomization analysis of pyroptosis-related proteins and ulcerative colitis. [file DataSheet1.zip › bdpqtlresult/9337_43_TAC1_TKN1/sensitivity-analysis.pdf]

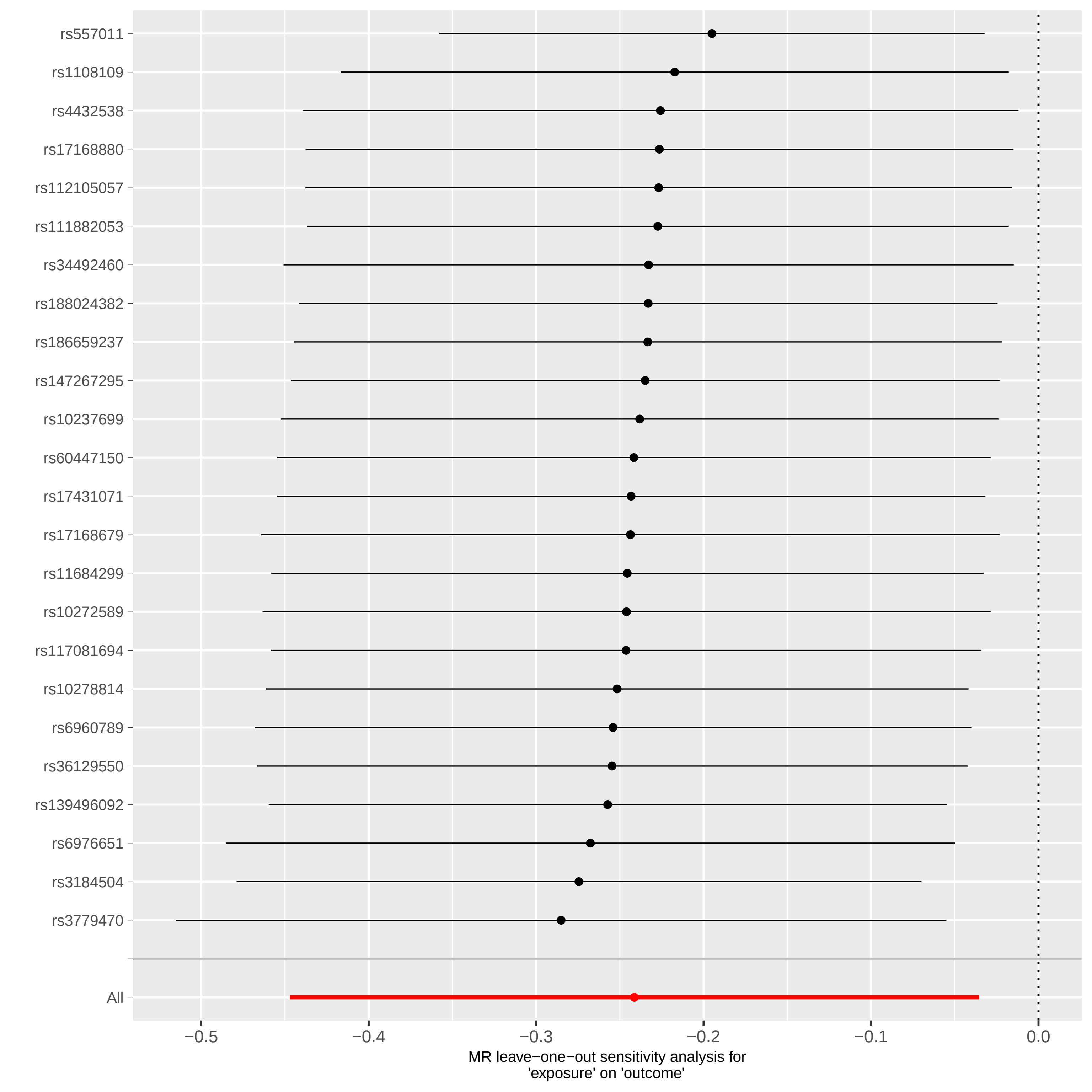

rs557011
rs1108109
rs4432538
rs17168880
rs112105057
rs111882053
rs34492460
rs188024382
rs186659237
rs147267295
rs10237699
rs60447150
rs17431071
rs17168679
rs11684299
rs10272589
rs117081694
rs10278814
rs6960789
rs36129550
rs139496092
rs6976651
rs3184504
rs3779470
All
−0.5
−0.4
−0.3
−0.2
−0.1
0.0
MR leave−one−out sensitivity analysis for
'exposure' on 'outcome'
