# Supplementary material for: Integrative Mendelian randomization and experimental validation prioritize KLF4 in the gut microbiota–pyroptosis–barrier axis of ulcerative colitis
Source: Front Immunol. 2026 Mar 16;17:1773990. doi: 10.3389/fimmu.2026.1773990 (PMC13033518; doi:10.3389/fimmu.2026.1773990)
Supplement: Supplementary Data Sheet 2 — Full GCST identifiers, taxonomic labels, and Mendelian randomization statistics for the gut microbial traits associated with ulcerative colitis. [file DataSheet2.zip › GM_result/GCST90032591/sensitivity-analysis.pdf]

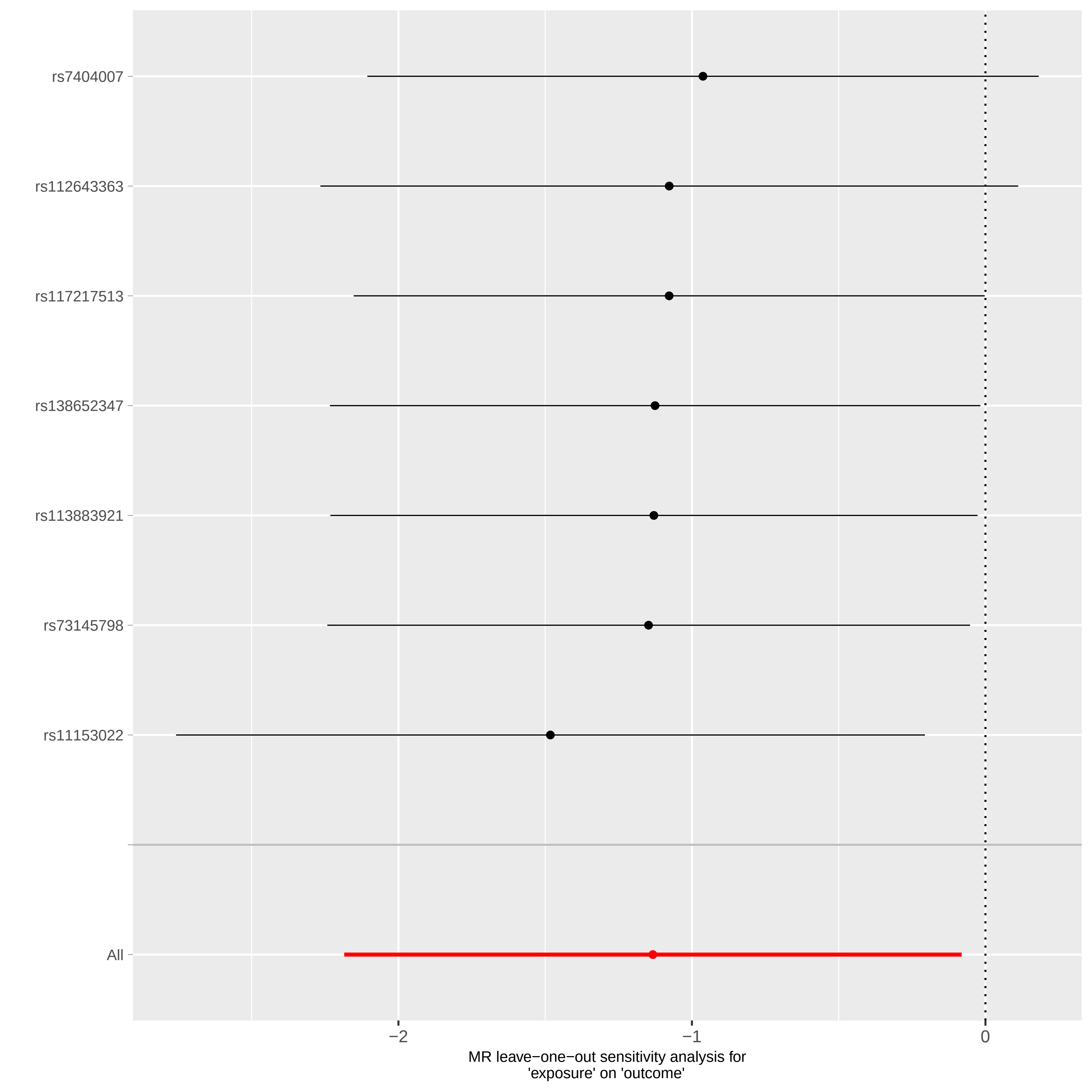
rs7404007
rs112643363
rs117217513
rs138652347
rs113883921
rs73145798
rs11153022
All
−2
−1
0
MR leave−one−out sensitivity analysis for
'exposure' on 'outcome'
