# Supplementary material for: Integrative Mendelian randomization and experimental validation prioritize KLF4 in the gut microbiota–pyroptosis–barrier axis of ulcerative colitis
Source: Front Immunol. 2026 Mar 16;17:1773990. doi: 10.3389/fimmu.2026.1773990 (PMC13033518; doi:10.3389/fimmu.2026.1773990)
Supplement: Supplementary Data Sheet 2 — Full GCST identifiers, taxonomic labels, and Mendelian randomization statistics for the gut microbial traits associated with ulcerative colitis. [file DataSheet2.zip › GM_result/GCST90032613/forest.pdf]

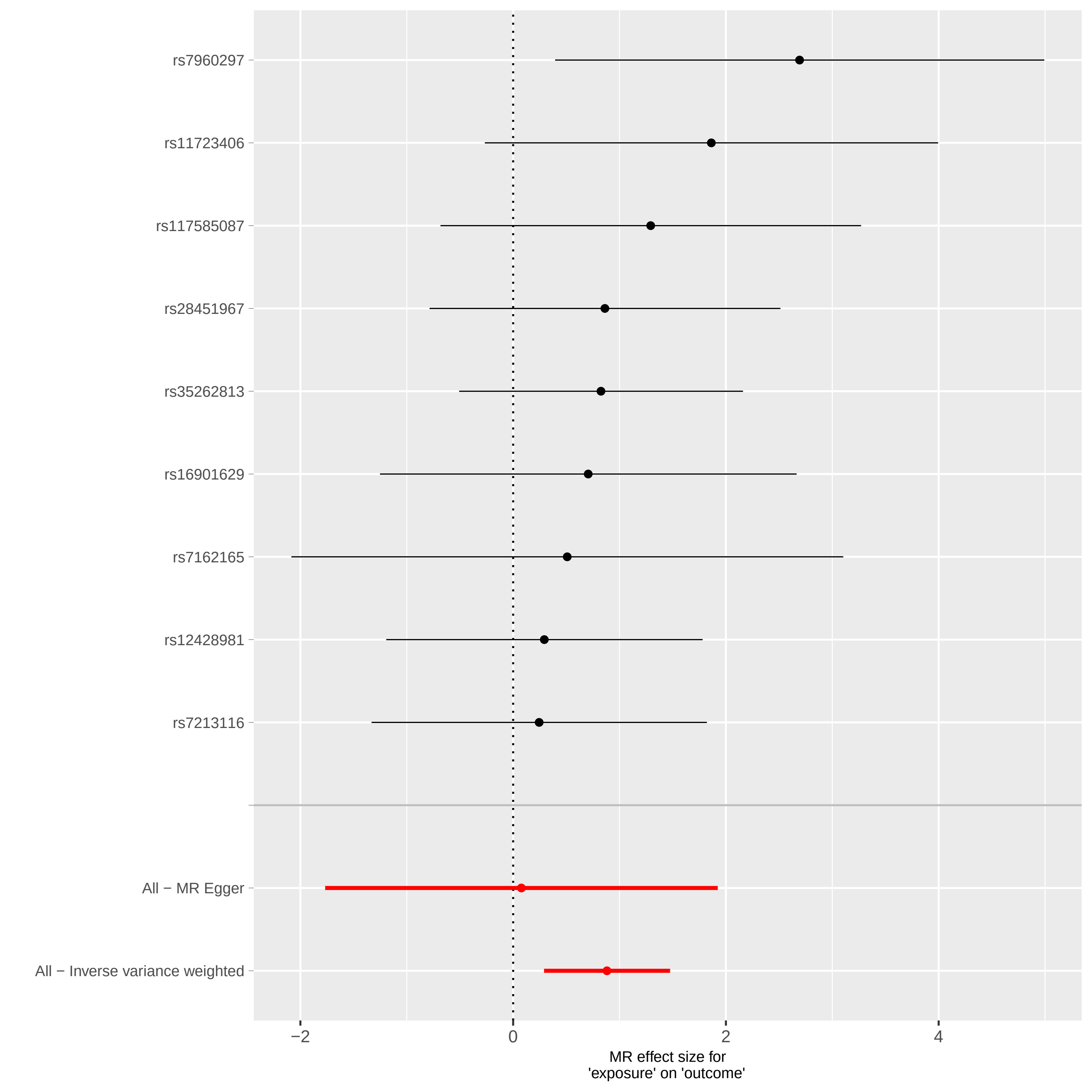

rs7960297
rs11723406
rs117585087
rs28451967
rs35262813
rs16901629
rs7162165
rs12428981
rs7213116
All − MR Egger
All − Inverse variance weighted
−2
0
2
4
MR effect size for
'exposure' on 'outcome'
