# Supplementary material for: Integrative Mendelian randomization and experimental validation prioritize KLF4 in the gut microbiota–pyroptosis–barrier axis of ulcerative colitis
Source: Front Immunol. 2026 Mar 16;17:1773990. doi: 10.3389/fimmu.2026.1773990 (PMC13033518; doi:10.3389/fimmu.2026.1773990)
Supplement: Supplementary Data Sheet 2 — Full GCST identifiers, taxonomic labels, and Mendelian randomization statistics for the gut microbial traits associated with ulcerative colitis. [file DataSheet2.zip › GM_result/GCST90032613/funnelplot.pdf]

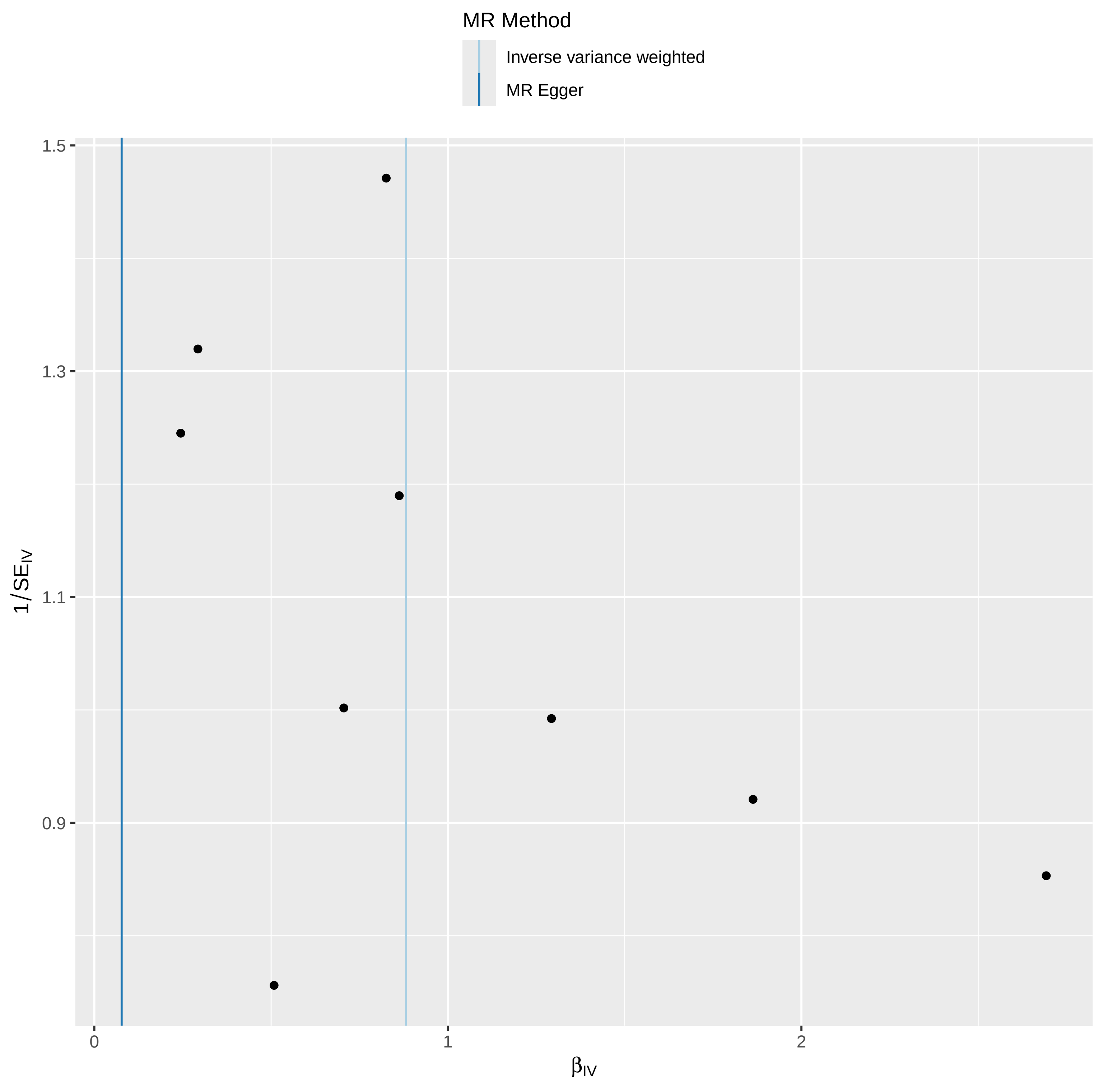
MR Method
Inverse variance weighted
MR Egger
1.5
1.3
1.1
0.9
1/SE_IV
0
1
2
β_IV
