# Supplementary material for: Integrative Mendelian randomization and experimental validation prioritize KLF4 in the gut microbiota–pyroptosis–barrier axis of ulcerative colitis
Source: Front Immunol. 2026 Mar 16;17:1773990. doi: 10.3389/fimmu.2026.1773990 (PMC13033518; doi:10.3389/fimmu.2026.1773990)
Supplement: Supplementary Data Sheet 3 — Full results of the pairwise Mendelian randomization analyses between ulcerative colitis-associated microbial taxa and ulcerative colitis-associated pyroptosis proteins, used for the downstream mediation analysis. [file DataSheet3.zip › GM_bd_fer_result/GCST90032583+3447_64_CXCL8_IL_8/scatter.pdf]

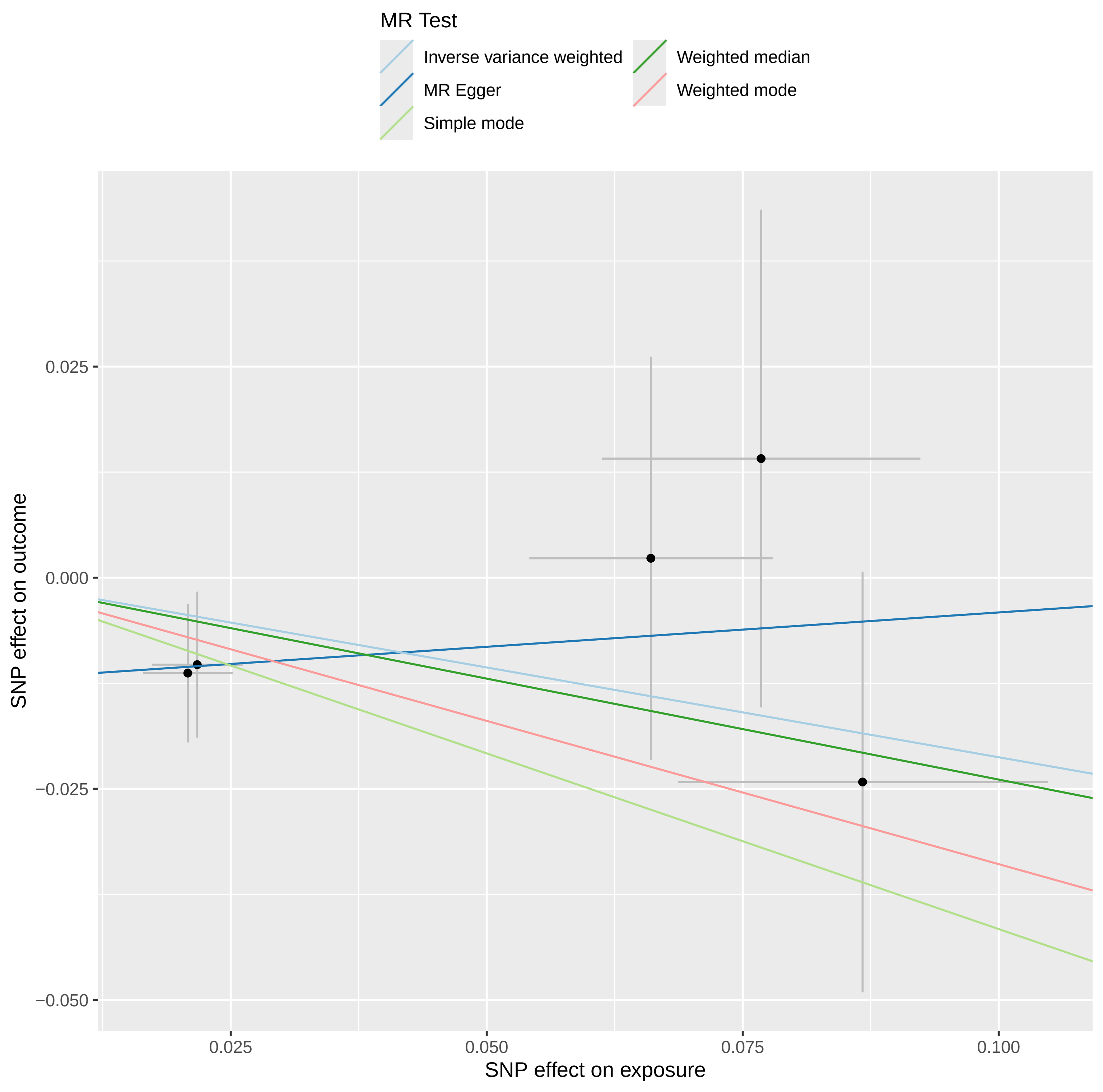
MR Test
Inverse variance weighted
MR Egger
Simple mode
Weighted median
Weighted mode
0.025
0.000
−0.025
−0.050
0.025
0.050
0.075
0.100
SNP effect on outcome
SNP effect on exposure
