# Supplementary material for: Integrative Mendelian randomization and experimental validation prioritize KLF4 in the gut microbiota–pyroptosis–barrier axis of ulcerative colitis
Source: Front Immunol. 2026 Mar 16;17:1773990. doi: 10.3389/fimmu.2026.1773990 (PMC13033518; doi:10.3389/fimmu.2026.1773990)
Supplement: Supplementary Data Sheet 3 — Full results of the pairwise Mendelian randomization analyses between ulcerative colitis-associated microbial taxa and ulcerative colitis-associated pyroptosis proteins, used for the downstream mediation analysis. [file DataSheet3.zip › GM_bd_fer_result/GCST90032583+4911_49_GSTP1_Glutathione_S_transferase_Pi/forest.pdf]

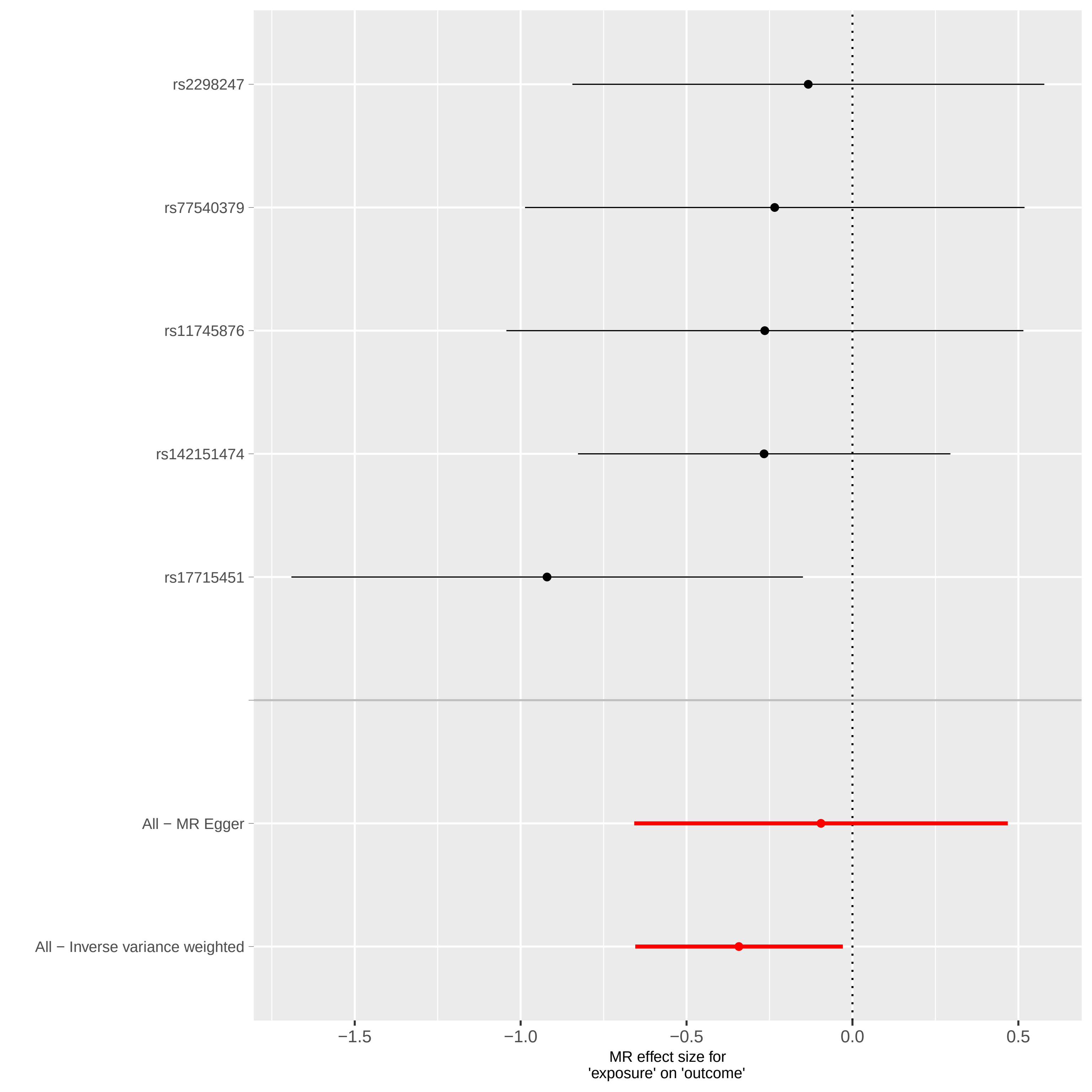
rs2298247
rs77540379
rs11745876
rs142151474
rs17715451
All − MR Egger
All − Inverse variance weighted
−1.5
−1.0
−0.5
0.0
0.5
MR effect size for
'exposure' on 'outcome'
