# Supplementary material for: Integrative Mendelian randomization and experimental validation prioritize KLF4 in the gut microbiota–pyroptosis–barrier axis of ulcerative colitis
Source: Front Immunol. 2026 Mar 16;17:1773990. doi: 10.3389/fimmu.2026.1773990 (PMC13033518; doi:10.3389/fimmu.2026.1773990)
Supplement: Supplementary Data Sheet 3 — Full results of the pairwise Mendelian randomization analyses between ulcerative colitis-associated microbial taxa and ulcerative colitis-associated pyroptosis proteins, used for the downstream mediation analysis. [file DataSheet3.zip › GM_bd_fer_result/GCST90032583+4911_49_GSTP1_Glutathione_S_transferase_Pi/sensitivity-analysis.pdf]

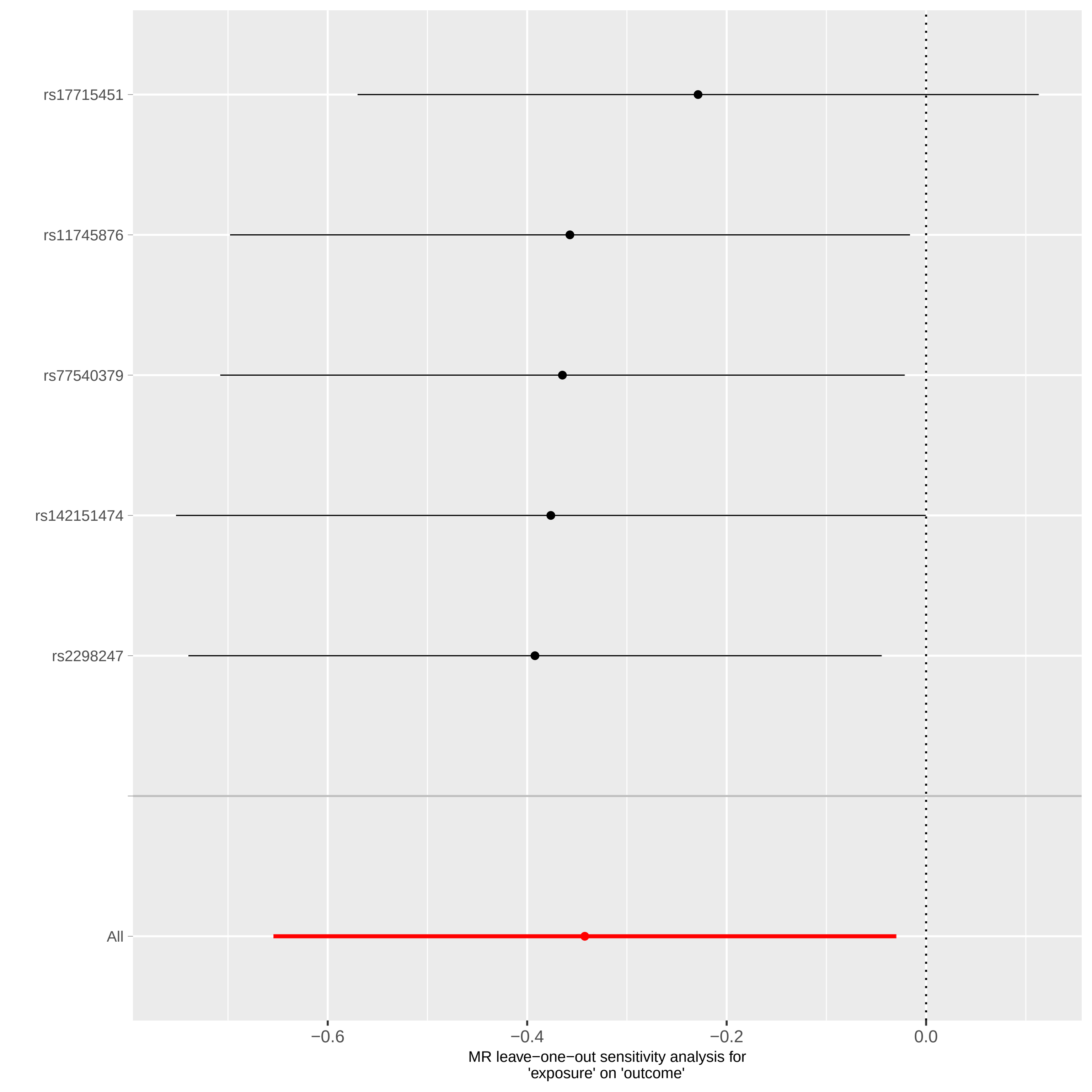
rs17715451
rs11745876
rs77540379
rs142151474
rs2298247
All
−0.6
−0.4
−0.2
0.0
MR leave−one−out sensitivity analysis for
'exposure' on 'outcome'
