# Supplementary material for: Integrative Mendelian randomization and experimental validation prioritize KLF4 in the gut microbiota–pyroptosis–barrier axis of ulcerative colitis
Source: Front Immunol. 2026 Mar 16;17:1773990. doi: 10.3389/fimmu.2026.1773990 (PMC13033518; doi:10.3389/fimmu.2026.1773990)
Supplement: Supplementary Data Sheet 3 — Full results of the pairwise Mendelian randomization analyses between ulcerative colitis-associated microbial taxa and ulcerative colitis-associated pyroptosis proteins, used for the downstream mediation analysis. [file DataSheet3.zip › GM_bd_fer_result/GCST90032583+5004_69_MAPK11_MK11/funnelplot.pdf]

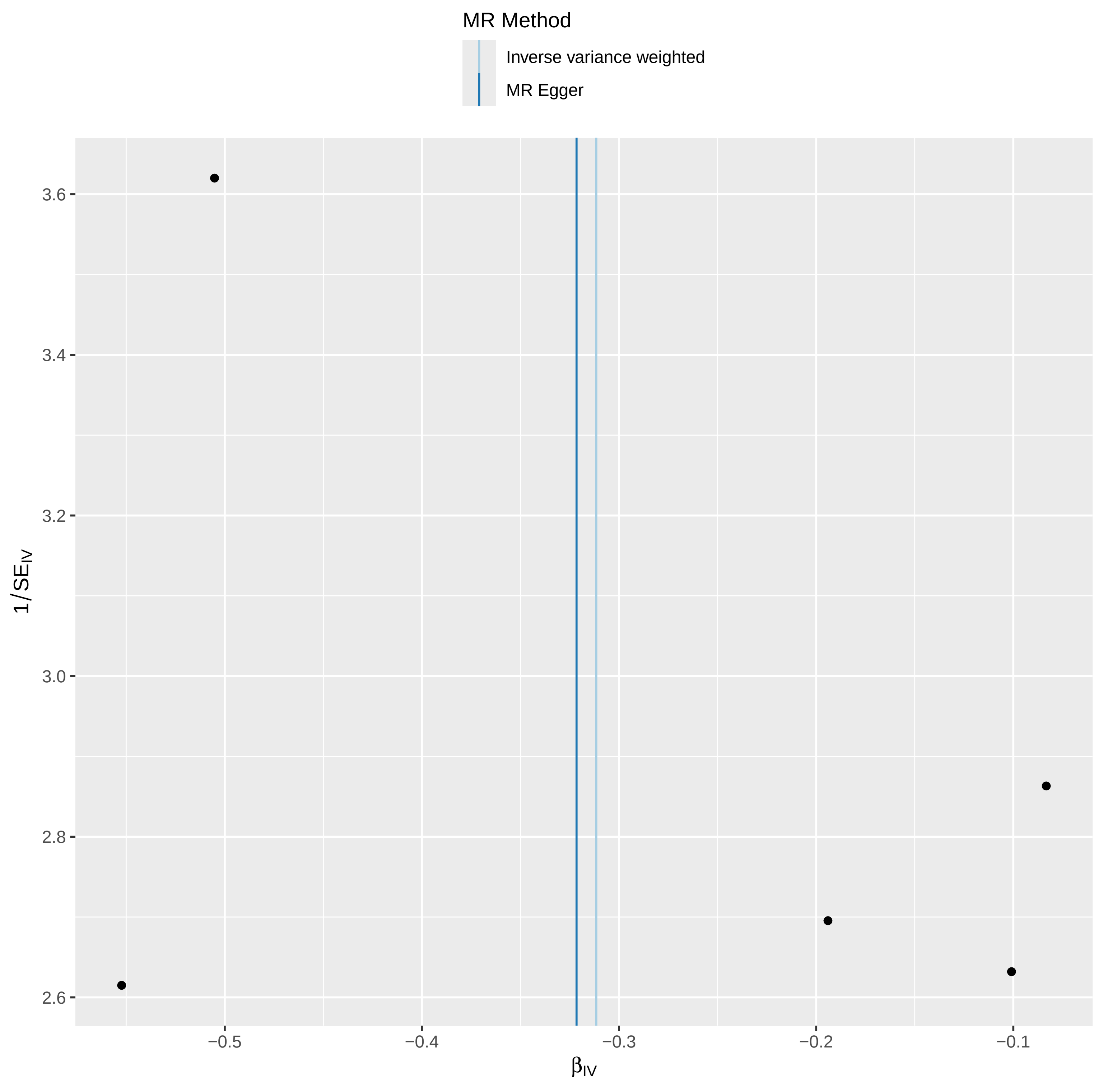
MR Method
Inverse variance weighted
MR Egger
3.6
3.4
3.2
3.0
2.8
2.6
$1/SE_{IV}$
−0.5
−0.4
−0.3
−0.2
−0.1
β_{IV}
